# Angelica polysaccharides relieve blood glucose levels in diabetic KKAy mice possibly by modulating gut microbiota: an integrated gut microbiota and metabolism analysis

**DOI:** 10.1186/s12866-023-03029-y

**Published:** 2023-10-03

**Authors:** Xiaolong Tang, Lixia Yang, Yandong Miao, Wuhua Ha, Zheng Li, Denghai Mi

**Affiliations:** 1 The First Clinical Medical College, Lanzhou University, Lanzhou City, Gansu Province China; 2 The Second Department of Gastrointestinal Surgery, Affiliated Hospital of North Sichuan Medical College, Sichuan Province, Nanchong City, China; 3 Gansu Academy of Traditional Chinese Medicine, Lanzhou City, Gansu Province China; 4 Department of Oncology, Yantai Affiliated Hospital of Binzhou Medical University, The Second Clinical Medical College of Binzhou Medical University, Yantai City, Shandong Province China; 5 Department of Radiotherapy, Cancer Center, West China Hospital of Sichuan University, Chengdu, Sichuan Province China

**Keywords:** Type 2 diabetes, Gut microbiota, Metagenomics, Metabolomics, 16S *rRNA* gene sequences

## Abstract

**Background:**

Angelica polysaccharides (AP) have numerous benefits in relieving type 2 diabetes (T2D). However, the underlying mechanisms have yet to be fully understood. Recent many reports have suggested that altering gut microbiota can have adverse effects on the host metabolism and contribute to the development of T2D. Here, we successfully established the T2D model using the male KKAy mice with high-fat and high-sugar feed. Meanwhile, the male C57BL/6 mice were fed with a normal feed. T2D KKAy mice were fed either with or without AP supplementation. In each group, we measured the mice's fasting blood glucose, weight, and fasting serum insulin levels. We collected the cecum content of mice, the gut microbiota was analyzed by targeted full-length 16S *rRNA* metagenomic sequencing and metabolites were analyzed by untargeted-metabolomics.

**Results:**

We found AP effectively alleviated glycemic disorders of T2D KKAy mice, with the changes in gut microbiota composition and function. Many bacteria species and metabolites were markedly changed in T2D KKAy mice and reversed by AP. Additionally, 16 altered metabolic pathways affected by AP were figured out by combining metagenomic pathway enrichment analysis and metabolic pathway enrichment analysis. The key metabolites in 16 metabolic pathways were significantly associated with the gut microbial alteration. Together, our findings showed that AP supplementation could attenuate the diabetic phenotype. Significant gut microbiota and gut metabolite changes were observed in the T2D KKAy mice and AP intervention.

**Conclusions:**

Administration of AP has been shown to improve the composition of intestinal microbiota in T2D KKAy mice, thus providing further evidence for the potential therapeutic application of AP in the treatment of T2D.

**Supplementary Information:**

The online version contains supplementary material available at 10.1186/s12866-023-03029-y.

## Introduction

Type 2 diabetes (T2D), in particular, is now acknowledged as a major metabolic illness that endangers public health and is viewed as a critical public health issue in all countries [1]. The role of gut microbiota in metabolic disorders has recently attracted much attention. The gut microbiota is a complex microbial community made up of highly interactive bacteria with a strong link to their host [2]. Through decreased glucose tolerance and increased insulin resistance, the gut microbiota has been attributed to the development of obesity, metabolic syndrome, and T2D [3]. A cross-sectional study based on 1,495 people showed that in the groups with impaired glucose tolerance, combined glucose intolerance, and T2D, the overall gut microbial composition was altered. Furthermore, both in the prediabetes and T2D groups, the quantity of several butyrate producers and their functional capability for butyrate generation were reduced [4]. The previous report indicated that butyrate has emerged as a promising therapeutic target for T2D. Therapy techniques have been established to boost intestinal levels, such as butyrate-producing bacteria, dietary fiber supplements, or fecal microbiota transplantation [5]. Through a randomized clinical trial with 450 T2D patients, Tong et al. (2018) found that changes of gut microbiota may alleviate T2D with hyperlipidemia through metformin and some specifically designed herbal formula [6]. Yu et al. (2019) reported that by using C57BL/Ks (db/db) male mice as a T2D mouse model, they discovered that the abundances of gut bacteria were altered compared with the m/m male mice. Moreover, when fecal bacteria from db/db and m/m mice were transplanted into pseudo-germ-free mice, metabolic parameters changed significantly, suggesting that changes may aid the development of T2D in the gut microbiota's composition [7]. Therefore, changes in the gut microbiota composition may play a vital role in developing T2D, and prospective therapeutic techniques for gut microbiota may benefit patients with T2D.

Plant polysaccharides are macromolecules. Most of them cannot be digested by human digestive enzymes. Short-chain fatty acids (SCFAs) such as butyrate, acetate, and propionate can be produced by microbes by metabolizing these polysaccharides [8]. Angelica polysaccharide (AP) is the primary active ingredient of Dang Gui. Dang Gui, also known as *Angelica sinensis* (Oliv.) Diels, is a perennial herbaceous plant that is mainly produced in the southeastern part of Gansu, China, and has strong adaptability at an altitude of 1500–2000 m [9]. It can grow to a height of about 0.4–1 m. Through an experiment in vivo, Wang et al. (2016) reported that AP has shown certain beneficial effects on hyperglycemia, insulin secretion, hepatic glycogen synthesis, adipokine release, and liver fat accumulation by regulating the PI3K-Akt pathway [10]. However, it has not been determined whether AP affects diabetic metabolism through gut microbiota.

In this study, we explored the treatment effect of AP in the glycemic disorder of KKAy mice, a model of T2D, with a high-fructose diet (HFD). We did 16S full-length *rRNA* sequencing, metagenomic sequencing, and liquid chromatography-tandem mass spectrometry (LC–MS/MS) to analyze the bacteria composition and metabolomics. Our findings indicated that AP efficiently restored HFD-induced alterations in gut microbial composition, function, and gut metabolites. In addition, combining metagenomic pathway enrichment analysis and metabolites pathway enrichment analysis, we discovered 16 common metabolism pathways, which might be crucial for the treatment effects in regulating blood sugar disorder by AP. The 16 metabolism pathways contained 11 differential metabolites. Based on the correlation analysis, we found many bacterial species that changed by AP significantly related to the 11 metabolites, suggesting gut microbiota may be a critical element in the anti-diabetes effect of AP. In conclusion, based on 16S full-length *rRNA* sequencing, metagenomic sequencing, and LC–MS/MS, we provided new evidence for AP regulating blood glucose disorders from a gut microbiota perspective.

## Materials and methods

### Preparation of AP extracts

Yang Lingci edge biotechnology co., LTD (Room 3–316, Chuangye Building, No. 16 shennong Road, Yangling Demonstration Zone, Shaanxi Province) supplied AP with 90% purity. The polysaccharides were extracted from *Angelica Sinensis* by boiling distilled water then followed by precipitation by 70% ethanol. It was then deprotenised and dialysis, the crude polysaccharide extract was deproteinized using the Sevag method, then lyophilized for the subsequent experiment [11].

### Animals

Beijing Huafukang Biological Technology Co., LTD provided male KKAy mice (10–12-week-old, mean body weight 35 g) and male C57BL/6 mice (10–12-week-old, mean body weight 20 g), License Number: SCXK (Beijing) 2019–0008. In this study, KKAy mice were used as a T2D model, while C57BL/6 mice were used as the control. KKAy mice are commonly used as a T2D animal model due to their genetic predisposition to develop insulin resistance and obesity [12]. During a 12 h light and 12 h dark period cycles, all mice were kept in a temperature-controlled environment with food and water readily available. All of the studies were performed as per the Chinese National Institutes of Health's Guide for the Care and Use of Laboratory Animals and were approved by Gansu Traditional Chinese Medicine University's Animal Research Ethics Committee. The approval reference number for this study is 2021–368. We adhered strictly to ethical standards for animal experimentation, ensuring that housing and care conditions met relevant welfare standards. To minimize animal distress and discomfort during the experimental process, we employed a range of measures, including the use of anesthetics and sedatives. Upon conclusion of the experiments, all animals were treated in a humane manner in accordance with the ARRIVE guidelines [13].

### Experimental design

**Fig. 1:**
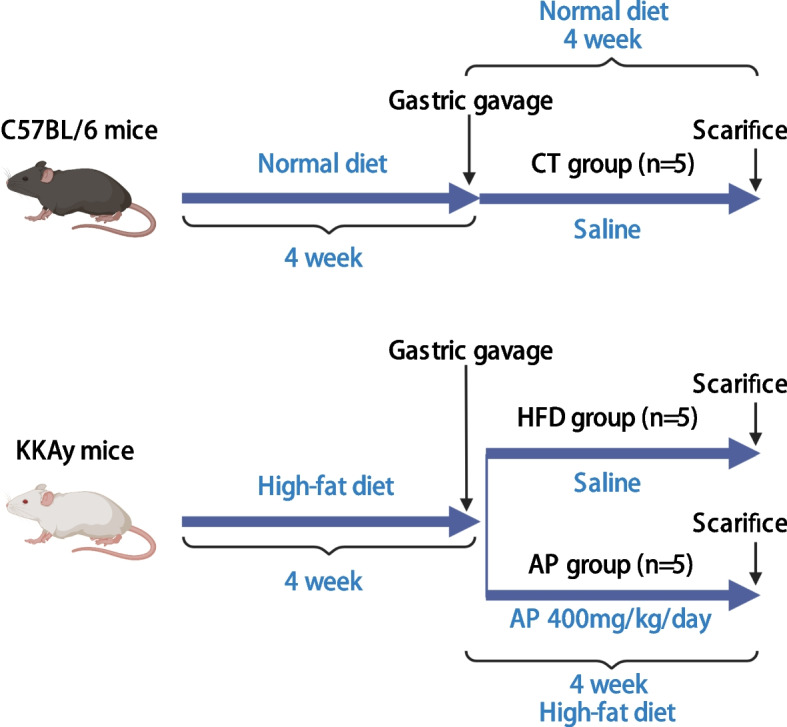
The flow chart of the experimental design for all groups

Male KKAy mice aged 10–12 weeks were initially selected for the study. A blood glucose meter (Gansu Yinhao Biotechnology Co., LTD) was used to analyze the FBG. Mouse with FBG levels exceeding 7 mmol/l after a 4-week high-fat, high-sugar diet was deemed diabetic and included in the study [14]. Mouse with discernible health issues unrelated to diabetes or showing signs of extreme stress was excluded. The same age and health criteria were applied to male C57BL/6 mice, which served as controls and were maintained on a standard chow diet. The KKAy mice were fed a high-fat, high-sugar diet specifically designed according to the nutritional requirements of KKAy mice, which is useful for establishing obesity and T2D models. This diet was provided by Beijing Huafukang Biological Technology Co., LTD. The diet's caloric distribution was as follows: 16.46% of the calories were derived from protein, 45.65% from fat, and 37.89% from carbohydrates, with a total energy content of 4.25 kcal/g. The C57BL/6 mice were fed a regular mice diet, with 23.07% of the calories derived from protein, 11.85% from fat, and 65.08% from carbohydrates, providing a total energy content of 3.40 kcal/g. Following the establishment of diabetes (FBG > 7 mmol/l), the KKAy mice were randomly allocated into two groups (*n* = 5 each group): the HFD group and the AP group. The randomization was performed using a random number generator to ensure unbiased group assignment. This randomization process was performed by a research assistant not involved in data collection or analysis to maintain blinding. Male C57BL/6 mice (*n* = 5) served as the CT group. The sample size of 5 mice per group was chosen based on prior power analysis, ensuring adequate statistical power for detecting meaningful differences between groups while minimizing the number of animals used. It was also supported by previous research indicating effective group sizes in similar studies [11]. For the next 4 weeks, the KKAy mice continued their respective diets while the C57BL/6 mice remained on standard chow. We opted for a daily oral dose of 400 mg/kg AP for the AP group, based on prior research suggesting the greatest metabolic protective effect at this dosage compared to 200 mg/kg and 100 mg/kg [10]. The CT and HFD groups received an equivalent volume of saline. The administration of AP and saline was performed orally using gastric gavage. The gastric gavage procedure in mice, adhering to strict ethical guidelines, involves the following steps: The mice were first comfortably restrained to minimize stress. A gavage solution, such as AP or saline, was prepared according to the appropriate concentration and volume. A suitable gavage needle or tube was selected and lubricated for easy insertion. The needle or tube was gently inserted into the mice’s mouth, directed towards the esophagus. The predetermined volume of the gavage solution was then slowly and steadily administered, ensuring a gentle and controlled delivery. After complete administration, the gavage needle or tube was carefully removed. Post-gavage, the mice were monitored for any immediate signs of distress or complications. Proper disposal of used gavage equipment was carried out in accordance with biohazard waste guidelines. These steps ensure that the gastric gavage procedure was conducted with utmost consideration for the mice’s welfare and well-being, aligning with ethical principles in animal research. During this study, FBG levels and body weights of the various groups were measured weekly. FBG levels were assessed using glucose meter kits after the mice had fasted for 12 h. Blood samples were collected from the tail vein of the mice. To facilitate blood flow, we temporarily immersed the mouse’s tail in warm water, and the blood was drawn only from the tail tips. After completion of the blood collection, the puncture site was disinfected and pressure was applied to achieve hemostasis. Since the required blood volume for FBG measurement was minimal, and the associated trauma was minimal as well, anaesthesia was not administered. Despite the absence of local anaesthesia, the procedure was executed swiftly and meticulously to minimize potential discomfort and stress for the mice. An electric balance was used to investigate the mice’s weights. The experimental flowchart is summarized in Fig. 1.

### Sample collection

At the conclusion of the experiment, mice were anesthetized by intraperitonial injection of 1% pentobarbital sodium solution prior to being humanely euthanized. Before the euthanasia, we carefully collected blood from the eyeball while the mice were under anaesthesia to minimize discomfort and stress. The blood was collected into a heparin-coated syringe to prevent coagulation. Dissection was carried out promptly after the euthanasia. All these procedures were performed with the utmost care to minimize distress to the mice and were strictly in accordance with our institution's animal care guidelines and approved protocol. After piercing the end of the cecum with sterile scissors, cecum contents were squeezed out with sterile gloves and collected in sterile tubes. Blood samples were centrifuged at 860 g for 15 min at 4 °C to get the serum. Cecum contents and serum were immediately frozen in liquid nitrogen before storing them at -80 °C. An ELISA kit (Shanghai Enzyme-linked Biotechnology Co., Ltd., Shanghai, China) was used to determine the fasting serum insulin levels. Briefly, we conduct the tests of ELISA according to the instruction of the ELISA kit. We added the serum to the microplate. After incubation, we add Biotin-conjugated anti-Mouse INS(Insulin) antibody. It is then combined with HRP-conjugated streptavidin to form an immune complex. The unbound enzyme was then removed by incubation and washing. Next, we added the chromogenic substrate TMB to generate a blue color, which was later changed by the action of acid to a final yellow color. At 450 nm, the absorbance optical density (OD) value was determined.

### 16S *rRNA* sequencing

In order to amplify full-length 16S *rRNA*, universal primers (27 F, AGAGTTTGATCMTGGCTCAG; 1492 R, ACCTTGTTACGACTT) were used for PCR [15]. A pair of 16-nt symmetric barcodes were added to the 5' primer ends. The thermocycling method was: 1 cycle of 95 °C for 5 min, followed by 28 cycles of 95 °C for 1 min, annealing at 58 °C for 1 min, and 72 °C for 2 min, followed by 1 cycle of 72 °C for 10 min. Agilent DNA 1000 Kit and Agilent 2100 Bioanalyser (both from Agilent Technologies) were used for amplifying Amplicon. The Pacific Biosciences Template Prep Kit 2.0 was enrolled in building DNA libraries using amplicons (2 g/sample). The PacBio RS II instrument in CCS mode was used to sequence the P6/C4 chemistry [16]. Raw data was processed using the protocol RS ReadsOfInsert.1 (available through SMRT Portal, version 2.7). We used these restrictive filter criteria: (1) Minimum number of full passes of 5; (2) minimum projected accuracy of 99; (3) minimum insert read length of 1400; (4) maximum insert read length of 1800. The first step was to categorize readings based on primer barcodes specific to each sample. The Quantitative Insights Into Microbial Ecology software eliminated barcode sequences before extracting high-quality sequences (QIIME; version1.7). Then, USEARCH (Version 10.0) was used to align the most abundant sequences within each cluster [17]. Following that, USEARCH assigned the unique sequences to OTUs (cutoff at 97 percent). ChimeraSlayer was used to screen and delete chimeric OTU sequences [18]. To define the remaining OTUs, we used the Ribosomal Database Project (RDP) II and Greengenes (version 13_8) databases (80% bootstrap threshold) [19]. Shannon indexes were used to detect species diversity in each sample community, which is influenced by species richness and community evenness. The PCoA and heatmap were utilized to presume the difference or distance between these samples.

### Metagenomics

The PowerSoil® DNA Isolation Kit (Mo Bio Laboratories) was used to extract total genomic DNA from cecum contents samples according to the manufacturer's instructions. Using a Qubit 3.0 Fluorometer (Life Technologies, Carlsbad, CA, USA) and electrophoresis on a 1% agarose gel, the extracted DNA was assessed for quality and quantity. Vazyme Biotech supplied the VAHTS Universal Plus DNA Library Prep Kit for Illumina (insert size, 350 bp) to prepare the paired-end libraries. The library was sequenced by Illumina NovaSeq 6000 (Biomarker Technologies Co., Ltd., Beijing, China) in paired-end mode using 150-bp reads. We used trimmomatic (version 0.33) to trim sequencing adapters, reads with a quality score < 20 over a sliding window size of 50 bp, and reads with a sequence length < 100 bp. Bioinformatics analyses of the clean sequence data were carried out after trimming adaptors and removing low-quality reads. The reads were aligned using Bowtie2 (version 2.2.4), and any hits associated with their mated reads were removed [20]. MEGAHIT [21] (https://github.com/voutcn/megahit), which uses succinct de Bruijn graphs, was used to assemble metagenomics data. QUAST software version 2.3 was used to calculate assembly summary statistics [22]. Gene predictions and annotation were performed using contigs with a length of or greater than 300 bp. We used MetaGeneMark (version 3.26, default parameters) to predict open reading frames (ORFs) from each assembled contig [23]. With MMseqs2 (https://github.com/soedinglab/mmseqs2, version 12-113e3), all genes predicted with 95% confidence sequence identity (90% coverage) were clustered [24]. In order to align non-redundant gene sequences to the NCBI NR dataset, we used Diamond software and an e-value cutoff of 0.05. Analyses of the Kyoto Encyclopedia of Genes and Genomes database were performed with Diamond (version 0.9.29) and an e-value cutoff of 0.05, using the Kyoto Encyclopedia of Genes and Genomes database. If several alignment results are found (HIT), the best alignment result is chosen as the sequence's annotation.

### LC–MS/MS analysis

In the research of metabolomic investigations, a quantity of 10 mg of cecal matter was subjected to homogenization in a volume of 200 L of water. Cecal matter was processed and analyzed using a high-resolution LC–MS/MS system, mainly comprising a Waters Acquity I-Class PLUS instrument and a Waters Xevo G2-XS QTof mass spectrometer. The raw data collected was processed using Progenesis QI software, with all mass deviations maintained under 100 ppm. Subsequent investigations involved normalization of initial peak area data, repeatability assessment within each sample group, and exploration of identified compounds in KEGG, HMDB, and lipidmaps databases. Statistical analysis was conducted using the T-test and Orthogonal Partial Least Squares Discriminant Analysis (OPLS-DA) modeling with the R language package “ropls” [25]. Screening criteria for differential metabolites were set as FC > 2, *P* < 0.05, and VIP > 1. These parameters align harmoniously with those utilized in analogous research endeavors [26]. The differential metabolites of significant KEGG pathway enrichment were calculated utilizing a hypergeometric distribution test.

### Statistical analysis

Multiple comparisons were conducted by using a one-way ANOVA followed by a Tukey's honest significant difference post hoc analysis. The data are presented as means ± sem. Correlations were analyzed using Spearman’s correlations. Statistics were calculated through the Prism software (7.0). *P* < 0.05 was regarded as statistically significant.

## Results

### AP treatment alleviates the hyperglycemia of HDF KKAy mice

**Fig. 2:**
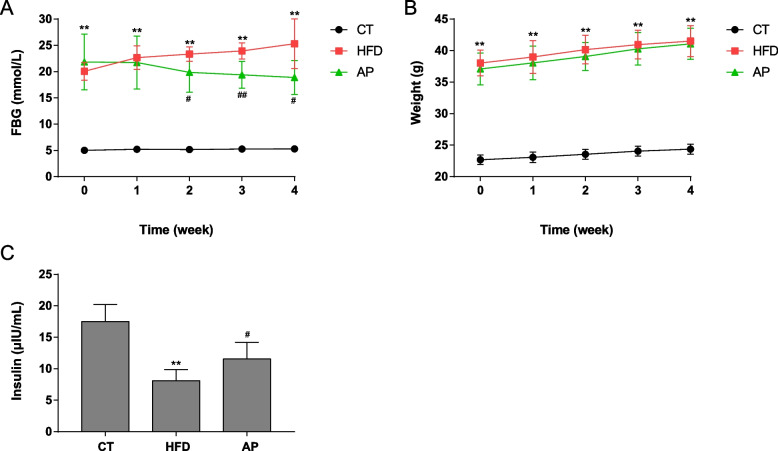
AP supplementation alleviates the hyperglycemia of HDF KKAy mice. Male C57BL/6 mice were treated with a regular diet (CT). KKAy mice were treated with a high-fat diet without (HFD) or with AP supplementation (AP). The experiment lasted for four weeks. **A** The effects of AP supplementation on FBG level. **B** The effects of AP supplementation on body weight level. **C** The effects of AP supplementation on the fasting serum insulin level. Mean ± SEM (*n* = 5). **p < 0.01 compared with CT group; ^#^*p* < 0.05 and ^##^*p* < 0.01 compared with HFD group

To investigate the effect of AP on alleviating hyperglycemia, we investigated the fasting blood glucose (FBG) and body weights of every mice in each group weekly. At the conclusion of the fourth week, the fasting serum insulin levels of the mice were assessed. In the FBG levels between the HFD and control (CT) groups, a significant difference was observed (*p* < 0.01). It is worth noting that after AP supplementation for two weeks, the FBG levels gradually decreased and presented a significant difference compared to the HFD group (Fig. 2A). In the body weight levels, A significant difference was also observed between the HFD and CT groups (*p* < 0.01). However, no significant difference was found between HFD and AP groups after AP supplementation (Fig. 2B). In addition, by the end of the fourth week, the fasting serum insulin levels were significantly downregulated in the HFD group and reversed by AP supplementation (Fig. 2C).

### Full-length 16S *rRNA* gene sequences revealed AP reverses gut dysbiosis in HFD KKAy mice

**Fig. 3:**
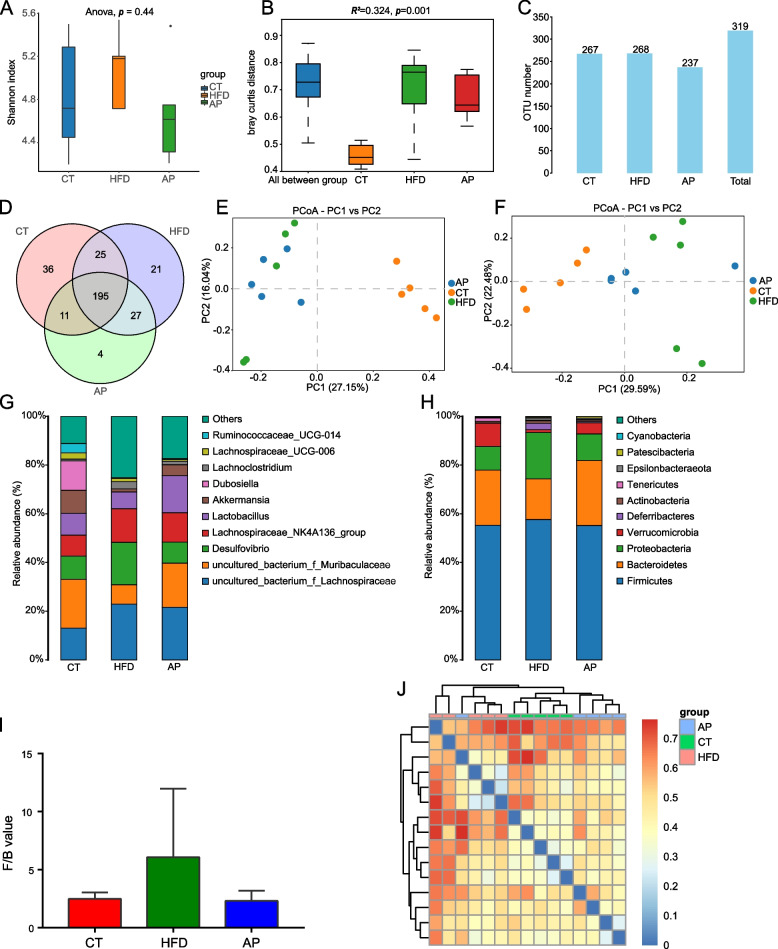
16S full-length *rRNA* sequencing discovered that AP reversed gut dysbiosis in HFD KKAy mice (*n* = 5). **A** Shannon index analysis based on Bray–Curtis distance showed no significant differences were observed among CT, HFD, and AP groups at the OTU level. **B** Bray–Curtis PERMANOVA analysis showed a significant difference between the three groups (*p* = 0.001). **C** The number of OTUs found in the CT, HFD, and AP groups; the total number of OTUs. **D** The Venn diagram showed the extent of overlapped and specific OTUs among the three groups. **E** Bray–Curtis PCoA analysis displayed clear separation among the three groups at the OTU level. **F** Bray–Curtis PCoA analysis displayed clear separation among the three groups at the genus level. **G** The bar graph showed each group's top 10 relative abundance gut microbiota at the genus level. **H** The top 10 relative abundance gut microbiota at the phyla level in each group. Most of the dominant bacteria changed by HFD were reversed by AP treatment. **I** The ratio of Firmicutes/Bacteroidetes was higher in the HFD group than in the CT group and reversed by AP treatment. **J** Heatmap based on Bray–Curtis distance showed that the distance between AP and CT groups was relatively closer than the distance between HDF and CT groups. The color gradient on the adjacent bar chart, transitioning from blue to red, represents the magnitude of differences between the samples, with blue indicating minimal differences and red indicating maximal differences

To explore whether AP can alleviate the gut dysbiosis of HFD KKAy mice, we collected the cecum contents of 5 mice in each group and analyzed them through full-length 16 s *rRNA* gene sequences. The quality control of sequences complied with the standards (Supplementary Fig. 1). No significant differences were observed among CT, HFD, and AP groups in the Shannon index (Fig. 3A). Bray–Curtis PERMANOVA analysis showed a significant difference between the three groups of mice (*p* = 0.001). The intragroup differences were larger in the HFD group than in the CT group. After AP treatment, the intragroup differences were partially reduced (Fig. 3B). Based on 97% sequence similarity, the OTU number of each group was obtained. The number of OTU was less in the AP group than in the HFD group (Fig. 3C). To catch the specific OTU, which might affect the benefits of AP, we constructed a Venn diagram to determine the extent of OTU overlap between groups (Fig. 3D). Venn diagram analysis showed that 195 OTU were shared among the 3 groups, and 36, 21, and 4 unique OTU were identified in CT, HFD, and AP groups, respectively. Compared with the CT group, the HFD group has 48 additional OTU, 36 of them belong to Firmicutes, 5 of them belong to Bacteroidetes, and 4 of them belong to Proteobacteria. 47 OTU in the CT group were not found in the HFD group, 28 of them belong to Firmicutes, and 5 of them belong to Bacteroidetes. In addition, compared with the HFD group, the AP group has 15 additional OTU, 8 of them belong to Firmicutes, and 4 of them belong to Bacteroidetes. 46 OTU in the HFD group were not found in the AP group, 38 of them belong to Firmicutes (Supplementary Table 1). Taken as a whole, the result of the Venn diagram analysis suggested that HFD increased the number of Firmicutes and Proteobacteria phyla and reduced the number of Bacteroidetes phylum, whereas AP partially reverses these changes. Next, we sought to explore the abundance changes at the OTU and genus levels. Bray–Curtis Principal Coordinates Analysis (PCoA) analysis displayed clear separation among the three groups at the genus level, suggesting the differences may mainly occur at the genus level, not the OTU level (Fig. 3E and F). We showed the top 10 relative abundance gut microbiota at the genus and phyla level in each group through a bar graph. Interestingly, we found that most of the dominant bacteria changed by HFD and reversed by AP treatment, such as *Akkermansia*, *Lactobacillus*, *Lachnospiraceae*, *Desulfovibrio*, at genus level, and Lachnoclostridium, Muribaculaceae in family level, and Firmicutes, Bacteroidetes, Proteobacteria, Verrucomicrobia, Deferribacteres in phyla level (Fig. 3G and H). The ratio of Firmicutes/Bacteroidetes was higher in the HFD group than in the CT group and reversed by AP (Fig. 3I). A heatmap based on Bray–Curtis distance was used to perform pairwise distance between samples and hierarchical clustering, respectively. The length of the lines connecting the samples in the heatmap represents the degree of difference between the samples. We found that the distance between AP and CT groups was relatively closer than the distance between HDF and CT groups, indicating that the abundance of gut microbiota of the AP treated KKAy mice was more similar to that of the normal mice in the CT group than the KKAy mice in the HFD group (Fig. 3J). These data suggested that AP may play a crucial role in alleviating gut dysbiosis caused by HFD. This phenomenon may be relevant to the curative effect of AP against T2D.

### Metagenomics sequencing explored the changes of gut microbiota and function by AP treatment in HFD KKAy mice

**Fig. 4:**
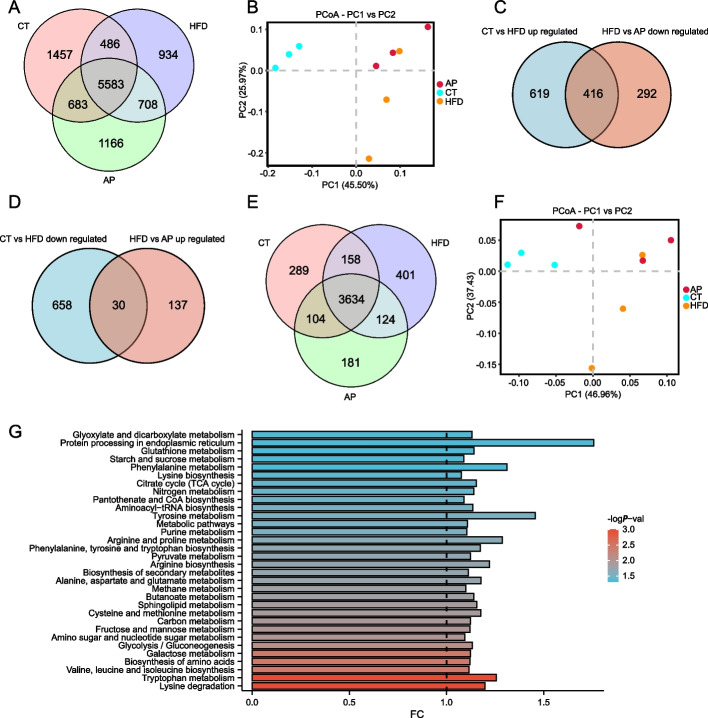
Metagenomics sequencing discovered that AP reversed gut dysbiosis in HFD KKAy mice (*n* = 3). **A** Venn diagram showed the extent of overlapped and specific bacterial species among the three groups. **B** Bray–Curtis PCoA analysis displayed clear separation among the three groups at the species level. **C** Based on the criteria of *p* < 0.05, 416 species abundance significantly increased by HFD and reversed by AP. **D** Based on the criteria of *p* < 0.05, 30 species significantly decreased by HFD and reversed by AP. **E** Venn diagram showed the extent of overlapped and specific KO among the three groups. **F** Bray–Curtis PCoA analysis displayed clear separation among the three groups at the KO level. **G** The different KEGG pathways related to metabolism between HFD and AP groups

Changes in gut microbiota are often associated with altered function. To further detect the changes of bacteria species and relative potential function by AP treatment, metagenomics sequencing was used with the cecum contents of 3 randomly selected mice in each group. The quality control of metagenomic sequencing complied with the standards (Supplementary Fig. 2). 8209 bacterial species were annotated in the CT group, while only 7711 species were annotated in the HFD group. AP treatment increased the species number to 8140. Venn diagram showed that 5583 species were shared among three groups, and 1457, 934, and 1166 unique bacteria species were identified in CT, HFD, and AP groups (Fig. 4A). The detailed bacteria species distribution in each group was displayed in Supplementary Table 2. Bray–Curtis PCoA analysis displayed clear separation among the three groups at the species level (Fig. 4B). Based on the criteria of *p* < 0.05, 416 species' abundance significantly increased by HFD and reversed by AP, which major belong to Proteobacteria, Firmicutes, and Actinobacteria phyla (Fig. 4C, Supplementary Fig. 3A). Meanwhile, 30 bacterial species' abundance significantly decreased by HFD and reversed by AP, which major belong to Firmicutes, Proteobacteria, and Bacteroidetes (Fig. 4D, Supplementary Fig. 3B). These results further provided more evidence that AP may alleviate gut dysbiosis caused by HFD through metagenomics sequencing. Then, we applied the KEGG orthologues (KO) to assess the potential microbial functional roles in the gut microbiota of each treatment. Venn diagram showed the extent of overlapped and specific KO between groups (Fig. 4E). Bray–Curtis PCoA analysis also displayed clear separation among the three groups at the KO level (Fig. 4F). Then we screened the significantly altered pathway between HFD and AP groups to explore the potential effective function of AP treatment. Based on the criteria of *p* < 0.05, we found that 49 KEGG pathways were significantly differentially expressed between HFD and AP groups (Supplementary Table 3). We selected and showed the metabolism-related pathway, which had the highest proportion (Fig. 4G). These results suggested that AP treatment was effective in alleviating HFD-induced gut dysbiosis, and changes in bacterial taxa also affect metabolic functions of the gut microbiota, which might be associated with the alleviating of diabetes.

### LC–MS/MS-based untargeted metabolic profiling explored the metabonomic changes by AP treatment in HFD KKAy mice

**Fig. 5:**
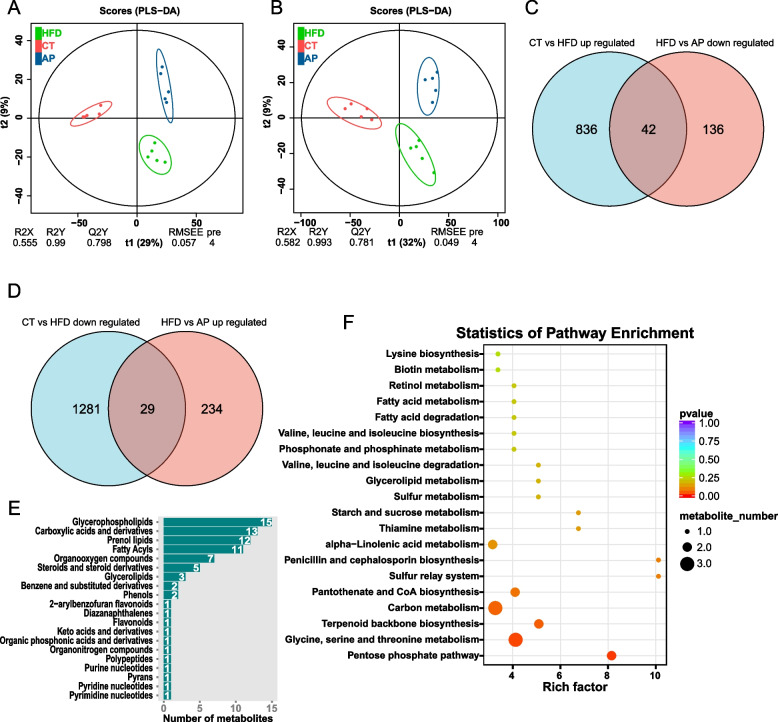
LC–MS/MS-based untargeted metabolic profiling in positive and negative modes discovered that AP changes gut metabolome in HFD KKAy mice (*n* = 5). **A**, **B** The PLS-DA analysis showed clear separation among three groups in the positive model (R2X = 0.555, R2Y = 0.99, Q2Y = 0.798) and in the negative model (R2X = 0.582, R2Y = 0.993, Q2Y = 0.781). **C**, **D** With the criteria of either VIP > 1 and *p* < 0.05, the Venn diagram showed the number of differential metabolites between groups. 42 metabolites were up-regulated by HFD and reversed by AP, 29 metabolites were down-regulated by HFD and reversed by AP. **E** The differential metabolites class categories between HFD and AP groups. **F** The top 20 of KEGG enrichment analysis based on the differential metabolites between HFD and AP groups

To clarify the metabolic feature of AP treatment, we performed the LC–MS/MS-based untargeted metabolic profiling in the positive and negative models on cecum contents. The PLS-DA analysis showed clear separation among three groups in a positive model (R2X = 0.555, R2Y = 0.99, Q2Y = 0.798, Fig. 5A) and in a negative model (R2X = 0.582, R2Y = 0.993, Q2Y = 0.781, Fig. 5B). With the criteria of either VIP > 1 (multivariate statistical analysis) and *p* < 0.05 (univariate statistics), we screened the differential metabolites between groups. AP can partially reverse the differential metabolites in the HFD group, suggesting AP treatment may improve the abnormalities in metabolites caused by HFD (Fig. 5C and D,Supplementary Table 4). To further explore the metabolic alteration induced by AP, the differential metabolites between HFD and AP groups were classified and performed enrichment analysis. The metabolite class categories showed that the differential metabolites were mainly classified in Glycerophospholipids, Carboxylic acids and derivatives, Prenol lipids, Fatty Acyls, Organooxygen compounds, Steroids, Steroid derivatives, and so on (Fig. 5E). The enrichment analysis showed that the differential metabolites were mainly enriched in 50 pathways (Supplementary Table 5), mainly containing Pentose phosphate pathway, Glycine, Serine and threonine metabolism, Terpenoid backbone biosynthesis, Carbon metabolism, Pantothenate and CoA biosynthesis, and so on (Fig. 5F). These results suggested that AP treatment may help to ameliorate dysregulated gut metabolic status, possibly as a result of its effect on gut microbiota composition regulation.

### Correlation between gut microbial species and differential metabolites

**Fig. 6:**
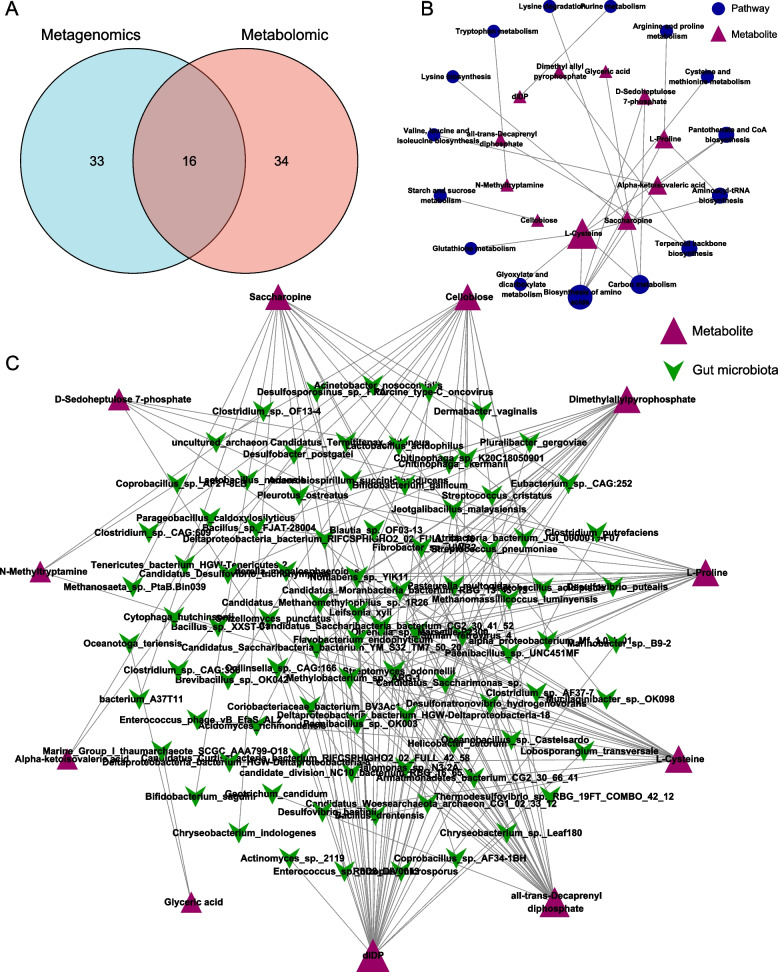
Exploration of the relationship between gut microbial species and differential metabolites. **A** Between the HFD and AP groups, there were 16 common metabolic pathways that overlapped between the metagenomic sequencing and metabolomics. **B** The network analysis showed that the 16 common metabolic pathways contained 11 differential metabolites. **C** The network analysis showed the significant relationship between the metabolites and the bacterial species based on the criteria of the spearman’s coefficient less than -0.7 or more than 0.7, and *p* < 0.01

To further explore the relationship between metabolites and gut microbiota in AP treatment, we performed a joint analysis of metagenomics sequencing and metabolomics. Between the HFD and AP groups, we found 16 common metabolic pathways in metagenomics sequencing and metabolomics (Fig. 6A). The network analysis between the metabolic pathway and differential metabolites showed that the 16 common metabolic pathways contained 11 differential metabolites (Fig. 6B). Based on the criteria of the spearman’s coefficient of less than -0.7 or more than 0.7 and *p* < 0.01, we also constructed a network to present the significant relationship between the metabolites and the bacterial species and found that each metabolite was significantly related to multiple bacterial species (Fig. 6C). These findings suggested that AP changed certain bacteria, which were correlated with some key metabolites in diabetes, indicating the crucial roles of these bacteria in AP treatment.

## Discussion

Accumulating studies have revealed a correlation between diabetes and alteration in gut microbiota composition and diversity, implying that gut microbiota may play a pivotal role in developing an anti‐diabetes therapy strategy. This strategy has been effectively employed in both animal models and human subjects, demonstrating significant improvements in symptoms and disease progression [3]. In addition, many studies indicate that polysaccharides have beneficial effects on gut microbiota and metabolism. Sui, Yi et al. (2019) discovered that a branched arabinoglucan, a type of polysaccharide derived from *Angelica sinensis*, effectively reversed renal dysfunction and inflammation in diabetic rats, suggesting that AP could be a promising treatment for diabetic nephropathy [27]. Using 72 mice, Wang, Kaiping et al. (2015) found that AP has potential hypoglycemic and hypolipidemic effects, reducing blood glucose and lipid levels and improving insulin resistance in prediabetic and streptozotocin-induced diabetic mice, suggesting its potential in diabetes prevention and treatment [28]. However, it remains unclear whether AP can influence the development and progression of T2D through modulating gut microbiota. Therefore, it is worth to further exploring the effect of polysaccharides on diabetic metabolism through gut microbiota. This present study was designed to investigate whether AP has a potentially protective effect on diabetes through gut microbiota and gut metabolites. We performed 16S full-length *rRNA* sequencing, metagenomic sequencing, and liquid chromatography-tandem mass spectrometry (LC–MS/MS), respectively, to discover the mechanism of AP on host health. Our results indicated that AP possesses a beneficial effect on T2D. The beneficial effect may associate with the changes in gut microbial composition, function, and gut metabolites.

Polysaccharides can be used by gut microbiota as a carbon source because they are partially or completely fermented in the colon [29]. We found that AP treatment effectively alleviated glycemic disorders with the alteration of gut microbiota composition and function. A series of empirical studies have also discovered that various polysaccharides can modulate gut microbiota, enhancing beneficial microbes while reducing harmful ones, thereby mitigating symptoms of obesity, T2D, and metabolic syndrome [30–32]. Through type 2 diabetic rat models, Yao et al. (2020) found that by promoting the production of SCFAs and upregulating SCFA-GLP1/PYY associated sensory mediators, *Cyclocarya paliurus* polysaccharide may be able to alleviate T2D symptoms [33]. Likewise, Chen et al. (2019) reported that the polysaccharides from adlay seed also possessed anti-diabetic functions by regulation of the gut microbiota and its metabolic pathways in the T2D mouse model [34]. These findings are consistent with our results.

The composition of gut microbiota has been proved to be closely associated with the host's health. Various taxa of the gut microbiota have been substantiated to maintain a significant association with type 2 diabetes [35]. Based on the full-length 16S *rRNA* gene sequences, we found apparent differences in bacterial composition between the groups. The most obvious finding to emerge from the analysis is that the ratio of Firmicutes/Bacteroidetes phyla was increased in the HFD group, while AP effectively reversed these alters. The Firmicutes/Bacteroidetes ratio is widely considered to play a significant role in maintaining appropriate intestinal homeostasis [36]. This ratio is closely associated with many metabolic-related diseases, such as T2D and non-alcoholic fatty liver disease [37, 38]. Previous clinical studies show that the ratio of Firmicutes/Bacteroidetes was higher in T2D patients than in healthy subjects [39, 40]. Pascale et al. (2019) have noted that a rise in the Firmicutes/Bacteroidetes ratio is associated with high-grade inflammation and greater ability to harvest energy from food [41]. The lipopolysaccharide and deoxycholic acid produced by Firmicutes will enters the liver through the portal vein, leading inflammation in the liver and glucose metabolism disorders [42]. Bacteroidetes are mostly located in the distal colon and ferment indigestible polysaccharides to produce SCFAs [43]. SCFAs work as an energy source, activates G protein-coupled receptors (GPCRs), and inhibit histone deacetylases, regulating a variety of physiological processes and potentially contributing to health [42]. Another finding of our research is that AP reduced the abundance of Proteobacteria. As mentioned in the literature review, Proteobacteria is being identified as a probable disease microbial signature, and some research indicated that Proteobacteria involve in metabolic disorders related disease [44]. Shin et al. (2015) pointed out that members of the phylum Proteobacteria are present at low levels in the intestines of healthy individuals. There is a correlation between the proliferation of Proteobacteria and disease states. It has been suggested that the load of Proteobacteria could serve as a potential diagnostic criterion for ecological imbalance and diseases [45]. A previous report proposed that Proteobacteria increase can be recognized as a potential diagnostic signature of dysbiosis linked to diabetes [46]. Likewise, Su et al. (2022) found that taxifolin improved obesity and gut microbiota regulation in HFD-fed C57BL/6 J mice. Taxifolin reduced body weight gain, inhibited fat accumulation, improved liver health, enhanced SOD activity, and improved insulin resistance. They discovered that in HFD-fed C57BL/6 J mice, there was a reduction in gut microbiota diversity, while the Firmicutes/Bacteroidetes ratio increased. However, taxifolin improved the diversity and decreased the Firmicutes/Bacteroidetes ratio. Importantly, taxifolin suppressed the proliferation of Proteobacteria [47]. We also found AP decreased the abundance of Deferribacteres. Deferribacteres gain energy through obligate or facultative anaerobic metabolism. Through clinical trials, Nuli et al. (2019) found that compared to healthy people, Deferribacteres were significantly increased in the patients with T2D [48]. Through the diabetic mouse model, Li et al. (2019) found that the abundance of Deferribacteres was closely related to IL-6, TNF-α, and IL-17A, respectively, suggesting the decreasing of Deferribacteres by AP may alleviate T2D via anti-inflammation [49]. Our result showed that the abundance of Verrucomicrobia was decreased in the HFD group and reversed by AP treatment. Likewise, Zhang, Qi, and Nan Hu. reported that in patients with T2D, phyla Verrucomicrobia was significantly increased after metformin treatment [50]. These findings are contrary to previous studies reported by Zhao et al. (2020), which have suggested that the abundance of Verrucomicrobia was increased in patients with T2D [51]. However, Ahmad et al. (2019) reported that in the Pakistani population, Verrucomicrobia was decreased in patients with T2D [52]. These contradicted results may be due to size, quality, and race differences. Differences were also observed at the genus level. We found that at genus level, the abundance of *Akkermansia* and *Lactobacillus* were increased by AP, whereas the abundance of *Desulffovibrio* was decreased. The most prevalent species under the *Akkermansia* genus is *Akkermansia muciniphila*. *Askkermansia muciniphila* is a Gram-negative anaerobic bacterium that constitutes 3–5% of the gut microbial community in healthy adults and degrades mucin in the gut. The lack of *Akkermansia muciniphila* may cause many diseases [53]. Research indicates an inverse relationship between *Akkermansia muciniphila* and conditions such as obesity, diabetes, inflammation, and metabolic disorders, and due to its beneficial probiotic properties against obesity and diabetes, enhancing the abundance of *Akkermansia muciniphila* in the gut through dietary interventions has attracted significant interest as a potential strategy for treating metabolic diseases [54]. Recently, Zhang et al. (2021) reported that in lean type 2 diabetes, a decreased abundance of *Akkermansia muciniphila* impairs insulin secretion and glucose homeostasis, suggesting that improving *Akkermansia muciniphila* may be a new strategy for treating diabetes [55]. *Lactobacillus* is a genus of bacteria that are considered beneficial or protective for health. Rodrigues, Richard R et al. (2021) used a data-driven approach to identify specific gut microbiota that influence metabolic changes under a western diet, and finds that *Lactobacillus* genus can improve lipid metabolism through effects on liver mitochondria, suggesting potential probiotic interventions for T2D [56]. *Desulfovibrio* genus has been considered harmful health. The *Desulfovibrio* genus reduces dietary sulfites and sulfates, as well as sulfated mucopolysaccharides in mucin, producing hydrogen sulfide, a harmful chemical [57]. Viewed as a whole, we found that the full-length 16S *rRNA* gene sequence showed AP improved gut microbiota structure.

The gut microbiota significantly impacts the host’s health by regulating metabolic functions. Through metagenomics sequencing, we found that many gut microbiotas were significantly changed by HFD and reversed by AP at the species level, further suggesting the benefit of AP against T2D through regulating gut microbiota. In addition, this study show that many metabolic function pathways were changed by AP in the HFD KKAy mice, containing Tryptophan metabolism, Purine metabolism, Pantothenate and CoA biosynthesis, Starch and sucrose metabolism, and Glutathione metabolism. Tryptophan metabolism is crucial in both physiology and physiopathology. Disorder of tryptophan metabolism has been demonstrated that strongly related to diabetes and related complications [58]. Qi et al. (2022) reported that Tryptophan metabolism was associated with T2D risk through a cohort study [59]. Moreover, a cross-sectional study showed that Pantothenate and CoA biosynthesis significantly changed in diabetic kidney disease [60]. Leonardi et al. (2014) have pointed out a possible connection between CoA and insulin homeostasis [61]. The starch and sucrose metabolisms are both part of carbohydrate metabolism, which provides the cells with energy [62]. Improving Starch and sucrose metabolism may be beneficial against T2D. Glutathione is mainly converted from glutamate during glutathione metabolism. Glutathione plays an essential role in antioxidant defense. Many studies revealed that the increased oxidative stress in diabetes pathogenesis was associated with impaired Glutathione metabolism [63]. The purine metabolic pathway is involved in a number of inflammatory processes, and its end product, uric acid, is linked to a number of metabolic disorders such as insulin resistance and obesity [64, 65]. In conclusion, these metagenomic sequencing results help us to find the functional pathways involved in AP treatment of T2D.

Gut metabolites, produced jointly by the host and the gut microbiota, are critical for supporting host health [66]. We analyzed the metabolite profile and metabolic pathway in cecum contents to see which metabolites were involved in the positive effect of AP. In this study we found that AP changed many metabolites and partially reversed the metabolites changed by HFD. Many metabolites are classified as Glycerophospholipids. Precious studies have revealed the association between T2D and Glycerophospholipids. Fu et al. (2020) indicated that abnormalities in the glycerophospholipids metabolism are one of the features of diabetes [67]. The adjustment of AP on glycerophospholipids may be beneficial to the treatment of T2D. What is surprising is that 16 pathways enriched by differential metabolites were the same as those enriched by metagenomics, enhancing the reliability of these results. These results also indicated that the metabolites in the 16 pathways might be closely related to the gut microbiota. The 16 pathways contained 11 differential metabolites. As shown in Fig. 6B, L-Cysteine was involved in the most metabolites pathways, suggesting L-Cysteine may be an essential metabolite in the treatment effect by AP. L-Cysteine is a crucial metabolite involved in glutathione metabolism. Diabetic patients have lower blood levels of L-Cysteine. Based on an in vitro experiment, Achari and Jain (2017) suggested that L-Cysteine supplementation can increase insulin sensitivity and can be used as adjuvant therapy for diabetes [68]. Another prior study also reported that in T2D patients, L-Cysteine could improve glucose metabolism. Likewise, in animal studies, supplementing with L-Cysteine leads to a significant improvement in glucose metabolism [69]. In addition, this study shows the relationship between the 11 metabolites and gut microbiota. The results showed that many bacterial species were correlated with these 11 different metabolites, suggesting these bacterial species might participate in the effect of AP against T2D.

## Conclusions

Altogether, in this study, we explored the therapy effect of AP against T2D through HFD KKAy mouse model. The results showed that AP effectively reversed HFD-induced alterations in gut microbial composition, function, and gut metabolites. These data suggest the potential of AP as a candidate for T2D management. Moreover, the observed anti-diabetic properties of AP may be partially associated with its influence on gut microbiota. These improved gut microbes and metabolites by AP will provide a basis for further research.

### Supplementary Information

**Additional file 1: Supplementary Figure 1. **The quality control of full-length 16S rRNA gene sequences. (A) Multiple samples' rarefaction curves. (B) Multiple samples' Shannon curves. (C) Multiple samples' rank abundance curve. (D) Multiple samples' species cumulation curves. 

**Additional file 2: Supplementary Figure 2. **The quality control of metagenomics sequencing. (A) Multiple samples' rarefaction curves. (B) Multiple samples' Shannon curves. (C) Multiple samples' rank abundance curve. (D) Multiple samples' species cumulation curves. 

**Additional file 3: Supplementary Figure 3. **The category of the bacteria species changed by HFD and reversed by AP at the phyla level. (A) The proportion of bacteria species increased by HFD and was reversed by AP at the phyla level. (B) The proportion of bacteria species decreased by HFD and was reversed by AP at the phyla level.

**Additional file 4: Supplementary Table 1. **The detail OTU distribution in CT, HFD, and AP groups.

**Additional file 5: Supplementary Table 2. **The detail bacteria species distribution in CT, HFD, and AP groups.

**Additional file 6: Supplementary Table 3. **The significant altered KEGG pathway by AP treatment.

**Additional file 7: Supplementary Table 4. **The differential metabolites between.

**Additional file 8: Supplementary Table 5. **The enrichment analysis of differential metabolites between HDF and ASP groups.

## Data Availability

The 16S *rRNA* sequencing and metagenomics datasets generated during the current study are available in the NCBI sequence reads archive (SRA) [https://www.ncbi.nlm.nih.gov/bioproject/PRJNA951203]. The metabolomics datasets used to support the findings of this study are included within the article and the supplementary information files.

## Abbreviations

AP: Angelica polysaccharides
T2D: Type 2 diabetes
SCFAs: Short-chain fatty acids
HFD: High-fructose diet
KO: KEGG orthologues
LC–MS/MS: Liquid chromatography-tandem mass spectrometry
GPCRs: G protein-coupled receptors
CT: Control
OD: Optical density
QIIME: Quantitative Insights Into Microbial Ecology
RDP: Ribosomal Database Project
PCoA: Principal Coordinates Analysis
ORFs: Open reading frames
FBG: Fasting blood glucose
TMB: 3,3',5,5'-Tetramethylbenzidine
