# Supplementary material for: Angelica polysaccharides relieve blood glucose levels in diabetic KKAy mice possibly by modulating gut microbiota: an integrated gut microbiota and metabolism analysis
Source: BMC Microbiol. 2023 Oct 3;23:281. doi: 10.1186/s12866-023-03029-y (PMC10546737; doi:10.1186/s12866-023-03029-y)
Supplement: Supplementary file 1 — Additional file 1: Supplementary Figure 1. The quality control of full-length 16S rRNA gene sequences. (A) Multiple samples' rarefaction curves. (B) Multiple samples' Shannon curves. (C) Multiple samples' rank abundance curve. (D) Multiple samples' species cumulation curves. [file 12866_2023_3029_MOESM1_ESM.docx]

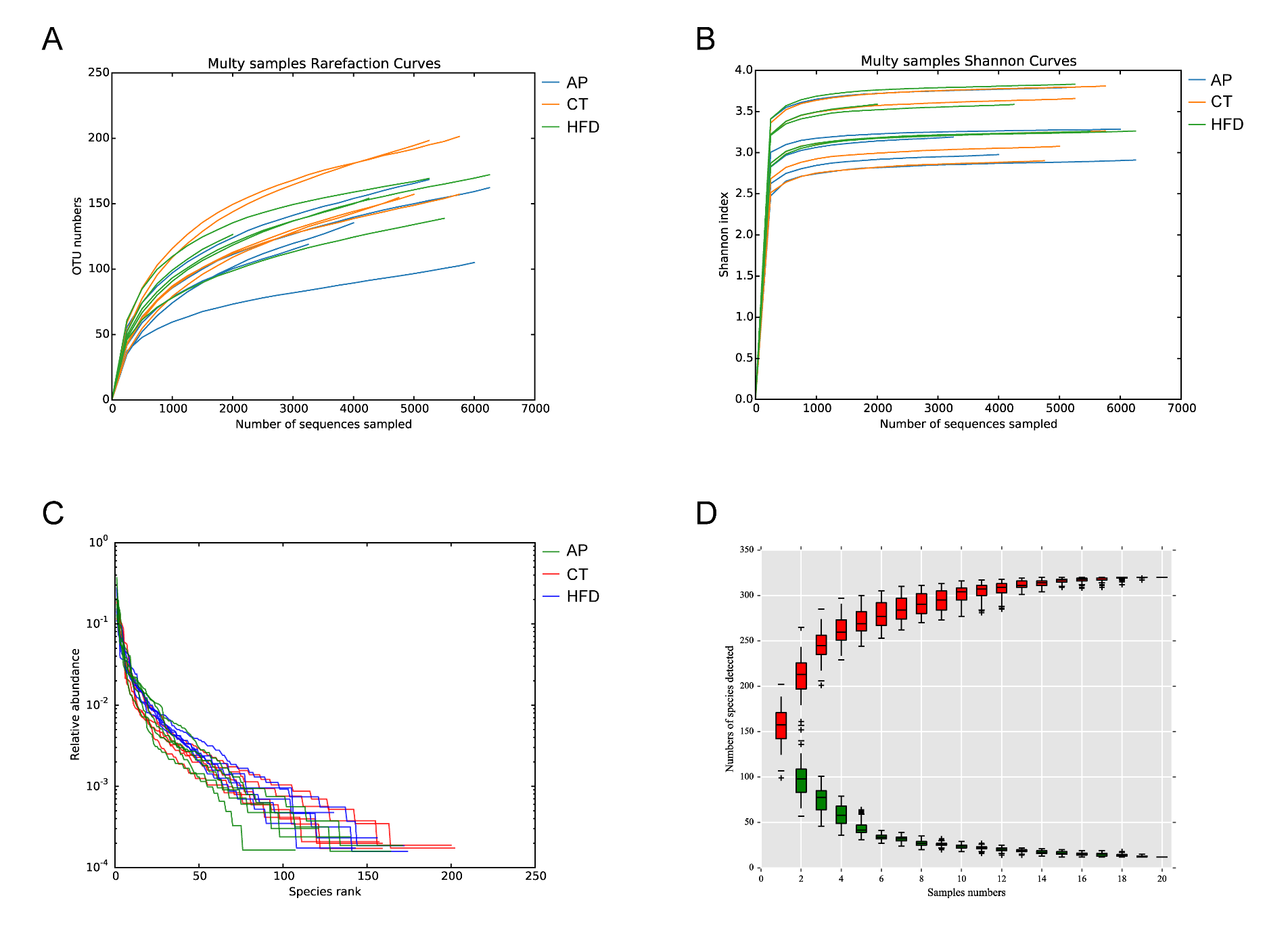


Supplementary figure 1: The quality control of full-length 16S rRNA gene sequences. (A) Multiple samples' rarefaction curves. (B) Multiple samples' Shannon curves. (C) Multiple samples' rank abundance curve. (D) Multiple samples' species cumulation curves.
