# Supplementary material for: Angelica polysaccharides relieve blood glucose levels in diabetic KKAy mice possibly by modulating gut microbiota: an integrated gut microbiota and metabolism analysis
Source: BMC Microbiol. 2023 Oct 3;23:281. doi: 10.1186/s12866-023-03029-y (PMC10546737; doi:10.1186/s12866-023-03029-y)
Supplement: Supplementary file 2 — Additional file 2: Supplementary Figure 2. The quality control of metagenomics sequencing. (A) Multiple samples' rarefaction curves. (B) Multiple samples' Shannon curves. (C) Multiple samples' rank abundance curve. (D) Multiple samples' species cumulation curves. [file 12866_2023_3029_MOESM2_ESM.docx]

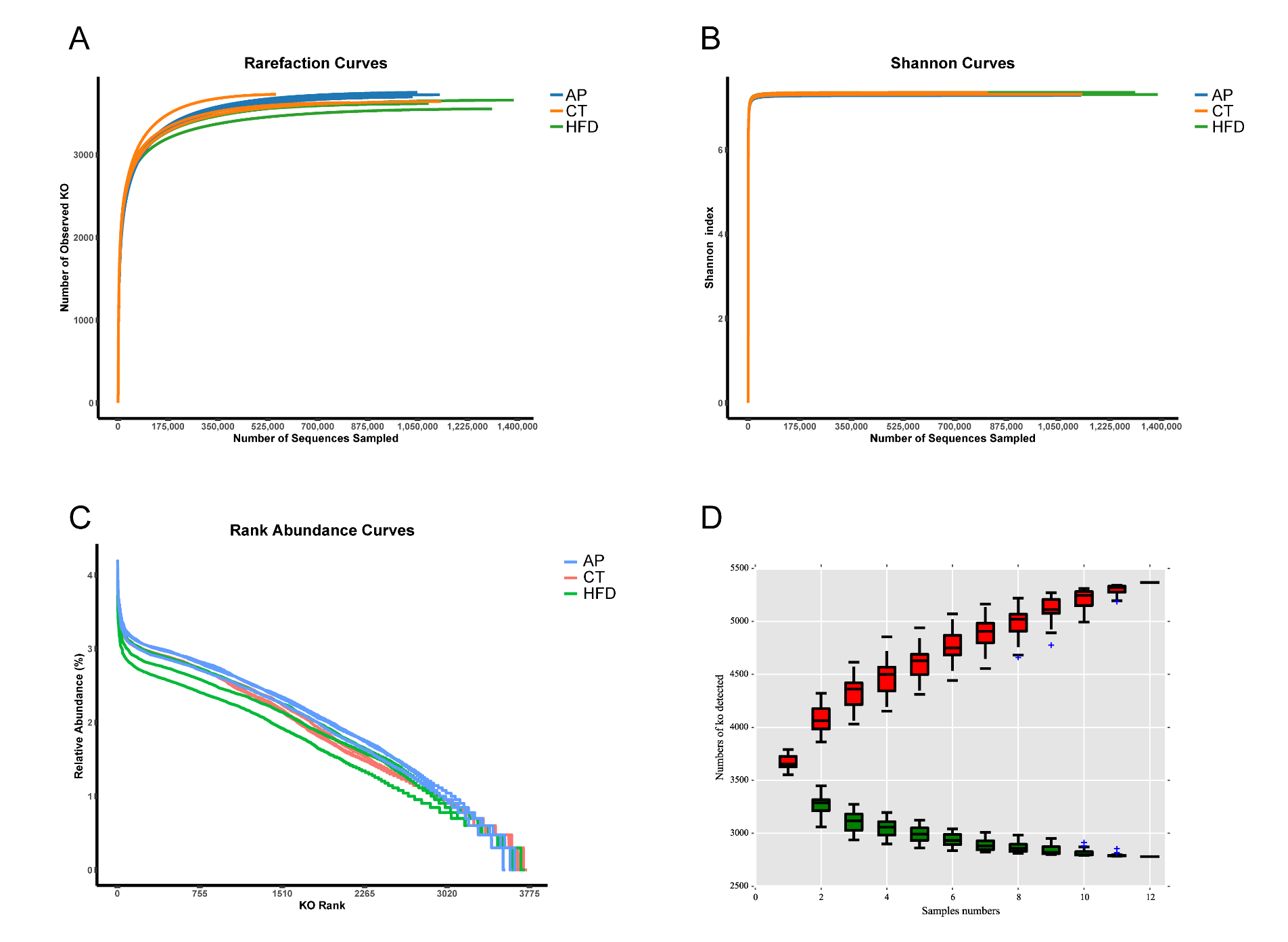


Supplementary figure 2: The quality control of metagenomics sequencing. (A) Multiple samples' rarefaction curves. (B) Multiple samples' Shannon curves. (C) Multiple samples' rank abundance curve. (D) Multiple samples' species cumulation curves.
