# Supplementary material for: Angelica polysaccharides relieve blood glucose levels in diabetic KKAy mice possibly by modulating gut microbiota: an integrated gut microbiota and metabolism analysis
Source: BMC Microbiol. 2023 Oct 3;23:281. doi: 10.1186/s12866-023-03029-y (PMC10546737; doi:10.1186/s12866-023-03029-y)
Supplement: Supplementary file 3 — Additional file 3: Supplementary Figure 3. The category of the bacteria species changed by HFD and reversed by AP at the phyla level. (A) The proportion of bacteria species increased by HFD and was reversed by AP at the phyla level. (B) The proportion of bacteria species decreased by HFD and was reversed by AP at the phyla level. [file 12866_2023_3029_MOESM3_ESM.docx]

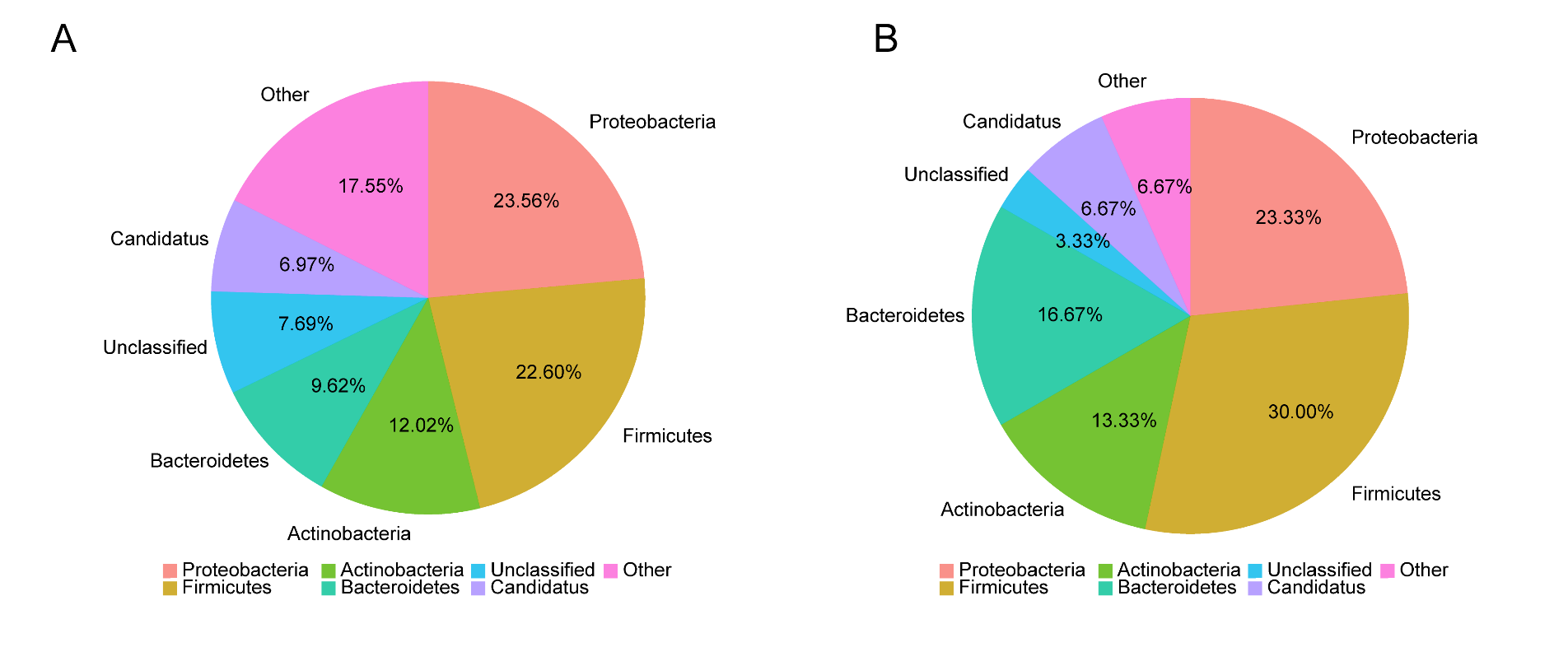


Supplementary figure 3. The category of the bacteria species changed by HFD and reversed by AP at the phyla level. (A) The proportion of bacteria species increased by HFD and was reversed by AP at the phyla level. (B) The proportion of bacteria species decreased by HFD and was reversed by AP at the phyla level.
