# Supplementary material for: Angelica polysaccharides relieve blood glucose levels in diabetic KKAy mice possibly by modulating gut microbiota: an integrated gut microbiota and metabolism analysis
Source: BMC Microbiol. 2023 Oct 3;23:281. doi: 10.1186/s12866-023-03029-y (PMC10546737; doi:10.1186/s12866-023-03029-y)
Supplement: Supplementary file 4 — Additional file 4: Supplementary Table 1. The detail OTU distribution in CT, HFD, and AP groups. [file 12866_2023_3029_MOESM4_ESM.docx]

Supplementary Table 1. The detail OTU distribution in CT, HFD, and AP groups.

| OTU_id | CT | HFD | AP | taxonomy |
| --- | --- | --- | --- | --- |
| OTU9 | + | + | + | k__Bacteria; p__Firmicutes; c__Bacilli; o__Lactobacillales; f__Lactobacillaceae; g__Lactobacillus; s__Lactobacillus_murinus |
| OTU28 | + | + | + | k__Bacteria; p__Firmicutes; c__Clostridia; o__Clostridiales; f__Lachnospiraceae; g__uncultured_bacterium_f_Lachnospiraceae; s__uncultured_bacterium_f_Lachnospiraceae |
| OTU2 | + | + | + | k__Bacteria; p__Proteobacteria; c__Deltaproteobacteria; o__Desulfovibrionales; f__Desulfovibrionaceae; g__Desulfovibrio; s__uncultured_bacterium_g_Desulfovibrio |
| OTU7 | + | + | + | k__Bacteria; p__Firmicutes; c__Clostridia; o__Clostridiales; f__Lachnospiraceae; g__uncultured_bacterium_f_Lachnospiraceae; s__uncultured_bacterium_f_Lachnospiraceae |
| OTU8 | + | + | + | k__Bacteria; p__Firmicutes; c__Clostridia; o__Clostridiales; f__Lachnospiraceae; g__Lachnospiraceae_NK4A136_group; s__uncultured_bacterium_g_Lachnospiraceae_NK4A136_group |
| OTU1 | + | + | + | k__Bacteria; p__Verrucomicrobia; c__Verrucomicrobiae; o__Verrucomicrobiales; f__Akkermansiaceae; g__Akkermansia; s__Akkermansia_muciniphila |
| OTU25 | + | + | + | k__Bacteria; p__Bacteroidetes; c__Bacteroidia; o__Bacteroidales; f__Muribaculaceae; g__uncultured_bacterium_f_Muribaculaceae; s__uncultured_bacterium_f_Muribaculaceae |
| OTU54 | + | + | + | k__Bacteria; p__Firmicutes; c__Clostridia; o__Clostridiales; f__Lachnospiraceae; g__Lachnospiraceae_NK4A136_group; s__uncultured_bacterium_g_Lachnospiraceae_NK4A136_group |
| OTU4 | + | + | + | k__Bacteria; p__Bacteroidetes; c__Bacteroidia; o__Bacteroidales; f__Muribaculaceae; g__uncultured_bacterium_f_Muribaculaceae; s__uncultured_bacterium_f_Muribaculaceae |
| OTU13 | + | + | + | k__Bacteria; p__Firmicutes; c__Bacilli; o__Lactobacillales; f__Lactobacillaceae; g__Lactobacillus; s__Lactobacillus_reuteri |
| OTU60 | + | + | + | k__Bacteria; p__Firmicutes; c__Clostridia; o__Clostridiales; f__Lachnospiraceae; g__uncultured_bacterium_f_Lachnospiraceae; s__uncultured_bacterium_f_Lachnospiraceae |
| OTU33 | + | + | + | k__Bacteria; p__Bacteroidetes; c__Bacteroidia; o__Bacteroidales; f__Muribaculaceae; g__uncultured_bacterium_f_Muribaculaceae; s__uncultured_bacterium_f_Muribaculaceae |
| OTU15 | + | + | + | k__Bacteria; p__Bacteroidetes; c__Bacteroidia; o__Bacteroidales; f__Rikenellaceae; g__Alistipes; s__uncultured_bacterium_g_Alistipes |
| OTU96 | + | + | + | k__Bacteria; p__Firmicutes; c__Clostridia; o__Clostridiales; f__Lachnospiraceae; g__uncultured_bacterium_f_Lachnospiraceae; s__uncultured_bacterium_f_Lachnospiraceae |
| OTU18 | - | + | + | k__Bacteria; p__Proteobacteria; c__Deltaproteobacteria; o__Desulfovibrionales; f__Desulfovibrionaceae; g__uncultured_bacterium_f_Desulfovibrionaceae; s__uncultured_bacterium_f_Desulfovibrionaceae |
| OTU11 | - | + | + | k__Bacteria; p__Bacteroidetes; c__Bacteroidia; o__Bacteroidales; f__Marinifilaceae; g__Odoribacter; s__uncultured_bacterium_g_Odoribacter |
| OTU17 | + | + | + | k__Bacteria; p__Bacteroidetes; c__Bacteroidia; o__Bacteroidales; f__Muribaculaceae; g__uncultured_bacterium_f_Muribaculaceae; s__uncultured_bacterium_f_Muribaculaceae |
| OTU65 | + | + | + | k__Bacteria; p__Firmicutes; c__Clostridia; o__Clostridiales; f__Lachnospiraceae; g__uncultured_bacterium_f_Lachnospiraceae; s__uncultured_bacterium_f_Lachnospiraceae |
| OTU69 | + | + | + | k__Bacteria; p__Bacteroidetes; c__Bacteroidia; o__Bacteroidales; f__Muribaculaceae; g__uncultured_bacterium_f_Muribaculaceae; s__uncultured_bacterium_f_Muribaculaceae |
| OTU14 | + | + | + | k__Bacteria; p__Proteobacteria; c__Deltaproteobacteria; o__Desulfovibrionales; f__Desulfovibrionaceae; g__Desulfovibrio; s__Desulfovibrio_fairfieldensis |
| OTU16 | + | + | + | k__Bacteria; p__Firmicutes; c__Clostridia; o__Clostridiales; f__Lachnospiraceae; g__Lachnospiraceae_NK4A136_group; s__Lachnospiraceae_bacterium_10-1 |
| OTU22 | + | + | + | k__Bacteria; p__Firmicutes; c__Clostridia; o__Clostridiales; f__Ruminococcaceae; g__Ruminiclostridium_5; s__uncultured_bacterium_g_Ruminiclostridium_5 |
| OTU90 | + | + | + | k__Bacteria; p__Bacteroidetes; c__Bacteroidia; o__Bacteroidales; f__Muribaculaceae; g__uncultured_bacterium_f_Muribaculaceae; s__uncultured_bacterium_f_Muribaculaceae |
| OTU23 | + | + | + | k__Bacteria; p__Firmicutes; c__Clostridia; o__Clostridiales; f__Lachnospiraceae; g__Lachnospiraceae_NK4A136_group; s__uncultured_bacterium_g_Lachnospiraceae_NK4A136_group |
| OTU43 | + | + | + | k__Bacteria; p__Firmicutes; c__Clostridia; o__Clostridiales; f__Lachnospiraceae; g__uncultured_bacterium_f_Lachnospiraceae; s__uncultured_bacterium_f_Lachnospiraceae |
| OTU20 | + | + | + | k__Bacteria; p__Firmicutes; c__Clostridia; o__Clostridiales; f__Lachnospiraceae; g__Lachnospiraceae_UCG-006; s__uncultured_bacterium_g_Lachnospiraceae_UCG-006 |
| OTU41 | + | + | + | k__Bacteria; p__Firmicutes; c__Clostridia; o__Clostridiales; f__Lachnospiraceae; g__Lachnospiraceae_NK4A136_group; s__Clostridium_sp |
| OTU27 | + | + | + | k__Bacteria; p__Bacteroidetes; c__Bacteroidia; o__Bacteroidales; f__Bacteroidaceae; g__Bacteroides; s__Bacteroides_acidifaciens |
| OTU40 | + | + | + | k__Bacteria; p__Firmicutes; c__Clostridia; o__Clostridiales; f__Lachnospiraceae; g__Lachnoclostridium; s__uncultured_bacterium_g_Lachnoclostridium |
| OTU19 | + | + | + | k__Bacteria; p__Bacteroidetes; c__Bacteroidia; o__Bacteroidales; f__Prevotellaceae; g__Alloprevotella; s__uncultured_bacterium_g_Alloprevotella |
| OTU82 | + | + | + | k__Bacteria; p__Bacteroidetes; c__Bacteroidia; o__Bacteroidales; f__Muribaculaceae; g__uncultured_bacterium_f_Muribaculaceae; s__uncultured_bacterium_f_Muribaculaceae |
| OTU26 | + | + | + | k__Bacteria; p__Bacteroidetes; c__Bacteroidia; o__Bacteroidales; f__Marinifilaceae; g__Odoribacter; s__uncultured_bacterium_g_Odoribacter |
| OTU55 | + | + | + | k__Bacteria; p__Bacteroidetes; c__Bacteroidia; o__Bacteroidales; f__Muribaculaceae; g__uncultured_bacterium_f_Muribaculaceae; s__uncultured_bacterium_f_Muribaculaceae |
| OTU6 | + | + | + | k__Bacteria; p__Firmicutes; c__Clostridia; o__Clostridiales; f__Caloramatoraceae; g__Clostridium; s__Clostridium_cocleatum |
| OTU50 | + | + | + | k__Bacteria; p__Proteobacteria; c__Deltaproteobacteria; o__Desulfovibrionales; f__Desulfovibrionaceae; g__uncultured_bacterium_f_Desulfovibrionaceae; s__uncultured_bacterium_f_Desulfovibrionaceae |
| OTU31 | + | + | + | k__Bacteria; p__Epsilonbacteraeota; c__Campylobacteria; o__Campylobacterales; f__Helicobacteraceae; g__Helicobacter; s__Helicobacter_bilis |
| OTU37 | + | + | + | k__Bacteria; p__Deferribacteres; c__Deferribacteres; o__Deferribacterales; f__Deferribacteraceae; g__Mucispirillum; s__Mucispirillum_sp |
| OTU47 | + | + | + | k__Bacteria; p__Bacteroidetes; c__Bacteroidia; o__Bacteroidales; f__Muribaculaceae; g__uncultured_bacterium_f_Muribaculaceae; s__uncultured_bacterium_f_Muribaculaceae |
| OTU42 | + | + | + | k__Bacteria; p__Bacteroidetes; c__Bacteroidia; o__Bacteroidales; f__Muribaculaceae; g__uncultured_bacterium_f_Muribaculaceae; s__uncultured_bacterium_f_Muribaculaceae |
| OTU78 | + | + | + | k__Bacteria; p__Bacteroidetes; c__Bacteroidia; o__Bacteroidales; f__Muribaculaceae; g__uncultured_bacterium_f_Muribaculaceae; s__uncultured_bacterium_f_Muribaculaceae |
| OTU66 | + | + | + | k__Bacteria; p__Bacteroidetes; c__Bacteroidia; o__Bacteroidales; f__Rikenellaceae; g__Rikenellaceae_RC9_gut_group; s__uncultured_bacterium_g_Rikenellaceae_RC9_gut_group |
| OTU101 | + | + | + | k__Bacteria; p__Firmicutes; c__Clostridia; o__Clostridiales; f__Lachnospiraceae; g__Lachnospiraceae_NK4A136_group; s__uncultured_bacterium_g_Lachnospiraceae_NK4A136_group |
| OTU52 | + | + | + | k__Bacteria; p__Bacteroidetes; c__Bacteroidia; o__Bacteroidales; f__Bacteroidaceae; g__Bacteroides; s__Bacteroides_sartorii |
| OTU76 | + | + | + | k__Bacteria; p__Bacteroidetes; c__Bacteroidia; o__Bacteroidales; f__Muribaculaceae; g__uncultured_bacterium_f_Muribaculaceae; s__uncultured_bacterium_f_Muribaculaceae |
| OTU79 | + | + | + | k__Bacteria; p__Bacteroidetes; c__Bacteroidia; o__Bacteroidales; f__Prevotellaceae; g__Prevotellaceae_UCG-001; s__uncultured_bacterium_g_Prevotellaceae_UCG-001 |
| OTU58 | + | + | + | k__Bacteria; p__Patescibacteria; c__Saccharimonadia; o__Saccharimonadales; f__Saccharimonadaceae; g__Candidatus_Saccharimonas; s__uncultured_bacterium_g_Candidatus_Saccharimonas |
| OTU36 | + | + | + | k__Bacteria; p__Firmicutes; c__Clostridia; o__Clostridiales; f__Lachnospiraceae; g__Lachnoclostridium; s__uncultured_bacterium_g_Lachnoclostridium |
| OTU80 | + | + | + | k__Bacteria; p__Bacteroidetes; c__Bacteroidia; o__Bacteroidales; f__Muribaculaceae; g__uncultured_bacterium_f_Muribaculaceae; s__uncultured_bacterium_f_Muribaculaceae |
| OTU176 | + | + | + | k__Bacteria; p__Firmicutes; c__Clostridia; o__Clostridiales; f__Lachnospiraceae; g__Lachnospiraceae_NK4A136_group; s__uncultured_bacterium_g_Lachnospiraceae_NK4A136_group |
| OTU3 | + | + | + | k__Bacteria; p__Firmicutes; c__Bacilli; o__Lactobacillales; f__Lactobacillaceae; g__Lactobacillus; s__Lactobacillus_taiwanensis |
| OTU53 | + | + | + | k__Bacteria; p__Firmicutes; c__Clostridia; o__Clostridiales; f__Lachnospiraceae; g__uncultured_bacterium_f_Lachnospiraceae; s__uncultured_bacterium_f_Lachnospiraceae |
| OTU32 | + | + | + | k__Bacteria; p__Actinobacteria; c__Coriobacteriia; o__Coriobacteriales; f__Eggerthellaceae; g__Enterorhabdus; s__uncultured_bacterium_g_Enterorhabdus |
| OTU140 | + | + | + | k__Bacteria; p__Firmicutes; c__Clostridia; o__Clostridiales; f__Lachnospiraceae; g__uncultured_bacterium_f_Lachnospiraceae; s__uncultured_bacterium_f_Lachnospiraceae |
| OTU64 | + | + | + | k__Bacteria; p__Bacteroidetes; c__Bacteroidia; o__Bacteroidales; f__Muribaculaceae; g__uncultured_bacterium_f_Muribaculaceae; s__uncultured_bacterium_f_Muribaculaceae |
| OTU170 | + | + | + | k__Bacteria; p__Firmicutes; c__Clostridia; o__Clostridiales; f__Lachnospiraceae; g__uncultured_bacterium_f_Lachnospiraceae; s__uncultured_bacterium_f_Lachnospiraceae |
| OTU107 | + | + | + | k__Bacteria; p__Bacteroidetes; c__Bacteroidia; o__Bacteroidales; f__Bacteroidaceae; g__Bacteroides; s__Bacteroides_acidifaciens |
| OTU56 | + | + | + | k__Bacteria; p__Firmicutes; c__Clostridia; o__Clostridiales; f__Lachnospiraceae; g__Lachnospiraceae_NK4A136_group; s__uncultured_bacterium_g_Lachnospiraceae_NK4A136_group |
| OTU34 | + | + | + | k__Bacteria; p__Firmicutes; c__Clostridia; o__Clostridiales; f__Lachnospiraceae; g__uncultured_bacterium_f_Lachnospiraceae; s__uncultured_bacterium_f_Lachnospiraceae |
| OTU51 | + | + | + | k__Bacteria; p__Bacteroidetes; c__Bacteroidia; o__Bacteroidales; f__Rikenellaceae; g__Alistipes; s__uncultured_bacterium_g_Alistipes |
| OTU153 | + | + | + | k__Bacteria; p__Bacteroidetes; c__Bacteroidia; o__Bacteroidales; f__Muribaculaceae; g__uncultured_bacterium_f_Muribaculaceae; s__uncultured_bacterium_f_Muribaculaceae |
| OTU177 | + | + | + | k__Bacteria; p__Firmicutes; c__Clostridia; o__Clostridiales; f__Lachnospiraceae; g__uncultured_bacterium_f_Lachnospiraceae; s__uncultured_bacterium_f_Lachnospiraceae |
| OTU183 | + | + | + | k__Bacteria; p__Bacteroidetes; c__Bacteroidia; o__Bacteroidales; f__Muribaculaceae; g__uncultured_bacterium_f_Muribaculaceae; s__uncultured_bacterium_f_Muribaculaceae |
| OTU94 | + | + | + | k__Bacteria; p__Bacteroidetes; c__Bacteroidia; o__Bacteroidales; f__Muribaculaceae; g__uncultured_bacterium_f_Muribaculaceae; s__uncultured_bacterium_f_Muribaculaceae |
| OTU126 | + | + | + | k__Bacteria; p__Bacteroidetes; c__Bacteroidia; o__Bacteroidales; f__Muribaculaceae; g__uncultured_bacterium_f_Muribaculaceae; s__uncultured_bacterium_f_Muribaculaceae |
| OTU81 | + | + | + | k__Bacteria; p__Firmicutes; c__Erysipelotrichia; o__Erysipelotrichales; f__Erysipelotrichaceae; g__uncultured_bacterium_f_Erysipelotrichaceae; s__uncultured_bacterium_f_Erysipelotrichaceae |
| OTU87 | + | + | + | k__Bacteria; p__Firmicutes; c__Clostridia; o__Clostridiales; f__Ruminococcaceae; g__Ruminiclostridium_9; s__uncultured_bacterium_g_Ruminiclostridium_9 |
| OTU73 | + | + | + | k__Bacteria; p__Bacteroidetes; c__Bacteroidia; o__Bacteroidales; f__Muribaculaceae; g__uncultured_bacterium_f_Muribaculaceae; s__uncultured_bacterium_f_Muribaculaceae |
| OTU39 | + | + | + | k__Bacteria; p__Bacteroidetes; c__Bacteroidia; o__uncultured_bacterium_c_Bacteroidia; f__uncultured_bacterium_c_Bacteroidia; g__uncultured_bacterium_c_Bacteroidia; s__uncultured_bacterium_c_Bacteroidia |
| OTU118 | + | + | + | k__Bacteria; p__Bacteroidetes; c__Bacteroidia; o__Bacteroidales; f__Muribaculaceae; g__uncultured_bacterium_f_Muribaculaceae; s__uncultured_bacterium_f_Muribaculaceae |
| OTU45 | + | + | + | k__Bacteria; p__Firmicutes; c__Clostridia; o__Clostridiales; f__Lachnospiraceae; g__uncultured_bacterium_f_Lachnospiraceae; s__uncultured_bacterium_f_Lachnospiraceae |
| OTU129 | + | + | + | k__Bacteria; p__Bacteroidetes; c__Bacteroidia; o__Bacteroidales; f__Rikenellaceae; g__Alistipes; s__uncultured_bacterium_g_Alistipes |
| OTU122 | + | + | + | k__Bacteria; p__Bacteroidetes; c__Bacteroidia; o__Bacteroidales; f__Rikenellaceae; g__Rikenella; s__uncultured_bacterium_g_Rikenella |
| OTU59 | + | + | + | k__Bacteria; p__Firmicutes; c__Clostridia; o__Clostridiales; f__Lachnospiraceae; g__uncultured_bacterium_f_Lachnospiraceae; s__uncultured_bacterium_f_Lachnospiraceae |
| OTU131 | + | + | + | k__Bacteria; p__Bacteroidetes; c__Bacteroidia; o__Bacteroidales; f__Rikenellaceae; g__Alistipes; s__uncultured_bacterium_g_Alistipes |
| OTU137 | + | + | + | k__Bacteria; p__Firmicutes; c__Clostridia; o__Clostridiales; f__Ruminococcaceae; g__Ruminococcaceae_UCG-013; s__uncultured_bacterium_g_Ruminococcaceae_UCG-013 |
| OTU97 | - | + | + | k__Bacteria; p__Firmicutes; c__Clostridia; o__Clostridiales; f__Lachnospiraceae; g__Lachnoclostridium; s__uncultured_bacterium_g_Lachnoclostridium |
| OTU72 | + | + | + | k__Bacteria; p__Firmicutes; c__Clostridia; o__Clostridiales; f__Lachnospiraceae; g__Lachnospiraceae_NK4A136_group; s__uncultured_bacterium_g_Lachnospiraceae_NK4A136_group |
| OTU92 | + | + | + | k__Bacteria; p__Patescibacteria; c__Saccharimonadia; o__Saccharimonadales; f__Saccharimonadaceae; g__Candidatus_Saccharimonas; s__uncultured_bacterium_g_Candidatus_Saccharimonas |
| OTU70 | - | + | + | k__Bacteria; p__Firmicutes; c__Erysipelotrichia; o__Erysipelotrichales; f__Erysipelotrichaceae; g__uncultured_bacterium_f_Erysipelotrichaceae; s__uncultured_bacterium_f_Erysipelotrichaceae |
| OTU168 | + | + | + | k__Bacteria; p__Bacteroidetes; c__Bacteroidia; o__Bacteroidales; f__Muribaculaceae; g__uncultured_bacterium_f_Muribaculaceae; s__uncultured_bacterium_f_Muribaculaceae |
| OTU35 | + | + | + | k__Bacteria; p__Proteobacteria; c__Deltaproteobacteria; o__Desulfovibrionales; f__Desulfovibrionaceae; g__Desulfovibrio; s__uncultured_bacterium_g_Desulfovibrio |
| OTU218 | + | + | + | k__Bacteria; p__Bacteroidetes; c__Bacteroidia; o__Bacteroidales; f__Muribaculaceae; g__uncultured_bacterium_f_Muribaculaceae; s__uncultured_bacterium_f_Muribaculaceae |
| OTU117 | + | + | + | k__Bacteria; p__Firmicutes; c__Clostridia; o__Clostridiales; f__Ruminococcaceae; g__Ruminococcaceae_UCG-014; s__uncultured_bacterium_g_Ruminococcaceae_UCG-014 |
| OTU46 | + | - | + | k__Bacteria; p__Firmicutes; c__Clostridia; o__Clostridiales; f__Ruminococcaceae; g__Ruminococcaceae_UCG-014; s__uncultured_bacterium_g_Ruminococcaceae_UCG-014 |
| OTU121 | + | + | + | k__Bacteria; p__Actinobacteria; c__Coriobacteriia; o__Coriobacteriales; f__Eggerthellaceae; g__Enterorhabdus; s__uncultured_bacterium_g_Enterorhabdus |
| OTU48 | + | + | + | k__Bacteria; p__Firmicutes; c__Clostridia; o__Clostridiales; f__Lachnospiraceae; g__uncultured_bacterium_f_Lachnospiraceae; s__uncultured_bacterium_f_Lachnospiraceae |
| OTU133 | + | + | + | k__Bacteria; p__Bacteroidetes; c__Bacteroidia; o__Bacteroidales; f__Muribaculaceae; g__uncultured_bacterium_f_Muribaculaceae; s__uncultured_bacterium_f_Muribaculaceae |
| OTU119 | + | + | + | k__Bacteria; p__Bacteroidetes; c__Bacteroidia; o__Bacteroidales; f__Bacteroidaceae; g__Bacteroides; s__Bacteroides_acidifaciens |
| OTU102 | + | + | + | k__Bacteria; p__Firmicutes; c__Clostridia; o__Clostridiales; f__Ruminococcaceae; g__Anaerotruncus; s__Anaerotruncus_sp |
| OTU24 | + | + | + | k__Bacteria; p__Firmicutes; c__Clostridia; o__Clostridiales; f__Clostridiaceae_1; g__Candidatus_Arthromitus; s__Candidatus_Arthromitus_sp |
| OTU132 | + | + | + | k__Bacteria; p__Firmicutes; c__Clostridia; o__Clostridiales; f__Lachnospiraceae; g__GCA-900066575; s__uncultured_bacterium_g_GCA-900066575 |
| OTU12 | + | + | + | k__Bacteria; p__Firmicutes; c__Clostridia; o__Clostridiales; f__Lachnospiraceae; g__Lachnospiraceae_NK4A136_group; s__uncultured_bacterium_g_Lachnospiraceae_NK4A136_group |
| OTU220 | - | + | + | k__Bacteria; p__Bacteroidetes; c__Bacteroidia; o__Bacteroidales; f__Tannerellaceae; g__Parabacteroides; s__Parabacteroides_distasonis |
| OTU155 | - | - | + | k__Bacteria; p__Firmicutes; c__Clostridia; o__Clostridiales; f__Lachnospiraceae; g__[Ruminococcus]_torques_group; s__uncultured_bacterium_g_[Ruminococcus]_torques_group |
| OTU191 | + | + | + | k__Bacteria; p__Firmicutes; c__Clostridia; o__Clostridiales; f__Lachnospiraceae; g__uncultured_bacterium_f_Lachnospiraceae; s__uncultured_bacterium_f_Lachnospiraceae |
| OTU146 | + | + | + | k__Bacteria; p__Firmicutes; c__Clostridia; o__Clostridiales; f__Lachnospiraceae; g__Lachnoclostridium; s__Clostridium_fusiformis |
| OTU103 | + | + | + | k__Bacteria; p__Firmicutes; c__Clostridia; o__Clostridiales; f__Ruminococcaceae; g__Acutalibacter; s__Acutalibacter_muris |
| OTU74 | + | + | + | k__Bacteria; p__Proteobacteria; c__Deltaproteobacteria; o__Desulfovibrionales; f__Desulfovibrionaceae; g__Desulfovibrio; s__uncultured_bacterium_g_Desulfovibrio |
| OTU207 | + | + | + | k__Bacteria; p__Bacteroidetes; c__Bacteroidia; o__Bacteroidales; f__Muribaculaceae; g__Muribaculum; s__Muribaculum_intestinale |
| OTU167 | + | + | + | k__Bacteria; p__Bacteroidetes; c__Bacteroidia; o__Bacteroidales; f__Muribaculaceae; g__uncultured_bacterium_f_Muribaculaceae; s__uncultured_bacterium_f_Muribaculaceae |
| OTU124 | + | + | + | k__Bacteria; p__Firmicutes; c__Clostridia; o__Clostridiales; f__Ruminococcaceae; g__Ruminiclostridium_9; s__uncultured_bacterium_g_Ruminiclostridium_9 |
| OTU109 | + | + | + | k__Bacteria; p__Bacteroidetes; c__Bacteroidia; o__Bacteroidales; f__Tannerellaceae; g__Parabacteroides; s__uncultured_bacterium_g_Parabacteroides |
| OTU136 | + | + | + | k__Bacteria; p__Bacteroidetes; c__Bacteroidia; o__Bacteroidales; f__Rikenellaceae; g__Tidjanibacter; s__Tidjanibacter_massiliensis |
| OTU138 | + | + | + | k__Bacteria; p__Firmicutes; c__Clostridia; o__Clostridiales; f__Ruminococcaceae; g__Ruminococcaceae_UCG-010; s__uncultured_bacterium_g_Ruminococcaceae_UCG-010 |
| OTU198 | - | + | + | k__Bacteria; p__Firmicutes; c__Clostridia; o__Clostridiales; f__Lachnospiraceae; g__[Ruminococcus]_torques_group; s__uncultured_bacterium_g_[Ruminococcus]_torques_group |
| OTU83 | + | + | + | k__Bacteria; p__Tenericutes; c__Mollicutes; o__Mollicutes_RF39; f__uncultured_bacterium_o_Mollicutes_RF39; g__uncultured_bacterium_o_Mollicutes_RF39; s__uncultured_bacterium_o_Mollicutes_RF39 |
| OTU99 | + | + | + | k__Bacteria; p__Firmicutes; c__Clostridia; o__Clostridiales; f__Family_XIII; g__[Eubacterium]_nodatum_group; s__uncultured_bacterium_g_[Eubacterium]_nodatum_group |
| OTU89 | + | + | + | k__Bacteria; p__Firmicutes; c__Clostridia; o__Clostridiales; f__Lachnospiraceae; g__Lachnoclostridium; s__uncultured_bacterium_g_Lachnoclostridium |
| OTU210 | + | + | + | k__Bacteria; p__Actinobacteria; c__Coriobacteriia; o__Coriobacteriales; f__Eggerthellaceae; g__Enterorhabdus; s__Enterorhabdus_caecimuris |
| OTU112 | + | + | + | k__Bacteria; p__Tenericutes; c__Mollicutes; o__Mollicutes_RF39; f__uncultured_bacterium_o_Mollicutes_RF39; g__uncultured_bacterium_o_Mollicutes_RF39; s__uncultured_bacterium_o_Mollicutes_RF39 |
| OTU185 | + | - | + | k__Bacteria; p__Firmicutes; c__Clostridia; o__Clostridiales; f__Ruminococcaceae; g__Ruminococcaceae_UCG-014; s__uncultured_bacterium_g_Ruminococcaceae_UCG-014 |
| OTU29 | + | + | + | k__Bacteria; p__Bacteroidetes; c__Bacteroidia; o__Bacteroidales; f__Prevotellaceae; g__Prevotellaceae_UCG-003; s__uncultured_bacterium_g_Prevotellaceae_UCG-003 |
| OTU184 | + | + | + | k__Bacteria; p__Bacteroidetes; c__Bacteroidia; o__Bacteroidales; f__Bacteroidaceae; g__Bacteroides; s__Bacteroides_intestinalis |
| OTU111 | + | - | + | k__Bacteria; p__Tenericutes; c__Mollicutes; o__Mollicutes_RF39; f__uncultured_bacterium_o_Mollicutes_RF39; g__uncultured_bacterium_o_Mollicutes_RF39; s__uncultured_bacterium_o_Mollicutes_RF39 |
| OTU244 | + | + | + | k__Bacteria; p__Firmicutes; c__Clostridia; o__Clostridiales; f__Lachnospiraceae; g__Lachnospiraceae_NK4A136_group; s__uncultured_bacterium_g_Lachnospiraceae_NK4A136_group |
| OTU104 | + | + | + | k__Bacteria; p__Proteobacteria; c__Deltaproteobacteria; o__Desulfovibrionales; f__Desulfovibrionaceae; g__Bilophila; s__uncultured_bacterium_g_Bilophila |
| OTU38 | + | + | + | k__Bacteria; p__Firmicutes; c__Clostridia; o__Clostridiales; f__Ruminococcaceae; g__uncultured_bacterium_f_Ruminococcaceae; s__uncultured_bacterium_f_Ruminococcaceae |
| OTU156 | - | + | + | k__Bacteria; p__Firmicutes; c__Clostridia; o__Clostridiales; f__Ruminococcaceae; g__Ruminiclostridium_6; s__uncultured_bacterium_g_Ruminiclostridium_6 |
| OTU123 | + | + | + | k__Bacteria; p__Patescibacteria; c__Saccharimonadia; o__Saccharimonadales; f__Saccharimonadaceae; g__Candidatus_Saccharimonas; s__uncultured_bacterium_g_Candidatus_Saccharimonas |
| OTU217 | + | + | + | k__Bacteria; p__Firmicutes; c__Clostridia; o__Clostridiales; f__Ruminococcaceae; g__Negativibacillus; s__uncultured_bacterium_g_Negativibacillus |
| OTU85 | - | + | + | k__Bacteria; p__Firmicutes; c__Clostridia; o__Clostridiales; f__Ruminococcaceae; g__Ruminiclostridium_5; s__uncultured_bacterium_g_Ruminiclostridium_5 |
| OTU257 | + | + | + | k__Bacteria; p__Firmicutes; c__Clostridia; o__Clostridiales; f__Lachnospiraceae; g__uncultured_bacterium_f_Lachnospiraceae; s__uncultured_bacterium_f_Lachnospiraceae |
| OTU143 | + | + | + | k__Bacteria; p__Firmicutes; c__Clostridia; o__Clostridiales; f__Ruminococcaceae; g__Ruminococcaceae_UCG-004; s__uncultured_bacterium_g_Ruminococcaceae_UCG-004 |
| OTU225 | + | + | + | k__Bacteria; p__Cyanobacteria; c__Oxyphotobacteria; o__Chloroplast; f__uncultured_bacterium_o_Chloroplast; g__uncultured_bacterium_o_Chloroplast; s__uncultured_bacterium_o_Chloroplast |
| OTU141 | + | + | + | k__Bacteria; p__Bacteroidetes; c__Bacteroidia; o__Bacteroidales; f__Marinifilaceae; g__Odoribacter; s__uncultured_bacterium_g_Odoribacter |
| OTU71 | + | + | + | k__Bacteria; p__Firmicutes; c__Clostridia; o__Clostridiales; f__Family_XIII; g__Family_XIII_AD3011_group; s__uncultured_bacterium_g_Family_XIII_AD3011_group |
| OTU127 | + | + | + | k__Bacteria; p__Bacteroidetes; c__Bacteroidia; o__Bacteroidales; f__Muribaculaceae; g__uncultured_bacterium_f_Muribaculaceae; s__uncultured_bacterium_f_Muribaculaceae |
| OTU188 | - | + | + | k__Bacteria; p__Proteobacteria; c__Gammaproteobacteria; o__Enterobacterales; f__Enterobacteriaceae; g__Escherichia; s__Escherichia_coli |
| OTU192 | + | + | + | k__Bacteria; p__Proteobacteria; c__Gammaproteobacteria; o__Betaproteobacteriales; f__Burkholderiaceae; g__Parasutterella; s__uncultured_bacterium_g_Parasutterella |
| OTU63 | + | + | + | k__Bacteria; p__Firmicutes; c__Clostridia; o__Clostridiales; f__Ruminococcaceae; g__Ruminococcaceae_UCG-014; s__uncultured_bacterium_g_Ruminococcaceae_UCG-014 |
| OTU149 | + | + | + | k__Bacteria; p__Bacteroidetes; c__Bacteroidia; o__Bacteroidales; f__Muribaculaceae; g__uncultured_bacterium_f_Muribaculaceae; s__uncultured_bacterium_f_Muribaculaceae |
| OTU134 | + | + | + | k__Bacteria; p__Firmicutes; c__Clostridia; o__Clostridiales; f__Lachnospiraceae; g__uncultured_bacterium_f_Lachnospiraceae; s__uncultured_bacterium_f_Lachnospiraceae |
| OTU91 | + | + | + | k__Bacteria; p__Firmicutes; c__Clostridia; o__Clostridiales; f__Lachnospiraceae; g__uncultured_bacterium_f_Lachnospiraceae; s__uncultured_bacterium_f_Lachnospiraceae |
| OTU158 | + | + | + | k__Bacteria; p__Bacteroidetes; c__Bacteroidia; o__Bacteroidales; f__Rikenellaceae; g__Rikenella; s__uncultured_bacterium_g_Rikenella |
| OTU263 | + | + | + | k__Bacteria; p__Firmicutes; c__Clostridia; o__Clostridiales; f__Lachnospiraceae; g__uncultured_bacterium_f_Lachnospiraceae; s__uncultured_bacterium_f_Lachnospiraceae |
| OTU256 | - | + | + | k__Bacteria; p__Bacteroidetes; c__Bacteroidia; o__Bacteroidales; f__Bacteroidaceae; g__Bacteroides; s__Bacteroides_thetaiotaomicron |
| OTU241 | + | - | + | k__Bacteria; p__Firmicutes; c__Clostridia; o__Clostridiales; f__Ruminococcaceae; g__Ruminococcaceae_UCG-014; s__uncultured_bacterium_g_Ruminococcaceae_UCG-014 |
| OTU150 | + | + | + | k__Bacteria; p__Firmicutes; c__Clostridia; o__Clostridiales; f__Lachnospiraceae; g__Blautia; s__Lachnospiraceae_bacterium_610 |
| OTU282 | - | + | + | k__Bacteria; p__Firmicutes; c__Bacilli; o__Lactobacillales; f__Leuconostocaceae; g__Weissella; s__Weissella_cibaria |
| OTU95 | + | + | + | k__Bacteria; p__Firmicutes; c__Clostridia; o__Clostridiales; f__Lachnospiraceae; g__uncultured_bacterium_f_Lachnospiraceae; s__uncultured_bacterium_f_Lachnospiraceae |
| OTU169 | - | + | + | k__Bacteria; p__Actinobacteria; c__Coriobacteriia; o__Coriobacteriales; f__Eggerthellaceae; g__Enterorhabdus; s__uncultured_bacterium_g_Enterorhabdus |
| OTU186 | - | + | + | k__Bacteria; p__Firmicutes; c__Clostridia; o__Clostridiales; f__Peptococcaceae; g__uncultured_bacterium_f_Peptococcaceae; s__uncultured_bacterium_f_Peptococcaceae |
| OTU160 | + | + | + | k__Bacteria; p__Firmicutes; c__Clostridia; o__Clostridiales; f__Peptococcaceae; g__uncultured_bacterium_f_Peptococcaceae; s__uncultured_bacterium_f_Peptococcaceae |
| OTU152 | + | + | + | k__Bacteria; p__Bacteroidetes; c__Bacteroidia; o__Bacteroidales; f__Tannerellaceae; g__Parabacteroides; s__Parabacteroides_goldsteinii |
| OTU75 | - | + | + | k__Bacteria; p__Bacteroidetes; c__Bacteroidia; o__Bacteroidales; f__Bacteroidaceae; g__Bacteroides; s__Bacteroides_vulgatus |
| OTU21 | + | + | + | k__Bacteria; p__Firmicutes; c__Clostridia; o__Clostridiales; f__Ruminococcaceae; g__[Eubacterium]_coprostanoligenes_group; s__uncultured_bacterium_g_[Eubacterium]_coprostanoligenes_group |
| OTU247 | + | + | + | k__Bacteria; p__Firmicutes; c__Clostridia; o__Clostridiales; f__Family_XIII; g__Family_XIII_UCG-001; s__uncultured_bacterium_g_Family_XIII_UCG-001 |
| OTU98 | + | + | + | k__Bacteria; p__Bacteroidetes; c__Bacteroidia; o__Bacteroidales; f__Muribaculaceae; g__uncultured_bacterium_f_Muribaculaceae; s__uncultured_bacterium_f_Muribaculaceae |
| OTU145 | + | + | + | k__Bacteria; p__Firmicutes; c__Clostridia; o__Clostridiales; f__Ruminococcaceae; g__Ruminococcaceae_NK4A214_group; s__uncultured_bacterium_g_Ruminococcaceae_NK4A214_group |
| OTU61 | + | + | + | k__Bacteria; p__Bacteroidetes; c__Bacteroidia; o__Bacteroidales; f__Muribaculaceae; g__uncultured_bacterium_f_Muribaculaceae; s__uncultured_bacterium_f_Muribaculaceae |
| OTU162 | + | + | + | k__Bacteria; p__Firmicutes; c__Clostridia; o__Clostridiales; f__Lachnospiraceae; g__uncultured_bacterium_f_Lachnospiraceae; s__uncultured_bacterium_f_Lachnospiraceae |
| OTU224 | + | + | + | k__Bacteria; p__Firmicutes; c__Clostridia; o__Clostridiales; f__Lachnospiraceae; g__uncultured_bacterium_f_Lachnospiraceae; s__uncultured_bacterium_f_Lachnospiraceae |
| OTU190 | + | + | + | k__Bacteria; p__Bacteroidetes; c__Bacteroidia; o__Bacteroidales; f__Rikenellaceae; g__Alistipes; s__uncultured_bacterium_g_Alistipes |
| OTU240 | - | + | + | k__Bacteria; p__Firmicutes; c__Clostridia; o__Clostridiales; f__Lachnospiraceae; g__uncultured_bacterium_f_Lachnospiraceae; s__uncultured_bacterium_f_Lachnospiraceae |
| OTU139 | - | + | + | k__Bacteria; p__Proteobacteria; c__Gammaproteobacteria; o__Enterobacteriales; f__Enterobacteriaceae; g__Klebsiella; s__Klebsiella_pneumoniae |
| OTU77 | + | + | + | k__Bacteria; p__Firmicutes; c__Clostridia; o__Clostridiales; f__Lachnospiraceae; g__uncultured_bacterium_f_Lachnospiraceae; s__uncultured_bacterium_f_Lachnospiraceae |
| OTU144 | + | + | + | k__Bacteria; p__Firmicutes; c__Clostridia; o__Clostridiales; f__Lachnospiraceae; g__Blautia; s__Blautia_coccoides |
| OTU212 | + | + | + | k__Bacteria; p__Firmicutes; c__Clostridia; o__Clostridiales; f__Ruminococcaceae; g__Ruminococcaceae_UCG-014; s__uncultured_bacterium_g_Ruminococcaceae_UCG-014 |
| OTU108 | + | + | + | k__Bacteria; p__Bacteroidetes; c__Bacteroidia; o__Bacteroidales; f__Muribaculaceae; g__uncultured_bacterium_f_Muribaculaceae; s__uncultured_bacterium_f_Muribaculaceae |
| OTU306 | + | + | + | k__Bacteria; p__Actinobacteria; c__Coriobacteriia; o__Coriobacteriales; f__Eggerthellaceae; g__Enterorhabdus; s__uncultured_bacterium_g_Enterorhabdus |
| OTU283 | + | + | + | k__Bacteria; p__Firmicutes; c__Clostridia; o__Clostridiales; f__Ruminococcaceae; g__Anaerotruncus; s__Anaerotruncus_colihominis |
| OTU62 | + | + | + | k__Bacteria; p__Firmicutes; c__Clostridia; o__Clostridiales; f__Ruminococcaceae; g__Ruminococcaceae_UCG-014; s__uncultured_bacterium_g_Ruminococcaceae_UCG-014 |
| OTU284 | + | + | + | k__Bacteria; p__Actinobacteria; c__Coriobacteriia; o__Coriobacteriales; f__Eggerthellaceae; g__Parvibacter; s__Parvibacter_caecicola |
| OTU204 | + | + | + | k__Bacteria; p__Tenericutes; c__Mollicutes; o__Mollicutes_RF39; f__uncultured_bacterium_o_Mollicutes_RF39; g__uncultured_bacterium_o_Mollicutes_RF39; s__uncultured_bacterium_o_Mollicutes_RF39 |
| OTU285 | - | + | + | k__Bacteria; p__Firmicutes; c__Clostridia; o__Clostridiales; f__Ruminococcaceae; g__Ruminiclostridium_5; s__uncultured_bacterium_g_Ruminiclostridium_5 |
| OTU293 | - | + | + | k__Bacteria; p__Firmicutes; c__Clostridia; o__Clostridiales; f__Lachnospiraceae; g__[Eubacterium]_xylanophilum_group; s__uncultured_bacterium_g_[Eubacterium]_xylanophilum_group |
| OTU223 | + | - | + | k__Bacteria; p__Bacteroidetes; c__Bacteroidia; o__Bacteroidales; f__Muribaculaceae; g__uncultured_bacterium_f_Muribaculaceae; s__uncultured_bacterium_f_Muribaculaceae |
| OTU291 | - | - | + | k__Bacteria; p__Tenericutes; c__Mollicutes; o__Mycoplasmatales; f__Mycoplasmataceae; g__Mycoplasma; s__Mycoplasma_sualvi |
| OTU135 | + | + | + | k__Bacteria; p__Actinobacteria; c__Coriobacteriia; o__Coriobacteriales; f__Eggerthellaceae; g__Enterorhabdus; s__uncultured_bacterium_g_Enterorhabdus |
| OTU178 | + | + | + | k__Bacteria; p__Bacteroidetes; c__Bacteroidia; o__Bacteroidales; f__Rikenellaceae; g__Alistipes; s__uncultured_bacterium_g_Alistipes |
| OTU235 | + | + | + | k__Bacteria; p__Bacteroidetes; c__Bacteroidia; o__Bacteroidales; f__Muribaculaceae; g__uncultured_bacterium_f_Muribaculaceae; s__uncultured_bacterium_f_Muribaculaceae |
| OTU175 | + | + | + | k__Bacteria; p__Bacteroidetes; c__Bacteroidia; o__Bacteroidales; f__Rikenellaceae; g__Alistipes; s__Alistipes_timonensis |
| OTU260 | + | + | + | k__Bacteria; p__Firmicutes; c__Clostridia; o__Clostridiales; f__Lachnospiraceae; g__uncultured_bacterium_f_Lachnospiraceae; s__uncultured_bacterium_f_Lachnospiraceae |
| OTU221 | + | + | + | k__Bacteria; p__Firmicutes; c__Clostridia; o__Clostridiales; f__Christensenellaceae; g__uncultured_bacterium_f_Christensenellaceae; s__uncultured_bacterium_f_Christensenellaceae |
| OTU289 | + | + | + | k__Bacteria; p__Firmicutes; c__Clostridia; o__Clostridiales; f__Clostridiales_vadinBB60_group; g__uncultured_bacterium_f_Clostridiales_vadinBB60_group; s__uncultured_bacterium_f_Clostridiales_vadinBB60_group |
| OTU286 | - | + | + | k__Bacteria; p__Firmicutes; c__Clostridia; o__Clostridiales; f__Ruminococcaceae; g__Papillibacter; s__uncultured_bacterium_g_Papillibacter |
| OTU310 | + | + | + | k__Bacteria; p__Actinobacteria; c__Coriobacteriia; o__Coriobacteriales; f__Eggerthellaceae; g__uncultured_bacterium_f_Eggerthellaceae; s__uncultured_bacterium_f_Eggerthellaceae |
| OTU200 | + | - | + | k__Bacteria; p__Tenericutes; c__Mollicutes; o__Mollicutes_RF39; f__uncultured_bacterium_o_Mollicutes_RF39; g__uncultured_bacterium_o_Mollicutes_RF39; s__uncultured_bacterium_o_Mollicutes_RF39 |
| OTU238 | + | - | + | k__Bacteria; p__Bacteroidetes; c__Bacteroidia; o__Bacteroidales; f__Rikenellaceae; g__Alistipes; s__uncultured_bacterium_g_Alistipes |
| OTU219 | + | - | + | k__Bacteria; p__Firmicutes; c__Clostridia; o__Clostridiales; f__Ruminococcaceae; g__uncultured_bacterium_f_Ruminococcaceae; s__uncultured_bacterium_f_Ruminococcaceae |
| OTU93 | - | - | + | k__Bacteria; p__Firmicutes; c__Clostridia; o__Clostridiales; f__Lachnospiraceae; g__uncultured_bacterium_f_Lachnospiraceae; s__uncultured_bacterium_f_Lachnospiraceae |
| OTU161 | - | + | + | k__Bacteria; p__Firmicutes; c__Clostridia; o__Clostridiales; f__Lachnospiraceae; g__uncultured_bacterium_f_Lachnospiraceae; s__uncultured_bacterium_f_Lachnospiraceae |
| OTU147 | + | + | + | k__Bacteria; p__Firmicutes; c__Clostridia; o__Clostridiales; f__Lachnospiraceae; g__Lachnoclostridium; s__uncultured_bacterium_g_Lachnoclostridium |
| OTU120 | + | + | + | k__Bacteria; p__Firmicutes; c__Bacilli; o__Lactobacillales; f__Streptococcaceae; g__Lactococcus; s__Lactococcus_garvieae |
| OTU199 | + | + | + | k__Bacteria; p__Firmicutes; c__Clostridia; o__Clostridiales; f__Lachnospiraceae; g__uncultured_bacterium_f_Lachnospiraceae; s__uncultured_bacterium_f_Lachnospiraceae |
| OTU142 | + | + | + | k__Bacteria; p__Firmicutes; c__Erysipelotrichia; o__Erysipelotrichales; f__Erysipelotrichaceae; g__Erysipelatoclostridium; s__uncultured_bacterium_g_Erysipelatoclostridium |
| OTU265 | + | + | + | k__Bacteria; p__Bacteroidetes; c__Bacteroidia; o__Bacteroidales; f__Muribaculaceae; g__uncultured_bacterium_f_Muribaculaceae; s__uncultured_bacterium_f_Muribaculaceae |
| OTU171 | + | + | + | k__Bacteria; p__Firmicutes; c__Bacilli; o__Lactobacillales; f__Streptococcaceae; g__Streptococcus; s__uncultured_bacterium_g_Streptococcus |
| OTU187 | + | + | + | k__Bacteria; p__Firmicutes; c__Clostridia; o__Clostridiales; f__Christensenellaceae; g__Christensenellaceae_R-7_group; s__uncultured_bacterium_g_Christensenellaceae_R-7_group |
| OTU309 | - | + | + | k__Bacteria; p__Firmicutes; c__Clostridia; o__Clostridiales; f__Ruminococcaceae; g__uncultured_bacterium_f_Ruminococcaceae; s__uncultured_bacterium_f_Ruminococcaceae |
| OTU202 | + | + | + | k__Bacteria; p__Firmicutes; c__Clostridia; o__Clostridiales; f__Ruminococcaceae; g__Ruminiclostridium_5; s__Clostridium_sp |
| OTU236 | + | + | + | k__Bacteria; p__Firmicutes; c__Clostridia; o__Clostridiales; f__Ruminococcaceae; g__GCA-900066225; s__uncultured_bacterium_g_GCA-900066225 |
| OTU319 | + | + | + | k__Bacteria; p__Firmicutes; c__Clostridia; o__Clostridiales; f__Lachnospiraceae; g__uncultured_bacterium_f_Lachnospiraceae; s__uncultured_bacterium_f_Lachnospiraceae |
| OTU258 | + | + | + | k__Bacteria; p__Fusobacteria; c__Fusobacteriia; o__Fusobacteriales; f__Fusobacteriaceae; g__Cetobacterium; s__Cetobacterium_somerae |
| OTU261 | + | + | + | k__Bacteria; p__Patescibacteria; c__Saccharimonadia; o__Saccharimonadales; f__Saccharimonadaceae; g__Candidatus_Saccharimonas; s__uncultured_bacterium_g_Candidatus_Saccharimonas |
| OTU166 | + | - | + | k__Bacteria; p__Bacteroidetes; c__Bacteroidia; o__Bacteroidales; f__Marinifilaceae; g__Odoribacter; s__uncultured_bacterium_g_Odoribacter |
| OTU313 | - | - | + | k__Bacteria; p__Bacteroidetes; c__Bacteroidia; o__Bacteroidales; f__Muribaculaceae; g__uncultured_bacterium_f_Muribaculaceae; s__uncultured_bacterium_f_Muribaculaceae |
| OTU68 | + | + | + | k__Bacteria; p__Firmicutes; c__Clostridia; o__Clostridiales; f__Lachnospiraceae; g__Lachnospiraceae_NK4A136_group; s__uncultured_bacterium_g_Lachnospiraceae_NK4A136_group |
| OTU86 | - | + | + | k__Bacteria; p__Firmicutes; c__Clostridia; o__Clostridiales; f__Lachnospiraceae; g__Blautia; s__Blautia_coccoides |
| OTU128 | + | + | + | k__Bacteria; p__Bacteroidetes; c__Bacteroidia; o__Bacteroidales; f__Rikenellaceae; g__Rikenellaceae_RC9_gut_group; s__uncultured_bacterium_g_Rikenellaceae_RC9_gut_group |
| OTU250 | + | + | + | k__Bacteria; p__Firmicutes; c__Clostridia; o__Clostridiales; f__Ruminococcaceae; g__Oscillibacter; s__uncultured_bacterium_g_Oscillibacter |
| OTU226 | + | + | + | k__Bacteria; p__Firmicutes; c__Clostridia; o__Clostridiales; f__Lachnospiraceae; g__Lachnospiraceae_NK4A136_group; s__uncultured_bacterium_g_Lachnospiraceae_NK4A136_group |
| OTU193 | - | + | + | k__Bacteria; p__Firmicutes; c__Clostridia; o__Clostridiales; f__Lachnospiraceae; g__uncultured_bacterium_f_Lachnospiraceae; s__uncultured_bacterium_f_Lachnospiraceae |
| OTU154 | + | + | + | k__Bacteria; p__Firmicutes; c__Clostridia; o__Clostridiales; f__Lachnospiraceae; g__Blautia; s__Blautia_coccoides |
| OTU216 | + | + | + | k__Bacteria; p__Firmicutes; c__Clostridia; o__Clostridiales; f__Ruminococcaceae; g__Ruminococcaceae_UCG-010; s__uncultured_bacterium_g_Ruminococcaceae_UCG-010 |
| OTU88 | + | + | + | k__Bacteria; p__Firmicutes; c__Clostridia; o__Clostridiales; f__Ruminococcaceae; g__Ruminococcaceae_UCG-014; s__uncultured_bacterium_g_Ruminococcaceae_UCG-014 |
| OTU312 | + | + | + | k__Bacteria; p__Firmicutes; c__Clostridia; o__Clostridiales; f__Ruminococcaceae; g__Anaerotruncus; s__uncultured_bacterium_g_Anaerotruncus |
| OTU163 | + | + | + | k__Bacteria; p__Firmicutes; c__Clostridia; o__Clostridiales; f__Lachnospiraceae; g__uncultured_bacterium_f_Lachnospiraceae; s__uncultured_bacterium_f_Lachnospiraceae |
| OTU242 | + | + | + | k__Bacteria; p__Tenericutes; c__Mollicutes; o__Mollicutes_RF39; f__uncultured_bacterium_o_Mollicutes_RF39; g__uncultured_bacterium_o_Mollicutes_RF39; s__uncultured_bacterium_o_Mollicutes_RF39 |
| OTU288 | + | + | + | k__Bacteria; p__Firmicutes; c__Clostridia; o__Clostridiales; f__Lachnospiraceae; g__uncultured_bacterium_f_Lachnospiraceae; s__uncultured_bacterium_f_Lachnospiraceae |
| OTU239 | + | + | + | k__Bacteria; p__Firmicutes; c__Bacilli; o__Lactobacillales; f__Enterococcaceae; g__Enterococcus; s__Enterococcus_faecalis |
| OTU290 | + | + | + | k__Bacteria; p__Firmicutes; c__Clostridia; o__Clostridiales; f__Lachnospiraceae; g__Lachnospiraceae_FCS020_group; s__uncultured_bacterium_g_Lachnospiraceae_FCS020_group |
| OTU243 | - | + | + | k__Bacteria; p__Firmicutes; c__Clostridia; o__Clostridiales; f__Lachnospiraceae; g__uncultured_bacterium_f_Lachnospiraceae; s__uncultured_bacterium_f_Lachnospiraceae |
| OTU255 | - | + | + | k__Bacteria; p__Firmicutes; c__Clostridia; o__Clostridiales; f__Lachnospiraceae; g__Lachnospiraceae_NK4A136_group; s__Clostridiales_bacterium_CIEAF_020 |
| OTU222 | + | + | + | k__Bacteria; p__Firmicutes; c__Clostridia; o__Clostridiales; f__Ruminococcaceae; g__Ruminococcaceae_UCG-010; s__uncultured_bacterium_g_Ruminococcaceae_UCG-010 |
| OTU315 | + | + | + | k__Bacteria; p__Firmicutes; c__Clostridia; o__Clostridiales; f__Lachnospiraceae; g__uncultured_bacterium_f_Lachnospiraceae; s__uncultured_bacterium_f_Lachnospiraceae |
| OTU262 | - | + | + | k__Bacteria; p__Firmicutes; c__Clostridia; o__Clostridiales; f__Caloramatoraceae; g__Clostridium; s__Clostridium_innocuum |
| OTU106 | + | - | + | k__Bacteria; p__Firmicutes; c__Clostridia; o__Clostridiales; f__Ruminococcaceae; g__Ruminiclostridium_6; s__uncultured_bacterium_g_Ruminiclostridium_6 |
| OTU311 | + | - | + | k__Bacteria; p__Firmicutes; c__Bacilli; o__Lactobacillales; f__Enterococcaceae; g__Enterococcus; s__Enterococcus_mundtii |
| OTU5 | + | + | + | k__Bacteria; p__Bacteroidetes; c__Bacteroidia; o__Bacteroidales; f__Muribaculaceae; g__uncultured_bacterium_f_Muribaculaceae; s__uncultured_bacterium_f_Muribaculaceae |
| OTU115 | + | + | + | k__Bacteria; p__Firmicutes; c__Clostridia; o__Clostridiales; f__Ruminococcaceae; g__Ruminococcaceae_UCG-010; s__uncultured_bacterium_g_Ruminococcaceae_UCG-010 |
| OTU44 | + | + | + | k__Bacteria; p__Bacteroidetes; c__Bacteroidia; o__Bacteroidales; f__Muribaculaceae; g__uncultured_bacterium_f_Muribaculaceae; s__uncultured_bacterium_f_Muribaculaceae |
| OTU205 | - | + | + | k__Bacteria; p__Firmicutes; c__Erysipelotrichia; o__Erysipelotrichales; f__Erysipelotrichaceae; g__uncultured_bacterium_f_Erysipelotrichaceae; s__uncultured_bacterium_f_Erysipelotrichaceae |
| OTU259 | + | + | + | k__Bacteria; p__Firmicutes; c__Clostridia; o__Clostridiales; f__Lachnospiraceae; g__uncultured_bacterium_f_Lachnospiraceae; s__uncultured_bacterium_f_Lachnospiraceae |
| OTU307 | + | + | + | k__Bacteria; p__Firmicutes; c__Clostridia; o__Clostridiales; f__Ruminococcaceae; g__Ruminococcaceae_UCG-014; s__uncultured_bacterium_g_Ruminococcaceae_UCG-014 |
| OTU159 | + | + | + | k__Bacteria; p__Tenericutes; c__Mollicutes; o__Mollicutes_RF39; f__uncultured_bacterium_o_Mollicutes_RF39; g__uncultured_bacterium_o_Mollicutes_RF39; s__uncultured_bacterium_o_Mollicutes_RF39 |
| OTU280 | + | + | + | k__Bacteria; p__Firmicutes; c__Clostridia; o__Clostridiales; f__Ruminococcaceae; g__Ruminiclostridium_5; s__uncultured_bacterium_g_Ruminiclostridium_5 |
| OTU194 | + | + | + | k__Bacteria; p__Proteobacteria; c__Alphaproteobacteria; o__Rhodospirillales; f__uncultured_bacterium_o_Rhodospirillales; g__uncultured_bacterium_o_Rhodospirillales; s__uncultured_bacterium_o_Rhodospirillales |
| OTU287 | + | + | + | k__Bacteria; p__Actinobacteria; c__Coriobacteriia; o__Coriobacteriales; f__Eggerthellaceae; g__Enterorhabdus; s__uncultured_bacterium_g_Enterorhabdus |
| OTU208 | + | + | + | k__Bacteria; p__Firmicutes; c__Clostridia; o__Clostridiales; f__Lachnospiraceae; g__uncultured_bacterium_f_Lachnospiraceae; s__uncultured_bacterium_f_Lachnospiraceae |
| OTU254 | + | + | + | k__Bacteria; p__Firmicutes; c__Clostridia; o__Clostridiales; f__Lachnospiraceae; g__Marvinbryantia; s__uncultured_bacterium_g_Marvinbryantia |
| OTU334 | + | + | + | k__Bacteria; p__Firmicutes; c__Clostridia; o__Clostridiales; f__Ruminococcaceae; g__Ruminococcaceae_UCG-014; s__uncultured_bacterium_g_Ruminococcaceae_UCG-014 |
| OTU206 | + | + | + | k__Bacteria; p__Firmicutes; c__Clostridia; o__Clostridiales; f__Family_XIII; g__Family_XIII_AD3011_group; s__uncultured_bacterium_g_Family_XIII_AD3011_group |
| OTU201 | + | + | + | k__Bacteria; p__Firmicutes; c__Clostridia; o__Clostridiales; f__Ruminococcaceae; g__Ruminiclostridium_1; s__uncultured_bacterium_g_Ruminiclostridium_1 |
| OTU303 | + | + | + | k__Bacteria; p__Firmicutes; c__Clostridia; o__Clostridiales; f__uncultured_bacterium_o_Clostridiales; g__uncultured_bacterium_o_Clostridiales; s__uncultured_bacterium_o_Clostridiales |
| OTU249 | + | + | + | k__Bacteria; p__Firmicutes; c__Clostridia; o__Clostridiales; f__Eubacteriaceae; g__Anaerofustis; s__uncultured_bacterium_g_Anaerofustis |
| OTU321 | + | + | + | k__Bacteria; p__Firmicutes; c__Clostridia; o__Clostridiales; f__Lachnospiraceae; g__Lachnospiraceae_NK4A136_group; s__Lachnospiraceae_bacterium_COE1 |
| OTU180 | - | + | - | k__Bacteria; p__Firmicutes; c__Clostridia; o__Clostridiales; f__Lachnospiraceae; g__uncultured_bacterium_f_Lachnospiraceae; s__uncultured_bacterium_f_Lachnospiraceae |
| OTU67 | + | + | - | k__Bacteria; p__Firmicutes; c__Clostridia; o__Clostridiales; f__Ruminococcaceae; g__uncultured_bacterium_f_Ruminococcaceae; s__uncultured_bacterium_f_Ruminococcaceae |
| OTU179 | - | + | - | k__Bacteria; p__Firmicutes; c__Clostridia; o__Clostridiales; f__Ruminococcaceae; g__Butyricicoccus; s__uncultured_bacterium_g_Butyricicoccus |
| OTU245 | - | + | - | k__Bacteria; p__Thermotogae; c__Thermotogae; o__Kosmotogales; f__Kosmotogaceae; g__Mesotoga; s__Mesotoga_infera |
| OTU182 | + | + | - | k__Bacteria; p__Firmicutes; c__Bacilli; o__Lactobacillales; f__Lactobacillaceae; g__Lactobacillus; s__Lactobacillus_intestinalis |
| OTU203 | + | + | - | k__Bacteria; p__Firmicutes; c__Clostridia; o__Clostridiales; f__Ruminococcaceae; g__uncultured_bacterium_f_Ruminococcaceae; s__uncultured_bacterium_f_Ruminococcaceae |
| OTU298 | + | + | - | k__Bacteria; p__Firmicutes; c__Clostridia; o__Clostridiales; f__Lachnospiraceae; g__uncultured_bacterium_f_Lachnospiraceae; s__uncultured_bacterium_f_Lachnospiraceae |
| OTU268 | - | + | - | k__Bacteria; p__Firmicutes; c__Clostridia; o__Clostridiales; f__Lachnospiraceae; g__uncultured_bacterium_f_Lachnospiraceae; s__uncultured_bacterium_f_Lachnospiraceae |
| OTU264 | - | + | - | k__Bacteria; p__Firmicutes; c__Clostridia; o__Clostridiales; f__Lachnospiraceae; g__uncultured_bacterium_f_Lachnospiraceae; s__uncultured_bacterium_f_Lachnospiraceae |
| OTU130 | + | + | - | k__Bacteria; p__Firmicutes; c__Clostridia; o__Clostridiales; f__Family_XIII; g__[Eubacterium]_brachy_group; s__uncultured_bacterium_g_[Eubacterium]_brachy_group |
| OTU113 | + | + | - | k__Bacteria; p__Firmicutes; c__Erysipelotrichia; o__Erysipelotrichales; f__Erysipelotrichaceae; g__uncultured_bacterium_f_Erysipelotrichaceae; s__uncultured_bacterium_f_Erysipelotrichaceae |
| OTU157 | - | + | - | k__Bacteria; p__Firmicutes; c__Clostridia; o__Clostridiales; f__Ruminococcaceae; g__[Eubacterium]_coprostanoligenes_group; s__uncultured_bacterium_g_[Eubacterium]_coprostanoligenes_group |
| OTU84 | + | + | - | k__Bacteria; p__Firmicutes; c__Clostridia; o__Clostridiales; f__Ruminococcaceae; g__Ruminococcaceae_UCG-014; s__uncultured_bacterium_g_Ruminococcaceae_UCG-014 |
| OTU209 | - | + | - | k__Bacteria; p__Firmicutes; c__Clostridia; o__Clostridiales; f__Clostridiales_vadinBB60_group; g__uncultured_bacterium_f_Clostridiales_vadinBB60_group; s__uncultured_bacterium_f_Clostridiales_vadinBB60_group |
| OTU164 | - | + | - | k__Bacteria; p__Cyanobacteria; c__Melainabacteria; o__Gastranaerophilales; f__uncultured_bacterium_o_Gastranaerophilales; g__uncultured_bacterium_o_Gastranaerophilales; s__uncultured_bacterium_o_Gastranaerophilales |
| OTU349 | - | + | - | k__Bacteria; p__Firmicutes; c__Clostridia; o__Clostridiales; f__Ruminococcaceae; g__Oscillibacter; s__Oscillibacter_sp |
| OTU237 | + | + | - | k__Bacteria; p__Tenericutes; c__Mollicutes; o__Mollicutes_RF39; f__uncultured_bacterium_o_Mollicutes_RF39; g__uncultured_bacterium_o_Mollicutes_RF39; s__uncultured_bacterium_o_Mollicutes_RF39 |
| OTU343 | - | + | - | k__Bacteria; p__Firmicutes; c__Clostridia; o__Clostridiales; f__Lachnospiraceae; g__uncultured_bacterium_f_Lachnospiraceae; s__uncultured_bacterium_f_Lachnospiraceae |
| OTU49 | + | + | - | k__Bacteria; p__Firmicutes; c__Erysipelotrichia; o__Erysipelotrichales; f__Erysipelotrichaceae; g__Dubosiella; s__uncultured_bacterium_g_Dubosiella |
| OTU227 | + | + | - | k__Bacteria; p__Firmicutes; c__Clostridia; o__Clostridiales; f__Lachnospiraceae; g__Lachnospiraceae_NK4A136_group; s__Clostridiales_bacterium_CIEAF_020 |
| OTU281 | + | + | - | k__Bacteria; p__Firmicutes; c__Clostridia; o__Clostridiales; f__Ruminococcaceae; g__Candidatus_Soleaferrea; s__uncultured_bacterium_g_Candidatus_Soleaferrea |
| OTU296 | - | + | - | k__Bacteria; p__Firmicutes; c__Clostridia; o__Clostridiales; f__Lachnospiraceae; g__uncultured_bacterium_f_Lachnospiraceae; s__uncultured_bacterium_f_Lachnospiraceae |
| OTU308 | - | + | - | k__Bacteria; p__Firmicutes; c__Clostridia; o__Clostridiales; f__Lachnospiraceae; g__uncultured_bacterium_f_Lachnospiraceae; s__uncultured_bacterium_f_Lachnospiraceae |
| OTU246 | + | + | - | k__Bacteria; p__Firmicutes; c__Clostridia; o__Clostridiales; f__Lachnospiraceae; g__[Ruminococcus]_torques_group; s__uncultured_bacterium_g_[Ruminococcus]_torques_group |
| OTU348 | + | + | - | k__Bacteria; p__Firmicutes; c__Clostridia; o__Clostridiales; f__Ruminococcaceae; g__Ruminiclostridium; s__uncultured_bacterium_g_Ruminiclostridium |
| OTU347 | + | + | - | k__Bacteria; p__Firmicutes; c__Clostridia; o__Clostridiales; f__Lachnospiraceae; g__uncultured_bacterium_f_Lachnospiraceae; s__uncultured_bacterium_f_Lachnospiraceae |
| OTU273 | - | + | - | k__Bacteria; p__Bacteroidetes; c__Bacteroidia; o__Bacteroidales; f__Bacteroidaceae; g__Bacteroides; s__Bacteroides_uniformis |
| OTU234 | + | + | - | k__Bacteria; p__Firmicutes; c__Clostridia; o__Clostridiales; f__Lachnospiraceae; g__[Eubacterium]_xylanophilum_group; s__uncultured_bacterium_g_[Eubacterium]_xylanophilum_group |
| OTU196 | + | + | - | k__Bacteria; p__Actinobacteria; c__Coriobacteriia; o__Coriobacteriales; f__Eggerthellaceae; g__Gordonibacter; s__uncultured_bacterium_g_Gordonibacter |
| OTU271 | + | + | - | k__Bacteria; p__Firmicutes; c__Erysipelotrichia; o__Erysipelotrichales; f__Erysipelotrichaceae; g__Candidatus_Stoquefichus; s__uncultured_bacterium_g_Candidatus_Stoquefichus |
| OTU114 | + | + | - | k__Bacteria; p__Firmicutes; c__Erysipelotrichia; o__Erysipelotrichales; f__Erysipelotrichaceae; g__uncultured_bacterium_f_Erysipelotrichaceae; s__uncultured_bacterium_f_Erysipelotrichaceae |
| OTU294 | - | + | - | k__Bacteria; p__Firmicutes; c__Clostridia; o__Clostridiales; f__Lachnospiraceae; g__Tyzzerella; s__uncultured_bacterium_g_Tyzzerella |
| OTU324 | - | + | - | k__Bacteria; p__Firmicutes; c__Clostridia; o__Clostridiales; f__Lachnospiraceae; g__GCA-900066575; s__uncultured_bacterium_g_GCA-900066575 |
| OTU327 | + | + | - | k__Bacteria; p__Firmicutes; c__Clostridia; o__Clostridiales; f__Lachnospiraceae; g__[Eubacterium]_xylanophilum_group; s__uncultured_bacterium_g_[Eubacterium]_xylanophilum_group |
| OTU276 | + | + | - | k__Bacteria; p__Firmicutes; c__Clostridia; o__Clostridiales; f__Lachnospiraceae; g__uncultured_bacterium_f_Lachnospiraceae; s__uncultured_bacterium_f_Lachnospiraceae |
| OTU125 | - | + | - | k__Bacteria; p__Firmicutes; c__Clostridia; o__Clostridiales; f__Ruminococcaceae; g__uncultured_bacterium_f_Ruminococcaceae; s__uncultured_bacterium_f_Ruminococcaceae |
| OTU272 | - | + | - | k__Bacteria; p__Firmicutes; c__Clostridia; o__Clostridiales; f__Lachnospiraceae; g__Lachnospiraceae_NK4A136_group; s__uncultured_bacterium_g_Lachnospiraceae_NK4A136_group |
| OTU151 | + | + | - | k__Bacteria; p__Firmicutes; c__Erysipelotrichia; o__Erysipelotrichales; f__Erysipelotrichaceae; g__Faecalibaculum; s__Faecalibaculum_rodentium |
| OTU231 | + | + | - | k__Bacteria; p__Firmicutes; c__Clostridia; o__Clostridiales; f__Lachnospiraceae; g__Lachnospiraceae_NK4A136_group; s__uncultured_bacterium_g_Lachnospiraceae_NK4A136_group |
| OTU229 | + | + | - | k__Bacteria; p__Tenericutes; c__Mollicutes; o__Mollicutes_RF39; f__uncultured_bacterium_o_Mollicutes_RF39; g__uncultured_bacterium_o_Mollicutes_RF39; s__uncultured_bacterium_o_Mollicutes_RF39 |
| OTU295 | - | + | - | k__Bacteria; p__Firmicutes; c__Clostridia; o__Clostridiales; f__Ruminococcaceae; g__Harryflintia; s__uncultured_bacterium_g_Harryflintia |
| OTU301 | - | + | - | k__Bacteria; p__Firmicutes; c__Clostridia; o__Clostridiales; f__Lachnospiraceae; g__uncultured_bacterium_f_Lachnospiraceae; s__uncultured_bacterium_f_Lachnospiraceae |
| OTU100 | + | + | - | k__Bacteria; p__Tenericutes; c__Mollicutes; o__Mollicutes_RF39; f__uncultured_bacterium_o_Mollicutes_RF39; g__uncultured_bacterium_o_Mollicutes_RF39; s__uncultured_bacterium_o_Mollicutes_RF39 |
| OTU267 | + | + | - | k__Bacteria; p__Firmicutes; c__Clostridia; o__Clostridiales; f__Ruminococcaceae; g__uncultured_bacterium_f_Ruminococcaceae; s__uncultured_bacterium_f_Ruminococcaceae |
| OTU165 | - | + | - | k__Bacteria; p__Proteobacteria; c__Gammaproteobacteria; o__Enterobacteriales; f__Enterobacteriaceae; g__Morganella; s__Morganella_morganii |
| OTU269 | - | + | - | k__Bacteria; p__Firmicutes; c__Clostridia; o__Clostridiales; f__Lachnospiraceae; g__Lachnospiraceae_UCG-006; s__uncultured_bacterium_g_Lachnospiraceae_UCG-006 |
| OTU10 | + | - | - | k__Bacteria; p__Firmicutes; c__Clostridia; o__Clostridiales; f__Lachnospiraceae; g__Lachnospiraceae_NK4A136_group; s__uncultured_bacterium_g_Lachnospiraceae_NK4A136_group |
| OTU30 | + | - | - | k__Bacteria; p__Bacteroidetes; c__Bacteroidia; o__Bacteroidales; f__Prevotellaceae; g__Alloprevotella; s__uncultured_bacterium_g_Alloprevotella |
| OTU57 | + | - | - | k__Bacteria; p__Bacteroidetes; c__Bacteroidia; o__Bacteroidales; f__Muribaculaceae; g__uncultured_bacterium_f_Muribaculaceae; s__uncultured_bacterium_f_Muribaculaceae |
| OTU105 | + | - | - | k__Bacteria; p__Firmicutes; c__Clostridia; o__Clostridiales; f__Ruminococcaceae; g__Ruminococcaceae_UCG-014; s__uncultured_bacterium_g_Ruminococcaceae_UCG-014 |
| OTU110 | + | - | - | k__Bacteria; p__Tenericutes; c__Mollicutes; o__Mollicutes_RF39; f__uncultured_bacterium_o_Mollicutes_RF39; g__uncultured_bacterium_o_Mollicutes_RF39; s__uncultured_bacterium_o_Mollicutes_RF39 |
| OTU116 | + | - | - | k__Bacteria; p__Firmicutes; c__Clostridia; o__Clostridiales; f__Ruminococcaceae; g__Ruminococcaceae_UCG-014; s__uncultured_bacterium_g_Ruminococcaceae_UCG-014 |
| OTU148 | + | - | - | k__Bacteria; p__Firmicutes; c__Clostridia; o__Clostridiales; f__Ruminococcaceae; g__uncultured_bacterium_f_Ruminococcaceae; s__uncultured_bacterium_f_Ruminococcaceae |
| OTU215 | + | - | - | k__Bacteria; p__Firmicutes; c__Erysipelotrichia; o__Erysipelotrichales; f__Erysipelotrichaceae; g__uncultured_bacterium_f_Erysipelotrichaceae; s__uncultured_bacterium_f_Erysipelotrichaceae |
| OTU172 | + | - | - | k__Bacteria; p__Firmicutes; c__Clostridia; o__Clostridiales; f__Lachnospiraceae; g__uncultured_bacterium_f_Lachnospiraceae; s__uncultured_bacterium_f_Lachnospiraceae |
| OTU173 | + | - | - | k__Bacteria; p__Tenericutes; c__Mollicutes; o__Mollicutes_RF39; f__uncultured_bacterium_o_Mollicutes_RF39; g__uncultured_bacterium_o_Mollicutes_RF39; s__uncultured_bacterium_o_Mollicutes_RF39 |
| OTU189 | + | - | - | k__Bacteria; p__Firmicutes; c__Clostridia; o__Clostridiales; f__Ruminococcaceae; g__Ruminococcaceae_UCG-014; s__uncultured_bacterium_g_Ruminococcaceae_UCG-014 |
| OTU233 | + | - | - | k__Bacteria; p__Tenericutes; c__Mollicutes; o__Mollicutes_RF39; f__uncultured_bacterium_o_Mollicutes_RF39; g__uncultured_bacterium_o_Mollicutes_RF39; s__uncultured_bacterium_o_Mollicutes_RF39 |
| OTU274 | + | - | - | k__Bacteria; p__Firmicutes; c__Clostridia; o__Clostridiales; f__Lachnospiraceae; g__Lachnoclostridium; s__Clostridium_sp |
| OTU197 | + | - | - | k__Bacteria; p__Tenericutes; c__Mollicutes; o__Mollicutes_RF39; f__uncultured_bacterium_o_Mollicutes_RF39; g__uncultured_bacterium_o_Mollicutes_RF39; s__uncultured_bacterium_o_Mollicutes_RF39 |
| OTU181 | + | - | - | k__Bacteria; p__Firmicutes; c__Clostridia; o__Clostridiales; f__Ruminococcaceae; g__Ruminococcaceae_UCG-014; s__uncultured_bacterium_g_Ruminococcaceae_UCG-014 |
| OTU213 | + | - | - | k__Bacteria; p__Firmicutes; c__Clostridia; o__Clostridiales; f__Ruminococcaceae; g__Ruminococcaceae_UCG-014; s__uncultured_bacterium_g_Ruminococcaceae_UCG-014 |
| OTU252 | + | - | - | k__Bacteria; p__Firmicutes; c__Clostridia; o__Clostridiales; f__Ruminococcaceae; g__[Eubacterium]_coprostanoligenes_group; s__uncultured_bacterium_g_[Eubacterium]_coprostanoligenes_group |
| OTU251 | + | - | - | k__Bacteria; p__Firmicutes; c__Clostridia; o__Clostridiales; f__Ruminococcaceae; g__Ruminococcaceae_UCG-014; s__uncultured_bacterium_g_Ruminococcaceae_UCG-014 |
| OTU174 | + | - | - | k__Bacteria; p__Cyanobacteria; c__Melainabacteria; o__Gastranaerophilales; f__uncultured_bacterium_o_Gastranaerophilales; g__uncultured_bacterium_o_Gastranaerophilales; s__uncultured_bacterium_o_Gastranaerophilales |
| OTU211 | + | - | - | k__Bacteria; p__Firmicutes; c__Clostridia; o__Clostridiales; f__Ruminococcaceae; g__Ruminococcaceae_UCG-014; s__uncultured_bacterium_g_Ruminococcaceae_UCG-014 |
| OTU232 | + | - | - | k__Bacteria; p__Firmicutes; c__Clostridia; o__Clostridiales; f__Ruminococcaceae; g__Ruminococcaceae_UCG-014; s__uncultured_bacterium_g_Ruminococcaceae_UCG-014 |
| OTU277 | + | - | - | k__Bacteria; p__Firmicutes; c__Clostridia; o__Clostridiales; f__Defluviitaleaceae; g__Defluviitaleaceae_UCG-011; s__uncultured_bacterium_g_Defluviitaleaceae_UCG-011 |
| OTU278 | + | - | - | k__Bacteria; p__Firmicutes; c__Clostridia; o__Clostridiales; f__Ruminococcaceae; g__Ruminococcaceae_UCG-014; s__uncultured_bacterium_g_Ruminococcaceae_UCG-014 |
| OTU279 | + | - | - | k__Bacteria; p__Firmicutes; c__Clostridia; o__Clostridiales; f__Ruminococcaceae; g__Ruminococcaceae_UCG-014; s__uncultured_bacterium_g_Ruminococcaceae_UCG-014 |
| OTU214 | + | - | - | k__Bacteria; p__Tenericutes; c__Mollicutes; o__Mollicutes_RF39; f__uncultured_bacterium_o_Mollicutes_RF39; g__uncultured_bacterium_o_Mollicutes_RF39; s__uncultured_bacterium_o_Mollicutes_RF39 |
| OTU275 | + | - | - | k__Bacteria; p__Tenericutes; c__Mollicutes; o__Mollicutes_RF39; f__uncultured_bacterium_o_Mollicutes_RF39; g__uncultured_bacterium_o_Mollicutes_RF39; s__uncultured_bacterium_o_Mollicutes_RF39 |
| OTU332 | + | - | - | k__Bacteria; p__Actinobacteria; c__Coriobacteriia; o__Coriobacteriales; f__Eggerthellaceae; g__uncultured_bacterium_f_Eggerthellaceae; s__uncultured_bacterium_f_Eggerthellaceae |
| OTU230 | + | - | - | k__Bacteria; p__Firmicutes; c__Clostridia; o__Clostridiales; f__Ruminococcaceae; g__Ruminococcaceae_UCG-013; s__uncultured_bacterium_g_Ruminococcaceae_UCG-013 |
| OTU304 | + | - | - | k__Bacteria; p__Firmicutes; c__Clostridia; o__Clostridiales; f__Ruminococcaceae; g__uncultured_bacterium_f_Ruminococcaceae; s__uncultured_bacterium_f_Ruminococcaceae |
| OTU253 | + | - | - | k__Bacteria; p__Tenericutes; c__Mollicutes; o__Mollicutes_RF39; f__uncultured_bacterium_o_Mollicutes_RF39; g__uncultured_bacterium_o_Mollicutes_RF39; s__uncultured_bacterium_o_Mollicutes_RF39 |
| OTU325 | + | - | - | k__Bacteria; p__Cyanobacteria; c__Melainabacteria; o__Gastranaerophilales; f__uncultured_bacterium_o_Gastranaerophilales; g__uncultured_bacterium_o_Gastranaerophilales; s__uncultured_bacterium_o_Gastranaerophilales |
| OTU341 | + | - | - | k__Bacteria; p__Firmicutes; c__Clostridia; o__Clostridiales; f__Christensenellaceae; g__uncultured_bacterium_f_Christensenellaceae; s__uncultured_bacterium_f_Christensenellaceae |
| OTU266 | + | - | - | k__Bacteria; p__Firmicutes; c__Clostridia; o__Clostridiales; f__Ruminococcaceae; g__Ruminococcaceae_UCG-005; s__uncultured_bacterium_g_Ruminococcaceae_UCG-005 |
| OTU248 | + | - | - | k__Bacteria; p__Tenericutes; c__Mollicutes; o__Mollicutes_RF39; f__uncultured_bacterium_o_Mollicutes_RF39; g__uncultured_bacterium_o_Mollicutes_RF39; s__uncultured_bacterium_o_Mollicutes_RF39 |
| OTU195 | + | - | - | k__Bacteria; p__Firmicutes; c__Clostridia; o__Clostridiales; f__Lachnospiraceae; g__uncultured_bacterium_f_Lachnospiraceae; s__uncultured_bacterium_f_Lachnospiraceae |
| OTU300 | + | - | - | k__Bacteria; p__Cyanobacteria; c__Melainabacteria; o__Gastranaerophilales; f__uncultured_bacterium_o_Gastranaerophilales; g__uncultured_bacterium_o_Gastranaerophilales; s__uncultured_bacterium_o_Gastranaerophilales |
