# Supplementary material for: Angelica polysaccharides relieve blood glucose levels in diabetic KKAy mice possibly by modulating gut microbiota: an integrated gut microbiota and metabolism analysis
Source: BMC Microbiol. 2023 Oct 3;23:281. doi: 10.1186/s12866-023-03029-y (PMC10546737; doi:10.1186/s12866-023-03029-y)
Supplement: Supplementary file 5 — Additional file 5: Supplementary Table 2. The detail bacteria species distribution in CT, HFD, and AP groups. [file 12866_2023_3029_MOESM5_ESM.pdf]

Supplementary Table 2. The detail bacteria species distribution in CT, HFD, and AP groups.

ID	CT	HFD	AP
Lachnospiraceae_bacterium_A4	+	+	+
bacterium_1xD42-87	+	+	+
Oscillibacter_sp._1-3	+	+	+
Dorea_sp._5-2	+	+	+
Lachnospiraceae_bacterium_3-2	+	+	+
bacterium_1XD8-76	+	+	+
Mucispirillum_schaedleri	+	+	+
Lactobacillus_murinus	+	+	+
Firmicutes_bacterium_ASF500	+	+	+
bacterium_1XD8-92	+	+	+
Prevotella_sp._MGM2	+	+	+
Chlamydia_abortus	+	+	+
Lachnospiraceae_bacterium_M18-1	+	+	+
bacterium_1xD42-67	+	+	+
bacterium_1XD21-70	+	+	+
Acetatifactor_muris	+	+	+
Bacteroides_sartorii	+	+	+
Clostridium_sp._ASF502	+	+	+
bacterium_0.1xD8-71	+	+	+
Lachnospiraceae_bacterium_COE1	+	+	+
Ruminococcus_sp._1xD21-23	+	+	+
Lachnospiraceae_bacterium_A2	+	+	+
bacterium_J10(2018)	+	+	+
Prevotella_sp._MGM1	+	+	+
bacterium_0.1xD8-82	+	+	+
Desulfovibrionaceae_bacterium	+	+	+
Roseburia_sp._1XD42-69	+	+	+
Lachnospiraceae_bacterium_3-1	+	+	+
Lachnoclostridium_sp._An196	+	+	+
Clostridiaceae_bacterium_DSM_106076	+	+	+
Eubacterium_plexicaudatum	+	+	+
Muribaculaceae_bacterium_Isolate-080_(Janvier)	+	+	+
Lachnospiraceae_bacterium_10-1	+	+	+
Eubacterium_sp._14-2	+	+	+
Muribaculaceae_bacterium_Isolate-104_(HZI)	+	+	+
Helicobacter_bilis	+	+	+
bacterium_D16-50	+	+	+
Hungatella_hathewayi	+	+	+
Odoribacter_splanchnicus	+	+	+
Anaerotruncus_sp._1XD22-93	+	+	+
Acutalibacter_muris	+	+	+
Bacteroides_uniformis	+	+	+
uncultured_Clostridium_sp.	+	+	+
Acutalibacter_sp._1XD8-33	+	+	+
Muribaculaceae_bacterium_Isolate-110_(HZI)	+	+	+
Muribaculaceae_bacterium_Isolate-013_(NCI)	+	+	+
Butyricicoccus_sp._1XD8-22	+	+	+
Muribaculaceae_bacterium_Isolate-102_(HZI)	+	+	+
Alistipes_sp._CHKCI003	+	+	+
Muribaculaceae_bacterium_Isolate-037_(Harlan)	+	+	+
[Clostridium]_clostridioforme	+	+	+
Clostridiaceae_bacterium_OM08-6BH	+	+	+
Alistipes_senegalensis	+	+	+

bacterium_C-53	+	+	+
Clostridiales_bacterium	+	+	+
Bacteroides_caecimuris	+	+	+
Anaerotruncus_sp._G3(2012)	+	+	+
Lachnospiraceae_bacterium_AM48-27BH	+	+	+
Clostridium_sp._chh4-2	+	+	+
Lachnospiraceae_bacterium_28-4	+	+	+
Roseburia_sp._499	+	+	+
Duncaniella_muris	+	+	+
Oscillibacter_sp._PC13	+	+	+
Bacteroides_acidifaciens	+	+	+
Alistipes_sp._CAG:268	+	+	+
Alistipes_timonensis	+	+	+
Faecalibacterium_prausnitzii	+	+	+
Pseudoflavonifractor_sp._Marseille-P3106	+	+	+
Flavobacteriales_bacterium	+	+	+
Muribaculum_intestinale	+	+	+
Lachnoclostridium_sp._An138	+	+	+
Oscillibacter_sp._CAG:155	+	+	+
uncultured_prokaryote	+	+	+
Bacteroides_vulgatus	+	+	+
Angelakisella_massiliensis	+	+	+
Paramuribaculum_intestinale	+	+	+
Bacteroidales_bacterium_WCE2008	+	+	+
Lachnoclostridium_sp._An131	+	+	+
Alistipes_finegoldii	+	+	+
Mailhella_massiliensis	+	+	+
Prevotella_sp._CAG:873	+	+	+
Rikenella_microfusus	+	+	+
Firmicutes_bacterium	+	+	+
Flavonifractor_plautii	+	+	+
Bilophila_wadsworthia	+	+	+
Alistipes_ihumii	+	+	+
[Ruminococcus]_gnavus	+	+	+
Alistipes_sp._An66	+	+	+
Bacteroides_sp._CAG:709	+	+	+
Roseburia_inulinivorans	+	+	+
[Clostridium]_scindens	+	+	+
[Eubacterium]_rectale	+	+	+
Bacteroidales_bacterium	+	+	+
Bacteroides_sp._CAG:770	+	+	+
Odoribacter_laneus	+	+	+
bacterium_1XD42-54	+	+	+
Muribaculaceae_bacterium_Isolate-002_(NCI)	+	+	+
Lactobacillus_reuteri	+	+	+
Muribaculaceae_bacterium_Isolate-039_(Harlan)	+	+	+
Bacteroides_fragilis	+	+	+
Bacteroides_sp._CAG:927	+	+	+
bacterium_1XD42-8	+	+	+
Bacteroides_sp._CAG:545	+	+	+
Marvinbryantia_formatexigens	+	+	+
Alistipes_sp._CAG:435	+	+	+
Anaerotruncus_colihominis	+	+	+
Alistipes_sp._Marseille-P2431	+	+	+
Clostridioides_difficile	+	+	+

Blautia_schinkii	+	+	+
Blautia_obeum	+	+	+
Alistipes_onderdonkii	+	+	+
[Clostridium]_aldenense	+	+	+
Roseburia_intestinalis	+	+	+
Corallococcus_sp._CAG:1435	+	+	+
[Clostridium]_bolteae	+	+	+
Blautia_producta	+	+	+
Lachnoclostridium_sp._An14	+	+	+
Alistipes_putredinis	+	+	+
[Clostridium]_symbiosum	+	+	+
Dorea_formicigenerans	+	+	+
[Ruminococcus]_torques	+	+	+
Parabacteroides_distasonis	+	+	+
Intestinimonas_butyriciproducens	+	+	+
Muribaculaceae_bacterium_Isolate-105_(HZI)	+	+	+
Acetivibrio_ethanolgignens	+	+	+
Culturomica_massiliensis	+	+	+
Alistipes_sp._CAG:514	+	+	+
Clostridium_sp._CAG:58	+	+	+
uncultured_Flavonifractor_sp.	+	+	+
Intestinimonas_timonensis	+	+	+
Dorea_longicatena	+	+	+
Clostridium_sp._Marseille-P8228	+	+	+
[Clostridium]_glycyrrhizinilyticum	+	+	+
Bacteroidales_bacterium_52_46	+	+	+
Kineothrix_alysoides	+	+	+
Eisenbergiella_massiliensis	+	+	+
Candidatus_Borkfalki_ceftriaxensis	+	+	+
Ruminococcus_flavefaciens	+	+	+
Lachnotalea_sp._AF33-28	+	+	+
Lachnospiraceae_bacterium	+	+	+
Bacteroides_intestinalis	+	+	+
Muribaculaceae_bacterium_Isolate-004_(NCI)	+	+	+
Firmicutes_bacterium_CAG:534	+	+	+
Roseburia_sp._CAG:100	+	+	+
Muribaculaceae_bacterium_Isolate-036_(Harlan)	+	+	+
Roseburia_sp._CAG:10041_57	+	+	+
Lachnospiraceae_bacterium_7_1_58FAA	+	+	+
Alistipes_shahii	+	+	+
Bacteroides_sp._OF04-15BH	+	+	+
Blautia_coccoides	+	+	+
uncultured_bacterium	+	+	+
uncultured_Ruminococcus_sp.	+	+	+
Desulfovibrio_piger	+	+	+
Clostridium_sp._AM58-1XD	+	+	+
Roseburia_sp._40_7	+	+	+
[Clostridium]_citroniae	+	+	+
Prevotella_sp._CAG:1031	+	+	+
Bacteroides_thetaiotaomicron	+	+	+
Candidatus_Saccharibacteria_bacterium_TM7_G3_2_Rum_HO	+	+	+
Neglecta_timonensis	+	+	+
Muribaculaceae_bacterium_Isolate-001_(NCI)	+	+	+
Pseudoflavonifractor_sp._AF19-9AC	+	+	+
Muribaculaceae_bacterium_Isolate-042_(Harlan)	+	+	+

Alistipes_obesi	+	+	+
Parabacteroides_merdae	+	+	+
Chlamydia_trachomatis	+	+	+
Lactonifactor_longoviformis	+	+	+
Bilophila_sp._4_1_30	+	+	+
Clostridium_sp._1xD42-85	+	+	+
uncultured_Eubacterium_sp.	+	+	+
Intestinimonas_massiliensis	+	+	+
Clostridium_sp._AT4	+	+	+
[Clostridium]_lavalense	+	+	+
uncultured_Blautia_sp.	+	+	+
Blautia_wexlerae	+	+	+
Desulfovibrio_fairfieldensis	+	+	+
Alistipes_sp._HGB5	+	+	+
Eisenbergiella_tayi	+	+	+
Bacteroides_rodentium	+	+	+
Parabacteroides_johnsonii	+	+	+
[Clostridium]_asparagiforme	+	+	+
Lachnoclostridium_sp._An169	+	+	+
Paraprevotella_clara	+	+	+
Hespellia_stercorisuis	+	+	+
Alistipes_sp._Marseille-P5997	+	+	+
Butyricicoccus_pullicaecorum	+	+	+
Clostridium_sp._ASF356	+	+	+
Roseburia_hominis	+	+	+
Oscillibacter_valericigenes	+	+	+
Alistipes_sp._An31A	+	+	+
Flavonifractor_sp._An100	+	+	+
Clostridiales_bacterium_Marseille-P5551	+	+	+
Anaeroplasma_bactoclasticum	+	+	+
Lactobacillus_animalis	+	+	+
Butyrivibrio_fibrisolvens	+	+	+
Odoribacter_sp._OF09-27XD	+	+	+
Hungatella_effluvii	+	+	+
Oscillibacter_sp._57_20	+	+	+
Clostridia_bacterium	+	+	+
Lachnoclostridium_edouardi	+	+	+
Lawsonibacter_asaccharolyticus	+	+	+
Bacteroides_ovatus	+	+	+
Flavonifractor_sp._An306	+	+	+
Cuneatibacter_caecimuris	+	+	+
Desulfovibrio_sp._3_1_syn3	+	+	+
Bacteroidales_bacterium_55_9	+	+	+
Ruminococcaceae_bacterium	+	+	+
[Clostridium]_innocuum	+	+	+
Eisenbergiella_sp._OF01-20	+	+	+
Clostridiales_bacterium_CHKCI001	+	+	+
Klebsiella_oxytoca	+	+	+
Candidatus_Amulumruptor_caecigallinarius	+	+	+
Lachnotalea_glycerini	+	+	+
Blautia_hydrogenotrophica	+	+	+
Ruminococcus_gauvreauii	+	+	+
Millionella_massiliensis	+	+	+
Enterorhabdus_caecimuris	+	+	+
Lachnospiraceae_bacterium_OM04-12BH	+	+	+

Bacteroides_dorei	+	+	+
Alistipes_sp._AF14-19	+	+	+
Clostridiales_bacterium_CCNA10	+	+	+
Bacteroides_stercoris	+	+	+
Roseburia_sp._CAG:309	+	+	+
Blautia_sp._An249	+	+	+
Ruminococcaceae_bacterium_AM07-15	+	+	+
Prevotella_sp._CAG:485	+	+	+
[Eubacterium]_eligens	+	+	+
Pseudoflavonifractor_sp._An184	+	+	+
Paraprevotella_xylaniphila	+	+	+
Lachnospiraceae_bacterium_1_4_56FAA	+	+	+
Oscillibacter_ruminantium	+	+	+
Bacteroides_fluxus	+	+	+
Firmicutes_bacterium_CAG:552_39_19	+	+	+
Bacteroides_plebeius	+	+	+
Bacteroidales_bacterium_WCE2004	+	+	+
Anaeromassilibacillus_sp._An200	+	+	+
Lactobacillus_johnsonii	+	+	+
Parabacteroides_goldsteinii	+	+	+
Enterococcus_faecium	+	+	+
[Clostridium]_methoxybenzovorans	+	+	+
Coprococcus_comes	+	+	+
Selenomonas_ruminantium	+	+	+
Blautia_sp._An81	+	+	+
Bacteroides_sp._CAG:1060_57_27	+	+	+
Intestinibacillus_sp._Marseille-P6563	+	+	+
[Eubacterium]_hallii	+	+	+
Clostridium_sp._Marseille-P2415	+	+	+
Pseudoflavonifractor_capillosus	+	+	+
Clostridium_sp._KLE_1755	+	+	+
Clostridium_sp._SN20	+	+	+
Ruminococcus_albus	+	+	+
Clostridiales_bacterium_1_7_47FAA	+	+	+
Ruminococcaceae_bacterium_D16	+	+	+
Alistipes_sp._58_9_plus	+	+	+
Desulfovibrio_sp._6_1_46AFAA	+	+	+
Bariatricus_massiliensis	+	+	+
Clostridiales_bacterium_45_37	+	+	+
Faecalicatena_contorta	+	+	+
Siphoviridae_sp.	+	+	+
Anaerocolumna_aminovalerica	+	+	+
Dorea_sp._CAG:317	+	+	+
Alistipes_sp._An54	+	+	+
Alistipes_indistinctus	+	+	+
Odoribacter_laneus_CAG:561	+	+	+
Negativibacillus_massiliensis	+	+	+
Butyricimonas_virosa	+	+	+
Clostridium_sp._PI-S10-A1B	+	+	+
[Clostridium]_methylpentosum	+	+	+
Faecalibacterium_sp._CAG:1138	+	+	+
Clostridium_sp._AF27-2AA	+	+	+
Firmicutes_bacterium_CAG:552	+	+	+
Coprococcus_catus	+	+	+
Clostridiales_bacterium_VE202-15	+	+	+

Oscillibacter_sp._PEA192	+	+	+
Clostridiales_bacterium_Nov_37_41	+	+	+
Bacteroides_sp._CAG:1060	+	+	+
Firmicutes_bacterium_CAG:95	+	+	+
Anaerosporobacter_mobilis	+	+	+
Clostridium_sp._AF19-22AC	+	+	+
Flavonifractor_sp._An10	+	+	+
Firmicutes_bacterium_CAG:124	+	+	+
Peptococcaceae_bacterium_DCMF	+	+	+
Alistipes_sp._56_sp_Nov_56_25	+	+	+
Clostridium_sp._AF18-27	+	+	+
Prevotella_sp._P3-122	+	+	+
uncultured_Clostridiales_bacterium	+	+	+
Alistipes_sp._CAG:831	+	+	+
Anaerotignum_lactatifermentans	+	+	+
Alistipes_sp._AM16-43	+	+	+
Bacilliculturomica_massiliensis	+	+	+
Clostridiales_bacterium_52_15	+	+	+
Butyrivibrio_proteoclasticus	+	+	+
Anaerocolumna_jejuensis	+	+	+
Muribaculaceae_bacterium_Isolate-043_(Harlan)	+	+	+
Clostridium_sp._C105KSO14	+	+	+
Mordavella_sp._Marseille-P3756	+	+	+
Merdimonas_faecis	+	+	+
Oscillibacter_sp._KLE_1728	+	+	+
Clostridium_sp._CAG:349	+	+	+
Anaerobium_acetethylicum	+	+	+
Roseburia_sp._831b	+	+	+
Roseburia_faecis	+	+	+
Clostridium_sp._CAG:1013	+	+	+
Odoribacter_sp._CAG:788	+	+	+
Bacteroides_xylanisolvens	+	+	+
Lachnoclostridium_sp._An76	+	+	+
Lachnoclostridium_sp._An298	+	+	+
Eubacteriaceae_bacterium_CHKCI004	+	+	+
Roseburia_sp._CAG:182	+	+	+
bacterium_1XD42-1	+	+	+
Firmicutes_bacterium_CAG:129	+	+	+
Clostridiales_bacterium_59_14	+	+	+
[Clostridium]_hylemonae	+	+	+
Flavonifractor_sp._An82	+	+	+
Parabacteroides_sp._CH2-D42-20	+	+	+
Bacteroides_cellulosilyticus	+	+	+
Bacteroides_xylanolyticus	+	+	+
Blautia_marasmi	+	+	+
Eubacterium_sp._An11	+	+	+
Clostridium_botulinum	+	+	+
Cloacibacillus_sp._An23	+	+	+
Clostridiales_bacterium_42_27	+	+	+
Anaerostipes_sp._992a	+	+	+
Bacteroides_faecichinchillae	+	+	+
Desulfovibrio_desulfuricans	+	+	+
Lawsonia_intracellularis	+	+	+
Prevotella_sp._CAG:279	+	+	+
Clostridium_sp._Marseille-P3244	+	+	+

[Clostridium]_amygdalinum	+	+	+
Firmicutes_bacterium_CAG:882	+	+	+
Clostridiales_bacterium_41_12_two_minus	+	+	+
[Clostridium]_leptum	+	+	+
[Clostridium]_saccharolyticum	+	+	+
Faecalicatena_orotica	+	+	+
Clostridiales_bacterium_VE202-28	+	+	+
Clostridium_sp._C105KSO13	+	+	+
Muribaculaceae_bacterium_Isolate-007_(NCI)	+	+	+
Escherichia_coli	+	+	+
Acidaminococcus_sp._CAG:917	+	+	+
Bacteroidetes_bacterium	+	+	+
[Clostridium]_fimetarium	+	+	+
Clostridium_sp._CAG:510	+	+	+
Bacteroidales_bacterium_43_36	+	+	+
uncultured_Roseburia_sp.	+	+	+
Lachnospiraceae_bacterium_Marseille-P3773	+	+	+
Butyricimonas_synergistica	+	+	+
Clostridium_sp._TF11-13AC	+	+	+
Lachnoclostridium_phytofermentans	+	+	+
Desulfovibrio_legallii	+	+	+
Clostridium_sp._KNHs205	+	+	+
Bacteroides_eggerthii	+	+	+
Ruminococcus_lactaris	+	+	+
uncultured_delta_proteobacterium	+	+	+
Marasmitruncus_massiliensis	+	+	+
Blautia_sp._CAG:257	+	+	+
Clostridium_phoceensis	+	+	+
Enterococcus_faecalis	+	+	+
Bacteroides_caccae	+	+	+
uncultured_Desulfovibrio_sp.	+	+	+
Pseudoflavonifractor_sp._An85	+	+	+
Tyzzerella_nexilis	+	+	+
Blautia_sp._OF03-15BH	+	+	+
Robinsoniella_sp._MCWD5	+	+	+
Butyricimonas_sp._Marseille-P3923	+	+	+
Desulfovibrio_vulgaris	+	+	+
Lactobacillus_ruminis	+	+	+
bacterium_D16-59	+	+	+
Anaerocolumna_xylanovorans	+	+	+
Anaerobacterium_chartisolvens	+	+	+
Lachnoclostridium_sp._An118	+	+	+
Anaerotignum_sp._KCTC_15736	+	+	+
uncultured_Bacteroides_sp.	+	+	+
Roseburia_sp._TF10-5	+	+	+
Subdoligranulum_variabile	+	+	+
Mouse_Intracisternal_A-particle	+	+	+
Firmicutes_bacterium_CAG:475	+	+	+
Firmicutes_bacterium_CAG:137	+	+	+
Ruminococcus_bromii	+	+	+
Sarcina_sp._DSM_11001	+	+	+
[Eubacterium]_siraeum	+	+	+
Blautia_hominis	+	+	+
Firmicutes_bacterium_CAG:646	+	+	+
Fusicatenibacter_saccharivorans	+	+	+

Clostridium_beijerinckii	+	+	+
Drancourtella_sp._An57	+	+	+
Dorea_sp._D27	+	+	+
Alistipes_sp._Marseille-P5061	+	+	+
Alistipes_putredinis_CAG:67	+	+	+
[Clostridium]_celerecrescens	+	+	+
Butyrivibrio_sp._INlla16	+	+	+
Alistipes_sp._AL-1	+	+	+
Lachnospiraceae_bacterium_C10	+	+	+
Prevotella_copri	+	+	+
Bacteroides_sp._D20	+	+	+
Emergencia_timonensis	+	+	+
Bacteroides_salyersiae	+	+	+
Roseburia_sp._CAG:303	+	+	+
Firmicutes_bacterium_CAG:94	+	+	+
Bacteroides_coprocola	+	+	+
Prevotella_sp._CAG:891	+	+	+
Clostridium_butyricum	+	+	+
Desulfovibrio_litoralis	+	+	+
Candidatus_Saccharibacteria_bacterium_TM7_KMM_G3_1_H(	+	+	+
Dehalobacterium_formicoaceticum	+	+	+
Lachnospiraceae_bacterium_TF09-5	+	+	+
Ruminococcaceae_bacterium_Marseille-P2935	+	+	+
Firmicutes_bacterium_CAG:114	+	+	+
Robinsoniella_sp._KNHs210	+	+	+
Alistipes_sp._CAG:29	+	+	+
Gemmiger_sp._An120	+	+	+
Desulfovibrio_cuneatus	+	+	+
Bacteroides_finegoldii	+	+	+
Blautia_sp._N6H1-15	+	+	+
Lachnospiraceae_bacterium_OF09-33XD	+	+	+
Anaerostipes_hadrus	+	+	+
Coprococcus_eutactus	+	+	+
Clostridiales_bacterium_VE202-26	+	+	+
Clostridium_sp._Marseille-P299	+	+	+
Anaeromassilibacillus_sp._An250	+	+	+
Hungateiclostridiaceae_bacterium_KB18	+	+	+
Butyrivibrio_sp._YAB3001	+	+	+
Drancourtella_sp._An12	+	+	+
Butyrivibrio_hungatei	+	+	+
Lachnospiraceae_bacterium_AD3010	+	+	+
Clostridium_sp._M62/1	+	+	+
unidentified_phage	+	+	+
Clostridium_sp._AM33-3	+	+	+
Lachnoclostridium_phocaeense	+	+	+
Anaerotruncus_sp._AF02-27	+	+	+
Eubacterium_sp._SB2	+	+	+
Bacteroides_pyogenes	+	+	+
Butyrivibrio_sp._LB2008	+	+	+
Catabacter_hongkongensis	+	+	+
Eubacterium_ramulus	+	+	+
Robinsoniella_peoriensis	+	+	+
Firmicutes_bacterium_CAG:424	+	+	+
Clostridium_sp._CAG:678	+	+	+
Intestinibacillus_sp._Marseille-P4005	+	+	+

Parabacteroides_sp._SN4	+	+	+
Eubacterium_sp._CAG:251	+	+	+
Anaerostipes_caccae	+	+	+
Lactobacillus_apodemi	+	+	+
Lachnospiraceae_bacterium_XBB2008	+	+	+
Ruminococcus_sp._HUN007	+	+	+
Clostridium_sp._AF50-3	+	+	+
Bacteroides_sp._43_108	+	+	+
Oribacterium_sp._NK2B42	+	+	+
Clostridiales_bacterium_AM23-16LB	+	+	+
Lachnospiraceae_bacterium_OF09-6	+	+	+
Firmicutes_bacterium_CAG:110	+	+	+
Clostridium_sp._CAG:299	+	+	+
Clostridium_sp._7_3_54FAA	+	+	+
Butyrivibrio_sp._AC2005	+	+	+
[Clostridium]_indolis	+	+	+
Clostridium_sp._AF32-12BH	+	+	+
Dorea_sp._OM02-2LB	+	+	+
Lachnospiraceae_bacterium_CAG:215	+	+	+
[Clostridium]_populeti	+	+	+
Flavonifractor_sp._An92	+	+	+
Firmicutes_bacterium_CAG:83	+	+	+
Clostridiales_bacterium_VE202-06	+	+	+
Clostridium_sp._C105KSO15	+	+	+
[Desulfotomaculum]_guttoideum	+	+	+
Alistipes_sp._An116	+	+	+
Prevotella_ruminicola	+	+	+
Dorea_sp._Marseille-P4003	+	+	+
Lactobacillus_salivarius	+	+	+
Clostridium_sp._12(A)	+	+	+
Clostridium_sp._CAG:411	+	+	+
Blautia_hansenii	+	+	+
Ruminococcaceae_bacterium_AF10-16	+	+	+
Clostridiales_bacterium_KLE1615	+	+	+
Coprococcus_sp._AM27-12LB	+	+	+
Eubacterium_ruminantium	+	+	+
Odoribacter_sp._AF15-53	+	+	+
bacterium_D16-36	+	+	+
Muribaculaceae_bacterium_Isolate-113_(HZI)	+	+	+
Bacteroides_nordii	+	+	+
Firmicutes_bacterium_CAG:194	+	+	+
Phocea_massiliensis	+	+	+
Lachnospiraceae_bacterium_V9D3004	+	+	+
Ruminiclostridium_cellobioparum	+	+	+
Clostridium_sp._AF15-17LB	+	+	+
Bacteroides_sp._4_1_36	+	+	+
Clostridium_sp._ATCC_BAA-442	+	+	+
Alistipes_sp._AF48-12	+	+	+
Lachnospiraceae_bacterium_NC2004	+	+	+
[Clostridium]_aerotolerans	+	+	+
Dielma_fastidiosa	+	+	+
Eubacterium_ventriosum	+	+	+
Coprococcus_sp._AF21-14LB	+	+	+
Lachnospiraceae_bacterium_TWA4	+	+	+
Bacteroides_sp._AF34-31BH	+	+	+

bacterium_D16-63	+	+	+
Halodesulfovibrio_marinisediminis	-	+	+
Ruminococcaceae_bacterium_D5	+	+	+
Clostridiales_bacterium_NK3B98	+	+	+
Flavonifractor_sp._An4	+	+	+
Ruminococcaceae_bacterium_TF06-43	+	+	+
Lachnospiraceae_bacterium_MA2020	+	+	+
Anaerotignum_propionicum	+	+	+
Alistipes_sp._AF17-16	+	+	+
Agathobaculum_desmolans	+	+	+
Firmicutes_bacterium_HGW-Firmicutes-16	+	+	+
uncultured_Oscillibacter_sp.	+	+	+
Intestinibacillus_massiliensis	+	+	+
Collinsella_tanakaei	+	+	+
Clostridiales_bacterium_Choco116	+	+	+
Massilimaliae_massiliensis	+	+	+
bacterium_D16-54	+	+	+
Butyrivibrio_sp._MC2013	+	+	+
Ruminococcus_sp._AF18-22	+	+	+
Cellulosilyticum_sp._WCF-2	+	+	+
Oscillibacter_sp._ER4	+	+	+
Prevotella_sp._P2-180	+	+	+
Firmicutes_bacterium_TM09-10	+	+	+
Butyricicoccus_porcorum	+	+	+
Firmicutes_bacterium_OM08-11AC	+	+	+
Holdemania_filiformis	+	+	+
Hydrogenoanaerobacterium_saccharovorans	+	+	+
Oceanospirillum_multiglobuliferum	+	+	+
Clostridium_sp._CAG:632	+	+	+
Clostridium_sp._AM29-11AC	+	+	+
[Eubacterium]_cellulosolvens	+	+	+
Salinibacter_ruber	+	+	+
Clostridium_sp._CAG:169	+	+	+
Hungateiclostridium_cellulolyticum	+	+	+
Parabacteroides_sp._D13	+	+	+
Ruminococcaceae_bacterium_AM28-23LB	+	+	+
Pseudoclostridium_thermosuccinogenes	+	+	+
Desulfovibrio_sp._An276	+	+	+
Agathobaculum_sp._Marseille-P7918	+	+	+
Herbinix_hemicellulosilytica	+	+	+
Butyrivibrio_sp._AE3004	+	+	+
Eubacterium_sp._CAG:38	+	+	+
Ruminiclostridium_papyrosolvens	+	+	+
Clostridium_sp._AF34-10BH	+	+	+
Eubacterium_sp._An3	+	+	+
Streptococcus_sp.	+	+	+
Prevotella_timonensis	+	+	+
Firmicutes_bacterium_CAG:176_63_11	+	+	+
Oribacterium_sp._FC2011	+	+	+
Paraprevotella_clara_CAG:116	+	+	+
Clostridium_sp._FS41	+	+	+
Clostridium_sp._AM49-4BH	+	+	+
Papillibacter_cinnamivorans	+	+	+
Subdoligranulum_sp._CAG:314	+	+	+
Ruminococcus_sp._OM05-10BH	+	+	+

Clostridiales_bacterium_Marseille-P2846	+	+	+
Desulfovibrio_sp._MES5	+	+	+
Bacteroides_heparinolyticus	+	+	+
Lachnospiraceae_bacterium_5_1_57FAA	+	+	+
Anaerotignum_neopropionicum	+	+	+
Lachnospiraceae_bacterium_oral_taxon_082	+	+	+
Lachnospiraceae_bacterium_oral_taxon_500	+	+	+
Clostridiaceae_bacterium_AF31-3BH	+	+	+
Clostridium_sp._E02	+	+	+
Desulfitobacterium_hafniense	+	+	+
Clostridiales_bacterium_TF09-2AC	+	+	+
Desulfovibrio_sp._DS-1	+	+	+
Bacillus_cereus	+	+	+
Bacteroides_togonis	+	+	+
Roseburia_sp._CAG:197	+	+	+
Roseburia_sp._OF03-24	+	+	+
Clostridiales_Family_XIII_bacterium	+	+	+
Alloprevotella_sp._E39	+	+	+
Bacteroides_helcogenes	+	+	+
Lachnospiraceae_bacterium_2_1_46FAA	+	+	+
Lachnospiraceae_bacterium_YSD2013	+	+	+
Bacteroides_sp._Marseille-P3684	+	+	+
Anaerostipes_sp._BG01	+	+	+
Firmicutes_bacterium_CAG:102	+	+	+
Clostridium_sp._Marseille-P2538	+	+	+
Ruthenibacterium_lactatiformans	+	+	+
Clostridium_hathewayi_CAG:224	+	+	+
Oscillibacter_sp._CAG:241_62_21	+	+	+
[Clostridium]_sphenoides	+	+	+
Ruminococcaceae_bacterium_P7	+	+	+
Butyrivibrio_sp._AE2032	+	+	+
Bacteroides_stercorirosoris	+	+	+
Bacteroides_ndongoniae	+	+	+
Faecalibacterium_sp._An192	+	+	+
Blautia_sp._An46	+	+	+
Faecalicatena_fissicatena	+	+	+
Clostridium_sp._D5	+	+	+
Akkermansia_muciniphila	+	+	+
Butyricimonas_sp._Marseille-P2440	+	+	+
Enterorhabdus_mucosicola	+	+	+
Lachnoclostridium_pacaense	+	+	+
Mobilisporobacter_senegalensis	+	+	+
Catonella_morbi	+	+	+
Clostridium_sp._CAG:167	+	+	+
Prevotella_sp._P5-92	+	+	+
Firmicutes_bacterium_CAG:176	+	+	+
Pygmaiobacter_massiliensis	+	+	+
Ruminococcus_sp._Marseille-P6503	+	+	+
Eubacterium_sp._ER2	+	+	+
Butyrivibrio_sp._INlla14	+	+	+
Anaerofilum_sp._An201	+	+	+
Bacteroides_timonensis	+	+	+
Acidiphilium_sp._CAG:727	+	+	+
Barnesiella_viscericola	+	+	+
Anaeromassilibacillus_sp._An172	+	+	+

Flavonifractor_sp._An135	+	+	+
Lachnospiraceae_bacterium_NE2001	+	+	+
Bacteroides_clarus	+	+	+
Fusicatenibacter_sp._2789STDY5834925	+	+	+
Bacteroides_sp._AF29-11	+	+	+
Candidatus_Soleaferrea_massiliensis	+	+	+
Firmicutes_bacterium_CAG:170	+	+	+
Gemmiger_sp._An87	+	+	+
Bacteroides_coprophilus	+	+	+
Muribaculaceae_bacterium_Isolate-114_(HZI)	+	+	+
Bacteroides_oleiciplenus	+	+	+
Lachnospiraceae_bacterium_FE2018	+	+	+
Anaeromassilibacillus_senegalensis	+	+	+
Firmicutes_bacterium_AF25-13AC	+	+	+
Pseudobutyrivibrio_ruminis	+	+	+
Mediterraneibacter_massiliensis	+	+	+
Blautia_sp._Marseille-P3201T	+	+	+
Niameybacter_massiliensis	+	+	+
Ruminococcus_sp._AM42-11	+	+	+
Prevotella_sp._885	+	+	+
uncultured_bacterium_BAC10G6	+	+	+
Robinsoniella_sp._RHS	+	+	+
Prevotellamassilia_timonensis	+	+	+
Subdoligranulum_sp._OF01-18	+	+	+
Clostridiaceae_bacterium_AF18-31LB	+	+	+
Bacteroides_sp._KCTC_15687	+	+	+
Streptococcus_suis	+	+	+
Clostridium_sp._CAG:307	+	+	+
Butyrivibrio_sp._AE2015	+	+	+
Butyrivibrio_sp._AE2005	+	+	+
Lachnospiraceae_bacterium_AB2028	+	+	+
Faecalimonas_umbilicata	+	+	+
Clostridiaceae_bacterium_MS3	+	+	+
Paeniclostridium_sordellii	+	+	+
Lachnospiraceae_bacterium_GAM79	+	+	+
Tannerella_forsythia	+	+	+
Ruminiclostridium_sufflavum	+	+	+
Blautia_sp._SG-772	+	+	+
Butyrivibrio_sp._CB08	+	+	+
Anaerostipes_sp._494a	+	+	+
Prevotella_sp._tf2-5	+	+	+
Coprococcus_sp._HPP0074	+	+	+
Alistipes_sp._cv1	+	+	+
Firmicutes_bacterium_CAG:24053_14	+	+	+
Dorea_sp._AM58-8	+	+	+
Sporobacter_termitidis	+	+	+
Subdoligranulum_sp._APC924/74	+	+	+
Sanguibacteroides_justesenii	+	+	+
Enterococcus_cecorum	+	+	+
Desulfomicrobium_orale	+	+	+
Prevotella_sp._CAG:1058	+	+	+
Barnesiella_intestinihominis	+	+	+
[Clostridium]_aminophilum	+	+	+
Campylobacter_fetus	+	+	+
Barnesiella_sp._An22	+	+	+

uncultured_bacterium_BAC25G1	+	+	+
Bacteroidales_bacterium_KHT7	+	+	+
Clostridium_sp._CAG:1024	+	+	+
Firmicutes_bacterium_AM10-47	+	+	+
Firmicutes_bacterium_AM43-11BH	+	+	+
Firmicutes_bacterium_AM41-5BH	+	+	+
Eubacterium_xylanophilum	+	+	+
[Clostridium]_sporosphaeroides	+	+	+
[Clostridium]_polysaccharolyticum	+	+	+
Agathobacter_ruminis	+	+	+
Desulfovibrio_sp._AM18-2	+	+	+
Pseudobutyrivibrio_xylanivorans	+	+	+
Flavonifractor_sp._An91	+	+	+
Ethanoligenens_harbinense	+	+	+
Butyricimonas_faecihominis	+	+	+
Clostridium_saccharobutylicum	+	+	+
Firmicutes_bacterium_CAG_194_44_15	+	+	+
Muribaculum_sp._An287	+	+	+
Bacteroides_salanitronis	+	+	+
Clostridium_sp._ASBs410	+	+	+
Lachnospiraceae_bacterium_KH1T2	+	+	+
Caproiciproducens_sp._NJN-50	+	+	+
Oribacterium_sp._C9	+	+	+
Prevotellaceae_bacterium	+	+	+
Mucinivorans_hirudinis	+	+	+
bacterium_MS4	+	+	+
Lachnospiraceae_bacterium_3_1_46FAA	+	+	+
Clostridiales_bacterium_VE202-16	+	+	+
Denitrovibrio_acetiphilus	+	+	+
Lachnospiraceae_bacterium_G11	+	+	+
Parabacteroides_sp._CAG:409	+	+	+
[Clostridium]_algidixylanolyticum	+	+	+
Firmicutes_bacterium_AF16-15	+	+	+
Subdoligranulum_sp._AF14-43	+	+	+
Prevotella_sp._Marseille-P8229	+	+	+
Massilimaliae_timonensis	+	+	+
Clostridiales_bacterium_CHKCI006	+	+	+
Rhizopus_delemar	+	+	+
Lutispora_thermophila	+	+	+
Bacteroidetes_bacterium_41-46	+	+	+
Thermoclostridium_stercorarium	+	+	+
Ruminococcaceae_bacterium_YRB3002	+	+	+
Oscillibacter_sp._KLE_1745	+	+	+
Anaerovorax_odorimutans	+	+	+
Chloroflexi_bacterium_RBG_19FT_COMBO_55_16	+	+	+
Bacteroides_phage_crAss001	+	+	+
butyrate-producing_bacterium_SS3/4	+	+	+
Fusobacterium_naviforme	+	+	+
Peptoanaerobacter_stomatis	+	+	+
Prevotella_intermedia	+	+	+
Parabacteroides_sp._20_3	+	+	+
Ruminococcaceae_bacterium_cv2	+	+	+
Clostridium_sp._SY8519	+	+	+
Faecalibacterium_sp._An58	+	+	+
Clostridium_sp._AM42-4	+	+	+

uncultured_murine_large_bowel_bacterium_BAC_31B	+	+	+
Clostridium_sp._HMb25	+	+	+
Butyrivibrio_crossotus	+	+	+
Clostridium_perfringens	+	+	+
Clostridium_sp._AF36-18BH	+	+	+
Aminipila_sp._JN-39	+	+	+
Lachnospiraceae_bacterium_P6A3	+	+	+
Clostridiaceae_bacterium_AF42-6	+	+	+
Lachnospiraceae_bacterium_MC2017	+	+	+
Butyrivibrio_sp._NC3005	+	+	+
Ruminococcaceae_bacterium_FB2012	+	+	+
Prevotella_sp._CAG:755	+	+	+
Subdoligranulum_sp._4_3_54A2FAA	+	+	+
Lachnospiraceae_bacterium_G41	+	+	+
Oscillospiraceae_bacterium_VE202-24	+	+	+
Clostridium_sp.	+	+	+
Firmicutes_bacterium_CAG:272	+	+	+
[Clostridium]_cocleatum	+	+	+
Pseudoflavonifractor_sp._An44	+	+	+
Butyrivibrio_sp._M55	+	+	+
Lachnospiraceae_bacterium_P6B14	+	+	+
Parabacteroides_sp._AF48-14	+	+	+
Bacteroides_sp._AF39-16AC	+	+	+
Lactobacillus_fermentum	+	+	+
Parabacteroides_sp._Marseille-P3668	+	+	+
Tyzzerella_sp._An114	+	+	+
Clostridium_sp._OF09-36	+	+	+
Firmicutes_bacterium_CAG:129_59_24	+	+	+
Prevotella_oris	+	+	+
Christensenella_massiliensis	+	+	+
Selenomonadales_bacterium	+	+	+
Butyrivibrio_sp._MB2005	+	+	+
Bacteroides_sp._HPS0048	+	+	+
Lachnospiraceae_bacterium_AC2031	+	+	+
Parabacteroides_sp._AF17-28	+	+	+
Lachnospiraceae_bacterium_YSB2008	+	+	+
Lachnospiraceae_bacterium_NK3A20	+	+	+
Lachnoclostridium_sp._An181	+	+	+
Alistipes_finegoldii_CAG:68	+	+	+
Bacteroides_sp._CAG:530	+	+	+
Clostridiales_bacterium_41_21_two_genomes	+	+	+
Clostridium_sp._W14A	+	+	+
Lachnospiraceae_bacterium_NK4A179	+	+	+
Eubacterium_limosum	+	+	+
Bacteroides_sp._3_1_13	+	+	+
Dorea_sp._AGR2135	+	+	+
Francisella_tularensis	+	-	+
Anaerolineaceae_bacterium	+	+	+
Ruminococcus_callidus	+	+	+
Provencibacterium_massiliense	+	+	+
Agathobaculum_butyriciproducens	+	+	+
Ruminococcus_sp._AF21-42	+	+	+
Butyrivibrio_sp._WCD2001	+	+	+
Clostridiales_bacterium_36_14	+	+	+
Prevotella_oryzae	+	+	+

Erysipelotrichaceae_bacterium_6_1_45	+	+	+
Clostridium_sp._CAG:307_30_263	+	+	+
Eubacterium_sp._CAG:252	+	+	+
Lachnospira_pectinoschiza	+	+	+
Eubacterium_barkeri	+	+	+
Treponema_primitia	+	+	+
Criibacterium_bergeronii	+	+	+
Campylobacter_jejuni	+	+	+
Ruminococcaceae_bacterium_HV4-5-B5C	+	+	+
Odoribacter_sp._43_10	+	+	+
Christensenella_sp._AF73-05CM02	+	+	+
Clostridium_sp._CAG:43	+	+	+
Fusobacterium_necrophorum	+	+	+
uncultured_Butyricicoccus_sp.	+	+	+
Bacteroidales_bacterium_CF	+	+	+
Oscillibacter_sp.	+	+	+
Bacteroides_sp._3_1_40A	+	+	+
Parabacteroides_sp._426-9	+	+	+
Clostridium_tetani	+	+	+
Butyrivibrio_sp._ob235	+	+	+
Anaerotruncus_sp._22A2-44	+	+	+
Roseburia_sp._OM02-15	+	+	+
Anaerotruncus_sp._CAG:528	+	+	+
Bacteroides_congonensis	+	+	+
Anaeromassilibacillus_sp._Marseille-P4683	+	+	+
Lachnospiraceae_bacterium_NLAE-zl-G231	+	+	+
Drancourtella_sp._An210	+	+	+
Alloprevotella_sp.	+	+	+
Dorea_sp._AF36-15AT	+	+	+
Candidatus_Gastranaerophilales_bacterium_HUM_17	+	+	+
Parabacteroides_sp._Marseille-P4001	+	+	+
Clostridium_sp._CAG:590	+	+	+
Oribacterium_sp._oral_taxon_078	+	+	+
Lachnoanaerobaculum_saburreum	+	+	+
Coriobacteriia_bacterium	+	+	+
Clostridium_tyrobutyricum	+	+	+
Firmicutes_bacterium_CAG:103	+	+	+
Roseburia_sp._AM59-24XD	+	+	+
Prevotella_sp._CAG:1185	+	+	+
Ruminococcus_sp._AF41-9	+	+	+
Muribaculaceae_bacterium_Isolate-100_(HZI)	+	+	+
Ruminococcus_sp._AF20-12LB	+	+	+
Prevotella_sp._CAG:5226	+	+	+
Candidatus_Stoquefichus_massiliensis	+	+	+
Clostridiales_bacterium_70B-A	+	+	+
Ruminococcus_sp._AF34-12	+	+	+
Eubacterium_sp._CAG:76	+	+	+
Phascolarctobacterium_faecium	+	+	+
Clostridium_collagenovorans	+	+	+
Clostridium_sp._TF01-11	+	+	+
Eubacterium_sp._CAG:248	+	+	+
Barnesiella_sp._An55	+	+	+
uncultured_bacterium_fosmid_pJB28H11	+	+	+
Ruminococcus_sp._AF46-10NS	+	+	+
Bacteroides_sp._AM25-34	+	+	+

Candidatus_Stoquefichus_sp._KLE1796	+	+	+
Clostridium_sp._CAG:413	+	+	+
Bacteroidales_bacterium_43_8	+	+	+
Butyrivibrio_sp._CAG:318	+	+	+
Geovibrio_thiophilus	+	+	+
Clostridium_sp._AM22-11AC	+	+	+
Clostridiales_bacterium_COT073_COT-073	+	+	+
Clostridium_sp._OM02-18AC	+	+	+
Clostridium_sp._AM32-2	+	+	+
Faecalitalea_cylindroides	+	+	+
Vallitalea_sp._S15	+	+	+
Firmicutes_bacterium_CAG:56	+	+	+
Bacteroides_sp._AR29	+	+	+
Lachnospiraceae_bacterium_KHCPX20	+	+	+
Clostridium_sp._CAG:253	+	+	+
Paenibacillus_odorifer	+	+	+
Bacteroides_gallinarum	+	+	+
Catenibacterium_mitsuokai	+	+	+
Clostridium_sp._AM34-11AC	+	+	+
uncultured_Lachnospira_sp.	+	+	+
Clostridiales_bacterium_GWF2_38_85	+	+	+
Parabacteroides_chinchillae	+	+	+
Bacteroides_sp._AM32-11AC	+	+	+
Mediterranea_sp._An20	+	+	+
Butyricimonas_sp._An62	+	+	+
Pelosinus_sp._UFO1	+	+	+
bacterium_F082	+	+	+
Bacillus_thuringiensis	+	+	+
Eubacterium_rectale_CAG:36	+	+	+
Ruminococcus_sp._AF42-9BH	+	+	+
Desulfovibrio_alaskensis	+	+	+
Clostridium_sp._CAG:798	+	+	+
Anaerofustis_stercorihominis	+	+	+
Flavonifractor_sp._An9	+	+	+
Desulfosporosinus_sp._BICA1-9	+	+	+
Gemmiger_sp._An50	+	+	+
Coprococcus_sp._HPP0048	+	+	+
Clostridiaceae_bacterium_AM27-36LB	+	+	+
Tissierella_sp._P1	+	+	+
Anaerovibrio_lipolyticus	+	+	+
Bacteroides_sp._CAG:754	+	+	+
Bacteroides_reticulotermitis	+	+	+
Prevotella_sp._KH2C16	+	+	+
Lachnospira_multipara	+	+	+
Firmicutes_bacterium_CAG:449	+	+	+
Clostridium_saccharoperbutylacetonicum	+	+	+
Vallitalea_guaymasensis	+	+	+
Faecalibacterium_sp._An122	+	+	+
Terrisporobacter_glycolicus	+	+	+
Helicobacter_trogontum	+	+	+
Treponema_brennaborense	+	+	+
Lachnospiraceae_bacterium_ND2006	+	+	+
Neobitarella_massiliensis	+	+	+
Filifactor_alocis	+	+	+
Ruminococcus_sp._AF12-5	+	+	+

Firmicutes_bacterium_CAG:65_45_313	+	+	+
Alistipes_sp._CAG:157	+	+	+
Clostridiales_bacterium_AF36-10	+	+	+
Clostridia_bacterium_BRH_c25	+	+	+
Mediterraneibacter_sp._KCTC_15684	+	+	+
Parabacteroides_sp._An277	+	+	+
Coprobacter_sp.	+	+	+
Roseburia_sp._AM16-25	+	+	+
Clostridia_bacterium_UC5.1-2H11	+	+	+
Muribaculaceae_bacterium_Isolate-083_(Janvier)	+	+	+
Bacteroides_ilei	+	+	+
Lachnospiraceae_bacterium_3_1_57FAA_CT1	+	+	+
Lachnoclostridium_sp._Marseille-P6806	+	+	+
Subdoligranulum_sp._AM16-9	+	+	+
Parasporobacterium_paucivorans	+	+	+
Blautia_hydrogenotrophica_CAG:147	+	+	+
Oribacterium_sp._WCC10	+	+	+
Flexilinea_flocculi	+	+	+
Alloprevotella_rava	+	+	+
Clostridium_sp._AF37-5	+	+	+
Petroclostridium_xylanilyticum	+	+	+
Anaerotruncus_massiliensis	+	+	+
Treponema_bryantii	+	+	+
bacterium_P3	+	+	+
Clostridiaceae_bacterium_AF29-16BH	+	+	+
Traorella_massiliensis	+	+	+
Hungateiclostridium_thermocellum	+	+	+
Coprobacillus_cateniformis	+	+	+
Prevotella_brevis	+	+	+
Firmicutes_bacterium_CAG:238	+	+	+
Bacteroides_sp._CAG:144	+	+	+
Prevotella_sp._P4-65	+	+	+
Alistipes_inops	+	+	+
Massilioclostridium_coli	+	+	+
Prevotella_sp._10(H)	+	+	+
Subdoligranulum_sp.	+	+	+
Massiliomicrobiota_timonensis	+	+	+
Paenibacillus_chondroitinus	+	+	+
Sedimentibacter_sp._B4	+	+	+
Lachnospiraceae_bacterium_NK4A136	+	+	+
Clostridiales_bacterium_VE202-03	+	+	+
Oribacterium_sp._P6A1	+	+	+
Eubacterium_sp._OM08-24	+	+	+
Alloprevotella_sp._OH1205_COT-284	+	+	+
Candidatus_Stoquefichus_sp._SB1	+	+	+
Campylobacter_coli	+	+	+
Helicobacter_cinaedi	+	+	+
Desulfovibrio_bizertensis	+	+	+
Lachnospiraceae_bacterium_AC2029	+	+	+
Lachnospiraceae_bacterium_NK4A144	+	+	+
Lachnospiraceae_bacterium_C7	+	+	+
Monoglobus_pectinilyticus	+	+	+
Blautia_sp._KLE_1732	+	+	+
Butyricicoccus_sp._AM28-25	+	+	+
Clostridiaceae_bacterium	+	+	+

Desulfobulbus_oralis	+	+	+
Eubacteriaceae_bacterium_CHKCI005	+	+	+
Lachnoanaerobaculum_umeaense	+	+	+
Tenericutes_bacterium_HGW-Tenericutes-4	+	+	+
Clostridia_bacterium_UC5.1-1D10	+	+	+
Lactococcus_lactis	+	+	+
Clostridiales_bacterium_VE202-07	+	+	+
Clostridium_sp._OF03-18AA	+	+	+
Acetanaerobacterium_elongatum	+	+	+
Chlamydia_psittaci	-	-	+
Oribacterium_sp._KHPX15	+	+	+
Bacteroidetes_bacterium_GWF2_40_14	+	+	+
Succinimonas_amylolytica	+	+	+
Sebaldella_termitidis	+	+	+
Firmicutes_bacterium_CAG:137_57_8	+	+	+
Firmicutes_bacterium_CAG:65	+	+	+
Firmicutes_bacterium_AF22-6AC	+	+	+
Ruminococcus_sp._37_24	+	+	+
Lachnospiraceae_bacterium_XBD2001	+	+	+
Clostridiales_bacterium_DRI-13	+	+	+
Drancourtella_sp._An177	+	+	+
Firmicutes_bacterium_CAG:555	+	+	+
Dysgonomonas_capnocytophagoides	+	+	+
Clostridium_nexile_CAG:348	+	+	+
Prevotella_melaninogenica	+	+	+
Bacteroides_sp._An322	+	+	+
Oxalobacter_formigenes	+	+	+
Eubacterium_sp._CAG:274	+	+	+
Alistipes_sp._CAG:53	+	+	+
Clostridium_sp._CAG:149	+	+	+
Desulfovibrio_bastinii	+	+	+
Lachnospiraceae_bacterium_XBB1006	+	+	+
Succinivibrio_dextrinosolvens	+	+	+
uncultured_Anaerotruncus_sp.	+	+	+
Eubacterium_sp._AF19-12LB	+	+	+
Sellimonas_intestinalis	+	+	+
Anaerotruncus_sp._Marseille-P4302	+	+	+
Deltaproteobacteria_bacterium	+	+	+
Prevotella_sp._OH937_COT-195	+	+	+
Clostridium_sp._AM16-23	+	+	+
Clostridia_bacterium_UC5.1-1D1	+	+	+
Christensenella_timonensis	+	+	+
Firmicutes_bacterium_HGW-Firmicutes-9	+	+	+
Parabacteroides_sp._AM08-6	+	+	+
Blautia_sp._Marseille-P3087	+	+	+
Harryflintia_acetispora	+	+	+
Asaccharobacter_celatus	+	+	+
Clostridiales_bacterium_44_9	+	+	+
Parabacteroides_sp._Marseille-P3160	+	+	+
Oscillibacter_sp._CAG:241	+	+	+
Bacteroides_sp._AM16-15	+	+	+
Cellulosilyticum_lentocellum	+	+	+
Eubacterium_uniforme	+	+	+
Firmicutes_bacterium_AF36-19BH	+	+	+
Fusobacterium_ulcerans	+	+	+

Parabacteroides_timonensis	+	+	+
Eubacterium_sp._CAG:603	+	+	+
Fusobacterium_mortiferum	+	+	+
Bacteroides_massiliensis	+	+	+
Prevotella_sp._CAG:617	+	+	+
Acinetobacter_baumannii	+	+	+
Clostridium_intestinale	+	+	+
Bacteroides_ihuae	+	+	+
Clostridium_sp._AF22-10	+	+	+
Oxobacter_pfennigii	+	+	+
Bacteriodetes_bacterium_13_1_20CM_4_60_6	+	+	+
Clostridium_lundense	+	+	+
Bacteroides_sp._AF39-11AC	+	+	+
Ruminococcus_sp._AF31-8BH	+	+	+
Clostridium_sp._AM42-36	+	+	+
Clostridium_sp._CAG:230	+	+	+
Lachnospiraceae_bacterium_9_1_43BFAA	+	+	+
Firmicutes_bacterium_CAG:466	+	+	+
Clostridium_bolteae_CAG:59	+	+	+
Ruminococcus_sp._AM58-7XD	+	+	+
Clostridiaceae_bacterium_OF09-1	+	+	+
Firmicutes_bacterium_CAG:791	+	+	+
Clostridiales_bacterium_MCWD3	+	+	+
Bacteroides_sp._AM41-16	+	+	+
Clostridium_sp._CAG:62_40_43	+	+	+
Acetobacterium_bakii	+	+	+
Clostridium_sp._TM06-18	+	+	+
Ndongobacter_massiliensis	+	+	+
Prevotella_sp._Marseille-P4119	+	+	+
Prevotella_stercorea	+	+	+
Fusobacterium_nucleatum	+	+	+
Bacteroides_sp._1_1_14	+	+	+
Phascolarctobacterium_sp.	+	+	+
Bacillus_phage_AR9	+	+	+
Butyrivibrio_sp._FC2001	+	+	+
Eubacterium_sp._AM47-9	+	+	+
[Clostridium]_viride	+	+	+
Pseudoflavonifractor_sp._An187	+	+	+
Prevotellaceae_bacterium_HUN156	+	+	+
Parabacteroides_sp._AF14-59	+	+	+
Fusobacteriia_bacterium_4572_132	+	+	+
Lachnospiraceae_bacterium_CAG:364	+	+	+
Clostridium_sp._CAG:62	+	+	+
Clostridium_carnis	+	+	+
Clostridiales_bacterium_VE202-21	+	+	+
Firmicutes_bacterium_AF36-3BH	+	+	+
Roseburia_sp._CAG:471	+	+	+
Bacteroides_sp._An269	+	+	+
Eubacterium_sp._CAG:180	+	+	+
Butyrivibrio_sp._TB	+	+	+
Prevotella_bivia	+	+	+
Butyrivibrio_sp._WCD3002	+	+	+
Faecalibacterium_sp._AF10-46	+	+	+
Clostridium_acetobutylicum	+	+	+
Parabacteroides_gordonii	+	+	+

Pseudobutyrivibrio_sp._YE44	+	+	+
Eubacterium_sp._68-3-10	+	+	+
Clostridium_sp._AM25-23AC	+	+	+
Lachnospiraceae_bacterium_AC3007	+	+	+
Clostridium_sp._CAG:127	+	+	+
Clostridium_sp._CAG:81	+	+	+
Clostridium_chromiireducens	+	+	+
Clostridium_sp._CAG:221	+	+	+
Blautia_sp._OM07-19	+	+	+
Natronincola_ferrireducens	+	+	+
Faecalibacterium_sp._OM04-11BH	+	+	+
Bacteroides_luti	+	+	+
Holdemania_sp._Marseille-P2844	+	+	+
Massilibacillus_massiliensis	+	+	+
Clostridium_sp._CT4	+	+	+
Porphyromonadaceae_bacterium_H1	+	+	+
Alistipes_sp._56_11	+	+	+
Peptococcaceae_bacterium_BRH_c23	+	+	+
Clostridiales_bacterium_VE202-01	+	+	+
Clostridium_scatologenes	+	+	+
[Bacteroides]_pectinophilus	+	+	+
Lachnospiraceae_bacterium_AM25-17	+	+	+
Ruminococcus_sp._CAG:177	+	+	+
Chloroflexi_bacterium	+	+	+
Eubacterium_oxidoreducens	+	+	+
Butyrivibrio_sp._INlla18	+	+	+
Blautia_sp._AM23-13AC	+	+	+
Bacteroides_sp._CAG:633	+	+	+
Fusibacter_sp._3D3	+	+	+
Neglecta_sp._Marseille-P3890	+	+	+
Bacteroides_neonati	+	+	+
Youngiibacter_fragilis	+	+	+
Butyrivibrio_sp._X503	+	+	+
Prevotella_sp._tc2-28	+	+	+
Clostridium_disporicum	+	+	+
Microviridae_sp.	+	+	+
Ruminiclostridium_josui	+	+	+
Dorea_formicigenerans_CAG:28	+	+	+
Helicobacter_magdeburgensis	+	+	+
Clostridium_sp._AF34-13	+	+	+
Prevotella_sp._CAG:1092	+	+	+
Coprococcus_sp._CAG:131-related_45_246	+	+	+
Clostridium_sp._BL8	+	+	+
Firmicutes_bacterium_CAG:24	+	+	+
Eggerthella_lenta	+	+	+
Clostridium_sp._CAG:571	+	+	+
Clostridium_oryzae	+	+	+
Roseburia_sp._AF15-21	+	+	+
Clostridium_sp._CAG:448	+	+	+
Bacteroides_faecis	+	+	+
Oribacterium_parvum	+	+	+
Weissella_cibaria	+	+	+
Coprococcus_sp._AF18-48	+	+	+
Holdemania_massiliensis	+	+	+
Clostridium_sp._CAG:288	+	+	+

Odoribacter_splanchnicus_CAG:14	+	+	+
Bacteroides_sp._AM44-19	+	+	+
Clostridiales_bacterium_oral_taxon_876	+	+	+
Bacteroidales_bacterium_Barb4	+	+	+
Candidatus_Saccharibacteria_bacterium	+	+	+
Lachnoclostridium_sp._YL32	+	+	+
Ruminococcus_sp._AM40-10AC	+	+	+
Clostridium_jeddahense	+	+	+
Butyrivibrio_sp._MC2021	+	+	+
Ruminococcus_sp._CAG:563	+	+	+
Prevotella_bryantii	+	+	+
Desulfitobacterium_metallireducens	+	+	+
Desulfovibrio_hydrothermalis	+	+	+
Clostridium_paraputrificum	+	+	+
Ruminococcus_sp._AM31-15AC	+	+	+
Butyrivibrio_sp._AE3009	+	+	+
Christensenella_minuta	+	+	+
Absiella_dolichum	+	+	+
Candidatus_Melainabacteria_bacterium	+	+	+
Clostridium_sp._CAG:226	+	+	+
Bacteroides_barnesiae	+	+	+
Herbinix_luporum	+	+	+
Bacteroides_sp._CAG:462	+	+	+
Listeria_monocytogenes	+	+	+
Lachnospiraceae_bacterium_AC2012	+	+	+
Anaerotruncus_sp._CAG:390	+	+	+
Roseburia_sp._CAG:18	+	+	+
Olsenella_sp._An285	+	+	+
Muribaculum_sp._An289	+	+	+
Butyrivibrio_sp._Su6	+	+	+
Cohnella_kolymensis	+	+	+
uncultured_Dorea_sp.	+	+	+
Flavonifractor_sp._An112	+	+	+
Ruminococcus_sp._AF32-2AC	+	+	+
Clostridium_sp._AF23-8	+	+	+
Blautia_sp._OM05-6	+	+	+
Gilliamella_apicola	+	+	+
Parabacteroides_sp._AF18-52	+	+	+
Fretibacterium_sp._OH1220_COT-178	+	+	+
Blautia_sp._KGMB01111	+	+	+
Bacteroides_sp._CAG:714	+	+	+
Megasphaera_elsdenii	+	+	+
Porphyromonas_gulae	+	+	+
Ruminococcus_sp._NK3A76	+	+	+
Clostridium_sp._BL-8	+	+	+
Eubacterium_coprostanoligenes	+	+	+
Parvibacter_caecicola	+	+	+
Ruminiclostridium_hungatei	+	+	+
Bacteroides_sp._4_3_47FAA	+	+	+
Bacteroides_paurosaccharolyticus	+	+	+
Sporomusa_acidovorans	+	+	+
Acidobacteria_bacterium	+	+	+
Bacteroides_graminisolvens	+	+	+
Erysipelotrichaceae_bacterium_NK3D112	+	+	+
Clostridium_sp._AM34-9AC	+	+	+

uncultured_bacterium_fosmid_pJB42G5	+	+	+
Faecalibacterium_sp._CAG:74_58_120	+	+	+
Prevotella_buccalis	+	+	+
Clostridium_sp._SCN_57-10	+	+	+
Ruminococcus_sp._CAG:488	+	+	+
butyrate-producing_bacterium_SM4/1	+	+	+
Ruminococcus_sp._AF17-22AC	+	+	+
Roseburia_sp._AF42-8	+	+	+
Phascolarctobacterium_succinatutens	+	+	+
Peptococcaceae_bacterium_1109	+	+	+
Bacteroidetes_bacterium_GWE2_39_28	+	+	+
Staphylococcus_sp._CAG:324	+	+	+
Prevotella_sp._AM42-24	+	+	+
Lachnospiraceae_bacterium_RM5	+	+	+
Butyrivibrio_sp._VCD2006	+	+	+
Coriobacteriaceae_bacterium	+	+	+
Prevotella_salivae	+	+	+
Butyrivibrio_sp._WCE2006	+	+	+
Prevotella_saccharolytica	+	+	+
Clostridium_sp._AM51-4	+	+	+
Bacillus_toyonensis	+	+	+
Kandleria_vitulina	+	+	+
Ruminococcus_sp._CAG:624	+	+	+
Clostridium_sp._AF36-4	+	+	+
Ruminococcus_sp._FC2018	+	+	+
Eubacterium_sp._AF17-7	+	+	+
Blautia_sp._BCRC_81119	+	+	+
Pseudobutyrivibrio_sp._ACV-2	+	+	+
Clostridium_sporogenes	+	+	+
Acidaminococcus_sp._Marseille-P4266	+	+	+
Desulfovibrio_frigidus	+	+	+
Pseudobutyrivibrio_sp._LB2011	+	+	+
Halodesulfovibrio_spirochaetisodalis	+	+	+
Bacteroides_sp._2_2_4	+	+	+
Calderihabitans_maritimus	+	+	+
Desulfovibrio_sp._HK-II	+	+	+
Firmicutes_bacterium_AM55-24TS	+	+	+
Ruminococcus_sp._OM06-36AC	+	+	+
Synergistes_jonesii	+	+	+
uncultured_Faecalibacterium_sp.	+	+	+
Clostridiales_bacterium_GWF2_36_10	+	+	+
Faecalibacterium_sp._An77	+	+	+
Butyrivibrio_sp._INlla21	+	+	+
Desulfovibrio_salexigens	+	+	+
Murine_leukemia_virus	+	+	+
Anaerotruncus_rubiinfantis	+	+	+
Desulfosporosinus_sp._OL	+	+	+
Ruminococcus_sp._AM41-2AC	+	+	+
Butyricicoccus_sp._AM42-5AC	+	+	+
Faecalitalea_sp._Marseille-P3755	+	+	+
Clostridium_sp._CAG:964	+	+	+
Massiliomicrobiota_sp._An80	+	+	+
Clostridium_sp._AF02-29	+	+	+
Eubacterium_sp._CAG:115	+	+	+
uncultured_Dysgonomonas_sp.	+	+	+

Bacteroidales_bacterium_Barb7	+	+	+
Faecalicoccus_pleomorphus	+	+	+
Holdemanella_biformis	+	+	+
Bacteroides_sp._14(A)	+	+	+
Clostridium_leptum_CAG:27	+	+	+
Alkaliphilus_peptidifermentans	+	+	+
Clostridium_sp._Marseille-P4200	+	+	+
Lactobacillus_acidophilus	+	+	+
Dorea_sp._AM13-35	+	+	+
Tannerella_sp._6_1_58FAA_CT1	+	+	+
Blautia_sp._AF17-9LB	+	+	+
Clostridium_sp._TF08-15	+	+	+
Megamonas_hypermegale	+	+	+
Syntrophobotulus_glycolicus	+	+	+
Bacillus_megaterium	+	+	+
Ruminococcus_sp._CAG:353	+	+	+
Desulfoplanes_formicivorans	+	+	+
Prevotella_sp._CAG:1320	+	+	+
Butyrivibrio_sp._LC3010	+	+	+
Lachnospiraceae_bacterium_C6A11	+	+	+
Lactomassilus_timonensis	+	+	+
Erysipelatoclostridium_ramosum	+	+	+
Ruminococcus_sp._AF42-10	+	+	+
Firmicutes_bacterium_CAG:145	+	+	+
Coprococcus_sp._AM14-16	+	+	+
Dorea_sp._Marseille-P4042	+	+	+
Prevotella_amnii	+	+	+
Dysgonomonas_sp._BGC7	+	+	+
Coprococcus_sp._AF27-8	+	+	+
Clostridium_bornimense	+	+	+
Bacteroides_pectinophilus_CAG:437	+	+	+
Hungateiclostridium_clariflavum	+	+	+
Butyricicoccus_sp._AM05-1	+	+	+
Clostridiaceae_bacterium_BRH_c20a	+	+	+
uncultured_bacterium_fosmid_pJB135F11	+	+	+
Coprococcus_eutactus_CAG:665	+	+	+
Fournierella_massiliensis	+	+	+
Butyrivibrio_sp._VCB2006	+	+	+
Dorea_sp._AF24-7LB	+	+	+
Ruminococcaceae_bacterium_KH2T8	+	+	+
Prevotella_sp._P4-119	+	+	+
Acetobacterium_woodii	+	+	+
Firmicutes_bacterium_CAG:212	+	+	+
Anaerolineaceae_bacterium_oral_taxon_439	+	+	+
Bacteroides_sp._CAG:875	+	+	+
Anaeromassilibacillus_sp._Marseille-P3371	+	+	+
Solobacterium_moorei	+	+	+
Prevotella_sp._CAG:1124	+	+	+
Clostridium_sp._AM45-5	+	+	+
[Clostridium]_saccharogumia	+	+	+
Ruminococcus_sp._TM10-9AT	+	+	+
Prevotella_multisaccharivorax	+	+	+
Mediterranea_massiliensis	+	+	+
Catonella_sp.	+	+	+
Peptococcaceae_bacterium_CEB3	+	+	+

Pseudobacteroides_cellulosolvens	+	+	+
Clostridium_sp._CAG:122	+	+	+
Christensenella_sp._Marseille-P3954	+	+	+
Hungateiclostridium_saccincola	+	+	+
Clostridium_sp._Ade.TY	+	+	+
Firmicutes_bacterium_CAG:227	+	+	+
Candidatus_Desulfovibrio_trichonymphae	+	+	+
Clostridium_sp._CAG:7	+	+	+
Prevotella_sp._CAG:255	+	+	+
Firmicutes_bacterium_CAG:631	+	+	+
Bacteroides_sp._OM08-11	+	+	+
Fusobacterium_varium	+	+	+
Enterococcus_villorum	+	+	+
uncultured_bacterium_URE4	+	+	+
Clostridium_sp._LS	+	+	+
Hathewaya_proteolytica	+	+	+
Ruminococcus_sp._AM57-5	+	+	+
Clostridium_sp._OM05-9	+	+	+
Azospirillum_sp._47_25	+	+	+
Clostridium_merdae	+	+	+
Porphyromonas_asaccharolytica	+	+	+
Clostridium_sp._TF06-15AC	+	+	+
Ruminococcus_sp._CAG:108-related_41_35	+	+	+
Thermotalea_metallivorans	+	+	+
Clostridiales_bacterium_VE202-13	+	+	+
Roseburia_sp._AM23-20	+	+	+
Succiniclasticum_ruminis	+	+	+
Eubacterium_sp._CAG:841	+	+	+
Firmicutes_bacterium_HGW-Firmicutes-12	+	+	+
[Clostridium]_ultunense	+	+	+
Desulfitibacter_alkalitolerans	+	+	+
Tyzzerella_sp._Marseille-P3062	+	+	+
Treponema_socranskii	+	+	+
Beduini_massiliensis	+	+	+
Phocaeicola_abscessus	+	+	+
Alkaliphilus_transvaalensis	+	+	+
Paenibacillus_glycanilyticus	+	+	+
Clostridium_sp._CAG:356	+	+	+
Alloprevotella_tannerae	+	+	+
Coriobacteriales_bacterium_OH1046	+	+	+
Butyrivibrio_sp._NC2002	+	+	+
Bacillus_circulans	+	+	+
Rhodobacteraceae_bacterium_CH30	+	+	+
Gammaproteobacteria_bacterium	+	+	+
Lachnospiraceae_bacterium_XPB1003	+	+	+
Firmicutes_bacterium_CAG:313	+	+	+
Lachnospiraceae_bacterium_oral_taxon_096	+	+	+
Dubosiella_newyorkensis	+	+	+
Desulfosporosinus_youngiae	+	+	+
Prevotella_sp._kh1p2	+	+	+
Eggerthella_sp._CAG:298	+	+	+
Clostridium_sp._OM05-5BH	+	+	+
Pseudobutyrivibrio_sp._AR14	+	+	+
Clostridiales_bacterium_VE202-27	+	+	+
Bacteroides_mediterraneensis	+	+	+

Lachnobacterium_bovis	+	+	+
Clostridium_sp._42_12	+	+	+
Firmicutes_bacterium_AM59-13	+	+	+
Selenomonas_flueggei	+	+	+
Candidatus_Symbiothrix_dinenymphae	+	+	+
Clostridium_cadaveris	+	+	+
Eubacterium_pyruvativorans	+	+	+
[Clostridium]_cellulosi	+	+	+
Prevotella_sp._CAG:732	+	+	+
Clostridium_baratii	+	+	+
Blautia_sp._AF19-1	+	+	+
Bacteroides_sp._AM10-21B	+	+	+
Pedobacter_steynii	+	+	+
Prevotella_sp._BP1-145	+	+	+
Eggerthella_sp._YY7918	+	+	+
Caenibacillus_caldisaponilyticus	+	+	+
Piromyces_sp._E2	+	+	+
Clostridium_sp._CAG:465	+	+	+
Candidatus_Poribacteria_bacterium	+	+	+
Elizabethkingia_anophelis	+	+	+
Eubacterium_sp._CAG:161	+	+	+
Firmicutes_bacterium_OM07-11	+	+	+
Ruminococcus_champanellensis	+	+	+
Alistipes_sp._ZOR0009	+	+	+
Gemmiger_sp._An194	+	+	+
Geosporobacter_ferrireducens	+	+	+
Desulfovibrio_mexicanus	+	+	+
Desulfovibrio_sp._G11	+	+	+
Eubacterium_sp._am_0171	+	+	+
Clostridium_sp._CL-6	+	+	+
Parabacteroides_sp._TM07-1AC	+	+	+
Bacteroides_sp._AF26-10BH	+	+	+
Clostridium_cavendishii	+	+	+
Asaccharospora_irregularis	+	+	+
Paraeggerthella_hongkongensis	+	+	+
Ruminococcus_sp._AM28-41	+	+	+
Clostridium_sp._AM27-31LB	+	+	+
Eubacterium_sp._AM05-23	+	+	+
Firmicutes_bacterium_AM31-12AC	+	+	+
Bacteroides_fragilis_CAG:558	+	+	+
Acholeplasma_sp._CAG:878	+	+	+
Azospirillum_sp.	+	+	+
Bacteroides_sp._9_1_42FAA	+	+	+
Pseudobutyrivibrio_sp._MD2005	+	+	+
Clostridium_sp._AF20-7	+	+	+
Paenibacillus_ginsengarvi	+	+	+
bacterium_D16-51	+	+	+
Clostridium_sp._SS2/1	+	+	+
Jonquetella_anthropi	+	+	+
Gemmiger_formicilis	+	+	+
Pseudobutyrivibrio_sp._49	+	+	+
Clostridium_sp._AM09-51	+	+	+
Eubacterium_sp._AF34-35BH	+	+	+
Verrucomicrobia_bacterium	+	+	+
Ruminococcus_sp._AF31-14BH	+	+	+

Faecalibacterium_sp._An121	+	+	+
Butyrivibrio_sp._FCS014	+	+	+
Prevotella_sp.	+	+	+
Eubacterium_sp._Marseille-P5640	+	+	+
Bacteroides_bouchesdurhonensis	+	+	+
Bacillus_luciferensis	+	+	+
Ruminococcaceae_bacterium_AB4001	+	+	+
Treponema_saccharophilum	+	+	+
Prevotella_sp._ne3005	+	+	+
Clostridium_sp._CAG:306	+	+	+
Brachyspira_pilosicoli	+	+	+
Paramaledivibacter_caminithermalis	+	+	+
Bacteroides_sp._CAG:443	+	+	+
Blautia_sp._AF19-10LB	+	+	+
Clostridium_sp._3-3	+	+	+
Vibrio_phage_vB_VorS-PVo5	+	+	+
Bacillus_sp._FJAT-21351	+	+	+
Syntrophorhabdus_sp._PtaU1.Bin002	+	+	+
Lactobacillus_taiwanensis	+	+	+
Flavonifractor_sp._An52	+	+	+
Maledivibacter_halophilus	+	+	+
Clostridium_felsineum	+	+	+
Bacillus_tequilensis	+	+	+
Clostridium_sp._CAG:354_28_25	+	+	+
Fusobacterium_perfoetens	+	+	+
Lachnospiraceae_bacterium_AC2028	+	+	+
Clostridium_sp._AF20-17LB	+	+	+
Geobacillus_virus_E3	+	+	+
Clostridium_diolis	+	+	+
Gabonibacter_massiliensis	+	+	+
Desulfovibrio_termitidis	+	+	+
Helicobacter_sp._MIT_11-5569	+	+	+
Clostridium_sp._CAG:273	+	+	+
uncultured_bacterium_Contig1450	+	+	+
Gabonia_massiliensis	+	+	+
Paenibacillus_sp._Soil787	+	+	+
Clostridium_minihomine	+	+	+
Syntrophomonas_wolfei	+	+	+
Treponema_denticola	+	+	+
Prevotella_sp._S7-1-8	+	+	+
Brevibacillus_phage_Sundance	+	+	+
Mason-Pfizer_monkey_virus	+	+	+
Coprococcus_sp._AF38-1	+	+	+
Bacteroides_sp._AM22-3LB	+	+	+
Clostridium_puniceum	+	+	+
Azospirillum_sp._CAG:260	+	+	+
Tepidibacter_thalassicus	+	+	+
Bacillus_glycinifermentans	+	+	+
Sporomusa_sphaeroides	+	+	+
Geminocystis_sp._NIES-3708	+	+	+
Tepidanaerobacter_acetatoxydans	+	+	+
Bacteroides_sp._An51A	+	+	+
Firmicutes_bacterium_HGW-Firmicutes-3	+	+	+
Lachnospiraceae_bacterium_6_1_37FAA	+	+	+
Prevotella_sp._109	+	+	+

Lachnospiraceae_bacterium_JC7	+	+	+
Ruminococcus_sp._AF17-12	+	+	+
Bacteroides_sp._AF25-38AC	+	+	+
Proteiniphilum_sp._X52	+	+	+
Bacteroides_zoogleoformans	+	+	+
Hungateiclostridium_sp._N2K1	+	+	+
Bacteroides_sp._AM16-24	+	+	+
Azospirillum_sp._51_20	+	+	+
Streptococcus_macedonicus	+	+	+
Streptococcus_pneumoniae	+	+	+
Pseudoflavonifractor_sp._An176	+	+	+
Ruminococcus_sp._CAG:382	+	+	+
Clostridium_sp._CAG:710	+	+	+
Lachnospiraceae_bacterium_6_1_63FAA	+	+	+
Eubacterium_sp._AF18-3	+	+	+
Ruminococcus_sp._OM02-16LB	+	+	+
Peptoniphilus_duerdenii	+	+	+
Acetitomaculum_ruminis	+	+	+
Ruminococcus_sp._DSM_100440	+	+	+
Bacteroides_sp._CAG:598	+	+	+
Clostridiales_bacterium_38-18	+	+	+
Ruminococcus_sp._AM27-16	+	+	+
Cloacibacillus_evryensis	+	+	+
Enorma_phocaeensis	+	+	+
Methanocaldococcus_bathoardescens	+	+	+
Geosporobacter_subterraneus	+	+	+
Ruminococcus_sp._CAG:403	+	+	+
Acetoanaerobium_noterae	+	+	+
Campylobacter_geochelonis	+	+	+
Prevotella_disiens	+	+	+
Propionispira_arboris	+	+	+
Clostridium_sp._Maddingley_MBC34-26	+	+	+
Campylobacter_lari	+	+	+
Dorea_sp._42_8	+	+	+
Lachnospiraceae_bacterium_8_1_57FAA	+	+	+
Ornithobacterium_rhinotracheale	+	+	+
Sporolactobacillus_laevolacticus	+	+	+
Prevotella_paludivivens	+	+	+
Bacteroides_sp._AM23-12	+	+	+
Acetobacterium_sp._KB-1	+	+	+
uncultured_Caudovirales_phage	+	+	+
Ezakiella_peruensis	+	+	+
Clostridium_sp._26_22	+	+	+
Cecembia_calidifontis	+	+	+
Pirellula_sp._SH-Sr6A	+	+	+
Prevotella_albensis	+	+	+
Roseburia_sp._CAG:197_41_10	+	+	+
Bacteroides_sp._AF14-46	+	+	+
Staphylococcus_pseudintermedius	+	+	+
Megasphaera_cerevisiae	+	+	+
Paenibacillus_pinihumi	+	+	+
Desulfomicrobium_norvegicum	+	+	+
Parabacteroides_sp._AF19-14	+	+	+
Bacteroides_sp._AF20-13LB	+	+	+
Lachnospiraceae_bacterium_Choco86	+	+	+

Porphyromonas_gingivalis	+	+	+
Streptococcus_anginosus	+	+	+
Cetobacterium_ceti	+	+	+
Clostridiales_bacterium_43-6	+	+	+
Firmicutes_bacterium_HGW-Firmicutes-7	+	+	+
Clostridium_sp._AM30-24	+	+	+
Proteiniphilum_acetatigenes	+	+	+
Selenomonas_sp._ND2010	+	+	+
Roseburia_sp._CAG:45	+	+	+
Bacillus_phage_vB_BpuM-BpSp	+	+	+
Butyrivibrio_sp._XB500-5	+	+	+
Prevotella_dentasini	+	+	+
Clostridium_sp._CAG:354	+	+	+
Clostridium_sp._N3C	+	+	+
Leptotrichia_trevisanii	+	+	+
Anaerostipes_sp._AF04-45	+	+	+
Enterococcus_hirae	+	+	+
Prevotella_aff._ruminicola_Tc2-24	+	+	+
Dorea_sp._OM07-5	+	+	+
Faecalibacterium_sp._AF28-13AC	+	+	+
Acetobacterium_sp._MES1	+	+	+
Adlercreutzia_equolifaciens	+	+	+
Candidatus_Fischerbacteria_bacterium_RBG_13_37_8	+	+	+
Clostridium_sp._AF28-12	+	+	+
Blautia_sp._AF34-10	+	+	+
Clostridium_sp._L2-50	+	+	+
Clostridium_sp._CAG:343	+	+	+
Treponema_pedis	+	+	+
Desulfotomaculum_sp._46_80	+	+	+
Tepidibacter_formicigenes	+	+	+
Dethiosulfatibacter_aminovorans	+	+	+
Yersinia_phage_phiR1-37	+	+	+
Bacillus_phage_PBC2	+	+	+
Lactobacillus_rhamnosus	-	-	+
Listeria_costaricensis	+	+	+
Lachnospiraceae_bacterium_NC2008	+	+	+
Faecalibacterium_sp._OF03-6AC	+	+	+
Tidjanibacter_massiliensis	+	+	+
Propionispora_vibrioides	+	+	+
Eubacterium_sp._CAG:786	+	+	+
Defluviitalea_phaphyphila	+	+	+
Bacteroides_sp._D2	+	+	+
Bacteroides_sp._2_1_33B	+	+	+
Prevotella_sp._P4-51	+	+	+
Bacillus_coagulans	+	+	+
Fontibacillus_phaseoli	+	+	+
Candidatus_Gastranaerophilales_bacterium	+	+	+
Prevotella_lascolaii	+	+	+
Subdoligranulum_sp._AM23-21AC	+	+	+
Bacteroides_sp._3_1_33FAA	+	+	+
Desulfovibrio_sp._OH1209_COT-279	+	+	+
Candidatus_Muproteobacteria_bacterium_RIFCSPHIGHO2_01_	+	+	+
Deltaproteobacteria_bacterium_HGW-Deltaproteobacteria-8	+	+	+
Prevotella_sp._BP1-148	+	+	+
Clostridium_frigidicarnis	+	+	+

Anaerobiospirillum_thomasii	+	+	+
Sphaerochaeta_pleomorpha	+	+	+
Coprobacillus_sp._CAG:826	+	+	+
Tannerella_sp._oral_taxon_HOT-286	+	+	+
Desulfitobacterium_dichloroeliminans	+	+	+
Eubacterium_maltosivorans	+	+	+
Anaerovirgula_multivorans	+	+	+
Butyrivibrio_sp._XPD2006	+	+	+
Bacteroidetes_bacterium_CG2_30_32_10	+	+	+
archaeon	+	+	+
Clostridiales_bacterium_VE202-14	+	+	+
Bacteroides_uniformis_CAG:3	+	+	+
Bacteroidetes_bacterium_GWA2_40_14	+	+	+
Streptococcus_gallolyticus	+	+	+
Desulfosporosinus_sp._FKA	+	+	+
Klebsiella_pneumoniae	+	+	+
Ruminococcus_sp._AF19-29	+	+	+
Bacteroides_sp._AM16-13	+	+	+
Ruminococcus_sp._AF21-3	+	+	+
bacterium_F083	+	+	+
Desulfovibrio_africanus	+	+	+
Acetoanaerobium_sticklandii	+	+	+
Acetobacterium_wieringae	+	+	+
Inoviridae_sp.	+	+	+
Helicobacter_rappini	+	+	+
Cellulosilyticum_ruminicola	+	+	+
Phycicoccus_cremeus	+	+	+
Clostridium_sp._CAG:245_30_32	+	+	+
Clostridium_aurantibutyricum	+	+	+
Clostridium_sp._OM04-7	+	+	+
Ruminococcus_sp._AM29-26	+	+	+
Cohnella_sp._HS21	+	+	+
Prevotella_colorans	+	+	+
Prevotella_nanceiensis	+	+	+
Enterococcus_gallinarum	+	+	+
Faecalibacterium_sp._OF04-11AC	+	+	+
Clostridiales_bacterium_PH28_bin88	+	+	+
Clostridium_acetireducens	+	+	+
Clostridiales_bacterium_mt7	+	+	+
Firmicutes_bacterium_CAG:110_56_8	+	+	+
Clostridium_sp._CAG:91	+	+	+
Bacteroides_sp._An19	+	+	+
Lactobacillus_delbrueckii	+	+	+
Paenibacillus_massiliensis	+	+	+
Clostridium_clostridioforme_CAG:511	+	+	+
Ammoniphilus_oxalaticus	+	+	+
Helicobacter_aurati	+	+	+
Peptoclostridium_acidaminophilum	+	+	+
Desulfofarcimen_acetoxidans	+	+	+
Bacillus_sp._FJAT-27264	+	+	+
Pseudobutyrivibrio_sp._JW11	+	+	+
Firmicutes_bacterium_OM04-13BH	+	+	+
Clostridium_clostridioforme_CAG:132	+	+	+
Clostridium_sp._DL-VIII	+	+	+
Paenibacillus_phocaensis	+	+	+

Desulfobacteraceae_bacterium	+	+	+
Clostridium_formicaceticum	+	+	+
Paenibacillus_durus	+	+	+
Ruminococcus_sp._CAG:579	+	+	+
Coprobacillus_sp._CAG:698	+	+	+
Bacteroides_sp._D22	+	+	+
Ruminococcaceae_bacterium_AE2021	+	+	+
Clostridium_nigeriense	+	+	+
Chitinophaga_jiangningensis	+	+	+
Clostridium_sp._2-1	+	+	+
Methanosarcina_acetivorans	+	+	+
Prolixibacter_bellariivorans	+	+	+
Ruminococcus_sp._OM08-9BH	+	+	+
Sutterella_sp._CAG:397	+	+	+
Prevotella_sp._CAG:487	+	+	+
Clostridium_carboxidivorans	+	+	+
Acholeplasma_granularum	+	+	+
Desulfovibrio_putealis	+	+	+
Helicobacter_sp._MIT_99-5507	+	+	+
Parabacteroides_sp._HGS0025	+	+	+
Sphingobacteriales_bacterium	+	+	+
Petrimonas_mucosa	+	+	+
Clostridiales_bacterium_SYSU_GA17129	+	+	+
Eggerthella_sp._CAG:1427	+	+	+
Paenibacillus_sp._598K	+	+	+
Lactobacillus_aviarius	+	+	+
Brevibacillus_sp.	+	+	+
Clostridium_estertheticum	+	+	+
Slackia_heliotrinireducens	+	+	+
uncultured_bacterium_pUR16A2	+	+	+
Halanaerobium_saccharolyticum	+	+	+
Sporosarcina_newyorkensis	+	+	+
Desulfocucumis_palustris	+	+	+
Parabacteroides_sp._AF21-43	+	+	+
Ruminococcus_sp._OF03-6AA	+	+	+
Desulfovibrio_sp._TomC	+	+	+
Clostridium_sp._PYR-10	+	+	+
Desulfovibrio_fructosivorans	+	+	+
Proteiniborus_ethanoligenes	+	+	+
Brachyspira_hampsonii	+	+	+
Clostridium_isatidis	+	+	+
Faecalibacterium_phage_FP_Brigit	+	+	+
Methanocorpusculum_parvum	+	+	+
Ruminococcus_sp._AF14-10	+	+	+
Planctomycetes_bacterium	+	+	+
Clostridium_sp._CAG:452	+	+	+
Clostridium_sp._CAG:12237_41	+	+	+
Bacteroides_sp._CAG:1076	+	+	+
Blautia_sp._CAG:52	+	+	+
Mahella_australiensis	+	+	+
Paenibacillus_sp._LC231	+	+	+
Spirochaetae_bacterium_HGW-Spirochaetae-4	+	+	+
Caldanaerobius_polysaccharolyticus	+	+	+
Streptococcus_equinus	+	+	+
Blautia_sp._OF09-25XD	+	+	+

Clostridiaceae_bacterium_AF02-42	+	+	+
Paenibacillus_fonticola	+	+	+
Oribacterium_sinus	+	+	+
Raoultibacter_timonensis	+	+	+
Eubacterium_callanderi	+	+	+
Butyricimonas_sp._Marseille-P4593	+	+	+
Alkalibacter_saccharofermentans	+	+	+
Bacteroides_sp._AF37-16AC	+	+	+
Anaeromusa_acidaminophila	+	+	+
Bacteroidia_bacterium_44-10	+	+	+
Desulfallas_arcticus	+	+	+
Staphylococcus_aureus	+	+	+
Roseburia_sp._AF12-17LB	+	+	+
Lachnospiraceae_bacterium_AC2014	+	+	+
Paenibacillus_catalpae	+	+	+
Ruminococcus_sp._AF24-32LB	+	+	+
Desulfocurvus_vexinensis	+	+	+
Bacillus_sp._FJAT-18019	+	+	+
Roseburia_sp._AM51-8	+	+	+
Clostridiales_bacterium_mt11	+	+	+
Eubacterium_sp._CAG:86	+	+	+
Desulfotomaculum_ruminis	+	+	+
Prevotella_sp._AGR2160	+	+	+
Clostridium_roseum	+	+	+
Marininema_mesophilum	+	+	+
Candidatus_Gastranaerophilales_bacterium_HUM_9	+	+	+
Dysgonomonas_sp._Marseille-P4356	+	+	+
Paenibacillus_polymyxa	+	+	+
Halocella_sp._SP3-1	+	+	+
Desulfomicrobium_escambiense	+	+	+
Prevotella_sp._HUN102	+	+	+
Bacteroides_sp._AF18-33	+	+	+
Clostridium_sp._CAG:264	+	+	+
Acetobacter_sp._46_36	+	+	+
[Eubacterium]_yurii	+	+	+
Epulopiscium_sp._SCG-B05WGA-EpuloA1	+	+	+
Pseudodesulfovibrio_indicus	+	+	+
Turicimonas_muris	+	+	+
Clostridium_taeniosporum	+	+	+
uncultured_bacterium_LAB20	+	+	+
Fibrobacter_sp._UWS2	+	+	+
Prevotella_sp._P4-98	+	+	+
Prevotella_sp._lc2012	+	+	+
Bradymonadaceae_bacterium_TMQ3	+	+	+
Bacillus_licheniformis	+	+	+
Marinobacter_sp._B9-2	+	+	+
Nautilia_sp.	+	+	+
Bacteroides_galacturonicus	+	+	+
uncultured_spirochete	+	+	+
Paenibacillus_beijingensis	+	+	+
Geitlerinema_sp._PCC_7105	+	+	+
Tepidanaerobacter_syntrophicus	+	+	+
Bacteroides_sp._CF01-10NS	+	+	+
Ruminococcus_sp._SR1/5	+	+	+
Clostridium_sp._AM18-55	+	+	+

Desulfosporosinus_orientis	+	+	+
Macellibacteroides_sp._HH-ZS	+	+	+
Pelotomaculum_sp._PtaU1.Bin065	+	+	+
Nitrosospira_multiformis	+	+	+
Cellulosilyticum_sp._I15G10I2	+	+	+
Sphaerochaeta_halotolerans	+	+	+
Romboutsia_weinsteinii	+	+	+
Clostridium_luticellarii	+	+	+
Clostridium_sp._CAG:568	+	+	+
Johnsonella_ignava	+	+	+
Desulfotomaculum_sp._OF05-3	+	+	+
Phascolarctobacterium_sp._CAG:266	+	+	+
Clostridia_bacterium_UC5.1-2F7	+	+	+
Rhizobiales_bacterium	+	+	+
Clostridium_sp._AF21-20LB	+	+	+
Peptostreptococcus_sp._D1	+	+	+
Ruminiclostridium_cellulolyticum	+	+	+
Desulfitobacterium_chlororespirans	+	+	+
Tannerella_sp._AM09-19	+	+	+
Mageeibacillus_indolicus	+	+	+
Clostridium_tertium	+	+	+
Gorillibacterium_timonense	+	+	+
Desulfovibrio_sp._L21-Syr-AB	+	+	+
Clostridium_sp._CAG:352	+	+	+
Cellulomonas_carbonis	+	+	+
Clostridium_sp._AM22-16AC	+	+	+
Desulfovibrio_sp._A2	+	+	+
[Bacteroides]_coagulans	+	+	+
Clostridium_sp._CAG:138	+	+	+
Bacteroidetes_bacterium_HGW-Bacteroidetes-14	+	+	+
Eubacterium_sp._36_13	+	+	+
Desulfobulbus_sp.	+	+	+
Anoxybacillus_amylolyticus	+	+	+
Peptostreptococcaceae_bacterium_pGA-8	+	+	+
Bacteroidetes_bacterium_HGW-Bacteroidetes-10	+	+	+
Bacteroides_sp._AF04-22	+	+	+
Domibacillus_indicus	+	+	+
Moorea_producens	+	+	+
Atopobium_vaginae	+	+	+
Caloramator_sp._ALD01	+	+	+
Bacillus_farraginis	+	+	+
Thermoanaerobacterium_thermosaccharolyticum	+	+	+
Clostridium_aceticum	+	+	+
Veillonella_sp._CAG:933	+	+	+
Prevotella_sp._CAG:520	+	+	+
Myoviridae_sp.	+	+	+
Lachnospiraceae_bacterium_2_1_58FAA	+	+	+
Peptoniphilus_sp._KHD2	+	+	+
Clostridium_senegalense	+	+	+
Gordonibacter_massiliensis	+	+	+
Pseudobutyrivibrio_sp._OR37	+	+	+
Cohnella_sp._6021052837	+	+	+
Atopobacter_sp._AH10	+	+	+
Prevotella_buccae	+	+	+
Ruminococcus_sp._CAG:724	+	+	+

Absiella_sp._AM29-15	+	+	+
Firmicutes_bacterium_CAG:341	+	+	+
Prevotella_multiformis	+	+	+
Treponema_porcinum	+	+	+
Trichococcus_patagoniensis	+	+	+
Dickeya_sp._NCPPB_569	-	-	+
Mycobacteroides_abscessus	+	+	+
Clostridium_homopropionicum	+	+	+
Dysgonomonas_alginatilytica	+	+	+
Paenibacillus_sp._FSL_R7-0337	+	+	+
Desulfosporosinus_meridiei	+	+	+
Firmicutes_bacterium_CAG:176_59_8	+	+	+
Prevotella_marshii	+	+	+
Veillonellaceae_bacterium_DNF00751	+	+	+
Bacteroides_sp._AR20	+	+	+
Marinifilum_fragile	+	+	+
Firmicutes_bacterium_CAG:345	+	+	+
Peptococcaceae_bacterium_BRH_c8a	+	+	+
uncultured_microorganism	+	+	+
Parabacteroides_sp._AF39-10AC	+	+	+
Brachyspira_innocens	+	+	+
Clostridium_ragsdalei	+	+	+
Prevotella_baroniae	+	+	+
Acidaminococcus_fermentans	+	+	+
Olsenella_uli	+	+	+
Firmicutes_bacterium_CAG:822	+	+	+
Roseburia_sp._CAG:18_43_25	+	+	+
Mesorhizobium_sp._M4B.F.Ca.ET.019.03.1.1	+	+	+
Ruminococcus_sp._AM30-15AC	+	+	+
Prevotella_sp._P5-126	+	+	+
Eubacterium_sp._CAG:581	+	+	+
Fermentimonas_caenicola	+	+	+
Candidatus_Saccharimonas_sp.	+	+	+
Desulfobulbus_elongatus	-	+	+
Lachnospiraceae_bacterium_A10	+	+	+
Ruminococcus_sp._OF02-6	+	+	+
Bacteroides_coprophilus_CAG:333	+	+	+
Pelotomaculum_sp._PtaB.Bin104	+	+	+
Prevotella_oralis	+	+	+
Clostridium_sp._CAG:567	+	+	+
Blautia_sp._AF22-5LB	+	+	+
Clostridiales_bacterium_GWC2_40_7	+	+	+
Alkaliphilus_metalliredigens	+	+	+
Clostridium_amylolyticum	+	+	+
Paenibacillus_sp._FSL_R7-0333	+	+	+
Prevotella_pleuritidis	+	+	+
Ruminococcus_sp._YE78	+	+	+
Megasphaera_sp._ASD88	+	+	+
Coprobacter_secundus	+	+	+
Ruminococcaceae_bacterium_CPB6	+	+	+
Bacillus_phage_SP-15	+	+	+
Fusobacterium_sp._CM1	+	+	+
Sediminispirochaeta_smaragdinae	+	+	+
Acetonema_longum	+	+	+
Roseburia_sp._CAG:380	+	+	+

Alkalitalea_saponilacus	+	+	+
Campylobacter_concisus	+	+	+
Bifidobacterium_pseudocatenulatum	+	+	+
Clostridium_sp._AF29-8BH	+	+	+
Clostridium_sp._CAG:265	+	+	+
Bacteroidetes_bacterium_GWE2_40_15	-	+	+
Coprothermobacter_platensis	+	+	+
Anaerovibrio_sp._JC8	+	+	+
Oxalobacteraceae_bacterium_AB_14	+	+	+
Clostridium_novyi	+	+	+
Paenibacillus_tianmuensis	+	+	+
Lachnospiraceae_bacterium_TM07-2AC	+	+	+
Porcine_type-C_oncovirus	+	+	+
Paenibacillus_sp._IHB_B_3415	+	+	+
Proteiniborus_sp._DW1	+	+	+
Coprobacillus_sp._AF29-3BH	+	+	+
Paenibacillus_sp._7523-1	+	+	+
Clostridium_arbusti	+	+	+
Tannerella_sp._CAG:118	+	+	+
Candidatus_Nomurabacteria_bacterium_RIFCSPHIGHO2_12_F	-	+	+
Burkholderiales_bacterium_1_1_47	+	+	+
Candidatus_Nealsonbacteria_bacterium	+	+	+
Peptostreptococcaceae_bacterium_oral_taxon_113	+	+	+
Clostridium_tetanomorphum	+	+	+
Parabacteroides_chartae	+	+	+
Clostridium_sp._CAG:440	+	+	+
Firmicutes_bacterium_M10-2	+	+	+
[Clostridium]_spiroforme	+	+	+
Mangrovibacterium_marinum	+	+	+
Dorea_sp._CAG:105	+	+	+
Butyricimonas_faecalis	+	+	+
Paenibacillus_paeoniae	+	+	+
Flavobacterium_sp._LM4	+	+	+
Clostridium_grantii	+	+	+
Megasphaera_micronuciformis	+	+	+
Bittarella_massiliensis	+	+	+
Pseudomonas_syringae	+	+	+
Peptoclostridium_sp._AF21-18	+	+	+
Bacteroidales_bacterium_Barb6	+	+	+
bacterium_P201	+	+	+
Bacillus_cytotoxicus	+	+	+
Hymenobacter_chitinivorans	+	+	+
Hippea_jasoniae	+	+	+
Bacillus_caseinilyticus	+	+	+
Bifidobacterium_merycicum	+	+	+
Bacillus_phage_SP-10	+	+	+
Butyricicoccus_sp._AM27-36	+	+	+
Prevotella_sp._P5-108	+	+	+
Enterococcus_termitis	+	+	+
Anaerostipes_sp._CAG:276	-	-	+
Proteiniphilum_saccharofermentans	+	+	+
Massiliomicrobiota_sp._An105	+	+	+
Caldanaerobius_fijiensis	+	+	+
Fibrobacter_sp._UWH8	+	+	+
Bacteroidetes_bacterium_HGW-Bacteroidetes-1	+	+	+

Paenibacillus_sp._MY03	+	+	+
Anaeromassilibacillus_sp._Marseille-P3876	+	+	+
Clostridium_sp._CAG:389	+	+	+
Azospirillum_sp._CAG:239	+	+	+
Ruminococcus_sp._CAG:330	+	+	+
Roseburia_sp._AF34-16	+	+	+
Collinsella_provencensis	+	+	+
Paenibacillus_algorifonticola	+	+	+
Candidatus_Magnetomorum_sp._HK-1	+	+	+
Paenibacillus_sanguinis	+	+	+
Clostridium_sp._27_14	+	+	+
Clostridiaceae_bacterium_TF01-6	+	+	+
Prevotella_sp._AG:487_50_53	+	+	+
Bacteroidetes_bacterium_HGW-Bacteroidetes-7	+	+	+
Selenomonas_sp._Marseille-P3560	+	+	+
Paenibacillus_assamensis	+	+	+
Clostridium_sp._CAG:470	+	+	+
Candidatus_Margulisbacteria_bacterium_GWF2_38_17	+	+	+
Butyrivibrio_sp._NC2007	+	+	+
Clostridium_cellulovorans	+	+	+
Ruminococcus_sp._OM08-7	+	+	+
Erysipelatoclostridium_sp._An173	+	+	+
Allobaculum_stercoricanis	+	+	+
Cloacibacillus_porcorum	+	+	+
Tannerella_sp._AF04-6	+	+	+
Paenibacillus_sp._N2SHLJ1	+	+	+
Sediminibacillus_albus	+	+	+
Clostridium_sp._AF43-10	+	+	+
Campylobacter_curvus	+	+	+
Golden_hamster_intracisternal_A-particle_H18	+	+	+
Drancourtella_massiliensis	+	+	+
Prevotella_pallens	+	+	+
Paraclostridium_bifermentans	+	+	+
Eubacterium_sp._AF36-5BH	+	+	+
Bacteroidetes_bacterium_oral_taxon_272	+	+	+
Gemella_haemolysans	+	+	+
Geobacteraceae_bacterium_GWC2_53_11	+	+	+
Clostridium_sp._CAG:492	+	+	+
Desulfovibrio_magneticus	+	+	+
Parabacteroides_bouchesdurhonensis	+	+	+
Clostridium_sp._ATCC_29733	+	+	+
Butyrivibrio_sp._VCB2001	+	+	+
Eubacterium_sp._AM28-29	+	+	+
Phoenicibacter_massiliensis	+	+	+
Erwinia_phage_vB_EamP-L1	-	-	+
Fretibacterium_fastidiosum	+	+	+
Psychrobacillus_sp._FJAT-21963	+	+	+
Deltaproteobacteria_bacterium_HGW-Deltaproteobacteria-18	+	+	+
Kangiella_spongicola	+	+	+
Butyricicoccus_sp._AF22-28AC	+	+	+
Dysgonomonas_macrotermitis	+	+	+
Paenibacillus_physcomitrellae	+	+	+
Sphingobacterium_wenxiniae	+	+	+
Ruminococcus_sp._AM46-18	+	+	+
Ruminococcus_sp._AF16-50	+	+	+

Brevibacillus_brevis	+	+	+
Desulfonatronum_thioautotrophicum	+	+	+
Clostridium_sp._CAG:508	+	+	+
Prevotella_sp._TF12-30	+	+	+
Clostridium_sp._OF09-10	+	+	+
Bacteroides_sp._CAG:189	+	+	+
Acetobacterium_dehalogenans	+	+	+
Candidatus_Gracilibacteria_bacterium	+	+	+
Butyrivibrio_sp._AE3006	+	+	+
Eggerthella_sp._AM16-19	+	+	+
Lachnospiraceae_bacterium_OF11-28	+	+	+
Paenibacillus_donghaensis	+	+	+
Nocardia_asiatica	+	+	+
Desnuesiella_massiliensis	+	+	+
Theionarchaea_archaeon_DG-70-1	+	+	+
Fastidiosipila_sanguinis	+	+	+
Eubacterium_sp._AB3007	+	+	+
Lachnospiraceae_bacterium_TF01-11	+	+	+
Spirochaetae_bacterium_HGW-Spirochaetae-8	+	+	+
Sphaerochaeta_sp.	+	+	+
Desulfosporosinus_sp._Tol-M	+	+	+
Firmicutes_bacterium_CAG:41	+	+	+
Bacteroides_sp._OM08-17BH	+	+	+
Bifidobacterium_dentium	+	+	+
Roseburia_sp._AF02-12	+	+	+
Chitinophagaceae_bacterium	+	+	+
Paenibacillus_macerans	+	+	+
Clostridium_magnum	+	+	+
Salmonella_enterica	+	+	+
Pseudobutyrivibrio_sp._NOR37	+	+	+
Parascardovia_denticolens	+	+	+
Prevotella_denticola	+	+	+
Bacillus_sp._AFS001701	+	+	+
bacterium_336/3	+	+	+
Eggerthella_sp._1_3_56FAA	+	+	+
Merdibacter_massiliensis	+	+	+
Clostridium_sp._CAG:242	+	+	+
Fibrobacter_sp._UWCM	+	+	+
Eggerthia_catenaformis	+	+	+
Streptomyces_sp._150FB	+	+	+
Campylobacter_sp._73/13	+	+	+
Eubacterium_sp._CAG:146	+	+	+
Clostridium_sp._CAG:269	+	+	+
Desulfosporosinus_acidiphilus	+	+	+
Clostridium_sp._CAG:75	+	+	+
Ruminococcus_sp._AM16-34	+	+	+
Desulfovibrio_carbinolicus	+	+	+
Desulfovibrio_oxyclinae	+	+	+
Prevotellaceae_bacterium_MN60	+	+	+
Pseudobutyrivibrio_sp._UC1225	+	+	+
Streptococcus_pyogenes	+	+	+
Bat_gammaretrovirus	+	+	+
uncultured_Aquificaceae_bacterium	+	+	+
Porphyromonas_loveana	+	+	+
Desulfatibacillum_alkenivorans	+	+	+

Fusobacterium_russii	+	+	+
Denitrovibrio_sp.	+	+	+
Flexistipes_sinusarabici	+	+	+
Coprococcus_sp._OM04-5BH	+	+	+
Helicobacter_sp._16-1353	+	+	+
Clostridium_cochlearium	+	+	+
Cohnella_phaseoli	+	+	+
Coprobacter_fastidiosus	+	+	+
Nocardioides_sp._SZ4R5S7	+	+	+
Anaerosphaera_aminiphila	+	+	+
Prevotella_oulorum	+	+	+
Clostridium_liquoris	+	+	+
Enterococcus_raffinosus	+	+	+
Clostridiales_bacterium_VE202-08	+	+	+
Arthrobacter_sp._SX1312	+	+	+
Butyrivibrio_sp._AD3002	+	+	+
Pyramidobacter_sp._CG50-2	+	+	+
Staphylococcus_cohnii	+	+	+
Blautia_sp._TM10-2	+	+	+
Sediminispirochaeta_bajacaliforniensis	+	+	+
Paenibacillus_daejeonensis	+	+	+
Nitrospiraceae_bacterium	+	+	+
Clostridium_sp._CAG:594	+	+	+
Snodgrassella_alvi	+	+	+
Clostridium_tunisiense	+	+	+
Paenibacillus_sp._Soil750	+	+	+
Ruminococcus_sp._AF14-5	+	+	+
Flavobacterium_daejeonense	+	+	+
Arcobacter_ebronensis	+	+	+
Parabacteroides_sp._AF17-3	+	+	+
Jeotgalibaca_dankookensis	+	+	+
Paenibacillus_silvae	+	+	+
Clostridiales_bacterium_38_11	+	+	+
Pyramidobacter_piscolens	+	+	+
Moorella_thermoacetica	+	+	+
Gelatoporia_subvermispora	+	+	+
Gracilibacter_sp._BRH_c7a	+	+	+
Sporanaerobacter_sp._PP17-6a	+	+	+
Paenibacillus_sp._32O-W	+	+	+
Thermosyntropha_lipolytica	+	+	+
Bacteroides_sp._CAG:661	+	+	+
Blautia_sp._AM16-16B	+	+	+
Natronincola_peptidivorans	+	+	+
Tenericutes_bacterium_GWF2_57_13	+	+	+
Dorea_sp._AM10-31	+	+	+
Oribacterium_sp._oral_taxon_108	+	+	+
Clostridium_bartlettii_CAG:1329	+	+	+
Ruminococcus_sp._TF06-23	+	+	+
Muribaculaceae_bacterium_Isolate-077_(Janvier)	+	+	+
Armatimonadetes_bacterium_CG2_30_66_41	+	+	+
Rickettsiales_bacterium_TMED127	-	-	+
Bacillus_subtilis	+	+	+
Megamonas_funiformis	+	+	+
Acidaminobacter_hydrogenoformans	+	+	+
Prevotella_scopos	+	+	+

Bacteroides_sp._AF27-10BH	+	+	+
Enorma_massiliensis	+	+	+
Clostridium_tagluense	+	+	+
Clostridium_colicanis	+	+	+
Desulfonispora_thiosulfatigenes	+	+	+
Mogibacterium_sp._CM50	-	+	+
Thiotrichaceae_bacterium	-	-	+
Clostridium_sp._CL-2	+	+	+
uncultured_bacterium_Lq_025_E06	+	+	+
Porphyromonas_cangingivalis	+	+	+
Paenibacillus_sp._YN15	+	+	+
Prevotella_sp._KCOM_3155	+	+	+
Bacteroidetes_oral_taxon_274	+	+	+
Desulfotomaculum_aeronauticum	+	+	+
Clostridium_autoethanogenum	+	+	+
Peptococcaceae_bacterium_BICA1-8	+	+	+
Lachnoanaerobaculum_sp._OBRC5-5	+	+	+
Helicobacter_sp._MIT_17-337	+	+	+
Clostridium_sp._AM48-13	+	+	+
uncultured_bacterium_fosmid_pJB65E1	+	+	+
Acidobacterium_capsulatum	+	+	+
Candidatus_Methanomassiliicoccus_intestinalis	+	+	+
Heliobacterium_modesticaldum	+	+	+
Bacillus_wiedmannii	+	+	+
Clostridium_sp._CAG:780	+	+	+
Ruminococcus_sp._AF25-19	+	+	+
Proteiniclasticum_ruminis	+	+	+
Clostridiales_bacterium_GWD2_32_59	+	+	+
Clostridium_sp._CAG:575	+	+	+
Carboxydocella_sp._JDF658	+	+	+
Olsenella_sp._oral_taxon_807	+	+	+
Solobacterium_sp._Marseille-P4301	+	+	+
Prevotella_sp._AM23-5	+	+	+
Prevotella_sp._P5-60	+	+	+
Clostridium_neonatale	+	+	+
Prevotella_sp._MSX73	+	+	+
Candidatus_Vecturithrix_granuli	+	+	+
Halanaerobium_congolense	+	+	+
Eggerthella_timonensis	+	+	+
Subdoligranulum_sp._60_17	+	+	+
Acinetobacter_pittii	+	+	+
Sharpea_azabuensis	+	+	+
Clostridium_sp._AF37-5AT	+	+	+
Dysgonomonas_gadei	+	+	+
Prevotella_sp._oral_taxon_820	+	+	+
Paenibacillus_taihuensis	+	+	+
Gordonibacter_pamelaeae	+	+	+
Bacillus_sp._OK048	+	+	+
bacterium	+	+	+
Butyrivibrio_sp._XPD2002	+	+	+
Alkaliphilus_oremlandii	+	+	+
Bacillus_azotoformans	+	+	+
Desulfitobacterium_dehalogenans	+	+	+
Ruminococcus_sp._AF37-3AC	+	+	+
Cronobacter_sakazakii	+	+	+

Bacteroides_finegoldii_CAG:203	+	+	+
Desulfotomaculum_ferrireducens	+	+	+
Dehalococcoides_mccartyi	+	+	+
Tangfeifania_diversioriginum	+	+	+
Schwartzia_succinivorans	+	+	+
Firmicutes_bacterium_CAG:582	+	+	+
Clostridium_polynesiense	+	+	+
Blautia_sp._aa_0143	+	+	+
Collinsella_ihuae	+	+	+
Absiella_sp._AM27-20	+	+	+
Ruminococcus_sp._AM36-18	+	+	+
Clostridium_sp._OF13-4	+	+	+
Desulfosporosinus_acididurans	+	+	+
Khelaifiella_massiliensis	+	+	+
Methanosarcina_siciliae	+	+	+
Blautia_sp._AF26-2	+	+	+
Porphyromonas_canoris	+	+	+
Bacteroides_coprocola_CAG:162	+	+	+
Lucifera_butyrica	+	+	+
Marinilabilia_sp._WTE	+	+	+
Lachnospiraceae_bacterium_AM23-7LB	-	+	+
Bacillus_mycoides	+	+	+
Enterobacter_hormaechei	+	+	+
Prevotella_sp._CAG:924	+	+	+
Tepidimicrobium_xylanilyticum	+	+	+
Brevibacillus_fluminis	+	+	+
Prevotella_sp._khp7	+	+	+
Ignavibacteriales_bacterium	+	+	+
Mycolicibacterium_elephantis	+	+	+
Firmicutes_bacterium_CAG:240	+	+	+
[Eubacterium]_infirmum	+	+	+
Blautia_sp._AF19-34	+	+	+
Prevotella_sp._P6B4	+	+	+
Lachnospiraceae_bacterium_CAG:25	+	+	+
Thermanaeromonas_toyohensis	+	+	+
Subdoligranulum_sp._TF05-17AC	+	+	+
Bifidobacterium_pseudolongum	+	+	+
Brevibacillus_nitrificans	+	+	+
Fusobacterium_periodonticum	+	+	+
Prevotella_maculosa	+	+	+
Bacteroides_sp._CAG:702	+	+	+
Desulfohalotomaculum_alkaliphilum	+	+	+
Sphaerochaeta_dissipatitropha	+	+	+
Clostridium_sp._AF32-7AC	+	+	+
Shuttleworthia_satelles	+	+	+
Trichococcus_palustris	+	+	+
Acinetobacter_sp._CAG:196_36_41	+	+	+
Anaerorhabdus_furcosa	+	+	+
Erysipelatoclostridium_sp._An15	+	+	+
Prevotella_sp._P5-119	+	+	+
Desulfallas_geothermicus	+	+	+
Bacillus_sp._CAG:988	+	+	+
Bacteroidetes_bacterium_GWF2_35_48	+	+	+
Cryptobacterium_sp._CAG:338	+	+	+
Selenomonas_artemidis	+	+	+

Pseudosphingobacterium_domesticum	+	+	+
Bacteroides_sp._OF03-11BH	+	+	+
Porphyromonadaceae_bacterium_COT-184_OH4590	+	+	+
Synergistes_sp._3_1_syn1	+	+	+
Williamwhitmania_taraxaci	+	+	+
Thermoanaerobacter_thermohydrosulfuricus	+	+	+
Treponema_azotonutricium	+	+	+
Pseudobutyrivibrio_sp._C4	+	+	+
Lachnospiraceae_bacterium_5_1_63FAA	+	+	+
Selenomonas_sputigena	+	+	+
Clostridium_sp._CAG:793	+	+	+
Bifidobacterium_magnum	+	+	+
Pelolinea_submarina	+	+	+
Firmicutes_bacterium_HGW-Firmicutes-11	+	+	+
Parasutterella_excrementihominis	+	+	+
Oceanispirochaeta_sp._M1	+	+	+
Butyrivibrio_crossotus_CAG:259	+	+	+
Chitinophaga_niastensis	+	+	+
uncultured_bacterium_Contig39	+	+	+
Intestinibacter_bartlettii	+	+	+
Eubacterium_sp._CAG76_36_125	+	+	+
Bacteroides_sp._An279	+	+	+
Clostridium_sp._CAG:967	+	+	+
Clostridia_bacterium_UC5.1-1E11	+	+	+
Bacillus_asahii	+	+	+
Clostridiaceae_bacterium_MJB2	+	+	+
Peptoniphilus_sp._KHD4	+	+	+
Desulfurispora_thermophila	+	+	+
Streptococcus_caballi	+	+	+
uncultured_Coprococcus_sp.	+	+	+
Prevotella_sp._MA2016	+	+	+
Acholeplasma_equifetale	+	+	+
Lachnospiraceae_bacterium_MD2004	+	+	+
Muricauda_antarctica	+	+	+
Syntrophaceae_bacterium_PtaU1.Bin231	+	-	+
Clostridium_ljungdahlii	+	+	+
Fervidicella_metallireducens	+	+	+
Peptostreptococcus_russellii	+	+	+
Clostridium_kluyveri	+	+	+
Dehalobacter_sp._E1	+	+	+
Prevotella_dentalis	+	+	+
Bacteroides_faecis_CAG:32	+	+	+
Bacillus_massiliosenegalensis	+	+	+
Acholeplasma_palmae	+	+	+
Prevotella_shahii	+	+	+
Paenibacillus_ihumii	+	+	+
Bacteroides_cutis	+	+	+
uncultured_bacterium_Contig1522a	+	+	+
Macrococcus_caseolyticus	+	+	+
Brevibacillus_centrosporus	+	+	+
Caldiserica_bacterium	+	+	+
Arc_I_group_archaeon_U1lsi0528_Bin055	+	-	+
Lentisphaerae_bacterium_GWF2_52_8	+	+	+
Clostridium_sp._MSTE9	+	+	+
Burkholderia_pseudomallei	+	+	+

Syntrophobacter_sp._SbD1	+	+	+
Paenibacillus_jamilae	+	+	+
Eubacterium_sp._41_20	+	+	+
bacterium_1xD8-6	+	+	+
Ruminococcus_bicirculans	+	+	+
Carnobacterium_iners	+	+	+
Desulfomonile_tiedjei	+	+	+
Bifidobacterium_adolescentis	+	+	+
Butyricicoccus_sp._AF10-3	+	+	+
Bacteroidales_bacterium_36-12	+	+	+
Dysgonomonas_sp._Marseille-P4361	+	+	+
Raoultibacter_massiliensis	+	+	+
Sphingobacteriaceae_bacterium	+	+	+
Desulfovibrio_sp._U5L	+	+	+
Eisenibacter_elegans	-	+	+
Helicobacter_heilmannii	-	+	+
Brevibacillus_borstelensis	+	+	+
Squirrel_monkey_retrovirus	+	+	+
Clostridium_pasteurianum	+	+	+
Bacteroides_sp._AF39-10AT	+	+	+
Flavobacterium_columnare	+	+	+
Prevotella_sp._RM4	+	+	+
Porphyromonas_levii	+	+	+
Coprobacillus_sp._CAG:605	+	+	+
Erysipelotrichaceae_bacterium_5_2_54FAA	+	+	+
Enterococcus_haemoperoxidus	+	+	+
Aerococcus_viridans	+	+	+
Gordonibacter_sp._28C	+	+	+
Clostridium_septicum	+	+	+
Veillonella_sp.	+	+	+
Selenomonas_sp._oral_taxon_920	+	+	+
Capnocytophaga_sp._oral_taxon_863	+	+	+
Desulfovibrio_gigas	+	+	+
Ruminococcus_sp._AF26-25AA	+	+	+
Brevibacillus_reuszeri	+	+	+
Coprococcus_sp._TF11-13	+	+	+
Paenibacillus_peoriae	+	+	+
Coriobacteriaceae_bacterium_CHKCI002	+	+	+
Aneurinibacillus_migulanus	+	+	+
Streptococcus_cristatus	+	+	+
Paludibacter_sp._47-17	+	-	+
Slackia_equolifaciens	+	+	+
Porphyromonadaceae_bacterium_KH3CP3RA	+	+	+
Desulfosporosinus_sp._FKB	+	+	+
Candidatus_Bathyarchaeota_archaeon	+	+	+
Clostridiales_bacterium_VE202-09	+	+	+
Lactobacillus_plantarum	+	+	+
Clostridium_sp._ATCC_25772	+	+	+
Paenibacillus_baekrokdamisoli	+	+	+
Paenibacillus_lentus	+	+	+
Desulfosporosinus_sp._BRH_c37	+	+	+
Mobilibacterium_timonense	+	+	+
Prevotella_stercorea_CAG:629	+	+	+
uncultured_organism	+	+	+
Acholeplasma_axanthum	+	+	+

Enterobacteria_phage_vB_KleM-RaK2	+	+	+
Desulfovibrio_inopinatus	+	+	+
Desulfarculus_sp.	+	+	+
Lactobacillus_amylolyticus	+	+	+
Flavobacterium_sp._102	+	-	+
Dehalobacter_sp._MCB1	+	+	+
Prevotella_sp._oral_taxon_472	+	+	+
Clostridium_uliginosum	+	+	+
Bacteroidales_bacterium_KA00344	+	+	+
Pelodictyon_phaeoclathratiforme	+	+	+
Marinobacter_subterrani	+	+	+
Pseudomonas_putida	+	+	+
Prevotella_nigrescens	+	+	+
Paenibacillus_sp._P46E	+	+	+
Bacillus_simplex	+	+	+
Blautia_sp._SF-50	+	+	+
Desulfonatronovibrio_magnus	+	+	+
Fibrobacter_sp._UWH4	+	+	+
Faecalibacterium_sp._CAG:74	+	+	+
Clostridium_sp._CAG:277	+	+	+
Paenibacillus_pinisoli	+	+	+
Bacteroides_sp._AM07-16	+	+	+
Parcubacteria_bacterium_33_209	+	+	+
Pelotomaculum_sp._PtaU1.Bin035	+	+	+
Ruminococcus_sp._AM34-10LB	+	+	+
Sulfurospirillum_arsenophilum	-	-	+
Brachyspira_alvinipulli	+	+	+
Clostridium_sp._CAG:505	+	+	+
Ruminococcus_sp._AF43-11	+	+	+
Candidatus_Parcubacteria_bacterium	+	+	+
Fibrobacter_sp._UWEL	+	+	+
Bacteroides_sp._AM30-16	+	+	+
Butyricicoccus_sp._AM18-35	+	+	+
Labilibacter_marinus	+	+	+
Bacillus_sp._HMSC76G11	+	+	+
Syntrophus_gentianae	-	+	+
Ruminococcus_sp._OM07-7	+	+	+
Acidaminococcus_massiliensis	+	+	+
Xenorhabdus_bovienii	+	+	+
Firmicutes_bacterium_HGW-Firmicutes-8	+	+	+
Brevibacillus_panacihumi	+	+	+
Geobacter_sp._DSM_2909	+	+	+
Candidatus_Magasanikbacteria_bacterium_GW2011_GWC2_40	+	+	+
Candidatus_Altiarchaeales_archaeon_WOR_SM1_86-2	-	-	+
Proteobacteria_bacterium_CAG:495	+	+	+
Brachyspira_sp._G79	+	+	+
Clostridiales_bacterium_GWD2_32_19	+	+	+
Acidaminococcus_intestini	+	+	+
Saccharibacillus_sacchari	+	+	+
Butyrivibrio_sp._AE3003	+	+	+
Elusimicrobia_bacterium_GWA2_61_42	+	+	+
Desulfovibrio_gracilis	+	+	+
Syntrophorhabdus_sp._PtaB.Bin047	+	+	+
Pectobacterium_carotovorum	+	+	+
Oceanobacter_kriegii	-	+	+

Ruminococcus_sp._AF45-4BH	+	+	+
Pyramidobacter_sp._C12-8	+	+	+
Chryseobacterium_sp.	+	+	+
Prevotella_sp._DNF00663	+	+	+
Bacteroides_sp._CAG:98	+	+	+
Prevotella_sp._P6B1	+	+	+
Propionispora_sp._2/2-37	+	+	+
Ruminococcus_sp._CAG:17	+	+	+
Dehalobacter_sp._UNSWDHB	+	+	+
Arabia_massiliensis	+	+	+
Tenericutes_bacterium_HGW-Tenericutes-2	+	+	+
Bacillus_sp._P14.5	-	-	+
Dehalococcoidia_bacterium_CG2_30_46_19	-	-	+
Butyricicoccus_sp._AM32-19	+	+	+
Eubacterium_sp._AF22-9	+	+	+
Butyricicoccus_sp._GAM44	+	+	+
Lachnoanaerobaculum_sp._ICM7	+	+	+
Marinilabiliaceae_bacterium_JC017	+	+	+
Hymenobacter_sp.	+	+	+
Prevotella_sp._P4-67	+	+	+
Thermoanaerobacter_kivui	+	+	+
Bacillus_infantis	+	+	+
Blautia_massiliensis	+	+	+
Coprococcus_sp._OM06-34AC	+	+	+
Eubacterium_sp._TF12-12	+	+	+
Aquificae_bacterium	+	+	+
Thioalkalivibrio_nitratireducens	-	-	+
Clostridium_sp._CAG:349_48_7	+	+	+
Ruminococcus_sp._AF33-11BH	+	+	+
Fibrobacter_sp._UWB8	+	+	+
Butyrivibrio_sp._XBB1001	+	+	+
Clostridium_tepidiprofundi	+	+	+
Coprobacillus_sp._8_2_54BFAA	+	+	+
Faecalibacterium_sp._CAG:82	+	+	+
Epsilonproteobacteria_bacterium	+	+	+
Marinomonas_arctica	-	+	+
uncultured_Mediterranean_phage_uvMED	+	+	+
Thermophagus_xiamenensis	+	+	+
Miniphocibacter_massiliensis	+	+	+
Bacteroidales_bacterium_45-6	+	+	+
Paenibacillus_sp._1011MAR3C5	+	+	+
Paenibacillus_sp._32352	+	+	+
Thermovibrio_guaymasensis	+	+	+
Prevotella_fusca	+	+	+
Blautia_sp._AM47-4	+	+	+
Clostridium_sp._26_21	+	+	+
Clostridium_sp._HMP27	+	+	+
Proteocatella_sphenisci	+	+	+
Butyricicoccus_sp._OF10-2	+	+	+
Selenomonas_sp._oral_taxon_126	+	+	+
Ruminococcaceae_bacterium_YAD3003	+	+	+
Streptococcus_gordonii	+	+	+
Coprobacillus_sp.	+	+	+
Clostridium_sp._OM08-29	+	+	+
Ruminococcus_sp._AM42-10AC	+	+	+

Paenibacillus_curdlanolyticus	+	+	+
Porphyromonas_sp._COT-108_OH2963	+	+	+
Desulfomicrobium_baculatum	+	+	+
Arthrobacter_sp._MWB30	+	+	+
Desulfosarcina_sp._BuS5	+	+	+
Megasphaera_sp._An286	+	+	+
uncultured_murine_large_bowel_bacterium_BAC_54B	+	+	+
Capnocytophaga_stomatis	+	+	+
Spirochaetes_bacterium	+	+	+
Bacteroides_sp._AM37-9	+	+	+
Bacillus_massilioanorexius	+	+	+
Clostridium_sp._BNL1100	+	+	+
Tenericutes_bacterium_MZ-XQ	+	+	+
Prevotella_corporis	+	+	+
Rhodospirillaceae_bacterium_SYSU_D60007	-	-	+
Candidatus_Altiarchaeales_archaeon_HGW-Altiarchaeales-3	-	-	+
Campylobacter_ureolyticus	+	+	+
Devosia_geojensis	+	+	+
Dysgonomonas_mossii	+	+	+
Desulfallas_gibsoniae	+	+	+
Elizabethkingia_miricola	+	+	+
Paenibacillus_validus	+	+	+
Tannerella_sp._oral_taxon_808	+	+	+
Acetobacter_sp._CAG:267	+	+	+
Candidatus_Gastranaerophilales_bacterium_HUM_21	+	+	+
Bacteroidetes_bacterium_4572_117	+	+	+
Candidatus_Omnitrophica_bacterium_CG07_land_8_20_14_0_8	-	-	+
Pelosinus_propionicus	+	+	+
Aquiflexum_balticum	+	+	+
Bifidobacterium_saeculare	+	+	+
Eggerthella_sinensis	+	+	+
Enterococcus_columbae	+	+	+
Eikenella_corrodens	+	+	+
Ancylomarina_subtilis	+	+	+
Dethiosulfatarculus_sandiegensis	+	+	+
Chryseobacterium_daeguense	+	+	+
Aliifodinibius_sp._WN023	+	+	+
Campylobacter_sputorum	+	+	+
Geobacter_sp._M21	-	+	+
Morococcus_cerebrosus	-	-	+
Oceanimonas_smirnovii	-	-	+
Frischella_perrara	-	-	+
Mycobacterium_tuberculosis	+	+	+
Mycoplasma_bovirhinis	+	+	+
Desulfococcus_multivorans	+	+	+
Bacteroides_dorei_CAG:222	+	+	+
Fibrobacter_sp._UWB1	+	+	+
Proteus_mirabilis	-	+	+
Massilioclostridium_sp.	+	+	+
Achromobacter_xylosoxidans	+	+	+
Candidatus_Rokubacteria_bacterium	+	+	+
Lactobacillus_paracasei	+	+	+
Lutibacter_profundi	+	+	+
Bacillus_sp._XXST-01	+	+	+
Sporosarcina_sp._D27	-	+	+

Nitrosovibrio_tenuis	+	-	+
Gammaproteobacteria_bacterium_RIFOXYD12_FULL_61_37	-	-	+
Bartonella_queenslandensis	-	-	+
Rhizophagus_irregularis	+	+	+
Firmicutes_bacterium_HGW-Firmicutes-1	+	+	+
Flammeovirgaceae_bacterium_311	+	+	+
Tissierella_creatinophila	+	+	+
Bacteroides_eggerthii_CAG:109	+	+	+
Prevotella_enoeca	+	+	+
Ruminococcus_sp._AM33-14	+	+	+
Paenibacillus_sonchi	+	+	+
Nonlabens_arenilitoris	-	+	+
Parabacteroides_johnsonii_CAG:246	+	+	+
Paenibacillus_contaminans	+	+	+
Megasphaera_stantonii	+	+	+
Sutterella_wadsworthensis	+	+	+
Mycoplasma_sp._CAG:776	+	+	+
Clostridium_peptidivorans	+	+	+
Desulfobacteraceae_bacterium_4572_35.1	+	+	+
Streptococcus_parasanguinis	+	+	+
Vibrio_splendidus	+	+	+
Acinetobacter_sp._WCHAc060012	-	+	+
Paracoccus_solventivorans	-	-	+
Desulfofarcimen_intricatum	+	+	+
Streptococcus_mitis	+	+	+
Paenibacillus_sp._FJAT-27812	+	+	+
Verrucomicrobiaceae_bacterium	+	+	+
Desulfatirhabdium_butyrativorans	+	+	+
Ruminococcus_sp._5_1_39BFAA	+	+	+
Selenomonas_sp._F0473	+	+	+
Coprobacillus_sp._28_7	+	+	+
Desulfobacter_postgatei	+	+	+
Veillonella_parvula	-	+	+
Desulfonatronovibrio_hydrogenovorans	+	+	+
Candidatus_Methanoperedens_nitroreducens	+	+	+
Xenorhabdus_ishibashii	-	-	+
Campylobacter_sp._P0107	-	-	+
Eubacteriaceae_bacterium	+	+	+
Clostridium_drakei	+	+	+
Clostridium_cylindrosporum	+	+	+
Ruminococcus_sp._AM41-10BH	+	+	+
Fibrobacter_sp._UWB3	+	+	+
Bacteroidetes_bacterium_OLB11	+	+	+
Desulfosporosinus_sp._I2	+	+	+
Lachnospiraceae_bacterium_FD2005	+	+	+
Fibrobacter_succinogenes	+	+	+
Isobaculum_melis	+	+	+
Oceanobacillus_halophilus	+	+	+
Helicobacter_sp._MIT_05-5293	+	+	+
Spirochaetes_bacterium_GWC1_27_15	+	+	+
Chondromyces_crocatus	+	+	+
Cohnella_panacarvi	+	+	+
Candidatus_Melainabacteria_bacterium_RIFOXYA12_FULL_3	+	-	+
Halodesulfovibrio_aestuarii	+	-	+
Spirochaetes_bacterium_GWC2_52_13	+	+	+

Candidatus_Melainabacteria_bacterium_MEL.A1	+	+	+
Bordetella_hinzii	+	+	+
Eubacterium_sp._45_250	+	+	+
Bacillus_endophyticus	+	+	+
Roseofilum_reptotaenium	+	+	+
Bacillus_sp._URHB0009	+	+	+
Nakamurella_silvestris	+	+	+
Ignavibacteriales_bacterium_CG_4_9_14_3_um_filter_34_10	-	-	+
Butyricicoccus_sp._OM04-18BH	+	+	+
Brachyspira_hyodysenteriae	+	+	+
Clostridium_celatum	+	+	+
Sporolituus_thermophilus	+	+	+
Gorillibacterium_massiliense	+	+	+
Candidatus_Lambdaproteobacteria_bacterium_RIFOXYD2_FUI	+	+	+
Desulfovibrio_aminophilus	+	+	+
uncultured_Megasphaera_sp.	+	+	+
Thermincola_potens	+	+	+
Clostridiales_bacterium_GWB2_37_7	+	+	+
Prevotella_jejuni	+	+	+
Candidatus_Gastranaerophilales_bacterium_HUM_18	+	+	+
Paludibacter_jiangxiensis	+	+	+
Nonlabens_spongiae	+	+	+
Listeria_phage_LMSP-25	+	+	+
Pusillimonas_harenae	+	-	+
Ruminococcus_sp._CAG:254	+	+	+
Burkholderia_sp._Ch1-1	+	+	+
uncultured_bacterium_Contig140	+	+	+
Flavobacteria_bacterium_RIFCSPLOWO2_12_FULL_35_11	+	+	+
Budvicia_aquatica	-	+	+
Syntrophomonas_zehnderi	+	+	+
Prevotella_sp._C561	+	+	+
Moorella_humiferrea	+	+	+
Clostridium_gasigenes	+	+	+
Paenibacillus_oryzae	+	+	+
Moorella_sp._Hama-1	+	+	+
Caminicella_sporogenes	+	+	+
Nostoc_sp._'Lobaria_pulmonaria_(5183)_cyanobiont'	-	+	+
Blautia_sp._AF13-16	+	+	+
Thermoanaerobacteraceae_bacterium_SP2	+	+	+
Veillonella_sp._AF36-20BH	+	+	+
Blautia_sp._TF11-31AT	+	+	+
Thermoplasmatales_archaeon_BRNA1	+	+	+
Bacillus_nealsonii	+	+	+
Clostridium_sp._YH-panp20	+	+	+
Massiliomicrobiota_sp._An134	+	+	+
Senegalimassilia_anaerobia	+	+	+
Acholeplasma_oculi	+	+	+
Paenibacillus_sp._OV191	+	+	+
Lactobacillus_equicursoris	+	+	+
Lactobacillus_gasseri	+	+	+
Maricaulis_maris	-	-	+
Pectobacterium_parmentieri	-	-	+
Erwinia_billingiae	-	-	+
Burkholderiales_bacterium	+	+	+
Campylobacter_sp._RM8835	+	+	+

Sulfurovum_sp._AS07-7	+	+	+
Bifidobacterium_longum	+	+	+
Prevotella_histicola	+	+	+
Prevotella_loescheii	+	+	+
Finegoldia_magna	+	+	+
Ruminococcus_sp._AM26-12LB	+	+	+
Orientia_tsutsugamushi	+	+	+
Paenibacillus_tuaregi	+	+	+
Desulfotomaculum_hydrothermale	+	+	+
Coprothermobacter_proteolyticus	+	+	+
Clostridium_sp._44_14	+	+	+
Carnobacterium_jeotgali	+	+	+
Sutterella_parvirubra	+	+	+
Coprobacillus_sp._AF21-8LB	+	+	+
Clostridium_sp._28_17	+	+	+
Bradyrhizobium_sp._STM_3843	-	-	+
Turicibacter_sanguinis	+	+	+
Campylobacter_mucosalis	+	+	+
Geobacter_sp.	+	+	+
Ruminococcus_sp._CAG:90	-	+	+
Alkalibaculum_bacchi	+	+	+
Dendrosporobacter_quercicolus	+	+	+
Bacteroidetes_bacterium_HGW-Bacteroidetes-8	+	+	+
Prevotellaceae_bacterium_Marseille-P2826	+	+	+
Jiulongibacter_sediminis	+	+	+
bacterium_BMS3Abin03	+	+	+
Streptococcus_thermophilus	+	+	+
Clostridiaceae_bacterium_14S0207	+	+	+
Clostridium_sp._CAG:1219	+	+	+
Paenibacillus_harenae	+	+	+
Paenibacillus_barengoltzii	+	+	+
Lachnospiraceae_bacterium_1_1_57FAA	+	+	+
Pelosinus_fermentans	+	+	+
Mannheimia_varigena	-	+	+
Lactobacillus_sp.	+	+	+
Tumebacillus_avium	-	+	+
Legionella_nautarum	-	+	+
Desulfocapsa_sulfexigens	-	-	+
Maribius_salinus	-	-	+
Idiomarina_tyrosinivorans	-	-	+
Helicobacter_sp._MIT_01-3238	+	+	+
Arcobacter_trophiarum	+	+	+
Achromobacter_sp._2789STDY5608628	+	+	+
Clostridium_sp._JN-9	+	+	+
Eubacterium_sp._AM46-8	+	+	+
Dysgonomonas_sp._HGC4	+	+	+
uncultured_bacterium_IN-06	+	+	+
Sporanaerobacter_sp._NJN-17	+	+	+
Caldicoprobacter_oshimai	+	+	+
Clostridium_sp._CAG:557	+	+	+
Vagococcus_acidifermentans	+	+	+
Anaerobacillus_alkalilacustris	+	+	+
Bacillus_koreensis	+	+	+
Bacillus_sp._FJAT-45505	+	+	+
Atopobium_sp._oral_taxon_810	+	+	+

candidate_division_NC10_bacterium_RBG_16_65_8	+	+	+
Flavobacterium_sp._IMCC34759	+	+	+
uncultured_bacterium_scaffold00090	+	-	+
Streptococcus_constellatus	+	+	+
Epulopiscium_sp._SCG-C07WGA-EpuloA2	+	+	+
Patescibacteria_group_bacterium	+	+	+
Rhodovarius_sp._CCP-6	+	+	+
Peptostreptococcaceae_bacterium_VA2	+	+	+
Edwardsiella_tarda	+	+	+
Butyricicoccus_sp._AF35-5AC	+	+	+
Caloramator_australicus	+	+	+
Weissella_hellenica	+	+	+
Senegalia_massiliensis	+	+	+
Prevotella_ihumii	+	+	+
Romboutsia_timonensis	+	+	+
Mesotoga_sp._H07pep.5.4	-	+	+
Trichormus_sp._NMC-1	-	+	+
Desulfovibrio_ferrophilus	+	+	+
Desulfobacterales_bacterium_RIFOXYA12_FULL_46_15	+	-	+
Zooshikella_ganghwensis	-	-	+
Pseudomonas_aeruginosa	+	+	+
Treponema_putidum	+	+	+
Paenibacillus_elgii	+	+	+
Helicobacteraceae_bacterium_4484_230	+	+	+
Neocallimastix_californiae	+	+	+
Butyrivibrio_sp._FCS006	+	+	+
Lentisphaerae_bacterium_GWF2_50_93	+	+	+
Coprococcus_sp._CAG:782	+	+	+
Lactobacillus_pentosiphilus	+	+	+
Prevotella_micans	+	+	+
Paenibacillus_riograndensis	+	+	+
Clostridium_sp._JN-1	+	+	+
Clostridioides_mangenotii	+	+	+
Bacillus_alkalitelluris	+	+	+
Megamonas_rupellensis	+	+	+
Clostridium_sp._AM27-28	+	+	+
Thermoanaerobacterium_aotearoense	+	+	+
Lactobacillus_sanfranciscensis	+	+	+
Jeotgalibacillus_malaysiensis	+	+	+
Oceanobacillus_limi	+	-	+
Candidatus_Raymondbacteria_bacterium_RIFOXYA2_FULL_4	+	-	+
Helicobacter_equorum	-	-	+
bacterium_D16-56	+	+	+
Fusobacterium_sp._HMSC073F01	+	+	+
Campylobacter_lanienae	+	+	+
Campylobacter_showae	+	+	+
Fibrobacter_sp._UWT2	+	+	+
Blautia_sp._Marseille-P2398	+	+	+
Prevotella_sp._P5-125	+	+	+
Firmicutes_bacterium_ML8_F2	+	+	+
[Hallella]_seregens	+	+	+
Caloranaerobacter_sp._TR13	+	+	+
Porphyromonadaceae_bacterium_KH3R12	+	+	+
Clostridium_thermobutyricum	+	+	+
Bacillus_phage_BCD7	+	+	+

Massilia_niastensis	+	+	+
Delftia_sp._HK171	-	+	+
Bacillus_hisashii	+	-	+
[Haemophilus]_ducreyi	-	-	+
Candidatus_Electrothrix_marina	-	-	+
Pseudothermotoga_thermarum	+	+	+
Paenibacillus_sp._KS1	+	+	+
Arcobacter_cryaerophilus	+	+	+
Clostridium_amazonitimonense	+	+	+
Pacificimonas_flava	+	+	+
Hapalosiphon_sp._MRB220	+	+	+
Desulforegula_conservatrix	+	+	+
Bacteroides_caccae_CAG:21	+	+	+
Epulopiscium_sp._Nuni2H_MBin003	+	+	+
Clostridium_argentinense	+	+	+
Clostridium_sp._JN500901	+	+	+
Carboxydothermus_islandicus	+	+	+
Paenibacillus_sp._Marseille-P3787	+	+	+
Candidatus_Moranbacteria_bacterium_GW2011_GWE1_35_17	+	+	+
Roseburia_sp._AF25-13LB	+	+	+
Helicobacter_cetorum	-	+	+
Psychroflexus_sp._S27	-	-	+
Clostridium_sp._HMSC19B11	-	-	+
Firmicutes_bacterium_HGW-Firmicutes-21	+	+	+
Blautia_sp._AF19-13LB	+	+	+
Clostridium_sp._CAG:217	+	+	+
Candidatus_Arcanobacter_lacustris	+	+	+
Sporanaerobacter_acetigenes	+	+	+
Bacteroidetes_bacterium_GWF2_43_63	+	+	+
Anaerolineae_bacterium	+	+	+
Blautia_sp._AF25-12LB	+	+	+
Marinifilum_flexuosum	+	+	+
Geobacteraceae_bacterium_GWC2_58_44	+	+	+
Noviherbaspirillum_denitrificans	+	+	+
Bacillus_sp._SB49	+	+	+
Prevotella_sp._CAG:474	+	+	+
Leuconostoc_gelidum	+	+	+
Bacillus_sp._UNC322MFChir4.1	+	+	+
Yangia_sp._SAOS_153D	-	+	+
Candidatus_Melainabacteria_bacterium_RIFOXYA2_FULL_32	+	-	+
Deferribacter_desulfuricans	+	+	+
Pelobacter_acetylenicus	+	+	+
Xanthomonadaceae_bacterium_NML120232	+	+	+
Prevotella_sp._P5-64	+	+	+
Methylophaga_sulfidovorans	+	+	+
Prevotella_sp._S7_MS_2	+	+	+
Parabacteroides_sp._AM58-2XD	+	+	+
Faecalibacterium_sp._AF27-11BH	+	+	+
Bacillus_wakoensis	+	+	+
Vaginella_massiliensis	+	+	+
Bacillus_manliponensis	+	+	+
Enterococcus_sp._Gos25-1	+	+	+
Selenomonas_noxia	+	+	+
Bacillus_ginsengihumi	-	+	+
Salegentibacter_mishustinae	+	+	+

Neisseria_polysaccharea	+	+	+
Mitsuokella_sp._AF21-1AC	+	+	+
Vibrio_rotiferianus	+	+	+
Proteus_penneri	+	+	+
Helicobacter_canis	+	+	+
Anaeromyces_robustus	+	+	+
Desulfovibrio_longus	+	+	+
Methylophaga_sp.	+	+	+
Campylobacter_helveticus	+	+	+
Bacillus_sp._7586-K	+	+	+
Corynebacterium_sp._HMSC073D01	+	+	+
Prevotella_sp._AM34-19LB	+	+	+
Tenericutes_bacterium_HGW-Tenericutes-1	+	+	+
Eubacterium_sp._CAG:156	+	+	+
Lachnospiraceae_bacterium_AM40-2BH	+	+	+
Anaerosalibacter_sp._Marseille-P3206	+	+	+
Gillisia_sp._JM1	+	+	+
Firmicutes_bacterium_HGW-Firmicutes-14	+	+	+
Paenibacillus_pabuli	+	+	+
Thermacetogenium_phaeum	+	+	+
Bacteroides_sp._AF33-23	+	+	+
Paenibacillus_koleovorans	+	+	+
Roseburia_sp._CAG:50	+	+	+
delta_proteobacterium_NaphS2	+	-	+
Fusobacterium_necrogenes	+	-	+
Microbacterium_sp._CGR1	-	-	+
Delftia_acidovorans	-	-	+
Aliagarivorans_marinus	-	-	+
Arcobacter_halophilus	+	+	+
Eubacterium_sp._TM06-47	+	+	+
Thermoanaerobacterales_bacterium_SK-G1	+	+	+
Pedobacter_panaciterrae	+	+	+
Pseudoalteromonas_phage_Maelstrom	+	+	+
Marinobacter_sp._LV10R510-11A	+	+	+
Pelotomaculum_sp._PtaB.Bin117	+	+	+
Propionibacterium_sp._HMSC067A01	+	+	+
bacterium_endosymbiont_of_Escarpia_laminata	+	+	+
Prevotella_sp._Marseille-P4334	+	+	+
Deltaproteobacteria_bacterium_RIFOXYA12_FULL_58_15	-	+	+
Bacteroidetes_bacterium_GWF2_41_61	+	+	+
Porphyromonas_sp._CAG:1061	+	+	+
Roseburia_intestinalis_CAG:13	+	+	+
Roseburia_sp._AF25-18LB	+	+	+
Acholeplasma_hippikon	+	+	+
Methylomonas_koyamae	+	-	+
Bacillus_sp._7894-2	-	-	+
Candidatus_Raymondbacteria_bacterium_RifOxyA12_full_50_3	-	-	+
Polaromonas_sp._35-63-35	-	-	+
Yersiniaceae_bacterium_ZS-11	-	-	+
Chromatiaceae_bacterium_2141T.STBD.0c.01a	-	-	+
Veillonella_sp._VA141	-	-	+
Aliiarcobacter_faecis	+	+	+
Neisseria_lactamica	+	+	+
Bacillus_zeae	+	+	+
Caldanaerobacter_subterraneus	+	+	+

Lactobacillus_hordei	+	+	+
Lactobacillus_equi	+	+	+
Bacteroides_sp._3_1_23	+	+	+
Bacillus_sp._Marseille-P3661	+	+	+
Olsenella_sp._An290	+	+	+
Anaerosalibacter_massiliensis	+	+	+
Pluralibacter_gergoviae	+	+	+
Enterococcus_pallens	+	+	+
Bdellovibrionales_bacterium_RIFOXYB1_FULL_39_21	-	-	+
Microcystis_aeruginosa	+	+	+
candidate_division_KSB1_bacterium	+	+	+
Paenibacillus_amylolyticus	+	+	+
Varibaculum_timonense	+	+	+
[Arcobacter]_porcinus	+	+	+
Prolixibacteraceae_bacterium_XSD2	+	+	+
Arcobacter_anaerophilus	+	+	+
Fodinicurvata_fenggangensis	+	+	+
Achromobacter_piechaudii	+	+	+
Caldicellulosiruptor_kristjanssonii	+	+	+
Cellulophaga_virus_ST	+	+	+
Candidatus_Desulfosporosinus_infrequens	+	+	+
Paenibacillus_polysaccharolyticus	+	+	+
Acinetobacter_sp._SFD	-	+	+
Lactobacillus_paralimentarius	+	+	+
Gordonibacter_sp._An230	+	+	+
Azospirillum_sp._K2W22B-5	+	+	+
Ruminococcus_sp._AM36-17	+	+	+
Enterococcus_durans	+	+	+
Lactobacillus_paraplantarum	+	+	+
Desulfotignum_phosphitoxidans	+	-	+
Castellaniella_caeni	-	-	+
Methanomassiliicoccales_archaeon_PtaU1.Bin030	-	-	+
Methanohalophilus_portucalensis	+	+	+
Helicobacter_fennelliae	+	+	+
gamma_proteobacterium_WG36	+	+	+
Helicobacter_winghamensis	+	+	+
Dehalobacter_sp._FTH1	+	+	+
Candidatus_Levybacteria_bacterium_RIFCSPLOWO2_02_FUL	+	+	+
Bathymodiolus_thermophilus_thioautotrophic_gill_symbiont	+	+	+
Sphaerochaeta_globosa	+	+	+
Gammaproteobacteria_bacterium_RIFCSPHIGHO2_12_FULL_	-	+	+
Acholeplasma_sp.	+	+	+
Megasphaera_paucivorans	+	+	+
Proteobacteria_bacterium_CAG:139	+	+	+
Megasphaera_sp._AM44-1BH	+	+	+
Flexibacter_flexilis	+	+	+
Bacillus_sp._M6-12	+	+	+
Oceanobacillus_senegalensis	+	+	+
Inordinaticella_fortuita	+	+	+
Enterococcus_asini	+	+	+
Paenisporosarcina_sp._HGH0030	+	+	+
Bacillus_atrophaeus	+	+	+
Clostridium_sp._7_2_43FAA	+	+	+
Ignavibacteriales_bacterium_UTCHB2	-	+	+
Anaerolineales_bacterium	+	+	+

Streptococcus_acidominimus	+	+	+
Spiroplasma_corruscae	-	+	+
Lactobacillus_pontis	-	-	+
Cohnella_thermotolerans	+	+	+
Bacteroides_sp._AF17-1	-	+	+
Thiomonas_sp._CB3	+	+	+
Clostridium_sp._CAG:302	+	+	+
Moorella_mulderi	+	+	+
Thermoanaerobacter_mathranii	+	+	+
Caldicellulosiruptor_kronotskyensis	+	+	+
Paenibacillus_antibioticophila	+	+	+
Prevotella_sp._khp1	+	+	+
Chryseobacterium_sp._SCN_40-13	+	+	+
Selenomonas_sp._oral_taxon_138	+	+	+
Candidatus_Saccharimonas_aalborgensis	+	+	+
Clostridium_sp._KNHs214	+	+	+
Mycoplasma_sp._CAG:877	+	+	+
Erysipelotrichaceae_bacterium_SG0102	+	+	+
Streptococcus_infantis	+	+	+
Stenoxybacter_acetivorans	+	+	+
Bacillus_phage_PBC6	+	+	+
Faecalibaculum_rodentium	+	+	+
Bacillus_niameyensis	+	+	+
Streptococcus_himalayensis	+	+	+
Clostridium_acidisoli	+	+	+
Fibrobacter_sp._UWB5	+	+	+
Chryseobacterium_molle	+	+	+
Butyricicoccus_sp._AF15-40	+	+	+
Paenibacillus_glucanolyticus	+	+	+
Bacteroides_sp._CAG:20	+	+	+
Saccharicrinis_fermentans	+	+	+
Desulfovirgula_thermocuniculi	+	+	+
Bacteroidetes_bacterium_RIFCSPLOWO2_12_FULL_35_15	+	+	+
Streptococcus_pasteurianus	+	+	+
Thiohalocapsa_sp._ML1	-	+	+
Enteroscipio_rubneri	+	+	+
Halanaerobium_sp._ST460_2HS_T2	+	+	+
Methanohalophilus_sp._RSK	-	-	+
Idiomarina_piscisalsi	-	-	+
Arcobacter_defluvii	-	-	+
Olavius_algarvensis_spirochete_endosymbiont	+	+	+
Sulfurovum_sp._UBA12169	+	+	+
Helicobacter_saguini	+	+	+
Roseburia_sp._AF20-18LB	+	+	+
Fibrobacter_sp._UWB13	+	+	+
Bacteroides_sp._2_1_22	+	+	+
Anaerophaga_thermohalophila	+	+	+
Eubacterium_sp._AF22-8LB	+	+	+
Clostridium_vincentii	+	+	+
Hungateiclostridium_straminisolvens	+	+	+
Selenomonas_massiliensis	+	+	+
Kallipyga_massiliensis	+	+	+
Paenibacillus_sp._VKM_B-2647	+	+	+
Flavobacterium_phragmitis	+	+	+
Helicobacter_pylori	+	+	+

Chloroherpeton_thalassium	+	+	+
Candidatus_Curtissbacteria_bacterium_RIFCSPHIGHO2_02_FU	+	-	+
Helicobacter_sp._MIT_01-6242	-	-	+
Collinsella_vaginalis	+	+	+
Bacillus_timonensis	+	+	+
Paenibacillus_jilunlii	+	+	+
Gottschalkia_acidurici	+	+	+
Caldisalinibacter_kiritimatiensis	+	+	+
Clostridium_sp._C8	+	+	+
Paenibacillaceae_bacterium	+	+	+
Anoxybacter_fermentans	+	+	+
Paenibacillus_agaridevorans	+	+	+
Lysinibacillus_xylanilyticus	+	+	+
Syntrophomonas_palmitatica	+	+	+
Enhydrobacter_sp._H5	+	+	+
Lachnospiraceae_bacterium_AM26-1LB	+	+	+
Bacillus_pseudomycoides	+	+	+
Bacteroides_intestinalis_CAG:564	+	+	+
Ruminococcus_obeum_CAG:39	+	+	+
Paenibacillus_sp._Leaf72	+	+	+
Chlorobi_bacterium_OLB5	+	+	+
Bacillus_tuaregi	+	-	+
Candidatus_Dependentiae_bacterium	+	+	+
Bacteriodetes_bacterium	+	+	+
bacterium_BMS3Bbin05	+	+	+
Stomatobaculum_longum	+	+	+
Blautia_sp._AM42-2	+	+	+
Porphyromonas_macacae	+	+	+
Gelidibacter_algens	+	+	+
Romboutsia_sp._Marseille-P6047	+	+	+
Flavobacterium_psychrophilum	+	+	+
Methylovulum_psychrotolerans	+	+	+
Erysipelotrichaceae_bacterium_AM17-60	+	+	+
Desulfohalobium_retbaense	+	+	+
Erysipelotrichaceae_bacterium_GAM147	+	+	+
Opitutaceae_bacterium_TSB47	+	+	+
Paenibacillus_macquariensis	+	+	+
Veillonella_seminalis	+	+	+
Bacteroidetes_bacterium_GWF2_38_335	+	+	+
Geodermatophilus_poikilotrophus	-	-	+
Mouse_mammary_tumor_virus	+	+	+
Microbacterium_arborescens	+	+	+
Desulfitibacter_sp._BRH_c19	+	+	+
Viridibacillus_sp._FSL_H7-0596	+	+	+
uncultured_Sporomusa_sp.	+	+	+
Haloplasma_contractile	+	+	+
Eubacterium_siraeum_CAG:80	+	+	+
Bacteroidetes_bacterium_RIFOXYC12_FULL_35_7	+	+	+
Prevotella_sp._FD3004	+	+	+
Bacteroides_sp._OM05-12	+	+	+
Calditrichaeota_bacterium	+	+	+
Cytophagales_bacterium	+	+	+
Bacteroides_sp._44_46	+	+	+
Bacteroides_coprosuis	+	+	+
Sporomusa_silvacetica	+	+	+

Prevotella_sp._oral_taxon_473	+	+	+
Bacteroidales_bacterium_KA00251	+	+	+
Chitinophaga_arvensicola	+	+	+
Tannerella_sp._CAG:51	+	+	+
Bacteroidales_bacterium_Barb6XT	+	+	+
Verrucomicrobia_bacterium_IMCC26134	+	+	+
Coprobacillus_sp._AM28-15LB	+	+	+
Collinsella_stercoris	+	+	+
Candidatus_Saccharibacteria_bacterium_HGW-Saccharibacteria	+	+	+
Bacillus_sp._491mf	-	-	+
Leifsonia_sp._SCN_70-46	-	-	+
Microbacterium_sp._AISO3	+	+	+
Bacillus_gobiensis	+	+	+
Dactylococcopsis_salina	+	+	+
Succinatimonas_hippei	+	+	+
Vibrio_xuii	+	+	+
Campylobacter_sp._S0112	+	+	+
Capnocytophaga_gingivalis	+	+	+
Enterobacter_cloacae	+	+	+
Brevibacillus_laterosporus	+	+	+
Clostridium_sp._29_15	+	+	+
Bacteroidetes_bacterium_GWC2_46_850	+	+	+
Firmicutes_bacterium_GWF2_51_9	+	+	+
Garciella_nitratireducens	+	+	+
Streptococcus_bovimastitidis	-	+	+
Lachnoclostridium_sp._SNUG30099	+	+	+
Treponema_sp._JC4	+	+	+
Paenibacillus_sp._PCH8	+	+	+
Coprobacillus_sp._AM37-9BH	-	+	+
Streptococcus_parauberis	+	+	+
Enterococcus_aquimarinus	+	+	+
Mitsuokella_jalaludinii	+	+	+
Acetomicrobium_flavidum	-	+	+
Streptomyces_griseocarneus	+	-	+
Azospira_sp._I13	-	-	+
Desulfosporosinus_hippei	+	+	+
[Ruminococcus]_gnavus_CAG:126	+	+	+
uncultured_bacterium_Contig99	+	+	+
Peptostreptococcaceae_bacterium_AS15	+	+	+
Firmicutes_bacterium_CAG:321_26_22	+	+	+
Ruminococcus_sp._AM44-9AT	+	+	+
Xanthomonadales_bacterium	+	+	+
Amphibacillus_jilinensis	+	+	+
Thermincola_ferriacetica	+	+	+
Thermoplasmatales_archaeon_SG8-52-4	+	+	+
Chloroflexi_bacterium_HGW-Chloroflexi-3	+	+	+
Gordonibacter_urolithinfaciens	+	+	+
Photobacterium_sp._J15	+	+	+
Atopobium_sp._oral_taxon_199	+	+	+
Clostridium_ihumii	+	+	+
Pseudoalteromonas_neustonica	+	+	+
Lactococcus_raffinolactis	+	+	+
Lactobacillus_nodensis	-	-	+
Alteromonas_stellipolaris	-	-	+
Candidatus_Uhrbacteria_bacterium_GW2011_GWF2_39_13	-	-	+

Ruminococcus_sp._AF18-29	+	+	+
Clostridiisalibacter_paucivorans	+	+	+
Anaerovibrio_sp._RM50	+	+	+
Caldibacillus_debilis	+	+	+
Kingella_negevensis	-	+	+
Prevotella_sp._CAG:386	+	+	+
Treponema_sp._C6A8	+	+	+
Clostridia_bacterium_41_269	+	+	+
Clostridium_algidicarnis	+	+	+
Collinsella_sp._An271	+	+	+
Thermodesulfitimonas_autotrophica	+	+	+
Firmicutes_bacterium_AM41-11	+	+	+
uncultured_bacterium_Contig16	+	+	+
Blautia_sp._TF12-12AT	-	+	+
Sphingobacteriales_bacterium_44-15	-	+	+
Ruminococcus_sp._AM23-1	+	+	+
Scardovia_wiggsiae	+	+	+
Lactobacillus_rogosae	+	+	+
Denitrobacterium_detoxificans	+	+	+
Lactobacillus_acidipiscis	+	+	+
Carnobacterium_maltaromaticum	+	+	+
Bradyrhizobium_sp.	+	+	+
Methanophagales_archaeon	+	+	+
Bacillus_andreraoultii	+	+	+
Olsenella_sp._KH1P3	+	+	+
Cohnella_lupini	+	+	+
Anaerococcus_hydrogenalis	+	+	+
Eubacterium_sp._TM05-53	+	+	+
uncultured_bacterium_Contig248	+	+	+
Campylobacter_upsaliensis	+	+	+
Paenibacillus_senegalimassiliensis	+	+	+
Desulfovibrio_zosterae	+	+	+
Nitrospirae_bacterium_HCH-1	+	+	+
Flavobacteriaceae_bacterium	+	+	+
Prevotella_veroralis	+	+	+
Prevotella_sp._P4-76	+	+	+
Coprococcus_sp._AF16-22	+	+	+
Anaeroarcus_burkinensis	+	+	+
Flammeovirga_sp._MY04	+	+	+
Bacteroidetes_bacterium_GWE2_29_8	+	+	+
Ruminococcus_sp.	+	+	+
Eubacterium_sp._38_16	+	+	+
Kribbella_sp._NEAU-SW521	+	+	+
Bacteroidia_bacterium_43-41	+	+	+
Enterococcus_sp._9E7_DIV0242	+	+	+
Peptococcaceae_bacterium_BRH_c4b	+	+	+
Firmicutes_bacterium_CAG:321	+	+	+
Oenococcus_oeni	+	+	+
Erysipelotrichaceae_bacterium_21_3	+	+	+
Bacteroides_sp._HMSC067B03	+	+	+
Bacillus_sp._YN-1	+	+	+
Soehngenia_sp._W6	+	+	+
Bifidobacterium_animalis	+	+	+
Myxococcus_macrosporus	+	+	+
Dolosigranulum_pigrum	+	+	+

Methanococcoides_methylutens	+	+	+
Eubacterium_aggregans	+	+	+
Bacteroides_sp._AF16-49	+	+	+
Polaribacter_sp._KT_15	+	+	+
Caloramator_mitchellensis	+	+	+
Candidatus_Jettenia_caeni	+	+	+
Porphyromonas_crevioricanis	+	+	+
Pelotomaculum_sp._PtaB.Bin013	+	+	+
Prevotella_sp._F0091	+	+	+
Bacillus_sp._T33-2	+	+	+
Clostridium_sp._CAG:914	+	+	+
Paenibacillus_kribbensis	+	+	+
Ruminococcus_sp._AM43-6	+	+	+
Firmicutes_bacterium_CAG:884	+	+	+
Blautia_sp._AF32-4BH	+	+	+
Vibrio_cholerae	+	+	+
Fusobacterium_hwasookii	-	+	+
Pseudomonas_veronii	+	+	+
Bifidobacterium_scardovii	-	+	+
[Collinsella]_massiliensis	+	+	+
Brachyspira_murdochii	+	+	+
Segetibacter_koreensis	-	+	+
Berkelbacteria_bacterium_GW2011_GWB1_38_5	+	-	+
Mobiluncus_curtisii	+	+	+
Paenibacillus_sp._P1XP2	+	+	+
Bacillus_salarius	+	+	+
Desulfosporosinus_sp._HMP52	+	+	+
Caloranaerobacter_azorensis	+	+	+
Candidatus_Omnitrophica_bacterium	+	+	+
Flavobacterium_sp.	+	+	+
candidate_division_NC10_bacterium_RIFCSPLOWO2_02_FUI	+	+	+
Massilibacteroides_vaginae	+	+	+
Clostridiales_bacterium_S5-A14a	+	+	+
Candidatus_Saccharibacteria_bacterium_TM7__EAM_G5_2_H	+	+	+
Rubneribacter_badeniensis	+	+	+
Gottschalkiaceae_bacterium	+	+	+
Laribacter_hongkongensis	+	+	+
Firmicutes_bacterium_HGW-Firmicutes-19	+	+	+
Senegalimassilia_sp._KGMB04484	+	+	+
Bacillus_sp._FJAT-28004	+	+	+
Chryseobacterium_limigenitum	-	+	+
Gulosibacter_sp._10	-	-	+
Campylobacter_sp._P0094	+	+	+
Methylomusa_anaerophila	+	+	+
Paenibacillus_terrae	+	+	+
Vibrio_panuliri	-	+	+
Bacillus_thermozeamaize	+	+	+
Thermobrachium_celere	+	+	+
uncultured_bacterium_scaffold00056	+	+	+
Pedobacter_sp._R20-19	+	+	+
Ileibacterium_valens	+	+	+
Slackia_faecicanis	+	+	+
bacterium_HR16	+	+	+
Dethiosulfovibrio_salsuginis	+	+	+
Streptococcus_sanguinis	+	+	+

Lentisphaerae_bacterium_GWF2_38_69	+	+	+
Lactobacillus_sp._54-2	+	-	+
uncultured_bacterium_Contig1604	+	+	+
Pelagibacteraceae_bacterium_TMED287	+	+	+
Paraburkholderia_sp._DHOA04	+	+	+
Aeromonas_hydrophila	+	+	+
Paenibacillus_pectinilyticus	+	+	+
Dictyoglomus_sp._NZ13-RE01	+	+	+
Caecibacter_massiliensis	+	+	+
Peptostreptococcus_anaerobius	+	+	+
Parabacteroides_sp._AT13	+	+	+
Bacillus_aidingensis	+	+	+
CrAssphage_sp.	-	+	+
Epulopiscium_sp._Nuni2H_MBin001	+	+	+
Blautia_sp._CAG:237	+	+	+
Bacteroidetes_bacterium_HGW-Bacteroidetes-16	+	+	+
Clostridium_sp._CAG:451	+	+	+
Bacillus_sp._FJAT-22090	+	+	+
Coriobacteriaceae_bacterium_68-1-3	+	+	+
Paenibacillus_graminis	+	+	+
Parabacteroides_sp._ASF519	+	+	+
Clostridium_sp._CAG:245	+	+	+
Desulfitobacterium_sp._PCE1	+	+	+
Veillonella_rogosae	-	+	+
[Eubacterium]_sulci	+	+	+
Bacillus_oleivorans	+	+	+
Acholeplasma_laidlawii	+	+	+
Candidatus_Pacearchaeota_archaeon_ex4484_31	+	+	+
Brachyspira_sp._CAG:700	+	+	+
Cytophaga_xylanolytica	+	+	+
Mycoplasma_hominis	+	+	+
Ilyobacter_polytropus	+	+	+
Prevotella_sp._P5-50	+	+	+
Fusobacterium_sp._CM22	+	+	+
Paenibacillus_sp._Soil766	+	+	+
Paenibacillus_sp._1_12	+	+	+
Desulfotomaculum_nigrificans	+	+	+
Acholeplasma_modicum	+	+	+
Balneicella_halophila	+	+	+
Paenibacillus_montanisoli	+	+	+
Collinsella_intestinalis	+	+	+
Paenibacillus_sp._DMB5	+	+	+
Haemophilus_parahaemolyticus	+	+	+
Pseudomonas_balearica	+	+	+
Peptostreptococcus_sp._Marseille-P4308	+	+	+
Clostridium_sp._AF37-7	+	+	+
Paenibacillus_alvei	+	+	+
Clostridium_sp._AF35-15	+	+	+
Thermosediminibacter_oceani	+	+	+
Piromyces_finnis	+	+	+
Enterococcus_rivorum	+	+	+
Chitinispirillum_alkaliphilum	+	+	+
Flavobacteriaceae_bacterium_UJ101	+	+	+
Actinomadura_echinospora	+	+	+
Thermoanaerobacterium_saccharolyticum	+	+	+

Bacteroides_sp._1_1_30	+	+	+
Inediibacterium_massiliense	+	+	+
Anaeromicrobium_sediminis	+	+	+
Blautia_sp._AM46-3MH	+	+	+
Epulopiscium_sp._SCG-D08WGA-EpuloA1	+	+	+
Ruminococcus_sp._CAG:60	+	+	+
Parabacteroides_sp._merdae-related_45_40	+	+	+
Thermoflexia_bacterium	+	+	+
Ruminococcus_sp._AF37-20	+	+	+
Orenia_marismortui	+	+	+
Bacillus_cohnii	+	+	+
Lactobacillus_sp._Marseille-P3519	+	+	+
Candidatus_Uhrbacteria_bacterium	-	-	+
Paenibacillus_sp._CAA11	+	+	+
Parcubacteria_group_bacterium_GW2011_GWF2_40_10	-	+	+
Faecalibacterium_sp._CAG:82-related_59_9	+	+	+
Gammaproteobacteria_bacterium_2W06	+	+	+
Clostridium_sp._DSM_8431	+	+	+
Thermosinus_carboxydivorans	+	+	+
Massilia_lutea	+	+	+
Pseudomonas_otitidis	-	+	+
Orenia_metallireducens	+	+	+
[Clostridium]_dakarense	+	+	+
Bacillus_clausii	+	+	+
candidate_division_WS6_bacterium_GW2011_GWF1_35_23	+	+	+
Paenibacillus_sp._FSL_H7-0357	+	+	+
Desulfosporosinus_lacus	+	+	+
Petrimonas_sp._IBARAKI	+	+	+
Blautia_sp._AM29-29	+	+	+
Collinsella_sp._An2	+	+	+
Aquimarina_sp._AD10	+	+	+
Porphyromonas_somerae	+	+	+
Paenibacillus_sp._JDR-2	+	+	+
Prevotella_copri_CAG:164	+	+	+
Lachnoclostridium_sp._SNUG30370	+	+	+
Bacteroides_ovatus_CAG:22	+	+	+
Eggerthella_sp._CAG:209	+	+	+
Peptococcaceae_bacterium_BRH_c4a	+	+	+
Roseburia_inulinivorans_CAG:15	+	+	+
Spirochaetae_bacterium_HGW-Spirochaetae-9	+	+	+
Geofilum_rubicundum	+	+	+
Leptotrichia_wadei	+	+	+
Lactobacillus_panis	+	+	+
Bacillus_cihuensis	+	+	+
Parcubacteria_bacterium_DG_74_2	+	+	+
Lactococcus_virus_KSY1	-	-	+
Hyalangium_minutum	+	+	+
Faecalibacterium_sp._AM43-5AT	+	+	+
Anaerococcus_sp._Marseille-P3625	+	+	+
Capnocytophaga_canis	+	+	+
Candidatus_Magnetobacterium_bavaricum	+	+	+
Clostridium_sp._DMHC_10	+	+	+
Clostridium_sulfidigenes	+	+	+
Nocardia_exalbida	+	+	+
Lachnoclostridium_sp._SNUG30386	+	+	+

Carnobacterium_inhibens	+	+	+
Urinacoccus_massiliensis	+	+	+
Cupriavidus_metallidurans	+	+	+
Elizabethkingia_meningoseptica	+	+	+
Paenibacillus_ginsengihumi	+	+	+
Prevotella_aurantiaca	+	+	+
Desulfosporosinus_sp._OT	+	+	+
Croceivirga_radicis	+	+	+
Bacteroidetes_bacterium_GWA2_42_15	+	+	+
Lagierella_massiliensis	-	+	+
Firmicutes_bacterium_HGW-Firmicutes-5	+	+	+
Enterococcus_mundtii	+	+	+
Absiella_sp._AM10-20	+	+	+
Candidatus_Adiutrix_intracellularis	+	+	+
Halonatronum_saccharophilum	+	+	+
Tenericutes_bacterium_GWA2_38_26	+	+	+
Fibrobacter_sp._UWOV1	+	+	+
Sutterella_megalosphaeroides	+	+	+
Candidatus_Scalindua_sp._SCAELEC01	+	+	+
Lactobacillus_bombi	+	-	+
Methanoplanus_limicola	-	+	+
Oribacterium_asaccharolyticum	+	+	+
Anaerosporomusa_subterranea	+	+	+
Cohnella_sp._18JY8-7	+	+	+
Virgibacillus_senegalensis	+	+	+
Slackia_isoflavoniconvertens	+	+	+
Bacillus_aerius	+	+	+
Desulfovibrio_sp._X2	+	+	+
Clostridium_sp._CAG:417	+	+	+
Syntrophothermus_lipocalidus	+	+	+
Psychrilyobacter_atlanticus	+	+	+
Candidatus_Magnetoglobus_multicellularis	+	+	+
Veillonella_sp._AS16	-	+	+
Methanobrevibacter_thaueri	+	+	+
Bradyrhizobium_sp._NAS96.2	+	+	+
Lactobacillus_antri	+	+	+
Lactobacillus_curieae	+	-	+
Sphaerochaeta_coccoides	+	+	+
Flavobacterium_sp._Root186	-	+	+
Candidatus_Aerophobetes_bacterium	+	+	+
Lentisphaera_araneosa	+	+	+
Avibacterium_paragallinarum	+	+	+
Dialister_invisus	+	+	+
Peptococcaceae_bacterium_BICA1-7	+	+	+
Paenibacillus_sp._VMFN-D1	+	+	+
Propionispira_raffinosivorans	+	+	+
Anaerococcus_sp._HMSC065G05	+	+	+
Methanofollis_liminatans	+	+	+
Tenacibaculum_agarivorans	+	+	+
Pedobacter_sp._FJ4-8	+	+	+
Sporomusa_malonica	+	+	+
Candidatus_Melainabacteria_bacterium_LEY3_CP_29_8	+	+	+
Candidatus_Cloacimonas_sp._4484_209	+	+	+
Lachnospiraceae_bacterium_AM21-21	+	+	+
Paenibacillus_rigui	+	+	+

Pseudomonas_fluorescens	+	+	+
Clostridium_sp._CAG:628	+	+	+
Paenibacillus_sp._HW567	+	+	+
Desulfovibrio_alcoholivorans	+	+	+
Paenibacillus_sp._FSL_R7-269	+	+	+
Blautia_sp._TF10-30	+	+	+
Paenibacillus_sp._FSL_R7-277	+	+	+
Bacillus_lonarensis	+	+	+
Symbiobacterium_thermophilum	+	+	+
Chitinophaga_dinghuensis	-	+	+
Sediminibacterium_sp._FEMGT703S	+	+	+
Bacillus_niacini	-	+	+
Eggerthellales_bacterium	+	+	+
Clostridium_sp._AF46-12NS	+	+	+
Arcobacter_bivalviorum	+	+	+
Cohnella_sp._M2MS4P-1	+	+	+
Streptococcus_marmotae	+	+	+
Syntrophus_aciditrophicus	-	+	+
Polaribacter_sp._MED152	+	-	+
Enterococcus_phage_EF1	-	-	+
Rhizobium_sp._CF048	-	+	+
Candidatus_Annandia_adelgestsuga	-	+	+
Paludibacter_propionicigenes	+	+	+
uncultured_Collinsella_sp.	+	+	+
Marinilabiliales_bacterium	+	+	+
Coprococcus_sp._AF16-5	+	+	+
Bacillus_obstructivus	+	+	+
Selenomonas_sp._FOBRC9	+	+	+
Bacillus_thermoamylovorans	+	+	+
Paenibacillus_selenitireducens	+	+	+
Clostridium_sp._DSM_4029	+	+	+
Sediminihabitans_luteus	+	+	+
Arenibacter_algicola	+	+	+
Fibrobacter_sp._UWRM	+	+	+
Pantoea_stewartii	-	+	+
Peptoniphilus_asaccharolyticus	+	+	+
Chryseobacterium_taklimakanense	+	+	+
Prevotella_bergensis	+	+	+
Siansivirga_zeaxanthinifaciens	+	+	+
Olsenella_umbonata	+	+	+
Prevotella_sp._CAG:604	+	+	+
Thermoanaerobacterales_bacterium_50_218	+	+	+
Thermoplasmata_archaeon	+	+	+
Bacillus_novalis	+	+	+
Lactobacillus_paragasseri	+	+	+
Nitrosomonas_ureae	+	+	+
Paenibacillus_sp._18JY67-1	+	+	+
Sphaerotilus_natans	+	+	+
Candidatus_Gastranaerophilales_bacterium_HUM_7	+	+	+
Paenibacillus_sp._FSL_P4-0081	+	+	+
Paenibacillus_sp._7541	+	+	+
Alkalicoccus_saliphilus	-	+	+
Cupriavidus_sp._SK-3	-	+	+
Streptococcus_dysgalactiae	-	+	+
Vibrio_spartinae	+	+	+

Anabaena_sp._CA_=_ATCC_33047	-	+	+
Lactobacillus_ingluviei	-	-	+
Clostridium_sp._AF15-41	+	+	+
Paenibacillus_sp._DXL2	+	+	+
Staphylococcus_lentus	+	+	+
Ignavibacteriae_bacterium_HGW-Ignavibacteriae-4	+	+	+
Peptococcus_niger	+	+	+
Halanaerobium_salsuginis	+	+	+
Blautia_sp._AF14-40	+	+	+
Geofilum_rhodophaeum	+	+	+
Bacillus_camelliae	+	+	+
Candidatus_Cloacimonas_sp._SDB	-	+	+
Bacillus_sp._CGMCC_1.16541	+	+	+
Adhaeribacter_aquaticus	+	+	+
Bacillus_subterraneus	+	+	+
Enterococcus_avium	+	+	+
Ruminococcus_sp._AM54-1NS	+	+	+
Candidatus_Marispirochaeta_associata	+	+	+
Firmicutes_bacterium_HGW-Firmicutes-10	-	+	+
Clostridium_thermopalmarium	+	+	+
Desulfotomaculum_copahuensis	+	+	+
Lysinibacillus_odysseyi	+	+	+
Desulfofundulus_kuznetsovii	+	+	+
Treponema_vincentii	+	+	+
Paenibacillus_sp._FSL_H7-0331	+	+	+
Atopobium_sp._HMSC064B08	+	+	+
Sporosarcina_sp._PTS2304	+	+	+
Caldanaerovirga_acetigignens	+	+	+
Candidatus_Saccharibacteria_bacterium_CG2_30_41_52	+	-	+
Streptomyces_sp._OK885	+	-	+
Mycoplasma_agalactiae	-	-	+
Ruminococcus_sp._OM04-4AA	+	+	+
Lactobacillus_harbinensis	+	+	+
Erythrobacter_lutimaris	+	+	+
Sunxiuqinia_dokdonensis	+	+	+
Selenomonas_sp._oral_taxon_149	+	+	+
Streptococcus_lutetiensis	+	+	+
Caldicellulosiruptor_bescii	+	+	+
Erysipelotrichaceae_bacterium_MTC7	+	+	+
Desulfotomaculum_reducens	+	+	+
Bacillus_acidiceler	+	+	+
Sphingobacteriales_bacterium_UPWRP_1	+	+	+
Ruminococcus_sp._AM50-15BH	+	+	+
Longilinea_arvoryzae	+	+	+
Bacteroides_thetaiotaomicron_CAG:40	+	+	+
Coprobacillus_sp._D7	+	+	+
Clostridium_sp._CAG:217_53_7	+	+	+
Desulfobulbaceae_bacterium	+	+	+
Erwiniaceae_bacterium_4572_131	+	+	+
Prevotella_sp._oral_taxon_376	+	+	+
Jeotgalibaca_sp._H21T32	+	+	+
Ruminococcus_sp._AM18-44	+	+	+
Ekhidna_lutea	+	+	+
Helcococcus_kunzii	+	+	+
Labilibaculum_manganireducens	-	+	+

Bacillus_salsus	+	+	+
Bacillus_sp._FJAT-27225	+	+	+
Enterobacter_sp._WP_7_1	+	+	+
Geobacter_sp._M18	+	+	+
Alicyclobacillus_acidiphilus	+	+	+
[Clostridium]_hiranonis	+	+	+
Synechocystis_sp._PCC_6803	+	+	+
Bacteroidales_bacterium_6E	+	+	+
Olsenella_sp._kh2p3	+	+	+
Eggerthellaceae_bacterium_AT8	+	+	+
Alteromonas_sp._Nap_26	-	-	+
Halomonas_sp._N3-2A	+	+	+
Desmodus_rotundus_endogenous_retrovirus	+	+	+
Mitsuokella_sp._oral_taxon_131	+	+	+
Ruminococcus_sp._AM34-9LB	+	+	+
Jaagsiekte_sheep_retrovirus	+	+	+
Thermohalobacter_berrensis	+	+	+
Paenibacillus_borealis	+	+	+
Prevotella_sp._CAG:592	+	+	+
Bacillus_lentus	+	+	+
Pectobacterium_polaris	+	+	+
Paenibacillus_mucilaginosus	+	+	+
Clostridium_coskatii	+	+	+
Tissierella_praeacuta	+	+	+
Treponema_sp._CETP13	+	+	+
uncultured_bacterium_Ad_087_C16_contig1	+	+	+
Bacteroidetes_bacterium_38_7	+	+	+
Kordia_periserrulae	+	+	+
Mariniphaga_sediminis	+	+	+
Sporomusaceae_bacterium	+	+	+
Candidatus_Woesearchaeota_archaeon_CG_4_10_14_0_2_um_	+	+	+
Mogibacterium_timidum	-	+	+
Campylobacter_virus_CPX	+	+	+
Streptococcus_sp._HMSC10E12	+	+	+
Leptotrichia_sp._OH3620_COT-345	+	+	+
Anabaenopsis_circularis	-	+	+
Coprococcus_comes_CAG:19	+	+	+
Fibrobacter_sp._UWR2	-	+	+
Arthrobacter_sp._ZXY-2	+	-	+
Rhodococcus_sp._ADH	-	-	+
Epulopiscium_sp._Nele67-Bin004	+	+	+
Sutterella_sp._AM18-8-1	+	+	+
Clostridiales_bacterium_GWE2_32_10	+	+	+
Paenibacillus_naphthalenovorans	+	+	+
Victivallis_vadensis	+	+	+
Lachnospiraceae_bacterium_AM25-40	-	+	+
Treponema_phagedenis	+	+	+
Scytonema_hofmannii	+	+	+
Bacteroides_sp._D1	+	+	+
Bacteroides_vulgatus_CAG:6	+	+	+
Flavobacterium_sp._IMCC34758	+	+	+
Fontibacillus_panacisegetis	+	+	+
Bacillus_horikoshii	+	+	+
Pelotomaculum_thermopropionicum	+	+	+
Mangrovibacterium_diazotrophicum	+	+	+

Rhizophagus_clarus	+	+	+
Kineosporia_sp._A_224	+	+	+
Sphingobacterium_sp._2c-3	+	+	+
Brevibacillus_formosus	+	+	+
Desulfallas_alcoholivorax	+	+	+
Flavobacterium_oncorhynchi	+	+	+
Acholeplasma_brassicae	+	+	+
bacterium_I07	+	+	+
Oceanibaculum_indicum	+	+	+
Nostoc_sp._CENA543	+	+	+
Firmicutes_bacterium_CAG:272_52_7	+	+	+
Candidatus_Competibacteraceae_bacterium	+	+	+
Peptostreptococcus_stomatis	+	+	+
Coprobacillus_sp._3_3_56FAA	+	+	+
Citrobacter_freundii	+	+	+
Clostridium_sp._AF27-5AA	+	+	+
Caldicellulosiruptor_morganii	-	+	+
Paenibacillus_xerothermodurans	+	+	+
bacterium_D16-34	+	+	+
Candidatus_Termititenax_aidoneus	+	-	+
Nostocales_cyanobacterium	+	-	+
Tetragenococcus_osmophilus	-	-	+
Eggerthella_sp._51_9	+	+	+
Solirubrobacterales_bacterium_67-14	-	+	+
Eubacterium_sp._CAG:192	+	+	+
Corynebacterium_diphtheriae	-	+	+
Alkaliphilus_sp.	+	+	+
Bulleidia_extructa	+	+	+
Porphyromonas_uenonis	+	+	+
Desulfuribacillus_alkaliarsenatis	+	+	+
Bacillus_sp._TS-2	+	+	+
Fervidicola_ferrireducens	+	+	+
Firmicutes_bacterium_CAG:308	+	+	+
Niastella_populi	+	+	+
[Flexibacter]_sp._ATCC_35208	+	+	+
Carboxydothermus_pertinax	+	+	+
Streptococcus_uberis	+	+	+
Marinilabilia_salmonicolor	+	+	+
Candidatus_Cloacimonetes_bacterium_HGW-Cloacimonetes-2	+	+	+
Anaerobacillus_isosaccharinicus	+	+	+
Firmicutes_bacterium_HGW-Firmicutes-2	+	+	+
Ruminococcus_sp._TM09-4	+	+	+
Bacteroides_sp._AF32-8BH	+	+	+
[Eubacterium]_minutum	+	+	+
Bacteroidetes_bacterium_GWE2_42_42	+	+	+
Pedobacter_jeongneungensis	-	+	+
Sunxiuqinia_elliptica	+	+	+
Paenibacillus_barcinonensis	+	+	+
Bacteroides_sp._OF02-3LB	+	+	+
Dyadobacter_soli	+	+	+
Paenibacillus_sp._FSL_R5-0345	+	+	+
Paenibacillus_sp._OSY-SE	+	+	+
Fibrobacter_sp._UWB16	+	+	+
Clostridium_sp._AF12-41	+	+	+
Armatimonadetes_bacterium_CG2_30_59_28	+	+	+

Clostridium_sp._CAG:609	+	-	+
Lactobacillus_rossiae	+	+	+
Devosia_sp._66-22	+	+	+
Caloramator_quimbayensis	+	+	+
Desulfobulbaceae_bacterium_DB1	+	+	+
Collinsella_aerofaciens	+	+	+
Staphylococcus_carnosus	+	+	+
Flavobacterium_sp._Root935	+	+	+
Veillonellaceae_bacterium_DNF00626	+	+	+
Enterococcus_silesiacus	+	+	+
Marinifilaceae_bacterium_SPP2	+	+	+
Firmicutes_bacterium_CAG:270	+	+	+
Ruminococcus_sp._AF17-11	+	+	+
Clostridium_akagii	+	+	+
Chryseobacterium_arachidis	+	+	+
Spirosoma_montaniterrae	-	+	+
Fibrobacter_sp._UWS1	+	+	+
Helicobacter_sp._TUL	+	+	+
Algoriphagus_aquimarinus	-	+	+
Desulfobacterium_sp._4572_20	+	+	+
Firmicutes_bacterium_HGW-Firmicutes-17	+	+	+
Bacteroidetes_bacterium_GWE2_32_14	+	+	+
Paenibacillus_gorillae	+	+	+
Pedobacter_nutrimenti	+	+	+
Marinirhabdus_gelatinilytica	+	+	+
Altibacter_lentus	+	+	+
Curvibacter_delicatus	+	+	+
Methanobrevibacter_filiformis	+	+	+
Sphingobacterium_sp._IITKGP-BTPF85	+	+	+
Halobacteroides_halobius	+	+	+
Paenibacillus_whitsoniae	+	+	+
Selenomonas_bovis	+	+	+
Methanomassiliicoccus_luminyensis	+	+	+
Helicobacter_ganmani	+	-	+
unicellular_cyanobacterium_SU3	-	-	+
Simian_retrovirus_8	+	+	+
Euhalothece_sp._KZN_001	+	+	+
Blautia_sp._AM22-22LB	+	+	+
Parvimonas_micra	+	+	+
Bacillus_massiliogorillae	+	+	+
Paenibacillus_sp._yr247	+	+	+
Hymenobacter_sp._MIMBbqt21	+	+	+
Bacteroides_sp._AM54-2NS	+	+	+
Caldicellulosiruptor_obsidiansis	+	+	+
Acidaminococcus_timonensis	+	+	+
Gelidibacter_gilvus	+	+	+
Schaalia_vaccimaxillae	+	+	+
Methanosarcina_lacustris	+	+	+
Methanosarcina_sp._MTP4	+	+	+
Bacteroides_clarus_CAG:160	-	+	+
Fenollaria_timonensis	+	+	+
Thalassospira_xiamenensis	+	+	+
Syntrophaceticus_schinkii	+	+	+
Candidatus_Methanoplasma_termitum	+	+	+
Paenibacillus_sp._FSL_H7-0737	+	+	+

Haloferax_elongans	+	+	+
Eubacterium_sp._CAG:202	+	+	+
Spirochaetes_bacterium_RIFOXYC1_FULL_54_7	+	+	+
Romboutsia_lituseburensis	+	+	+
Tenericutes_bacterium_GWA2_35_7	+	+	+
Parabacteroides_sp._AF27-14	+	+	+
Lysinibacillus_manganicus	+	+	+
Bacteroides_sp._AM26-2	+	+	+
Mogibacterium_diversum	+	+	+
Levyella_massiliensis	+	+	+
Virgibacillus_salinus	+	+	+
Caloramator_proteoclasticus	+	+	+
Candidatus_Saccharibacteria_bacterium_TM7_CMJM_G6_1_H	-	+	+
Bacillus_sp._LL01	+	-	+
Vibrio_phage_1.170.O._10N.261.52.C3	+	-	+
Gardnerella_vaginalis	+	+	+
Stenotrophomonas_maltophilia	+	+	+
Paenibacillus_sp._FF9	+	+	+
Bacillus_virus_SPO1	+	+	+
Caldilineae_bacterium	+	+	+
Helicobacter_sp._MIT_03-1616	+	+	+
Apibacter_mensalis	+	+	+
Thermoplasmata_archaeon_M8B2D	+	+	+
Clostridium_sp._AF46-9NS	+	+	+
Pseudopropionibacterium_propionicum	+	+	+
Clostridium_fallax	+	+	+
Streptococcus_agalactiae	+	+	+
Lewinella_nigricans	+	+	+
Granulicatella_adiacens	+	+	+
Tumebacillus_flagellatus	+	+	+
[Clostridium]_thermoalcaliphilum	-	+	+
Chloroflexi_bacterium_HGW-Chloroflexi-5	+	+	+
Bacillus_beveridgei	+	+	+
Acidimicrobiia_bacterium	+	+	+
Variovorax_sp._KBW07	+	+	+
Bacteroides_sp._AF25-18	+	+	+
Parabacteroides_sp._AM27-42	+	+	+
Smithella_sp._F21	+	+	+
Mycobacterium_sp._141	+	+	+
Paenibacillus_sp._FSL_R7-0273	+	+	+
Fibrobacter_sp._UWOS	+	+	+
Clostridium_sp._28_12	+	+	+
Virgibacillus_proomii	+	+	+
Candidatus_Saccharibacteria_bacterium_32-49-12	+	+	+
Lentisphaerae_bacterium_GWF2_44_16	+	+	+
Bacillus_taeanensis	+	+	+
Chloroflexi_bacterium_HGW-Chloroflexi-2	+	+	+
Mucilaginibacter_sp._OK268	-	+	+
Selenomonas_sp._oral_taxon_892	+	+	+
Psychrobacillus_psychrotolerans	-	+	+
Osedax_symbiont_Rs1	-	-	+
Clostridium_chauvoei	+	+	+
Fibrobacter_sp._UWS4	+	+	+
Firmicutes_bacterium_HGW-Firmicutes-4	+	+	+
Dialister_succinatiphilus	+	+	+

Paenibacillus_sp._TI45-13ar	+	+	+
Gottschalkia_purinilytica	+	+	+
Ruminococcus_sp._TF12-19AC	+	+	+
Moorella_sp._60_41	+	+	+
Thermoprotei_archaeon	+	+	+
Aerococcus_urinaeequi	+	+	+
Bacillus_sp._1NLA3E	+	+	+
Candidatus_Gastranaerophilales_bacterium_HUM_16	+	+	+
Blautia_sp._OM06-15AC	+	+	+
Bacteroidetes_bacterium_HGW-Bacteroidetes-2	-	+	+
Ruminococcus_sp._CAG:379	+	+	+
Fusobacterium_sp._CAG:439	+	+	+
Domibacillus_mangrovi	+	+	+
Amphibacillus_sediminis	+	+	+
Vagococcus_humatus	+	+	+
Belliella_baltica	+	+	+
uncultured_bacterium_Csd4	+	+	+
Prolixibacter_denitrificans	+	+	+
Bacillus_sp._MB353a	+	+	+
Coprococcus_sp._ART55/1	+	+	+
Bacteroidetes_bacterium_HGW-Bacteroidetes-21	+	+	+
Olsenella_sp._KCTC_15699	+	+	+
Bifidobacterium_reuteri	+	+	+
Clostridium_hydrogeniformans	+	+	+
Firmicutes_bacterium_CAG:460	+	+	+
Enterococcus_sp._kppr-6	+	+	+
Bifidobacterium_bifidum	+	+	+
Vibrio_lentus	+	+	+
Enterobacter_kobei	+	+	+
Enterococcus_phoeniculicola	+	+	+
candidate_division_TA06_bacterium_34_109	+	+	+
Marinospirillum_celere	+	+	+
Desulfotomaculum_putei	+	+	+
Treponema_endosymbiont_of_Eucomonympha_sp.	+	+	+
Fenollaria_massiliensis	+	+	+
Cellulophaga_algicola	+	-	+
Oscillatoriales_cyanobacterium_JSC-12	+	-	+
methanogenic_archaeon_mixed_culture_ISO4-G1	+	+	+
Clostridium_sp._AF24-2LB	+	+	+
Methylomagnum_ishizawai	+	+	+
Bacillus_sp._LF1	+	+	+
Desulfomicrobium_apsheronum	+	+	+
Butyricicoccus_sp._OF13-6	+	+	+
Collinsella_sp._An7	+	+	+
Candidatus_Gastranaerophilales_bacterium_HUM_23	+	+	+
Prevotella_sp._oral_taxon_313	+	+	+
Eubacterium_eligens_CAG:72	+	+	+
Corynebacterium_pelargi	+	+	+
Tenericutes_bacterium_MO-XQ	+	+	+
Paenibacillus_sp._UNC496MF	+	+	+
Heliobacillus_mobilis	+	+	+
Trichococcus_sp._ART1	+	+	+
Rhizoclosmatium_globosum	+	+	+
Geomicrobium_sp._JCM_19055	+	+	+
Flavobacterium_sp._5	-	+	+

Bacillus_virus_PBS1	+	+	+
Paenibacillus_xylanexedens	+	+	+
Nitrospirae_bacterium_CG1_02_44_142	+	+	+
Methylomicrobium_sp._wino1	+	+	+
Mycoplasma_verecundum	+	+	+
Sphingobacterium_sp._30C10-4-7	+	+	+
Lachnoanaerobaculum_sp._MSX33	+	+	+
Salipaludibacillus_aurantiacus	+	+	+
Bacillus_sp._MB2021	+	+	+
Bacillaceae_bacterium_SAS-127	+	+	+
Halanaerobium_praevalens	-	+	+
Succinatimonas_sp._CAG:777	+	+	+
Mesotoga_sp._TolDC	+	+	+
Paenibacillus_sp._XY044	+	+	+
Mycoplasma_sp._CAG:611	+	+	+
Coprococcus_sp._AF19-8AC	+	+	+
Treponema_lecithinolyticum	+	+	+
Streptococcus_oralis	+	+	+
Leptotrichia_sp._oral_taxon_215	+	+	+
Paenibacillus_kobensis	+	+	+
Paenibacillus_sp._BIHB4019	+	+	+
Ruminococcus_sp._AF25-28AC	+	+	+
bacterium_LF-3	+	+	+
Thalassobius_sp._ZQ172	+	+	+
Bacillus_onubensis	+	+	+
Archangium_gephyra	+	+	+
Enterococcus_sp._3H8_DIV0648	+	+	+
Eubacterium_dolichum_CAG:375	+	+	+
Neisseriaceae_bacterium	+	+	+
Aquimarina_sp._AU58	-	+	+
Acetobacter_sp._CAG:977	+	+	+
Thalassotalea_sp._ND16A	-	+	+
Bacteroides_plebeius_CAG:211	+	+	+
Blautia_sp._YL58	+	+	+
Geobacillus_stearothermophilus	+	+	+
Brevibacillus_agri	+	+	+
Candidatus_Atribacteria_bacterium_HGW-Atribacteria-1	+	+	+
Methanococcus_maripaludis	+	+	+
Clostridium_sp._CAG:433	+	+	+
Flavobacterium_glycines	+	+	+
Bacteroides_cellulosilyticus_CAG:158	+	+	+
Pedobacter_kyungheensis	+	+	+
Lentimicrobium_saccharophilum	+	+	+
Veillonella_dispar	+	+	+
Peptoniphilus_lacrimalis	+	+	+
Clostridium_sp._AWRP	+	+	+
Porphyromonas_gingivicanis	+	+	+
Mesorhizobium_sp.	+	+	+
Brevibacillus_thermoruber	+	+	+
Clostridium_sp._CAG:921	+	+	+
Brevibacterium_senegalense	-	+	+
Chryseobacterium_sp._SC28	+	+	+
Chromatiales_bacterium_(ex_Bugula_neritina_AB1)	+	+	+
Olivibacter_jilunii	+	+	+
Rubeoparvulum_massiliense	+	+	+

uncultured_bacterium_Contig1532b	+	+	+
[Clostridium]_paradoxum	+	+	+
Chloroflexi_bacterium_44-23	-	-	+
Anaerococcus_sp._HMSC075B03	-	-	+
Treponema_maltophilum	+	+	+
Saccharospirillum_mangrovi	+	+	+
Fusibacter_sp._A1	+	+	+
Eubacterium_sp._AM49-13BH	+	+	+
Tenericutes_bacterium_HGW-Tenericutes-5	+	+	+
Nitrospirae_bacterium	+	+	+
Acinetobacter_boissieri	+	+	+
Sinomicrobium_oceani	+	+	+
Brevibacillus_massiliensis	+	+	+
Caldicellulosiruptor_hydrothermalis	+	+	+
Bacillus_phage_vB_BceM-HSE3	+	+	+
Paenibacillus_lautus	+	+	+
Oscillatoriales_cyanobacterium	+	+	+
Candidatus_Firestonebacteria_bacterium_RIFOXYA2_FULL_4(	-	+	+
Deltaproteobacteria_bacterium_RIFCSPLOWO2_02_FULL_46_	-	+	+
Gemmatimonadetes_bacterium	+	+	+
Alphaproteobacteria_bacterium	+	+	+
Ruminobacter_sp._RM87	+	+	+
Desulfosporosinus_sp._BG	+	+	+
Bacteroidetes_bacterium_GWB2_41_8	+	+	+
Bifidobacterium_sp._2034B	+	+	+
bacterium_BMS3Bbin03	-	+	+
Candidatus_Marinimicrobia_bacterium	+	+	+
Pedobacter_luteus	+	+	+
Enorma_timonensis	+	+	+
Bacillus_sp._Y1	+	+	+
Ruminococcus_sp._AF24-16	-	+	+
Pedobacter_sp._V48	+	+	+
Centipeda_periodontii	+	+	+
Prevotella_sp._oral_taxon_299	+	+	+
Caloramator_fervidus	+	+	+
Megasphaera_hexanoica	+	+	+
Desulfuromonas_sp.	+	+	+
Parabacteroides_merdae_CAG:48	+	+	+
Caballeronia_arationis	+	+	+
Colibacter_massiliensis	+	+	+
Streptomyces_armeniacus	+	+	+
Terribacillus_saccharophilus	+	+	+
Lactococcus_termiticola	+	+	+
Campylobacter_virus_IBB35	-	+	+
Ruminococcus_sp._AM54-14NS	+	-	+
Photobacterium_proteolyticum	-	-	+
Leptospira_sp.	-	-	+
Paenibacillus_sp._CF384	+	+	+
Aneurinibacillus_aneurinilyticus	+	+	+
Lachnoanaerobaculum_sp._KCOM_2030	+	+	+
Clostridium_niameyense	+	+	+
Veillonella_sp._CHU594	+	+	+
Pseudanabaena_sp._ABRG5-3	+	+	+
Murdochiella_vaginalis	+	+	+
Ruminococcus_sp._TF12-2	+	+	+

Paenibacillus_aceti	+	+	+
Ruminococcus_sp._AM31-32	+	+	+
Cohnella_sp._K2E09-144	+	+	+
Helicobacter_sp._MIT_03-1614	+	+	+
Clostridium_sp._ND2	+	+	+
Bordetella_sp._SCN_68-11	+	+	+
Prevotella_sp._ICM33	+	+	+
Desulfurobacterium_sp._TC5-1	+	+	+
Coleofasciculus_chthonoplastes	+	+	+
Sphingobacterium_siyangense	+	+	+
Larkinella_knui	+	+	+
Porphyromonas_catoniae	+	+	+
Flavobacterium_aurantiibacter	+	+	+
Bacteroides_sp._43_46	+	+	+
Virgibacillus_halodenitrificans	+	+	+
Desulfovibrionales_bacterium_GWA2_65_9	+	+	+
Bacillus_sp._EB01	+	+	+
Candidatus_Cloacimonetes_bacterium	+	+	+
Flavobacterium_crassostreae	+	+	+
Clostridium_sp._Marseille-P4344	+	+	+
Cryobacterium_aureum	+	+	+
Sphingobacterium_mizutaii	+	+	+
Pseudoalteromonas_sp._'520P1_No._423'	+	+	+
Sorangium_cellulosum	+	+	+
Bacillaceae_bacterium_G1	+	+	+
Parapedobacter_sp.	+	+	+
Lysinibacillus_acetophenoni	+	+	+
Bacteroides_sp._AF36-11BH	+	+	+
Candidatus_Dactylopiibacterium_carminicum	+	+	+
Candidatus_Desulforudis_audaxviator	+	+	+
[Eubacterium]_brachy	+	+	+
Lactobacillus_satsumensis	+	+	+
Collinsella_sp._CAG:398	+	+	+
Streptomyces_sp._PsTaAH-124	+	+	+
Aquimarina_pacifica	-	+	+
Clostridium_sp._OM07-9AC	+	+	+
Rhizobium_leguminosarum	+	+	+
Clostridium_sp._CAG:533	+	+	+
Bacteroidetes_bacterium_GWA2_40_15	+	+	+
Carnobacterium_sp._CP1	+	+	+
Enterococcus_malodoratus	+	-	+
Oscillatoriales_cyanobacterium_MTP1	-	-	+
Ruminococcus_sp._TF10-6	-	-	+
Bacillus_sp._YR335	+	+	+
Bifidobacterium_thermophilum	+	+	+
Marinomonas_pollencensis	+	+	+
Paenibacillus_aquistagni	+	+	+
Lactobacillus_sp._YK43	+	+	+
Lactobacillus_tucceti	+	+	+
Paenibacillus_sp._KCOM_3021	+	+	+
Chlorobium_phaeobacteroides	+	+	+
Smithella_sp._D17	+	+	+
Burkholderia_cenocepacia	+	+	+
Photobacterium_aphoticum	+	+	+
Coprobacillus_sp._AF09-1A	+	+	+

Akkermansia_glycaniphila	+	+	+
Anaerococcus_obesiensis	+	+	+
Phycisphaerae_bacterium_SM23_33	+	+	+
Bacteroides_stercoris_CAG:120	+	+	+
Coprococcus_sp._CAG:131_42_139	+	+	+
Marivirga_lumbricoides	+	+	+
Candidatus_Schekmanbacteria_bacterium	+	+	+
Epulopiscium_sp._AS2M-Bin001	+	+	+
Lysinibacillus_composti	+	+	+
Candidatus_Pacearchaeota_archaeon	+	+	+
Geminocystis_sp._NIES-3709	+	+	+
Desulfotomaculum_sp._46_296	+	+	+
Fibrobacter_sp._UWB11	+	+	+
Paenibacillus_cellulosilyticus	+	+	+
Flavobacterium_chilense	-	+	+
Trueperella_bialowiezensis	-	+	+
Acidaminococcus_sp._AM05-11	+	+	+
Firmicutes_bacterium_HGW-Firmicutes-13	+	+	+
Chitinophagaceae_bacterium_N24	-	+	+
Desulfofundulus_thermosubterraneus	+	+	+
Leptotrichia_sp._oral_taxon_847	+	+	+
Marinifilum_breve	+	+	+
Olsenella_sp._HMSC062G07	+	+	+
Chitinophaga_sp._T22	+	+	+
Gordonibacter_faecihominis	+	+	+
Xanthomonas_oryzae	+	+	+
Pontibacter_roseus	+	+	+
Polaromonas_sp._OV174	+	+	+
Runella_sp._SP2	+	+	+
Bacillus_oleronius	-	+	+
Paenibacillus_sp._7197	+	+	+
Clostridium_sp._CAG:762	+	+	+
Candidatus_Saccharibacteria_bacterium_GW2011_GWC2_44_1	+	+	+
Algoriphagus_terrigena	+	+	+
Acuticoccus_sp._PTG4-2	+	+	+
Mucilaginibacter_gracilis	+	+	+
Gordonibacter_sp._Marseille-P4307	+	-	+
Halanaerobium_kushneri	+	-	+
Pantoea_sp._Ae16	-	-	+
Turicibacter_sp._H121	-	-	+
Bacillus_drentensis	+	+	+
Bacillus_foraminis	+	+	+
Burkholderia_multivorans	+	+	+
Kyrpidia_tusciae	+	+	+
Bacillus_virus_JL	+	+	+
Paenibacillus_sp._DCT19	+	+	+
Paenibacillus_nanensis	+	+	+
Syntrophorhabdus_sp._PtaB.Bin184	+	+	+
Clostridia_bacterium_UC5.1-1C12	+	+	+
Ileibacterium_massiliense	+	+	+
Micrococcales_bacterium	+	+	+
uncultured_bacterium_34R1	+	+	+
Spirochaetales_bacterium	+	+	+
Paenibacillus_zanthoxyli	+	+	+
Paraclostridium_benzoelyticum	+	+	+

Desmospora_activa	+	+	+
Bacteroides_sp._AM23-18	+	+	+
Candidatus_Izimaplasma_sp._ZiA1	+	+	+
Alkalibacterium_olivapovliticus	+	+	+
Paenibacillus_castaneae	+	+	+
Sporosarcina_globispora	+	+	+
Clostridium_sp._OF10-22XD	+	+	+
Methanomassiliicoccales_archaeon_PtaB.Bin134	+	+	+
Flavobacterium_sp._MEBiC07310	+	+	+
Carboxydocella_thermautotrophica	+	+	+
Thermogutta_terrifontis	+	+	+
Lysinibacillus_sp._B2A1	-	+	+
Lysinibacillus_fusiformis	+	+	+
Bacillus_sp._SJS	+	+	+
candidate_division_TA06_bacterium	-	+	+
Bacteroides_sp._3_1_19	+	+	+
candidate_division_Zixibacteria_bacterium	+	+	+
Phage_DP-2017a	+	+	+
Limnohabitans_sp._Hippo4	+	+	+
Bacillus_sp._FJAT-26652	+	+	+
Flavobacterium_fluvii	+	+	+
Firmicutes_bacterium_ZCTH02-B6	+	+	+
Acidithiobacillales_bacterium_SM1_46	+	+	+
candidate_division_KSB3_bacterium	+	+	+
Bacillus_sp._AFS006103	+	+	+
Pedobacter_sp.	+	+	+
Carboxydothermus_hydrogenoformans	+	+	+
Muricauda_sp._40DY170	+	+	+
Enterococcus_gilvus	+	+	+
Paenibacillus_forsythiae	+	+	+
Sulfurimonas_sp.	+	+	+
Bacillus_badius	+	+	+
Tenericutes_bacterium_HGW-Tenericutes-3	+	+	+
Filimonas_sp._YR581	+	+	+
Rhizobium_arenae	+	+	+
Cohnella_sp._AR92	+	+	+
Olsenella_sp._An270	+	+	+
Clostridium_sp._HMSC19A10	+	+	+
Podoviridae_sp.	+	+	+
Streptococcus_criceti	+	+	+
Candidatus_Saccharibacteria_oral_taxon_TM7x	-	+	+
[Eubacterium]_nodatum	+	+	+
Paenibacillus_sp._UNC451MF	+	+	+
Anaerolineae_bacterium_CG2_30_64_16	+	-	+
Acetohalobium_arabaticum	+	-	+
Jeotgalicoccus_psychrophilus	+	-	+
bacterium_BMS3Abin06	+	-	+
Plantibacter_flavus	+	-	+
Rhodoferax_antarcticus	-	-	+
Mitsuokella_sp._AF33-22	+	+	+
Vibrio_parahaemolyticus	+	+	+
Caldicellulosiruptor_danielii	+	+	+
Enterococcus_casseliflavus	+	+	+
Streptococcus_ovis	+	+	+
Bacillus_persicus	+	+	+

Lactococcus_plantarum	+	+	+
Bacillus_sp._OV166	+	+	+
Lactobacillus_pasteurii	+	+	+
Mycoplasma_sp._CAG:956	+	+	+
Planctomycetes_bacterium_GWF2_42_9	+	+	+
Novibacillus_thermophilus	+	+	+
uncultured_bacterium_Contig1773	+	+	+
Thermovenabulum_gondwanense	+	+	+
Brevibacillus_gelatini	+	+	+
Azospirillum_brasilense	+	+	+
Lebetimonas_natsushimae	-	+	+
Epulopiscium_sp._'N.t._morphotype_B'	+	+	+
Catenibacterium_sp._co_0103	+	+	+
Synergistetes_bacterium_HGW-Synergistetes-1	+	+	+
Treponema_medium	+	+	+
Epulopiscium_sp._SCG-B11WGA-EpuloA1	+	+	+
Cloacibacterium_normanense	+	+	+
Lysinibacillus_contaminans	+	+	+
Paenibacillus_thermophilus	+	+	+
Paenibacillus_senegalensis	+	+	+
Dysgonomonas_sp._37-18	+	+	+
candidate_division_WWE3_bacterium_CG22_combo_CG10-13	+	+	+
Porphyromonas_sp._oral_taxon_279	+	+	+
Bacillus_sp._J37	+	+	+
Paenibacillus_sp._A3	+	+	+
Paenibacillus_swuensis	+	+	+
Paenibacillus_sp._cl123	+	+	+
Paenibacillus_taiwanensis	+	+	+
Paenibacillus_prosopidis	+	+	+
Leptospira_noguchii	+	+	+
Lactobacillus_phage_LpeD	+	+	+
Coprobacillus_sp._AM18-4LB-d2	+	+	+
Halothermothrix_orenii	+	+	+
Anaerococcus_lactolyticus	+	+	+
Flavobacterium_frigoris	+	+	+
Ornithinibacillus_scapharcae	+	+	+
Blautia_sp._CAG:37	+	+	+
Alcaligenaceae_bacterium	+	+	+
Mesorhizobium_sp._F7	+	+	+
Terrimicrobium_sacchariphilum	+	+	+
Coriobacteriales_bacterium_DNF00809	+	+	+
Candidatus_Cryosericum_septentrionale	+	+	+
Desulfotalea_sp.	+	+	+
Tumebacillus_permanentifrigoris	+	+	+
Porphyromonas_sp._HMSC077F02	+	+	+
Cycloclasticus_sp._symbiont_of_Bathymodiolus_heckerae	+	+	+
Falsibacillus_pallidus	-	+	+
Flavobacterium_alvei	+	+	+
Tatlockia_micdadei	+	+	+
Paenibacillus_camerounensis	+	+	+
Peptostreptococcus_sp._MV1	+	+	+
Mogibacterium_pumilum	+	+	+
Pseudodesulfovibrio_hydrargyri	-	+	+
Marinobacter_hydrocarbonoclasticus	+	+	+
Bordetella_genomosp._9	+	+	+

Lacibacter_sp._TTM-7	+	+	+
Bacillus_gottheilii	+	+	+
Flavobacterium_endophyticum	+	-	+
Paenibacillus_sp._GM2	+	-	+
uncultured_bacterium_Contigcl_1149	-	-	+
Streptomyces_sp._840.1	-	-	+
Bifidobacteriaceae_bacterium_NR021	-	-	+
Basidiobolus_meristosporus	+	+	+
Megasphaera_massiliensis	+	+	+
Clostridium_sp._CAG:768	+	+	+
Paenibacillus_sophorae	+	+	+
Lachnospiraceae_bacterium_AM25-27	+	+	+
Massiliomicrobiota_sp._An142	+	+	+
Selenomonas_infelix	+	+	+
Elusimicrobia_bacterium_RIFOXYA2_FULL_40_6	-	+	+
Trichococcus_collinsii	+	+	+
Clostridium_sp._AF15-31	+	+	+
Bifidobacterium_lemurum	+	+	+
Ignavibacteria_bacterium_GWB2_35_6b	+	+	+
Candidatus_Vogelbacteria_bacterium_CG10_big_fil_rev_8_21_	-	+	+
Ruminococcus_sp._TF08-4	+	+	+
Bacteroides_intestinalis_CAG:315	+	+	+
Geomicrobium_sp._JCM_19039	+	+	+
Agrobacterium_sp._SUL3	+	+	+
Candidatus_Nephrothrix_sp._EaCA	+	+	+
Desulfamplus_magnetovallimortis	+	+	+
Alysiella_crassa	+	+	+
Erysipelothrix_rhusiopathiae	+	+	+
Sphingobacterium_sp._ML3W	+	+	+
Flavobacterium_caeni	+	+	+
Victivallales_bacterium_CCUG_44730	+	+	+
Erysipelotrichaceae_bacterium_OH741_COT-311	+	+	+
Ferrovum_myxofaciens	+	+	+
Tenericutes_bacterium_GWD2_38_27	+	+	+
Candiadtus_Scalindua_japonica	+	+	+
Bacteroides_sp._41_26	+	+	+
Virgibacillus_soli	+	+	+
Bacillus_pseudofirmus	+	+	+
Candidatus_Bacteroides_periocalifornicus	-	+	+
Candidatus_Dorea_massiliensis	+	+	+
Ruminococcus_sp._OM07-17	+	+	+
Elizabethkingia_ursingii	+	+	+
Lysinibacillus_mangiferihumi	+	+	+
Arsenicibacter_rosenii	+	+	+
Desulfosarcina_cetonica	+	+	+
Pseudodesulfovibrio_piezophilus	+	+	+
Acidiferrobacter_sp._SPIII_3	+	+	+
Aminobacterium_mobile	+	+	+
Cnuella_takakiae	+	+	+
Bacillus_sp._M5HDSG1-1	+	+	+
Gordonibacter_sp._An232A	+	+	+
Romboutsia_ilealis	+	+	+
Salmonella_phage_Astrid	-	+	+
Muribaculaceae_bacterium_Isolate-084_(Janvier)	+	+	+
Clostridium_sp._CAG:524	+	+	+

Oceanobacillus_manasiensis	+	+	+
Meiothermus_granaticius	+	+	+
Enterococcus_sp._4G2_DIV0659	+	+	+
Sphingomonas_sp._Ant_H11	-	+	+
Peptoniphilus_harei	+	+	+
Methanocaldococcus_villosus	+	+	+
Carnobacterium_sp._WN1374	-	+	+
Cryptobacterium_curtum	+	-	+
Mycoplasma_meleagridis	-	-	+
Chromobacterium_rhizoryzae	-	-	+
Gracilibacillus_dipsosauri	+	+	+
Parabacteroides_sp._CAG:2	+	+	+
Tetragenococcus_halophilus	+	+	+
Aneurinibacillus_terranovensis	+	+	+
Paenibacillus_bouchesdurhonensis	+	+	+
Arthrobacter_stackebrandtii	+	+	+
Klebsiella_aerogenes	+	+	+
Clostridiales_bacterium_Marseille-P2986	+	+	+
Euryarchaeota_archaeon	+	+	+
Ammonifex_degensii	+	+	+
Paenibacillus_sp._GP183	+	+	+
Enterococcus_timonensis	+	+	+
Desulfovibrio_sp._FW1012B	+	+	+
Clostridium_sartagoforme	+	+	+
Olsenella_sp._KH3B4	+	+	+
Sporolactobacillus_sp._THM7-7	+	+	+
Brevefilum_fermentans	+	+	+
Methanofollis_ethanolicus	+	+	+
unidentified_microorganism	+	+	+
[Eubacterium]_saphenum	+	+	+
Thiorhodovibrio_sp._970	+	+	+
Rhodopseudomonas_palustris	+	+	+
Ruminococcus_sp._TF11-2AC	+	+	+
Candidatus_Fluviicola_riflensis	+	+	+
Paenibacillus_sp._BC26	+	+	+
Psychroserpens_damuponensis	-	+	+
Ruminococcus_sp._AF21-11	+	+	+
Candidatus_Woesebacteria_bacterium_RBG_16_40_11	+	+	+
Parageobacillus_genomosp._1	+	+	+
Moorella_glycerini	+	+	+
Capnocytophaga_ochracea	+	+	+
Bacillus_dielmoensis	+	+	+
Afipia_sp._1NLS2	+	+	+
Paenibacillus_stellifer	+	+	+
Staphylococcus_hominis	+	+	+
Thiotrichaceae_bacterium_IS1	-	+	+
Prevotella_sp._P3-120	+	+	+
Vagococcus_salmoninarum	+	+	+
Moorella_stamsii	+	+	+
Pedobacter_namyangjuensis	+	+	+
Paenibacillus_apiarius	+	+	+
Azovibrio_restrictus	+	+	+
Cystobacter_fuscus	+	+	+
Helcococcus_sueciensis	+	+	+
Enterococcus_massiliensis	+	+	+

Desulfotomaculum_aquiferis	+	+	+
Selenomonas_sp._FOBRC6	+	+	+
Bacillus_sp._VT-16-64	+	+	+
Bacteroides_sp._AF25-5LB	+	+	+
Bacillus_dakarensis	+	+	+
bacterium_BMS3Bbin14	+	+	+
Desulfovibrio_ferrireducens	+	+	+
Gallionellales_bacterium_GWA2_60_142	+	+	+
Chloroflexi_bacterium_RBG_16_57_11	-	+	+
Mycoplasma_sp._CAG:472	+	+	+
Methanobrevibacter_smithii	+	+	+
Candidatus_Saccharibacteria_bacterium_TM7__EAM_G5_1_H[illegible]	-	+	+
Kazachstania_naganishii	+	+	+
Ruminococcus_sp._AM47-2BH	+	+	+
Leptolinea_tardivitalis	+	+	+
bacterium_AMD02	-	+	+
Candidatus_Saccharibacteria_bacterium_RAAC3_TM7_1	-	+	+
Sulfurivermis_fontis	+	-	+
Enterococcus_sp._6C8_DIV0013	+	-	+
Mediterraneibacter_sp._gm002	+	-	+
Fusobacterium_sp._oral_taxon_370	+	-	+
alpha_proteobacterium_LLX12A	-	-	+
Chloroflexi_bacterium_HGW-Chloroflexi-6	+	+	+
Bifidobacterium_breve	+	+	+
Lentisphaerae_bacterium_GWF2_49_21	+	+	+
Bacillus_massilionigeriensis	+	+	+
Paenibacillus_crassostreae	+	+	+
Facklamia_hominis	+	+	+
Prevotella_sp._HJM029	+	+	+
Megasphaera_sp._DJF_B143	+	+	+
Thermotoga_petrophila	+	+	+
Capsulimonas_corticalis	+	+	+
Exiguobacterium_phage_vB_EalM-132	+	+	+
Anaerosphaera_sp._GS7-6-2	+	+	+
Alcanivorax_pacificus	+	+	+
Bacillus_mesonae	+	+	+
Bdellovibrio_sp._SKB1291214	+	+	+
Verrucomicrobium_spinosum	+	+	+
Bacillus_sp._FJAT-27986	+	+	+
Lentisphaerae_bacterium_RIFOXYA12_64_32	+	+	+
Erysipelotrichaceae_bacterium_AF19-24AC	+	+	+
Spirulina_major	+	+	+
Lysinibacillus_sp._FJAT-14222	+	+	+
Caldalkalibacillus_thermarum	+	+	+
Prevotella_sp._oral_taxon_317	+	+	+
Prevotella_sp._oral_taxon_306	+	+	+
Acidobacteria_bacterium_13_1_20CM_3_53_8	+	+	+
Erysipelotrichaceae_bacterium_2_2_44A	+	+	+
Paenibacillus_sp._St-s	+	+	+
Candidatus_Atribacteria_bacterium_RBG_19FT_COMBO_35_[illegible]	-	+	+
Geoalkalibacter_ferrihydriticus	+	+	+
Taibaiella_koreensis	+	+	+
Streptococcus_azizii	+	+	+
Leminorella_grimontii	+	+	+
Paenibacillus_etheri	+	+	+

Prevotella_sp._RS2	+	+	+
Tenacibaculum_ovolyticum	+	+	+
Tamlana_nanhaiensis	+	+	+
Paenibacillus_anaericanus	+	+	+
Lysinibacillus_xyleni	+	+	+
Candidatus_Latescibacteria_bacterium	+	+	+
Caenispirillum_bisanense	+	+	+
Chloroflexi_bacterium_RBG_16_64_32	+	+	+
Arcobacter_cloacae	+	+	+
Ktedonobacter_racemifer	+	+	+
Myroides_odoratus	+	+	+
Paenibacillus_sp._Aloe-11	+	+	+
Fibrobacter_sp._UWS3	-	+	+
Pedobacter_sp._PACM_27299	+	+	+
Paraburkholderia_phenazinium	+	+	+
Sinirhodobacter_sp._D19-10-3-21	+	+	+
Thermotoga_sp._Mc24	+	+	+
Candidatus_Omnitrophica_bacterium_CG1_02_43_210	-	+	+
Candidatus_Saccharibacteria_bacterium_32-50-13	+	+	+
Turicibacter_sp._HGF1	+	+	+
Streptococcus_sp._45	-	+	+
Persephonella_sp.	+	+	+
Corallococcus_sp._AB032C	+	+	+
Streptococcus_sp._oral_taxon_071	+	+	+
Paenibacillus_sp._MBLB1234	+	+	+
Candidatus_Atribacteria_bacterium_RBG_16_35_8	-	+	+
Arcobacter_pacificus	-	+	+
Clostridium_combesii	+	+	+
Coprobacillus_sp._OM08-19	+	+	+
Meiothermus_timidus	+	+	+
Atribacteria_bacterium_JGI_0000014-F07	-	+	+
Aliivibrio_fischeri	+	-	+
Edwardsiella_piscicida	+	-	+
Serratia_sp._M24T3	-	-	+
Deltaproteobacteria_bacterium_RIFCSPHIGHO2_01_FULL_43	-	-	+
Kroppenstedtia_eburnea	-	-	+
Lactococcus_garvieae	+	+	+
Spizellomyces_punctatus	+	+	+
Woolly_monkey_sarcoma_virus	+	+	+
Butyricicoccus_sp._AM29-23AC	+	+	+
Lactobacillus_nagelii	+	+	+
Megasphaera_sp._Marseille-P4857	+	+	+
Paenibacillus_herberti	-	+	+
Coprobacillus_sp._AF31-1BH	+	+	+
Paenibacillus_chibensis	+	+	+
Desulfobulbus_sp._Tol-SR	+	+	+
Helicobacter_hepaticus	-	+	+
Nitratiruptor_sp._SB155-2	+	+	+
Chryseobacterium_bovis	+	+	+
Ruminococcus_faecis	+	+	+
Treponema_sp._GWA1_62_8	+	+	+
Bacillus_sp._7504-2	+	+	+
Megasphaera_sp._UPII_135-E	+	+	+
Archangium_violaceum	+	+	+
Succinivibrio_sp.	+	+	+

Bacillus_bogoriensis	+	+	+
Turneriella_parva	+	+	+
Flectobacillus_major	+	+	+
Formosa_sp._Hel1_33_131	+	+	+
Desulfobacteraceae_bacterium_A6	-	+	+
Erwinia_sp._198	-	+	+
Cyclobacterium_qasimii	+	+	+
Lachnoanaerobaculum_orale	+	+	+
Rhodanobacter_sp._B04	+	+	+
Streptomyces_vitaminophilus	+	+	+
candidate_division_TM6_bacterium_GW2011_GWF2_33_332	+	+	+
Nitrospirae_bacterium_GWD2_57_8	+	+	+
Rhodobacteraceae_bacterium_TMED111	+	+	+
Psychrobacillus_sp._OK028	+	+	+
Chryseobacterium_sp._Leaf405	+	+	+
Effusibacillus_pohliae	+	+	+
Desulfurispirillum_indicum	+	+	+
Candidatus_Gastranaerophilales_bacterium_HUM_3	+	+	+
Spirochaetes_bacterium_DG_61	+	+	+
Pedobacter_africanus	+	+	+
Chitinophaga_filiformis	+	+	+
Desulfonatronum_thiosulfatophilum	+	+	+
Echinicola_strongylocentroti	+	+	+
Desulfarculus_baarsii	+	+	+
Desemzia_incerta	+	+	+
Bifidobacterium_asteroides	+	+	+
Bosea_sp._TND4EK4	+	+	+
Niastella_yeongjuensis	+	+	+
Dialister_micraerophilus	+	+	+
Sphingopyxis_sp._113P3	-	+	+
Bacillus_sp._SKP7-4	+	+	+
Sphingosinicella_sp._CPCC_101087	+	+	+
Nitrosomonas_sp._Nm33	+	+	+
Carnobacterium_divergens	-	+	+
Polaribacter_butkevichii	+	+	+
Prevotella_conceptionensis	+	+	+
Paenibacillus_sp._FSL_H7-0326	+	+	+
Caedibacter_sp._38-128	+	+	+
Paenibacillus_wynnii	-	+	+
Oceanospirillales_bacterium_LUC14_002_19_P2	-	+	+
Clostridium_sp._AF23-6LB	-	+	+
Candidatus_Saccharibacteria_bacterium_TM7_ANC_38.39_G1_	-	+	+
Clostridium_sp._AM28-20LB	+	+	+
Chitinophaga_rupis	+	+	+
Vibrio_phage_BONAISHI	+	+	+
Olsenella_sp._An293	+	+	+
Ruminococcus_sp._AM29-12LB	+	-	+
Paenibacillus_sp._OK076	+	-	+
Comamonadaceae_bacterium	+	-	+
Brumimicrobium_aurantiacum	+	-	+
archaeon_GW2011_AR4	-	-	+
Staphylococcus_capitis	-	-	+
Klebsiella_phage_1611E-K2-1	-	-	+
Anabaena_sp._AL93	-	-	+
Paenibacillus_sp._PDC88	+	+	+

Paraglaciecola_arctica	+	+	+
Lutibacter_sp._1KV19	+	+	+
Cyanothece_sp._PCC_7822	+	+	+
Dialister_invisus_CAG:218	+	+	+
Bacillus_virus_Shanette	+	+	+
Ignavibacteriales_bacterium_CG18_big_fil_WC_8_21_14_2_50	+	+	+
Nocardioides_sp._Soil805	+	+	+
Faecalibacterium_phage_FP_Epona	+	+	+
Actinobacillus_ureae	+	+	+
Streptococcus_equi	+	+	+
Ruminococcus_sp._AM22-14LB	+	+	+
Selenihalanaerobacter_shriftii	+	+	+
Peptoniphilus_grossensis	+	+	+
Selenomonas_sp._oral_taxon_137	+	+	+
Bacillus_sp._HMF5848	+	+	+
Thermobacillus_composti	+	+	+
Bacillus_praedii	+	+	+
Paraburkholderia_tropica	+	+	+
Thermoanaerobacterium_sp._RBIITD	+	+	+
Bacillus_sp._SAJ1	+	+	+
Ralstonia_solanacearum	+	+	+
Brevibacillus_sp._NRRL_NRS-1210	+	+	+
Peptoniphilus_sp._ING2-D1G	+	+	+
candidate_division_Zixibacteria_bacterium_SM23_81	+	+	+
Sphingobacterium_cellulitidis	+	+	+
Paenibacillus_pini	+	+	+
Bacillus_sp._UNC41MFS5	+	+	+
Thermodesulforhabdus_norvegica	+	+	+
Pseudomonas_saponiphila	+	+	+
Pseudomonas_pelagia	+	+	+
Porphyromonas_endodontalis	+	+	+
Desulfuribacillus_stibiiarsenatis	+	+	+
Paenibacillus_typhae	+	+	+
Niabella_aurantiaca	+	+	+
Flavobacteriaceae_bacterium_CG1_02_35_72	+	+	+
Candidatus_Frackibacter_sp._T328-2	+	+	+
Listeria_seeligeri	+	+	+
Streptococcus_sinensis	-	+	+
Porphyromonas_circumdentaria	+	+	+
Rhodoblastus_sphagnicola	+	+	+
Roseivirga_ehrenbergii	+	+	+
Corallococcus_sp._CA040B	+	+	+
Fibrobacter_sp._UWT3	+	+	+
Brevibacillus_sp._SCSIO_07484	+	+	+
Candidatus_Saccharibacteria_bacterium_32-50-10	-	+	+
Candidatus_Aegiribacteria_bacterium_MLS_C	+	+	+
Halomonas_sp._KM-1	+	+	+
Ornatilinea_apprima	+	+	+
Bartonella_apis	+	+	+
Candidatus_Methanomethylophilus_alvus	+	+	+
Candidatus_Altiarchaeales_archaeon_ex4484_43	-	+	+
Bordetella_pseudohinzii	+	+	+
candidate_division_WS5_bacterium	+	+	+
Oceanobacillus_oncorhynchi	+	+	+
Bacillus_sp._B-jedd	+	+	+

Thauera_propionica	+	+	+
Spirochaetes_bacterium_RBG_13_68_11	+	+	+
Mycoplasma_fermentans	+	+	+
Mycobacterium_chimaera	+	+	+
Draconibacterium_orientale	+	+	+
Geobacillus_sp._8	+	+	+
Lactobacillus_gallinarum	+	+	+
Paludibacterium_yongneupense	+	+	+
Enterococcus_sp._12F9_DIV0723	+	+	+
Hadesarchaea_archaeon_YNP_N21	-	+	+
Bacillus_sp._FJAT-45385	+	+	+
Myroides_guanonis	+	+	+
Chromohalobacter_japonicus	-	+	+
Sphingomonas_haloaromaticamans	-	+	+
Chitinophaga_skermanii	+	+	+
Olsenella_profusa	+	+	+
Aquisalibacillus_elongatus	+	+	+
Paenibacillus_sp._VT-400	-	+	+
Blautia_sp._OF03-13	+	+	+
Paenibacillus_sp._FSL_R5-0912	+	-	+
Thermotoga_sp._47_83	+	-	+
Collinsella_sp._AM20-15AC	+	-	+
Burkholderia_sp._D7	+	-	+
Spirochaeta_africana	-	-	+
Aminobacterium_colombiense	-	-	+
Candidatus_Jorgensenbacteria_bacterium_CG23_combo_of_CG	-	-	+
Murine_AIDS_virus-related_provirus	+	+	+
Desulfoluna_spongiiphila	+	+	+
Fibrobacter_sp._UWR3	+	+	+
Ruminococcus_sp._AM32-17LB	+	+	+
Jimgerdemannia_flammicorona	-	+	+
Ruminococcus_sp._AF16-40	+	+	+
Parvimonas_sp._oral_taxon_393	-	+	+
Actinobacteria_bacterium_HGW-Actinobacteria-6	+	+	+
Tumebacillus_algifaecis	+	+	+
Exiguobacterium_arabatum	-	+	+
Thermobacillus_sp.	+	+	+
Pasteurella_langaaensis	+	+	+
Helicobacter_pullorum	+	+	+
Epulopiscium_sp._SCG-C06WGA-EpuloA1	+	+	+
Smithella_sp._PtaU1.Bin162	-	+	+
Oceanobacillus_sp._160	+	+	+
Paenibacillus_larvae	+	+	+
Desulfovermiculus_halophilus	+	+	+
Peptoniphilus_phoceensis	+	+	+
Marinococcus_halophilus	+	+	+
Chlamydiae_bacterium_SM23_39	+	+	+
Treponema_succinifaciens	+	+	+
Salinivirga_cyanobacteriivorans	+	+	+
Candidatus_Paracaedibacter_acanthamoebae	-	+	+
Pseudoclavibacter_faecalis	+	+	+
Hymenobacter_sedentarius	+	+	+
Sphingobacterium_psychroaquaticum	+	+	+
Paenibacillus_sp._FSL_H7-689	+	+	+
Acidobacteriaceae_bacterium_TAA166	+	+	+

Prevotella_falsenii	+	+	+
Tissierellia_bacterium_S7-1-4	+	+	+
Olsenella_provencensis	+	+	+
Candidatus_Atribacteria_bacterium_CG2_30_33_13	+	+	+
Citricoccus_massiliensis	+	+	+
Maribacter_orientalis	+	+	+
Pasteurellaceae_bacterium_15-036681	+	+	+
Fusobacterium_sp._CM21	+	+	+
Bacillus_aryabhattai	+	+	+
Candidatus_Accumulibacter_sp._SK-01	+	+	+
Lactococcus_piscium	-	+	+
Neofamilia_massiliensis	+	+	+
Agarilytica_rhodophyticola	+	+	+
Solibacillus_isronensis	+	+	+
Nitrospirae_bacterium_GWC2_42_7	-	+	+
Pseudoxanthomonas_sp._GM95	+	+	+
Chelativorans_sp._J32	+	+	+
Ignavibacteria_bacterium_GWF2_33_9	+	+	+
Anaerococcus_nagyae	+	+	+
Azospirillum_lipoferum	-	+	+
Labilibacter_aurantiacus	+	+	+
Spirochaetes_bacterium_GWB1_59_5	+	+	+
Paenibacillus_pasadenensis	+	+	+
Oligella_urethralis	+	+	+
Trichococcus_ilyis	+	+	+
Succinivibrionaceae_bacterium_WG-1	+	+	+
Methanosarcina_barkeri	+	+	+
Melissococcus_plutonius	+	+	+
Azotobacter_vinelandii	-	+	+
Acidaminococcus_sp._CAG:542	+	+	+
Chryseobacterium_jejuense	+	+	+
Paenibacillus_popilliae	+	+	+
Thermoactinomyces_sp._Gus2-1	+	+	+
Veillonella_sp._VA139	+	+	+
Arsenicitalea_aurantiaca	-	+	+
Streptococcus_vestibularis	+	+	+
Sandaracinus_amylolyticus	+	+	+
Dialister_sp._Marseille-P5638	+	+	+
Myroides_phaeus	+	+	+
Brevibacillus_choshinensis	+	+	+
Photobacterium_angustum	+	+	+
Paraliobacillus_sp._X-1174	+	+	+
Vibrio_phage_1.013.O._10N.286.54.F9	+	+	+
Microgenomates_group_bacterium_GW2011_GWC1_38_12	-	+	+
Streptococcus_iniae	+	-	+
Azohydromonas_australica	+	-	+
Flavobacteriales_bacterium_TMED113	+	-	+
Peptoniphilus_sp._Marseille-P3761	+	-	+
Spirochaetes_bacterium_GWF1_51_8	+	-	+
Campylobacter_cuniculorum	+	-	+
Morganella_morganii	+	-	+
Streptomyces_phage_Gilson	-	-	+
Pontibacillus_yanchengensis	-	-	+
Ruminococcus_sp._AM28-13	+	+	+
Paenibacillus_dendritiformis	+	+	+

Nostoc_sp._HK-01	+	+	+
Coprobacillus_sp._TF10-10	+	+	+
Veillonella_sp._AF13-2	+	+	+
Peptococcaceae_bacterium	+	+	+
Trichococcus_flocculiformis	-	+	+
Riemerella_anatipestifer	+	+	+
Bacillus_marisflavi	+	+	+
uncultured_bacterium_Ad_125_D08	+	+	+
Peptoclostridium_litorale	+	+	+
Romboutsia_sp._MT17	+	+	+
Thioflexothrix_psekupsii	+	+	+
Bacillus_sp._SA5d-4	+	+	+
Flavobacterium_hydatis	+	+	+
Pedobacter_xixiisoli	+	+	+
Syntrophobacteraceae_bacterium_CG2_30_61_12	+	+	+
Alicyclobacillus_macrosporangiidus	+	+	+
Caldicellulosiruptor_changbaiensis	+	+	+
Peptoniphilus_urinimassiliensis	+	+	+
Mitsuokella_multacida	+	+	+
uncultured_bacterium_Ad_125_H07_contig1	+	+	+
Paenibacillus_sp._7884-2	+	+	+
Sutterella_sp._CAG:521	+	+	+
Pandoraea_sp.	+	+	+
Psychroserpens_sp._Hel_I_66	+	+	+
Aliiglaciecola_lipolytica	+	+	+
Bifidobacterium_pullorum	+	+	+
Flavobacterium_anhuiense	+	+	+
Phyllobacteriaceae_bacterium_Z3-1	+	+	+
Sphingobacterium_spiritivorum	+	+	+
Chryseobacterium_carnis	+	+	+
Thermaerobacter_subterraneus	+	+	+
Prevotella_sp._HMSC073D09	+	+	+
Elizabethkingia_occulta	+	+	+
Dysgonamonadaceae_bacterium	+	+	+
Atopobium_sp._ICM42b	+	+	+
Macromonas_sp._BK-30	-	+	+
Alkalispirochaeta_americana	+	+	+
Candidatus_Arthromitus_sp._SFB-3	-	+	+
Andreesenia_angusta	+	+	+
Bacteroidetes_bacterium_RBG_13_42_15	+	+	+
Neiella_marina	+	+	+
Porphyromonas_sp._COT-052_OH4946	+	+	+
Candidatus_Izimaplasma_sp._HR2	+	+	+
Acidihalobacter_prosperus	+	+	+
Salinarimonadaceae_bacterium_HL-109	+	+	+
Terrabacter_sp._Root85	+	+	+
Ruminococcus_sp._YE71	+	+	+
Lactobacillus_vini	+	+	+
Kiritimatiella_glycovorans	+	+	+
Candidatus_Izimaplasma_sp._HR1	+	+	+
Burkholderiales_bacterium_RIFCSPLOWO2_12_FULL_61_40	+	+	+
Bacillus_fordii	+	+	+
Candidatus_Margulisbacteria_bacterium_GWD2_39_127	+	+	+
Bacteroides_sp._Ga6A2	+	+	+
Lutibacter_maritimus	+	+	+

Desulfobacter_curvatus	+	+	+
Methanobrevibacter_cuticularis	+	+	+
Solirubrobacter_soli	-	+	+
Streptococcus_porci	+	+	+
Thermanaerovibrio_velox	+	+	+
Leptospira_sp._E30	-	+	+
Streptococcus_salivarius	+	+	+
Pseudomonas_sp._WN033	+	+	+
Arcobacter_skirrowii	+	+	+
Roseomonas_deserti	+	+	+
Pseudomonas_sp._MWU12-2115	+	+	+
Thalassospira_sp._11-3	+	+	+
Mucilaginibacter_gotjawali	+	+	+
Pseudodesulfovibrio_aespoeensis	+	+	+
Citricoccus_muralis	+	+	+
Staphylococcus_lugdunensis	-	+	+
Ruminococcus_sp._AM12-48	+	+	+
Bacteroidia_bacterium	+	+	+
Paenibacillus_sp._O199	+	+	+
Actinomyces_sp._oral_taxon_172	+	+	+
Clostridium_sp._OM04-12AA	+	+	+
Dethiosulfovibrio_peptidovorans	+	+	+
Kocuria_palustris	+	+	+
Neisseriaceae_bacterium_DSM_100970	+	+	+
Coriobacteriaceae_bacterium_BV3Ac1	+	+	+
Francisella_sp._FSC1006	+	+	+
Parcubacteria_group_bacterium_GW2011_GWC2_39_11	-	+	+
alpha_proteobacterium_Mf_1.05b.01	+	+	+
Paenibacillus_sp._OK003	-	+	+
Eggerthella_sp._CAG:368	+	-	+
Calothrix_sp._PCC_7103	+	-	+
Candidatus_Melainabacteria_bacterium_HGW-Melainabacteria-	+	-	+
Azospirillum_sp._TSH58	+	-	+
Citrobacter_amalonaticus	+	-	+
Bacillus_aquimaris	+	-	+
Lactobacillus_mixtipabuli	+	-	+
Geobacter_daltonii	+	-	+
Planococcus_sp._Urea-3u-39	-	-	+
Bifidobacterium_bombi	-	-	+
Bavariicoccus_seileri	-	-	+
Candidatus_Vogelbacteria_bacterium_CG10_big_fil_rev_8_21_	-	-	+
Desmospora_sp._8437	-	-	+
Collinsella_sp._OF02-10	-	-	+
Staphylococcus_xylosus	-	-	+
Pseudomonas_sp._2(2015)	+	+	+
Clostridium_sp._HGF2	+	+	+
Bacillus_phage_vB_BsuM-Goe2	+	+	+
Bacillus_sp._AFS088145	+	+	+
Dehalobacter_sp._TeCB1	+	+	+
Acinetobacter_sp._SFA	+	+	+
Paenibacillus_terrigena	+	+	+
Olsenella_sp._Marseille-P4559	+	+	+
Flavobacterium_sp._MEB061	+	+	+
Petrotoga_mexicana	+	+	+
Amycolatopsis_benzoatilytica	+	+	+

Chlorogloeopsis_fritschii	+	+	+
Flavobacteriaceae_bacterium_CG18_big_fil_WC_8_21_14_2_5	-	+	+
Owenweeksia_hongkongensis	+	+	+
Chryseobacterium_greenlandense	+	+	+
Bacillus_trypoxylicola	+	+	+
Bacillus_cucumis	+	+	+
Thermoanaerobacter_uzonensis	+	+	+
Sporolactobacillus_sp._THM19-2	+	+	+
Rhodothermaceae_bacterium	+	+	+
Maribacter_thermophilus	+	+	+
Bacteroides_sp._AM56-10ce	+	+	+
Gemmata_sp._SH-PL17	+	+	+
Salinimicrobium_xinjiangense	+	+	+
Paenibacillus_sp._FSL_H8-0548	+	+	+
Enterococcus_phage_LY0322	+	+	+
Dorea_longicatena_CAG:42	+	+	+
Tuberibacillus_calidus	+	+	+
Clostridiales_bacterium_KA00274	+	+	+
Tenacibaculum_sp._MAR_2009_124	+	+	+
Vibrio_breoganii	+	+	+
Limnoraphis_robusta	+	+	+
Enterococcus_sp._4E1_DIV0656	-	+	+
Tenericutes_bacterium_HGW-Tenericutes-8	+	+	+
Zobellella_endophytica	+	+	+
Acidovorax_sp._106	+	+	+
Bacillus_sp._3-2-2	+	+	+
uncultured_bacterium_fosmid_pJB69A5	+	+	+
Bifidobacterium_kashiwanohense	+	+	+
Massilia_sp._BSC265	+	+	+
Actinobacteria_bacterium_RBG_16_68_12	+	+	+
Microbacterium_esteraromaticum	+	+	+
Paraburkholderia_symbiotica	+	+	+
Pragia_sp._CF-458	+	+	+
Pueribacillus_theae	+	+	+
Pseudarcicella_sp._HME7025	+	+	+
Psychrobacillus_sp._OK032	+	+	+
Spirochaeta_sp._LUC14_002_19_P3	+	+	+
Tindallia_californiensis	+	+	+
Proteiniphilum_sp._51_7	+	+	+
Thiocapsa_roseopersicina	+	+	+
Endomicrobium_proavitum	+	+	+
Salinibacillus_kushneri	+	+	+
Bordetella_avium	+	+	+
Azospirillum_halopraeferens	+	+	+
Bacteroidetes_bacterium_HGW-Bacteroidetes-3	+	+	+
Chitinophaga_sp._GDMCC_1.1288	+	+	+
Paraburkholderia_phenoliruptrix	+	+	+
Candidatus_Achromatium_palustre	-	+	+
Candidatus_Kentron_sp._TC	+	+	+
Terrisporobacter_othiniensis	+	+	+
Candidatus_Kentron_sp._FW	+	+	+
uncultured_Burkholderia_sp.	+	+	+
Chloroflexi_bacterium_CG07_land_8_20_14_0_80_51_10	+	+	+
Streptomyces_griseoplanus	+	+	+
Actinomyces_sp._ICM58	+	+	+

Pseudomonas_protegens	+	+	+
Enterococcus_sp._9D6_DIV0238	+	+	+
Salinicola_halophilus	-	+	+
Herpetosiphon_aurantiacus	+	+	+
Collinsella_sp._CAG:166	+	+	+
Bacillus_solimangrovi	+	+	+
Coprococcus_sp._CAG:131	+	+	+
Bifidobacterium_ruminantium	+	+	+
Coprobacillus_sp._OF03-2AA	-	+	+
Deltaproteobacteria_bacterium_RIFCSPHIGHO2_02_FULL_44	+	+	+
Arachidicoccus_rhizosphaerae	+	-	+
Slackia_piriformis	+	-	+
Leptotrichia_goodfellowii	+	-	+
Bifidobacterium_saguini	+	-	+
Promicromonosporaceae_bacterium_CFH_30434	+	-	+
Chthoniobacter_flavus	-	-	+
Bacillus_massiliogabonensis	-	-	+
Brevibacillus_virus_Jenst	-	-	+
Enterococcus_phage_ECP3	-	-	+
Deinococcus_geothermalis	-	-	+
Chrysanthemum_yellows_phytoplasma	-	-	+
Legionella_maceachernii	-	-	+
Brachyspira_sp._CAG:484	+	+	+
Wolfiporia_cocos	+	+	+
Firmicutes_bacterium_AF12-30	+	+	+
Caviibacterium_pharyngocola	-	+	+
Paenibacillus_ihuae	+	+	+
Tenacibaculum_sp._DSM_106434	+	+	+
Herminiimonas_arsenitoxidans	-	+	+
Confluentibacter_citreus	+	+	+
Actinomyces_ruminicola	+	+	+
Planctomycetes_bacterium_RBG_13_50_24	+	+	+
Brevibacillus_sp._CF112	+	+	+
Candidatus_Ozemobacter_sibiricus	+	+	+
Negativicoccus_succinicivorans	-	+	+
Fibrobacter_sp._UWB7	+	+	+
Bacillus_litoralis	+	+	+
Geobacter_thiogenes	+	+	+
Lysinibacillus_sphaericus	+	+	+
Streptococcus_mutans	+	+	+
Bifidobacterium_thermacidophilum	+	+	+
[Ruminococcus]_torques_CAG:61	+	+	+
Porphyromonas_sp._HMSC065F10	+	+	+
Flavobacteriia_bacterium	+	+	+
Scytonema_millei	+	+	+
candidate_division_WOR-3_bacterium_JGI_Cruoil_03_44_89	-	+	+
Chloroflexi_bacterium_HGW-Chloroflexi-1	+	+	+
Desulfurobacterium_atlanticum	+	+	+
Deltaproteobacteria_bacterium_RBG_13_47_9	+	+	+
Gammaproteobacteria_bacterium_TMED34	+	+	+
Pseudoramibacter_alactolyticus	+	+	+
Methanobacterium_formicicum	+	+	+
Flavobacterium_ginsenosidimutans	+	+	+
Viridibacillus_sp._OK051	+	+	+
Bacteroidetes_bacterium_37-13	+	+	+

Propionibacteriaceae_bacterium_CPCC_204711	+	+	+
Lactobacillus_crispatus	+	+	+
Jeotgalibaca_sp._PTS2502	+	+	+
Ruminococcus_sp._AF19-15	+	+	+
Desulfospira_joergensenii	+	+	+
Calditerricola_satsumensis	+	+	+
Tenacibaculum_jejuense	+	+	+
Lachnoanaerobaculum_sp.	+	+	+
Nitrospirae_bacterium_CG2_30_70_394	+	+	+
Bacteroides_sp._AF25-17LB	+	+	+
Parabacteroides_sp._OF01-14	+	+	+
Pontibacter_diazotrophicus	+	+	+
Echinicola_vietnamensis	+	+	+
Dethiobacter_sp.	+	+	+
Bartonella_tribocorum	+	+	+
Chlorobaculum_parvum	+	+	+
bacterium_HR32	+	+	+
Ruminococcus_sp._AM49-10BH	+	+	+
Lactobacillus_kitasatonis	+	+	+
Legionella_drancourtii	+	+	+
Candidatus_Mycoplasma_girerdii	+	+	+
Flavobacterium_sp._LM5	+	+	+
Thermofilum_sp._NZ13	+	+	+
Tindallia_sp._MSAO_Bac2	+	+	+
Selenomonas_sp._CM52	+	+	+
Chitinophaga_eiseniae	+	+	+
Granulicella_tundricola	+	+	+
Hafnia_alvei	+	+	+
Halobacillus_massiliensis	+	+	+
Candidatus_Tectomicrobia_bacterium_RIFCSPLOWO2_02_FU	+	+	+
Rhizobium_sp._2MFCol3.1	+	+	+
Ignavibacteriae_bacterium	+	+	+
Spirosoma_sp._TY50	+	+	+
Butyricicoccus_sp._OM06-6AC	+	+	+
Coprobacillus_sp._AF15-30	+	+	+
Petrotoga_sp._9PWA.NaAc.5.4	+	+	+
Lactobacillus_casei	+	+	+
Blautia_sp._CAG:37_48_57	+	+	+
Brachyspira_suanatina	+	+	+
Raineya_orbicola	+	+	+
Oceanobacillus_sp._E9	+	+	+
Lactobacillus_nantensis	-	+	+
Halanaerobium_sp._MA284_MarDTE_T2	-	+	+
uncultured_murine_large_bowel_bacterium_BAC_14	+	+	+
Cohaesibacter_haloalkalitolerans	-	+	+
Geobacillus_thermoleovorans	-	+	+
Lactobacillus_brevis	+	+	+
Caldicellulosiruptor_saccharolyticus	+	-	+
Micromonospora_pallida	+	-	+
Fusobacterium_massiliense	+	-	+
Bifidobacterium_sp._2033B	+	-	+
Leifsonia_xyli	+	-	+
Enterococcus_ureilyticus	+	-	+
Pseudoscardovia_suis	+	-	+
Listeria_kieliensis	+	-	+

Geobacillus_sp._44C	-	-	+
Actinobacteria_bacterium_HGW-Actinobacteria-5	-	-	+
Candidatus_Saccharibacteria_bacterium_GW2011_GWC2_48_9	-	-	+
Candidatus_Jorgensenbacteria_bacterium_RIFCSPLOWO2_01_	-	-	+
Andreprevotia_lacus	-	-	+
Caldicoprobacter_faecalis	+	+	+
Deltaproteobacteria_bacterium_SM23_61	+	+	+
Candidatus_Omnitrophus_magneticus	-	+	+
Clostridium_sp._USBA_49	+	+	+
Rhodospirillales_bacterium_URHD0017	+	+	+
Arenibacter_certesii	+	+	+
Desulfobacterales_bacterium_GWB2_56_26	+	+	+
Bifidobacterium_gallinarum	+	+	+
Caldicellulosiruptor_acetigenus	+	+	+
Saccharibacillus_sp._O23	+	+	+
Puccinia_graminis	+	+	+
Olsenella_sp._An188	+	+	+
Bacillus_deserti	+	+	+
Crocosphaera_watsonii	+	+	+
Blautia_sp._OF11-22	+	+	+
Clostridium_sp._AF16-25	-	+	+
Helicobacter_mustelae	+	+	+
Ktedonobacterales_bacterium_Uno3	+	+	+
Nafulsella_turpanensis	+	+	+
Spirochaetes_bacterium_GWB1_27_13	+	+	+
Desulfuromonas_acetoxidans	+	+	+
Rhodanobacteraceae_bacterium_Dysh456	+	+	+
Methanomassiliicoccales_archaeon_PtaB.Bin215	+	+	+
Veillonella_atypica	+	+	+
Hymenobacter_sp._NL	-	+	+
Bacillus_sp._VT_712	+	+	+
Saccharothrix_sp._ST-888	-	+	+
Paenibacillus_ehimensis	+	+	+
Sphingobacterium_lactis	+	+	+
Collinsella_sp._AF04-24	+	+	+
Pseudooceanicola_lipolyticus	+	+	+
Virgibacillus_sp._CM-4	+	+	+
Chryseobacterium_sp._36-9	+	+	+
Fischerella_sp._PCC_9431	-	+	+
Nocardioides_lianchengensis	-	+	+
Staphylococcus_simulans	+	+	+
Blautia_sp._OF01-4LB	+	+	+
Tenericutes_bacterium_4572_104	+	+	+
Pseudomonas_sp._GM50	+	+	+
Methanobrevibacter_curvatus	+	+	+
Absiella_sp._AM54-8XD	+	+	+
Methanomassiliicoccales_archaeon_RumEn_M2	+	+	+
Capnocytophaga_sp._oral_taxon_380	+	+	+
Clostridium_sp._AF15-49	-	+	+
Coprobacillus_sp._AF35-8	+	+	+
Virgibacillus_sp._LJ137	+	+	+
Adlercreutzia_sp._Marseille-P7992	+	+	+
Mycobacterium_intracellulare	+	+	+
Nocardiopsis_potens	-	+	+
Desulfonatronum_thiodismutans	+	+	+

Stigmatella_erecta	+	+	+
Deinococcus_misasensis	+	+	+
Brockia_lithotrophica	+	+	+
[Bacillus]_clarkii	+	+	+
Thioalkalivibrio_sp._AKL11	+	+	+
Acidithiobacillus_albertensis	+	+	+
Collinsella_sp._TF05-9AC	+	+	+
bacterium_CG2_30_54_10	-	+	+
Parcubacteria_group_bacterium_GW2011_GWA1_36_12	-	+	+
Pseudomonas_stutzeri	+	+	+
Candidatus_Gottesmanbacteria_bacterium_RIFCSPHIGHO2_02	+	+	+
Lysinibacillus_boronitolerans	+	+	+
Lysinibacillus_telephonicus	+	+	+
Paenibacillus_sp._AR247	-	+	+
Capnocytophaga_sp._CM59	+	+	+
Candidatus_Peregrinibacteria_bacterium_RIFOXYB2_FULL_3:	+	+	+
Pontibacillus_chungwhensis	+	+	+
Cellulomonas_fimi	+	+	+
Spirosoma_oryzae	+	+	+
Synechococcus_sp._NKBG042902	+	+	+
Xanthomonas_virus_XcP1	+	+	+
Porphyromonadaceae_bacterium_KHP3R9	+	+	+
Candidatus_Gottesmanbacteria_bacterium_GW2011_GWB1_49	+	+	+
Elusimicrobia_bacterium_HGW-Elusimicrobia-2	-	+	+
Archaeoglobales_archaeon	-	+	+
Kocuria_sp._WRN011	+	+	+
Tindallia_magadiensis	+	+	+
Limnohabitans_sp._Rim8	+	+	+
Nitrospira_bacterium_SM23_35	-	+	+
Pleomorphomonas_sp._SM30	+	+	+
Olsenella_sp._Marseille-P2300	+	-	+
Rhizobium_nepotum	+	-	+
Pectobacterium_phage_DU_PP_III	+	-	+
Lactobacillus_sp._17-4	+	-	+
Lactobacillus_sp._UMNPBX18	+	-	+
uncultured_Microbacterium_sp.	+	-	+
methanogenic_archaeon_ISO4-H5	+	-	+
Paraburkholderia_bannensis	-	-	+
Virgibacillus_profundi	-	-	+
Fischerella_thermalis	-	-	+
Candidatus_Moranbacteria_bacterium_RBG_13_45_13	-	-	+
Janthinobacterium_sp._PC23-8	-	-	+
Lacinutrix_venerupis	-	-	+
Helicobacteraceae_bacterium_CG1_02_36_14	-	-	+
Caballeronia_choica	-	-	+
Mesotoga_sp._B105.6.4	-	-	+
alpha_proteobacterium_L41A	-	-	+
Leifsonia_sp._Leaf264	-	-	+
Candidatus_Saccharibacteria_bacterium_RIFCSPHIGHO2_12_I	-	-	+
Candidatus_Woesearchaeota_archaeon_CG1_02_33_12	-	-	+
Methanothermococcus_okinawensis	-	-	+
Paenibacillus_glacialis	+	+	+
Selenomonas_sp._FC4001	+	+	+
Streptococcus_massiliensis	+	+	+
Bacillus_canaveralius	+	+	+

Bacillus_sp._FJAT-27245	+	+	+
Enterococcus_moraviensis	+	+	+
Bacillus_sp._SG-1	+	+	+
Bacillus_oceanisediminis	+	+	+
Exiguobacterium_sp._HVEsp1	+	+	+
Paenibacillus_sp._OV219	+	+	+
Parageobacillus_thermoglucosidasius	+	+	+
Paenibacillus_sp._1-49	+	+	+
Thermovirga_lienii	+	+	+
Chlorobaculum_sp._24CR	+	+	+
Flavobacterium_sp._RB1N8	+	+	+
Bifidobacterium_sp._2036B	+	+	+
Capnocytophaga_canimorsus	+	+	+
cyanobacterium_G8-9	+	+	+
Lactobacillus_gastricus	+	+	+
Desulfobacterales_bacterium_CG23_combo_of_CG06-09_8_20	+	+	+
Novosphingobium_sp._THN1	+	+	+
Desulfovibrionaceae_bacterium_CG1_02_65_16	+	+	+
Veillonellaceae_bacterium_KA00182	+	+	+
Lactobacillus_phage_LfeInf	-	+	+
Dialister_sp._CAG:486	-	+	+
Vibrio_campbellii	+	+	+
Meiothermus_hypogaeus	+	+	+
Geobacillus_genomosp._3	+	+	+
Paenibacillus_thiaminolyticus	+	+	+
Candidatus_Melainabacteria_bacterium_35_41	+	+	+
Flavobacterium_aquatile	+	+	+
bacterium_SM23_57	+	+	+
Metaprevotella_massiliensis	+	+	+
Empedobacter_falsenii	+	+	+
Carnobacterium_mobile	+	+	+
Methylomonas_lenta	-	+	+
Candidatus_Margulisbacteria_bacterium_GWF2_35_9	+	+	+
Rubrivivax_sp._ICH-3	+	+	+
Gordonia_sp._AMA120	+	+	+
Burkholderia_sp._OLGA172	+	+	+
Mesorhizobium_sp._LNHC221B00	+	+	+
Flavobacterium_pectinovorum	+	+	+
Draconibacterium_sediminis	+	+	+
Planctomycetes_bacterium_RBG_16_55_9	+	+	+
Streptomyces_sp._42	+	+	+
Candidatus_Aminicenantes_bacterium_RBG_16_63_16	+	+	+
Tepidibacter_mesophilus	+	+	+
Kosmotoga_pacifica	+	+	+
Flavobacteriia_bacterium_40-80	-	+	+
Bacillus_sp._FJAT-45122	-	+	+
Zetaproteobacteria_bacterium_CG17_big_fil_post_rev_8_21_14	-	+	+
Arthrobacter_sp._Y81	+	+	+
Parcubacteria_group_bacterium_CG_4_9_14_0_2_um_filter_48	+	+	+
Desulfotomaculum_sp._BICA1-6	+	+	+
Clostridium_saudiense	+	+	+
Bacillus_sporothermodurans	+	+	+
Veillonella_denticariosi	+	+	+
Bifidobacterium_hapali	+	+	+
Thioploca_ingrica	-	+	+

Planctomycetes_bacterium_DG_23	-	+	+
Candidatus_Saccharibacteria_bacterium_49-20	-	+	+
Levilinea_saccharolytica	+	+	+
Bacteroidetes_bacterium_43-93	+	+	+
Klebsiella_variicola	+	+	+
Candidatus_Thorarchaeota_archaeon	+	+	+
Ruegeria_sp._NKC1-1	+	+	+
Halomonas_sp._KCTC_52281	-	+	+
Burkholderia_thailandensis	-	+	+
Mesorhizobium_plurifarium	-	+	+
Paenibacillus_panacisoli	-	+	+
Streptococcus_hongkongensis	-	+	+
Caloranaerobacter_ferrireducens	+	+	+
Haliscomenobacter_hydrossis	+	+	+
Paenibacillus_xylaniclasticus	+	+	+
Candidatus_Gottesmanbacteria_bacterium_RIFCSPHIGHO2_01	+	+	+
Serratia_marcescens	+	+	+
Guyparkeria_sp._SCN-R1	+	+	+
Pedobacter_caeni	+	+	+
Edwardsiella_ictaluri	+	+	+
Photobacterium_gaetbulicola	+	+	+
Enzootic_nasal_tumour_virus_of_goats	+	+	+
Lactobacillus_shenzhenensis	-	+	+
Azoarcus_olearius	-	+	+
Bacillus_coahuilensis	+	+	+
Wolbachia_pipientis	+	+	+
Sporosarcina_psychrophila	-	+	+
Bacteroidetes_bacterium_RBG_13_43_22	-	+	+
Candidatus_Liberibacter_solanacearum	-	+	+
Salipaludibacillus_neizhouensis	+	+	+
Bacteroidetes_bacterium_HGW-Bacteroidetes-15	+	+	+
Enterococcus_plantarum	+	+	+
Paenibacillus_sp._FSL_R5-808	-	+	+
Aequorivita_sp._H23M31	-	+	+
Gramella_flava	-	+	+
Erysipelothrix_phage_phi1605	-	+	+
Leptotrichia_massiliensis	-	+	+
Candidatus_Wildermuthbacteria_bacterium_RIFCSPHIGHO2_0	-	+	+
Dyadobacter_psychrophilus	+	-	+
Rhodococcus_ruber	+	-	+
Lachnospiraceae_bacterium_VE202-23	+	-	+
Kingella_kingae	+	-	+
Bacteroidetes_bacterium_GWA2_31_9	+	-	+
Trueperella_pyogenes	+	-	+
Tenericutes_bacterium_GWC2_34_14	+	-	+
Jeotgalibacillus_soli	+	-	+
Flavobacterium_sp._140616W15	+	-	+
Micromonospora_chaiyaphumensis	+	-	+
Arthrobacter_sp._cf158	+	-	+
Vibrio_nigripulchritudo	+	-	+
Enterococcus_sp._10A9_DIV0425	+	-	+
Sutterella_sp._63_29	+	-	+
Chitinophaga_sp._K20C18050901	-	-	+
Candidatus_Roizmanbacteria_bacterium_RIFCSPHIGHO2_02_	-	-	+
Francisella_hispaniensis	-	-	+

Dehalococcoidia_bacterium	-	-	+
Candidatus_Sungbacteria_bacterium_RIFCSPLOWO2_01_FUL	-	-	+
Bacillus_virus_Bc431	-	-	+
Bacillus_sp._NFR08	-	-	+
Candidatus_Roizmanbacteria_bacterium_CG_4_10_14_0_8_um	-	-	+
Yersinia_kristensenii	-	-	+
Parcubacteria_group_bacterium_CG2_30_36_18	-	-	+
Koala_retrovirus	+	+	+
Bacillus_rubiinfantis	+	+	+
Shuttleworthia_sp._MSX8B	+	+	+
Gigaspora_rosea	+	+	+
Lactobacillus_sp._ASF360	+	+	+
Clostridium_putrefaciens	+	+	+
Streptococcus_pseudopneumoniae	+	+	+
Dehalobacter_restrictus	+	+	+
Lactobacillus_mudanjiangensis	+	+	+
Risungbinella_massiliensis	+	+	+
Bacteroidetes_bacterium_GWF2_33_38	+	+	+
Atopococcus_tabaci	+	+	+
Bacteroidetes_bacterium_HGW-Bacteroidetes-5	+	+	+
Murdochiella_massiliensis	+	+	+
Curvibacter_lanceolatus	+	+	+
Anaerococcus_prevotii	+	+	+
Sinomicrobium_sp._N-1-3-6	+	+	+
Bacillus_campisalis	+	+	+
Bacteroides_sp._AF35-22	+	+	+
Paenibacillus_sp._FJAT-26967	+	+	+
Flagellimonas_pacifica	-	+	+
Flexithrix_dorotheae	+	+	+
Selenomonas_sp._oral_taxon_136	+	+	+
Aquimarina_sp._Aq107	+	+	+
Leuconostoc_mesenteroides	+	+	+
Candidatus_Arthromitus_sp._SFB-rat-Yit	+	+	+
Ancylomarina_sp._A4	+	+	+
Ammonifex_sp.	+	+	+
Collinsella_sp._An268	+	+	+
Desulfotomaculum_profundi	+	+	+
Spirochaetes_bacterium_GWB1_36_13	+	+	+
Synergistetes_bacterium_HGW-Synergistetes-2	+	+	+
Bacillus_sp._PK3_68	+	+	+
Candidatus_Microgenomates_bacterium	+	+	+
Mesorhizobium_sp._M4A.F.Ca.ET.090.04.2.1	+	+	+
Candidatus_Amoebophilus_asiaticus	-	+	+
Thaumarchaeota_archaeon	+	+	+
Lactococcus_reticulitermitis	+	+	+
Sporocytophaga_myxococcoides	+	+	+
Paenibacillus_darwinianus	+	+	+
Marispirochaeta_aestuarii	+	+	+
Bradyrhizobium_sp._Rc3b	+	+	+
Methanosarcinales_archaeon	+	+	+
Bacillus_kwashiorkori	+	+	+
Sphingobacteriaceae_bacterium_DW12	+	+	+
Salinimicrobium_terrae	+	+	+
Marinobacterium_stanieri	+	+	+
Bacteroidetes_bacterium_4484_276	+	+	+

Burkholderia_cepacia	-	+	+
Sphingobacteriales_bacterium_41-5	-	+	+
Paenibacillus_humicus	+	+	+
uncultured_bacterium_pG7	+	+	+
Halobacillus_dabanensis	+	+	+
Salimicrobium_salexigens	-	+	+
Aminobacter_sp._J41	+	+	+
Yokenella_regensburgei	+	+	+
Sparassis_crispa	+	+	+
Rubrivivax_sp.	+	+	+
Candidatus_Aminicenantes_bacterium_RBG_13_59_9	+	+	+
Tamlana_sedimentorum	+	+	+
Chryseolinea_sp._KIS68-18	+	+	+
Propionivibrio_dicarboxylicus	-	+	+
Albidiferax_sp._OV413	+	+	+
Sphingobacterium_gobiense	+	+	+
Myroides_injenensis	+	+	+
Joostella_marina	+	+	+
Spirosoma_fluviale	+	+	+
Marinoscillum_furvescens	+	+	+
Lihuaxuella_thermophila	+	+	+
Roseivivax_roseus	+	+	+
Lysinibacillus_sinduriensis	-	+	+
Bacillus_sp._YSP-3	-	+	+
Selenomonas_sp._AE3005	+	+	+
Flavobacterium_filum	+	+	+
Flaviramulus_basaltis	+	+	+
Paenibacillus_campinasensis	+	+	+
Oceanobacillus_picturae	+	+	+
Listeria_booriae	+	+	+
Pseudogracilibacillus_auburnensis	+	+	+
Veillonella_tobetsuensis	-	+	+
Niabella_drilacis	+	+	+
Anaerococcus_senegalensis	+	+	+
Trichodesmium_erythraeum	+	+	+
Gracilibacillus_phocaeensis	+	+	+
Slackia_sp._CM382	+	+	+
Chlorobaculum_limnaeum	+	+	+
Pseudomonas_oleovorans	+	+	+
Marinobacter_sp._DSM_26671	+	+	+
Salinicola_socius	-	+	+
Alkaliflexus_imshenetskii	+	+	+
Arenibacter_sp._ARW7G5Y1	+	+	+
bacterium_BM706	+	+	+
Paenibacillus_sp._NAIST15-1	+	+	+
Candidatus_Saccharibacteria_bacterium_RIFCSPHIGHO2_01_I	+	+	+
Cupriavidus_taiwanensis	+	+	+
Pedobacter_yulinensis	-	+	+
Clostridiaceae_bacterium_OM02-2AC	+	-	+
Chloroflexi_bacterium_HGW-Chloroflexi-4	+	-	+
Thermoanaerobacter_ethanolicus	+	-	+
Bacillus_horneckiae	+	-	+
Streptomyces_odonnellii	+	-	+
Lactococcus_chungangensis	+	-	+
Alkalibacterium_putridalgicola	+	-	+

Brevundimonas_sp._SH203	+	-	+
Candidatus_Roizmanbacteria_bacterium_RIFCSPHIGHO2_01_	+	-	+
Acidovorax_wautersii	+	-	+
uncultured_archaeon	+	-	+
uncultured_archaeon_GZfos26D8	+	-	+
Coprobacillus_sp._AM23-9LB	+	-	+
Bacillus_firmus	+	-	+
Lactobacillus_jensenii	+	-	+
Dethiobacter_alkaliphilus	+	-	+
Methanocorpusculum_labreanum	+	-	+
Collinsella_sp._OM07-12	+	-	+
Thermogemmatispora_tikiterensis	+	-	+
Nocardia_cyriacigeorgica	+	-	+
Rhodohalobacter_barkolensis	-	-	+
Candidatus_Zambryskibacteria_bacterium_RIFOXYC1_FULL_	-	-	+
Flavobacterium_sp._1	-	-	+
Neisseria_cinerea	-	-	+
Actinomyces_sp._ICM47	-	-	+
Candidatus_Saccharibacteria_bacterium_32-49-10	-	-	+
Tenericutes_bacterium_HGW-Tenericutes-7	-	-	+
Lentisphaerae_bacterium_GWF2_57_35	-	-	+
Xenorhabdus_hominickii	-	-	+
Methanomethylovorans_hollandica	-	-	+
Chryseobacterium_gregarium	-	-	+
Endozoicomonas_ascidiicola	+	+	+
Lactobacillus_agilis	+	+	+
Glomus_cerebriforme	+	+	+
Ruminococcus_sp._CAG:108	+	+	+
Bhargavaea_cecembensis	+	+	+
Firmicutes_bacterium_AM29-6AC	+	+	+
Ignavibacteria_bacterium_CG1_02_37_35	+	+	+
Brevibacillus_parabrevis	+	+	+
Arthrobacter_sp._D2	+	+	+
Actinobacteria_bacterium	+	+	+
Desulfovibrio_alkalitolerans	-	+	+
Lactobacillus_sp._159-4	+	+	+
Clostridium_sp._CAG:1000	+	+	+
Vibrio_mangrovi	+	+	+
Micavibrio_aeruginosavorus	+	+	+
Parabacteroides_sp._D26	+	+	+
Megasphaera_sp._MJR8396C	+	+	+
Vagococcus_elongatus	+	+	+
Pseudomonas_luteola	+	+	+
bacterium_DOLZORAL124_64_63	+	+	+
Pseudoalteromonas_luteoviolacea	+	+	+
Leptospira_vanthielii	-	+	+
Streptococcus_plurextorum	+	+	+
Persicobacter_sp._CCB-QB2	+	+	+
Rhodobacteraceae_bacterium_Water-Bin34	+	+	+
Enterobacteriaceae_bacterium_B14	+	+	+
Planomicrobium_glaciei	+	+	+
Verrucomicrobia_bacterium_CAG:312_58_20	+	+	+
Thermoanaerobacterium_sp._PSU-2	+	+	+
Flaviflexus_massiliensis	-	+	+
Lachnospiraceae_bacterium_AM10-38	+	+	+

Paenibacillus_tyrfis	+	+	+
Chryseobacterium_sp._YR459	+	+	+
Bacteroidetes_bacterium_RBG_19FT_COMBO_42_7	+	+	+
Desulfobacca_sp._RBG_16_58_9	+	+	+
Desulfatitalea_tepidiphila	+	+	+
Paenibacillus_sp._NFR01	+	+	+
Chloroflexi_bacterium_RBG_16_51_9	-	+	+
Candidatus_Magnetobacterium_casensis	+	+	+
Muricauda_lutaonensis	+	+	+
Lysinibacillus_sp._FJAT-14745	+	+	+
Thioalbus_denitrificans	+	+	+
Thioalkalivibrio_thiocyanodenitrificans	+	+	+
Tetzosporium_hominis	+	+	+
Ancylobacter_rudongensis	+	+	+
Enhygromyxa_salina	+	+	+
Candidatus_Gottesmanbacteria_bacterium_GW2011_GWC2_39	+	+	+
Treponema_sp.	+	+	+
Waddlia_chondrophila	+	+	+
Sphingobium_sp._AP50	-	+	+
Candidatus_Kapabacteria_bacterium	-	+	+
Candidatus_Woesearchaeota_archaeon_CG11_big_fil_rev_8_21	-	+	+
Thiotrichales_bacterium_HS_08	-	+	+
Sedimentibacter_sp._SX930	+	+	+
Magnetofaba_australis	+	+	+
Riemerella_columbipharyngis	+	+	+
Thalassobacillus_cyri	+	+	+
Chryseobacterium_sp._KLBC_52	+	+	+
Olleya_sp._VCSM12	-	+	+
Persephonella_hydrogeniphila	-	+	+
Polaribacter_sp._WD7	+	+	+
Formosa_algae	+	+	+
Aeromonas_jandaei	+	+	+
Bacillus_selenatarsenatis	+	+	+
Gracilibacillus_lacisalsi	+	+	+
Bergeriella_denitrificans	-	+	+
Sporosarcina_sp._HYO08	-	+	+
bacterium_TMED80	-	+	+
uncultured_bacterium_Contig19	-	+	+
Bacteroidetes_bacterium_HGW-Bacteroidetes-9	+	+	+
Bacteroidetes_bacterium_HGW-Bacteroidetes-11	+	+	+
Sanguibacter_suarezii	+	+	+
Dialister_sp._CAG:588	+	+	+
Psychrobacter_lutiphocae	+	+	+
Lactobacillus_apis	-	+	+
Bdellovibrio_sp.	-	+	+
Atopobium_fossor	+	+	+
Fibrobacter_sp._UWH5	+	+	+
Oceanobacillus_massiliensis	+	+	+
Amycolatopsis_regifaucium	+	+	+
Methanosphaera_sp._rholeuAM130	+	+	+
Alicyclobacillus_pomorum	+	+	+
Flavobacterium_sp._1E403	+	+	+
Methyloferula_stellata	+	+	+
Bacteroidetes_bacterium_GWA2_32_17	+	+	+
Sporosarcina_koreensis	+	+	+

Nonlabens_sp._Hel1_33_55	+	+	+
Rhizomicrobium_sp._SCGC_AG-212-E05	+	+	+
Lysinibacillus_varians	+	+	+
Aerococcus_urinae	+	+	+
Peptoniphilus_sp._HMSC062D09	+	+	+
Blautia_sp._AM46-5	-	+	+
Chitinophagaceae_bacterium_K23C18032701	-	+	+
Gemmatimonadetes_bacterium_GWC2_71_10	-	+	+
Oceanimonas_sp._GK1	+	+	+
Geobacteraceae_bacterium_GWC2_55_20	+	+	+
Psychromonas_ossibalaenae	+	+	+
Paenibacillus_sabinae	+	+	+
Clostridiales_bacterium_VE202-18	+	+	+
Virgibacillus_ndiopensis	+	+	+
Bacillus_cecembensis	+	+	+
Chelatococcus_asaccharovorans	+	+	+
Bacteroides_sp._AM51-7	+	+	+
Tessaracoccus_aquimaris	+	+	+
Thermaerobacter_sp._PB12/4term	-	+	+
Candidatus_Saccharibacteria_bacterium_CG_4_10_14_0_2_um_	-	+	+
Calothrix_parasitica	+	+	+
Nitrospirae_bacterium_RBG_13_41_22	+	+	+
Candidatus_Peribacter_riflensis	+	+	+
Runella_zeae	+	+	+
Flectobacillus_sp._BAB-3569	+	+	+
Planctomycetaceae_bacterium_TMED10	-	+	+
Sporosarcina_sp._P3	-	+	+
Bacillus_phage_MG-B1	+	+	+
Acetobacter_pasteurianus	+	+	+
Bacillus_sp._J33	+	+	+
Winogradskyella_arenosi	+	+	+
Bacillus_sp._FJAT-26390	+	+	+
Gluconobacter_frateurii	-	+	+
Rhodohalobacter_halophilus	-	+	+
Ruminococcus_sp._CAG:9	+	-	+
Rhodobiaceae_bacterium	+	-	+
Bacillus_sp._MRMR6	+	-	+
Oleiphilus_messinensis	+	-	+
Candidatus_Accumulibacter_sp._66-26	+	-	+
Dyadobacter_alkalitolerans	+	-	+
Virgibacillus_sp._7505	+	-	+
Mesorhizobium_sp._M6A.T.Cr.TU.017.01.1.1	+	-	+
Aneurinibacillus_tyrosinisolvens	+	-	+
Glycomyces_tenuis	+	-	+
Paenibacillus_chitinolyticus	+	-	+
Burkholderiales_bacterium_70-64	+	-	+
Anaerolineaceae_bacterium_4572_5.1	+	-	+
Fictibacillus_solisalsi	+	-	+
Hydrogenimonas_thermophila	+	-	+
Lactobacillus_parafarraginis	+	-	+
Cytophagales_bacterium_CG17_big_fil_post_rev_8_21_14_2_5	-	-	+
Polaromonas_sp._YR568	-	-	+
Jeotgalibacillus_campisalis	-	-	+
Methanobrevibacter_olleyae	-	-	+
Candidatus_Saccharibacteria_bacterium_YM_S32_TM7_50_20	-	-	+

Wohlfahrtiimonas_chitiniclastica	-	-	+
Mameliella_alba	-	-	+
Sporosarcina_sp._BI001-red	-	-	+
Aggregatibacter_aphrophilus	-	-	+
Thermotoga_profunda	-	-	+
Anaeromyxobacter_sp._K	-	-	+
Paraburkholderia_sp._C35	-	-	+
Candidatus_Saccharibacteria_bacterium_CG10_big_fil_rev_8_2	-	-	+
Python_molurus_endogenous_retrovirus	+	+	+
Collinsella_sp._An307	+	+	+
Streptomyces_sparsogenes	+	+	+
Human_endogenous_retrovirus_K	+	+	+
Bacillus_indicus	+	+	+
Paenibacillus_sp._FSL_R7-0331	+	+	+
Candidatus_Arthromitus_sp._SFB-turkey	+	+	+
Nostocales_cyanobacterium_HT-58-2	+	+	+
Bacillus_sp._SA1-12	+	+	+
Halanaerobium_hydrogeniformans	+	+	+
Desulfofundulus_salinum	+	+	+
Coprobacillus_sp._AF37-2	-	+	+
Lactobacillus_amylovorus	+	+	+
Methanoculleus_bourgensis	+	+	+
Treponema_berlinense	+	+	+
Arcobacter_butzleri	+	+	+
Bacillus_oryziterrae	+	+	+
Tardiphaga_sp._YR296	+	+	+
Desulfacinum_infernum	+	+	+
Microcystis_phage_MaMV-DC	+	+	+
Chitinophaga_sp._GDMCC_1.1325	+	+	+
Neisseria_elongata	+	+	+
Bacillus_shacheensis	+	+	+
Enterovibrio_norvegicus	+	+	+
Clostridium_sp._AM46-21	+	+	+
uncultured_bacterium_Contig1762	+	+	+
Pedobacter_heparinus	+	+	+
Bacillus_halodurans	+	+	+
Enterococcus_sulfureus	+	+	+
Azohydromonas_lata	+	+	+
Acidovorax_sp._Leaf78	+	+	+
Janthinobacterium_sp._LM6	-	+	+
Fervidobacterium_pennivorans	-	+	+
Microvirga_vignae	-	+	+
Peptoniphilus_sp._oral_taxon_386	+	+	+
Sphingobacterium_sp._1.A.4	+	+	+
Desulfonatronum_lacustre	+	+	+
Pedobacter_sp._BAL39	+	+	+
Nitrospirae_bacterium_GWF2_44_13	+	+	+
Fusobacterium_sp.	+	+	+
Marinobacterium_rhizophilum	+	+	+
Grimontella_sp._AG753	+	+	+
Fibrobacter_sp._UWH9	-	+	+
Methylomonas_methanica	-	+	+
Paenibacillus_sp._FSL_A5-0031	+	+	+
Pseudomonas_sp._286	+	+	+
Flavobacterium_sp._MedPE-SWcel	+	+	+

Omnitrophica_bacterium_RIFCSPHIGHO2_02_FULL_45_28	+	+	+
Bacillus_filamentosus	+	+	+
Candidatus_Margulisbacteria_bacterium	-	+	+
SAR202_cluster_bacterium_MP-SInd-SRR3963457-G2	-	+	+
Bacteroides_sp._KFT8	+	+	+
Marinimicrobium_koreense	+	+	+
Caldicellulosiruptor_naganoensis	+	+	+
Lascolabacillus_massiliensis	+	+	+
Actinobacteria_bacterium_HGW-Actinobacteria-7	+	+	+
Bdellovibrionales_bacterium_RIFOXYC1_FULL_37_79	+	+	+
Listeria_aquatica	+	+	+
Chitinophaga_niabensis	+	+	+
Chromobacterium_sphagni	+	+	+
Fictibacillus_arsenicus	+	+	+
Bacillus_sp._MKU004	+	+	+
Larkinella_soli	+	+	+
Glaciecola_pallidula	+	+	+
Bacteroidetes_bacterium_GWE2_40_63	+	+	+
Parapedobacter_composti	+	+	+
Ignavibacteriae_bacterium_HGW-Ignavibacteriae-1	+	+	+
Candidatus_Magasanikbacteria_bacterium_GW2011_GWE2_42	+	+	+
Desulfuromusa_kysingii	-	+	+
Domibacillus_antri	+	+	+
Lelliottia_jeotgali	+	+	+
Pantoea_ananatis	+	+	+
Desulfotalea_psychrophila	+	+	+
Virgibacillus_sp._SK37	-	+	+
Actinomyces_sp.	-	+	+
Cecembia_rubra	+	+	+
Bifidobacterium_xylocopae	+	+	+
Chryseobacterium_sp._JAH	+	+	+
Aminomonas_paucivorans	+	+	+
Psychrobacillus_insolitus	+	+	+
Candidatus_Peregrinibacteria_bacterium_GW2011_GWF2_33_	+	+	+
Acinetobacter_sp._WCHAc060115	-	+	+
Streptomyces_fradiae	-	+	+
Lactobacillus_saerimneri	+	+	+
Bacteroides_sp._SM23_62_1	+	+	+
Fibrobacter_sp._UWH6	+	+	+
Polaribacter_sp._Hel_I_88	+	+	+
Arthrobacter_alpinus	+	+	+
Cohnella_laeviribosi	+	+	+
endosymbiont_'TC1'_of_Trimyema_compressum	+	+	+
Leptotrichia_sp._oral_taxon_212	+	+	+
Candidatus_Thiodiazotropha_endolucinida	+	+	+
Mucilaginibacter_kameinonensis	+	+	+
Paenibacillus_sp._FSL_R5-0490	+	+	+
Dyadobacter_sp._50-39	+	+	+
Actinobacteria_bacterium_CG2_30_50_142	+	+	+
Rufibacter_ruber	+	+	+
Candidatus_Paracaedibacter_symbiosus	+	+	+
Flavobacterium_subsaxonicum	+	+	+
Halobacillus_halophilus	+	+	+
Pseudovibrio_sp._Tun.PSC04-5.I4	-	+	+
Numidum_massiliense	-	+	+

Enterococcus_saccharolyticus	-	+	+
Pseudoruegeria_sp._SK021	-	+	+
Aeromicrobium_sp._PE09-221	-	+	+
Methanolobus_psychrophilus	+	+	+
Thermosulfurimonas_dismutans	+	+	+
Chryseobacterium_sp._H6466	+	+	+
Aquiflexum_sp._Z0201	+	+	+
Desulforhopalus_singaporensis	+	+	+
uncultured_bacterium_Contig1491	+	+	+
uncultured_haloarchaeon	+	+	+
Planctomycetes_bacterium_RIFCSPLOWO2_12_FULL_39_13	+	+	+
Streptococcus_sp._I-P16	-	+	+
Candidatus_Levybacteria_bacterium_RBG_16_35_6	-	+	+
Collinsella_sp._AM28-11LB	-	+	+
Gammaproteobacteria_bacterium_CG22_combo_CG10-13_8_2	-	+	+
Libanicoccus_massiliensis	+	+	+
Capnocytophaga_sp._oral_taxon_323	+	+	+
Emticicia_sp._17J42-9	+	+	+
Hymenobacter_gummosus	+	+	+
Moellerella_wisconsensis	+	+	+
Helicobacter_bizzozeronii	+	+	+
Rudanella_lutea	+	+	+
Candidatus_Saccharibacteria_bacterium_RIFCSPHIGHO2_02_I	+	+	+
Chania_multitudinisentens	-	+	+
Kazachstania_africana	-	+	+
Naumovozyma_dairenensis	-	+	+
Bacillus_hemicellulosilyticus	-	+	+
Brevibacillus_sp._BC25	-	+	+
Coprobacillus_sp._AM17-34	+	-	+
Cryomorphaceae_bacterium_BACL21_MAG-121220-bin10	+	-	+
Clostridium_sp._AM43-3BH	+	-	+
Methylobacterium_sp._ARG-1	+	-	+
Acinetobacter_sp._neg1	+	-	+
Flavobacterium_ummariense	+	-	+
Bacillus_sp._OxB-1	+	-	+
Candidatus_Methanomethylophilus_sp._1R26	+	-	+
Candidatus_Uhrbacteria_bacterium_CG22_combo_CG10-13_8_	+	-	+
Bacillus_sp._AFS029533	+	-	+
Apibacter_sp._HY039	+	-	+
Jeotgalibacillus_sp._22-7	+	-	+
Peptoniphilus_indolicus	+	-	+
Clostridium_sp._OM05-6BH	+	-	+
Thalassotalea_agarivorans	-	-	+
Desulfobulbus_mediterraneus	-	-	+
Brevibacillus_sp._OK042	-	-	+
Brevibacillus_sp._NRRL_B-41110	-	-	+
Candidatus_Electrothrix_aarhusiensis	-	-	+
Propionicimonas_paludicola	-	-	+
Parabacteroides_sp._AM25-14	-	-	+
Apibacter_sp._HY041	-	-	+
Marinitoga_sp._1137	-	-	+
Demequina_sp._NBRC_110055	-	-	+
Arthrobacter_phage_Mendel	-	-	+
Arthrobacter_sp._S39	-	-	+
Veillonella_caviae	-	-	+

Lactobacillus_sp._HMSC073D04	-	-	+
Prosthecochloris_sp._ZM_2	-	-	+
Parcubacteria_group_bacterium_GW2011_GWC2_32_10	-	-	+
Paraburkholderia_monticola	-	-	+
Candidatus_Uhrbacteria_bacterium_CG_4_10_14_0_8_um_filte	-	-	+
Aciduliprofundum_sp._MAR08-339	-	-	+
Halobacillus_sp._SKP4-6	-	-	+
Algoriphagus_marincola	-	-	+
Chitinophaga_terrae_Kim_and_Jung_2007	-	-	+
Alcanivorax_sp._DSM_26293	-	-	+
Carnobacterium_viridans	+	+	+
Gibbon_ape_leukemia_virus	+	+	+
Ruminobacter_amylophilus	-	+	+
Bacteroides_sp._NMBE5	+	+	+
Rozella_allomycis	+	+	+
Methanobrevibacter_ruminantium	+	+	+
Paenibacillus_alginolyticus	+	+	+
Staphylococcus_phage_vB_SscM-1	+	+	+
uncultured_bacterium_Ad_113_I18_contig2	+	+	+
Desulfovibrio_sp._K3S	-	+	+
Tenericutes_bacterium_HGW-Tenericutes-6	+	+	+
Chryseobacterium_haifense	+	+	+
Candidatus_Accumulibacter_sp._SK-11	+	+	+
Salinivirgaceae_bacterium	+	+	+
Chitinophaga_sp._CF118	+	+	+
Bacillus_sp._FJAT-29937	-	+	+
Candidatus_Atribacteria_bacterium_1244-E10-H5-B2	+	+	+
Bergeyella_cardium	+	+	+
Peptoniphilus_sp._BV3C26	+	+	+
Peptoniphilus_obesi	+	+	+
Planctomycetes_bacterium_GWC2_45_44	+	+	+
Bacillus_amyloliquefaciens	+	+	+
Dickeya_zeae	+	+	+
Candidatus_Magasanikbacteria_bacterium_CG10_big_fil_rev_8	+	+	+
Halolactibacillus_miurensis	+	+	+
Pseudomonas_aestusnigri	+	+	+
Paenibacillus_sp._1ZS3-15	+	+	+
Algoriphagus_chordae	-	+	+
Providencia_stuartii	+	+	+
Zobellella_denitrificans	+	+	+
Chamaesiphon_minutus	+	+	+
Gillisia_mitskevichiae	+	+	+
Alkalilimnicola_ehrlichii	+	+	+
Tenacibaculum_gallaicum	+	+	+
Bacteroidetes_bacterium_RIFCSPHIGHO2_02_FULL_44_7	+	+	+
Corynebacterium_sp._HMSC076G08	+	+	+
Mesonia_sp._K7	+	+	+
Treponema_sp._OMZ_838	+	+	+
Acinetobacter_sp._NIPH_809	+	+	+
Bacillus_sp._FJAT-44876	+	+	+
Parapedobacter_koreensis	+	+	+
Lactobacillus_helveticus	+	+	+
Bacteroides_sp._HMSC068A09	+	+	+
Amphibacillus_xylanus	+	+	+
Lactobacillus_virus_phiLdb	+	+	+

Bacillus_alkalinitrilicus	+	+	+
Bdellovibrionales_bacterium_CG10_big_fil_rev_8_21_14_0_10	+	+	+
Winogradskyella_sp._PG-2	+	+	+
Bacillus_wudalianchiensis	+	+	+
Syntrophobacterales_bacterium_CG_4_8_14_3_um_filter_49_1	+	+	+
Quasibacillus_thermotolerans	+	+	+
Erysipelatoclostridium_sp._AM42-17	+	+	+
Mesotoga_infera	+	+	+
Eremococcus_coleocola	+	+	+
Bacillus_vietnamensis	-	+	+
Streptomyces_sp._M41(2017)	+	+	+
Gallibacterium_genomosp._3	+	+	+
Planomicrobium_soli	+	+	+
Thermoactinomyces_daqus	+	+	+
Streptococcus_australis	-	+	+
Kurthia_senegalensis	-	+	+
Brevibacterium_epidermidis	-	+	+
Labilibaculum_filiforme	+	+	+
uncultured_bacterium_contig00010(2014)	+	+	+
Tepidibacillus_decaturensis	+	+	+
Rhodobacter_sp._CCB-MM2	+	+	+
Atopobium_parvulum	+	+	+
Emticicia_sp._TH156	+	+	+
Paenibacillus_sp._VT-16-81	+	+	+
Bacillus_okuhidensis	+	+	+
Flavobacteriales_bacterium_CG18_big_fil_WC_8_21_14_2_50_	+	+	+
Pseudomonas_sp._EGD-AKN5	-	+	+
Vagococcus_martis	-	+	+
Candidatus_Entotheonella_palauensis	-	+	+
Romboutsia_maritimum	+	+	+
Nocardiopsis_gilva	+	+	+
Trichococcus_pasteurii	+	+	+
Thermoflexus_hugenholtzii	+	+	+
Arcobacter_canalis	+	+	+
Syntrophorhabdus_sp._PtaU1.Bin153	+	+	+
Bacillus_virus_G	+	+	+
Collinsella_sp._AF08-23	+	+	+
Halorhodospira_halochloris	+	+	+
Granulicatella_balaenopterae	+	+	+
Succinispira_mobilis	+	+	+
Deltaproteobacteria_bacterium_CG17_big_fil_post_rev_8_21_1	+	+	+
Draconibacterium_sp.	+	+	+
Aeromonas_molluscorum	+	+	+
Fluviicola_taffensis	+	+	+
Carboxydothermus_ferrireducens	-	+	+
Variovorax_sp._CF079	-	+	+
Parapedobacter_indicus	-	+	+
Flammeovirga_pacifica	-	+	+
bacterium_AMD01	-	+	+
Parabacteroides_sp._AM44-16	+	+	+
Chitinophagaceae_bacterium_IBVUCB1	+	+	+
Desulfonema_ishimotonii	+	+	+
Fusobacterium_sp._HMSC064B12	+	+	+
Pelobium_manganitolerans	+	+	+
Mangrovimonas_sp._HN-E26	+	+	+

Cryomorphaceae_bacterium_BACL23_MAG-120924-bin60	+	+	+
Flavobacteriaceae_bacterium_14752	+	+	+
Megasphaera_sp._BV3C16-1	+	+	+
Chitinophaga_sp._MD30	+	+	+
Lactobacillus_pentosus	+	+	+
Criblamydia_sequanensis	+	+	+
Pontibacter_sp._2b14	+	+	+
Staphylococcus_auricularis	-	+	+
Clostridiales_bacterium_SIT11	-	+	+
Candidatus_Bathyarchaeota_archaeon_RBG_13_60_20	+	+	+
Oceanobacillus_bengalensis	+	+	+
Ignatzschineria_ureiclastica	+	+	+
Thermoanaerobacterium_xylanolyticum	+	+	+
Pedobacter_antarcticus	-	+	+
Anaerolinea_thermophila	-	+	+
Corynebacterium_urealyticum	-	+	+
Leucobacter_sp._DSM_101948	-	+	+
Oerskovia_sp._Root22	-	+	+
Cetobacterium_somerae	+	+	+
Lewinella_sp._IMCC34191	+	-	+
Nonlabens_sp._YIK11	+	-	+
Thermoflavifilum_aggregans	+	-	+
Actinomyces_bovis	+	-	+
Salmonella_phage_S100	+	-	+
Anaeroplasma_abactoclasticum	+	-	+
Bacillus_sp._FJAT-21945	+	-	+
Nitrospirae_bacterium_GWA2_46_11	+	-	+
Sinomicrobium_pectinilyticum	+	-	+
Lactobacillus_porcinae	+	-	+
Acetomicrobium_hydrogeniformans	+	-	+
Massilia_alkalitolerans	+	-	+
Planctomycetes_bacterium_SM23_25	+	-	+
Planctomycetes_bacterium_SM23_32	+	-	+
Peptoniphilus_sp._ChDC_B134	+	-	+
Chryseobacterium_elymi	+	-	+
Candidatus_Kentron_sp._FM	+	-	+
Candidatus_Desantisbacteria_bacterium_CG1_02_38_46	+	-	+
Cytophagaceae_bacterium	+	-	+
Chitinophaga_sp._YR573	+	-	+
Candidatus_Arthromitus_sp._SFB-mouse	+	-	+
Candidatus_Cryosericum_terrychapinii	+	-	+
Maribacter_arcticus	+	-	+
Desulfobacterium_vacuolatum	+	-	+
Glycomyces_arizonensis	+	-	+
Halobacteriovorax_sp._HLS	+	-	+
Actinomyces_virus_Av1	+	-	+
Clostridiales_bacterium_KA00134	-	-	+
Halobacillus_trueperi	-	-	+
Schaalia_turicensis	-	-	+
Gemella_cuniculi	-	-	+
Candidatus_Uhrbacteria_bacterium_CG_4_9_14_3_um_filter_5	-	-	+
Rhizobiaceae_bacterium	-	-	+
Paenibacillus_sp._ATY16	-	-	+
Nostoc_sp._NIES-2111	-	-	+
Pseudoalteromonas_sp._P1-13-1a	-	-	+

Lactobacillus_sp._M1530-1	-	-	+
Pantoea_anthophila	-	-	+
Paenisporosarcina_sp._TG-14	-	-	+
Pseudomonas_sp._SWI44	-	-	+
Lactobacillus_coleohominis	-	-	+
Bacillus_phage_BSP38	-	-	+
Bacillus_virus_BCP82	-	-	+
Oidiodendron_maius	-	-	+
Oleiphilus_sp._HI0125	-	-	+
Lysinibacillus_sp._ZYM-1	-	-	+
Candidatus_Woesebacteria_bacterium_RIFOXYA1_FULL_38_!	-	-	+
Achromobacter_sp._2789STDY5608615	-	-	+
Helicobacter_rodentium	-	-	+
Enterococcus_dispar	-	-	+
Virgibacillus_chiguensis	-	-	+
Saliterribacillus_persicus	+	+	+
Candidatus_Syntrophonatronum_acetioxidans	+	+	+
Lichtheimia_corymbifera	+	+	+
Lichtheimia_ramosa	+	+	+
Azospirillum_sp._TSO5	+	+	+
Lachnospiraceae_bacterium_TF10-8AT	+	+	+
Phascolarctobacterium_sp._CAG:207	+	+	+
Cohnella_sp._OV330	+	+	+
Stigmatella_aurantiaca	+	+	+
Bacillus_sonorensis	+	+	+
Microscilla_marina	+	+	+
Methanosarcina_sp._2.H.A.1B.4	+	+	+
Salegentibacter_salegens	+	+	+
Proteobacteria_bacterium	+	+	+
Ktedonobacterales_bacterium_SCAWS-G2	-	+	+
Trichococcus_alkaliphilus	+	+	+
Paucisalibacillus_globulus	+	+	+
Dictyoglomus_turgidum	-	+	+
Capnocytophaga_leadbetteri	+	+	+
Chryseobacterium_hominis	+	+	+
Lactobacillus_sakei	+	+	+
Burkholderiales_bacterium_21-58-4	-	+	+
Bacillus_solisilvae	+	+	+
Bacillus_okhensis	+	+	+
Dyadobacter_koreensis	+	+	+
Candidatus_Termititenax_persephonae	+	+	+
Devosia_sp._SCN_66-27	+	+	+
Anaerobacillus_alkalidiazotrophicus	+	+	+
Fibrobacter_sp._UWB12	+	+	+
Paludibacter_sp.	+	+	+
Muricauda_lutimaris	+	+	+
Flavobacterium_kingsejongi	+	+	+
Duganella_sp._Leaf126	+	+	+
Desulfobacteraceae_bacterium_IS3	+	+	+
Maribacter_sp.	+	+	+
Petrotoga_olearia	+	+	+
Tenericutes_bacterium_GWC2_39_45	+	+	+
Leeuwenhoekiella_nanhaiensis	+	+	+
bacterium_(Candidatus_Blackallbacteria)_CG18_big_fil_WC_8	+	+	+
Schleiferia_sp._LA	+	+	+

Porphyromonas_sp._COT-239_OH1446	+	+	+
Methylomicrobium_buryatense	+	+	+
Bacillus_sp._FJAT-25509	-	+	+
Fibrella_aestuarina	-	+	+
Ornithinibacillus_halophilus	-	+	+
Paenibacillus_sp._FSL_R5-0765	-	+	+
Flavobacterium_gelidilacus	+	+	+
Bacillus_sp._FJAT-45037	+	+	+
Algoriphagus_sp._M8-2	+	+	+
Phaeodactylibacter_xiamenensis	+	+	+
Lysinibacillus_meyeri	+	+	+
Actinokineospora_bangkokensis	+	+	+
Bacillus_sp._AFS031507	-	+	+
Actinomyces_israelii	-	+	+
Capnocytophaga_sputigena	+	+	+
Bacillus_korlensis	+	+	+
Gemmatimonas_phototrophica	+	+	+
Gramella_portivictoriae	+	+	+
Enterococcus_sp._Marseille-P4358	+	+	+
Hymenobacteraceae_bacterium_SYSU_D60016	+	+	+
Actinomyces_sp._HMSC065F11	+	+	+
Pustulibacterium_marinum	-	+	+
Methylobacterium_variabile	-	+	+
Marinilactibacillus_sp._15R	+	+	+
Mycoplasma_hyosynoviae	+	+	+
Nodularia_sp._NIES-3585	+	+	+
Thermoflavimicrobium_dichotomicum	+	+	+
Ruminococcus_sp._CAG:57	+	+	+
Pedobacter_sp._OK291	+	+	+
Odoribacter_sp._AM16-33	+	+	+
Pedobacter_sp._ok626	+	+	+
Muricauda_beolgyonensis	+	+	+
Bacteroidetes_bacterium_GWF2_29_10	+	+	+
Streptomyces_sp._TSRI0281	+	+	+
Ignavigranum_ruoffiae	+	+	+
Sulfurimonas_sp._UBA12504	+	+	+
Bacteroidetes_bacterium_CG18_big_fil_WC_8_21_14_2_50_41	-	+	+
Gemella_asaccharolytica	-	+	+
Mucilaginibacter_mallensis	-	+	+
Bacillus_methanolicus	-	+	+
uncultured_bacterium_Contig15	-	+	+
Zhouia_amylolytica	+	+	+
Roseibium_sp._TrichSKD4	+	+	+
Candidatus_Peribacteria_bacterium_RIFCSPHIGHO2_01_FULL	+	+	+
Lactobacillus_parabuchneri	+	+	+
Anaerococcus_mediterraneensis	+	+	+
Rhodoplanes_elegans	-	+	+
Candidatus_Azambacteria_bacterium_GW2011_GWA1_44_9	-	+	+
Citrobacter_braakii	+	+	+
Bacillus_humi	+	+	+
Pedobacter_terrae	+	+	+
Acidobacteria_bacterium_RBG_16_68_9	+	+	+
Psychroflexus_tropicus	+	+	+
Muricauda_sp._NH166	-	+	+
Methanobrevibacter_woesei	-	+	+

Acinetobacter_sp._WCHAc060033	-	+	+
Sphingomonas_sanguinis	-	+	+
Deltaproteobacteria_bacterium_RBG_13_53_10	-	+	+
candidate_division_Zixibacteria_bacterium_HGW-Zixibacteria-[illegible]	-	+	+
Zetaproteobacteria_bacterium_CG1_02_49_23	-	+	+
Ferruginibacter_sp._BO-59	-	+	+
Niabella_yanshanensis	+	+	+
Flavobacteriaceae_bacterium_CG2_30_34_30	+	+	+
Bacillus_virus_Bobb	+	+	+
Mucilaginibacter_polytrichastri	+	+	+
Candidatus_Moduliflexus_flocculans	+	+	+
Gracilibacillus_kekensis	+	+	+
Paenibacillus_sp._1-18	+	+	+
Haemophilus_influenzae	+	+	+
Nitrobacter_hamburgensis	+	+	+
Rhodococcus_sp._06-418-5	+	+	+
Candidatus_Aureabacteria_bacterium_SURF_26	-	+	+
Taibaiella_soli	-	+	+
Candidatus_Levybacteria_bacterium_RIFCSPHIGHO2_01_FUI	-	+	+
Paenibacillus_ferrarius	+	+	+
Fibrobacter_sp._UWB2	+	+	+
Algoriphagus_locisalis	+	+	+
Marinobacter_fuscus	+	+	+
Verrucomicrobia_bacterium_S94	+	+	+
Desulfobacteraceae_bacterium_4572_89	+	+	+
Desulfobacterales_bacterium_PC51MH44	+	+	+
Salegentibacter_salinarum	-	+	+
Lactobacillus_formosensis	-	+	+
Mycobacterium_sp._BK558	-	+	+
Microgenomates_group_bacterium_GW2011_GWF1_44_10	-	+	+
Sphingomonas_sp._TF3	+	+	+
Sediminicola_luteus	+	+	+
Lactobacillus_xiangfangensis	-	+	+
[Bacillus]_sp._KCTC_13219	+	-	+
Ralstonia_phage_RsoP1EGY	+	-	+
Streptococcus_sp._DD11	+	-	+
Marinobacterium_lutimaris	+	-	+
Tenacibaculum_dicentrarchi	+	-	+
Caviibacter_abscessus	+	-	+
Aeromonas_sp._ASNIH3	+	-	+
Brochothrix_thermosphacta	+	-	+
Flavobacterium_sp._11	+	-	+
Halomonas_denitrificans	+	-	+
Lactococcus_phage_936_sensu_lato	+	-	+
Anoxybacillus_tepidamans	+	-	+
Vibrio_alginolyticus	+	-	+
Mycobacterium_pseudoshottsii	+	-	+
Clostridium_sp._1_1_41A1FAA	+	-	+
Sporolactobacillus_pectinivorans	+	-	+
Candidatus_Amoebophilus_sp._36-38	+	-	+
Janthinobacterium_sp._CG23_2	+	-	+
Lysinibacillus_macroides	+	-	+
Deltaproteobacteria_bacterium_RBG_16_44_11	+	-	+
Algicola_sagamiensis	+	-	+
Mucilaginibacter_pineti	+	-	+

Peptoniphilus_sp._KHD5	+	-	+
Bdellovibrio_bacteriovorus	+	-	+
Cenarchaeum_symbiosum	+	-	+
Gallionellales_bacterium_RIFCSPLOWO2_02_FULL_59_110	+	-	+
Chromatocurvus_sp._F02	+	-	+
Bodo_saltans_virus	+	-	+
Acidibacillus_sulfuroxidans	+	-	+
uncultured_rumen_bacterium	+	-	+
Bacillus_fortis	+	-	+
Helicobacter_sp._12S02232-10	+	-	+
Porphyromonas_sp.	+	-	+
Candidatus_Acetothermum_autotrophicum	+	-	+
Scytonema_sp._NIES-4073	-	-	+
Acinetobacter_sp._CFCC_10889	-	-	+
Marivirga_tractuosa	-	-	+
Lysinibacillus_sp._Marseille-P5727	-	-	+
Leuconostoc_citreum	-	-	+
Desulfatitalea_sp._BRH_c12	-	-	+
Frankia_sp._Cc1.17	-	-	+
Lactococcus_sp._S-13	-	-	+
Haemophilus_haemolyticus	-	-	+
Candidatus_Levybacteria_bacterium_GW2011_GWA2_36_13	-	-	+
Nocardioides_sp._YIM_ART13	-	-	+
Bacillus_alcalophilus	-	-	+
Nitrospirae_bacterium_GWC2_57_9	-	-	+
Bacillus_sp._MUM_116	-	-	+
Oceanobacillus_sp._YLB-02	-	-	+
Candidatus_Saccharibacteria_bacterium_32-45-3	-	-	+
Lysinimicrobium_luteum	-	-	+
Candidatus_Micrarchaeum_acidiphilum	-	-	+
Brachybacterium_faecium	-	-	+
Actinomyces_sp._oral_taxon_181	-	-	+
Methanosaeta_sp._PtaB.Bin039	-	-	+
Vibrio_phage_pTD1	-	-	+
candidate_division_WWE3_bacterium_GW2011_GWF2_42_42	-	-	+
Cystobacter_ferrugineus	-	-	+
Naumovozyma_castellii	-	-	+
Kazachstania_saulgeensis	-	-	+
Lactobacillus_kullabergensis	-	-	+
Shewanella_sp._Shew256	-	-	+
Sinorhizobium_americanum	-	-	+
Candidatus_Aenigmarchaeota_archaeon_CG1_02_38_14	-	-	+
Nitrospinae_bacterium_RIFCSPLOWO2_12_FULL_45_22	-	-	+
Geobacillus_sp._1017	-	-	+
Woeseia_oceani	-	-	+
Pseudoflavitalea_sp._5GH32-13	-	-	+
Pasteurella_multocida	+	+	+
Lachnospiraceae_bacterium_OM02-26	+	+	+
Paenibacillus_antarcticus	+	+	+
Butyricicoccus_sp._AF24-19AC	+	+	+
Bifiguratus_adelaidae	+	+	+
Bacillus_phage_vB_BpsS-140	-	+	+
Fusarium_fujikuroi	-	+	+
Lactobacillus_pobuzihii	+	+	+
Lentibacillus_sp._SSKP1-9	+	+	+

Paenibacillus_dauci	-	+	+
Puccinia_striiformis	+	+	+
Phage_5P_2	+	+	+
Firmicutes_bacterium_HGW-Firmicutes-20	+	+	+
Ruminococcus_sp._OM05-7	+	+	+
Gracilibacillus_boraciitolerans	+	+	+
Skermanella_aerolata	+	+	+
Bacillus_sp._NSP9.1	+	+	+
Deinococcus_grandis	-	+	+
Bacteroidetes_bacterium_MED-G21	+	+	+
Flavobacterium_branchiophilum	+	+	+
Bacillus_virus_CP51	+	+	+
Bacillus_sp._UNC438CL73TsuS30	+	+	+
Moraxellaceae_bacterium	+	+	+
Gillisia_sp._Hel1_33_143	-	+	+
Candidatus_Gastranaerophilales_bacterium_HUM_10	+	+	+
[Erwinia]_teleogrylli	-	+	+
Chryseobacterium_koreense	+	+	+
Nitrosospira_sp._NpAV	+	+	+
Cyclobacterium_marinum	+	+	+
Methanophagales_archaeon_ANME-1-THS	+	+	+
Olsenella_mediterranea	+	+	+
Desulfococcus_sp._4484_242	+	+	+
Imtechella_halotolerans	+	+	+
Allofustis_seminis	+	+	+
Wenyingzhuangia_fucanilytica	+	+	+
Clostridium_ventriculi	+	+	+
Capnocytophaga_haemolytica	+	+	+
Sphingobium_yanoikuyae	+	+	+
Gokushovirus_WZ-2015a	+	+	+
Lamprocystis_purpurea	+	+	+
Nitrospirae_bacterium_GWC2_46_6	+	+	+
Flavobacterium_sp._123	+	+	+
Atlantibacter_hermannii	-	+	+
Legionella_fairfieldensis	-	+	+
Ereboglobus_luteus	+	+	+
Mongoliibacter_ruber	+	+	+
Clostridium_sp._AM54-37XD	+	+	+
Phyllobacterium_zundukense	-	+	+
Neisseria_dumasiana	-	+	+
Deltaproteobacteria_bacterium_GWA2_47_9	+	+	+
Rubricoccus_marinus	+	+	+
Polaribacter_filamentus	+	+	+
Rhodobacter_sp._SW2	-	+	+
Anoxybacillus_ayderensis	-	+	+
Thermotogae_bacterium	-	+	+
Bacteroidetes_bacterium_HGW-Bacteroidetes-6	+	+	+
Acinetobacter_proteolyticus	+	+	+
Bacillus_notoginsengisoli	+	+	+
Flavobacteriaceae_bacterium_BH-SD17	+	+	+
Paenibacillus_lactis	+	+	+
Capnocytophaga_sp._oral_taxon_336	+	+	+
Bdellovibrionales_bacterium_RIFCSPHIGHO2_01_FULL_40_[illegible]	+	+	+
Candidatus_Nitrosotalea_devanaterra	+	+	+
Pedobacter_cryoconitis	+	+	+

Bacillus_sp._UNCCL81	-	+	+
Chryseobacterium_sp._52	-	+	+
Planktothrix_rubescens	-	+	+
Candidatus_Dadabacteria_bacterium	+	+	+
Anaerococcus_octavius	+	+	+
Chryseobacterium_sp._1_F178	+	+	+
Halobacillus_litoralis	+	+	+
Falsibacillus_sp._GY_10110	+	+	+
Gemella_morbillorum	+	+	+
Bacillus_sp._AFS076308	+	+	+
Carnobacterium_sp._17-4	+	+	+
Candidatus_Abyssubacteria_bacterium_SURF_17	+	+	+
Pseudarcicella_hirudinis	+	+	+
Cellulophaga_baltica	+	+	+
Streptococcus_entericus	-	+	+
Megasphaera_sp._DISK_18	-	+	+
Lutibacter_oricola	+	+	+
Flavobacterium_sp._KJJ	+	+	+
Candidatus_Fermentibacteria_bacterium	+	+	+
Sphingobacterium_sp._M46	+	+	+
Paenibacillus_illinoisensis	+	+	+
Lysinibacillus_halotolerans	+	+	+
Gammaproteobacteria_bacterium_CG12_big_fil_rev_8_21_14_(	+	+	+
Lechevalieria_aerocolonigenes	+	+	+
Rivicola_pingtungensis	-	+	+
Pirellula_staleyi	-	+	+
Micavibrio_sp._TMED27	-	+	+
Herbiconiux_solani	-	+	+
Tannerella_sp.	+	+	+
Clostridium_sp._CAG:1193	+	+	+
Gemella_sanguinis	+	+	+
Mariniradius_saccharolyticus	+	+	+
bacterium_TMED181	+	+	+
Solitalea_canadensis	+	+	+
Tetragenococcus_muriaticus	+	+	+
Marine_Group_II_euryarchaeote	+	+	+
Deltaproteobacteria_bacterium_CG11_big_fil_rev_8_21_14_0_	+	+	+
Bacillus_sp._SYSU_K30001	-	+	+
Bacillus_sp._OV194	-	+	+
Candidatus_Falkowbacteria_bacterium_RIFCSPLOWO2_02_FU	-	+	+
Abiotrophia_defectiva	-	+	+
Porphyromonas_sp._31_2	+	+	+
Cellvibrio_sp._79	+	+	+
Bacillus_ndiopicus	+	+	+
Idiomarina_aquatica	+	+	+
Actinomyces_glycerinitolerans	+	+	+
Planctomycetes_bacterium_GWF2_41_51	+	+	+
Spirochaetae_bacterium_HGW-Spirochaetae-7	+	+	+
Tetrasphaera_phage_TJE1	+	+	+
Mycoplasma_spumans	-	+	+
Paenibacillus_wulumuqiensis	-	+	+
Streptococcus_pluranimalium	-	+	+
Streptococcus_sp._DD13	-	+	+
Candidatus_Sulfopaludibacter_sp._SbA4	-	+	+
Mangrovicoccus_ximenensis	-	+	+

Prosthecochloris_sp._CIB_2401	+	+	+
Amphibacillus_marinus	+	+	+
Methylotenera_sp._G11	-	+	+
Bacillus_sp._MZGC1	-	+	+
bacterium_A37T11	+	-	+
Paenibacillus_sp._URHA0014	+	-	+
Andreprevotia_chitinilytica	+	-	+
Parolsenella_catena	+	-	+
Myroides_odoratimimus	+	-	+
Roseivirga_seohaensis	+	-	+
Candidatus_Woesearchaeota_archaeon_CG1_02_47_18	+	-	+
Zetaproteobacteria_bacterium	+	-	+
Hydrogenivirga_sp._128-5-R1-1	+	-	+
Bifidobacterium_criceti	+	-	+
uncultured_bacterium_Contigcl_1523	+	-	+
Photobacterium_ganghwense	+	-	+
Bacillus_funiculus	+	-	+
Coprobacillus_sp._AM09-26	+	-	+
Methylobacterium_mesophilicum	+	-	+
Gallionella_capsiferriformans	+	-	+
Alkalibacterium_subtropicum	+	-	+
Serratia_proteamaculans	+	-	+
Sulfurospirillum_sp._UCH001	+	-	+
Neptuniibacter_caesariensis	+	-	+
Paramicrosporidium_saccamoebae	+	-	+
Mycoplasma_lipofaciens	+	-	+
Campylobacter_corcagiensis	+	-	+
Stappia_stellulata	+	-	+
Candidatus_Gastranaerophilales_bacterium_HUM_11	+	-	+
Listeria_welshimeri	+	-	+
Bacillus_sp._AFS073361	+	-	+
Aquimarina_megaterium	+	-	+
Conservatibacter_flavescens	+	-	+
SAR86_cluster_bacterium	+	-	+
Bradyrhizobium_sp._SUTN9-2	+	-	+
Dictyobacter_aurantiacus	+	-	+
Bacillus_sp._AFS041924	+	-	+
Candidatus_Marinamargulisbacteria_bacterium_SCGC_AG-414	+	-	+
Chitinophaga_ginsengisoli	+	-	+
Candidatus_Altiarchaeales_archaeon_WOR_SM1_79	+	-	+
Methanobrevibacter_sp._YE315	+	-	+
Clostridia_bacterium_UC5.1-2G4	-	-	+
Paenibacillus_sp._GM2FR	-	-	+
Micavibrio_sp._TMED2	-	-	+
Magnetospirillum_sp._64-120	-	-	+
Geobacillus_sp._PA-3	-	-	+
Parapedobacter_luteus	-	-	+
Saccharibacillus_sp._O16	-	-	+
Thalassobium_sp._R2A62	-	-	+
Helicobacter_pametensis	-	-	+
Sulfurivirga_caldicuralii	-	-	+
Vibrio_fluvialis	-	-	+
Brevundimonas_sp.	-	-	+
Geodermatophilus_sp._DSM_45219	-	-	+
Hydrogenimonas_sp.	-	-	+

Candidatus_Terrybacteria_bacterium_RIFCSPHIGHO2_02_41_	-	-	+
Microgenomates_group_bacterium_RBG_16_45_19	-	-	+
Actinomadura_latina	-	-	+
Oerskovia_enterophila	-	-	+
Carnobacterium_alterfunditum	-	-	+
SAR86_cluster_bacterium_SAR86B	-	-	+
Yersinia_enterocolitica	-	-	+
Nocardiopsis_sp._TSRI0078	-	-	+
Bartonella_sp._OE_1-1	-	-	+
beta_proteobacterium_AAP51	-	-	+
Sphingomonas_sp._Cra20	-	-	+
Halomonas_nigrificans	-	-	+
Rhodoluna_lacicola	-	-	+
Weissella_confusa	-	-	+
Ruminococcaceae_bacterium_R-25	-	-	+
Lactococcus_phage_1706	-	-	+
Candidatus_Gottesmanbacteria_bacterium_RBG_13_45_10	-	-	+
Thalassotalea_sp._PP2-459	-	-	+
Burkholderia_vietnamiensis	-	-	+
Anaerolineae_bacterium_49_20	-	-	+
Parcubacteria_group_bacterium_RIFCSPHIGHO2_01_FULL_4	-	-	+
Deltaproteobacteria_bacterium_RBG_19FT_COMBO_46_12	-	-	+
Brevibacillus_sp._WF146	-	-	+
Candidatus_Acetothermia_bacterium	-	-	+
Periconia_macrospinosa	-	-	+
Flavobacterium_sp._YO12	-	-	+
Amorphus_coralli	-	-	+
candidate_division_WWE3_bacterium_CG08_land_8_20_14_0_	-	-	+
Candidatus_Moranbacteria_bacterium_CG23_combo_of_CG06-	-	-	+
Dermabacter_hominis	-	-	+
Pestivirus_A	+	+	+
Lactobacillus_kisonensis	+	+	+
Choristoneura_rosaceana_entomopoxvirus	-	+	+
Candidatus_Scalindua_rubra	+	+	+
Blyttiomyces_helicus	+	+	+
Edwardsiella_hoshinae	+	+	+
Thamnocephalis_sphaerospora	+	+	+
Mortierella_elongata	+	+	+
Tissierellia_bacterium_S5-A11	+	+	+
Gracilibacillus_ureilyticus	+	+	+
Bacillus_sp._J13	+	+	+
Spirosoma_endophyticum	+	+	+
Mariniflexile_sp._TRM1-10	+	+	+
Blastopirellula_sp.	+	+	+
Pseudomonas_sp._ARP3	+	+	+
Paenibacillus_sp._IHBB_10380	+	+	+
Magnetococcus_marinus	+	+	+
Streptococcus_sp._HMSC072G04	-	+	+
Legionella_sp.	+	+	+
Algibacter_alginicilyticus	+	+	+
Firmicutes_bacterium_ZOR0006	+	+	+
Rhizobium_sp._AAP43	+	+	+
Pseudomonas_segetis	-	+	+
Pseudomonas_sp._MOIL14HWK12:I2	-	+	+
Bacillus_kribbensis	-	+	+

Clostridium_haemolyticum	+	+	+
Arthrobacter_sp._Bz4	+	+	+
Flavobacterium_sp._ICH-30	+	+	+
uncultured_firmicutes_bacterium_contig_31	+	+	+
uncultured_bacterium_Contig1771_n_1784_cl	+	+	+
Mycobacterium_sp._M26	+	+	+
Mycolicibacterium_conceptionense	-	+	+
uncultured_bacterium_Contig1586	+	+	+
Fastidiosibacteraceae_bacterium_SYSU_SYW-5	+	+	+
Photobacterium_damselae	+	+	+
Bifidobacterium_eulemuris	+	+	+
Comamonas_testosteroni	+	+	+
Streptococcus_orisratti	+	+	+
Methyloversatilis_universalis	-	+	+
Mycoplasma_penetrans	+	+	+
Helicobacter_sp._48519	+	+	+
uncultured_bacterium_Contigcl_1556	+	+	+
Olsenella_sp._GAM18	+	+	+
Exiguobacterium_sp._HF60	+	+	+
Vibrio_vulnificus	+	+	+
Sediminitomix_flava	+	+	+
Coprococcus_sp._43_8	+	+	+
Micromonospora_sp._NGC1-4	+	+	+
Bacteroidetes_bacterium_GWE2_42_24	+	+	+
Bacillus_sp._FJAT-27916	+	+	+
Corallococcus_sp._AB011P	+	+	+
Candidatus_Saccharibacteria_bacterium_47-87	+	+	+
Listeria_newyorkensis	+	+	+
Elusimicrobia_bacterium_GWA2_62_23	-	+	+
Bifidobacterium_angulatum	-	+	+
Bdellovibrionales_bacterium_RIFOXYB2_FULL_36_6	-	+	+
Lentisphaerae_bacterium	+	+	+
Chryseobacterium_scophthalmum	+	+	+
Pedobacter_sp._eg	+	+	+
Tamlana_sp._UJ94	+	+	+
Mastigocladopsis_repens	+	+	+
Caryophanon_latum	+	+	+
Sulfobacillus_benefaciens	+	+	+
Pseudoscardovia_radai	+	+	+
Eubacterium_sp._AF15-50	+	+	+
Aerococcus_sp._1KP-2016	+	+	+
Candidatus_Melainabacteria_bacterium_GWF2_37_15	+	+	+
Coraliomargarita_sp._CAG:312	+	+	+
Anoxybacillus_flavithermus	+	+	+
Leptospira_interrogans	+	+	+
uncultured_phage	+	+	+
Bacillus_flexus	+	+	+
Thermodesulfobacterium_geofontis	+	+	+
Bacillus_cavernae	+	+	+
Lentisphaerae_bacterium_RIFOXYC12_FULL_60_16	-	+	+
archaeon_BMS3Bbin15	-	+	+
Mucilaginibacter_sp._L294	+	+	+
Pseudomonas_fragi	+	+	+
Niastella_koreensis	+	+	+
Gramella_gaetbulicola	+	+	+

Polaribacter_tangerinus	+	+	+
Acetobacter_cerevisiae	+	+	+
Flavobacterium_sp._WWJ-16	+	+	+
Salegentibacter_salarius	+	+	+
Pelagibacteraceae_bacterium_GOM-A5	+	+	+
Propionibacterium_freudenreichii	+	+	+
Starkeya_novella	+	+	+
Anaerococcus_sp._Marseille-P3557	+	+	+
Jeotgalibacillus_alimentarius	-	+	+
Lactobacillus_suebicus	-	+	+
Pseudothermotoga_lettingae	-	+	+
gamma_proteobacterium_L18	-	+	+
Candidatus_Woesearchaeota_archaeon_CG10_big_fil_rev_8_21	-	+	+
Clostridium_sp._CAG:813	+	+	+
Coprobacillus_sp._8_1_38FAA	+	+	+
Lactobacillus_sp._UMNPBX3	+	+	+
Ancylomarina_sp._SHSM-M15	+	+	+
Paraburkholderia_fungorum	+	+	+
Eubacterium_sp._3_1_31	+	+	+
Sphingobacterium_faecium	+	+	+
Magnetococcales_bacterium_UR-1	+	+	+
Gammaproteobacteria_bacterium_RIFCSPHIGHO2_12_FULL_	+	+	+
Lysinibacillus_sp._2017	-	+	+
Polaribacter_vadi	-	+	+
Bacillus_acanthi	-	+	+
Cardiobacterium_hominis	-	+	+
Ignavibacteria_bacterium_GWC2_35_8	-	+	+
Porphyromonas_sp._COT-290_OH860	-	+	+
Vagococcus_fessus	+	+	+
Aquimarina_agarivorans	+	+	+
Flavobacteriales_bacterium_BRH_c54	+	+	+
Paenibacillus_rubinfantis	+	+	+
Lactobacillus_ceti	+	+	+
Lactobacillus_cacaonum	+	+	+
Steroidobacter_agariperforans	+	+	+
Streptobacillus_hongkongensis	+	+	+
Burkholderia_contaminans	+	+	+
Chloroflexi_bacterium_RBG_16_54_18	+	+	+
Pedobacter_insulae	+	+	+
Leptotrichia_hofstadii	+	+	+
Candidatus_Ryanbacteria_bacterium_RIFCSPHIGHO2_01_FUI	+	+	+
Arthrobacter_castelli	+	+	+
Bifidobacterium_aemilianum	+	+	+
Candidatus_Schekmanbacteria_bacterium_RBG_13_48_7	+	+	+
Collinsella_sp._AM17-1	+	+	+
bacterium_42_11	+	+	+
Bacillus_sp._YLB-04	+	+	+
Methanoculleus_sediminis	+	+	+
Persicobacter_sp._JZB09	+	+	+
Crocinitomicaceae_bacterium_TMED209	+	+	+
Bacillus_sp._FJAT-29814	+	+	+
Paenibacillus_virus_Hb10c2	-	+	+
Thalassospira_profundimaris	-	+	+
Corallococcus_sp._AB004	-	+	+
Mastigocoleus_testarum	-	+	+

Thermosporothrix_hazakensis	-	+	+
Candidatus_Curtissbacteria_bacterium_RIFCSPLOWO2_12_FU	-	+	+
Natranaerobius_trueperi	-	+	+
Spirosoma_sp._HMF3257	-	+	+
Bartonella_elizabethae	-	+	+
Helicobacter_apodemus	-	+	+
Clostridia_bacterium_UC5.1-2H6	+	+	+
Lactobacillus_vaginalis	+	+	+
Candidatus_Nomurabacteria_bacterium_RIFCSPHIGHO2_01_F	+	+	+
Aeromonas_encheleia	+	+	+
Limnochorda_pilosa	+	+	+
Kallipyga_gabonensis	+	+	+
Lactobacillus_perolens	+	+	+
Campylobacter_blaseri	+	+	+
Candidatus_Kuenenia_stuttgartiensis	+	+	+
Bacillus_velezensis	+	+	+
bacterium_BMS3Bbin07	+	+	+
Aliifodinibius_roseus	-	+	+
Burkholderia_gladioli	-	+	+
Streptomyces_sp._NRRL_F-5053	-	+	+
Paenisporosarcina_quisquiliarum	-	+	+
Bacillus_freudenreichii	-	+	+
Candidatus_Arthromitus_sp._SFB-mouse-NL	-	+	+
Pediococcus_argentinicus	-	+	+
Clostridiales_bacterium_VE202-29	+	+	+
Methanosphaera_sp._rholeuAM6	+	+	+
Candidatus_Magasanikbacteria_bacterium_GW2011_GWA2_45	-	+	+
filamentous_cyanobacterium_ESFC-1	+	-	+
Verrucomicrobia_bacterium_61-8	+	-	+
Fimbriiglobus_ruber	+	-	+
Fusobacteriia_bacterium_4572_74	+	-	+
Ignavibacteria_bacterium_GWA2_35_8	+	-	+
Maribacter_cobaltidurans	+	-	+
Bacillus_phage_VMY22	+	-	+
Clostridium_sp._AF17-2	+	-	+
candidate_division_TM6_bacterium_GW2011_GWF2_36_131	+	-	+
Nostoc_sp._PCC_7524	+	-	+
Elusimicrobia_bacterium_HGW-Elusimicrobia-3	+	-	+
Lactobacillus_brantae	+	-	+
Candidatus_Lumbricidophila_eiseniae	+	-	+
Deltaproteobacteria_bacterium_HGW-Deltaproteobacteria-6	+	-	+
Sulfurovum_sp._FS06-10	+	-	+
Sporolactobacillus_vineae	+	-	+
Streptococcus_sp._HMSC034E12	+	-	+
Mesorhizobium_sp._WSM3873	+	-	+
Conchiformibius_steedae	+	-	+
Aquifex_aeolicus	+	-	+
Lampropedia_cohaerens	+	-	+
Oceanimonas_doudoroffii	+	-	+
Lactobacillus_paracollinoides	+	-	+
Anaerolineae_bacterium_UTCFX2	+	-	+
Candidatus_Similichlamydia_laticola	+	-	+
Halanaerobium_sp._T82-1	+	-	+
Thermococci_archaeon	+	-	+
Syntrophaceae_bacterium_PtaB.Bin038	+	-	+

Candidatus_Gottesmanbacteria_bacterium_RIFCSPLOWO2_01_	+	-	+
Streptomyces_sp._NEAU-YY642	+	-	+
Candidatus_Aminicenantes_bacterium_RBG_13_63_10	+	-	+
Streptococcus_virus_Cp1	+	-	+
Bacillus_phage_vB_BcoS-136	+	-	+
Leptolyngbya_sp.	+	-	+
Marinobacter_sp._EVN1	+	-	+
Alicyclobacillus_ferrooxydans	+	-	+
Leptospirillum_ferrooxidans	+	-	+
Helicobacter_sp._10-6591	+	-	+
Anaerolineae_bacterium_SM23_84	+	-	+
Bordetella_genomosp._13	+	-	+
Mycolicibacterium_chitae	+	-	+
Mucilaginibacter_sp._94	+	-	+
Streptoalloteichus_hindustanus	+	-	+
Halobacillus_aidingensis	+	-	+
Geobacillus_thermocatenulatus	-	-	+
Macrococcus_goetzii	-	-	+
Chryseolinea_serpens	-	-	+
Bacillus_sp._AFS018417	-	-	+
Desulfatibacillum_aliphaticivorans	-	-	+
Thermosulfidibacter_takaii	-	-	+
Thiomicrospira_pelophila	-	-	+
Polaromonas_naphthalenivorans	-	-	+
Candidatus_Puniceispirillum_marinum	-	-	+
Thiothrix_lacustris	-	-	+
Leucobacter_chromiireducens	-	-	+
Gracilibacillus_orientalis	-	-	+
Neptunomonas_phycophila	-	-	+
Thermodesulfatator_atlanticus	-	-	+
Glaciecola_punicea	-	-	+
Globicatella_sp._HMSC072A10	-	-	+
Omnitrophica_WOR_2_bacterium_RIFCSPHIGHO2_02_FULL	-	-	+
bacterium_BMS3Abin09	-	-	+
Pedosphaera_sp._Tous-C6FEB	-	-	+
Sinorhizobium_sp._Sb3	-	-	+
Candidatus_Collierbacteria_bacterium_RIFCSPHIGHO2_02_FU	-	-	+
bacterium_(Candidatus_Gribaldobacteria)_CG_4_10_14_0_2_u	-	-	+
Calothrix_sp._NIES-2100	-	-	+
Frankia_sp._EI5c	-	-	+
Dialister_sp._CAG:357	-	-	+
Campylobacterales_bacterium_16-40-21	-	-	+
Nocardiopsis_synnemataformans	-	-	+
Candidatus_Levybacteria_bacterium_RIFCSPHIGHO2_01_FUL	-	-	+
Nocardioides_sp._J54	-	-	+
Paraburkholderia_piptadeniae	-	-	+
Enterobacter_ludwigii	-	-	+
Gammaproteobacteria_bacterium_GWE2_37_16	-	-	+
Paraburkholderia_sp._NEAU-SY24	-	-	+
Parcubacteria_group_bacterium_GW2011_GWF2_43_11	-	-	+
candidate_division_Kazan_bacterium_RBG_13_50_9	-	-	+
Parcubacteria_group_bacterium_RIFCSPHIGHO2_02_FULL_4	-	-	+
Aeromonas_caviae	-	-	+
Oceanobacillus_timonensis	-	-	+
candidate_division_WS6_bacterium_34_10	-	-	+

Malonomonas_rubra	-	-	+
Herbaspirillum_autotrophicum	-	-	+
Streptacidiphilus_jiangxiensis	-	-	+
Terribacillus_goriensis	-	-	+
Bacilli_bacterium_VT-13-104	-	-	+
Bacillus_sp._L27	-	-	+
Tabrizicola_aquatica	-	-	+
Arthrobacter_sp._Soil762	-	-	+
Moraxella_porci	-	-	+
Sulfobacillus_sp._hq2	-	-	+
Vibrio_sp._OY15	-	-	+
Streptomyces_sp._CB02009	-	-	+
Demequina_flava	-	-	+
Kangiella_sp.	-	-	+
Mucilaginibacter_oryzae	-	-	+
Thermoplasmatales_archaeon_ex4572_165	-	-	+
Mycobacterium_sp._AT1	-	-	+
Sphingobium_sp._D43FB	-	-	+
Spirosoma_linguale	-	-	+
Leptotrichia_sp._oral_taxon_498	-	+	+
Gonapodya_prolifera	+	+	+
Clostridium_sp._IBUN125C	+	+	+
Paucisalibacillus_sp._EB02	+	+	+
Firmicutes_bacterium_HGW-Firmicutes-15	+	+	+
Diversispora_versiformis	+	+	+
Syncephalis_pseudoplumigaleata	+	+	+
Methanomicrobium_mobile	+	+	+
Gemella_bergeri	-	+	+
Romboutsia_hominis	+	+	+
Dermabacter_vaginalis	+	+	+
Zancudomyces_culisetae	+	+	+
Melomys_burtoni_retrovirus	+	+	+
Arcobacter_sp.	+	+	+
Aneurinibacillus_soli	+	+	+
Globicatella_sulfidifaciens	+	+	+
Candidatus_Altiarchaeales_archaeon	+	+	+
Calocera_cornea	-	+	+
Lysinibacillus_sp._LK3	+	+	+
Olegusella_massiliensis	+	+	+
Clostridium_mediterraneense	+	+	+
Methanolobus_tindarius	+	+	+
bacterium_HR26	+	+	+
Parcubacteria_group_bacterium_GW2011_GWC1_39_29	+	+	+
Acinetobacter_sp._CIP_102637	-	+	+
Paenibacillus_sp._A9	-	+	+
Candidatus_Uhrbacteria_bacterium_GW2011_GWD2_52_7	-	+	+
Spirochaetes_bacterium_GWE1_32_154	+	+	+
Nubsella_zeaxanthinifaciens	+	+	+
Idiomarina_homiensis	-	+	+
Candidatus_Melainabacteria_bacterium_GWA2_34_9	+	+	+
Burkholderia_dabaoshanensis	+	+	+
Cupriavidus_sp._UYMMa02A	+	+	+
Muricauda_sp._72	+	+	+
Aquimarina_sp._AU119	+	+	+
Arc_I_group_archaeon_ADurb1113_Bin01801	+	+	+

Stenotrophomonas_panacihumi	-	+	+
Elusimicrobia_bacterium_CG22_combo_CG10-13_8_21_14_all	-	+	+
Parcubacteria_group_bacterium_GW2011_GWA1_47_8	+	+	+
Coprobacillus_sp._AF33-1AC	+	+	+
Rhodobacteraceae_bacterium_HLUCCO18	+	+	+
Pleomorphomonas_diazotrophica	+	+	+
Lactobacillus_sp._247-3	+	+	+
uncultured_bacterium_Contig3b	+	+	+
Domibacillus_robiginosus	+	+	+
Methanobacterium_arcticum	+	+	+
Nitrosopumilales_archaeon	+	+	+
Dickeya_aquatica	-	+	+
Mariniphaga_anaerophila	+	+	+
Desulfococcus_oleovorans	+	+	+
Pleurocapsa_minor	+	+	+
Pusillimonas_sp._T2	+	+	+
Gramella_sp._SH35	+	+	+
Corynebacterium_glutamicum	+	+	+
Dyadobacter_sp._Leaf189	+	+	+
Paenarthrobacter_nicotinovorans	+	+	+
uncultured_Bacteroides_sp._SMG1	-	+	+
Microbacterium_sp._CSI-V	-	+	+
Oceanobacillus_jeddahense	+	+	+
Actinobacteria_bacterium_RBG_13_35_12	+	+	+
Pontibacillus_marinus	+	+	+
Schleiferia_thermophila	+	+	+
Arenibacter_troitsensis	+	+	+
Collinsella_sp._TF08-11AT	+	+	+
Muricauda_sp._GCL-11	-	+	+
Anaerosphaera_sp._HMSC064C01	-	+	+
Lactococcus_sp._1JSPR-7	+	+	+
Ruminococcus_sp._AM22-13	+	+	+
Prevotella_sp._BV3P1	+	+	+
Ornithinibacillus_californiensis	+	+	+
Legionella_birminghamensis	+	+	+
Winogradskyella_psychrotolerans	+	+	+
Microbacterium_sp.	+	+	+
Eastern_grey_kangaroopox_virus	-	+	+
Rhizobium_sp._Leaf384	-	+	+
Fimbriimonas_ginsengisoli	+	+	+
Epulopiscium_sp._AS2M-Bin002	+	+	+
Tenacibaculum_holothuriorum	+	+	+
Planctomycetes_bacterium_GWF2_50_10	+	+	+
Bacteroidetes_bacterium_GWA2_30_7	+	+	+
Nonlabens_ulvanivorans	+	+	+
Azospirillum_sp._TSH64	+	+	+
Caulobacter_sp._Root655	+	+	+
Cyanobacteria_bacterium_J069	+	+	+
Cytophagaceae_bacterium_FSY-15	-	+	+
Lutibacter_agarilyticus	-	+	+
Aureispira_sp._CCB-QB1	-	+	+
Geobacillus_subterraneus	+	+	+
Mycobacterium_virus_BBPiebs31	+	+	+
Yersinia_ruckeri	+	+	+
Achromobacter_spanius	+	+	+

Aggregatibacter_kilianii	+	+	+
Bacteroidetes_bacterium_GWA2_33_15	+	+	+
Murine_osteosarcoma_virus	+	+	+
Paraliobacillus_sediminis	+	+	+
Marinobacter_daqiaonensis	+	+	+
Golovinomyces_cichoracearum	-	+	+
Roseomonas_aerilata	-	+	+
Proteobacteria_bacterium_TMED154	-	+	+
Bacillus_sp._MUM_13	-	+	+
Chryseobacterium_sp._G0240	-	+	+
Candidatus_Gastranaerophilus_sp._(ex_Termes_propinquus)	+	+	+
Duodenibacillus_massiliensis	+	+	+
Lactobacillus_sp._143-4(a)	+	+	+
Aeromonas_veronii	+	+	+
Lactobacillus_frumenti	+	+	+
Flavobacterium_sp._J27	+	+	+
Capnocytophaga_sp._oral_taxon_332	+	+	+
Moniliophthora_perniciosa	+	+	+
Mycolicibacterium_rhodesiae	+	+	+
Spirochaeta_thermophila	+	+	+
Pseudomonas_mendocina	+	+	+
Planctomycetaceae_bacterium	+	+	+
Chloroflexi_bacterium_RBG_16_52_11	+	+	+
Enterococcus_canintestini	+	+	+
Lactobacillus_crustorum	+	+	+
Azospirillum_sp._CFH_70021	+	+	+
Cuspidothrix_issatschenkoi	+	+	+
Candidatus_Thiodiazotropha_endoloripes	-	+	+
Pedosphaera_sp.	-	+	+
Kluyvera_georgiana	-	+	+
Plautia_stali_symbiont	-	+	+
Francisella_noatunensis	-	+	+
Lactobacillus_mellifer	-	+	+
Desulfurobacterium_indicum	-	+	+
Lactobacillus_hominis	+	+	+
Bacillus_sp._UMB0899	+	+	+
Lactobacillus_iners	+	+	+
Candidatus_Woesearchaeota_archaeon	+	+	+
endosymbiont_of_Lamellibrachia_luymesi	+	+	+
Streptococcus_sobrinus	+	+	+
Croceibacter_atlanticus	+	+	+
Vibrio_galatheae	+	+	+
Flavobacterium_segetis	+	+	+
Flavobacterium_marinum	+	+	+
Leptotrichia_buccalis	+	+	+
Azoarcus_sp._KH32C	+	+	+
Kurthia_sibirica	+	+	+
Pedobacter_nyackensis	+	+	+
Paenibacillus_sp._UNC217MF	-	+	+
Tessaracoccus_oleiagri	-	+	+
Malikia_granosa	-	+	+
Actinomadura_atramentaria	-	+	+
Betaproteobacteria_bacterium_RIFCSPLOWO2_02_FULL_65_	-	+	+
Alicyclobacillus_shizuokensis	-	+	+
Thalassobacillus_devorans	-	+	+

Bacillus_marmarensis	-	+	+
Zunongwangia_mangrovi	-	+	+
Vibrio_superstes	-	+	+
Emcibacter_congregatus	+	-	+
Clostridium_sp._CAG_433_25_7	+	-	+
Sutterella_sp._AM11-39	+	-	+
Epulopiscium_sp._Nele67-Bin005	+	-	+
Algoriphagus_sp._A40	+	-	+
uncultured_Desulfobacterium_sp.	+	-	+
Campylobacter_sp._P0109	+	-	+
Acidobacteriaceae_bacterium_KBS_96	+	-	+
Bacillus_sp._FJAT-45350	+	-	+
Pseudodesulfovibrio_sp._S3	+	-	+
Petrotoga_sp._Shatin.DS.tank11.9.2.9.3	+	-	+
Vagococcus_carniphilus	+	-	+
Methanosphaera_sp._rholeuAM74	+	-	+
Collinsella_phocaeensis	+	-	+
Pedobacter_arcticus	+	-	+
Marinobacter_sp._T13-3	+	-	+
Listeria_grayi	+	-	+
Bartonella_vinsonii	+	-	+
Spirochaeta_lutea	+	-	+
Erysipelotrichaceae_bacterium_AF15-26LB	+	-	+
Negadavirga_sp._SW125	+	-	+
Lactobacillus_sunkii	+	-	+
Bacillus_kochii	+	-	+
Moraxella_cuniculi	+	-	+
Synergistales_bacterium_57_84	+	-	+
Helicobacter_muridarum	+	-	+
Fusobacterium_gonidiaformans	+	-	+
Croceibacter_phage_P2559S	+	-	+
Azospirillum_sp._TSO22-1	+	-	+
Ruminococcus_sp._AM27-27	+	-	+
Flavobacterium_sp._81	+	-	+
Staphylococcus_sp._HMSC070D05	+	-	+
Lysinibacillus_sp._BF-4	+	-	+
Ignavibacteriales_bacterium_UTCHB1	+	-	+
uncultured_bacterium_fosmid_pJB84G2	+	-	+
Ornithinimicrobium_sp._KCTC_49018	+	-	+
Chishuiella_changwenlii	+	-	+
Planococcus_faecalis	+	-	+
Gallibacterium_anatis	+	-	+
Calothrix_sp._336/3	+	-	+
Commensalibacter_intestini	+	-	+
Parvularculaceae_bacterium	+	-	+
Staphylococcus_caprae	+	-	+
Legionella_clemsonensis	+	-	+
Paenisporosarcina_sp._OV554	+	-	+
Rhodopila_globiformis	+	-	+
Clavibacter_michiganensis	+	-	+
Thermodesulfobacteria_bacterium	+	-	+
Devosia_sp._YR412	+	-	+
Thiobacillus_denitrificans	+	-	+
Actinomyces_polynesiensis	+	-	+
Hydrogenibacillus_schlegelii	+	-	+

Streptococcus_cuniculi	+	-	+
Paracoccus_sp._DSL-16	+	-	+
Streptomyces_humi	+	-	+
bacterium_TMED144	+	-	+
Methanococcus_vannielii	+	-	+
Nitrospira_japonica	+	-	+
Campylobacter_ornithocola	+	-	+
Sporosarcina_sp._P18a	+	-	+
Neisseria_macacae	+	-	+
Azoarcus_sp._BH72	+	-	+
Candidatus_Falkowbacteria_bacterium_RIFOXYC2_FULL_48_	+	-	+
Enterobacter_sp._R1(2018)	+	-	+
Flavobacterium_sasangense	+	-	+
Candidatus_Cryosericum_hinesii	+	-	+
Candidatus_Omnitrophica_bacterium_4484_70.1	+	-	+
Acidobacteriaceae_bacterium_URHE0068	-	-	+
Elusimicrobia_bacterium_GWA2_51_34	-	-	+
Streptococcus_sp._DD04	-	-	+
Arenibacter_sp._GUO666	-	-	+
Sphingosinicella_microcystinivorans	-	-	+
Burkholderia_insecticola	-	-	+
Ruminococcus_sp._AF25-17	-	-	+
Sediminibacterium_salmoneum	-	-	+
Acinetobacter_brisouii	-	-	+
Thermoplasmata_archaeon_M9B2D	-	-	+
Staphylococcus_sp._ZWU0021	-	-	+
Acidocella_aminolytica	-	-	+
Photobacterium_profundum	-	-	+
Abyssicoccus_albus	-	-	+
Methanoregulaceae_archaeon_PtaB.Bin108	-	-	+
Candidatus_Verstraetearchaeota_archaeon	-	-	+
Vanderwaltozyma_polyspora	-	-	+
Geothermobacter_sp._HR-1	-	-	+
Boseongicola_aestuarii	-	-	+
Flavobacterium_sp._AG291	-	-	+
Deltaproteobacteria_bacterium_RIFOXYD12_FULL_53_23	-	-	+
Spirochaetaceae_bacterium_4572_7	-	-	+
Aquimarina_macrocephali	-	-	+
Promicromonospora_sukumoe	-	-	+
Citrobacter_rodentium	-	-	+
Methylorubrum_populi	-	-	+
Planococcus_sp._Y42	-	-	+
Candidatus_Portnoybacteria_bacterium_RBG_19FT_COMBO_[illegible]	-	-	+
Pantoea_sp._aB	-	-	+
Burkholderiales_bacterium_RIFOXYC12_FULL_60_6	-	-	+
Mesorhizobium_sp._M7D.F.Ca.US.005.01.1.1	-	-	+
Pseudonocardia_acaciae	-	-	+
candidate_division_WWE3_bacterium_CG10_big_fil_rev_8_21	-	-	+
Vibrio_sonorensis	-	-	+
Candidatus_Omnitrophica_bacterium_CG1_02_40_15	-	-	+
Candidatus_Doudnabacteria_bacterium_RIFCSPHIGHO2_01_5	-	-	+
Leuconostoc_suionicum	-	-	+
Cyanobacterium_sp._HL-69	-	-	+
Bosea_thiooxidans	-	-	+
Pseudoxanthomonas_wuyuanensis	-	-	+

Candidatus_Cyclonatronum_proteinivorum	-	-	+
Streptomyces_turgidiscabies	-	-	+
Campylobacter_phage_PC5	-	-	+
Maribacter_sp._MAR_2009_60	-	-	+
Candidatus_Peregrinibacteria_bacterium_RIFOXYA2_FULL_3	-	-	+
Candidatus_Phytoplasma_ziziphi	-	-	+
Type-E_symbiont_of_Plautia_stali	-	-	+
Pseudomonas_jessenii	-	-	+
Deltaproteobacteria_bacterium_RBG_13_43_22	-	-	+
Legionella_anisa	-	-	+
Candidatus_Komeilibacteria_bacterium_CG11_big_fil_rev_8_2	-	-	+
Candidatus_Beckwithbacteria_bacterium_CG23_combo_of_CG	-	-	+
Burkholderiales_bacterium_PBB1	-	-	+
Candidatus_Levybacteria_bacterium_CG10_big_fil_rev_8_21_1	-	-	+
Enterococcus_quebecensis	-	-	+
Pseudomonas_sp._NFPP15	-	-	+
Lactobacillus_kosoi	-	-	+
Candidatus_Levybacteria_bacterium_RBG_16_35_11	-	-	+
Bacillus_acidiproducens	-	-	+
Polaromonas_sp._AET17H-212	-	-	+
Pseudomonas_sp._NFACC13-1	-	-	+
Nitrosospira_sp._Nsp44	-	-	+
Cellulomonas_sp._NEAU-TCZ24	-	-	+
Rothia_mucilaginosa	-	-	+
Aerococcus_suis	-	-	+
Weissella_sp._DD23	-	-	+
Chitinilyticum_aquatile	-	-	+
Moraxella_sp._VT-16-12	-	-	+
Bacillus_sp._OV322	-	-	+
Nocardiopsis_sp._SBT366	-	-	+
Streptobacillus_moniliformis	-	-	+
bacterium_SCN_62-11	-	-	+
bacterium_enrichment_culture_clone_MC3F	-	-	+
Actinoplanes_sp._OR16	-	-	+
Streptococcus_sp._GMD6S	-	-	+
Mycolicibacterium_monacense	-	-	+
Gordonia_sp._CNJ-863	-	-	+
archaeon_BMS3Bbin16	-	-	+
Mycolicibacterium_sp.	-	-	+
Chloroflexi_bacterium_RBG_13_51_36	-	-	+
Sinirhodobacter_sp._SK2B-1	-	-	+
Mycolicibacterium_fortuitum	-	-	+
SAR202_cluster_bacterium_Io17-Chloro-G9	-	-	+
Gillisia_sp._CAL575	-	-	+
Natronohydrobacter_thiooxidans	-	-	+
Chloracidobacterium_thermophilum	-	-	+
Mesorhizobium_sp._LNJC391B00	-	-	+
Rhodococcus_kyotonensis	-	-	+
Methanomassiliicoccales_archaeon_PtaU1.Bin124	-	-	+
Bacillus_sp._CDB3	-	-	+
Meira_miltonrushii	-	-	+
Occidentia_massiliensis	-	-	+
Bifidobacterium_sp._wkB344	-	-	+
Actinobacteria_bacterium_RBG_19FT_COMBO_36_27	-	-	+
Rhodobacteraceae_bacterium_CCMM004	-	-	+

Methanocaldococcus_vulcanius	-	-	+
Bifidobacterium_catulorum	-	-	+
Bacillus_sp._AFS040349	-	-	+
Candidatus_Tokpelaia_hoelldoblerii	-	-	+
Frigoribacterium_sp._MEB024	-	-	+
Hymenobacter_psychrotolerans	-	-	+
Ectothiorhodospira_magna	-	-	+
Methanosphaera_cuniculi	-	-	+
Rhizobium_sp._AC44/96	-	-	+
Simian_retrovirus_4	+	+	+
Paenibacillaceae_bacterium_SCSIO_06110	+	+	+
Paenibacillus_sp._DMB20	+	+	+
Sphaerobacter_thermophilus	+	+	+
Aquimarina_latercula	+	+	+
Caulochytrium_protostelioides	+	+	+
Bacillus_anthracis	-	+	+
Batrachochytrium_dendrobatidis	+	+	+
Candidimonas_sp.	+	+	+
Bacillus_pumilus	+	+	+
uncultured_bacterium_Contig1500	+	+	+
Paenibacillus_yonginensis	+	+	+
Vagococcus_entomophilus	-	+	+
Enterococcus_wangshanyuanii	+	+	+
Staphylococcus_phage_pSco-10	+	+	+
Dictyoglomus_thermophilum	+	+	+
Mortierella_verticillata	+	+	+
Paenibacillaceae_bacterium_GAS479	+	+	+
Conidiobolus_coronatus	+	+	+
Desulfonatronospira_thiodismutans	+	+	+
Wolbachia_endosymbiont_of_Cimex_lectularius	-	+	+
Ruminococcus_sp._AM28-29LB	+	+	+
Acaryochloris_sp._CCMEE_5410	+	+	+
Ruminococcus_sp._AF25-23LB	+	+	+
Atopostipes_suicloacalis	+	+	+
Pectobacterium_wasabiae	+	+	+
Microbacterium_sp._CH12i	-	+	+
Desertifilum_sp._IPPAS_B-1220	+	+	+
Bacillus_fastidiosus	+	+	+
Bacillus_sp._AFS017336	+	+	+
Erysipelothrix_larvae	+	+	+
Candidatus_Komeilibacteria_bacterium_RIFOXYC1_FULL_37_	+	+	+
Marinobacter_sp._es.042	+	+	+
Bacillus_mannanilyticus	+	+	+
Arachidicoccus_sp._KIS59-12	+	+	+
Bacillus_cellulosilyticus	+	+	+
Neisseria_meningitidis	+	+	+
Candidatus_Hydrothermae_bacterium	+	+	+
Candidatus_Nomurabacteria_bacterium_GW2011_GWB1_37_5	+	+	+
Enterococcus_ureasiticus	-	+	+
Olsenella_urininfantis	+	+	+
Odoribacter_sp._AF21-41	+	+	+
Fibrobacter_sp._UWB4	+	+	+
Celeribacter_halophilus	+	+	+
Paenibacillus_rhizosphaerae	+	+	+
anaerobic_bacterium_MO-CFX2	+	+	+

Robiginitalea_sp._O458	+	+	+
Dokdonia_sp._4H-3-7-5	+	+	+
Thermosipho_melanesiensis	-	+	+
bacterium_OL-1	+	+	+
Flavobacteria_bacterium_GWF1_32_7	+	+	+
Shewanella_oneidensis	+	+	+
Candidatus_Dichloromethanomonas_elyunquensis	+	+	+
Photobacterium_phosphoreum	-	+	+
Arcanobacterium_phocae	-	+	+
Candidatus_Altiarchaeales_archaeon_WOR_SM1_SCG	+	+	+
Methanoculleus_sp._SDB	+	+	+
Campylobacter_sp._P0111	+	+	+
Bacteroidetes_bacterium_HGW-Bacteroidetes-18	+	+	+
Rhizoctonia_solani	+	+	+
Bacillus_sp._JCM_19034	+	+	+
Bacillus_sp._V5-8f	-	+	+
Planctomycetes_bacterium_GWA2_39_15	-	+	+
Bacillus_sp._es.036	+	+	+
Coprobacillus_sp._CAG:235_29_27	+	+	+
Pseudomonas_sp._Ant30-3	+	+	+
Limnohabitans_sp._TS-CS-82	+	+	+
Cellulophaga_virus_Cba41	+	+	+
Listeria_riparia	+	+	+
Spirochaetae_bacterium_HGW-Spirochaetae-2	+	+	+
Bacillus_sp._Marseille-P3800	+	+	+
Siphonobacter_aquaeclarae	+	+	+
Sugiyamaella_lignohabitans	+	+	+
Ciceribacter_sp._F8825	-	+	+
Thiovulum_sp._ES	-	+	+
Sinobacterium_caligoides	-	+	+
Nitrospirae_bacterium_RBG_16_43_11	-	+	+
Gynuella_sunshinyii	-	+	+
Bifidobacterium_biavatii	-	+	+
Desulfobacter_vibrioformis	+	+	+
Helicobacter_sp._13S00401-1	+	+	+
uncultured_eukaryote	+	+	+
Massilia_sp._JS1662	+	+	+
Tissierellia_bacterium_KA00581	+	+	+
Propionibacterium_sp.	+	+	+
Geobacteraceae_bacterium	+	+	+
Flavobacterium_gilvum	+	+	+
Mesotoga_sp._BH458_6_3_2_1	-	+	+
Opitutaceae_bacterium_TAV5	-	+	+
Caldithrix_abyssi	-	+	+
candidate_division_NPL-UPA2_bacterium_Unc8	+	+	+
Planifilum_fimeticola	+	+	+
Dickeya_phage_BF25/12	+	+	+
Tenacibaculum_soleae	+	+	+
Leptolyngbya_ohadii	+	+	+
Ktedonobacter_sp._13_2_20CM_53_11	+	+	+
Acaryochloris_marina	+	+	+
Candidatus_Levybacteria_bacterium_GW2011_GWA2_40_8	+	+	+
Ophiocordyceps_australis	+	+	+
Chitinophaga_sp._Mgbs1	+	+	+
Pontibacter_korlensis	+	+	+

Azoarcus_communis	-	+	+
Bacillus_pseudalcaliphilus	-	+	+
Thermoanaerobacter_siderophilus	+	+	+
Burkholderiales_bacterium_CNM695-12	+	+	+
Paenibacillus_sp._FW100M-2	+	+	+
Facklamia_languida	+	+	+
Pyricularia_grisea	+	+	+
Cyanothece_sp._PCC_7425	+	+	+
Cytophagales_bacterium_TFI_002	+	+	+
Chlorobium_ferrooxidans	+	+	+
Paenibacillus_sp._Root444D2	+	+	+
Candidatus_Cloacimonetes_bacterium_4572_65	+	+	+
Lactobacillus_concavus	+	+	+
Calothrix_sp._NIES-2098	-	+	+
Halobacillus_sp._Marseille-P3879	-	+	+
Halobacteriovorax_sp._BALOs_7	-	+	+
Firmicutes_bacterium_HGW-Firmicutes-18	-	+	+
Alkalispirochaeta_alkalica	-	+	+
Leeuwenhoekiella_marinoflava	-	+	+
Pseudomonas_japonica	-	+	+
Bacillus_sp._CX-1	-	+	+
Methylobacter_tundripaludum	-	+	+
Endozoicomonas_acroporae	-	+	+
uncultured_bacterium_Contigcl_1794	-	+	+
Bacteroidetes_bacterium_CG2_30_33_31	-	+	+
Candidatus_Goldbacteria_bacterium_HGW-Goldbacteria-1	+	+	+
Gracilibacillus_halophilus	+	+	+
Bifidobacterium_crudilactis	+	+	+
Anaerobranca_gottschalkii	+	+	+
Alloscardovia_omnicolens	+	+	+
Candidatus_Cloacimonetes_bacterium_4572_55	+	+	+
Atopobium_minutum	+	+	+
Roseiflexus_castenholzii	+	+	+
Olsenella_scatoligenes	+	+	+
Peptoniphilus_ivorii	+	+	+
Sphingobacterium_sp._CZ-UAM	+	+	+
Hyphomonas_beringensis	+	+	+
Chryseobacterium_sp._T16E-39	+	+	+
Schaalia_suimastitidis	+	+	+
Porticoccaceae_bacterium	-	+	+
Phycisphaerae_bacterium_SM-Chi-D1	-	+	+
Streptomyces_sp._CB01201	-	+	+
Brachybacterium_sp._YJGR34	-	+	+
Peptoniphilus_coxii	-	+	+
Candidatus_Staskawiczbacteria_bacterium_RIFCSPHIGHO2_01	-	+	+
Helicobacter_sp._11S03491-1	-	+	+
Crenarchaeota_archaeon	-	+	+
Campylobacter_peloridis	-	+	+
Kiloniella_litopenaei	-	+	+
Granulicatella_elegans	-	+	+
Catenibacterium_sp._CAG:290	-	+	+
Flavobacterium_hercynium	-	+	+
Flavobacterium_sp._ACN6	-	+	+
Opitutaceae_bacterium_EW11	-	+	+
Veillonella_magna	+	+	+

Paenibacillus_taichungensis	+	+	+
Porphyromonadaceae_bacterium_NLAE-zl-C104	+	+	+
Fusobacterium_sp._CAG:649	+	+	+
Fibrobacter_sp._UWT1	+	+	+
Bacillus_ligniniphilus	+	+	+
Moumouvirus_goulette	+	+	+
Ignavibacteria_bacterium	+	+	+
Tenacibaculum_litoreum	+	+	+
Paenibacillaceae_bacterium_ZCTH02-B3	+	+	+
Candidatus_Kryptobacter_tengchongensis	+	+	+
Mesotoga_sp._H07.pep.5.3	+	+	+
Peptoniphilus_rhinitidis	+	+	+
Chloroflexi_bacterium_RBG_16_57_8	+	+	+
Ruminococcus_sp._AM49-8	+	+	+
Rhodopirellula_sp._SM50	-	+	+
Actinobacteria_bacterium_HGW-Actinobacteria-1	-	+	+
Actinoplanes_awajinensis	-	+	+
Candidatus_Kaiserbacteria_bacterium_RIFCSPHIGHO2_01_FU	-	+	+
Pedobacter_sp._Hv1	-	+	+
Nostoc_sphaeroides	-	+	+
Methyloterricola_oryzae	-	+	+
Methylococcaceae_bacterium_TMED69	-	+	+
Micrococcales_bacterium_73-15	-	+	+
Streptomyces_violaceoruber	-	+	+
Bacillus_paralicheniformis	-	+	+
Sphingomonas_wittichii	-	+	+
Mycolicibacterium_moriokaense	-	+	+
Maribacter_sp._Hel_I_7	+	+	+
Cytophagaceae_bacterium_SCN_52-12	+	+	+
Muricauda_olearia	-	+	+
Armatimonadetes_bacterium_RBG_16_58_9	-	+	+
Candidatus_Blackburnbacteria_bacterium_RIFCSPHIGHO2_12	-	+	+
Anaerococcus_sp._Marseille-P2143	-	+	+
Nostoc_calcicola	+	-	+
Candidatus_Gastranaerophilales_bacterium_HUM_6	+	-	+
Streptococcus_sp._DD12	+	-	+
Petrotoga_sp._HKA.pet.4.5	+	-	+
Planctomycetes_bacterium_RIFCSPHIGHO2_02_FULL_40_12	+	-	+
Kluyvera_cryocrescens	+	-	+
Melghirimyces_profundicolus	+	-	+
Ancylomarina_sp._M1P	+	-	+
Sphingobacteriales_bacterium_16-39-50	+	-	+
Candidatus_Levybacteria_bacterium_RIFCSPHIGHO2_01_FUI	+	-	+
Novispirillum_itersonii	+	-	+
Chryseobacterium_sp._ISE14	+	-	+
Anaerobranca_californiensis	+	-	+
Staphylococcus_warneri	+	-	+
Candidatus_Gastranaerophilales_bacterium_HUM_8	+	-	+
Acinetobacter_junii	+	-	+
Ruminococcus_sp._AM36-2AA	+	-	+
Azospirillum_thiophilum	+	-	+
Lactobacillus_rodentium	+	-	+
Tepidibacillus_sp._HK-1	+	-	+
Aggregatibacter_actinomycetemcomitans	+	-	+
Parageobacillus_thermantarcticus	+	-	+

Chryseobacterium_sp._JV274	+	-	+
Weeksella_virosa	+	-	+
Marinospirillum_minutulum	+	-	+
Vagococcus_lutrae	+	-	+
Salegentibacter_sediminis	+	-	+
Bifidobacterium_bohemicum	+	-	+
Bacillus_sp._AFS055030	+	-	+
Cyclobacteriaceae_bacterium_YHN15	+	-	+
Schizosaccharomyces_cryophilus	+	-	+
Xylella_fastidiosa	+	-	+
Thermoactinomyces_vulgaris	+	-	+
Kazachstania_unispora	+	-	+
Flavobacterium_akiainvivens	+	-	+
Sediminibacillus_massiliensis	+	-	+
Geopsychrobacter_electrodiphilus	+	-	+
Saprospira_grandis	+	-	+
Circular_genetic_element_sp.	+	-	+
Terrimonas_sp._NS-102	+	-	+
Rhodanobacter_sp._SCN_66-43	+	-	+
Beggiatoa_alba	+	-	+
Flavobacterium_xinjiangense	+	-	+
Candidatus_Dojkabacteria_bacterium_HGW-Dojkabacteria-1	+	-	+
Bifidobacterium_callitrichidarum	+	-	+
Gammaproteobacteria_bacterium_SG8_11	+	-	+
Caballeronia_ptereochthonis	+	-	+
Ectothiorhodospiraceae_bacterium	+	-	+
Streptococcus_phage_CHPC1029	+	-	+
Tenacibaculum_discolor	+	-	+
Pseudomonadaceae_bacterium_SI-3	+	-	+
Weissella_phage_phiYS61	+	-	+
Bacillus_krulwichiae	+	-	+
Bacillus_marinisedimentorum	+	-	+
candidate_division_WOR-1_bacterium_RIFOXYA12_FULL_43	+	-	+
Aequorivita_viscosa	+	-	+
Micromonospora_sp._CNZ299	+	-	+
Coprobacillus_sp._AF02-13	+	-	+
Caryophanon_tenue	+	-	+
Epulopiscium_sp._SCG-B10WGA-EpuloA2	+	-	+
Paenibacillus_sp._GM1FR	+	-	+
Lysinibacillus_massiliensis	+	-	+
Chlorobi_bacterium_NICIL-2	+	-	+
Staphylococcus_vitulinus	+	-	+
Xylanibacterium_sp._2JSPR-7	+	-	+
Geomicrobium_sp._JCM_19038	+	-	+
Acidipila_sp._4G-K13	+	-	+
uncultured_bacterium_Contig1772	+	-	+
Porphyromonas_bennonis	+	-	+
Firmicutes_bacterium_I2511	+	-	+
Bacillus_sp._AFS053548	+	-	+
Desulfobacteraceae_bacterium_4572_88	+	-	+
Candidatus_Bathyarchaeota_archaeon_B26-2	+	-	+
Rhodopirellula_sp._TMED11	+	-	+
Lasiodiplodia_theobromae	+	-	+
Candidatus_Curtissbacteria_bacterium_RIFCSPLOWO2_01_FU	+	-	+
Enterobacter_sp._NFR05	+	-	+

Prosthecomicrobium_hirschii	+	-	+
Parcubacteria_group_bacterium_GW2011_GWB1_40_14	+	-	+
Lentibacillus_persicus	+	-	+
Coprobacillus_sp._AM26-5AC	+	-	+
Staphylococcus_chromogenes	+	-	+
Zymomonas_mobilis	+	-	+
Coprobacillus_sp._OF02-11LB	+	-	+
Paenibacillus_sp._PAMC_26794	+	-	+
Chloroflexi_bacterium_RBG_16_58_14	+	-	+
Candidatus_Harrisonbacteria_bacterium_CG10_big_fil_rev_8_2	-	-	+
Skermanella_stibiiresistens	-	-	+
Mycoplasma_primatum	-	-	+
Anaerolineaceae_bacterium_4572_5.2	-	-	+
Chloroflexi_bacterium_GWB2_49_20	-	-	+
Salinicoccus_luteus	-	-	+
Ruminococcus_sp._AM45-2	-	-	+
Peptoniphilus_sp._DNF00840	-	-	+
Alphaproteobacteria_bacterium_MarineAlpha9_Bin3	-	-	+
Desulfopila_aestuarii	-	-	+
Candidatus_Methanodesulfokores_washburnensis	-	-	+
Rhodobacteraceae_bacterium	-	-	+
Ardenticatena_maritima	-	-	+
Streptomyces_anulatus	-	-	+
Paenibacillus_sp._453mf	-	-	+
Rufibacter_sp._DG31D	-	-	+
Staphylococcus_epidermidis	-	-	+
Muricauda_pacifica	-	-	+
Anaeromyxobacter_dehalogenans	-	-	+
Flavobacterium_sp._TAB_87	-	-	+
Deltaproteobacteria_bacterium_RIFCSPHIGHO2_12_FULL_43	-	-	+
Candidatus_Zambryskibacteria_bacterium_RIFOXYD1_FULL_	-	-	+
candidate_division_MSBL1_archaeon_SCGC-AAA259J03	-	-	+
Candidatus_Saccharibacteria_bacterium_RIFCSPHIGHO2_01_I	-	-	+
Mesoaciditoga_lauensis	-	-	+
Deltaproteobacteria_bacterium_37-65-8	-	-	+
Salsuginibacillus_kocurii	-	-	+
Vavraia_culicis	-	-	+
uncultured_bacterium_Contig9	-	-	+
Candidatus_Delongbacteria_bacterium_GWF2_40_14	-	-	+
Candidatus_Methanohalarchaeum_thermophilum	-	-	+
Bacillus_halosaccharovorans	-	-	+
Rhodothermaeota_bacterium_MED-G16	-	-	+
Verrucomicrobiales_bacterium_VVV1	-	-	+
Anabaena_sp._WA113	-	-	+
Aneurinibacillus_thermoaerophilus	-	-	+
Hymenobacter_sp._PAMC_26628	-	-	+
Deltaproteobacteria_bacterium_CG2_30_43_15	-	-	+
Sphingomonas_sp._HMF7854	-	-	+
candidate_division_SR1_bacterium	-	-	+
bacterium_(Candidatus_Gribaldobacteria)_CG23_combo_of_CC	-	-	+
Thermococcus_kodakarensis	-	-	+
Armatimonadetes_bacterium_RBG_16_67_12	-	-	+
Bacillus_phage_Moonbeam	-	-	+
Solemya_velesiana_gill_symbiont	-	-	+
Planctomycetes_bacterium_GWA2_40_7	-	-	+

miscellaneous_Crenarchaeota_group_archaeon_SMTZ-80	-	-	+
Psychrobacter_sp._PAMC_21119	-	-	+
Psychromonas_hadalis	-	-	+
Campylobacter_gracilis	-	-	+
Agrobacterium_sp._RAC06	-	-	+
Acetobacter_tropicalis	-	-	+
Rhizobiales_bacterium_35-66-30	-	-	+
Caldivirga_maquilingensis	-	-	+
Archaeoglobus_veneficus	-	-	+
Polaribacter_sp._SA4-12	-	-	+
Algoriphagus_resistens	-	-	+
Streptomyces_yeochonensis	-	-	+
Brevundimonas_diminuta	-	-	+
Pseudomonas_linyingensis	-	-	+
Brevibacillus_sp._SKDU10	-	-	+
Terriglobus_sp._TAA_43	-	-	+
Acidovorax_anthurii	-	-	+
Bacillus_sp._SRB_336	-	-	+
Pseudomonas_guineae	-	-	+
Pseudomonas_sp._BBP2017	-	-	+
Bacillus_sp._HNG	-	-	+
Pseudomonas_sp._QZS01	-	-	+
Tabrizicola_sp._K13M18	-	-	+
Bdellovibrionales_bacterium_CG12_big_fil_rev_8_21_14_0_65	-	-	+
Vitellibacter_aquimaris	-	-	+
Amycolatopsis_niigatensis	-	-	+
Pseudomonas_syringae_pv._coryli	-	-	+
Actinoplanes_sp._N902-109	-	-	+
Bacillus_sp._JCM_19045	-	-	+
Xanthomonas_sp._MUS_060	-	-	+
Pseudoroseicyclus_aestuarii	-	-	+
Pseudarthrobacter_siccitolerans	-	-	+
Sulfurovum_riftiae	-	-	+
Pseudoalteromonas_sp._A757	-	-	+
Prosthecochloris_aestuarii	-	-	+
Arthrobacter_sp._Soil761	-	-	+
Xenorhabdus_eapokensis	-	-	+
Bacteroidetes_bacterium_GWD2_40_43	-	-	+
bacterium_HR33	-	-	+
Anaerolineae_bacterium_CG1_02_58_13	-	-	+
Pseudoalteromonas_undina	-	-	+
Bacillus_sp._WP8	-	-	+
Bacillus_phage_Mater	-	-	+
Oenococcus_sp._UCMA_16435	-	-	+
Lachancea_nothofagi	-	-	+
Labedella_sp._8H24J-4-2	-	-	+
Candidatus_Shapirobacteria_bacterium_CG08_land_8_20_14_0	-	-	+
Candidatus_Saccharibacteria_bacterium_RIFCSPHIGHO2_12_I	-	-	+
Ketogulonicigenium_vulgare	-	-	+
Candidatus_Roizmanbacteria_bacterium_CG_4_10_14_0_2_um	-	-	+
Dehalogenimonas_alkenigignens	-	-	+
Candidatus_Woykebacteria_bacterium_RIFCSPHIGHO2_01_FI	-	-	+
Collinsella_sp._TM09-10AT	-	-	+
Coprobacillus_sp._AF27-24BH	-	-	+
Lactobacillus_phage_LJ	-	-	+

Candidatus_Thorarchaeota_archaeon_AB_25	-	-	+
Jiangella_alkaliphila	-	-	+
Hoeflea_sp._108	-	-	+
Hoeflea_sp.	-	-	+
Helcobacillus_massiliensis	-	-	+
Candidatus_Magasanikbacteria_bacterium_RIFCSPLOWO2_02	-	-	+
Candidatus_Lokiarchaeota_archaeon_CR_4	-	-	+
Halobacteriales_archaeon_QS_9_70_65	-	-	+
Candidatus_Kerfeldbacteria_bacterium_RIFOXYB2_FULL_38_	-	-	+
Halobacillus_sp._KGW1	-	-	+
Jeotgalicoccus_marinus	-	-	+
Deltaproteobacteria_bacterium_GWA2_54_12	-	-	+
Deltaproteobacteria_bacterium_HGW-Deltaproteobacteria-17	-	-	+
Candidatus_Pacebacteria_bacterium_CG10_big_fil_rev_8_21_1	-	-	+
Ignavibacteria_bacterium_RBG_16_35_7	-	-	+
Candidatus_Nomurabacteria_bacterium_GW2011_GWD2_39_1	-	-	+
Candidatus_Moranbacteria_bacterium_GW2011_GWF1_36_78	-	-	+
Hymenobacter_sp._CCM_8763	-	-	+
Nostoc_piscinale	-	-	+
Microbacteriaceae_bacterium_BACL28_MAG-120531-bin53	-	-	+
Myxococcales_bacterium	-	-	+
Chloroflexi_bacterium_HGW-Chloroflexi-9	-	-	+
Methanoculleus_taiwanensis	-	-	+
Negativicoccus_massiliensis	-	-	+
Microbacterium_sp._MRS-1	-	-	+
Mycobacterium_sp.	-	-	+
Micromonospora_aurantiaca	-	-	+
Chitinophaga_sp._ZY74	-	-	+
Loktanella_sp._5RATIMAR09	-	-	+
Clostridium_sp._IBUN22A	-	-	+
Nitrospirae_bacterium_CG2_30_53_67	-	-	+
Legionella_sp._km714	-	-	+
Nocardia_amamiensis	-	-	+
Nonlabens_marinus	-	-	+
Marinobacter_similis	-	-	+
Marichromatium_purpuratum	-	-	+
Maliponia_aquimaris	-	-	+
Candidatus_Doudnabacteria_bacterium_RIFCSPHIGHO2_02_F	-	-	+
Frankia_sp._R43	-	-	+
Pedobacter_sp._KBW01	-	-	+
Flavobacterium_urocaniciphilum	-	-	+
Parcubacteria_group_bacterium_GW2011_GWA2_40_37	-	-	+
Parcubacteria_group_bacterium_GW2011_GWC1_43_12	-	-	+
Geobacillus_sp._LEMMY01	-	-	+
Duck_infectious_anemia_virus	-	-	+
Duganella_sp._BK054	-	-	+
Flagellimonas_flava	-	-	+
Exiguobacterium_sp._S17	-	-	+
Campylobacter_sp._P0227	-	-	+
Flammeovirga_sp._OC4	-	-	+
Candidatus_Buchananbacteria_bacterium_RIFCSPHIGHO2_02_	-	-	+
Flavobacterium_sp._38-13	-	-	+
Enterococcus_canis	-	-	+
Flavobacterium_magnum	-	-	+
Candidatus_Atelocyanobacterium_thalassa	-	-	+

Candidatus_Arthromitus_sp._SFB-co	-	-	+
Petrotoga_sp._9T1HF07.CasAA.8.2	-	-	+
Candidatus_Gottesmanbacteria_bacterium_GW2011_GWA2_43	-	-	+
Halanaerobium_sp._DL-01	-	-	+
Haemophilus_parainfluenzae	-	-	+
Halanaerobium_sp.	-	-	+
Leptotrichia_shahii	+	+	+
Endogone_sp._FLAS-F59071	+	+	+
Streptococcus_milleri	+	+	+
Klebsiella_quasipneumoniae	+	+	+
Nitrosarchaeum_koreense	+	+	+
Lactobacillus_intestinalis	+	+	+
Candidatus_Wallbacteria_bacterium_HGW-Wallbacteria-1	-	+	+
Sulfurihydrogenibium_subterraneum	+	+	+
Thermodesulfovibrio_thiophilus	+	+	+
Firmicutes_bacterium_GWE2_51_13	+	+	+
Bacteroidetes_bacterium_HGW-Bacteroidetes-4	+	+	+
Salisediminibacterium_halotolerans	-	+	+
Candidatus_Gastranaerophilales_bacterium_HUM_13	+	+	+
Clostridium_sp._CAG:729	+	+	+
Staphylococcus_fleurettii	+	+	+
Paenibacillus_sp._oral_taxon_786	+	+	+
Bacteroidetes_bacterium_CG23_combo_of_CG06-09_8_20_14_	+	+	+
Candidatus_Gastranaerophilales_bacterium_HUM_20	+	+	+
Oceanotoga_teriensis	+	+	+
Prosthecobacter_debontii	+	+	+
Flavobacterium_sp._Fl	+	+	+
Aerococcaceae_bacterium_ZY16052	-	+	+
Mycoplasma_pullorum	-	+	+
Candidatus_Aquiluna_sp._XM-24bin5	-	+	+
Magnetospirillum_caucaseum	+	+	+
Helicobacter_sp._11S02629-2	+	+	+
Chloroflexi_bacterium_54-19	+	+	+
Bdellovibrionales_bacterium_GWB1_52_6	-	+	+
Parvimonas_sp._oral_taxon_110	+	+	+
Vibrio_sp._2017V-1124	+	+	+
Veillonella_sp._ACP1	+	+	+
Sphingopyxis_fribergensis	-	+	+
Collinsella_sp._AM13-34	-	+	+
Paenibacillus_sp._VTT_E-133280	+	+	+
Belliella_buryatensis	+	+	+
Pseudomonas_sp._ATCC_13867	+	+	+
Streptococcus_sp._F0442	+	+	+
Cohnella_sp.	+	+	+
Kurthia_sp._11kri321	+	+	+
Actinomyces_hominis	-	+	+
Geobacillus_jurassicus	-	+	+
Clostridium_sp._OM05-9BH	+	+	+
Clostridium_sp._enrichment_culture_clone_7-14	+	+	+
Candidatus_Kaiserbacteria_bacterium_GWA2_50_9	+	+	+
Corynebacterium_heidelbergense	+	+	+
Salinispora_arenicola	+	+	+
Lysinibacillus_endophyticus	+	+	+
Salibacterium_qingdaonense	-	+	+
Bacillus_altitudinis	-	+	+

Thermococcus_celericrescens	-	+	+
Spirochaetes_bacterium_RIFOXYB1_FULL_32_8	+	+	+
Kordia_jejudonensis	+	+	+
Megasphaera_sp._UPII_199-6	+	+	+
Roseiflexus_sp._RS-1	+	+	+
Candidatus_Carbobacillus_altaicus	+	+	+
Roseburia_sp._AF25-25LB	+	+	+
Granulicella_sp._GAS466	+	+	+
Candidatus_Entotheonella_serta	+	+	+
Geobacter_sp._S43	-	+	+
Candidatus_Thiodictyon_syntrophicum	-	+	+
Flavobacterium_sp._TCH3-2	+	+	+
Lysinibacillus_chungkukjangi	+	+	+
Candidatus_Firestonebacteria_bacterium_RIFOXYD2_FULL_3[illegible]	+	+	+
Pontibacter_ummariensis	+	+	+
Campylobacter_sp._P0209	+	+	+
Serratia_fonticola	+	+	+
Oceanobacillus_iheyensis	-	+	+
Bacteroidetes_bacterium_4484_249	-	+	+
Lepidopterella_palustris	-	+	+
Kingella_denitrificans	-	+	+
Elephant_endotheliotropic_herpesvirus_4	-	+	+
Actinomyces_vulturis	-	+	+
Phycisphaerae_bacterium_ST-NAGAB-D1	-	+	+
Lysinibacillus_sp._YLB-03	+	+	+
Listeria_weihenstephanensis	+	+	+
Actinomycetaceae_bacterium_Marseille-P6182	+	+	+
Streptococcus_henryi	+	+	+
Firmicutes_bacterium_AF19-2LB	+	+	+
Varibaculum_cambriense	+	+	+
Pasteurella_testudinis	+	+	+
Flavobacterium_sp._KMS	+	+	+
Veillonella_ratti	+	+	+
Crocinitomix_sp._SM1701	+	+	+
Mycolicibacterium_holsaticum	+	+	+
Microvirga_lotononidis	-	+	+
Alkalibacterium_sp._AK22	-	+	+
Bifidobacterium_felsineum	-	+	+
Nitratireductor_indicus	-	+	+
Dickeya_sp._2B12	-	+	+
Marinifilaceae_bacterium_T3-2_S1-C	-	+	+
Cellulosimicrobium_sp._CUA-896	-	+	+
Bacillus_halmapalus	-	+	+
Fervidicoccus_fontis	-	+	+
Mycoplasma_sp._CAG:611_25_7	+	+	+
Olsenella_sp._DNF00959	+	+	+
Clostridium_sp._Bc-iso-3	+	+	+
gamma_proteobacterium_IMCC1989	+	+	+
Methanolobus_profundi	+	+	+
uncultured_bacterium_Contig2	+	+	+
Desulfonauticus_submarinus	-	+	+
Lentinula_edodes	-	+	+
Wohlfahrtiimonas_sp._G9077	-	+	+
Alkalibacterium_pelagium	-	+	+
Pandoraea_sp._PE-S2T-3	-	+	+

Spirochaetes_bacterium_RBG_16_49_21	-	+	+
Bacteroidetes_bacterium_CG02_land_8_20_14_3_00_31_25	-	+	+
Planomicrobium_flavidum	+	+	+
Ruminococcus_sp._AF13-28	+	+	+
Calothrix_sp._PCC_7507	+	+	+
Vibrio_diazotrophicus	+	+	+
Bacillus_safensis	+	+	+
Bizionia_argentinensis	+	+	+
Carboxydocella_sp._ULO1	+	+	+
Raineyella_antarctica	+	+	+
Rhodobacteraceae_bacterium_Alg231-30	+	+	+
Weissella_koreensis	+	+	+
Vulcanibacillus_modesticaldus	+	+	+
Absidia_glauca	+	+	+
Pseudomonas_psychrotolerans	+	+	+
Candidatus_Syntrophoarchaeum_caldarius	+	+	+
Siphonobacter_curvatus	-	+	+
uncultured_Parabacteroides_sp.	-	+	+
Thioalkalivibrio_paradoxus	-	+	+
Yersinia_frederiksenii	-	+	+
Ketobacter_sp.	-	+	+
Flavobacterium_sp._BFFFF1	-	+	+
Pseudonocardiales_bacterium	-	+	+
Microbacterium_mangrovi	-	+	+
Agrobacterium_deltaense	-	+	+
Xanthobacter_tagetidis	-	+	+
Actinomyces_culturomici	-	+	+
Meiothermus_cerbereus	-	+	+
Leifsonia_sp._Leaf336	-	+	+
Fibrobacter_sp._NR9	-	+	+
Alteromonadales_bacterium_BS08	-	+	+
Polaribacter_reichenbachii	+	+	+
Pseudomonas_poae	+	+	+
Tupanvirus_deep_ocean	+	+	+
Pilibacter_termitis	+	+	+
Pedobacter_alluvionis	+	+	+
Phormidesmis_priestleyi	+	+	+
Burkholderia_territorii	+	+	+
Pusillimonas_noertemannii	+	+	+
Chloroflexi_bacterium_HGW-Chloroflexi-10	+	+	+
Ammoniphilus_sp._CFH_90114	+	+	+
Sphingobacterium_nematocida	+	+	+
Azospirillum_sp._L-25-5w-1	+	+	+
Roseomonas_gilardii	+	+	+
Chitinivibrio_alkaliphilus	+	+	+
Acidovorax_sp._35-64-16	+	+	+
bacterium_BMS3Abin04	+	+	+
Bacteroidetes_bacterium_HGW-Bacteroidetes-17	+	+	+
Rivibacter_subsaxonicus	+	+	+
Bacillus_smithii	+	+	+
Paenibacillus_uliginis	+	+	+
Salibacterium_halotolerans	+	+	+
Vibrio_maritimus	+	+	+
Desulfofustis_glycolicus	-	+	+
Gallibacterium_genomosp._1	-	+	+

Bacteroides_sp._AM28-6	-	+	+
Rubrivivax_gelatinosus	-	+	+
Xanthomonas_sp._DAR33341	-	+	+
Pseudomonas_chlororaphis	-	+	+
Plesiomonas_shigelloides	-	+	+
Vibrio_mimicus	-	+	+
Paracoccus_yeei	-	+	+
Actinomyces_urogenitalis	-	+	+
Blastococcus_sp._TF02-8	-	+	+
Flavobacteriales_bacterium_ALC-1	-	+	+
Bacillus_bingmayongensis	-	+	+
Alteromonas_naphthalenivorans	-	+	+
Merismopedia_glauca	-	+	+
Streptomyces_sp._BK022	-	+	+
Desulfobacteraceae_bacterium_4572_187	-	+	+
Aquitalea_magnusonii	-	+	+
Roseburia_sp._OM04-10AA	+	+	+
Taibaiella_sp._F-4	+	+	+
Calothrix_rhizosoleniae	+	+	+
Candidatus_Yanofskybacteria_bacterium_GW2011_GWA2_44_	+	+	+
Calothrix_parietina	+	+	+
Porphyromonadaceae_bacterium_CG2_30_38_12	+	+	+
Pararhizobium_haloflavum	+	+	+
Coprobacillus_sp._AF36-10BH	+	+	+
Rufibacter_roseus	+	+	+
Allisonella_histaminiformans	+	+	+
Olsenella_sp._oral_taxon_809	+	+	+
Parabacteroides_sp._AM18-12LB	+	+	+
Parabacteroides_sp._D25	+	+	+
Coriobacterium_glomerans	+	+	+
Algoriphagus_sp._XAY3209	+	+	+
Anaerobacillus_arseniciselenatis	+	+	+
Candidatus_Uhrbacteria_bacterium_CG10_big_fil_rev_8_21_14	+	+	+
Piptocephalis_cylindrospora	-	+	+
Candidatus_Handelsmanbacteria_bacterium_RIFCSPLOWO2_1	-	+	+
Salinicoccus_roseus	-	+	+
Ardenticatenia_bacterium	-	+	+
Aquifex_sp.	-	+	+
Hydrotalea_sandarakina	-	+	+
Geoalkalibacter_subterraneus	-	+	+
Bacillus_sp._FJAT-18017	-	+	+
Pseudomonas_yangmingensis	-	+	+
Paenibacillus_bovis	-	+	+
Achromobacter_sp._AONIH1	-	+	+
Neisseria_canis	-	+	+
Halobacillus_alkaliphilus	-	+	+
Sporosarcina_sp._P33	+	+	+
Lentisphaerae_bacterium_GWF2_45_14	-	+	+
Candidatus_Hydrogenedentes_bacterium	-	+	+
Bifidobacterium_boum	-	+	+
Psychromonas_aquimarina	-	+	+
Candidatus_Kentron_sp._UNK	-	+	+
Elusimicrobia_bacterium_GWA2_66_18	-	+	+
Flavobacterium_lacus	-	+	+
Akkermansia_sp._54_46	+	-	+

Sphingomonas_sp._URHD0057	+	-	+
Atopobium_rimae	+	-	+
Parcubacteria_bacterium_DG_74_1	+	-	+
Pisciglobus_halotolerans	+	-	+
Actinobacteria_bacterium_66_15	+	-	+
uncultured_bacterium_(gcode_4)	+	-	+
[Bacillus]_selenitireducens	+	-	+
Domibacillus_iocasae	+	-	+
Chryseobacterium_sp._G0186	+	-	+
Methanobrevibacter_arboriphilus	+	-	+
Caminibacter_mediatlanticus	+	-	+
Devosia_epidermidihirudinis	+	-	+
Olsenella_sp._Marseille-P4518	+	-	+
Parcubacteria_group_bacterium_GW2011_GWB1_43_6	+	-	+
Chlamydia_suis	+	-	+
Mariprofundus_ferrooxydans	+	-	+
Gallibacterium_salpingitidis	+	-	+
Flavobacterium_cutihirudinis	+	-	+
Bradyrhizobium_oligotrophicum	+	-	+
Cyanobacteria_bacterium_M5B4	+	-	+
Psychroserpens_jangbogonensis	+	-	+
Myxococcus_virescens	+	-	+
Alphaproteobacteria_bacterium_RIFCSPHIGHO2_02_FULL_4(	+	-	+
Fibrobacter_sp._UWH1	+	-	+
Streptobacillus_felis	+	-	+
Leeuwenhoekiella_blandensis	+	-	+
Aphanizomenon_flos-aquae	+	-	+
Bifidobacterium_vansinderenii	+	-	+
Vagococcus_fluvialis	+	-	+
Alphaproteobacteria_bacterium_MarineAlpha9_Bin4	+	-	+
Bacillus_testis	+	-	+
Gammaproteobacteria_bacterium_HGW-Gammaproteobacteria-[illegible]	+	-	+
Micromonospora_sp._HM5-17	+	-	+
Lactobacillus_ruminis_CAG:367	+	-	+
Leptotrichia_sp._oral_taxon_879	+	-	+
Fructobacillus_fructosus	+	-	+
Staphylococcus_pasteuri	+	-	+
Candida_tropicalis	+	-	+
Citrobacter_europaeus	+	-	+
Calditerrivibrio_nitroreducens	+	-	+
Lactobacillus_insicii	+	-	+
Propionibacterium_acidifaciens	+	-	+
Salmonella_phage_Melville	+	-	+
Chryseobacterium_sp._ERMR1:04	+	-	+
Janthinobacterium_lividum	+	-	+
Mucilaginibacter_sp._OV119	+	-	+
Algoriphagus_yeomjeoni	+	-	+
Acidipila_sp._EB88	+	-	+
Microbacterium_agarici	+	-	+
Streptococcus_ferus	+	-	+
Chitinophaga_pinensis	+	-	+
Collinsella_sp._TF09-1AT	+	-	+
Desulfovibrio_sp._DV	+	-	+
Lactobacillus_melliventris	+	-	+
candidate_division_Zixibacteria_bacterium_RBG_16_43_9	+	-	+

candidate_division_WOR-3_bacterium	+	-	+
Planococcus_maritimus	+	-	+
uncultured_bacterium_r_09	+	-	+
Wenyingzhuangia_marina	+	-	+
Rhodonellum_ikkaensis	+	-	+
Candidatus_Woesebacteria_bacterium_RIFCSPHIGHO2_01_FU	+	-	+
Lactococcus_phage_949	+	-	+
Acidipropionibacterium_acidipropionici	+	-	+
Streptomyces_xanthophaeus	+	-	+
Syntrophaceae_bacterium_CG2_30_58_14	+	-	+
Flavobacterium_enshiense	+	-	+
Paenibacillus_sp._M-152	+	-	+
Viridibacillus_arenosi	+	-	+
Psychrobacillus_psychrodurans	+	-	+
Tenacibaculum_finnmarkense	+	-	+
Weissella_oryzae	+	-	+
Pelobacter_propionicus	+	-	+
Hadesarchaea_archaeon	+	-	+
Kazachstania_sinensis	+	-	+
Francisella_philomiragia	+	-	+
Catellicoccus_marimammalium	+	-	+
Dyadobacter_sp._RS19	+	-	+
Capnocytophaga_sp._ChDC_OS43	+	-	+
Nosema_ceranae	+	-	+
Roseomonas_sp._CQN31	+	-	+
Helicobacter_marmotae	+	-	+
Thermoactinomyces_sp._DSM_45891	+	-	+
Streptomyces_toyocaensis	+	-	+
Alysiella_filiformis	+	-	+
Methanosarcina_vacuolata	+	-	+
Human_gut_gokushovirus	+	-	+
Lactobacillus_algidus	+	-	+
Muricauda_zhangzhouensis	+	-	+
Haloactinobacterium_album	+	-	+
Bacillus_muralis	+	-	+
Valsa_mali	+	-	+
Methanosarcina_horonobensis	+	-	+
Kaistia_soli	+	-	+
Planctomyces_sp._SH-PL14	+	-	+
Lactobacillus_sp._8-1(1)	+	-	+
Bradyrhizobium_japonicum	+	-	+
Paludifilum_halophilum	+	-	+
Flavobacterium_fontis	+	-	+
Aerococcus_sp._HMSC10H05	+	-	+
Streptococcus_orisasini	+	-	+
Chryseobacterium_balustinum	+	-	+
Dickeya_dianthicola	+	-	+
Rhizobium_sp._42MFCr.1	+	-	+
Kurthia_zopfii	+	-	+
Streptosporangium_roseum	+	-	+
Rhodovulum_sp._MB263	+	-	+
Crocinitomix_algicola	+	-	+
Candidimonas_nitroreducens	+	-	+
Granulibacter_bethesdensis	+	-	+
Alicyclobacillus_montanus	-	-	+

Rhodobacter_sp._24-YEA-8	-	-	+
Candidatus_Falkowbacteria_bacterium_RIFOXYC2_FULL_34_	-	-	+
Bordetella_pertussis	-	-	+
Candidatus_Bathyarchaeota_archaeon_CG07_land_8_20_14_0_	-	-	+
Nitrosomonas_eutropha	-	-	+
candidate_division_CPR3_bacterium_GW2011_GWF2_35_18	-	-	+
Wickerhamomyces_anomalus	-	-	+
Raoultella_ornithinolytica	-	-	+
Brenneria_sp._CFCC_11842	-	-	+
Marininema_halotolerans	-	-	+
Synechococcus_sp._JA-2-3B'a(2-13)	-	-	+
Pandoraea_apista	-	-	+
Polaribacter_sp._Hel1_33_78	-	-	+
Candidatus_Abyssubacteria_bacterium_SURF_5	-	-	+
Desulfobacterales_bacterium	-	-	+
Methanocella_arvoryzae	-	-	+
Sphingobacteriales_bacterium_TSM_CSM	-	-	+
Hymenobacter_mucosus	-	-	+
candidate_division_TM6_bacterium_GW2011_GWE2_42_60	-	-	+
Nitrincola_tibetensis	-	-	+
Colletotrichum_tofieldiae	-	-	+
Altererythrobacter_dongtanensis	-	-	+
bacterium_A52C2	-	-	+
Rhizobiales_bacterium_62-17	-	-	+
Ornithinibacillus_contaminans	-	-	+
uncultured_bacterium_Contigcl_30	-	-	+
Bacteroidetes_bacterium_RBG_13_44_24	-	-	+
Actinomycetaceae_bacterium_sk1b4	-	-	+
Wenzhouxiangella_marina	-	-	+
Coprinopsis_cinerea	-	-	+
Microvirga_sp._BSC39	-	-	+
Vibrio_ostreicida	-	-	+
Clostridium_sp._HMSC19D07	-	-	+
Klebsiella_virus_0507KN21	-	-	+
Sphingobacterium_sp._CFCC_11742	-	-	+
Armatimonadetes_bacterium_DC	-	-	+
Fervidobacterium_islandicum	-	-	+
Opitutae_bacterium_Tous-C1TDCM	-	-	+
Candidatus_Daviesbacteria_bacterium_RIFCSPLOWO2_02_FU	-	-	+
Cytophaga_aurantiaca	-	-	+
Molluscum_contagiosum_virus	-	-	+
Frigoribacterium_sp._PhB118	-	-	+
Virgibacillus_necropolis	-	-	+
Maritalea_myrionectae	-	-	+
Megasphaera_genomosp._type_2	-	-	+
Halomonas_sp._HAL1	-	-	+
Streptomyces_sp._NRRL_F-6131	-	-	+
Streptomyces_sp._NRRL_S-350	-	-	+
Streptomyces_sp._S10(2018)	-	-	+
Streptomyces_noursei	-	-	+
Halomonas_sp._A11-A	-	-	+
Enterococcus_sp._5B3_DIV0040	-	-	+
Streptomyces_phage_Gibson	-	-	+
Streptomyces_rhizosphaericus	-	-	+
Streptomyces_roseochromogenus	-	-	+

Streptomyces_sp._844.5	-	-	+
Clostridium_sp._HMSC19E03	-	-	+
Synechococcus_sp._KORDI-100	-	-	+
Leucobacter_celer	-	-	+
Tenacibaculum_sp._4G03	-	-	+
Gryllotalpicola_sp._2DFW10M-5	-	-	+
Marinithermus_hydrothermalis	-	-	+
Maribacter_antarcticus	-	-	+
Xanthomonas_arboricola	-	-	+
Halomonas_utahensis	-	-	+
Magnoliophyta_environmental_sample	-	-	+
uncultured_Acetothermia_bacterium	-	-	+
Methanocorpusculum_sp._MCE	-	-	+
Flavimarina_sp._Hel_I_48	-	-	+
Sporosarcina_sp._P17b	-	-	+
Micromonospora_echinospora	-	-	+
Erwinia_toletana	-	-	+
Microgenomates_group_bacterium_GW2011_GWB1_45_17	-	-	+
Staphylococcus_phage_Quidividi	-	-	+
Bacillus_sp._Soil531	-	-	+
Muricauda_taeanensis	-	-	+
Sphingosinicella_sp._BN140058	-	-	+
Sphingosinicella_vermicomposti	-	-	+
Mucilaginibacter_sp._YR332	-	-	+
Exiguobacterium_sp._KRL4	-	-	+
Spiroplasma_litorale	-	-	+
Citrobacter_sp._S-77	-	-	+
Bacillus_virus_Camphawk	-	-	+
Bacillus_virus_Bcp1	-	-	+
Citromicrobium_sp._WPS32	-	-	+
Zetaproteobacteria_bacterium_CG1_02_55_237	-	-	+
Exiguobacterium_sp._ZOR0005	-	-	+
Bacillus_sp._OV186	-	-	+
Methylobacterium_currus	-	-	+
Bacillus_sp._FJAT-25496	-	-	+
Microbulbifer_mangrovi	-	-	+
Epulopiscium_sp._SCG-B10WGA-EpuloB	-	-	+
Bacillus_sp._H1a	-	-	+
Desulfobacterales_bacterium_S7086C20	-	-	+
Anderseniella_sp._Alg231-50	-	-	+
Kitasatospora_phosalacinea	-	-	+
Kiloniella_spongiae	-	-	+
Gemmobacter_megaterium	-	-	+
Vagococcus_penaei	-	-	+
Dehalococcoidia_bacterium_CG2_30_46_9	-	-	+
Variovorax_paradoxus	-	-	+
Jiangella_sp._DSM_45060	-	-	+
bacterium_HR19	-	-	+
Deinococcus_puniceus	-	-	+
Torulaspora_delbrueckii	-	-	+
Gammaproteobacteria_bacterium_SG8_15	-	-	+
Thiobacillus_sp._65-29	-	-	+
Devriesea_agamarum	-	-	+
Apiotrichum_scarabaeorum	-	-	+
Thiomonas_intermedia	-	-	+

bacterium_HR36	-	-	+
Kordia_algicida	-	-	+
Deinococcus_sp._NW-56	-	-	+
Veillonella_sp._3_1_44	-	-	+
Deltaproteobacteria_bacterium_RIFOXYC2_FULL_48_10	-	-	+
Amycolatopsis_sp._WAC_01375	-	-	+
Ignavibacteria_bacterium_RIFOXYB2_FULL_37_11	-	-	+
Agarivorans_albus	-	-	+
Desulfobacteraceae_bacterium_4484_190.1	-	-	+
Vibrio_navarrensis	-	-	+
bacterium_G20	-	-	+
Geodermatophilus_africanus	-	-	+
Dichelobacter_nodosus	-	-	+
Deltaproteobacteria_bacterium_GWC2_65_14	-	-	+
Deltaproteobacteria_bacterium_RBG_13_52_11b	-	-	+
Aeromonas_sobria	-	-	+
Legionella_shakespearei	-	-	+
Actinobacteria_bacterium_69-20	-	-	+
Thauera_phenolivorans	-	-	+
Thermicanus_aegyptius	-	-	+
Atopobacter_phocae	-	-	+
candidate_division_TM6_bacterium_GW2011_GWE2_31_21	-	-	+
Herbidospora_mongoliensis	-	-	+
Elusimicrobia_bacterium_GWB2_63_22	-	-	+
Vibrio_sp._ZOR0018	-	-	+
candidate_division_WS6_bacterium_OLB20	-	-	+
Cohnella_sp._CIP_111063	-	-	+
Tetrapisispora_blattae	-	-	+
candidate_division_WS6_bacterium_GW2011_GWC1_36_11	-	-	+
Collimonas_pratensis	-	-	+
Acinetobacter_sp._NIPH_899	-	-	+
Collinsella_sp._AF11-11	-	-	+
Collinsella_sp._AF15-51	-	-	+
[Brevibacterium]_frigoritolerans	-	-	+
Lactobacillus_curvatus	-	-	+
Arcobacter_lanthieri	-	-	+
Thermoplasmatales_archaeon_ex4484_30	-	-	+
Corallococcus_sp._AB047A	-	-	+
Desulfofundulus_australicus	-	-	+
Arcanobacterium_urinimassiliense	-	-	+
Corynebacterium_sp._HMSC067D03	-	-	+
Gammaproteobacteria_bacterium_MFB021	-	-	+
Thioalkalivibrio_sp._AKL8	-	-	+
Arcticibacter_eurypsychrophilus	-	-	+
Thermofilum_adornatus	-	-	+
candidate_division_NC10_bacterium_CSP1-5	-	-	+
Hoyosella_altamirensis	-	-	+
Thermococcus_sibiricus	-	-	+
Lactobacillus_secaliphilus	-	-	+
Alphaproteobacteria_bacterium_65-37	-	-	+
Lactobacillus_oris	-	-	+
Ectothiorhodospira_sp._BSL-9	-	-	+
Streptomyces_sp._CNT372	-	-	+
Opitutus_sp._ER46	-	-	+
Robbsia_sp._DHC34	-	-	+

Parcubacteria_group_bacterium_CG10_big_fil_rev_8_21_14_0_	-	-	+
Parcubacteria_group_bacterium_GW2011_GWA1_47_11	-	-	+
Parcubacteria_group_bacterium_GW2011_GWA2_37_10	-	-	+
Burkholderia_stagnalis	-	-	+
Candidatus_Zambryskibacteria_bacterium_CG11_big_fil_rev_8_	-	-	+
Candidatus_Kaiserbacteria_bacterium_RIFOXYB1_FULL_46_1	-	-	+
Brevibacterium_jeotgali	-	-	+
Nocardia_vaccinii	-	-	+
Bacteroidetes_bacterium_SCGC_AAA795-G10	-	-	+
Pseudomonas_extremaustralis	-	-	+
Parcubacteria_group_bacterium_GW2011_GWA2_40_14	-	-	+
Pedobacter_lusitanus	-	-	+
Niastella_sp._SCN_39-18	-	-	+
Pediococcus_stilesii	-	-	+
Propionibacterium_sp._JV5	-	-	+
Chitinophaga_sp._S165	-	-	+
Pseudogulbenkiania_sp._NH8B	-	-	+
Burkholderiales_bacterium_PBB2	-	-	+
Rothia_nasimurium	-	-	+
Buttiauxella_izardii	-	-	+
Candidatus_Falkowbacteria_bacterium_RIFCSPLOWO2_12_FU	-	-	+
Patulibacter_minatonensis	-	-	+
Olsenella_sp._AM05-7	-	-	+
Bifidobacterium_sp._wkB338	-	-	+
Candidatus_Pacebacteria_bacterium_CG10_big_fil_rev_8_21_1	-	-	+
Candidatus_Spechtbacteria_bacterium_RIFCSPHIGHO2_01_FU	-	-	+
Candidatus_Moranbacteria_bacterium_GW2011_GWC2_45_10	-	-	+
Oxalobacteraceae_bacterium_IMCC9480	-	-	+
Candidatus_Propionivibrio_aalborgensis	-	-	+
Oscillatoriales_cyanobacterium_USR001	-	-	+
Rabbit_fibroma_virus	-	-	+
Paenibacillus_phage_BN12	-	-	+
Candidatus_Rokubacteria_bacterium_13_1_40CM_2_68_8	-	-	+
Pyrococcus_furiosus	-	-	+
Candidatus_Peregrinibacteria_bacterium	-	-	+
Paenibacillus_sp._3-5-3	-	-	+
Candidatus_Pacebacteria_bacterium_RIFOXYC1_FULL_39_21	-	-	+
Psychroflexus_sediminis	-	-	+
Candidatus_Pacebacteria_bacterium_CG2_30_36_39	-	-	+
Bosea_sp._Leaf344	-	-	+
Candidatus_Moranbacteria_bacterium_CG_4_8_14_3_um_filter	-	-	+
Bradyrhizobium_sp._CCH4-A6	-	-	+
Rhodococcus_sp.	-	-	+
Bradyrhizobium_sp._NAS80.1	-	-	+
Pseudomonas_sp._NBRC_111144	-	-	+
Betaproteobacteria_bacterium_RIFCSPLOWO2_02_FULL_62_	-	-	+
Candidatus_Magasanikbacteria_bacterium_CG11_big_fil_rev_8_	-	-	+
Candidatus_Levybacteria_bacterium_RIFCSPHIGHO2_02_FUL	-	-	+
Bdellovibrionales_bacterium_RIFOXYD1_FULL_53_11	-	-	+
Rhodonellum_psychrophilum	-	-	+
Ochrobactrum_sp._3-3	-	-	+
Candidatus_Microthrix_parvicella	-	-	+
Bifidobacterium_italicum	-	-	+
Pseudomonas_umsongensis	-	-	+
Pseudomonas_thermotolerans	-	-	+

Ochrobactrum_sp._LM19	-	-	+
Candidatus_Magnetoovum_chiemensis	-	-	+
Rhodobacteraceae_bacterium_HLUCCA08	-	-	+
Candidatus_Marinamargulisbacteria_bacterium_SCGC_AG-343	-	-	+
Betaproteobacteria_bacterium_RIFCSPLOWO2_12_FULL_66_	-	-	+
Pontibacter_sp._BAB1700	-	-	+
Photobacterium_leiognathi	-	-	+
Nakamurella_lactea	-	-	+
Candidatus_Buchananbacteria_bacterium_CG10_big_fil_rev_8_	-	-	+
Salipaludibacillus_agaradhaerens	-	-	+
Sphingomonas_sanxanigenens	-	-	+
Planctomycetes_bacterium_SM23_65	-	-	+
Planomicrobium_okeanokoites	-	-	+
Salinicoccus_qingdaonensis	-	-	+
Salinicoccus_kekensis	-	-	+
Polaribacter_sp._KT25b	-	-	+
Candidatus_Amesbacteria_bacterium_RIFCSPHIGHO2_01_FU	-	-	+
Candidatus_Amesbacteria_bacterium_GW2011_GWC2_45_19	-	-	+
Planococcus_rifietoensis	-	-	+
Myxococcus_stipitatus	-	-	+
Herbaspirillum_rubrisubalbicans	+	+	+
Phaeospirillum_molischianum	-	+	+
Parageobacillus_toebii	-	+	+
Paenibacillus_sp._JCM_10914	+	+	+
Batrachochytrium_salamandrivorans	+	+	+
Peptoniphilus_timonensis	+	+	+
Deltaproteobacteria_bacterium_HGW-Deltaproteobacteria-21	+	+	+
Helicobacter_sp._12S02634-8	+	+	+
Bacillus_galactosidilyticus	+	+	+
Olsenella_phocaeensis	+	+	+
Stenotrophomonas_sp._VV52	+	+	+
Parageobacillus_caldoxylosilyticus	+	+	+
Paraburkholderia_tuberum	-	+	+
Phycomyces_blakesleeanus	+	+	+
Lactobacillus_sp._33-1	-	+	+
Thermoanaerobacter_sp._YS13	+	+	+
Catenaria_anguillulae	+	+	+
Rhizobium_oryziradicis	-	+	+
Pedobacter_kyonggii	+	+	+
Erysipelotrichaceae_bacterium_3_1_53	+	+	+
Ureibacillus_thermosphaericus	+	+	+
Dolichospermum_circinale	+	+	+
Thermoanaerobacter_sp._X514	+	+	+
Streptomyces_scabrisporus	+	+	+
Actinobacillus_pleuropneumoniae	+	+	+
Eubacterium_sp._OF10-16	+	+	+
Acinetobacter_nosocomialis	-	+	+
Lishizhenia_tianjinensis	-	+	+
Listeria_cornellensis	+	+	+
Chitinophagaceae_bacterium_PMP191F	+	+	+
Desulfonatronovibrio_sp._MSAO_Bac4	+	+	+
Alkalibacterium_thalassium	+	+	+
Wallemia_mellicola	+	+	+
Methanoregula_sp._PtaB.Bin085	+	+	+
Microbacterium_indicum	-	+	+

Sulfurospirillum_barnesii	-	+	+
Comamonadaceae_bacterium_CG12_big_fil_rev_8_21_14_0_6	-	+	+
Syntrophus_sp._PtaB.Bin138	+	+	+
Paenibacillus_sp._Soil724D2	+	+	+
Alteromonas_mediterranea	+	+	+
uncultured_bacterium_Contig224	+	+	+
Saitoella_complicata	-	+	+
Polaribacter_sp._Hel1_85	+	+	+
Facklamia_ignava	+	+	+
Brumimicrobium_mesophilum	+	+	+
Leptolyngbya_sp._'hensonii'	+	+	+
Aeromonas_salmonicida	+	+	+
Schaalia_georgiae	+	+	+
Sulfurihydrogenibium_sp.	+	+	+
Deltaproteobacteria_bacterium_RIFCSPLOWO2_12_FULL_57_	+	+	+
Phycisphaerae_bacterium_SM23_30	-	+	+
Vibrio_sp._J2-12	-	+	+
Kaistia_sp._SCN_65-12	-	+	+
Omnitrophica_WOR_2_bacterium_RBG_13_41_10	-	+	+
Thermoflavimicrobium_sp._FBKL4.011	-	+	+
Tessaracoccus_flavus	-	+	+
Fomitopsis_pinicola	-	+	+
Nonomuraea_sp._SBT364	+	+	+
Bacillus_panaciterrae	+	+	+
Clostridium_sp._K25	+	+	+
Thermococcus_guaymasensis	+	+	+
Lutibacter_sp._BRH_c52	-	+	+
Candidatus_Giovannonibacteria_bacterium_GW2011_GWA2_4	-	+	+
Aquificaceae_bacterium	-	+	+
Candidatus_Dadabacteria_bacterium_CSP1-2	-	+	+
Anaerococcus_rubeinfantis	+	+	+
Chryseobacterium_takakiae	+	+	+
Celeribacter_persicus	+	+	+
Rouxiella_badensis	+	+	+
Spirochaetes_bacterium_GWF1_49_6	+	+	+
Domibacillus_enclensis	-	+	+
Mycoplasma_gallopavonis	-	+	+
Bacillus_sp._Soil768D1	-	+	+
Bifidobacterium_primatium	-	+	+
Hydrogenothermus_sp.	-	+	+
Selenomonas_sp._oral_taxon_478	-	+	+
Rhizobium_sp._CF080	-	+	+
Sporolactobacillus_nakayamae	+	+	+
Flavobacterium_croceum	+	+	+
Vibrio_harveyi	+	+	+
Brumimicrobium_glaciale	+	+	+
Anaerococcus_tetradius	+	+	+
Ventosimonas_gracilis	+	+	+
Helcococcus_massiliensis	+	+	+
Aeromonas_sp._ASNIH5	+	+	+
Flavobacterium_sp._YIM_102600	+	+	+
Dactylellina_haptotyla	-	+	+
Lonsdalea_populi	-	+	+
Candidatus_Methylomirabilis_oxyfera	-	+	+
Listeria_rocourtiae	-	+	+

Ochrobactrum_sp._MYb29	-	+	+
Glycomyces_artemisiae	-	+	+
Candidatus_Kentron_sp._SD	-	+	+
Bacillus_mediterraneensis	-	+	+
Desulfobacteraceae_bacterium_4572_130	-	+	+
Kocuria_halotolerans	-	+	+
Bacteriovorax_sp._MedPE-SWde	-	+	+
Terribacillus_halophilus	+	+	+
Elusimicrobium_sp._An273	+	+	+
uncultured_virus	+	+	+
Flagellimonas_sp._HME9304	+	+	+
Phenylobacterium_sp._Root1277	+	+	+
Paraliobacillus_ryukyuensis	+	+	+
Clostridiaceae_bacterium_JG1575	+	+	+
Paenibacillus_sp._VTT_E-133291	+	+	+
Bacillus_terrae	+	+	+
Halalkalibacillus_sp._B3227	+	+	+
Leadbetterella_byssophila	+	+	+
Pelagirhabdus_alkalitolerans	+	+	+
Spirosoma_radiotolerans	+	+	+
Candidatus_Accumulibacter_aalborgensis	-	+	+
Mesotoga_prima	-	+	+
Anaerolineae_bacterium_SG8_19	-	+	+
candidate_division_SR1_bacterium_CG_4_9_14_3_um_filter_4	-	+	+
Galidia_ERV	-	+	+
Burkholderiales_bacterium_35-55-47	-	+	+
Ignatzschineria_larvae	-	+	+
Acidocella_sp._20-57-95	-	+	+
Deltaproteobacteria_bacterium_CG12_big_fil_rev_8_21_14_0_(	-	+	+
uncultured_Sanguibacter_sp.	-	+	+
Trichophyton_violaceum	-	+	+
Pseudomonas_sp._NBRC_111124	-	+	+
Celeribacter_manganoxidans	-	+	+
Spongiibacter_tropicus	-	+	+
Methylocucumis_oryzae	-	+	+
Chloroflexi_bacterium_RBG_16_47_49	-	+	+
Virgibacillus_dokdonensis	-	+	+
Pontibacter_lucknowensis	+	+	+
Arcobacter_mytili	+	+	+
Spiroplasma_clarkii	+	+	+
Parvimonas_sp._KA00067	+	+	+
Candidatus_Accumulibacter_phosphatis	+	+	+
Microbacterium_sp._RURRCA19A	+	+	+
Thermosipho_atlanticus	+	+	+
Thermosipho_africanus	+	+	+
Facklamia_miroungae	+	+	+
Pediococcus_pentosaceus	+	+	+
Fistulina_hepatica	+	+	+
Runella_slithyformis	-	+	+
Pleomorphomonas_carboxyditropha	-	+	+
Candidatus_Latescibacteria_bacterium_4484_181	-	+	+
Nitriliruptor_alkaliphilus	-	+	+
bacterium_HR17	-	+	+
Candidatus_Pelagibacter_ubique	-	+	+
Chondromyces_apiculatus	-	+	+

Lentinus_tigrinus	-	+	+
Zygosaccharomyces_bailii	-	+	+
Vibrio_tritonius	-	+	+
Blastomyces_percursus	-	+	+
Berkelbacteria_bacterium_GW2011_GWA1_36_9	-	+	+
Streptococcus_sp._HMSC073A12	-	+	+
Asinibacterium_sp._OR53	-	+	+
Sporisorium_reilianum	-	+	+
Rubrobacter_aplysinae	-	+	+
Lactobacillus_allii	-	+	+
Cellvibrionales_bacterium_TMED21	-	+	+
Fluviicola_sp.	-	+	+
Dolosicoccus_paucivorans	-	+	+
Geodermatophilus_siccatus	-	+	+
Geitlerinema_sp._PCC_7407	-	+	+
Gracilimonas_amylolytica	-	+	+
Slackia_exigua	+	+	+
uncultured_Desulfofustis_sp._PB-SRB1	+	+	+
Alteromonas_sp._RKMC-009	+	+	+
Cellulosimicrobium_terreum	+	+	+
Pseudovibrio_sp._Ab134	+	+	+
Streptococcus_varani	-	+	+
Rhizobium_yanglingense	-	+	+
Exophiala_xenobiotica	-	+	+
Afipia_sp._P52-10	-	+	+
Deltaproteobacteria_bacterium_GWA2_43_19	-	+	+
Larkinella_sp._ZZJ9	-	+	+
Brachymonas_denitrificans	+	-	+
Cetia_pacifica	+	-	+
Oceanobacillus_damuensis	+	-	+
Spirochaetae_bacterium_HGW-Spirochaetae-3	+	-	+
Lactobacillus_kalixensis	+	-	+
Verrucomicrobia_bacterium_12-59-8	+	-	+
Synergistales_bacterium_53_16	+	-	+
Parcubacteria_group_bacterium_GW2011_GWF2_38_76	+	-	+
Methanobrevibacter_oralis	+	-	+
Thermoanaerobacter_italicus	+	-	+
Chryseobacterium_sp._Leaf201	+	-	+
Rhizobium_phaseoli	+	-	+
Lactobacillus_amylovorus_CAG:719	+	-	+
Pseudothermotoga_hypogea	+	-	+
Bacillus_sp._FJAT-14578	+	-	+
Salimicrobium_halophilum	+	-	+
Salegentibacter_sp._Hel_I_6	+	-	+
Methanobacterium_lacus	+	-	+
Mesoplasma_lactucae	+	-	+
Clostridium_sp._OM07-10AC	+	-	+
Gloeomargarita_lithophora	+	-	+
Archaeoglobus_sulfaticallidus	+	-	+
Clavispora_lusitaniae	+	-	+
Flavobacteriales_bacterium_32-34-25	+	-	+
Devosia_sp._Root436	+	-	+
Candidatus_Kuenenbacteria_bacterium_RIFCSPHIGHO2_02_F	+	-	+
Chryseobacterium_sp._YR005	+	-	+
Rhodococcus_sp._OK269	+	-	+

Idiomarina_sp._OT37-5b	+	-	+
Fictibacillus_phosphorivorans	+	-	+
Deltaproteobacteria_bacterium_HGW-Deltaproteobacteria-12	+	-	+
Paenibacillaceae_bacterium_JTherm	+	-	+
Serratia_phage_2050HW	+	-	+
Carnobacterium_pleistocenium	+	-	+
Niastella_vici	+	-	+
candidate_division_WOR-1_bacterium_RIFCSPHIGHO2_01_F	+	-	+
Candidatus_Magasanikbacteria_bacterium_GW2011_GWC2_34	+	-	+
Thiomonas_sp._FB-Cd	+	-	+
Cecembia_lonarensis	+	-	+
Planococcus_versutus	+	-	+
Bacillus_solani	+	-	+
Hyunsoonleella_sp._T58	+	-	+
Pontibacillus_litoralis	+	-	+
Candidatus_Altiarchaeales_archaeon_A3	+	-	+
Polaribacter_haliotis	+	-	+
Gemmatimonas_sp._SM23_52	+	-	+
Zunongwangia_atlantica	+	-	+
Thermomonas_haemolytica	+	-	+
Arcticibacter_pallidicorallinus	+	-	+
Mycoplasma_testudinis	+	-	+
Rhizobium_tropici	+	-	+
Rhodoplanes_roseus	+	-	+
Bacillus_sp._FJAT-44921	+	-	+
Trabulsiella_odontotermitis	+	-	+
Actinobacteria_bacterium_RBG_16_64_13	+	-	+
Mycoplasma_iowae	+	-	+
Anoxybacillus_vitaminiphilus	+	-	+
Lactobacillus_spicheri	+	-	+
Anabaena_sp._WA102	+	-	+
Coraliomargarita_akajimensis	+	-	+
Methylosinus_sp._R-45379	+	-	+
Nostoc_flagelliforme	+	-	+
Methylomonas_sp.	+	-	+
Chitinophaga_rhizosphaerae	+	-	+
Agrobacterium_tumefaciens	+	-	+
Desulfofundulus_thermocisternus	+	-	+
Olsenella_sp._TM06-36	+	-	+
Candidatus_Thioglobus_sp.	+	-	+
Rhodospirillales_bacterium	+	-	+
Collinsella_bouchesdurhonensis	+	-	+
Bifidobacterium_callitrichos	+	-	+
Candidatus_Omnitrophica_bacterium_CG1_02_46_14	+	-	+
Acinetobacter_piscicola	+	-	+
Oceanicella_sp._SM1341	+	-	+
Confluentibacter_sp._3B	+	-	+
Armatimonadetes_bacterium_CSP1-3	+	-	+
Thalassobacillus_sp._TM-1	+	-	+
Terribacillus_aidingensis	+	-	+
Kwoniella_heveanensis	+	-	+
Lactobacillus_sp._BCRC_12945	+	-	+
Candidatus_Nealsonbacteria_bacterium_CG_4_8_14_3_um_filt	+	-	+
Microbacterium_halotolerans	+	-	+
Collinsella_sp._AM31-2AC	+	-	+

Marinomonas_sp._YLB-05	+	-	+
Niabella_ginsenosidivorans	+	-	+
Halioglobus_sp._HI00S01	+	-	+
Duganella_sp._CF517	+	-	+
Flavobacterium_aciduliphilum	+	-	+
Corynebacterium_choanis	+	-	+
Rhodospirillales_bacterium_URHD0088	+	-	+
Flavobacterium_granuli	+	-	+
Flavobacterium_flevense	+	-	+
Bacillus_jeotgali	+	-	+
Alces_alces_faeces_associated_microvirus_MP21_4718	+	-	+
Dasania_marina	-	-	+
Fibrisoma_sp._HYT19	-	-	+
Halobacillus_karajensis	-	-	+
Thermotoga_caldifontis	-	-	+
Marinitoga_sp._LG1	-	-	+
Tamlana_sp._s12	-	-	+
Actinotignum_urinale	-	-	+
Bacillus_virus_B103	-	-	+
Trametes_coccinea	-	-	+
Acetobacteraceae_bacterium_DB1506	-	-	+
Flavisolibacter_sp._X7X	-	-	+
Brenneria_goodwinii	-	-	+
Verrucomicrobia_bacterium_TMED71	-	-	+
Deltaproteobacteria_bacterium_HGW-Deltaproteobacteria-19	-	-	+
Streptococcus_sp._263_SSPC	-	-	+
[Candida]_glabrata	-	-	+
Chryseobacterium_shigense	-	-	+
Facklamia_sourekii	-	-	+
Rhodobacteraceae_bacterium_LMIT002	-	-	+
Rhodosalinus_sp._E84	-	-	+
Methyloprofundus_sedimenti	-	-	+
Rhodoferax_sp._YR267	-	-	+
Legionella_taurinensis	-	-	+
Polyangiaceae_bacterium_UTPRO1	-	-	+
Amphritea_sp._MCCC_1K03512	-	-	+
Dyadobacter_fermentans	-	-	+
Azospira_oryzae	-	-	+
Lysinibacillus_sp._YS11	-	-	+
Limnobacter_thiooxidans	-	-	+
Lachancea_fermentati	-	-	+
Cupriavidus_sp._USMAA2-4	-	-	+
Lactobacillus_sp._UMNPBX5	-	-	+
Tolypothrix_sp._NIES-4075	-	-	+
Limnohabitans_sp._MMS-10A-192	-	-	+
Pachysolen_tannophilus	-	-	+
Enterocytozoon_bieneusi	-	-	+
Leptospirillum_ferrodiazotrophum	-	-	+
Streptococcus_respiraculi	-	-	+
Haloferula_sp._BvORR071	-	-	+
Natronorubrum_sulfidifaciens	-	-	+
Bacillus_sp._FJAT-27251	-	-	+
Nonomuraea_sp._WAC_01424	-	-	+
Edhazardia_aedis	-	-	+
Xanthophyllomyces_dendrorhous	-	-	+

Protomyces_lactucaedebilis	-	-	+
Pseudomonas_sp._T	-	-	+
Paenisporosarcina_indica	-	-	+
uncultured_bacterium_Contig13	-	-	+
Caldithrix_sp._RBG_13_44_9	-	-	+
Hydrogenophaga_sp._LA-38	-	-	+
candidate_division_WOR-1_bacterium_RIFOXYB2_FULL_36_	-	-	+
Winogradskyella_tangerina	-	-	+
Helicobacter_brantae	-	-	+
Alkalibacillus_haloalkaliphilus	-	-	+
Haloprofundus_marisrubri	-	-	+
Humibacter_albus	-	-	+
Hoeflea_sp._BRH_c9	-	-	+
Achromobacter_sp._Root565	-	-	+
uncultured_Prevotella_sp.	-	-	+
uncultured_bacterium_IN-05	-	-	+
Deltaproteobacteria_bacterium_RBG_13_49_15	-	-	+
uncultured_bacterium_DCM007Kan03	-	-	+
uncultured_bacterium_DCM007Kan02	-	-	+
Planktothricoides_sp._SR001	-	-	+
Phyllobacterium_sp._OV277	-	-	+
Paenibacillus_sp._A59	-	-	+
Candidatus_Omnitrophica_bacterium_CG11_big_fil_rev_8_21_	-	-	+
Deltaproteobacteria_bacterium_HGW-Deltaproteobacteria-15	-	-	+
Deinococcus_sp._Leaf326	-	-	+
Planctomycetes_bacterium_HGW-Planctomycetes-1	-	-	+
Exiguobacterium_sp._AM39-5BH	-	-	+
Candidatus_Peregrinibacteria_bacterium_CG10_big_fil_rev_8_[illegible]	-	-	+
Veillonella_sp._VA142	-	-	+
Deltaproteobacteria_bacterium_GWA2_55_82	-	-	+
Deltaproteobacteria_bacterium_GWC2_42_11	-	-	+
Paenibacillus_solani	-	-	+
Deltaproteobacteria_bacterium_HGW-Deltaproteobacteria-11	-	-	+
Alteromonadaceae_bacterium	-	-	+
Candidatus_Accumulibacter_sp._BA-91	-	-	+
Alteribacillus_bidgolensis	-	-	+
Altererythrobacter_sp._Ery12	-	-	+
Desulfobacteraceae_bacterium_4572_35.2	-	-	+
uncultured_bacterium_Contig1514	-	-	+
Candidatus_Micrarchaeota_archaeon	-	-	+
Filibacter_sp._TB-66	-	-	+
Borrelia_duttonii	-	-	+
Erwinia_virus_Ea2809	-	-	+
Candidatus_Moranbacteria_bacterium_RIFCSPHIGHO2_02_FU	-	-	+
Vibrio_rumoiensis	-	-	+
Vibrio_sp._BEI233	-	-	+
Erwinia_phage_vB_EamM_Joad	-	-	+
Pseudoalteromonas_sp._NJ631	-	-	+
Duganella_sp._CF458	-	-	+
Flavobacterium_sp._Leaf359	-	-	+
Acinetobacter_sp._ANC_3832	-	-	+
Ensifer_sp._YR511	-	-	+
Candidatus_Campbellbacteria_bacterium_CG22_combo_CG10-	-	-	+
Aeromonas_sp._ASNIH7	-	-	+
Flavobacterium_plurextorum	-	-	+

Parcubacteria_group_bacterium_GW2011_GWF2_39_13b	-	-	+
bacterium_HR28	-	-	+
Candidatus_Fraserbacteria_bacterium_RBG_16_55_9	-	-	+
Elusimicrobia_bacterium_RIFCSPLOWO2_01_FULL_59_12	-	-	+
candidate_division_WOR_3_bacterium_SM23_60	-	-	+
Actinobacteria_bacterium_HGW-Actinobacteria-9	-	-	+
Prochlorothrix_hollandica	-	-	+
Candidatus_Dependentiae_bacterium_HGW-Dependentiae-1	-	-	+
candidate_division_WOR-1_bacterium_RIFOXYA2_FULL_36_	-	-	+
candidate_division_WWE3_bacterium	-	-	+
Prevotella_sp._Sc00033	-	-	+
Actinomyces_hordeovulneris	-	-	+
Fusobacterium_sp._OBRC1	-	-	+
Candidatus_Desantisbacteria_bacterium_CG1_02_49_89	-	-	+
Candidatus_Berkelbacteria_bacterium_RBG_13_40_8	-	-	+
Flavobacteriales_bacterium_TMED288	-	-	+
Candidatus_Kentron_sp._DK	-	-	+
Pseudomonas_plecoglossicida	-	-	+
Candidatus_Kaiserbacteria_bacterium_CG10_big_fil_rev_8_21_	-	-	+
Desulfurobacterium_sp.	-	-	+
Pseudomonas_sp._LAB-08	-	-	+
Enterococcus_sp._CR-Ec1	-	-	+
Candidatus_Levybacteria_bacterium_RIFCSPHIGHO2_01_FUI	-	-	+
Pseudomonas_sp._FW305-130	-	-	+
Yaba_monkey_tumor_virus	-	-	+
Paracoccus_homiensis	-	-	+
candidate_division_WOR-1_bacterium_RIFOXYB2_FULL_37_	-	-	+
Enterococcus_sp._3G6_DIV0642	-	-	+
bacterium_BMS3Bbin06	-	-	+
Agromyces_atrinae	-	-	+
Glycomyces_sp._NRRL_B-16210	-	-	+
Agrobacterium_phage_Atu_ph07	-	-	+
bacterium_B17	-	-	+
Acidobacteriales_bacterium_59-55	-	-	+
Geovibrio_sp._L21-Ace-BES	-	-	+
Parcubacteria_group_bacterium_GW2011_GWA2_38_13b	-	-	+
Candidatus_Phytoplasma_solani	-	-	+
Candidatus_Arthromitus_sp._SFB-5	-	-	+
Caldicellulosiruptor_lactoaceticus	-	-	+
Desulfurococcus_amylolyticus	-	-	+
[Butyribacterium]_methylotrophicum	-	-	+
Acidobacteria_bacterium_RBG_13_68_16	-	-	+
Candidatus_Hydrogenedentes_bacterium_CG1_02_42_14	-	-	+
Marinobacter_sp._LQ44	-	-	+
Streptomyces_subrutilus	-	-	+
Streptomyces_thermoautotrophicus	-	-	+
Nitritalea_halalkaliphila	-	-	+
Ruminococcus_sp._AF17-6LB	-	-	+
Cesiribacter_andamanensis	-	-	+
Bacillus_luti	-	-	+
Mannheimia_massilioguelmaensis	-	-	+
Nitrosomonas_communis	-	-	+
Cellvibrionales_bacterium_TMED47	-	-	+
Bacillus_lehensis	-	-	+
SAR202_cluster_bacterium_Ae2-Chloro-G1	-	-	+

Mesorhizobium_sp._DCY119	-	-	+
Streptomyces_sp._CB02923	-	-	+
Melanoplus_sanguinipes_entomopoxvirus	-	-	+
Streptomyces_sp._CC0208	-	-	+
Saccharomyces_cerevisiae_x_Saccharomyces_kudriavzevii	-	-	+
Megasphaera_genomosp._type_1	-	-	+
Bacteroides_sp._SM23_62	-	-	+
Streptomyces_sp._NBS_14/10	-	-	+
Nitrospinae_bacterium_RIFCSPLOWO2_12_39_16	-	-	+
Lysinibacillus_sp._AC-3	-	-	+
Bacillaceae_bacterium_SAOS_7	-	-	+
Cohaesibacter_gelatinilyticus	-	-	+
Rickettsiales_bacterium_TMED174	-	-	+
Nocardia_puris	-	-	+
Rhodotorula_taiwanensis	-	-	+
Thalassospira_sp._GB04J01	-	-	+
Nonlabens_sp._MB-3u-79	-	-	+
Nitrospirae_bacterium_RBG_16_43_8	-	-	+
Roseovarius_sp._GCL-8	-	-	+
Cellulomonas_marina	-	-	+
Celeribacter_baekdonensis	-	-	+
Leuconostoc_pseudomesenteroides	-	-	+
Nitrospirae_bacterium_GWD2_57_9	-	-	+
Salimicrobium_jeotgali	-	-	+
Bacillus_sp._UMB0893	-	-	+
Solemya_elarraichensis_gill_symbiont	-	-	+
Microgenomates_group_bacterium_GW2011_GWA1_48_10	-	-	+
Microdochium_bolleyi	-	-	+
Snowella_sp.	-	-	+
Microbulbifer_sp._HZ11	-	-	+
Stenotrophomonas_koreensis	-	-	+
Chroogloeocystis_siderophila	-	-	+
Stenotrophomonas_sp._WZN-1	-	-	+
Sphingopyxis_flava	-	-	+
Bacillus_weihaiensis	-	-	+
Spiroplasma_diminutum	-	-	+
Sphingobacteriales_bacterium_BACL12_MAG-120813-bin55	-	-	+
Sphingobium_sp._Ant17	-	-	+
Sphingobium_baderi	-	-	+
Mycoplasma_californicum	-	-	+
Monosporascus_sp._mg162	-	-	+
Mesorhizobium_sp._M2C.T.Ca.TU.009.01.2.1	-	-	+
Bacillus_sp._MYb209	-	-	+
Natrialba_magadii	-	-	+
Bacillus_sp._AFS059628	-	-	+
Sanguibacter_keddieii	-	-	+
Chloroflexi_bacterium_GWB2_54_36	-	-	+
Bacillus_sp._AFS015896	-	-	+
Mesotoga_sp._SC_NapDC	-	-	+
Salinicoccus_carnicancri	-	-	+
Methanoregula_sp._PtaU1.Bin006	-	-	+
Methanoregulaceae_archaeon_PtaB.Bin056	-	-	+
Shimazuella_kribbensis	-	-	+
Mycoplasma_ovipneumoniae	-	-	+
Mycoplasma_sturni	-	-	+

Bacillus_sp._FJAT-45066	-	-	+
Chloroflexi_bacterium_RBG_13_46_14	-	-	+
Chloroflexi_bacterium_RIFCSPLOWO2_02_FULL_71_16	-	-	+
Nonomuraea_solani	-	-	+
Avibacterium_gallinarum	-	-	+
Oenococcus_sicerae	-	-	+
Corynebacterium_renale	-	-	+
Candidatus_Schekmanbacteria_bacterium_GWA2_38_9	-	-	+
Thermus_sp._L198	-	-	+
Candidatus_Shapirobacteria_bacterium_CG09_land_8_20_14_0	-	-	+
Candidatus_Uhrbacteria_bacterium_RIFCSPHIGHO2_12_FULI	-	-	+
Lactobacillus_oligofermentans	-	-	+
Betaproteobacteria_bacterium_UKL13-2	-	-	+
Bifidobacteriaceae_bacterium_NR026	-	-	+
Lactobacillus_hayakitensis	-	-	+
Lactobacillus_floricola	-	-	+
Thermoplasmatales_archaeon_I-plasma	-	-	+
Trichophyton_rubrum	-	-	+
Trichormus_variabilis	-	-	+
Kitasatospora_sp._MMS16-BH015	-	-	+
Kingella_sp.	-	-	+
Cytophagales_bacterium_CG12_big_fil_rev_8_21_14_0_65_40	-	-	+
Oligella_sp._HMSC05A10	-	-	+
Ureaplasma_canigenitalium	-	-	+
Ureaplasma_diversum	-	-	+
Candidatus_Riflebacteria_bacterium_HGW-Riflebacteria-2	-	-	+
Ramlibacter_tataouinensis	-	-	+
Kluyveromyces_lactis	-	-	+
Ancylobacter_sp._FA202	-	-	+
Bizionia_echini	-	-	+
Candidatus_Saccharibacteria_bacterium_RIFCSPHIGHO2_01_I	-	-	+
Cyanobacteria_bacterium_QH_9_48_43	-	-	+
Rhizobium_sp._11515TR	-	-	+
Omnitrophica_bacterium_RIFCSPHIGHO2_02_FULL_63_14	-	-	+
Lactobacillus_sp._247-4	-	-	+
Bdellovibrio_exovorus	-	-	+
Lactobacillus_sp._M1575	-	-	+
Coprobacillus_sp._AF18-40	-	-	+
Rhodohalobacter_sp._SW132	-	-	+
Candidatus_Wirthbacteria_bacterium_CG2_30_54_11	-	-	+
Collinsella_sp._TF12-2AT	-	-	+
Candidatus_Yanofskybacteria_bacterium_CG10_big_fil_rev_8_	-	-	+
Bdellovibrionales_bacterium_GWA2_49_15	-	-	+
Lactobacillus_sp._116-2	-	-	+
Candidatus_Wolfebacteria_bacterium_CG03_land_8_20_14_0_	-	-	+
Aspergillus_bombycis	-	-	+
Lactobacillus_sp._47-3	-	-	+
Arthrobacter_sp._EpRS66	-	-	+
Fictibacillus_gelatini	+	+	+
Seinonella_peptonophila	+	+	+
Paenibacillus_sp._Soil522	+	+	+
Marine_Group_I_thaumarchaeote_SCGC_AAA799-O18	+	+	+
Streptococcus_marimammalium	+	+	+
Paenibacillus_ihbetae	+	+	+
Gordonia_phage_GMA6	-	+	+

Laceyella_sacchari	-	+	+
Nodularia_spumigena	+	+	+
Syncephalastrum_racemosum	+	+	+
Candidatus_Phytoplasma_australiense	+	+	+
Lactobacillus_sp._187-3	-	+	+
Bacillus_sp._FJAT-42376	+	+	+
Porphyromonadaceae_bacterium_FC4	+	+	+
Geobacter_uraniireducens	+	+	+
Lactobacillus_songhuajiangensis	+	+	+
Gallaecimonas_pentaromativorans	-	+	+
Pseudomonas_pseudoalcaligenes	-	+	+
Elusimicrobium_minutum	-	+	+
Hydrogenophaga_sp._A37	-	+	+
Geobacter_metallireducens	+	+	+
Flavobacterium_suncheonense	+	+	+
Campylobacter_sp._P160	+	+	+
Vitiosangium_sp._GDMCC_1.1324	+	+	+
Tremella_mesenterica	+	+	+
Sulfitobacter_geojensis	+	+	+
Chryseobacterium_sp._FP211-J200	-	+	+
Sphingopyxis_granuli	-	+	+
Paenibacillus_sp._Root52	+	+	+
Bernardetia_litoralis	+	+	+
Bacillaceae_bacterium_EAG3	+	+	+
Desulfuromonadales_bacterium_GWD2_54_10	+	+	+
Natranaerobius_thermophilus	-	+	+
Calothrix_elsteri	-	+	+
Chloroflexi_bacterium_UTCFX4	-	+	+
Methanosphaera_sp._SHI1033	-	+	+
Gammaproteobacteria_bacterium_HGW-Gammaproteobacteria-1	+	+	+
Rivularia_sp._PCC_7116	+	+	+
Pseudomonas_sp._NFACC19-2	+	+	+
Schaalia_odontolytica	+	+	+
Candidatus_Pelagibacter_sp._TMED263	-	+	+
Confluentibacter_lentus	-	+	+
Capnocytophaga_sp._oral_taxon_878	-	+	+
Pseudomonas_formosensis	-	+	+
Chryseobacterium_piscium	-	+	+
Nitrosomonas_aestuarii	+	-	+
Neisseria_flavescens	+	-	+
Desulfobulbus_japonicus	+	-	+
Mucilaginibacter_rubeus	+	-	+
Leptolyngbya_sp._Heron_Island_J	+	-	+
uncultured_bacterium_Contigcl_7	+	-	+
Micromonospora_chersina	+	-	+
Enterococcus_pseudoavium	+	-	+
Fusobacterium_canifelinum	+	-	+
Paenibacillus_sp._P3E	+	-	+
Collinsella_sp._AM12-1	+	-	+
Bacillus_sp._OG2	+	-	+
Sporolactobacillus_inulinus	+	-	+
Collinsella_sp._AF33-16	+	-	+
Fusobacterium_sp._oral_taxon_203	+	-	+
Tortispora_caseinolytica	+	-	+
Neolecta_irregularis	+	-	+

Deinococcus_metallilatus	+	-	+
Labrys_sp._WJW	+	-	+
Candidatus_Shapirobacteria_bacterium_CG06_land_8_20_14_3	+	-	+
Pseudoalteromonas_sp._R3	+	-	+
Chondrocystis_sp._NIES-4102	-	-	+
Leucoagaricus_sp._SymC.cos	-	-	+
Syntrophorhabdaceae_bacterium_PtaU1.Bin034	-	-	+
Dickeya_sp._S29	-	-	+
Gammaproteobacteria_bacterium_SCGC_AG-212-F23	-	-	+
Chryseobacterium_sp._VAUSW3	-	-	+
Flavobacteriales_bacterium_CG_4_10_14_0_2_um_filter_32_8	-	-	+
Lachancea_meyersii	-	-	+
Oxalobacteraceae_bacterium	-	-	+
Morganella_psychrotolerans	-	-	+
Dacryopinax_primogenitus	-	-	+
Bosea_sp._DSM_18164	-	-	+
Microcella_sp._HL-107	-	-	+
Catellatospora_citrea	-	-	+
Microbacterium_sp._BR1	-	-	+
filamentous_cyanobacterium_Phorm_6	-	-	+
Legionella_londiniensis	-	-	+
Deltaproteobacteria_bacterium_CSP1-8	-	-	+
Chryseobacterium_sp._CF365	-	-	+
Deltaproteobacteria_bacterium_CG_4_10_14_3_um_filter_60_8	-	-	+
Nocardioides_immobilis	-	-	+
Jeotgalicoccus_saudimassiliensis	-	-	+
Acetobacter_malorum	-	-	+
Achromobacter_insolitus	-	-	+
Moniliophthora_roreri	-	-	+
Mycoplasma_capricolum	-	-	+
Flavobacterium_commune	-	-	+
Parcubacteria_group_bacterium_GW2011_GWA2_47_16	-	-	+
Cellulomonas_timonensis	-	-	+
Aliterella_atlantica	-	-	+
Propionibacteriaceae_bacterium_P6A17	-	-	+
Roseovarius_sp._217	-	-	+
Pseudomonas_trivialis	-	-	+
Synechococcus_sp._65AY6A5	-	-	+
Beluga_whale_alphaherpesvirus_1	-	-	+
Nitrobacter_vulgaris	-	-	+
Armillaria_gallica	-	-	+
Bradyrhizobium_sp._LTSPM299	-	-	+
Oceaniglobus_indicus	-	-	+
Marinosulfonomonas_sp.	-	-	+
Flavobacterium_rivuli	-	-	+
Nadsonia_fulvescens	-	-	+
Methylobacillus_flagellatus	-	-	+
Paenibacillus_sp._MYb63	-	-	+
Mycoplasma_subdolum	-	-	+
Lachancea_lanzarotensis	-	-	+
Geobacter_bremensis	-	-	+
Candidatus_Sulfotelmatobacter_kueseliae	-	-	+
Methanobrevibacter_sp._AbM4	-	-	+
Wolbachia_endosymbiont_of_Folsomia_candida	-	-	+
Candidatus_Moranbacteria_bacterium_CG_4_9_14_3_um_filter	-	-	+

Flavobacterium_sp._RSP46	-	-	+
Chryseobacterium_sp._XH07	-	-	+
Lactococcus_petauri	-	+	-
Lactococcus_sp._DD01	-	+	-
Crenothrix_polyspora	+	+	-
Feline_leukemia_virus	+	+	-
Simian_retrovirus_Y	+	+	-
Cetobacterium_sp._ZOR0034	-	+	-
Treponema_sp._GWC1_61_84	+	+	-
Rubrobacter_xylanophilus	+	+	-
Candidatus_Kerfeldbacteria_bacterium_RIFCSPHIGHO2_12_F	+	+	-
Gammaproteobacteria_bacterium_TMED104	+	+	-
Actinomycetales_bacterium_JB111	+	+	-
Bhargavaea_beijingensis	-	+	-
Candidatus_Aminicenantes_bacterium	-	+	-
Fischerella_muscicola	+	+	-
Nostoc_sp._PCC_7107	+	+	-
Lyngbya_sp._PCC_8106	+	+	-
candidate_division_MSBL1_archaeon_SCGC-AAA259I07	-	+	-
Pseudomonas_sp._2822-17	-	+	-
Roseivirga_echinicomitans	-	+	-
Mycobacterium_virus_Faith1	-	+	-
Candidatus_Yanofskybacteria_bacterium_RIFCSPHIGHO2_01_	+	+	-
Paenibacillus_sp._CF095	+	+	-
Corallococcus_coralloides	-	+	-
Pseudomonas_sp._GM60	-	+	-
Echidna_ERV	+	+	-
Betaproteobacteria_bacterium_HGW-Betaproteobacteria-5	+	+	-
Mucilaginibacter_sp._OK098	+	+	-
Rhizopus_microsporus	+	+	-
Lobosporangium_transversale	+	+	-
Enterococcus_sp._TR	+	+	-
Anaerobiospirillum_succiniciproducens	+	+	-
Allomyces_macrogynus	+	+	-
Mus_musculus_mobilized_endogenous_polytropic_provirus	-	+	-
Bovine_gammaherpesvirus_6	+	+	-
Methanoregulaceae_archaeon_PtaB.Bin152	+	+	-
Myxosarcina_sp._GI1	+	+	-
Cytophaga_hutchinsonii	-	+	-
Lactobacillus_composti	-	+	-
Deltaproteobacteria_bacterium_RBG_13_60_28	+	+	-
Dimargaris_cristalligena	+	+	-
Linderina_pennispora	+	+	-
Methylomicrobium_agile	+	+	-
Enterococcus_phage_IME_EF3	-	+	-
Elusimicrobia_bacterium_RIFOXYA2_FULL_39_19	+	+	-
Erysiphe_pulchra	+	+	-
Geotrichum_candidum	+	+	-
Acidaminococcus_sp._BV3L6	+	+	-
Bacillus_sp._UMB0728	+	+	-
Epulopiscium_sp._Nele67-Bin001	+	+	-
Aerococcus_christensenii	-	+	-
Ceraceosorus_guamensis	-	+	-
Globicatella_sanguinis	+	+	-
Candidatus_Taylorbacteria_bacterium_RIFCSPHIGHO2_02_FU	+	+	-

Gallionellales_bacterium_RIFCSPLOWO2_02_FULL_57_47	+	+	-
Streptococcus_sp._HMSC063B03	-	+	-
Candidatus_Peregrinibacteria_bacterium_GW2011_GWA2_33_	-	+	-
candidate_division_TM6_bacterium_GW2011_GWF2_30_66	-	+	-
Rhizobium_oryzae	+	+	-
Streptococcus_sp._28462	+	+	-
Chrysoporthe_austroafricana	+	+	-
Streptococcus_penaeicida	+	+	-
Lactobacillus_sp._CBA3605	-	+	-
Armatimonadetes_bacterium_13_1_20CM_4_65_7	-	+	-
Arcticibacterium_luteifluviistationis	-	+	-
Serratia_quinivorans	-	+	-
Streptomyces_sp._MH60	-	+	-
Beggiatoa_sp._IS2	-	+	-
Coprobacillus_sp._AM32-11LB	+	+	-
Nocardioides_sp._CCTCC_AB_2018079	+	+	-
Paenibacillus_sp._SSG-1	+	+	-
Smittium_mucronatum	+	+	-
Crinalium_epipsammum	-	+	-
Saccharomyces_cerevisiae	-	+	-
Akkermansia_muciniphila_CAG:154	+	+	-
Bacillus_sp._7884-1	+	+	-
Enterococcus_phage_vB_EfaS_AL2	+	+	-
Paenibacillus_sp._EZ-K15	+	+	-
Sulfobacillus_thermosulfidooxidans	+	+	-
Hyunsoonleella_pacifica	+	+	-
Mucor_ambiguus	-	+	-
Kibdelosporangium_aridum	-	+	-
Halothiobacillus_neapolitanus	-	+	-
Deltaproteobacteria_bacterium_RBG_16_48_10	-	+	-
Mycena_chlorophos	-	+	-
Vibrio_diabolicus	-	+	-
Actinotignum_sanguinis	-	+	-
Ignavibacteria_bacterium_GWA2_55_25	-	+	-
Ovine_enzootic_nasal_tumor_virus	+	+	-
Candidatus_Collierbacteria_bacterium_CG17_big_fil_post_rev_	+	+	-
Paenibacillus_sp._OK060	+	+	-
Erysipelothrix_tonsillarum	+	+	-
Bacillus_bataviensis	+	+	-
Caldilinea_aerophila	+	+	-
Absidia_repens	-	+	-
Parcubacteria_group_bacterium_GW2011_GWA2_31_28	-	+	-
Euryhalocaulis_caribicus	-	+	-
Pseudoalteromonas_sp._CO348	-	+	-
Thermoanaerobacter_thermocopriae	-	+	-
Colwellia_sp._12G3	-	+	-
Oceanobacillus_sp._Castelsardo	-	+	-
Spirochaeta_cellobiosiphila	-	+	-
Enterococcus_phage_Nonaheksakonda	-	+	-
Staphylococcus_equorum	+	+	-
Anaerolinea_thermolimosa	+	+	-
Bacillus_psychrosaccharolyticus	+	+	-
Pleurotus_ostreatus	+	+	-
Anaerococcus_vaginalis	+	+	-
Glaesserella_parasuis	+	+	-

Campylobacter_insulaenigrae	+	+	-
[Flavobacterium]_thermophilum	+	+	-
Marinitoga_sp._1155	+	+	-
Bovine_retrovirus_CH15	+	+	-
Magnetospirillum_sp._15-1	+	+	-
Cyberlindnera_fabianii	-	+	-
Serpula_lacrymans	-	+	-
Streptomyces_bungoensis	-	+	-
Bacillus_vireti	-	+	-
Candidatus_Woesebacteria_bacterium_RBG_16_34_12	-	+	-
Candidatus_Yanofskybacteria_bacterium_CG10_big_fil_rev_8_	-	+	-
Campylobacter_sp._P162	-	+	-
Halolactibacillus_alkaliphilus	+	+	-
Candidatus_Heimdallarchaeota_archaeon_AB_125	+	+	-
Capnocytophaga_cynodegmi	+	+	-
Bacillus_loiseleuriae	+	+	-
Flammeovirga_sp._SJP92	+	+	-
Sphingomonas_sp._ZDH117	+	+	-
Actinomyces_sp._2119	+	+	-
Paenibacillus_sp._P22	+	+	-
Arthrobacter_sp._DWC3	+	+	-
Rodentibacter_trehalosifermentans	+	+	-
Chloroflexi_bacterium_OLB14	+	+	-
Streptomyces_sp._CNH287	+	+	-
Parasitella_parasitica	+	+	-
Leucobacter_sp._G161	+	+	-
Acidomyces_richmondensis	-	+	-
Candidatus_Thermofonsia_Clade_2_bacterium	-	+	-
Lactobacillus_parabrevis	-	+	-
Coprobacillus_sp._AF34-1BH	-	+	-
Baboon_endogenous_virus	-	+	-
Photorhabdus_luminescens	-	+	-
Streptomyces_sp._PT12	-	+	-
Pelagibacteraceae_bacterium_GOM-A2	-	+	-
uncultured_bacterium_Contig203	-	+	-
Armatimonadetes_bacterium_CG_4_8_14_3_um_filter_66_20	-	+	-
Chaetothyriales_sp._CBS_134920	-	+	-
Claviceps_purpurea	-	+	-
Chryseobacterium_sp._Leaf180	-	+	-
Lactobacillus_acidifarinae	+	+	-
Microvirga_massiliensis	+	+	-
Sporolactobacillus_sp._THM7-4	+	+	-
Aminiphilus_circumscriptus	+	+	-
Robiginitalea_biformata	+	+	-
Pseudoalteromonas_sp._BMB	+	+	-
Mannheimia_haemolytica	+	+	-
Campylobacter_sp._RM8964	+	+	-
[Candida]_intermedia	+	+	-
Planococcus_kocurii	+	+	-
Chryseobacterium_indologenes	+	+	-
Absiella_sp._AM09-50	+	+	-
Lysinibacillus_fluoroglycofenilyticus	+	+	-
Coemansia_reversa	+	+	-
uncultured_bacterium_IN-02	+	+	-
Massilibacterium_senegalense	+	+	-

Rummeliibacillus_sp._POC4	-	+	-
Spiroplasma_turonicum	-	+	-
Absiella_sp._AM22-9	-	+	-
Exiguobacterium_sp._BMC-KP	-	+	-
Acidimicrobiaceae_bacterium_TMED130	-	+	-
Rhodobium_orientis	-	+	-
Reticuloendotheliosis_virus	-	+	-
Pseudozobellia_sp._CCMM003	-	+	-
Pseudoalteromonas_sp._BSi20652	-	+	-
Microcystis_flos-aquae	-	+	-
Chlamydia_avium	-	+	-
Cervidpoxvirus_mule_deer/Oregon/2005	-	+	-
Feline_endogenous_virus	-	+	-
Janthinobacterium_sp._HH107	-	+	-
Ensifer_sp._LC163	-	+	-
Bacteroidetes_bacterium_RIFOXYA12_FULL_33_9	+	+	-
delta_proteobacterium_ML8_F1	+	+	-
Choanephora_cucurbitarum	+	+	-
Arcicella_aurantiaca	+	+	-
Streptomyces_carpinensis	+	+	-
Streptococcus_merionis	+	+	-
Sedimentisphaera_salicampi	+	+	-
Zavarzinella_formosa	+	+	-
Geobacter_bemidjiensis	+	+	-
Listeria_ivanovii	+	+	-
Algoriphagus_zhangzhouensis	+	+	-
Candidatus_Hydrothermarchaeota_archaeon	+	+	-
Geobacillus_sp._WSUCF1	+	+	-
Acidibacillus_ferrooxidans	+	+	-
Tanapox_virus	+	+	-
Paraburkholderia_xenovorans	+	+	-
Bacillus_paramycoides	+	+	-
Equid_gammaherpesvirus_2	+	+	-
Streptococcus_sp._DD10	+	+	-
Kouleothrix_aurantiaca	+	+	-
Candidatus_Aminicenantes_bacterium_RBG_19FT_COMBO_5	+	+	-
Pseudoalteromonas_sp._BSi20439	-	+	-
Aquimarina_atlantica	-	+	-
Suhomyces_tanzawaensis	-	+	-
Proteus_hauseri	-	+	-
Schizophyllum_commune	-	+	-
Schizosaccharomyces_pombe	-	+	-
Phialophora_cf._hyalina_BP_5553	-	+	-
Cyanobacterium_aponinum	-	+	-
Yersinia_pestis	-	+	-
Streptococcus_sp._DORA_10	-	+	-
Mitosporidium_daphniae	-	+	-
Klosneuvirus_KNV1	-	+	-
Candidatus_Moranbacteria_bacterium_GW2011_GWF1_34_10	-	+	-
Alteromonas_macleodii	-	+	-
Bathymodiolus_azoricus_thioautotrophic_gill_symbiont	-	+	-
Nocardia_nova	-	+	-
Enterococcus_phage_phiSHEF4	-	+	-
Bifidobacterium_tsurumiense	-	+	-
Rhodopirellula_sp._SWK7	-	+	-

Clostridium_phage_phiCTP1	-	+	-
Limnothrix_rosea	-	+	-
Acidobacteria_bacterium_Mor1	-	+	-
Porphyrobacter_dokdonensis	-	+	-
Komagataeibacter_xylinus	-	+	-
Lactobacillus_mucosae	+	+	-
Bacillus_sp._CCTCCAB-2014251	+	+	-
Omnitrophica_WOR_2_bacterium_GWB2_45_9	+	+	-
Bacillus_akibai	+	+	-
Candidatus_Woesebacteria_bacterium_GW2011_GWA1_39_21	+	+	-
Oscillatoria_sp._PCC_10802	+	+	-
Natribacillus_halophilus	+	+	-
Megamonas_sp._Calf98-2	+	+	-
Effusibacillus_lacus	+	+	-
Burkholderia_sp._JS23	+	+	-
Caldisericum_exile	+	+	-
Lentibacillus_halodurans	+	+	-
Corynebacterium_glucuronolyticum	+	+	-
Bifidobacterium_stellenboschense	+	+	-
Geobacillus_sp._B4113_201601	+	+	-
Lysobacter_dokdonensis	+	+	-
Acinetobacter_sp._WCHAc060041	+	+	-
Paraliobacillus_sp._PM-2	+	+	-
Terfezia_boudieri	+	+	-
Cereibacter_changlensis	+	+	-
Methanosphaerula_palustris	+	+	-
Oceanobacillus_profundus	+	+	-
Adhaeribacter_sp._HMF7616	+	+	-
Tilletiaria_anomala	+	+	-
Bacillus_sp._m3-13	-	+	-
Pseudarthrobacter_sp._AG30	-	+	-
Avian_leukosis_virus	-	+	-
Rhodospirillaceae_bacterium_SYSU_D60009	-	+	-
Aequorivita_capsosiphonis	-	+	-
Catenuloplanes_japonicus	-	+	-
Micromonospora_violae	-	+	-
Myxococcus_fulvus	-	+	-
Candidatus_Methanolliviera_hydrocarbonicum	-	+	-
Fibroporia_radiculosa	-	+	-
Planctomycetes_bacterium_RBG_13_44_8b	-	+	-
Bifidobacterium_gallicum	-	+	-
Candidatus_Fermentibacter_daniensis	-	+	-
Metarhizium_album	-	+	-
Aequorivita_vladivostokensis	-	+	-
Chloroflexi_bacterium_13_1_40CM_65_17	-	+	-
Sporisorium_scitamineum	-	+	-
Domibacillus_tundrae	-	+	-
Beauveria_bassiana	-	+	-
Megasphaera_elsdenii_CAG:570	-	+	-
Enterococcus_phage_phiSHEF5	-	+	-
Palaeococcus_ferrophilus	-	+	-
Paracoccidioides_brasiliensis	-	+	-
Lachnospiraceae_bacterium_OM02-31	-	+	-
Marinobacter_excellens	-	+	-
Paenibacillus_sp._D9	-	+	-

Sulfurospirillum_arcachonense	-	+	-
Mangrovibacter_plantisponsor	-	+	-
Streptomyces_sp._MUSC_125	-	+	-
Flavobacterium_sp._HTF	-	+	-
Candidatus_Gastranaerophilales_bacterium_HUM_1	+	+	-
Fusobacterium_sp._CAG:815	+	+	-
Chitiniphilus_shinanonensis	+	+	-
Paenibacillus_sp._P26E	+	+	-
Pseudopedobacter_saltans	+	+	-
Erysipelotrichaceae_bacterium_NYU-BL-F16	+	+	-
Paenibacillus_sp._7516	+	+	-
Paenibacillus_sp._UNC499MF	+	+	-
Methanospirillum_lacunae	+	+	-
Moritella_viscosa	+	+	-
Jejuia_pallidilutea	+	+	-
Streptomyces_sp._JV178	+	+	-
Pisolithus_tinctorius	+	+	-
Mucilaginibacter_sp._YBJ-36	+	+	-
Thermotoga_sp._50_64	+	+	-
Apibacter_sp._wkB309	+	+	-
Bifidobacterium_sp._2028B	+	+	-
Auricularia_subglabra	+	+	-
Anaerococcus_provencensis	+	+	-
Planococcus_salinus	+	+	-
Methanobacterium_sp._PtaB.Bin024	+	+	-
bacterium_YEK0313	+	+	-
Rhodospirillaceae_bacterium_SYSU_D60006	+	+	-
Coniella_lustricola	+	+	-
Fusobacteria_bacterium	+	+	-
Shewanella_baltica	+	+	-
Thermodesulfovibrio_yellowstonii	+	+	-
Hugenholtzia_roseola	-	+	-
Neisseria_sp._HMSC068C04	-	+	-
Desulfonauticus_sp._38_4375	-	+	-
Candidatus_Woesebacteria_bacterium_GW2011_GWB1_39_12	-	+	-
Aeribacillus_pallidus	-	+	-
Dokdonia_sp._Dokd-P16	-	+	-
Rhizophagus_sp._MUCL_43196	-	+	-
Pedobacter_sp._HMWF019	-	+	-
Wickerhamomyces_ciferrii	-	+	-
Bacillus_sp._JCM_19047	-	+	-
Streptococcus_hyovaginalis	-	+	-
Lactobacillus_futsaii	-	+	-
Mangrovibacter_phragmitis	-	+	-
Desulfobacteraceae_bacterium_4572_87	-	+	-
Nitrospirae_bacterium_RIFCSPLOW2_12_42_9	-	+	-
Planctomycetes_bacterium_RBG_16_41_13	-	+	-
Dietzia_sp._UCD-THP	-	+	-
Filimonas_sp.	-	+	-
uncultured_bacterium_Contig1495	-	+	-
Myroides_xuanwuensis	-	+	-
Opitutus_terrae	-	+	-
Enterococcus_sp._7F3_DIV0205	-	+	-
Carnobacterium_gallinarum	-	+	-
Paeniglutamicibacter_antarcticus	-	+	-

Dyella_sp._L4-6	-	+	-
Pseudomonas_vranovensis	-	+	-
Acidithiobacillus_caldus	-	+	-
bacterium_JKG1	-	+	-
Flavobacterium_cupreum	-	+	-
Pseudovibrio_stylochi	-	+	-
Candidatus_Omnitrophica_bacterium_CG1_02_49_16	-	+	-
Flavobacterium_beibuense	-	+	-
Vibrio_cidicii	-	+	-
Stachybotrys_chartarum	-	+	-
Collinsella_sp._AF28-5AC	-	+	-
Streptomyces_lavendulae	-	+	-
Lewinella_sp._4G2	-	+	-
Alicyclobacillus_acidocaldarius	-	+	-
Citrobacter_werkmanii	-	+	-
Candidatus_Gastranaerophilales_bacterium_HUM_4	+	+	-
Clostridium_sp._CAG:715	+	+	-
Methanocorpusculum_bavaricum	+	+	-
Furculomyces_boomerangus	+	+	-
Mycoplasma_arginini	+	+	-
Sphingobacteriales_bacterium_46-32	+	+	-
Legionella_pneumophila	+	+	-
Mucilaginibacter_paludis	+	+	-
Synergistales_bacterium_54_24	+	+	-
Paenibacillus_sp._HGF5	+	+	-
Vibrio_coralliilyticus	+	+	-
Bacillus_sp._AFS002410	+	+	-
Sphingobacteriaceae_bacterium_GW460-11-11-14-LB5	+	+	-
candidate_division_CPR3_bacterium_4484_211	+	+	-
Brochothrix_campestris	+	+	-
Haloterrigena_thermotolerans	+	+	-
Paenibacillus_sp._FSL_R5-192	+	+	-
Chryseobacterium_nakagawai	+	+	-
Candidatus_Riflebacteria_bacterium_HGW-Riflebacteria-1	+	+	-
Marinobacter_sp._es.048	+	+	-
Caldicellulosiruptor_owensensis	+	+	-
Streptococcus_minor	+	+	-
Collinsella_sp._OM08-14AT	+	+	-
Duganella_sp._Root1480D1	+	+	-
Campylobacter_avium	+	+	-
Variovorax_sp.	+	+	-
Streptomyces_formicae	+	+	-
Coprobacillus_sp._AM23-2	+	+	-
uncultured_Mediterranean_phage	+	+	-
Streptomyces_chartreusis	+	+	-
Anaerococcus_sp._Marseille-P3915	+	+	-
Virgibacillus_massiliensis	+	+	-
Thermoanaerobacter_wiegelii	+	+	-
Bacteroidetes_bacterium_43-16	+	+	-
Chlorobium_limicola	+	+	-
Bradyrhizobiaceae_bacterium_SG-6C	+	+	-
Acidithiobacillales_bacterium_SM23_46	+	+	-
Actinobacteria_bacterium_HGW-Actinobacteria-2	+	+	-
Pseudomonas_sp._Irchel_3F6	+	+	-
Mycoplasma_canis	+	+	-

Streptococcus_hyointestinalis	+	+	-
Hymenobacter_sp._DG25A	+	+	-
Rhizopus_stolonifer	+	+	-
Oenococcus_kitaharae	+	+	-
Desulfurobacterium_thermolithotrophum	+	+	-
Avian_sarcoma_virus	+	+	-
Thermodesulfobium_narugense	+	+	-
Veillonella_montpellierensis	+	+	-
Hortaea_werneckii	+	+	-
Bradyrhizobium_ottawaense	+	+	-
Massilia_sp._NR_4-1	+	+	-
ANME-2_cluster_archaeon	+	+	-
uncultured_bacterium_Contig4	+	+	-
Verruconis_gallopava	+	+	-
Rhodopirellula_sallentina	+	+	-
Kockovaella_imperatae	+	+	-
Candidatus_Nitrotoga_fabula	+	+	-
Bacteroides_sp._AF32-15BH	-	+	-
unidentified_virus	-	+	-
Candidatus_Yanofskybacteria_bacterium_GW2011_GWC2_41_	-	+	-
Flavobacteriaceae_bacterium_A100	-	+	-
Serratia_sp._DD3	-	+	-
Thermodesulfovibrio_sp._RBG_19FT_COMBO_42_12	-	+	-
Moraxella_equi	-	+	-
Ignavibacteria_bacterium_RIFOXYA12_FULL_38_9	-	+	-
Sphaerobolus_stellatus	-	+	-
Diadromus_pulchellus_toursvirus	-	+	-
Cylindrobasidium_torrendii	-	+	-
Halomonas_heilongjiangensis	-	+	-
Colletotrichum_graminicola	-	+	-
Sporidiobolus_salmonicolor	-	+	-
Stereum_hirsutum	-	+	-
Cyanothece_sp._PCC_7424	-	+	-
Enterobacter_mori	-	+	-
Microsporum_canis	-	+	-
Rhodospirillaceae_bacterium_TMED256	-	+	-
Pseudomonas_sp._MWU13-2860	-	+	-
Y73_sarcoma_virus	-	+	-
Nesterenkonia_sp._M8	-	+	-
Methanobacterium_sp._BAmetb5	-	+	-
Smittium_culicis	-	+	-
Acidobacteria_bacterium_13_1_40CM_4_58_4	-	+	-
Rachicladosporium_antarcticum	-	+	-
Streptomyces_wuyuanensis	-	+	-
Endozoicomonas_numazuensis	-	+	-
Morchella_conica	-	+	-
Leucobacter_chromiiresistens	-	+	-
Bordetella_genomosp._7	-	+	-
Emticicia_sp._MM	-	+	-
Paenibacillus_sp._E194	-	+	-
Pseudomonas_sp._RL	-	+	-
Cyanobacteria_bacterium_TMED229	-	+	-
Paenibacillus_sp._cl141a	-	+	-
Amycolatopsis_decaplanina	-	+	-
Paenibacillus_sp._Y412MC10	-	+	-

Estrella_lausannensis	-	+	-
Candidatus_Daviesbacteria_bacterium_GW2011_GWC1_40_9	-	+	-
Testicularia_cyperi	-	+	-
Thaumasiovibrio_occultus	-	+	-
Pelistega_indica	-	+	-
Pseudoclavibacter_bifida	-	+	-
Azorhizobium_doebereinerae	-	+	-
Enterococcus_phage_IME-EF4	-	+	-
Thermodesulfobium_acidiphilum	-	+	-
Ensifer_sp._Root31	-	+	-
Deltaproteobacteria_bacterium_RIFOXYB12_FULL_58_9	-	+	-
Bradymonadales_bacterium_B210	-	+	-
Phaeobacter_inhibens	-	+	-
Coraliomargarita_sp._WN38	-	+	-
Candidatus_Liptonbacteria_bacterium_RIFCSPLOWO2_01_FU	-	+	-
Penicillium_steckii	-	+	-
Vibrio_hyugaensis	-	+	-
Acholeplasma_multilocale	-	+	-
Cellvibrionales_bacterium_TMED157	-	+	-
Elusimicrobia_bacterium_CG06_land_8_20_14_3_00_38_11	-	+	-
Chelonid_alphaherpesvirus_5	-	+	-
Mixia_osmundae	-	+	-
Herbaspirillum_sp._3R-3a1	-	+	-
Alkalispirochaeta_sphaeroplastigenens	-	+	-
Volepox_virus	-	+	-
Bacteroidetes_bacterium_TMED284	-	+	-
Myxoma_virus	-	+	-
Silicimonas_algicola	-	+	-
Saccharospirillum_sp._MSK14-1	-	+	-
Glycomyces_sambucus	-	+	-
Alicyclobacillus_herbarius	-	+	-
Halanaerobium_sp._MSAO_Bac5	-	+	-
Streptococcus_sp._GMD2S	-	+	-
Elaphomyces_granulatus	-	+	-
Rhizobium_vallis	-	+	-
candidate_division_Kazan_bacterium_GW2011_GWC1_52_13	-	+	-
Obesumbacterium_proteus	-	+	-
Candidatus_Staskawiczbacteria_bacterium_RIFCSPLOWO2_01	-	+	-
Omnitrophica_WOR_2_bacterium_RIFCSPHIGHO2_01_FULL	-	+	-
Methanocaldococcus_infernus	-	+	-
Bifidobacterium_aesculapii	-	+	-
Rhodobacteraceae_bacterium_WFHF2C18	-	+	-
Actinokineospora_spheciospongiae	-	+	-
candidate_division_MSBL1_archaeon_SCGC-AAA259E19	-	+	-
Rhodoferax_koreense	-	+	-
Planococcus_sp._CAU13	+	+	-
Mesoaciditoga_sp.	+	+	-
Mesorhizobium_loti	+	+	-
Piscibacillus_halophilus	+	+	-
Ferrimonas_marina	+	+	-
Salirhabdus_sp._Marseille-P4669	+	+	-
Ketobacter_alkanivorans	+	+	-
Orbus_hercynius	+	+	-
Tolypothrix_bouteillei	+	+	-
Fervidobacterium_gondwanense	+	+	-

Maribacter_sp._T28	+	+	-
Kangiella_koreensis	+	+	-
Methanomassiliicoccales_archaeon_RumEn_M1	+	+	-
Acidaminococcus_sp._AM33-14BH	+	+	-
Veillonella_sp._S13053-19	+	+	-
Collinsella_sp._AF18-8LB	+	+	-
Blastocatellia_bacterium	+	+	-
Caballeronia_sordidicola	+	+	-
Streptococcus_infantarius	+	+	-
Streptomyces_sp._LcepLS	+	+	-
uncultured_bacterium_Contig783	+	+	-
Nitrospirillum_amazonense	+	+	-
Candidatus_Levybacteria_bacterium_RIFCSPHIGHO2_01_FUL	+	+	-
Lactobacillus_sp._256-3	+	+	-
Siphonobacter_sp._BAB-5385	+	+	-
Sphingobacterium_multivorum	+	+	-
Planifilum_fulgidum	+	+	-
blood_disease_bacterium_R229	+	+	-
Streptococcus_sp._'caviae'	+	+	-
Oceanobacillus_sojae	+	+	-
Actinomyces_slackii	+	+	-
Petrotoga_mobilis	+	+	-
uncultured_bacterium_fosmid_pJB23D10	+	+	-
Veillonella_sp._DORA_A_3_16_22	+	+	-
Aspergillus_novofumigatus	+	+	-
Beijerinckiaceae_bacterium	+	+	-
Gloeophyllum_trabeum	+	+	-
Bacillus_sp._AFS017274	+	+	-
Thermosipho_globiformans	+	+	-
Bacillus_sp._K6W	+	+	-
Bacillus_aurantiacus	+	+	-
Pseudomonas_virus_EL	+	+	-
Mycobacterium_sp._EPa45	+	+	-
Bifidobacterium_cuniculi	+	+	-
Bifidobacterium_moukalabense	+	+	-
Acinetobacter_idrijaensis	+	+	-
Treponema_sp._RIFOXYC1_FULL_61_9	+	+	-
Malassezia_restricta	+	+	-
Ascobolus_immersus	+	+	-
Endozoicomonas_elysicola	+	+	-
[Mannheimia]_succiniciproducens	+	+	-
Enterobacter_sichuanensis	+	+	-
Paraburkholderia_sp._DHOM06	+	+	-
Micromonospora_citrea	+	+	-
Lewinella_xylanilytica	+	+	-
Streptomyces_achromogenes	+	+	-
Komagataella_phaffii	+	+	-
Alphaproteobacteria_bacterium_MarineAlpha12_Bin1	+	+	-
Helicobacter_mesocricetorum	+	+	-
Mesorhizobium_sp._M00.F.Ca.ET.216.01.1.1	-	+	-
bacterium_BMS3Abin10	-	+	-
Algoriphagus_ornithinivorans	-	+	-
Chryseobacterium_aurantiacum	-	+	-
Streptohalobacillus_salinus	-	+	-
Aquimarina_sediminis	-	+	-

Neisseria_subflava	-	+	-
Alcanivorax_indicus	-	+	-
Pediococcus_claussenii	-	+	-
Exiguobacterium_acetylicum	-	+	-
Rhodotorula_toruloides	-	+	-
Millerozyma_farinosa	-	+	-
Saccharomycodes_ludwigii	-	+	-
Obba_rivulosa	-	+	-
Collinsella_sp._AM15-2	-	+	-
Aspergillus_flavus	-	+	-
Enterococcus_sp._12C11_DIV0727	-	+	-
Porphyromonadaceae_bacterium_KA00676	-	+	-
Bdellovibrio_sp._qaytius	-	+	-
Extensimonas_vulgaris	-	+	-
Planctomycetes_bacterium_GWA2_50_13	-	+	-
Polaribacter_porphyrae	-	+	-
Planococcus_sp._PAMC_21323	-	+	-
Bacillus_sp._17376	-	+	-
Enterococcus_sp._RIT-PI-f	-	+	-
Methanocella_conradii	-	+	-
Chryseobacterium_flavum	-	+	-
Bacteroidetes_bacterium_HGW-Bacteroidetes-13	-	+	-
Kosmotoga_olearia	-	+	-
Marichromatium_gracile	-	+	-
Pyronema_omphalodes	-	+	-
Paraburkholderia_sp._UYCPa14C	-	+	-
Nonlabens_sp._MJ115	-	+	-
Paraburkholderia_caribensis	-	+	-
Granulicella_pectinivorans	-	+	-
Aspergillus_niger	-	+	-
Bacillus_sp._FJAT-27445	-	+	-
Clostridium_sp._DSM_18688	-	+	-
Torrubiella_hemipterigena	-	+	-
Truepera_radiovictrix	-	+	-
Avian_myelocytomatosis_virus	-	+	-
candidate_division_WPS-2_bacterium	-	+	-
Byssochlamys_spectabilis	-	+	-
Abelson_murine_leukemia_virus	-	+	-
Sphingobacterium_sp._1.A.5	-	+	-
Porphyromonas_sp._oral_taxon_278	-	+	-
Actinomadura_macra	-	+	-
Saccharospirillum_impatiens	-	+	-
Streptomyces_sp._NWU339	-	+	-
uncultured_bacterium_Contig575	-	+	-
Aigarchaeota_archaeon_NZ13_MG1	-	+	-
Streptomyces_sp._ST1020	-	+	-
Bifidobacterium_subtile	-	+	-
Streptosporangium_subroseum	-	+	-
Streptomyces_rubellomurinus	-	+	-
Sphingobacterium_sp._B29	-	+	-
Thermosynechococcus_elongatus	-	+	-
uncultured_bacterium_Contig1767	-	+	-
Pseudomonas_sp._DY-1	-	+	-
Pseudomonas_knackmussii	-	+	-
Adhaeribacter_sp._HMF7605	-	+	-

Tessaracoccus_massiliensis	-	+	-
Weissella_ceti	-	+	-
Terriglobus_saanensis	-	+	-
Rickettsia_prowazekii	-	+	-
Brevibacillus_phage_Emery	-	+	-
Amycolatopsis_antarctica	-	+	-
Pseudomonas_cichorii	-	+	-
Pseudomonas_sp._GR_6-02	-	+	-
Bacillus_sp._Leaf406	-	+	-
Bacillus_sp._LNXM12-2	-	+	-
Pseudomonas_sp._Irchel_s3f19	-	+	-
Xanthomonas_maliensis	-	+	-
Sulfurovum_sp._35-42-20	-	+	-
Anaerolineae_bacterium_CG2_30_57_67	-	+	-
Bacillus_sp._AFS033286	-	+	-
Proteobacteria_bacterium_SG_bin4	-	+	-
Ruminococcus_sp._OF05-2BH	-	+	-
Anaeromyxobacter_sp._PSR-1	-	+	-
Acidimicrobium_ferrooxidans	-	+	-
Pseudoflavitalea_rhizosphaerae	-	+	-
Pseudogymnoascus_sp._VKM_F-4520_(FW-2644)	-	+	-
Bacillus_fumarioli	-	+	-
Pseudocercospora_fijiensis	-	+	-
Ignavibacteriae_bacterium_HGW-Ignavibacteriae-2	-	+	-
Campylobacter_pinnipediorum	-	+	-
Croceicoccus_sp._H4	-	+	-
Ktedonobacterales_bacterium_Uno16	-	+	-
Cupriavidus_sp._WS	-	+	-
Curtobacterium_ammoniigenes	-	+	-
Kangiella_aquimarina	-	+	-
Haloplanus_vescus	-	+	-
Paraburkholderia_nodosa	-	+	-
Halomonas_sp._MES3-P3E	-	+	-
Candidatus_Magasanikbacteria_bacterium_CG10_big_fil_rev_8	-	+	-
Candidatus_Kerfeldbacteria_bacterium_CG08_land_8_20_14_0	-	+	-
Desulfobulbus_propionicus	-	+	-
Deinococcus_proteolyticus	-	+	-
Janthinobacterium_sp._Marseille	-	+	-
Iodobacter_sp._H11R3	-	+	-
Paenibacillus_sp._AD87	-	+	-
Ignavibacteriales_bacterium_UTCHB3	-	+	-
Candidatus_Moanabacter_tarae	-	+	-
Methylococcaceae_bacterium	-	+	-
Myxococcus_xanthus	-	+	-
Methanothermus_fervidus	-	+	-
Chloroflexi_bacterium_HGW-Chloroflexi-7	-	+	-
Chloroflexales_bacterium_ZM16-3	-	+	-
Microbacterium_sp._Root61	-	+	-
Muricauda_sp._CP2A	-	+	-
Chryseobacterium_sp._HMWF001	-	+	-
Citrobacter_pasteurii	-	+	-
Chryseobacterium_sp._5_R23647	-	+	-
Chryseobacterium_hungaricum	-	+	-
Nocardia_takedensis	-	+	-
Lysobacter_capsici	-	+	-

Melaminivora_alkalimesophila	-	+	-
Marinomonas_polaris	-	+	-
Macrococcus_sp._DPC7161	-	+	-
Marinobacter_daepoensis	-	+	-
Marinitoga_hydrogenitolerans	-	+	-
Gaetbulibacter_sp._4G1	-	+	-
Fusarium_verticillioides	-	+	-
Elusimicrobia_bacterium_RIFCSPHIGHO2_01_FULL_64_10	-	+	-
Flavobacterium_sp._YO64	-	+	-
Gallionellales_bacterium_RIFCSPHIGHO2_02_FULL_57_16	-	+	-
Parcubacteria_group_bacterium_GW2011_GWA2_45_14	-	+	-
Parcubacteria_group_bacterium_GW2011_GWA2_51_12	-	+	-
Parcubacteria_group_bacterium_GW2011_GWF2_44_8	-	+	-
Dyadobacter_beijingensis	-	+	-
Candidatus_Collierbacteria_bacterium_GW2011_GWC2_43_12	-	+	-
Candidatus_Collierbacteria_bacterium_GW2011_GWB2_42_12	-	+	-
Photobacterium_galatheae	-	+	-
Fervidobacterium_thailandense	-	+	-
Erwinia_gerundensis	-	+	-
Enterococcus_phage_EFRM31	-	+	-
Petrotoga_sp._9PW.55.5.1	-	+	-
Granulicatella_sp._HMSC30F09	-	+	-
Parcubacteria_group_bacterium_CG_4_10_14_0_2_um_filter_4	-	+	-
Candidatus_Jidaibacter_acanthamoeba	-	+	-
Candidatus_Harrisonbacteria_bacterium_CG10_big_fil_rev_8_2	-	+	-
Empedobacter_brevis	+	+	-
Exiguobacterium_sp._N4-1P	+	+	-
Psychroserpens_mesophilus	+	+	-
Cylindrospermum_stagnale	+	+	-
Sutterella_sp._CAG:351	+	+	-
Treponema_caldarium	+	+	-
Candidatus_Desulforudis_sp.	+	+	-
Streptococcus_intermedius	+	+	-
Bifidobacterium_choerinum	+	+	-
Collinsella_sp._MS5	+	+	-
Actinophytocola_xinjiangensis	+	+	-
Paraliobacillus_quinghaiensis	+	+	-
Parcubacteria_group_bacterium_GW2011_GWC2_44_22	+	+	-
Bacillus_massiliglaciei	+	+	-
Paenibacillus_sp._P32E	+	+	-
Pseudomonas_sp._AU12215	+	+	-
Lactobacillus_hamsteri	+	+	-
Pedobacter_tournemirensis	+	+	-
Fusarium_oxysporum	+	+	-
Dehalogenimonas_sp._GP	+	+	-
Clostridium_sp._NCR	+	+	-
Bacillus_sp._Aph1	+	+	-
Mucilaginibacter_sp._OK283	+	+	-
Betaproteobacteria_bacterium_HGW-Betaproteobacteria-17	+	+	-
Lactobacillus_fabifermentans	+	+	-
Brevibacillus_sp._Leaf182	+	+	-
Sterolibacteriaceae_bacterium_J5B	+	+	-
Paraburkholderia_oxyphila	+	+	-
Defluviitoga_tunisiensis	+	+	-
Bifidobacterium_sp._56_9_plus	+	+	-

Balneola_sp._EhC07	+	+	-
Cellulomonas_flavigena	+	+	-
Spirochaetae_bacterium_HGW-Spirochaetae-5	+	+	-
Phycisphaera_sp.	+	+	-
Telmatospirillum_siberiense	+	+	-
Campylobacter_sp._RM8966	+	+	-
Peptococcaceae_bacterium_SCADC1_2_3	+	+	-
Lactobacillus_kimchiensis	+	+	-
Bacteroidetes_bacterium_GWA2_31_9b	+	+	-
Conexibacter_woesei	+	+	-
Kaistia_algarum	+	+	-
Azonexus_fungiphilus	+	+	-
Acidobacteria_bacterium_RIFCSPLOWO2_02_FULL_59_13	+	+	-
Helicobacter_anseris	+	+	-
Streptomyces_albus	+	+	-
Candidatus_Woesebacteria_bacterium_RIFCSPHIGHO2_01_FU	+	+	-
Lactobacillus_musae	+	+	-
Oceanobacillus_chungangensis	+	+	-
Sphingomonas_sp.	+	+	-
Rhodocyclaceae_bacterium	+	+	-
Tuberibacillus_sp._Marseille-P3662	+	+	-
Polaribacter_atrinae	+	+	-
Chryseobacterium_oranimense	+	+	-
Frankia_elaeagni	+	+	-
Rhodocyclales_bacterium_GWA2_65_19	+	+	-
Lentibacillus_jeotgali	+	+	-
Acidimicrobiales_bacterium	+	+	-
Pontibacter_indicus	+	+	-
Virgibacillus_dakarensis	+	+	-
Lodderomyces_elongisporus	+	+	-
Neocallimastix_cameroonii	+	+	-
Chlorobium_phaeovibrioides	+	+	-
Trichoderma_virens	+	+	-
Lactobacillus_mali	+	+	-
Magnetospira_sp._QH-2	+	+	-
Chitinophaga_sancti	+	+	-
Planomicrobium_sp._Y74	+	+	-
Vibrio_penaeicida	+	+	-
Candidatus_Amesbacteria_bacterium_RIFOXYB1_FULL_44_2	+	+	-
Candidatus_Micrarchaeota_archaeon_CG1_02_47_40	+	+	-
Lactobacillus_rennini	+	+	-
Babjeviella_inositovora	+	+	-
Collinsella_sp._OF03-4AA	+	+	-
Cyclobacterium_lianum	+	+	-
Bacillus_sp._V3-13	+	+	-
Nocardiopsis_sp._Huas11	+	+	-
Neisseria_bacilliformis	+	+	-
Methylacidiphilum_infernorum	+	+	-
Candidatus_Schmidhempelia_bombi	+	+	-
Saccharibacillus_kuerlensis	+	+	-
Desulfobulbaceae_bacterium_S5133MH15	+	+	-
Desulfobacterales_bacterium_S5133MH4	+	+	-
Achromobacter_sp._Root170	-	+	-
Murine_leukemia-related_retroviruses	-	+	-
Rhodospirillaceae_bacterium_Spongia-Bin9	-	+	-

Herbaspirillum_sp._XFZ15_10_5	-	+	-
Amphritea_japonica	-	+	-
Bifidobacterium_aquikefiri	-	+	-
Alphaproteobacteria_bacterium_TMED89	-	+	-
Smittium_megazygosporum	-	+	-
Jaminaea_rosea	-	+	-
Leptospira_sp._YH101	-	+	-
Methanobacterium_sp._BRmetb2	-	+	-
Kyrpidia_spormannii	-	+	-
Staphylococcus_felis	-	+	-
Mesonia_algae	-	+	-
candidate_division_Zixibacteria_bacterium_SM23_73_3	-	+	-
Candidatus_Glassbacteria_bacterium_RIFCSPLOWO2_12_FUL	-	+	-
Algoriphagus_aquaeductus	-	+	-
Planktothrix_serta	-	+	-
Alphaproteobacteria_bacterium_13_2_20CM_2_64_7	-	+	-
Georgenia_sp._ZLJ0423	-	+	-
Nostoc_linckia	-	+	-
Thalassospira_sp._MCCC_1A02898	-	+	-
Martelella_endophytica	-	+	-
Bacillus_sp._FJAT-46582	-	+	-
Tetragenococcus_solitarius	-	+	-
Verrucosispora_sediminis	-	+	-
Epulopiscium_sp._Nele67-Bin002	-	+	-
Flavobacterium_urumqiense	-	+	-
Paracoccus_sp._Arc7-R13	-	+	-
Acidobacteriaceae_bacterium_KBS_89	-	+	-
Alkalibacterium_sp._20	-	+	-
Chryseobacterium_luteum	-	+	-
Photobacterium_frigidiphilum	-	+	-
Agromyces_sp._FW100M-8	-	+	-
Alphaproteobacteria_bacterium_MarineAlpha6_Bin5	-	+	-
Geobacillus_thermodenitrificans	-	+	-
Bacillus_urumqiensis	-	+	-
Acidobacteria_bacterium_RIFCSPLOWO2_02_FULL_64_15	-	+	-
Deltaproteobacteria_bacterium_HGW-Deltaproteobacteria-13	-	+	-
Deltaproteobacteria_bacterium_RBG_16_71_12	-	+	-
Verrucomicrobium_sp._GAS474	-	+	-
Listeria_sp._SHR_NRA_18	-	+	-
Candidatus_Synechococcus_spongiarum	-	+	-
Mycoplasma_orale	-	+	-
Acidihalobacter_ferrooxidans	-	+	-
Endozoicomonas_atrinae	-	+	-
Candidatus_Uhrbacteria_bacterium_RIFCSPLOWO2_02_FULL	-	+	-
Beutenbergia_cavernae	-	+	-
Leifsonia_sp._OV430	-	+	-
Herbidospora_sakaeratensis	-	+	-
Anaeromyxobacter_sp._Fw109-5	-	+	-
Desulfatiglans_anilini	-	+	-
Alteromonas_sp._TMED35	-	+	-
Indivirus_ILV1	-	+	-
Ruminococcus_sp._AF25-3LB	-	+	-
Halomonas_sp._PBN3	-	+	-
Runella_limosa	-	+	-
Rhodotorula_sp._JG-1b	-	+	-

Sphingomonas_crusticola	-	+	-
Agrobacterium_vitis	-	+	-
Streptococcus_thoraltensis	-	+	-
Metarhizium_robertsii	-	+	-
Enterococcus_phage_phiSHEF2	-	+	-
Zunongwangia_profunda	-	+	-
Enterococcus_phage_EfaCPT1	-	+	-
Enterococcus_phage_SANTOR1	-	+	-
Flavobacterium_cucumis	-	+	-
delta_proteobacterium_PSCGC_5296	-	+	-
Flavobacterium_denitrificans	-	+	-
Hymenobacter_terrenus	-	+	-
Streptomyces_himastatinicus	-	+	-
Melissococcus_sp._OM08-11BH	-	+	-
Mesorhizobium_sp._LSHC420B00	-	+	-
Streptomyces_sp._AM-2504	-	+	-
Streptomyces_showdoensis	-	+	-
Hanstruepera_neustonica	-	+	-
Acinetobacter_soli	-	+	-
Synechococcus_sp._PCC_6312	-	+	-
Acinetobacter_sp._ACNIH2	-	+	-
Syntrophobacteraceae_bacterium_CG07_land_8_20_14_0_80_6	-	+	-
Listeria_innocua	-	+	-
Leptospira_yanagawae	-	+	-
Syntrophus_sp._PtaU1.Bin208	-	+	-
Talaromyces_verruculosus	-	+	-
Tardiphaga_robiniae	-	+	-
Lentzea_kentuckyensis	-	+	-
Encephalitozoon_intestinalis	-	+	-
Halopelagius_inordinatus	-	+	-
Sulfobacillus_acidophilus	-	+	-
Luteibacter_sp._UNCMF366Tsu5.1	-	+	-
Algoriphagus_halophilus	-	+	-
Flavobacterium_sp._BFFFF2	-	+	-
Lysobacter_antibioticus	-	+	-
Facklamia_sp._Marseille-P5643	-	+	-
Moraxella_lacunata	-	+	-
Moraxella_bovoculi	-	+	-
Desulfovibrio_sp._S3730MH75	-	+	-
Sporosarcina_sp._P29	-	+	-
Micromonospora_krabiensis	-	+	-
Staphylococcus_kloosii	-	+	-
Staphylococcus_sciuri	-	+	-
Motiliproteus_coralliicola	-	+	-
Motiliproteus_sp._MSK22-1	-	+	-
Halobacillus_mangrovi	-	+	-
Yokapox_virus	-	+	-
Muricauda_sp._MAR_2010_75	-	+	-
uncultured_soil_bacterium	-	+	-
Spirochaetes_bacterium_GWF2_52_7	-	+	-
Exiguobacterium_sp._AB2	-	+	-
Microcella_putealis	-	+	-
Microbulbifer_sp._Q7	-	+	-
Filimonas_lacunae	-	+	-
Filimonas_sp._TTM-71	-	+	-

Achromobacter_sp._2789STDY5608625	-	+	-
Methanothermobacter_wolfeii	-	+	-
Methanosphaera_sp._WGK6	-	+	-
Streptococcus_troglodytae	-	+	-
uncultured_Termite_group_1_bacterium	-	+	-
Streptococcus_virus_MS1	-	+	-
Methanohalobium_evestigatum	-	+	-
Streptomyces_albulus	-	+	-
Clostridium_sp._AF12-28	-	+	-
Fervidicoccus_sp.	-	+	-
Yellowstone_lake_phycodnavirus_2	-	+	-
Halobacteriales_archaeon_QS_4_69_31	-	+	-
Fervidobacterium_nodosum	-	+	-
Microbacterium_humi	-	+	-
Haladaptatus_sp._R4	-	+	-
Halobacteriales_archaeon_SW_12_69_24	-	+	-
uncultured_bacterium_Contig1625	-	+	-
Xylaria_longipes	-	+	-
Legionella_sp._km772	-	+	-
Kocuria_sp._257	-	+	-
Kluyveromyces_dobzhanskii	-	+	-
Tropicimonas_sediminicola	-	+	-
Vagococcus_sp._SS1995	-	+	-
archaeon_GW2011_AR3	-	+	-
Kocuria_sp._WN036	-	+	-
Gordonia_sp._1D	-	+	-
Aquaspirillum_sp._LM1	-	+	-
Cupriavidus_pauculus	-	+	-
Tilletia_indica	-	+	-
Cutaneotrichosporon_oleaginosum	-	+	-
Dermabacter_sp._HMSC06F07	-	+	-
Ignavibacteria_bacterium_CG22_combo_CG10-13_8_21_14_al	-	+	-
Ignavibacteria_bacterium_13_1_40CM_2_61_4	-	+	-
Vibrio_sp._2016V-1018	-	+	-
Ignatzschineria_cameli	-	+	-
Amanita_muscaria	-	+	-
Alteromonas_sp._U0105	-	+	-
Veillonella_sp._6_1_27	-	+	-
Invertebrate_iridescent_virus_6	-	+	-
Aeromonas_dhakensis	-	+	-
Alphaproteobacteria_bacterium_WS11	-	+	-
Deltaproteobacteria_bacterium_CG2_30_63_29	-	+	-
Verrucomicrobia_subdivision_3_bacterium	-	+	-
bacterium_BMS3Bbin12	-	+	-
bacterium_BMS3Bbin10	-	+	-
Deltaproteobacteria_bacterium_HGW-Deltaproteobacteria-20	-	+	-
Jaapia_argillacea	-	+	-
Deltaproteobacteria_bacterium_RBG_19FT_COMBO_46_9	-	+	-
Vibrio_mediterranei	-	+	-
Dichomitus_squalens	-	+	-
Theionarchaea_archaeon_DG-70	-	+	-
Collinsella_sp._AM36-4AA	-	+	-
Elusimicrobia_bacterium_CG08_land_8_20_14_0_20_51_18	-	+	-
Lactobacillus_sp._ZW163	-	+	-
Aspergillus_terreus	-	+	-

Actinobacteria_bacterium_RBG_16_67_15	-	+	-
Avian_musculoaponeurotic_fibrosarcoma_virus_AS42	-	+	-
Collinsella_sp._AF16-8	-	+	-
Elusimicrobia_bacterium_RIFCSPLOWO2_12_FULL_59_9	-	+	-
Collinsella_sp._AF37-9	-	+	-
Aspergillus_lacticoffeatus	-	+	-
Virgibacillus_sp._Bac332	-	+	-
Arcobacter_sp._RW43-9	-	+	-
Corynebacterium_cystitidis	-	+	-
Lactobacillus_silagei	-	+	-
Actinomyces_graevenitzii	-	+	-
Coprobacillus_sp._CAG:183	-	+	-
Edaphobacillus_lindanitolerans	-	+	-
candidate_division_KSB1_bacterium_4484_188	-	+	-
Ecterococcus_phage_vB_EfaS_AL3	-	+	-
Humitalea_rosea	-	+	-
Bacteroidetes_bacterium_OLB12	-	+	-
Bacteroidetes_bacterium_OLB10	-	+	-
Candidatus_Gracilibacteria_bacterium_GN02-873	-	+	-
Catenibacterium_sp._AM22-6LB	-	+	-
Rodentibacter_heylii	-	+	-
Pseudogymnoascus_sp._VKM_F-4515_(FW-2607)	-	+	-
Candidatus_Giovannonibacteria_bacterium_GW2011_GWB1_4	-	+	-
Candidatus_Kentron_sp._H	-	+	-
Capnocytophaga_sp.	-	+	-
Rhodospirillales_bacterium_70-18	-	+	-
Parahaliea_mediterranea	-	+	-
Candidatus_Giovannonibacteria_bacterium_GW2011_GWB1_4	-	+	-
Candidatus_Kentron_sp._LPFa	-	+	-
Chicken_stool-associated_circular_virus	-	+	-
Nissabacter_archeti	-	+	-
Rubidibacter_lacunae	-	+	-
Rufibacter_immobilis	-	+	-
Nitrosomonas_europaea	-	+	-
Salinibacter_sp._10B	-	+	-
Candidatus_Omnitrophica_bacterium_CG11_big_fil_rev_8_21_	-	+	-
Pseudomonas_xanthomarina	-	+	-
Rhizobiales_bacterium_TMED83	-	+	-
Psychroflexus_halocasei	-	+	-
Candidatus_Saccharibacteria_bacterium_CG11_big_fil_rev_8_2	-	+	-
Bradyrhizobium_sp._AS23.2	-	+	-
Paraburkholderia_sp._BL17N1	-	+	-
Candidatus_Woesebacteria_bacterium_GW2011_GWB1_41_10	-	+	-
Paracoccus_siganidrum	-	+	-
Candidatus_Tagabacteria_bacterium_RIFCSPLOWO2_01_FUL	-	+	-
Pseudomonas_sp._R4-35-07	-	+	-
Ralstonia_pseudosolanacearum	-	+	-
Chryseobacterium_soldanellicola	-	+	-
Mycoplasma_feriruminatoris	-	+	-
Planomicrobium_sp._MB-3u-38	-	+	-
Sphingobacterium_thalpophilum	-	+	-
Chromobacterium_sp._IIBBL_112-1	-	+	-
Sphingobium_chungbukense	-	+	-
Chromobacterium_sp._LK1	-	+	-
Sphingobacteriales_bacterium_50-39	-	+	-

Petrotoga_sp._HWH.PT.55.6.1	-	+	-
Caldiserica_bacterium_CG02_land_8_20_14_3_00_36_38	-	+	-
Chryseobacterium_joostei	-	+	-
Chromatocurvus_halotolerans	-	+	-
Phanerochaete_carnosa	-	+	-
Sphingobacterium_sp._G1-14	-	+	-
Candidatus_Chrysopegis_kryptomonas	-	+	-
Penicillium_camemberti	-	+	-
Serinicoccus_sp._JLT9	-	+	-
Sphaerisporangium_sp._LHW63015	-	+	-
Serratia_sp._Leaf50	-	+	-
Smittium_angustum	-	+	-
Bacteroides_sp._AF27-33	-	+	-
Penicilliopsis_zonata	-	+	-
Chlorobi_bacterium	-	+	-
Salinicoccus_sediminis	-	+	-
Bacteroides_propionicifaciens	+	+	-
Arenibacter_latericius	+	+	-
Parabacteroides_sp._CT06	+	+	-
Capnocytophaga_sp._oral_taxon_326	+	+	-
Lutibacter_sp._HS1-25	+	+	-
Methanosphaera_sp._SHI613	+	+	-
uncultured_bacterium_SRF2	+	+	-
Sulfurimonas_hongkongensis	+	+	-
Idiomarina_taiwanensis	+	+	-
Candidatus_Melainabacteria_bacterium_GWF2_32_7	+	+	-
Lactobacillus_ultunensis	+	+	-
Rodentibacter_rarus	+	+	-
Acanthamoeba_polyphaga_moumouvirus	+	+	-
Candidatus_Protochlamydia_amoebophila	+	+	-
Necropsobacter_massiliensis	+	+	-
Halolactibacillus_halophilus	+	+	-
Myxococcus_hansupus	+	+	-
Rhodospirillales_bacterium_RIFCSPLOWO2_02_FULL_58_16	+	+	-
Pelagibacterium_sp._SCN_64-44	+	+	-
Cycloclasticus_sp._symbiont_of_Poecilosclerida_sp._M	+	+	-
Bacillus_sinesaloumensis	+	+	-
Bacillus_alveayuensis	+	+	-
Enterococcus_thailandicus	+	+	-
Rhodanobacter_denitrificans	+	+	-
Litorilinea_aerophila	+	+	-
Algoriphagus_machipongonensis	+	+	-
Methanomicrobiales_archaeon_HGW-Methanomicrobiales-1	+	+	-
Lactobacillus_lindneri	+	+	-
Chlamydiae_bacterium	+	+	-
Spirochaetaceae_bacterium_4572_59	+	+	-
Bradyrhizobium_erythrophlei	+	+	-
Methanobacterium_sp.	+	+	-
Paraburkholderia_ginsengiterrae	+	+	-
Kosmotoga_arenicorallina	+	+	-
Pararhodospirillum_photometricum	+	+	-
Sphingobacterium_sp._PM2-P1-29	+	+	-
Rhodospirillaceae_bacterium_BRH_c57	+	+	-
Lipomyces_starkeyi	+	+	-
Piromyces_sp.	+	+	-

Collinsella_sp._AF38-3AC	+	+	-
Candidatus_Symbiobacter_mobilis	+	+	-
Phaeomoniella_chlamydospora	+	+	-
Podospora_comata	+	+	-
Geobacillus_sp._44B	+	+	-
Cellvibrio_sp._pealriver	+	+	-
Candidatus_Falkowbacteria_bacterium_GW2011_GWF2_43_32	+	+	-
Candidatus_Ornithobacterium_hominis	+	+	-
Bacillaceae_bacterium_B16-10	+	+	-
Aspergillus_nidulans	+	+	-
Thermoactinomycetaceae_bacterium_SCSIO_07575	+	+	-
Methanosaeta_sp._PtaU1.Bin060	+	+	-
Kiloniella_sp._EL199	+	+	-
Candidatus_Wildermuthbacteria_bacterium_RIFCSPHIGHO2_0	+	+	-
bacterium_TMED15	+	+	-
Bdellovibrionales_bacterium_RIFOXYC1_FULL_54_43	+	+	-
Balneola_vulgaris	+	+	-
Acetomicrobium_mobile	+	+	-
Labrenzia_sp._OB1	+	+	-
Salinicoccus_sp._YB14-2	+	+	-
Lactobacillus_sp._218-6	+	+	-
Amantichitinum_ursilacus	+	+	-
Fontimonas_thermophila	+	+	-
Labilithrix_luteola	+	+	-
Malassezia_pachydermatis	+	+	-
Deinococcus_gobiensis	+	+	-
uncultured_bacterium_Contig1756	+	+	-
Deltaproteobacteria_bacterium_CG11_big_fil_rev_8_21_14_0_[illegible]	+	+	-
Vibrio_anguillarum	+	+	-
Isoptericola_variabilis	+	+	-
Firmicutes_bacterium_HGW-Firmicutes-6	+	+	-
Candidatus_Magasanikbacteria_bacterium_RIFOXYD2_FULL_	+	+	-
Chamaesiphon_polymorphus	+	+	-
Oceanivirga_salmonicida	+	+	-
Anncaliia_algerae	+	+	-
Desulfotignum_balticum	+	+	-
Stella_humosa	+	+	-
Gemmata_obscuriglobus	+	+	-
Candidatus_Entotheonella_gemina	+	+	-
Coniochaeta_pulveracea	+	+	-
Candidatus_Phycosocius_bacilliformis	+	+	-
Herpetosiphon_geysericola	+	+	-
Bacillus_sp._7520-S	+	+	-
Streptococcus_ruminantium	+	+	-
Bacteroidetes_bacterium_CG12_big_fil_rev_8_21_14_0_65_60	+	+	-
Sporomusa_ovata	+	+	-
Acinetobacter_lwoffii	+	+	-
Candidatus_Woesebacteria_bacterium_RIFOXYA1_FULL_40_	+	+	-
Methylovulum_sp.	+	+	-
Mycobacterium_sp._CECT_8779	+	+	-
Nitrospira_moscoviensis	+	+	-
Cyanobacteria_bacterium_QH_1_48_107	+	+	-
Flavobacterium_succinicans	+	+	-
Ezakiella_massiliensis	+	+	-
Mucilaginibacter_sp._RS1	+	+	-

Mucor_circinelloides	-	+	-
Aquimarina_longa	-	+	-
Parcubacteria_group_bacterium_GW2011_GWA2_40_143	-	+	-
Burkholderia_sp._WSM2232	-	+	-
Geomicrobium_sp._JCM_19037	-	+	-
Lactobacillus_sp._chh01	-	+	-
Kwoniella_pini	-	+	-
Bacteroides_sp._AM27-13	-	+	-
Ruminococcus_sp._AM07-21	-	+	-
Parcubacteria_group_bacterium_SW_4_49_11	-	+	-
Acidovorax_cavernicola	-	+	-
Candida_maltosa	-	+	-
Psychrobacter_aquaticus	-	+	-
Ohtaekwangia_koreensis	-	+	-
Erysipelothrix_sp._15TAL0474	-	+	-
Allochromatium_vinosum	-	+	-
Verrucomicrobia_bacterium_GWF2_62_7	-	+	-
Candidatus_Magasanikbacteria_bacterium_CG10_big_fil_rev_8	-	+	-
Bacillus_phage_PK16	-	+	-
Dissulfuribacter_thermophilus	-	+	-
Rhodopirellula_sp._TMED283	-	+	-
Dyella_sp._C11	-	+	-
Crenotalea_thermophila	-	+	-
Canarypox_virus	-	+	-
Streptomyces_sp._alain-838	-	+	-
Calothrix_sp._NIES-3974	-	+	-
uncultured_bacterium_Contig1753	-	+	-
Paenibacillus_sp._FSL_H8-457	-	+	-
Candidatus_Moranbacteria_bacterium_GW2011_GWF2_35_39	-	+	-
Psychrobacter_sp._JCM_18900	-	+	-
Wallemia_ichthyophaga	-	+	-
Bacillus_sp._FJAT-45086	-	+	-
Ascoidea_rubescens	-	+	-
Hypholoma_sublateritium	-	+	-
Methanospirillum_hungatei	-	+	-
Cryptococcus_amylolentus	-	+	-
Sphingobacterium_sp._JB170	-	+	-
Acinetobacter_rudis	-	+	-
Lewinella_marina	-	+	-
Variovorax_sp._HW608	-	+	-
Tulasnella_calospora	-	+	-
Lentisphaerae_bacterium_RIFOXYB12_FULL_60_10	-	+	-
Bacteroidetes_bacterium_GWF2_41_31	-	+	-
Geobacter_sulfurreducens	-	+	-
Meyerozyma_sp._JA9	-	+	-
Glarea_lozoyensis	-	+	-
Mucilaginibacter_sp._F01003	-	+	-
candidate_division_Kazan_bacterium	-	+	-
Novosphingobium_sp._P6W	-	+	-
Salinicola_peritrichatus	-	+	-
Rasamsonia_emersonii	-	+	-
Plesiocystis_pacifica	-	+	-
Alicyclobacillus_acidoterrestris	-	+	-
Alkalinema_sp._CACIAM_70d	-	+	-
Pseudomonas_virus_PA7	-	+	-

Vibrio_proteolyticus	-	+	-
Pseudogymnoascus_destructans	-	+	-
Verrucomicrobia_bacterium_RIFCSPLOWO2_12_FULL_64_8	-	+	-
Corynespora_cassiicola	-	+	-
Lactobacillus_senioris	-	+	-
Neisseria_dentiae	-	+	-
Nitrospinae_bacterium	-	+	-
Glonium_stellatum	-	+	-
Ganoderma_sinense	-	+	-
Hydrogenophaga_flava	-	+	-
Brachyspira_intermedia	-	+	-
uncultured_Trichoderma	-	+	-
uncultured_bacterium_CSL12	-	+	-
Alphaproteobacteria_bacterium_MarineAlpha10_Bin2	-	+	-
Phaeospirillum_fulvum	-	+	-
Halorientalis_sp._F13-25	-	+	-
Candidatus_Magasanikbacteria_bacterium_GW2011_GWC2_41	-	+	-
uncultured_Gemmatimonadetes_bacterium_Rifle_16ft_4_minim	-	+	-
Phenylobacterium_immobile	-	+	-
Human_endogenous_retrovirus_H	-	+	-
Pantoea_sp._LMG_27579	-	+	-
Virgibacillus_sp._Bac330	-	+	-
Viridibacillus_arvi	-	+	-
uncultured_Pelodictyon_sp.	-	+	-
Deltaproteobacteria_bacterium_CG11_big_fil_rev_8_21_14_0_	-	+	-
Campylobacter_sp._P159	-	+	-
Deltaproteobacteria_bacterium_HGW-Deltaproteobacteria-14	-	+	-
Pyrenophora_teres	-	+	-
Verrucomicrobia_bacterium_RIFCSPHIGHO2_12_FULL_41_1	-	+	-
Anabaena_sp._PCC_7108	-	+	-
Ferrovum_sp._21-44-67	-	+	-
Hypoxylon_sp._EC38	-	+	-
Planococcus_halocryophilus	-	+	-
Vibrio_sp._qd031	-	+	-
Brachybacterium_nesterenkovii	-	+	-
Amycolatopsis_sp._CA-126428	-	+	-
Pseudonocardia_sp._MH-G8	-	+	-
Candidatus_Nitrososphaera_gargensis	-	+	-
Candidatus_Nealsonbacteria_bacterium_RIFCSPLOWO2_01_F	-	+	-
Pseudoalteromonas_sp._Xi13	-	+	-
Gammaproteobacteria_bacterium_TMED30	-	+	-
bacterium_TMED88	-	+	-
Flavobacterium_sp._LLJ-11	-	+	-
Gammaproteobacteria_bacterium_RIFCSPHIGHO2_12_FULL_	-	+	-
Parcubacteria_group_bacterium_GW2011_GWA2_47_21	-	+	-
Aeromonas_diversa	-	+	-
Aerococcus_sp._SJQ22	-	+	-
Gemmatimonas_sp._SG8_17	-	+	-
Frankia_inefficax	-	+	-
Francisella_sp._TX077310	-	+	-
Actinobacteria_bacterium_13_2_20CM_2_72_6	-	+	-
Actinobacillus_minor	-	+	-
Acinetobacter_sp._MYb10	-	+	-
Candidatus_Curtissbacteria_bacterium_RIFCSPHIGHO2_02_39	-	+	-
Actinomyces_oris	-	+	-

Actinomyces_johnsonii	-	+	-
Candidatus_Curtissbacteria_bacterium_RIFCSPHIGHO2_12_FU	-	+	-
Fusarium_avenaceum	-	+	-
Aestuariimicrobium_kwangyangense	-	+	-
Enterococcus_sp._2G9_DIV0600	-	+	-
Alicyclobacillus_mali	-	+	-
Candidatus_Arthromitus_sp._SFB-2	-	+	-
Brevibacterium_luteolum	-	+	-
Zobellia_uliginosa	-	+	-
Pseudomonas_kribbensis	-	+	-
Gymnopus_luxurians	-	+	-
Acidipila_dinghuensis	-	+	-
Pseudomonas_sp._FW306-2-11AA	-	+	-
Xenococcus_sp._PCC_7305	-	+	-
Pseudomonas_sp._GV047	-	+	-
Yersinia_intermedia	-	+	-
Acidimicrobium_sp._BACL27_MAG-120823-bin4	-	+	-
Enterococcus_phage_vB_EfaS_Max	-	+	-
Agrococcus_jejuensis	-	+	-
Candidatus_Gottesmanbacteria_bacterium_RBG_16_43_7	-	+	-
Parcubacteria_group_bacterium_GW2011_GWA2_36_10	-	+	-
Agromyces_cerinus	-	+	-
Enterococcus_phage_vB_EfaS_LM99	-	+	-
Granulicatella_sp._HMSC31F03	-	+	-
Candidatus_Hodgkinia_cicadicola	-	+	-
Peptoniphilus_sp._oral_taxon_375	-	+	-
Pediococcus_inopinatus	-	+	-
Neurospora_crassa	-	+	-
Marinomonas_mediterranea	-	+	-
Bacteroidetes/Chlorobi_group_bacterium_MS-B_bin-24	-	+	-
Streptomyces_viridifaciens	-	+	-
Cellulosimicrobium_cellulans	-	+	-
Sulfolobales_archaeon_SCGC_AB-777_K09	-	+	-
Nitrospina_gracilis	-	+	-
Saimiriine_gammaherpesvirus_2	-	+	-
Streptomyces_sp._CNZ288	-	+	-
Saccharomyces_arboricola	-	+	-
Nesterenkonia_sp._RB2	-	+	-
Streptomyces_sp._NEAU-D10	-	+	-
Legionella_israelensis	-	+	-
Coccidioides_immitis	-	+	-
Lautropia_sp.	-	+	-
Lautropia_mirabilis	-	+	-
Bacteroidetes_bacterium_RIFOXYA12_FULL_40_10	-	+	-
Colletotrichum_incarnatum	-	+	-
Collinsella_sp._60_9	-	+	-
Synechococcus_sp._NIES-970	-	+	-
Roseovarius_nubinhibens	-	+	-
Synechococcus_sp._BDU_130192	-	+	-
Lewinella_cohaerens	-	+	-
Leptotrichia_sp._oral_taxon_225	-	+	-
Deinococcus_pimensis	-	+	-
Leptolyngbya_sp._O-77	-	+	-
Micrococcus_lylae	-	+	-
Bacillus_sp._S66	-	+	-

Bacillus_sp._cl95	-	+	-
Chryseomicrobium_excrementi	-	+	-
Mycobacterium_kubicae	-	+	-
Spirobacillus_cienkowskii	-	+	-
Mycobacterium_talmoniae	-	+	-
Spirochaetaceae_bacterium	-	+	-
Sphingomonadales_bacterium_12-68-11	-	+	-
Sphingobium_sp._66-54	-	+	-
Microbacterium_testaceum	-	+	-
Bacillus_sp._MYb56	-	+	-
Methanomicrobiales_archaeon_HGW-Methanomicrobiales-2	-	+	-
Bacillus_sp._BO	-	+	-
Bacillus_sp._B14905	-	+	-
Methanofollis_sp._FWC-SCC2	-	+	-
Chlorobi_bacterium_OLB7	-	+	-
Chlamydiales_bacterium_38-26	-	+	-
Streptomyces_hirsutus	-	+	-
Streptomyces_melanosporofaciens	-	+	-
Simonsiella_muelleri	-	+	-
Myxococcales_bacterium_SG8_38	-	+	-
Chloroflexi_bacterium_RBG_13_52_14	-	+	-
Chloroflexi_bacterium_RBG_13_51_18	-	+	-
Bacillus_sp._FJAT-20673	-	+	-
Leptospira_terpstrae	-	+	-
Labrys_okinawensis	-	+	-
Candidatus_Syntrophoarchaeum_sp._WYZ-LMO15	-	+	-
Rhodobaca_barguzinensis	-	+	-
Lachancea_mirantina	-	+	-
Rhodanobacter_sp._Root480	-	+	-
Thielaviopsis_punctulata	-	+	-
Aquimarina_sp._AD1	-	+	-
Candidatus_Shapirobacteria_bacterium	-	+	-
Thermodesulfatator_sp.	-	+	-
Oceanobacillus_kimchii	-	+	-
Thermofilum_sp._ex4484_15	-	+	-
Thermoplasma_volcanium	-	+	-
Candidatus_Taylorbacteria_bacterium_RIFCSPHIGHO2_02_49	-	+	-
Kushneria_sp._YCWA18	-	+	-
Candidatus_Schekmanbacteria_bacterium_GWA2_38_11	-	+	-
Blastococcus_sp._DSM_46838	-	+	-
Trichophyton_tonsurans	-	+	-
Opitutaceae_bacterium_TAV1	-	+	-
Blastococcus_sp._DSM_44272	-	+	-
Candidatus_Rubidus_massiliensis	-	+	-
Jiangella_alba	-	+	-
Candidatus_Portnoybacteria_bacterium_RBG_13_40_8	-	+	-
Aquabacterium_sp.	-	+	-
Candidatus_Saganbacteria_bacterium_CG08_land_8_20_14_0_[illegible]	-	+	-
Kriegella_aquimaris	-	+	-
Bifidobacterium_sp._AGR2158	-	+	-
Omnitrophica_WOR_2_bacterium_RIFCSPHIGHO2_01_FULL	-	+	-
Curvibacter_sp._AEP1-3	-	+	-
Rhizobium_sp._63-7	-	+	-
Rhizobium_hainanense	-	+	-
Treponema_sp._GWB1_62_6	-	+	-

Corallococcus_sp._AB038B	-	+	-
Candidatus_Yonathbacteria_bacterium_CG23_combo_of_CG06	-	+	-
Aspergillus_sclerotialis	-	+	-
Coprobacillus_sp._AF24-1LB	-	+	-
Candidatus_Woesearchaeota_archaeon_CG10_big_fil_rev_8_21	-	+	-
Lactobacillus_sp._143-6	-	+	-
Thermaerobacter_marianensis	-	+	-
Noviherbaspirillum_sp._K1S02-23	-	+	-
Candidatus_Woykebacteria_bacterium_GWA1_44_8	-	+	-
Candidatus_Woesebacteria_bacterium_RIFOXYD1_FULL_43_	-	+	-
Thermoactinospora_rubra	-	+	-
Arthrobacter_saudimassiliensis	-	+	-
Rhodococcus_sp._EsD8	-	+	-
Arthrobacter_oryzae	-	+	-
Oceanicaulis_sp._PT13A	-	+	-
Thiocapsa_sp._KS1	+	+	-
Myroides_marinus	+	+	-
Sutterella_sp._KLE1602	+	+	-
Nitrospira_bacterium_SG8_35_4	+	+	-
Spiroplasma_culicicola	+	+	-
Rubritalea_profundi	+	+	-
Collinsella_sp._AM41-2BH	+	+	-
Flavobacteriaceae_bacterium_KYPW7	+	+	-
Massilia_sp._PDC64	+	+	-
Catalinimonas_alkaloidigena	+	+	-
Arthrobacter_sp._JZ_R-35	+	+	-
Nitrosospira_briensis	+	+	-
Pneumocystis_murina	+	+	-
Helicobacter_sp._CNRCH_2005/566H	+	+	-
Melioribacter_roseus	+	+	-
Deltaproteobacteria_bacterium_RBG_16_49_23	+	+	-
Cyanothece_sp._BG0011	+	+	-
Lactobacillus_selangorensis	+	+	-
Microbotryum_intermedium	+	+	-
Photobacterium_aquae	+	+	-
Marinilactibacillus_piezotolerans	+	+	-
Cyanothece_sp._ATCC_51142	+	+	-
Pseudoalteromonas_sp._BSi20429	+	+	-
Candidatus_Sulfopaludibacter_sp._SbA6	+	+	-
uncultured_crAssphage	-	+	-
Flavobacterium_sp._IMCC34762	-	+	-
Nautilia_profundicola	-	+	-
Flavobacterium_terrigena	-	+	-
Deltaproteobacteria_bacterium_HGW-Deltaproteobacteria-23	-	+	-
Gryllotalpicola_ginsengisoli	-	+	-
Lactobacillus_nasuensis	-	+	-
Chryseobacterium_sp._39-10	-	+	-
Fusarium_graminearum	-	+	-
Leptolyngbya_valderiana	-	+	-
uncultured_bacterium_Ad_091_F22_contig2	-	+	-
Paraphaeosphaeria_sporulosa	-	+	-
Nocardioides_sp._LS1	-	+	-
Pediococcus_parvulus	-	+	-
Pneumocystis_carinii	-	+	-
Candidatus_Pacearchaeota_archaeon_CG10_big_fil_rev_8_21_[illegible]	-	+	-

Chromatiales_bacterium_21-64-14	-	+	-
Microbulbifer_pacificus	-	+	-
Aspergillus_udagawae	-	+	-
Aspergillus_ruber	-	+	-
Amycolatopsis_jejuensis	-	+	-
Henriciella_pelagia	-	+	-
Iodobacter_sp._BJB302	-	+	-
Verminephrobacter_aporrectodeae	-	+	-
Citrobacter_youngae	-	+	-
Rhodothermaeota_bacterium_MED-G19	-	+	-
Exiguobacterium_undae	-	+	-
Rhizobacter_sp._S-16	-	+	-
Mycobacterium_kansasii	-	+	-
Nonlabens_sp._MIC269	-	+	-
Ophiocordyceps_camponoti-rufipedis	-	+	-
Leeia_oryzae	-	+	-
Staphylococcus_massiliensis	-	+	-
Solibacillus_sp._R5-41	-	+	-
Anaplasma_phagocytophilum	-	+	-
Erwinia_sp._Leaf53	-	+	-
Paramecium_bursaria_Chlorella_virus_CviKI	-	+	-
Fusarium_acuminatum	-	+	-
Thermococcus_gammatolerans	-	+	-
Parcubacteria_group_bacterium_SW_4_46_8	-	+	-
candidate_division_TM6_bacterium_GW2011_GWE2_41_16	-	+	-
Salana_multivorans	-	+	-
Promicromonospora_thailandica	-	+	-
Actinoplanes_sp._ATCC_53533	-	+	-
Candidatus_Falkowbacteria_bacterium_RIFOXYA2_FULL_38_	-	+	-
Armillaria_solidipes	-	+	-
candidate_division_KD3-62_bacterium_DG_56	-	+	-
Helicobacter_sp._MIT_05-5294	-	+	-
Panaeolus_cyanescens	-	+	-
Bradymonas_sediminis	-	+	-
Salinicoccus_albus	-	+	-
Geobacillus_yumthangensis	-	+	-
Chloroflexi_bacterium_OLB13	-	+	-
bacterium_BMS3Bbin04	-	+	-
Schizosaccharomyces_octosporus	-	+	-
Rhizobium_taibaishanense	-	+	-
Ascidiaceihabitans_donghaensis	-	+	-
cyanobacterium_PCC_7702	-	+	-
Nitrospira_sp._CG24C	-	+	-
Synechococcus_sp._BL107	-	+	-
Arthropoda_environmental_sample	-	+	-
bacterium_BMS3Bbin09	-	+	-
Halomonas_venusta	-	+	-
Parasutterella_excrementihominis_CAG:233	+	-	-
Salegentibacter_flavus	+	-	-
Streptomyces_sp._DSM_15324	+	-	-
Hymenobacter_sp._sh-6	+	-	-
Flavobacterium_sp._BBQ-12	+	-	-
Pseudomonas_fuscovaginae	+	-	-
Akkermansia_sp._CAG:344	+	-	-
Weeksella_massiliensis	+	-	-

Chryseobacterium_soli	+	-	-
Achromobacter_pulmonis	+	-	-
Flavobacterium_haoranii	+	-	-
Sulfuritalea_hydrogenivorans	+	-	-
Candidatus_Gastranaerophilales_bacterium_HUM_19	+	-	-
Geobacillus_kaustophilus	+	-	-
Rickettsiaceae_bacterium_4572_127	+	-	-
Nocardioides_sp._Soil797	+	-	-
Acinetobacter_sp._CAG:196	+	-	-
Anditalea_andensis	+	-	-
Leeuwenhoekiella_palythoae	+	-	-
Paracoccus_aestuarii	+	-	-
Nocardioides_insulae	+	-	-
Mucilaginibacter_sp._MYSH2	+	-	-
Olsenella_sp._AM39-30AC	+	-	-
Akkermansia_sp._KLE1797	+	-	-
Firmicutes_bacterium_CAG:536	+	-	-
Clostridium_phage_phiCP39-O	+	-	-
Synechococcus_sp._PCC_7003	+	-	-
Anaerobacillus_macyae	+	-	-
Carnobacterium_sp._AT7	+	-	-
Shewanella_sp._POL2	+	-	-
Chloroflexi_bacterium_RBG_16_50_11	+	-	-
Lysinibacillus_parviboronicapiens	+	-	-
Geobacter_pickeringii	+	-	-
Burkholderiales_bacterium_YL45	+	-	-
Akkermansia_sp._aa_0143	+	-	-
Dehalogenimonas_formicexedens	+	-	-
Chryseobacterium_sp._G0162	+	-	-
Brochothrix_phage_NF5	+	-	-
Chlorobium_chlorochromatii	+	-	-
Clostridium_phage_phiCP13O	+	-	-
Staphylococcus_phage_66	+	-	-
Candidatus_Woesebacteria_bacterium_CG22_combo_CG10-13_	+	-	-
Moraxella_macacae	+	-	-
bacterium_BMS3Abin05	+	-	-
Enterococcus_phage_vB_EfaP_IME199	+	-	-
Staphylococcus_phage_SCH1	+	-	-
Pedobacter_hartonius	+	-	-
Rhodomicrobium_sp._JA980	+	-	-
Enterococcus_phage_vB_EfaP_IME195	+	-	-
Staphylococcus_phage_Pontiff	+	-	-
Prevotella_sp._Sc00028	+	-	-
Erysipelotrichaceae_bacterium_CAG:64	+	-	-
Rhizobium_sp._AC27/96	+	-	-
Candidatus_Gastranaerophilales_bacterium_HUM_22	+	-	-
Algoriella_xinjiangensis	+	-	-
Persephonella_marina	+	-	-
Bacteroidetes_bacterium_RIFCSPLOWO2_02_FULL_36_8	+	-	-
Rhodobacterales_bacterium_RIFCSPHIGHO2_02_FULL_62_1	+	-	-
Variovorax_sp._YR216	+	-	-
Staphylococcus_phage_Pike	+	-	-
Candidatus_Peregrinibacteria_bacterium_GW2011_GWC2_33_	+	-	-
Nitrospira_sp._SCGC_AG-212-E16	+	-	-
Parafilimonas_terrae	+	-	-

Arcobacter_sp._F2176	+	-	-
Lactobacillus_kefiranofaciens	+	-	-
Flavobacterium_omnivorum	+	-	-
Spirosoma_pollinicola	+	-	-
Chryseobacterium_sp._Leaf394	+	-	-
Longibacter_salinarum	+	-	-
Bacillus_sp._JCA	+	-	-
Emticicia_sp._C21	+	-	-
Paenibacillus_sp._TCA20	+	-	-
Candidatus_Campbellbacteria_bacterium_CG11_big_fil_rev_8_	+	-	-
Leptolyngbya_sp._PCC_7375	+	-	-
Candidatus_Thorarchaeota_archaeon_SMTZ-45	+	-	-
Chryseobacterium_daecheongense	+	-	-
Proteus_vulgaris	+	-	-
Moraxella_osloensis	+	-	-
Methanosarcina_sp._Kolksee	+	-	-
Bacillus_xiamenensis	+	-	-
Clostridium_phage_phiCP26F	+	-	-
Pseudodesulfovibrio_profundus	+	-	-
Bifidobacterium_minimum	+	-	-
Larkinella_arboricola	+	-	-
uncultured_bacterium_fosmid_pJB154B8_contig_I	+	-	-
Olsenella_sp._SIT9	+	-	-
Bifidobacterium_tissieri	+	-	-
Lactobacillus_sp._wkB8	+	-	-
Flavobacterium_lindanitolerans	+	-	-
Delftia_lacustris	+	-	-
Clostridium_sp._HMSC19A11	+	-	-
Bacteroidetes_bacterium_SW_10_40_5	+	-	-
uncultured_bacterium_Contigcl_23	+	-	-
Candidatus_Gastranaerophilales_bacterium_HUM_5	+	-	-
Bacillus_virus_phi29	+	-	-
Candidatus_Gastranaerophilales_bacterium_HUM_15	+	-	-
Candidatus_Moranbacteria_bacterium_CG06_land_8_20_14_3_	+	-	-
Chryseobacterium_taeanense	+	-	-
Sutterella_wadsworthensis_CAG:135	+	-	-
Gimesia_maris	+	-	-
Leptolyngbya_boryana	+	-	-
Mesotoga_sp._SC_NapDC3	+	-	-
Eoetvoesia_caeni	+	-	-
Sulfurospirillum_cavolei	+	-	-
Streptococcus_canis	+	-	-
Polaribacter_dokdonensis	+	-	-
Lactobacillus_sp._HT111-2	+	-	-
Dakarella_massiliensis	+	-	-
Flavivirga_eckloniae	+	-	-
Roseospirillum_parvum	+	-	-
Agrobacterium_rhizogenes	+	-	-
Endozoicomonas_montiporae	+	-	-
Neisseria_bergeri	+	-	-
Bacteroides_sp._AM18-9	+	-	-
Bifidobacterium_actinocoloniiforme	+	-	-
Catenovulum_maritimum	+	-	-
Candidatus_Staskawiczbacteria_bacterium_RIFOXYC1_FULL_	+	-	-
Candidatus_Gastranaerophilales_bacterium_HUM_12	+	-	-

Legionella_lansingensis	+	-	-
Thermotoga_sp._RQ7	+	-	-
Microcoleus_sp._PCC_7113	+	-	-
Hydrogenobaculum_sp._Y04AAS1	+	-	-
Xanthomonadaceae_bacterium_SCN_69-320	+	-	-
Lactobacillus_coryniformis	+	-	-
Candidatus_Electronema_sp._GS	+	-	-
Alteromonas_australica	+	-	-
Arcobacter_sp._LMG_29976	+	-	-
Streptomyces_azureus	+	-	-
Streptomyces_sp._RTd22	+	-	-
Desulfonatronum_sp._SC1	+	-	-
uncultured_Mediterranean_phage_uvDeep-CGR2-KM24-C165	+	-	-
Flavobacterium_sp._40-81	+	-	-
Staphylococcus_succinus	+	-	-
Rhodovibrio_salinarum	+	-	-
Abiotrophia_sp._HMSC24B09	+	-	-
Elusimicrobia_bacterium_GWF2_62_30	+	-	-
Streptococcus_sp._oral_taxon_431	+	-	-
Flavobacterium_sp._URHB0058	+	-	-
Riemerella_columbina	+	-	-
Jiangella_muralis	+	-	-
Marinobacterium_aestuarii	+	-	-
Sulfurihydrogenibium_yellowstonense	+	-	-
Inquilinus_limosus	+	-	-
Sneathiella_sp.	+	-	-
Leptospira_macculloughii	+	-	-
Leptospira_borgpetersenii	+	-	-
Acinetobacter_bereziniae	+	-	-
Eggerthella_phage_PMBT5	+	-	-
Streptomyces_sp._NBRC_110465	+	-	-
Deltaproteobacteria_bacterium_HGW-Deltaproteobacteria-16	+	-	-
Clostridium_phage_D-1873	+	-	-
Polaribacter_sp._ALD11	+	-	-
Flavobacteria_bacterium_BAL38	+	-	-
Chromobacterium_vaccinii	+	-	-
Burkholderia_sp._Bp9140	+	-	-
Fictibacillus_macauensis	+	-	-
Methylobacillus_sp._MM3	+	-	-
Candidatus_Gastranaerophilales_bacterium_HUM_14	+	-	-
Salipiger_bermudensis	+	-	-
Porphyromonas_sp._COT-108_OH1349	+	-	-
Brevundimonas_sp._NS26	+	-	-
Castellaniella_defragrans	+	-	-
Thermoflexibacter_ruber	+	-	-
Staphylococcus_muscae	+	-	-
Bacteroidetes_bacterium_OLB8	+	-	-
Mesotoga_sp._HF07.pep.5.2.highcov	+	-	-
bacterium_CAVE-375	+	-	-
Halobacterium_salinarum	+	-	-
CRESS_virus_sp.	+	-	-
candidate_division_WOR-1_bacterium_RIFOXYA2_FULL_51_	+	-	-
Elusimicrobia_bacterium_RIFOXYA2_FULL_50_26	+	-	-
Pelobacter_sp._SFB93	+	-	-
Belnapia_rosea	+	-	-

Synechococcus_elongatus	+	-	-
Elusimicrobia_bacterium_CG03_land_8_20_14_0_80_50_18	+	-	-
Candidatus_Falkowbacteria_bacterium_CG10_big_fil_rev_8_21	+	-	-
Rhodospirillaceae_bacterium	+	-	-
Sphingobium_phenoxybenzoativorans	+	-	-
Methylorubrum_extorquens	+	-	-
Synechococcus_sp._WH_5701	+	-	-
Brevibacterium_aurantiacum	+	-	-
Desulfuromonas_sp._SDB	+	-	-
Massilia_sp._Leaf139	+	-	-
Thiocapsa_rosea	+	-	-
Apis_mellifera_associated_microvirus_35	+	-	-
Aquimarina_sp._BL5	+	-	-
Vibrio_natriegens	+	-	-
Lactobacillus_siliginis	+	-	-
Lactobacillus_panisapium	+	-	-
Caecomyces_sp.	+	-	-
Prosthecochloris_sp._V1	+	-	-
candidate_division_TM6_bacterium_GW2011_GWF2_32_72	+	-	-
Bisgaard_Taxon_44	+	-	-
Flavobacterium_micromati	+	-	-
Phenylobacterium_sp._SCN_70-31	+	-	-
Campylobacter_sp._P0087	+	-	-
Sporosarcina_sp._EUR3_2.2.2	+	-	-
Thiomonas_sp._X19	+	-	-
Gilliamella_mensalis	+	-	-
Neptuniibacter_marinus	+	-	-
Chroococcidiopsis_thermalis	+	-	-
Aestuariispira_insulae	+	-	-
Ignavibacteria_bacterium_RIFCSPHIGHO2_02_FULL_56_12	+	-	-
Kiloniella_laminariae	+	-	-
Pseudoalteromonas_rubra	+	-	-
Deltaproteobacteria_bacterium_RBG_13_52_11	+	-	-
Paenibacillus_sp._276b	+	-	-
Virgibacillus_alimentarius	+	-	-
Thermotoga_maritima	+	-	-
Bacillus_sp._V33-4	+	-	-
Bacillus_sp._FJAT-42315	+	-	-
Serratia_plymuthica	+	-	-
Acinetobacter_sp._RIT592	+	-	-
Rhodospirillales_bacterium_CG15_BIG_FIL_POST_REV_8_21	+	-	-
Lacticigenium_naphtae	+	-	-
Thauera_sp._K11	+	-	-
Phyllobacterium_myrsinacearum	+	-	-
Planktothrix_agardhii	+	-	-
Pseudoxanthomonas_suwonensis	+	-	-
Parcubacteria_group_bacterium_GW2011_GWA2_42_11	+	-	-
Shewanella_algae	+	-	-
Streptomyces_sp._CBMAI_2042	+	-	-
Chryseobacterium_chaponense	+	-	-
Citrobacter_koseri	+	-	-
Tepidimonas_fonticaldi	+	-	-
Azospirillum_sp._B2	+	-	-
Candidatus_Roizmanbacteria_bacterium_RIFCSPHIGHO2_01_	+	-	-
Caulobacter_sp._UNC279MFTsu5.1	+	-	-

Bacteroidetes_bacterium_RBG_19FT_COMBO_42_10	+	-	-
Nonomuraea_sp._KC333	+	-	-
Listeria_grandensis	+	-	-
Acidovorax_sp._56	+	-	-
uncultured_marine_bacterium_Ant29B7	+	-	-
bacterium_HR25	+	-	-
Winogradskyella_sp._PC-19	+	-	-
Winogradskyella_thalassocola	+	-	-
Thermopetrobacter_sp._TC1	+	-	-
Rhodospira_trueperi	+	-	-
Kordiimonas_gwangyangensis	+	-	-
delta_proteobacterium_MLMS-1	+	-	-
Alphaproteobacteria_bacterium_41-28	+	-	-
Azospirillum_doebereinerae	+	-	-
Flavobacterium_johnsoniae	+	-	-
Psychrosphaera_saromensis	+	-	-
Stenotrophomonas_acidaminiphila	+	-	-
Alphaproteobacteria_bacterium_CG_4_9_14_3_um_filter_47_1	+	-	-
Campylobacter_sp._P146	+	-	-
Tepidicaulis_marinus	+	-	-
Staphylococcus_phage_Stab23	+	-	-
Candidatus_Kaiserbacteria_bacterium_RIFCSPHIGHO2_01_FU	+	-	-
Maribacter_sp._4G9	+	-	-
Lysobacter_sp._cf310	+	-	-
Opitutaceae_bacterium_TAV4	+	-	-
Rathayibacter_phage_NCPPB3778	+	-	-
Marinitoga_piezophila	+	-	-
Candidatus_Woesebacteria_bacterium_GW2011_GWC2_45_9	+	-	-
Neisseria_sicca	+	-	-
Lactobacillus_aquaticus	+	-	-
Kocuria_sp._442	+	-	-
Salmonella_virus_SPN3US	+	-	-
Planctomicrobium_piriforme	+	-	-
Corynebacterium_vitaeruminis	+	-	-
Veillonella_rodentium	+	-	-
Chelatococcus_daeguensis	+	-	-
Candidatus_Falkowbacteria_bacterium_CG_4_10_14_0_8_um_	+	-	-
Caenispirillum_salinarum	+	-	-
Colwellia_psychrerythraea	+	-	-
Nitrosomonas_sp._Nm132	+	-	-
Rhodobacteraceae_bacterium_BAR1	+	-	-
Bacillus_sp._V44-8	+	-	-
Sphingobium_ummariense	+	-	-
Oceanibaculum_pacificum	+	-	-
Rhodobacter_megalophilus	+	-	-
Sphingobium_sp.	+	-	-
Tropicimonas_sp._IMCC6043	+	-	-
Haematospirillum_jordaniae	+	-	-
Tolypothrix_campylonemoides	+	-	-
Candidatus_Cloacimonas_sp._4484_275	+	-	-
Rhodobacterales_bacterium_34-62-10	+	-	-
Rhodospirillales_bacterium_69-11	+	-	-
Rothia_sp._HMSC08A08	+	-	-
Candidatus_Finniella_inopinata	+	-	-
Indibacter_alkaliphilus	+	-	-

Flavobacterium_sp._83	+	-	-
Petrotoga_sp._HWHPT.55.6.3	+	-	-
Leucobacter_komagatae	+	-	-
Riemerella_sp.	+	-	-
Methanobrevibacter_millerae	+	-	-
Rhizobium_sp._RU36D	+	-	-
Desulfofaba_hansenii	+	-	-
Actinotalea_ferrariae	+	-	-
Clostridium_massiliodielmoense	+	-	-
Duganella_sp._BJB475	+	-	-
Mucilaginibacter_sp._NFR10	+	-	-
Gaiellales_bacterium	+	-	-
Halomarina_oriensis	+	-	-
Alphaproteobacteria_bacterium_CG11_big_fil_rev_8_21_14_0_	+	-	-
Sporosarcina_pasteurii	+	-	-
Lactobacillus_timberlakei	+	-	-
Candidatus_Gastranaerophilales_bacterium_HUM_2	+	-	-
Pseudoalteromonas_piratica	+	-	-
Enterobacteriaceae_bacterium_ENNIH1	+	-	-
Lautropia_sp._SCN_70-15	+	-	-
Pseudomonas_phage_phiAH14a	+	-	-
Exiguobacterium_antarcticum	+	-	-
Eudoraea_adriatica	+	-	-
Lacinutrix_sp._5H-3-7-4	+	-	-
Variovorax_sp._502	+	-	-
Flavobacteriaceae_bacterium_3519-10	+	-	-
Lactobacillus_phage_P1174	+	-	-
Kazachstania_servazzii	+	-	-
methanotrophic_bacterial_endosymbiont_of_Bathymodiolus_sp.	+	-	-
Psychroserpens_burtonensis	+	-	-
Candidatus_Omnitrophica_bacterium_CG11_big_fil_rev_8_21_	+	-	-
Ammonifex_thiophilus	+	-	-
Streptomyces_sp._TLI_053	+	-	-
Bordetella_parapertussis	+	-	-
Acinetobacter_sp._SWAC57	+	-	-
Ottowia_thiooxydans	+	-	-
Maribacter_sp._1_2014MBL_MicDiv	+	-	-
Candidatus_Nardonella_dryophthoridicola	+	-	-
Cronobacter_dublinensis	+	-	-
Robiginitomaculum_sp.	+	-	-
Labrenzia_sp._VG12	+	-	-
Bacillus_sp._CHD6a	+	-	-
Rhodococcus_opacus	+	-	-
Pelobacter_carbinolicus	+	-	-
Arthrobacter_phage_Anjali	+	-	-
Candidatus_Endolissoclinum_sp._TMED37	+	-	-
Sphingobacterium_sp._40-24	+	-	-
Candidatus_Melainabacteria_bacterium_RIFCSPHIGHO2_02_F	+	-	-
Cyanobacteria_bacterium_QS_8_64_29	+	-	-
Derxia_lacustris	+	-	-
Syntrophus_sp._PtaU1.Bin005	+	-	-
Geminicoccus_roseus	+	-	-
Porphyromonas_sp._COT-290_OH3588	+	-	-
Marinobacter_nanhaiticus	+	-	-
Leptolyngbya_sp._NIES-3755	+	-	-

Balneola_sp.	+	-	-
Acidobacteria_bacterium_RIFCSPLOWO2_02_FULL_60_20	+	-	-
Lactobacillus_herbarum	+	-	-
Gordonia_sp._KTR9	+	-	-
Candidatus_Buchananbacteria_bacterium_RIFCSPHIGHO2_01_	+	-	-
Rhodanobacter_sp._FW104-R8	+	-	-
Piscirickettsia_salmonis	+	-	-
Pedobacter_sp._RS10	+	-	-
Alcanivorax_sp._NBRC_102024	+	-	-
Plantibacter_sp._YR521	+	-	-
Agromyces_sp._CF514	+	-	-
Candidatus_Aminicenantes_bacterium_RBG_16_63_14	+	-	-
uncultured_bacterium_Contig160	+	-	-
Frankia_sp._CcI156	+	-	-
Eikenella_sp._NML120348	+	-	-
Nocardia_sp._BMG51109	+	-	-
Candidatus_Nomurabacteria_bacterium_GW2011_GWA1_46_1	+	-	-
Bipolaris_oryzae	+	-	-
Dyella_marensis	+	-	-
bacterium_HR20	+	-	-
bacterium_HR15	+	-	-
Enterococcus_sp._3G1_DIV0629	+	-	-
Ralstonia_virus_RSL1	+	-	-
Proteobacteria_bacterium_TMED72	+	-	-
Parcubacteria_group_bacterium_GW2011_GWD2_38_11	+	-	-
Pseudomonadales_bacterium	+	-	-
Candidatus_Gottesmanbacteria_bacterium_RBG_16_38_7b	+	-	-
Caulobacteraceae_bacterium_OTSz_A_272	+	-	-
Cupriavidus_sp._HPC(L)	+	-	-
Microbacterium_trichothecenolyticum	+	-	-
Microbacterium_sp._CF335	+	-	-
Microbulbifer_thermotolerans	+	-	-
Lactobacillus_sp._ESL0230	+	-	-
Lactobacillus_sp._ESL0259	+	-	-
Bacillus_sp._V59.32a	+	-	-
Streptomyces_fulvoviolaceus	+	-	-
Sulfuricurvum_sp._PD_MW2	+	-	-
Bacillus_phage_vB_BhaS-171	+	-	-
Hymenobacter_sp._9PBR-2	+	-	-
Syntrophobacterales_bacterium_RBG_19FT_COMBO_59_10	+	-	-
Methanosphaera_sp._rholeuAM270	+	-	-
Methanosarcina_sp._MSH10X1	+	-	-
Limnohabitans_sp._WS1	+	-	-
Streptomyces_acidiscabies	+	-	-
Vibrio_phage_VP4B	+	-	-
Methanobrevibacter_wolinii	+	-	-
Bacillus_phage_Harambe	+	-	-
Coprococcus_sp._OM06-25	+	-	-
Alicyclobacillus_kakegawensis	+	-	-
Chryseobacterium_sp._CBTAP_102	+	-	-
Cryomorphaceae_bacterium_BACL22_MAG-120619-bin32	+	-	-
Cruoricaptor_ignavus	+	-	-
Thiohalospira_halophila	+	-	-
Desulfurella_multipotens	+	-	-
Kordia_zhangzhouensis	+	-	-

Thielavia_terrestris	+	-	-
Thermodesulfobacterium_hydrogeniphilum	+	-	-
uncultured_Alphaproteobacteria_bacterium	+	-	-
Azospirillum_sp._M2T2B2	+	-	-
[Candida]_boidinii	+	-	-
Rhodoplanes_piscinae	+	-	-
Helicobacter_macacae	+	-	-
Kiloniella_majae	+	-	-
Ruminococcus_sp._AM23-1LB	+	-	-
Reyranella_sp.	+	-	-
Sulfuritortus_calidifontis	+	-	-
Ruminococcus_sp._AF31-16BH	+	-	-
Sphingopyxis_macrogoltabida	+	-	-
Paenibacillus_sp._FSL_H8-0259	+	-	-
Variovorax_sp._DXTD-1	+	-	-
Sulfurovum_sp._NBC37-1	+	-	-
Pelagibacteraceae_bacterium_TMED13	+	-	-
Tropicibacter_phthalicicus	+	-	-
Alteribacillus_persepolensis	+	-	-
Alphaproteobacteria_bacterium_CG1_02_46_17	+	-	-
endosymbiont_of_Acanthamoeba_sp._UWC8	+	-	-
Roseomonas_rhizosphaerae	+	-	-
Paenibacillus_sp._SMB1	+	-	-
Pediococcus_acidilactici	+	-	-
Lactobacillus_koreensis	+	-	-
Chitinimonas_koreensis	+	-	-
Xanthomonas_hyacinthi	+	-	-
Veillonella_criceti	+	-	-
Bibersteinia_trehalosi	+	-	-
Arenibacter_hampyeongensis	+	-	-
Armatimonadetes_bacterium_JP3_11	+	-	-
Veillonella_sp._T11011-6	+	-	-
Sinorhizobium_meliloti	+	-	-
Ruminococcus_sp._AM09-18-1	+	-	-
Burkholderiales_bacterium_JOSHI_001	+	-	-
Corynebacterium_ulceribovis	+	-	-
uncultured_bacterium_Contigcl_1787	+	-	-
Lunatimonas_lonarensis	+	-	-
Labrenzia_sp._DG1229	+	-	-
Lysinibacillus_sp._AR18-8	+	-	-
Candidatus_Accumulibacter_sp._SK-12	+	-	-
Flavobacterium_sp._CF136	+	-	-
Synechococcus_sp._PCC_8807	+	-	-
Candidatus_Altiarchaeales_archaeon_ex4484_2	+	-	-
Aquitalea_sp._MWU14-2217	+	-	-
Pseudomonas_virus_R18	+	-	-
Pseudaminobacter_salicylatoxidans	+	-	-
Proteobacteria_bacterium_ST_bin12	+	-	-
Planctomycetes_bacterium_RIFOXYB12_FULL_42_10	+	-	-
Planctomycetes_bacterium_RBG_13_62_9	+	-	-
Floricoccus_penangensis	+	-	-
uncultured_pig_faeces_bacterium	+	-	-
Candidatus_Roizmanbacteria_bacterium_CG2_30_33_16	+	-	-
Candidatus_Roizmanbacteria_bacterium_RIFCSPHIGHO2_01_	+	-	-
Kozakia_baliensis	+	-	-

Lactobacillus_phage_JCL1032	+	-	-
Cryptosporangium_aurantiacum	+	-	-
Aphanothece_hegewaldii	+	-	-
Lactobacillus_phage_KC5a	+	-	-
Idiomarina_sediminum	+	-	-
Pedobacter_oryzae	+	-	-
Thiohalorhabdus_denitrificans	+	-	-
Lactobacillus_pantheris	+	-	-
Kushneria_aurantia	+	-	-
Propionibacterium_australiense	+	-	-
Brackiella_oedipodis	+	-	-
Collimonas_fungivorans	+	-	-
Pseudomonas_monteilii	+	-	-
Tersicoccus_sp._Bi-70	+	-	-
Candidatus_Kerfeldbacteria_bacterium_RIFCSPHIGHO2_12_F	+	-	-
Cadophora_sp._DSE1049	+	-	-
Veillonellaceae_bacterium	+	-	-
Deefgea_rivuli	+	-	-
Achromobacter_sp._NFACC18-2	+	-	-
Lactobacillus_sp._UMNPBX1	+	-	-
Burkholderia_diffusa	+	-	-
Flavobacterium_cyanobacteriorum	+	-	-
Arcobacter_sp._PSE-93	+	-	-
Leptospira_kmetyi	+	-	-
Candidatus_Marinimicrobia_bacterium_TMED108	+	-	-
Pandoraea_oxalativorans	+	-	-
Lachnospiraceae_bacterium_AM23-2LB	+	-	-
Ensifer_adhaerens	+	-	-
Pseudomonas_sp._HLS-6	+	-	-
Flavobacteriales_bacterium_CG_4_9_14_3_um_filter_40_17	+	-	-
Deltaproteobacteria_bacterium_RBG_16_50_11	+	-	-
Leptolyngbya_sp._BC1307	+	-	-
Bradyrhizobium_sp._MOS003	+	-	-
Sodalis_praecaptivus	+	-	-
Micromonospora_avicenniae	+	-	-
Roseomonas_stagni	+	-	-
Rugamonas_rubra	+	-	-
Micromonospora_matsumotoense	+	-	-
Microcystis_wesenbergii	+	-	-
Sporosarcina_sp._P19	+	-	-
Gayadomonas_joobiniege	+	-	-
Capnocytophaga_sp._oral_taxon_338	+	-	-
Alloscardovia_macacae	+	-	-
Microbacterium_chocolatum	+	-	-
Microbacterium_sp._GCS4	+	-	-
Carnobacterium_sp._ZWU0011	+	-	-
Desulfosoma_caldarium	+	-	-
Nocardioides_sp._Root140	+	-	-
Chloroflexi_bacterium_RBG_16_48_8	+	-	-
Sedimenticola_sp.	+	-	-
Sphingomonas_indica	+	-	-
Schaalia_meyeri	+	-	-
Alcanivorax_sp._N3-2A	+	-	-
Chryseobacterium_lathyri	+	-	-
Shewanella_putrefaciens	+	-	-

Shewanella_halifaxensis	+	-	-
[Pseudomonas]_geniculata	+	-	-
Sphingobacterium_sp._HMA12	+	-	-
Bacillus_virus_Troll	+	-	-
Saccharophagus_degradans	+	-	-
Moritella_dasanensis	+	-	-
bacterium_(Candidatus_Howlettbacteria)_CG_4_10_14_0_8_un	+	-	-
Neisseria_sp._10022	+	-	-
Saliphagus_sp._LR7	+	-	-
Streptomyces_peucetius	+	-	-
Oleiphilus_sp._HI0072	+	-	-
Streptomyces_scabiei	+	-	-
Edaphobacter_aggregans	+	-	-
Ectothiorhodospira_mobilis	+	-	-
Rhizobium_sp._C16	+	-	-
Methanolinea_tarda	+	-	-
Methanobacterium_congolense	+	-	-
Fusobacterium_sp._HMSC064B11	+	-	-
Actinokineospora_cianjurensis	+	-	-
Streptococcus_sp._OH4692_COT-348	+	-	-
candidate_division_CPR2_bacterium_GW2011_GWC2_39_10	+	-	-
Novosphingobium_sp._PC22D	+	-	-
Rhodopirellula_islandica	+	-	-
Hymenobacter_sp._ELS1360	+	-	-
Omnitrophica_WOR_2_bacterium_SM23_72	+	-	-
Virgibacillus_pantothenticus	+	-	-
Rhizobium_sp._ACO-34A	+	-	-
Candidatus_Buchananbacteria_bacterium_RIFCSPHIGHO2_01_	+	-	-
Methanobacterium_sp._MB1	+	-	-
Anthracocystis_flocculosa	+	-	-
Nitratireductor_basaltis	+	-	-
Alphaproteobacteria_bacterium_GWF2_58_20	+	-	-
Gammaproteobacteria_bacterium_RIFCSPHIGHO2_12_FULL_	+	-	-
Alphaproteobacteria_bacterium_RIFCSPHIGHO2_02_FULL_4(	+	-	-
Sphingomonas_sp._ABOLF	+	-	-
Candidatus_Termititenax_spirochaetophilus	+	-	-
Caedimonas_varicaedens	+	-	-
Archangium_sp._Cb_G35	+	-	-
Alphaproteobacteria_bacterium_CG_4_10_14_0_8_um_filter_5	+	-	-
Gloeobacter_kilaueensis	+	-	-
Pseudomonas_alkylphenolica	+	-	-
Deltaproteobacteria_bacterium_SG8_13	+	-	-
Alphaproteobacteria_bacterium_MarineAlpha3_Bin2	+	-	-
Carnobacterium_funditum	+	-	-
Dyadobacter_jejuensis	+	-	-
Sphingorhabdus_marina	+	-	-
Gracilimonas_tropica	+	-	-
Candidatus_Pacearchaeota_archaeon_CG10_big_fil_rev_8_21_	+	-	-
Candidatus_Glomeribacter_gigasporarum	+	-	-
Thermobaculum_terrenum	+	-	-
Ralstonia_insidiosa	+	-	-
Anoxybacillus_gonensis	+	-	-
Verrucomicrobia_bacterium_21-51-4	+	-	-
Alphaproteobacteria_bacterium_TMED194	+	-	-
Actinopolymorpha_alba	+	-	-

Pararhizobium_polonicum	+	-	-
Alphaproteobacteria_bacterium_MarineAlpha10_Bin1	+	-	-
Sulfurimonas_sp._RIFOXYB12_FULL_35_9	+	-	-
Allochromatium_warmingii	+	-	-
Flavisolibacter_tropicus	+	-	-
Tistlia_consotensis	+	-	-
Aliihoeflea_sp._2WW	+	-	-
Candidatus_Zambryskibacteria_bacterium_RIFCSPHIGHO2_01	+	-	-
Rhodovulum_viride	+	-	-
Actinomyces_sp._CtC_72	+	-	-
Lactobacillus_sp._wkB10	+	-	-
Psathyrella_aberdarensis	+	-	-
Endocarpon_pusillum	+	-	-
Pedobacter_yonginense	+	-	-
Salipaludibacillus_sp._KQ-12	+	-	-
Calothrix_sp._NIES-4101	+	-	-
Flavobacterium_frigidimaris	+	-	-
Parcubacteria_group_bacterium_CG2_30_44_18	+	-	-
Taphrina_deformans	+	-	-
Holophaga_foetida	+	-	-
Chryseobacterium_sp._FH2	+	-	-
Alicyclobacillus_vulcanalis	+	-	-
Mycoplasma_gallinarum	+	-	-
Chryseobacterium_halperniae	+	-	-
Kutzneria_buriramensis	+	-	-
Metallibacterium_scheffleri	+	-	-
Blautia_sp._AM28-27	+	-	-
Streptomyces_mirabilis	+	-	-
Bacillus_sp._123MFChir2	+	-	-
Alsobacter_sp._SH9	+	-	-
Salinispora_pacifica	+	-	-
Sagittula_stellata	+	-	-
Actinomadura_sp._CNU-125	+	-	-
Spirosoma_spitsbergense	+	-	-
Prochlorococcus_marinus	+	-	-
Thermococcus_zilligii	+	-	-
Ponticoccus_sp._LZ-14	+	-	-
Antarctobacter_heliothermus	+	-	-
Solibacteres_bacterium_SbA2	+	-	-
Sphingomonas_sp._NIC1	+	-	-
Arcobacter_sp._LPB0137	+	-	-
Arcanobacterium_haemolyticum	+	-	-
Thermotoga_sp._SG1	+	-	-
Bifidobacterium_sp._2020B	+	-	-
Shewanella_sp._Alg231_23	+	-	-
uncultured_bacterium_Contigcl_1769	+	-	-
Rhizobiales_bacterium_63-22	+	-	-
candidate_division_WOR-1_bacterium_DG_54_3	+	-	-
Thioalkalivibrio_sp._ALE12	+	-	-
Sphingobacterium_sp._T2	+	-	-
Caldimicrobium_thiodismutans	+	-	-
Thiotrichales_bacterium_TMED285	+	-	-
Acinetobacter_sp._WCHAc060025	+	-	-
Arcobacter_sp._FW59	+	-	-
uncultured_bacterium_Ad_136_J17_contig2	+	-	-

Vibrio_sp._LJC006	+	-	-
[Candida]_auris	+	-	-
Aquabacter_sp._Sn-9-2	+	-	-
Aquaspirillum_serpens	+	-	-
Varibaculum_sp._Marseille-P2802	+	-	-
Pseudomonas_phage_PhiPA3	+	-	-
Buchnera_aphidicola	+	-	-
Staphylococcus_hyicus	+	-	-
Thalassomonas_actiniarum	+	-	-
Staphylococcus_rostri	+	-	-
Bradyrhizobium_sp._S23321	+	-	-
Vibrio_mexicanus	+	-	-
Streptococcus_sp._HMSC070B10	+	-	-
Bacillus_sp._HBCD-sjtu	+	-	-
Streptococcus_sp._HMSC065E03	+	-	-
Vibrio_owensii	+	-	-
Bacillus_sp._KQ-3	+	-	-
Acinetobacter_baylyi	+	-	-
Streptococcus_sp._X13SY08	+	-	-
candidate_division_WWE3_bacterium_RIFCSPLOWO2_01_FU	+	-	-
Tenacibaculum_sp._MAR_2010_89	+	-	-
Pseudoalteromonas_byunsanensis	+	-	-
Bacteroidetes_bacterium_4572_77	+	-	-
Bacteroidetes_bacterium_47-18	+	-	-
Rummeliibacillus_pycnus	+	-	-
Pseudoxanthomonas_sp._GSS15	+	-	-
Thalassomonas_viridans	+	-	-
Winogradskyella_sp._KYW1333	+	-	-
Verrucomicrobia_bacterium_LW23	+	-	-
Burkholderia_sp._KK1	+	-	-
Burkholderia_seminalis	+	-	-
Alphaproteobacteria_bacterium_CG_4_10_14_0_2_um_filter_6	+	-	-
Aureimonas_sp._AU40	+	-	-
Burkholderiales_bacterium_66-5	+	-	-
Bifidobacteriaceae_bacterium_NR047	+	-	-
Xanthomonadales_bacterium_63-13	+	-	-
delta_proteobacterium_PSCGC_5451	+	-	-
Burkholderiales_bacterium_RIFCSPLOWO2_12_FULL_64_33	+	-	-
Thauera_linaloolentis	+	-	-
Candidatus_Sulfotelmatomonas_gaucii	+	-	-
Kushneria_indalinina	+	-	-
Colwellia_mytili	+	-	-
Lactobacillus_sp._54-5	+	-	-
OCS116_cluster_bacterium	+	-	-
Oceanicella_actignis	+	-	-
Lactobacillus_rapi	+	-	-
Lactobacillus_raoultii	+	-	-
Candidatus_Uhrbacteria_bacterium_RIFCSPLOWO2_02_FULL	+	-	-
Oceanihabitans_sediminis	+	-	-
Lactobacillus_kunkeei	+	-	-
Ochrobactrum_haematophilum	+	-	-
Deinococcus_frigens	+	-	-
Hydrogenophaga_crassostreae	+	-	-
Pandoraea_sp._ISTKB	+	-	-
Hippea_sp._KM1	+	-	-

Candidatus_Kerfeldbacteria_bacterium_RIFCSPLOWO2_02_FU	+	-	-
Candidatus_Peregrinibacteria_bacterium_CG11_big_fil_rev_8_2	+	-	-
Deltaproteobacteria_bacterium_HGW-Deltaproteobacteria-1	+	-	-
Candidatus_Nitrospira_nitrosa	+	-	-
Candidatus_Nealsonbacteria_bacterium_CG_4_9_14_3_um_filte	+	-	-
Lactobacillus_thailandensis	+	-	-
Myxococcales_bacterium_68-20	+	-	-
Methanothrix_sp.	+	-	-
Mesosutterella_multiformis	+	-	-
Mucilaginibacter_sp._HYN0043	+	-	-
Chryseobacterium_sp._CH21	+	-	-
Mucilaginibacter_sp._MD40	+	-	-
Mycoplasma_alvi	+	-	-
Moraxella_sp._RCAD0137	+	-	-
Moraxella_caprae	+	-	-
Micromonospora_sp._CNZ309	+	-	-
Micromonospora_halophytica	+	-	-
Chryseobacterium_antarcticum	+	-	-
Mesorhizobium_tamadayense	+	-	-
Lysinimicrobium_iriomotense	+	-	-
Lewinella_persica	+	-	-
Leptospira_biflexa	+	-	-
Nitrospirae_bacterium_RBG_16_64_22	+	-	-
Lawsonella_clevelandensis	+	-	-
Cohnella_sp._SGD-V74	+	-	-
Lactobacilus_nuruki	+	-	-
Nocardiopsis_sp._JB363	+	-	-
Lactobacillus_virus_Ld25A	+	-	-
Clostridium_sp._AM54-14XD	+	-	-
Neorhizobium_galegae	+	-	-
Marivirga_sericea	+	-	-
Malassezia_vespertilionis	+	-	-
Frankia_sp._ACN1ag	+	-	-
Gilvimarinus_chinensis	+	-	-
Pedobacter_suwonensis	+	-	-
Candidatus_Falkowbacteria_bacterium_HGW-Falkowbacteria-1	+	-	-
Pelagibacteraceae_bacterium_TMED232	+	-	-
Enterovibrio_sp._CAIM_600	+	-	-
Fictibacillus_aquaticus	+	-	-
Phycisphaera_sp._TMED9	+	-	-
Fischerella_major	+	-	-
Candidatus_Cloacimonetes_bacterium_HGW-Cloacimonetes-1	+	-	-
Enterococcus_sp._8G7_MSG3316	+	-	-
Hahella_sp._CCB-MM4	+	-	-
Pseudobacteriovorax_antillogorgiicola	+	-	-
Oceanobacillus_arenosus	+	-	-
Candidatus_Staskawiczbacteria_bacterium_RIFCSPHIGHO2_01	+	-	-
Azorhizobium_caulinodans	+	-	-
Chlorobium_sp._KB01	+	-	-
Alphaproteobacteria_bacterium_MarineAlpha11_Bin1	+	-	-
Brevundimonas_abyssalis	+	-	-
Aspergillus_nomius	+	-	-
Campylobacter_sp._RM16704	+	-	-
Elusimicrobia_bacterium_RIFOXYD2_FULL_34_15	+	-	-
'Sphingomonas_ginsengisoli'_Hoang_et_al._2012	+	-	-

Parcubacteria_group_bacterium_GW2011_GWC2_39_14	+	-	-
Salipiger_marinus	+	-	-
Polymorphum_gilvum	+	-	-
Betaproteobacteria_bacterium	+	-	-
Hyphomonas_atlantica	+	-	-
Sulfitobacter_noctilucae	+	-	-
Hoeflea_sp._BAL378	+	-	-
Pseudoalteromonas_sp._T1lg75	+	-	-
Roseibacterium_elongatum	+	-	-
Burkholderiales_bacterium_GJ-E10	+	-	-
Bifidobacterium_scaligerum	+	-	-
Rhodospirillaceae_bacterium_HHTR118	+	-	-
Zhengella_mangrovi	+	-	-
Caballeronia_pedi	+	-	-
Parcubacteria_group_bacterium_CG1_02_37_51	+	-	-
Shigella_boydii	+	-	-
Salinisphaera_sp._LB1	+	-	-
Steroidobacter_denitrificans	+	-	-
Terasakiella_pusilla	+	-	-
Caulobacter_sp._Root1455	+	-	-
Oceanibaculum_nanhaiense	+	-	-
Acetobacter_aceti	+	-	-
Dehalobacter_sp._DCA	+	-	-
Ensifer_sp._Root423	+	-	-
Alphaproteobacteria_bacterium_MarineAlpha5_Bin7	+	-	-
Thalassospira_xianhensis	+	-	-
Maricaulis_salignorans	+	-	-
Rhodobacteraceae_bacterium_MA-7-27	+	-	-
Candidatus_Peribacteria_bacterium_RIFCSPHIGHO2_01_FULl	+	-	-
Betaproteobacteria_bacterium_RIFCSPLOWO2_12_FULL_62_	+	-	-
Candidatus_Nealsonbacteria_bacterium_CG08_land_8_20_14_(	+	-	-
Thermoplasmatales_archaeon_SM1-50	+	-	-
Thermodesulfovibrio_islandicus	+	-	-
Neomegalonema_perideroedes	+	-	-
Pelagibacterium_montanilacus	+	-	-
alpha_proteobacterium_AAP38	+	-	-
Zymobacter_palmae	+	-	-
Sanghuangporus_baumii	+	-	-
Aliagarivorans_taiwanensis	+	-	-
Rhodoblastus_acidophilus	+	-	-
Desulfurococcaceae_archaeon_AG1	+	-	-
Hahella_chejuensis	+	-	-
Agaricus_bisporus	+	-	-
Bacteroides_sp._Ga6A1	+	-	-
Metschnikowia_bicuspidata	+	-	-
Nostoc_sp._ATCC_43529	+	-	-
Candidatus_Yanofskybacteria_bacterium_RIFOXYA1_FULL_4	+	-	-
Alphaproteobacteria_bacterium_BRH_c36	+	-	-
Candidatus_Endomicrobium_trichonymphae	+	-	-
Fibrella_sp._ES10-3-2-2	+	-	-
Kosmotoga_sp._DU53	+	-	-
Enterococcus_sp._HMSC05C03	+	-	-
Caedibacter_sp._37-49	+	-	-
Lomentospora_prolificans	+	-	-
Floricoccus_tropicus	+	-	-

Marinitoga_sp._1197	+	-	-
Scardovia_inopinata	+	-	-
Blastococcus_sp._DSM_44268	+	-	-
Bifidobacterium_margollesii	+	-	-
Candidatus_Woesebacteria_bacterium_RIFCSPHIGHO2_01_FU	+	-	-
Rhodospirillum_centenum	+	-	-
Rhodoplanes_sp._Z2-YC6860	+	-	-
Catenovulum_agarivorans	+	-	-
Roseiarcus_fermentans	+	-	-
Verticillium_nonalfalfae	+	-	-
Lactobacillus_sp._151-2B	+	-	-
Methylobacter_sp._KRF1	+	-	-
Marinicauda_sp._WD6-1	+	-	-
Halobacillus_salinus	+	-	-
Alicyclobacillus_sendaiensis	+	-	-
Undibacterium_pigrum	+	-	-
Lactobacillus_namurensis	+	-	-
Marinobacter_sp._ES-1	+	-	-
Magnetospirillum_sp._ME-1	+	-	-
Brevundimonas_sp._LM2	+	-	-
Comamonas_terrigena	+	-	-
uncultured_bacterium_Contig1552	+	-	-
Bacillus_butanolivorans	+	-	-
Herbaspirillum_sp._HC18	+	-	-
Sphingomonas_echinoides	+	-	-
Serendipita_indica	+	-	-
Xylanimonas_cellulosilytica	+	-	-
Gallionellales_bacterium_CG_4_10_14_3_um_filter_54_96	+	-	-
Brettanomyces_naardenensis	+	-	-
Oceanicoccus_sp._KOV_DT_Chl	+	-	-
Streptomyces_sp._FxanaD5	+	-	-
Flavobacterium_aquidurense	+	-	-
Streptomyces_sp._CNT318	+	-	-
Gulosibacter_sp._Marseille-P7157	+	-	-
Bacillus_phage_Phrodo	+	-	-
Streptomyces_sp._CMB-StM0423	+	-	-
Gulbenkiania_indica	+	-	-
Streptomyces_sp._OTB305	+	-	-
gamma_proteobacterium_symbiont_of_Ctena_orbiculata	+	-	-
Flavobacteriaceae_bacterium_144Ye	+	-	-
Methanococcoides_vulcani	+	-	-
Methanobacterium_bryantii	+	-	-
Methanobacteriales_archaeon_HGW-Methanobacteriales-1	+	-	-
Enterococcus_sp._3C8_DIV0646	+	-	-
Bacillus_sp._171095_106	+	-	-
Bacillus_sp._166amftsu	+	-	-
Enterococcus_rotai	+	-	-
Mesorhizobium_sp._LNJC384A00	+	-	-
candidate_division_Zixibacteria_bacterium_RBG_16_53_22	+	-	-
Limnohabitans_sp._T6-5	+	-	-
Flavobacterium_sp._Leaf82	+	-	-
Halorubrum_sp._ZC67	+	-	-
Leuconostoc_sp._C2	+	-	-
Leuconostoc_lactis	+	-	-
Granulicella_rosea	+	-	-

Algoriphagus_boritolerans	+	-	-
Leptospira_meyeri	+	-	-
Talaromyces_atroroseus	+	-	-
Talaromyces_stipitatus	+	-	-
Helicobacter_himalayensis	+	-	-
Lentibacillus_sp._Marseille-P4043	+	-	-
Listeria_fleischmannii	+	-	-
Enterococcus_phage_156	+	-	-
Streptomyces_avicenniae	+	-	-
Acidovorax_caeni	+	-	-
Mannheimia_sp._USDA-ARS-USMARC-1261	+	-	-
Flavobacterium_limnosediminis	+	-	-
Sulfuricurvum_sp._RIFCSPLOWO2_12_FULL_43_24	+	-	-
Halorhabdus_tiamatea	+	-	-
Flavobacterium_sp._HYN0086	+	-	-
Luteipulveratus_mongoliensis	+	-	-
Moraxella_boevrei	+	-	-
Sporolactobacillus_terrae	+	-	-
Mobiluncus_mulieris	+	-	-
Mobilicoccus_pelagius	+	-	-
Microvirga_ossetica	+	-	-
Erwinia_virus_Asesino	+	-	-
Microgenomates_group_bacterium_GW2011_GWA2_39_19	+	-	-
Staphylococcus_phage_vB_SauM_0414_108	+	-	-
Moraxellaceae_bacterium_HYN0046	+	-	-
Mycoavidus_cysteinexigens	+	-	-
Yersinia_pseudotuberculosis	+	-	-
Muricauda_sp._TMED12	+	-	-
Spirochaetes_bacterium_RBG_13_51_14	+	-	-
Citrobacter_portucalensis	+	-	-
uncultured_bacterium_r_02	+	-	-
Fictibacillus_sp._S7	+	-	-
uncultured_bacterium_Contig14	+	-	-
Streptococcus_sp._400_SSPC	+	-	-
Clostridium_phage_CDSH1	+	-	-
Clostridium_phage_c-st	+	-	-
Streptococcus_sp._UMB1385	+	-	-
Streptococcus_sp._oral_taxon_058	+	-	-
Flavihumibacter_sp._CACIAM_22H1	+	-	-
Methyloligella_halotolerans	+	-	-
uncultured_bacterium_Contig52	+	-	-
Bacillus_sp._NSP22.2	+	-	-
Microbacterium_sp._cl127	+	-	-
Microbacterium_sp._cf046	+	-	-
Fervidobacterium_sp._SC_NGM5_G05	+	-	-
Microbacterium_sp._Leaf203	+	-	-
Microbacterium_pygmaeum	+	-	-
Microbacterium_phyllosphaerae	+	-	-
Bacillus_sp._K2I17	+	-	-
Methylotenera_sp._RIFCSPLOWO2_02_FULL_45_14	+	-	-
Methylophaga_muralis	+	-	-
Legionella_beliardensis	+	-	-
Alphaproteobacteria_bacterium_MarineAlpha5_Bin2	+	-	-
Gemmatimonas_sp._SG8_28	+	-	-
Actinoplanes_lutulentus	+	-	-

Kaumoebavirus	+	-	-
Valsa_sordida	+	-	-
Geobacillus_sp._Y4.1MC1	+	-	-
Aerococcus_urinaehominis	+	-	-
Trametes_pubescens	+	-	-
Cyanothece_sp._PCC_8802	+	-	-
Thiobacillus_sp._SCN_63-57	+	-	-
Gammaproteobacteria_bacterium_TMED112	+	-	-
Gammaproteobacteria_bacterium_TMED183	+	-	-
Crocinitomix_sp._MedPE-SWsnd	+	-	-
Kurthia_gibsonii	+	-	-
Cupriavidus_plantarum	+	-	-
Cupriavidus_sp._S23	+	-	-
Curtobacterium_sp._ER1/6	+	-	-
Gemmatimonadetes_bacterium_13_1_40CM_4_69_8	+	-	-
Veillonella_sp._3310	+	-	-
Ilumatobacter_coccineus	+	-	-
Ignavibacterium_album	+	-	-
Gilliamella_apis	+	-	-
Ignavibacteria_bacterium_GWA2_36_19	+	-	-
Vibrio_sinaloensis	+	-	-
Glaciecola_sp._THG-3.7	+	-	-
Idiomarina_sp._H105	+	-	-
Vibrio_sp._J2-31	+	-	-
Intrasporangium_chromatireducens	+	-	-
Veillonella_sp._ICM51a	+	-	-
Anaerolinea_sp._4484_236	+	-	-
Janthinobacterium_sp._CG3	+	-	-
Altererythrobacter_sp._AY-3R	+	-	-
Vibrio_metschnikovii	+	-	-
Coxiella_sp._DG_40	+	-	-
Collinsella_sp._AM34-10	+	-	-
Hippea_maritima	+	-	-
Vitreoscilla_filiformis	+	-	-
Actinobacteria_bacterium_HGW-Actinobacteria-10	+	-	-
Collinsella_sp._TF10-11AT	+	-	-
Thaumasiovibrio_subtropicus	+	-	-
Hydrogenovibrio_sp._Milos-T1	+	-	-
Collinsella_sp._TM05-38	+	-	-
Elizabethkingia_bruuniana	+	-	-
Actinobacteria_bacterium_IMCC19121	+	-	-
Asticcacaulis_sp._AC460	+	-	-
Lactobacillus_sp._HBUAS52074	+	-	-
Colwellia_chukchiensis	+	-	-
Thauera_aromatica	+	-	-
Weissella_kandleri	+	-	-
Leclercia_adecarboxylata	+	-	-
Vulgatibacter_incomptus	+	-	-
Lacunisphaera_sp._TWA-58	+	-	-
Collinsella_sp._4_8_47FAA	+	-	-
Collinsella_sp._AF31-11	+	-	-
Herbaspirillum_sp._RV1423	+	-	-
Herbiconiux_sp._YR403	+	-	-
Lactobacillus_buchneri	+	-	-
Lactobacillus_bombicola	+	-	-

Lactobacillus_bambusae	+	-	-
Hyaloscypha_bicolor	+	-	-
Thermonema_rossianum	+	-	-
Arcobacter_sp._CECT_9188	+	-	-
Arcobacter_sp._AF1028	+	-	-
Gallionellaceae_bacterium	+	-	-
Actinomyces_sp._oral_taxon_848	+	-	-
Corallococcus_sp._CA054A	+	-	-
Corynebacterium_genitalium	+	-	-
Gaiella_sp._SCGC_AG-212-M14	+	-	-
Arthrobacter_sp._NEAU-SA2	+	-	-
Actinomadura_oligospora	+	-	-
Alcanivorax_borkumensis	+	-	-
Arthrobacter_sp._B1805	+	-	-
Gaetbulibacter_saemankumensis	+	-	-
Lactobacillus_odoratitofui	+	-	-
Lactobacillus_malefermentans	+	-	-
Thermodesulfovibrio_sp._RBG_19FT_COMBO_41_18	+	-	-
Lactobacillus_sp._OTU4228	+	-	-
Pseudomonas_caspiana	+	-	-
Bacteroidetes_bacterium_MedPE-SWsnd-G2	+	-	-
Nitrospirae_bacterium_RBG_13_43_8	+	-	-
Robiginitalea_myxolifaciens	+	-	-
Nitrospirae_bacterium_GWB2_47_37	+	-	-
Roseibacillus_sp._TMED18	+	-	-
Roseimicrobium_gellanilyticum	+	-	-
Pseudogymnoascus_sp._VKM_F-4281_(FW-2241)	+	-	-
Rickettsia_bellii	+	-	-
Rhodopirellula_maiorica	+	-	-
Paraglaciecola_polaris	+	-	-
Candidatus_Kaiserbacteria_bacterium_RIFCSPHIGHO2_02_FU	+	-	-
Brevundimonas_subvibrioides	+	-	-
Pseudanabaena_sp._SR411	+	-	-
Bacteroidetes/Chlorobi_group_bacterium_Naka2016	+	-	-
Candidatus_Electrothrix_communis	+	-	-
SAR116_cluster_alpha_proteobacterium_HIMB100	+	-	-
Pedobacter_glucosidilyticus	+	-	-
Pseudoalteromonas_atlantica	+	-	-
Pedobacter_sp._AJM	+	-	-
Candidatus_Daviesbacteria_bacterium_RIFCSPHIGHO2_01_FU	+	-	-
Propionibacteriales_bacterium	+	-	-
Neisseria_zoodegmatis	+	-	-
Neisseria_wadsworthii	+	-	-
Neisseria_sp._HMSC069H12	+	-	-
Pseudoalteromonas_phenolica	+	-	-
Bacteroidetes_bacterium_GWF2_39_10	+	-	-
Rothia_sp._HMSC071F11	+	-	-
Buttiauxella_gaviniae	+	-	-
Rothia_sp._HSID18067	+	-	-
Rous_sarcoma_virus	+	-	-
Bacteroidetes_bacterium_46-16	+	-	-
Cercospora_zeina	+	-	-
Candidatus_Firestonebacteria_bacterium_GWA2_43_8	+	-	-
Candidatus_Yanofskybacteria_bacterium_GW2011_GWA2_44_	+	-	-
Olsenella_sp._AF21-51	+	-	-

Omnitrophica_bacterium_OLB16	+	-	-
Rhizobium_album	+	-	-
Pseudoxanthomonas_sp._CF385	+	-	-
Omnitrophica_WOR_2_bacterium_SM23_29	+	-	-
Paenibacillus_sp._LK1	+	-	-
Candidatus_Nomurabacteria_bacterium_GW2011_GWF1_34_2(	+	-	-
Rhizobium_sp._CF097	+	-	-
Pseudonocardiaceae_bacterium	+	-	-
Bifidobacterium_sp.	+	-	-
Paenibacillus_sp._RUD330	+	-	-
Candidatus_Solibacter_usitatus	+	-	-
Okeania_hirsuta	+	-	-
Candidatus_Peregrinibacteria_bacterium_HGW-Peregrinibacteri	+	-	-
Pyrinomonas_methylaliphatogenes	+	-	-
Candidatus_Pelagibacter_sp._IMCC9063	+	-	-
Candidatus_Pacebacteria_bacterium_CG1_02_43_31	+	-	-
Bradyrhizobium_sp._STM_3809	+	-	-
Bradyrhizobium_sp._Leo121	+	-	-
Paraburkholderia_soli	+	-	-
Candidatus_Woesebacteria_bacterium_GW2011_GWB1_38_8	+	-	-
Brettanomyces_bruxellensis	+	-	-
Candidatus_Woesebacteria_bacterium_RBG_13_36_22	+	-	-
Pseudomonas_sp._F51	+	-	-
Bradyrhizobium_sp._ARR65	+	-	-
Rhodanobacteraceae_bacterium	+	-	-
Pseudomonas_vancouverensis	+	-	-
Rhodobacter_capsulatus	+	-	-
Rhodobacteraceae_bacterium_63075	+	-	-
Candidatus_Termititenax_dinenymphae	+	-	-
Paenisporosarcina_sp._K2R23-3	+	-	-
Bradyrhizobium_liaoningense	+	-	-
Salinarchaeum_sp._Harcht-Bsk1	+	-	-
Sodalis_sp._TME1	+	-	-
Pontibacter_mucosus	+	-	-
Shinella_sp._JR1-6	+	-	-
Candidatus_Beckwithbacteria_bacterium_RBG_13_42_9	+	-	-
Prevotella_sp._HMSC077E08	+	-	-
Porphyromonas_sp._KLE_1280	+	-	-
Sanguibacter_gelidistatuariae	+	-	-
Polynucleobacter_sp._35-46-207	+	-	-
Chromobacterium_sp._MWU14-2602	+	-	-
Candida_dubliniensis	+	-	-
Planktothrix_sp._PCC_11201	+	-	-
Sphingobacteriia_bacterium_RIFOXYC2_FULL_35_18	+	-	-
Chloroflexi_bacterium_RBG_13_51_52	+	-	-
Campylobacteraceae_bacterium_4484_166	+	-	-
Mycoplasma_phocirhinis	+	-	-
Chromobacterium_amazonense	+	-	-
Candidatus_Chloroploca_sp._Khr17	+	-	-
Candidatus_Berkelbacteria_bacterium_RIFCSPHIGHO2_01_FU	+	-	-
Sinobacteraceae_bacterium_GT1R17	+	-	-
Candidatus_Altiarchaeales_archaeon_HGW-Altiarchaeales-2	+	-	-
Chlorogloea_sp._CCALA_695	+	-	-
Planctomycetes_bacterium_RBG_16_64_12	+	-	-
Neisseria_sp.	+	-	-

Campylobacter_sp._P091	+	-	-
Sphingomonas_sp._STIS6.2	+	-	-
Campylobacter_iguaniorum	+	-	-
Candidatus_Actinomarinales_bacterium	+	-	-
Chryseobacterium_sp._CF356	+	-	-
Candidatus_Aenigmarchaeota_archaeon	+	-	-
Mesorhizobium_australicum	+	-	-
Human_endogenous_retrovirus	+	-	-
Rickettsiales_bacterium	+	-	-
Suttonella_ornithocola	+	-	-
Hesseltinella_vesiculosa	+	-	-
Geobacter_sp._OR-1	+	-	-
Rhodotorula_graminis	+	-	-
Rhodospirillaceae_bacterium_Gri0909	+	-	-
Rhodospirillaceae_bacterium_SYSU_D60014	+	-	-
Candidatus_Endolissoclinum_sp._TMED55	+	-	-
Fibrobacter_sp._UWP2	+	-	-
bacterium_BMS3Abin15	+	-	-
Sphingomonas_laterariae	+	-	-
Hylemonella_gracilis	+	-	-
Beijerinckia_mobilis	+	-	-
Bacillus_daliensis	+	-	-
Tistrella_mobilis	+	-	-
Bifidobacterium_simiarum	+	-	-
Cryptococcus_neoformans	+	-	-
Lachancea_thermotolerans	+	-	-
Marichromatium_sp._AB32	+	-	-
Robiginitomaculum_antarcticum	+	-	-
Serratia_rubidaea	+	-	-
Phenylobacterium_zucineum	+	-	-
Thalassococcus_sp._WRAS1	+	-	-
Sphingomonas_sp._K2R01-6	+	-	-
Candidatus_Terasakiella_magnetica	+	-	-
Ornithinimicrobium_sp._AMA3305	+	-	-
Bdellovibrionales_bacterium_CG22_combo_CG10-13_8_21_14	+	-	-
Caulobacteraceae_bacterium	+	-	-
Parasphingopyxis_lamellibrachiae	+	-	-
Flavobacterium_seoulense	+	-	-
Elstera_cyanobacteriorum	+	-	-
Altererythrobacter_sp._ZODW24	+	-	-
Bdellovibrio_sp._ArHS	+	-	-
Salegentibacter_echinorum	+	-	-
Kurthia_sp._3B1D	+	-	-
Roseobacter_sp._GAI101	+	-	-
Acidisphaera_rubrifaciens	+	-	-
Parvibium_lacunae	+	-	-
Chloroflexi_bacterium_OLB15	+	-	-
Alphaproteobacteria_bacterium_HGW-Alphaproteobacteria-12	+	-	-
Mesorhizobium_sp._WSM4313	+	-	-
Bradyrhizobiaceae_bacterium	+	-	-
Tateyamaria_omphalii	+	-	-
Anoxybacillus_mongoliensis	+	-	-
Thiomicrospira_sp.	+	-	-
Epibacterium_mobile	+	-	-
Opitutaceae_bacterium_BACL24_MAG-120322-bin51	+	-	-

Parvularcula_oceani	+	-	-
Flavobacterium_aquaticum	+	-	-
Oceanicaulis_sp._HTCC2633	+	-	-
Thermobacillus_sp._ZCTH02-B1	+	-	-
Alkalibacterium_gilvum	+	-	-
Magnetospirillum_sp.	+	-	-
Mannheimia_granulomatis	+	-	-
Synechococcus_sp._Baikal-G1	+	-	-
Alphaproteobacteria_bacterium_HGW-Alphaproteobacteria-3	+	-	-
Kuraishia_capsulata	+	-	-
Brevibacillus_invocatus	+	-	-
Lactobacillus_versmoldensis	+	-	-
Flammeovirga_sp._L12M1	+	-	-
Leptolyngbya_foveolarum	+	-	-
[Candida]_arabinofermentans	+	-	-
Hydnomerulius_pinastri	+	-	-
Agitococcus_lubricus	+	-	-
Streptomyces_ochraceiscleroticus	+	-	-
Thioalkalivibrio_sp._ALSr1	+	-	-
Planctomycetes_bacterium_TMED75	+	-	-
Bifidobacterium_myosotis	+	-	-
Schaalia_cardiffensis	+	-	-
Bifidobacterium_psychraerophilum	+	-	-
Bordetella_sp._J329	+	-	-
Streptococcus_halotolerans	+	-	-
Pseudaestuariivita_atlantica	+	-	-
Thermoactinomyces_sp._DSM_45892	+	-	-
Candidatus_Methanoperedens_sp._BLZ2	+	-	-
Hymenobacter_swuensis	+	-	-
Nitrospinae_bacterium_RIFCSPHIGHO2_12_FULL_39_42	+	-	-
Leptotrichia_sp.	+	-	-
Marinobacter_vinifirmus	+	-	-
Pichia_kudriavzevii	+	-	-
Eutypa_lata	+	-	-
Devosia_lucknowensis	+	-	-
Devosia_psychrophila	+	-	-
Exidia_glandulosa	+	-	-
Zymoseptoria_tritici	+	-	-
Azoarcus_sp._PA01	+	-	-
Lactobacillus_sp._143-1	+	-	-
Candida_viswanathii	+	-	-
Desulfobacterales_bacterium_S5133MH16	+	-	-
Hydrogenophaga_sp._Root209	+	-	-
Hydrogenophilales_bacterium_17-62-8	+	-	-
Bradyrhizobium_sp._SK17	+	-	-
Flavimaricola_marinus	+	-	-
Xanthomonadaceae_bacterium	+	-	-
Bradyrhizobium_sp._WSM2793	+	-	-
Candidatus_Magasanikbacteria_bacterium_CG_4_10_14_0_2_u	+	-	-
Halomonas_sp._WRN001	+	-	-
Helicobacter_valdiviensis	+	-	-
Candidatus_Amesbacteria_bacterium_GW2011_GWA2_47_11	+	-	-
uncultured_Rhodococcus_sp.	+	-	-
Herbaspirillum_frisingense	+	-	-
Henriciella_sp.	+	-	-

Candidatus_Moranbacteria_bacterium_GW2011_GWA2_39_41	+	-	-
Violaceomyces_palustris	+	-	-
Anaerococcus_sp._HMSC068A02	+	-	-
Jannaschia_helgolandensis	+	-	-
Candidatus_Omnitrophica_bacterium_CG11_big_fil_rev_8_21_	+	-	-
Vibrio_orientalis	+	-	-
Candidatus_Odyssella_thessalonicensis	+	-	-
Candidatus_Pacebacteria_bacterium_RIFCSPLOWO2_01_FULl	+	-	-
Demequina_aestuarii	+	-	-
Jeongeupia_sp._USM3	+	-	-
uncultured_bacterium_fosmid_pJB77G10	+	-	-
Altererythrobacter_sp._Root672	+	-	-
Alteromonadaceae_bacterium_2052S.S.stab0a.01	+	-	-
Planococcus_salinarum	+	-	-
Photorhabdus_namnaonensis	+	-	-
Hymenobacter_sp._APR13	+	-	-
Idiomarina_aestuarii	+	-	-
Pseudophaeobacter_arcticus	+	-	-
uncultured_bacterium_Contigcl_1738	+	-	-
Vibrio_phage_1.187.O._10N.286.49.F1	+	-	-
Pseudonocardia_spinosispora	+	-	-
Vibrio_scophthalmi	+	-	-
Ignatzschineria_indica	+	-	-
Phaeoacremonium_minimum	+	-	-
marine_gamma_proteobacterium_HTCC2080	+	-	-
Acinetobacter_defluvii	+	-	-
Gammaproteobacteria_bacterium_TMED180	+	-	-
Flavobacterium_sp._LB2P30	+	-	-
Pseudoalteromonas_sp._GCY	+	-	-
Acinetobacter_sp._38-8	+	-	-
Actinoplanes_regularis	+	-	-
Actinoplanes_missouriensis	+	-	-
Acinetobacter_sp._ANC_3862	+	-	-
candidate_division_Zixibacteria_bacterium_RBG_16_48_11	+	-	-
Aeromonas_simiae	+	-	-
Aeromonas_piscicola	+	-	-
Acidovorax_sp._JHL-9	+	-	-
Parcubacteria_group_bacterium_GW2011_GWA2_42_14	+	-	-
Acidovorax_sp._RAC01	+	-	-
Parcubacteria_group_bacterium_GW2011_GWA2_46_10	+	-	-
Aeromicrobium_sp._592	+	-	-
Candidatus_Buchananbacteria_bacterium_CG10_big_fil_rev_8_	+	-	-
Polyporus_brumalis	+	-	-
Aerococcus_sp._HMSC062B07	+	-	-
Geobacillus_lituanicus	+	-	-
Pseudocercospora_eumusae	+	-	-
candidate_division_Zixibacteria_bacterium_RBG_16_40_9	+	-	-
Gammaproteobacteria_bacterium_CG_4_10_14_0_8_um_filter_	+	-	-
Candidatus_Cloacimonetes_bacterium_HGW-Cloacimonetes-3	+	-	-
Acinetobacter_sp._ANC_5318	+	-	-
Promicromonospora_sp._AC04	+	-	-
Candidatus_Daviesbacteria_bacterium_RIFCSPHIGHO2_02_FU	+	-	-
Propionimicrobium_sp._BV2F7	+	-	-
Actinobacteria_bacterium_IMCC26256	+	-	-
Fortiea_contorta	+	-	-

Candidatus_Dadabacteria_bacterium_RIFCSPHIGHO2_12_FUI	+	-	-
Acinetobacter_sp._WCHAc060096	+	-	-
Candidatus_Daviesbacteria_bacterium_GW2011_GWB1_41_5	+	-	-
Frateuria_terrea	+	-	-
Actinomyces_succiniciruminis	+	-	-
Actinomyces_sp._HMSC075B09	+	-	-
Acinetobacter_sp._MB5	+	-	-
Pedobacter_chitinilyticus	+	-	-
Fulvivirga_imtechensis	+	-	-
candidate_division_NC10_bacterium	+	-	-
Elusimicrobia_bacterium_RIFCSPLOWO2_01_FULL_60_11	+	-	-
Flavobacterium_limicola	+	-	-
Acidithiobacillales_bacterium_SG8_45	+	-	-
Haemophilus_pittmaniae	+	-	-
Brevibacterium_sp._HMSC24B04	+	-	-
Acidobacteria_bacterium_13_1_20CM_58_21	+	-	-
Paraburkholderia_susongensis	+	-	-
marine_bacterium_AO1-C	+	-	-
Pleomorphomonas_oryzae	+	-	-
Yaniella_halotolerans	+	-	-
Candidatus_Korarchaeum_cryptofilum	+	-	-
Brevundimonas_sp._Leaf280	+	-	-
Pseudomonas_acidophila	+	-	-
Candidatus_Harrisonbacteria_bacterium_CG10_big_fil_rev_8_2	+	-	-
Candidatus_Azambacteria_bacterium_GW2011_GWC1_46_13	+	-	-
Polaromonas_sp._AER18D-145	+	-	-
Gordonia_sihwensis	+	-	-
candidate_division_Zixibacteria_bacterium_RBG_16_50_21	+	-	-
Pseudomonas_amygdali	+	-	-
Candidatus_Jorgensenbacteria_bacterium_GW2011_GWF2_41_	+	-	-
Alcanivorax_sp._Nap_24	+	-	-
Desulfuromonadales_bacterium_C00003093	+	-	-
Alcanivorax_sp._MD8A	+	-	-
Peptoniphilus_senegalensis	+	-	-
[Propionibacterium]_namnetense	+	-	-
Gracilibacillus_timonensis	+	-	-
Gracilibacillus_massiliensis	+	-	-
Devosia_elaeis	+	-	-
Afifella_sp._JA968	+	-	-
Deinococcus_planocerae	+	-	-
Marinomonas_phage_P12026	+	-	-
Marinococcus_luteus	+	-	-
Marinobacter_sp._C1S70	+	-	-
Marinitoga_sp._4572_148	+	-	-
Ruminococcus_sp._CAG:55	+	-	-
Chitinophaga_sp._YR627	+	-	-
Streptomyces_sp._4121.5	+	-	-
Planctomyces_sp._SH-PL62	+	-	-
Marixanthomonas_ophiurae	+	-	-
Nesterenkonia_alba	+	-	-
Chitinophaga_costaii	+	-	-
Rubrivirga_sp._SAORIC476	+	-	-
Bacteroidetes_bacterium_QH_2_64_74	+	-	-
Cobetia_amphilecti	+	-	-
Rickettsia_massiliae	+	-	-

Nocardia_flavorosea	+	-	-
Latescibacteria_bacterium_DG_63	+	-	-
Cohnella_sp._OV312	+	-	-
Thalassobacillus_sp._C254	+	-	-
Nonlabens_sp._1Q3	+	-	-
Bacillus_aerophilus	+	-	-
Sulfurospirillum_sp._UBA12182	+	-	-
Cellulomonas_sp._73-92	+	-	-
Lewinella_sp._IMCC34183	+	-	-
Nitrospira_bacterium_SG8_35_1	+	-	-
Synechococcus_sp._MW101C3	+	-	-
Leucobacter_salsicius	+	-	-
Syntrophorhabdus_sp._PtaU1.Bin050	+	-	-
Caulobacteraceae_bacterium_PMMR1	+	-	-
Leptolyngbya_sp._PCC_7376	+	-	-
Leptolyngbya_frigida	+	-	-
Bacteroidetes_bacterium_MedPE-SWsnd-G1	+	-	-
Neisseria_sp._HMSC072F04	+	-	-
Bacteroides_sp._3_2_5	+	-	-
Microvirga_subterranea	+	-	-
Sphaerulina_musiva	+	-	-
Stanieria_cyanosphaera	+	-	-
Spartobacteria_bacterium_AMD-G5	+	-	-
Sorangiineae_bacterium_NIC37A_2	+	-	-
Solirubrobacter_sp._URHD0082	+	-	-
Mycoplasma_glycophilum	+	-	-
Chroococcales_cyanobacterium_metabat2.561	+	-	-
Miniimonas_sp._PCH200	+	-	-
Mycobacterium_sp._852014-50255_SCH5639931	+	-	-
Methanoregula_boonei	+	-	-
Streptomyces_alboflavus	+	-	-
Salsuginibacillus_halophilus	+	-	-
Necropsobacter_rosorum	+	-	-
Methanobacterium_sp._SMA-27	+	-	-
Salinivenus_iranica	+	-	-
Streptomyces_hygroscopicus	+	-	-
Metakosakonia_sp._MRY16-398	+	-	-
Bacillus_sp._522_BSPC	+	-	-
Scheffersomyces_stipitis	+	-	-
Methylocystis_sp._ATCC_49242	+	-	-
Chromobacterium_subtsugae	+	-	-
Silvanigrella_aquatica	+	-	-
Microbacterium_phage_Camille	+	-	-
Methyloversatilis_sp._RAC08	+	-	-
Methylophaga_frappieri	+	-	-
Sediminibacterium_sp._C3	+	-	-
Streptococcus_sp._HMSC034B05	+	-	-
Naematelia_encephala	+	-	-
Bacillus_sp._FJAT-27231	+	-	-
Streptococcus_sp._HSISS3	+	-	-
Sciscionella_marina	+	-	-
Candidatus_Sungbacteria_bacterium_RIFCSPHIGHO2_01_FUI	+	-	-
Corynebacterium_deserti	+	-	-
Bifidobacterium_imperatoris	+	-	-
Rhodanobacter_sp.	+	-	-

Corynebacterium_sp._YIM_101343	+	-	-
Candidatus_Sulfobium_mesophilum	+	-	-
Thermoplasmatales_archaeon_SCGC_AB-540-F20	+	-	-
Thermocrinis_sp._GBS	+	-	-
Armatimonadetes_bacterium_RBG_19FT_COMBO_69_19	+	-	-
Rhodococcus_hoagii	+	-	-
Thermodesulfovibrio_aggregans	+	-	-
Lactobacillus_gigeriorum	+	-	-
Lactobacillus_ghanensis	+	-	-
Lactobacillus_fructivorans	+	-	-
Candidatus_Thioglobus_sp._MED-G25	+	-	-
Coraliomargarita_sp._TMED73	+	-	-
Arcobacter_sp._CECT_8986	+	-	-
Lactobacillus_saniviri	+	-	-
Kineococcus_xinjiangensis	+	-	-
Rhizobiales_bacterium_GAS113	+	-	-
Orpinomyces_joyonii	+	-	-
Candidatus_Roizmanbacteria_bacterium_RIFCSPHIGHO2_01_	+	-	-
Kluyveromyces_marxianus	+	-	-
Oligella_ureolytica	+	-	-
Apis_mellifera_associated_microvirus_59	+	-	-
Cryobacterium_sp._M25	+	-	-
Kribbella_sp._VKM_Ac-2569	+	-	-
Aphanocapsa_montana	+	-	-
Olsenella_sp._AM04-33	+	-	-
Anoxybacillus_pushchinoensis	+	-	-
Omnitrophica_WOR_2_bacterium_RIFCSPLOWO2_02_FULL_	+	-	-
Cutibacterium_avidum	+	-	-
Rhizobium_aegyptiacum	+	-	-
Comamonas_badia	+	-	-
Collinsella_sp._CAG:289	+	-	-
Thermococcus_cleftensis	+	-	-
Rhodomicrobium_vannielii	+	-	-
Lactobacillus_sp._HT06-2	+	-	-
Aspergillus_uvarum	+	-	-
Nosema_bombycis	+	-	-
Nostoc_sp._'Peltigera_malacea_cyanobiont'_DB3992	+	-	-
Thauera_hydrothermalis	+	-	-
Aureobasidium_pullulans	+	-	-
Collinsella_sp._AM33-4BH	+	-	-
Beggiatoa_sp._PS	+	-	-
Thermococcus_profundus	+	-	-
Arthrobacter_sp._Hiyo8	+	-	-
Aspergillus_calidoustus	+	-	-
Novosphingobium_sp._12-62-10	+	-	-
Cyanothece_sp._CCY0110	+	-	-
Streptococcus_urinalis	+	-	-
bacterium_RmlP026	+	-	-
Duddingtonia_flagrans	+	-	-
Xenorhabdus_innexi	+	-	-
Caulobacter_segnis	+	-	-
Methylobacterium_sp.	+	-	-
Plantactinospora_sp._CNZ320	+	-	-
Candidatus_Phycorickettsia_trachydisci	+	-	-
Syntrophobacter_fumaroxidans	+	-	-

Enterobacillus_tribolii	+	-	-
Leptospira_kirschneri	+	-	-
Litorimonas_taeanensis	+	-	-
Kordiimonas_lipolytica	+	-	-
Chloroflexi_bacterium_RBG_13_68_17	+	-	-
Sphingopyxis_sp.	+	-	-
Desulfobacula_sp._GWF2_41_7	+	-	-
Methylococcales_bacterium	+	-	-
Trichosporon_asahii	+	-	-
Gemmatimonadetes_bacterium_13_1_40CM_2_60_3	+	-	-
Deltaproteobacteria_bacterium_GWD2_55_8	+	-	-
Actinomyces_sp._410	+	-	-
Pseudolabrys_taiwanensis	+	-	-
Actinobacteria_bacterium_13_2_20CM_2_66_6	+	-	-
Staphylococcus_virus_JD7	+	-	-
Rhizobium_sp._LCM_4573	+	-	-
Methylomonas_sp._LWB	+	-	-
Halobacteriovorax_sp._JY17	+	-	-
Microbacterium_sp._SA39	+	-	-
Legionella_quinlivanii	+	-	-
Candidatus_Latescibacteria_bacterium_4484_7	+	-	-
Gluconobacter_oxydans	+	-	-
Bacillaceae_bacterium_MTCC_10057	+	-	-
Candidatus_Heimdallarchaeota_archaeon_B3-JM-08	+	-	-
Silvibacterium_bohemicum	+	-	-
Asticcacaulis_sp._AC402	+	-	-
Phyllobacterium_sp._YR531	+	-	-
Aureimonas_sp._M2BS4Y-1	+	-	-
Candidatus_Kaiserbacteria_bacterium_RIFCSPHIGHO2_01_FU	+	-	-
Sphingomonas_sp._Root241	+	-	-
Sphingomonas_sp._YZ-8	+	-	-
Collinsella_sp._AM16-21	+	-	-
Rickettsia_endosymbiont_of_Ixodes_scapularis	+	-	-
Cytophagaceae_bacterium_CAR-16	+	-	-
Verrucomicrobiaceae_bacterium_TMED86	+	-	-
Sphingobacteriales_bacterium_40-81	+	-	-
Anaerococcus_sp._SB3	+	-	-
Desulfuromonadaceae_bacterium_GWC2_58_13	+	-	-
Methylomicrobium_alcaliphilum	+	-	-
Candidatus_Thermofonsia_bacterium	+	-	-
Oceanospirillum_maris	+	-	-
Candidatus_Methanoperedenaceae_archaeon_HGW-Methanoper	+	-	-
Hydrotalea_flava	+	-	-
Luteibacter_rhizovicinus	+	-	-
Thermoplasmatales_archaeon_SCGC_AB-539-C06	+	-	-
Mesorhizobium_sanjuanii	+	-	-
Thermoplasmatales_archaeon_SG8-52-1	+	-	-
Actinobacillus_capsulatus	+	-	-
Megamonas_funiformis_CAG:377	+	-	-
Paraburkholderia_heleia	+	-	-
Arthrobacter_crystallopoietes	+	-	-
Herbaspirillum_sp._GW103	+	-	-
Armatimonadetes_bacterium_Cent15-Ar3	+	-	-
Pseudanabaena_sp.	+	-	-
Candidatus_Frackibacter_sp._WG11	+	-	-

Candidatus_Diapherotrites_archaeon_CG08_land_8_20_14_0_2	+	-	-
Desulfobacterales_bacterium_C00003106	+	-	-
bacterium_HR14	+	-	-
Idiomarina_sp._29L	+	-	-
Bacillus_sp._COPE52	+	-	-
Flavobacterium_sp._9	+	-	-
Vibrio_sp._E4404	+	-	-
Crenothrix_sp._D3	+	-	-
Caulobacterales_bacterium_32-67-6	+	-	-
Henriciella_algicola	+	-	-
Streptococcus_sp._AS14	+	-	-
Syntrophorhabdus_aromaticivorans	+	-	-
Roseimaritima_ulvae	+	-	-
Wolbachia_endosymbiont_of_Drosophila_yakuba	+	-	-
Thermotoga_sp._50_1627	+	-	-
Synechococcus_sp._GFB01	+	-	-
Arthrospira_sp._O9.13F	+	-	-
Synechococcus_sp._PCC_7335	+	-	-
Saprospiraceae_bacterium	+	-	-
Legionella_oakridgensis	-	-	-
candidate_division_TM6_bacterium_GW2011_GWF2_37_49	-	-	-
Roseisalinus_antarcticus	-	-	-
Candidatus_Magasanikbacteria_bacterium_RIFOXYC2_FULL_	-	-	-
Leminorella_richardii	-	-	-
Streptococcus_sp._HSISS2	-	-	-
Candidatus_Korarchaeota_archaeon	-	-	-
Deltaproteobacteria_bacterium_CG03_land_8_20_14_0_80_45_	-	-	-
Neochlamydia_sp._EPS4	-	-	-
Rhodospirillum_rubrum	-	-	-
Thorsellia_anophelis	-	-	-
Bacillus_haynesii	-	-	-
Parabacteroides_sp._2_1_7	-	-	-
Yersinia_aldovae	-	-	-
Paenibacillus_sp._Mc5Re-14	-	-	-
Kalmanozyma_brasiliensis	-	-	-
Dermabacter_sp._HSID17554	-	-	-
Desulfocarbo_indianensis	-	-	-
Parendozoicomonas_haliclonae	-	-	-
Propionicicella_superfundia	-	-	-
Shigella_flexneri	-	-	-
Colwellia_sp._RSH04	-	-	-
Chryseobacterium_defluvii	-	-	-
Candidatus_Woykebacteria_bacterium_RBG_13_40_15	-	-	-
Desulfobacteraceae_bacterium_4484_190.3	-	-	-
Methanolacinia_petrolearia	-	-	-
Enterobacter_sp._WCHEn090032	-	-	-
Botryobasidium_botryosum	-	-	-
Apibacter_adventoris	-	-	-
Candidatus_Micropelagos_thuwalensis	-	-	-
Ulvibacter_litoralis	-	-	-
Pseudohongiella_acticola	-	-	-
Methanobacterium_paludis	-	-	-
Limimaricola_hongkongensis	-	-	-
Persephonella_sp._KM09-Lau-8	-	-	-
Murid_betaherpesvirus_8	-	-	-

Mixta_theicola	-	-	-
Klebsiella_sp._4_1_44FAA	-	-	-
Methylovorus_sp._MM2	-	-	-
Flavipsychrobacter_stenotrophus	-	-	-
Burkholderiaceae_bacterium	-	-	-
Micromonospora_saelicesensis	-	-	-
Ichthyobacterium_seriolicida	-	-	-
Mucilaginibacter_sp._BJC16-A31	-	-	-
Bacillus_shackletonii	-	-	-
Chryseobacterium_taihuense	-	-	-
Methylobacterium_sp._WSM2598	-	-	-
Deltaproteobacteria_bacterium_CG_4_9_14_3_um_filter_63_12	-	-	-
Candidatus_Lambdaproteobacteria_bacterium_RIFOXYD2_FUI	-	-	-
Trichoderma_harzianum	-	-	-
Enterobacter_sp._638	-	-	-
Proteus_alimentorum	-	-	-
Eubacterium_sp._TF05-29	-	-	-
Candidatus_Falkowbacteria_bacterium_RIFOXYA2_FULL_47_	-	-	-
Defluviimonas_denitrificans	-	-	-
Verrucomicrobia_bacterium_Tous-C9LFEB	-	-	-
Collinsella_sp._TF06-26	-	-	-
Porphyrobacter_sanguineus	-	-	-
Aerococcus_sp._HMSC23C02	-	-	-
Roseomonas_sp._CPCC_101021	-	-	-
Bosea_sp._Root483D1	-	-	-
Candidatus_Nomurabacteria_bacterium_RIFOXYC2_FULL_36_	-	-	-
Candidatus_Yonathbacteria_bacterium_RIFOXYC2_FULL_47_	-	-	-
Rhodanobacter_spathiphylli	-	-	-
Fischerella_sp._PCC_9605	-	-	-
Photobacterium_lipolyticum	-	-	-
Phlebiopsis_gigantea	-	-	-
Planctomycetales_bacterium_4572_13	-	-	-
Erysipelotrichaceae_bacterium_NYU-BL-E8	-	-	-
Fibrobacteres_bacterium_CG2_30_45_31	-	-	-
Pseudoruegeria_aquimaris	-	-	-
Bordetella_bronchiseptica	-	-	-
Aureimonas_sp._Leaf324	-	-	-
Candidatus_Curtissbacteria_bacterium_RBG_16_39_7	-	-	-
Corynebacterium_xerosis	-	-	-
Pneumocystis_jirovecii	-	-	-
Parachlamydia_acanthamoebae	-	-	-
Mycoplasma_pulmonis	-	-	-
Martelella_mediterranea	-	-	-
Sneathiella_glossodoripedis	-	-	-
Stenotrophomonas_phage_vB_SmaS-DLP_6	-	-	-
Minicystis_rosea	-	-	-
Bacillus_stratosphericus	-	-	-
Cladophialophora_psammophila	-	-	-
Spiroplasma_sp._ChiS	-	-	-
Methanopyrus_sp._KOL6	-	-	-
Streptomyces_viridosporus	-	-	-
Rhizopogon_vinicolor	-	-	-
Puccinia_sorghi	-	-	-
Lactococcus_phage_AM4	-	-	-
Noviherbaspirillum_autotrophicum	-	-	-

Candidatus_Wolfebacteria_bacterium	-	-	-
Leptonema_illini	-	-	-
Eubacterium_sp._AF16-48	-	-	-
Giesbergeria_anulus	-	-	-
Verrucomicrobiae_bacterium_Tous-C3TDCM	-	-	-
Veillonella_sp._DORA_B_18_19_23	-	-	-
Yamadazyma_tenuis	-	-	-
Edaphobacter_modestus	-	-	-
Fowlpox_virus	-	-	-
Exiguobacterium_sp._RIT341	-	-	-
Insolitispirillum_peregrinum	-	-	-
Geminisphaera_colitermitum	-	-	-
Flavobacterium_pallidum	-	-	-
Rickettsiales_endosymbiont_of_Stachyamoeba_lipophora	-	-	-
Candidatus_Phaeomarinobacter_ectocarpi	-	-	-
Rhizobium_sp._NFR07	-	-	-
Streptomyces_sp._AC1-42W	-	-	-
Marinobacter_psychrophilus	-	-	-
Belnapia_moabensis	-	-	-
Methylocaldum_szegediense	-	-	-
Sulfurovum_sp._AR	-	-	-
Litoreibacter_albidus	-	-	-
Methanospirillum_stamsii	-	-	-
Wickerhamiella_sorbophila	-	-	-
Candidatus_Bathyarchaeota_archaeon_B24-2	-	-	-
Corallincola_sp._C4	-	-	-
Candidatus_Bipolaricaulis_sp._Ch78	-	-	-
Sphingomonas_sp._JJ-A5	-	-	-
Albimonas_donghaensis	-	-	-
Phreatobacter_cathodiphilus	-	-	-
uncultured_marine_group_II/III_euryarchaeote_SAT1000_27_D	-	-	-
Nannizzia_gypsea	-	-	-
Chloroflexi_bacterium_RBG_13_46_9	-	-	-
Variovorax_sp._369	-	-	-
Prosthecochloris_sp._ZM	-	-	-
Nitrosopumilus_sp._BACL13_MAG-121220-bin23	-	-	-
Acidimicrobium_sp._BACL19_MAG-120924-bin39	-	-	-
Microscilla_sp._PRE1	-	-	-
bacterium_HR39	-	-	-
Nitrospirae_bacterium_CG_4_10_14_3_um_filter_44_29	-	-	-
alpha_proteobacterium_Q-1	-	-	-
Sphingomonas_oleivorans	-	-	-
Stappia_sp._ES.058	-	-	-
Silvanigrellales_bacterium_RF1110005	-	-	-
Solirubrobacter_pauli	-	-	-
Mycoplasma_felifaucium	-	-	-
Microcystis_sp._MC19	-	-	-
Myxococcaceae_bacterium	-	-	-
Bacillus_sp._RJGP41	-	-	-
Alphaproteobacteria_bacterium_MarineAlpha3_Bin4	-	-	-
Alphaproteobacteria_bacterium_MarineAlpha3_Bin5	-	-	-
Henriciella_aquimarina	-	-	-
Spirosoma_panaciterrae	-	-	-
Rhodospirillaceae_bacterium_SYSU_D60015	-	-	-
Bartonella_washoeensis	-	-	-

Halogranum_salarium	-	-	-
Halomonas_beimenensis	-	-	-
Gammaproteobacteria_bacterium_RIFCSPHIGHO2_12_FULL_	-	-	-
Notoacmeibacter_marinus	-	-	-
Aliidiomarina_maris	-	-	-
Schizopora_paradoxa	-	-	-
Thalassospira_lohafexi	-	-	-
Candidatus_Geothermarchaeota_archaeon_ex4572_27	-	-	-
Collinsella_sp._AM44-11	-	-	-
Aureimonas_frigidaquae	-	-	-
Candidatus_Erwinia_haradaeae	-	-	-
Acinetobacter_calcoaceticus	-	-	-
Lentisphaerae_bacterium_RIFOXYA12_FULL_48_11	-	-	-
Tenericutes_bacterium	-	-	-
Janibacter_sp._HTCC2649	-	-	-
Legionella_hackeliae	-	-	-
Laceyella_sediminis	-	-	-
uncultured_bacterium_Contig643	-	-	-
Kordia_sp._SMS9	-	-	-
Labrenzia_sp._011	-	-	-
Planktomarina_temperata	-	-	-
Cryptococcus_depauperatus	-	-	-
Erythrobacter_sp._SG61-1L	-	-	-
Thermococcus_sp._EXT12c	-	-	-
Candidatus_Bathyarchaeota_archaeon_BA2	-	-	-
Pelagibacteraceae_bacterium_TMED259	-	-	-
Tropicimonas_isoalkanivorans	-	-	-
Entomoplasmatales_bacterium_EntAcro10	-	-	-
Candidatus_Portnoybacteria_bacterium_RIFCSPHIGHO2_12_F	-	-	-
Amphiamblys_sp._WSBS2006	-	-	-
Streptomyces_sp._NRRL_B-1381	-	-	-
Magnetospirillum_marisnigri	-	-	-
Hyphomicrobium_nitrativorans	-	-	-
Marine_Group_III_euryarchaeote_CG-Epi1	-	-	-
Candidatus_Moranbacteria_bacterium_CG2_30_41_165	-	-	-
Actinobacteria_bacterium_21-73-9	-	-	-
Candidatus_Berkelbacteria_bacterium_CG23_combo_of_CG06-	-	-	-
Shigella_sonnei	-	-	-
Oryctes_rhinoceros_nudivirus	-	-	-
Exiguobacterium_mexicanum	-	-	-
Fusarium_proliferatum	-	-	-
Rhizobium_sp._Leaf155	-	-	-
Candidatus_Protochlamydia_sp._R18	-	-	-
Ophiostoma_piceae	-	-	-
Campylobacter_sp._AAUH-44UCsig-a	-	-	-
Dichotomicrobium_thermohalophilum	-	-	-
Elusimicrobia_bacterium_CG08_land_8_20_14_0_20_59_10	-	-	-
Candidatus_Ryanbacteria_bacterium_RIFCSPHIGHO2_01_FUI	-	-	-
Namao_virus	-	-	-
Schizosaccharomyces_japonicus	-	-	-
Serendipita_vermifera	-	-	-
Elephantid_betaherpesvirus_1	-	-	-
Chloroflexi_bacterium_RBG_13_56_8	-	-	-
Actinobaculum_sp._313	-	-	-
Phyllobacteriaceae_bacterium_SYSU_D60012	-	-	-

Gluconobacter_kondonii	-	-	-
Salinivenus_lutea	-	-	-
Phaeobacter_piscinae	-	-	-
Gammaproteobacteria_bacterium_45_16_T64	-	-	-
Pseudomonas_sp._GM33	-	-	-
Novosphingobium_guangzhouense	-	-	-
Flavobacterium_sp._316	-	-	-
Paraburkholderia_ferrariae	-	-	-
Bradyrhizobium_pachyrhizi	-	-	-
Candidatus_Muproteobacteria_bacterium_RBG_16_60_9	-	-	-
Bartonella_melophagi	-	-	-
Acidobacteriales_bacterium_13_1_40CM_3_55_5	-	-	-
Candidatus_Peregrinibacteria_bacterium_CG2_30_44_17	-	-	-
RD114_retrovirus	-	-	-
Butyricicoccus_sp._TM10-16AC	-	-	-
Rahnella_sp._DSM_105170	-	-	-
Flavobacteriaceae_bacterium_CG17_big_fil_post_rev_8_21_14_	-	-	-
Acidobacteria_bacterium_13_1_40CM_3_55_6	-	-	-
Novosphingobium_tardaugens	-	-	-
Advenella_kashmirensis	-	-	-
Elusimicrobia_bacterium_RIFOXYD12_FULL_66_9	-	-	-
Lebetimonas_sp._JH292	-	-	-
Streptococcus_downei	-	-	-
Bacillus_sp._JCM_19041	-	-	-
Streptococcus_macacae	-	-	-
Leptospirillum_ferriphilum	-	-	-
Microgenomates_group_bacterium_GW2011_GWB1_44_8	-	-	-
Compostimonas_suwonensis	-	-	-
Aspergillus_saccharolyticus	-	-	-
Staphylococcus_argenteus	-	-	-
Azobacteroides_phage_ProJPt-Bp1	-	-	-
Hanseniaspora_osmophila	-	-	-
Lactobacillus_yonginensis	-	-	-
Syntrophorhabdus_sp._PtaU1.Bin058	-	-	-
Mesorhizobium_sp._M4A.F.Ca.ET.020.02.1.1	-	-	-
Ideonella_sp._B508-1	-	-	-
Streptosporangiaceae_bacterium_YIM_75507	-	-	-
Hypoxylon_sp._CI-4A	-	-	-
Melampsora_larici-populina	-	-	-
Sulfurifustis_variabilis	-	-	-
Methanosaeta_sp._PtaU1.Bin112	-	-	-
Bacillus_sp._AFS054943	-	-	-
Alphaproteobacteria_bacterium_HGW-Alphaproteobacteria-11	-	-	-
Sulfurospirillum_sp._MES	-	-	-
Vibrio_sp._C7	-	-	-
Komagataeibacter_sucrofermentans	-	-	-
Sphingopyxis_sp._Root1497	-	-	-
Sphingomonas_elodea	-	-	-
Croceicoccus_sp._GM-16	-	-	-
Haloferax_sp._ATB1	-	-	-
Xenorhabdus_nematophila	-	-	-
Thermus_thermophilus	-	-	-
Flavobacteriaceae_bacterium_CRH	-	-	-
Jhaorihella_thermophila	-	-	-
Pseudomonas_sp._p106	-	-	-

Caldimonas_manganoxidans	-	-	-
Flavobacterium_sp._CJ74	-	-	-
Bosea_sp._Root381	-	-	-
Sulfurovum_sp._FS08-3	-	-	-
Sulfurimonas_gotlandica	-	-	-
Prochlorococcus_phage_P-SSM5	-	-	-
Hyphomicrobium_sp.	-	-	-
Valsa_malicola	-	-	-
Fomitiporia_mediterranea	-	-	-
Candidatus_Rokubacteria_bacterium_13_1_20CM_4_70_14	-	-	-
Rhizobiales_bacterium_TMED29	-	-	-
Pigmentiphaga_kullae	-	-	-
candidate_division_WWE3_bacterium_CG08_land_8_20_14_0_	-	-	-
Acinetobacter_sp._NRRL_B-65365	-	-	-
Cribrihabitans_marinus	-	-	-
Lactobacillus_oeni	-	-	-
Elusimicrobia_bacterium_RIFCSPLOWO2_02_FULL_61_11	-	-	-
Sulfitobacter_sp._AM1-D1	-	-	-
Kordiimonadales_bacterium	-	-	-
Oxalobacter_sp.	-	-	-
Arenicella_xantha	-	-	-
Flavihumibacter_petaseus	-	-	-
Pseudomonas_oryzihabitans	-	-	-
Cohaesibacter_marisflavi	-	-	-
Pseudomonas_sp._AD21	-	-	-
Candidatus_Cardinium_hertigii	-	-	-
Paracoccus_contaminans	-	-	-
Terracidiphilus_gabretensis	-	-	-
Rhizobiales_bacterium_YIM_77505	-	-	-
Collinsella_sp._AM40-7AC	-	-	-
Thalassospira_sp._MCCC_1A02491	-	-	-
Azoarcus_sp._SY39	-	-	-
Pseudomicrostroma_glucosiphilum	-	-	-
Candidatus_Gracilibacteria_bacterium_CG17_big_fil_post_rev_	-	-	-
Candidatus_Gracilibacteria_bacterium_GN02-872	-	-	-
Candidatus_Heimdallarchaeota_archaeon	-	-	-
Trichoderma_reesei	-	-	-
Lactobacillus_sp._ESL0260	-	-	-
Postia_placenta	-	-	-
Candidatus_Marinoinvertebrata_rohwerii	-	-	-
Inmirania_thermothiophila	-	-	-
Podospora_anserina	-	-	-
Vagococcus_teuberi	-	-	-
Flavobacterium_sp._AED	-	-	-
Bacillus_abyssalis	-	-	-
Candidatus_Filomicrobium_marinum	-	-	-
Bradyrhizobium_sp._Ai1a-2	-	-	-
Peniophora_sp._CONT	-	-	-
Ruegeria_arenilitoris	-	-	-
Micromonospora_sp._NRRL_B-16802	-	-	-
Micromonospora_rhizosphaerae	-	-	-
Nitrospirae_bacterium_CG_4_9_14_0_8_um_filter_70_14	-	-	-
Xanthomonadales_bacterium_CG02_land_8_20_14_3_00_62_1	-	-	-
Afipia_broomeae	-	-	-
Desulfovibrio_brasiliensis	-	-	-

Nitratireductor_aquibiodomus	-	-	-
Xanthomonas_citri	-	-	-
Chaetomium_thermophilum	-	-	-
Halomonas_sp._QHL1	-	-	-
Methylocella_silvestris	-	-	-
Candidatus_Zambryskibacteria_bacterium_RIFCSPHIGHO2_02	-	-	-
Methylobacterium_sp._MIMD6	-	-	-
Methylobacterium_sp._Leaf87	-	-	-
Microbotryum_silenes-dioicae	-	-	-
Catelliglobosispora_koreensis	-	-	-
Microbacterium_sp._oral_taxon_186	-	-	-
Microbacterium_sorbitolivorans	-	-	-
Dokdonia_pacifica	-	-	-
SAR202_cluster_bacterium_Casp-Chloro-G4	-	-	-
Chloroflexi_bacterium_RBG_16_72_14	-	-	-
Gordonia_sp._RS15-1S	-	-	-
Mycolicibacterium_diernhoferi	-	-	-
Mycoplasma_mobile	-	-	-
Sphingobacteriales_bacterium_BACL12_MAG-120802-bin5	-	-	-
Mycoplasma_bovigenitalium	-	-	-
Chryseobacterium_palustre	-	-	-
Choristoneura_rosaceana_nucleopolyhedrovirus	-	-	-
Agromyces_ramosus	-	-	-
Neorhizobium_sp._NCHU2750	-	-	-
Halobacteriovorax_sp._DA5	-	-	-
Citromicrobium_sp._JLT1363	-	-	-
Mucilaginibacter_sp._44-25	-	-	-
Spirosoma_rigui	-	-	-
Chitinophagaceae_bacterium_BSSC1	-	-	-
Mycobacterium_sp._1100029.7	-	-	-
Gordonia_polyisoprenivorans	-	-	-
Sphingopyxis_sp._LPB0140	-	-	-
Methanomethylovorans_sp._PtaU1.Bin073	-	-	-
candidate_division_TM6_bacterium_RIFCSPHIGHO2_12_FUL	-	-	-
Alphaproteobacteria_bacterium_MarineAlpha9_Bin2	-	-	-
Metallosphaera_sedula	-	-	-
Rhodobacter_sphaeroides	-	-	-
Alphaproteobacteria_bacterium_MarineAlpha3_Bin1	-	-	-
Mesorhizobium_sp._WSM4312	-	-	-
Alphaproteobacteria_bacterium_MarineAlpha10_Bin3	-	-	-
Streptococcus_sp._JS71	-	-	-
Gallaecimonas_xiamenensis	-	-	-
Methanosarcina_spelaei	-	-	-
Methanosarcina_sp._Ant1	-	-	-
Candidatus_Wolfebacteria_bacterium_RIFOXYD12_FULL_48_	-	-	-
Clostridium_phage_phiZP2	-	-	-
Novimethylophilus_kurashikiensis	-	-	-
Sulfitobacter_sp._JL08	-	-	-
Bacillus_virus_GA1	-	-	-
Punctularia_strigosozonata	-	-	-
Xylona_heveae	-	-	-
Blastocatellia_bacterium_AA13	-	-	-
uncultured_bacterium_A1Q1_fos_18	-	-	-
Polynucleobacter_sp._39-46-10	-	-	-
uncultured_marine_microorganism_HF4000_APKG2J17	-	-	-

Rhodobacter_azotoformans	-	-	-
Tilletiopsis_washingtonensis	-	-	-
Prochlorococcus_sp._HOT208_60m_805A16	-	-	-
Planctomycetales_bacterium_4484_113	-	-	-
Sodiomyces_alkalinus	-	-	-
Prevotella_sp._Sc00044	-	-	-
Solemya_pervernicosa_gill_symbiont	-	-	-
Bacillus_sp._AFS015802	-	-	-
Siphonobacter_sp._BAB-5404	-	-	-
Blastocladiella_emersonii	-	-	-
Tilletia_caries	-	-	-
Streptomyces_sp._RV15	-	-	-
Rhizobacter_gummiphilus	-	-	-
Rheinheimera_sp._SA_1	-	-	-
Alphaproteobacteria_bacterium_MarineAlpha9_Bin1	-	-	-
Calothrix_sp._NIES-4071	-	-	-
Thiomicrospira_aerophila	-	-	-
Seonamhaeicola_aphaedonensis	-	-	-
Acanthamoeba_polyphaga_mimivirus	-	-	-
Serratia_ficaria	-	-	-
uncultured_bacterium_EB5	-	-	-
Streptomyces_sp._NRRL_F-5135	-	-	-
Polaribacter_sp._BM10	-	-	-
Arcobacter_sp._CECT_9299	-	-	-
Alphaproteobacteria_bacterium_MarineAlpha3_Bin7	-	-	-
Aquimarina_aggregata	-	-	-
Polaribacter_sejongensis	-	-	-
Sphingomonas_sp._CF311	-	-	-
Pleurocapsa_sp._PCC_7319	-	-	-
Antricoccus_suffuscus	-	-	-
Promicromonospora_sp._PT9	-	-	-
Sphingomonas_jaspsi	-	-	-
Providencia_heimbachae	-	-	-
Azotobacter_chroococcum	-	-	-
Streptacidiphilus_neutrinimicus	-	-	-
Superficieibacter_electus	-	-	-
Verrucomicrobiae_bacterium_DG1235	-	-	-
Acidobacteriales_bacterium	-	-	-
Thalassobius_activus	-	-	-
Burkholderia_anthina	-	-	-
Azospirillum_oryzae	-	-	-
Vibrio_phage_1.081.O._10N.286.52.C2	-	-	-
Pseudomonas_delhiensis	-	-	-
Acidovorax_sp._Root267	-	-	-
Roseobacter_sp._AzwK-3b	-	-	-
Bradyrhizobium_sp._TSA1	-	-	-
Vulcanococcus_limneticus	-	-	-
Rhodospirillaceae_bacterium_NAU-10	-	-	-
Talaromyces_amestolkiae	-	-	-
Pseudomonas_sp._P818	-	-	-
Brenneria_salicis	-	-	-
Weeksella_sp._HMSC059D05	-	-	-
Bartonella_australis	-	-	-
Vibrio_azureus	-	-	-
Rosellinia_necatrix	-	-	-

Aequorivita_sublithincola	-	-	-
gamma_proteobacterium_IMCC2047	-	-	-
Pseudoalteromonas_sp._JW3	-	-	-
Pseudorhodoplanes_sinuspersici	-	-	-
candidate_division_WWE3_bacterium_GW2011_GWC2_41_23	-	-	-
Aspergillus_sclerotiicarbonarius	-	-	-
Aspergillus_taichungensis	-	-	-
fungal_sp._No.11243	-	-	-
Proteobacteria_bacterium_ST_bin15	-	-	-
Acinetobacter_qingfengensis	-	-	-
Amorphotheca_resinae	-	-	-
Proteus_sp._3M	-	-	-
Asaia_bogorensis	-	-	-
Pseudoalteromonas_sp._DSM_26666	-	-	-
Bogoriella_caseilytica	-	-	-
Rufibacter_sp._R-22-1c-1	-	-	-
Burkholderiales_bacterium_28-67-8	-	-	-
Vibrio_phage_1.123.O._10N.286.48.F3	-	-	-
delta_proteobacterium_PSCGC_5342	-	-	-
Burkholderia_sp._YR277	-	-	-
Verrucomicrobia_bacterium_SCGC_AAA164-O14	-	-	-
Ruania_albidiflava	-	-	-
Sporosarcina_ureae	-	-	-
Aureimonas_sp._AU20	-	-	-
Thauera_sp._D20	-	-	-
Rhodococcus_corynebacterioides	-	-	-
Aurantimonas_coralicida	-	-	-
Nostoc_punctiforme	-	-	-
Laccaria_bicolor	-	-	-
Cowpox_virus	-	-	-
Oleiagrimonas_soli	-	-	-
Kwoniella_dejecticola	-	-	-
Cryobacterium_arcticum	-	-	-
Cylindrospermopsis_sp._CR12	-	-	-
Debaryomyces_hansenii	-	-	-
Candidatus_Rokubacteria_bacterium_CSP1-6	-	-	-
Cordyceps_sp._RAO-2017	-	-	-
Cordyceps_militaris	-	-	-
Lacinutrix_algicola	-	-	-
Candidatus_Woesebacteria_bacterium_RIFCSPHIGHO2_01_FU	-	-	-
Lactobacillus_gorillae	-	-	-
Candidatus_Thorarchaeota_archaeon_SMTZ1-45	-	-	-
Dehalogenimonas_lykanthroporepellens	-	-	-
Human_mammary_tumor_virus	-	-	-
Helicobacter_sp._MIT_14-3879	-	-	-
Hanseniaspora_opuntiae	-	-	-
Candidatus_Magasanikbacteria_bacterium_RIFOXYA1_FULL_	-	-	-
Halomonas_sp._LBP4	-	-	-
Candidatus_Lloydbacteria_bacterium_CG22_combo_CG10-13_	-	-	-
Haloferax_sp._Atlit-4N	-	-	-
Haloferax_mediterranei	-	-	-
Halobacillus_sp._BAB-2008	-	-	-
Deltaproteobacteria_bacterium_RIFOXYD12_FULL_57_12	-	-	-
Deltaproteobacteria_bacterium_CG07_land_8_20_14_0_80_38_	-	-	-
Deltaproteobacteria_bacterium_HGW-Deltaproteobacteria-2	-	-	-

Deltaproteobacteria_bacterium_RIFCSPLOWO2_12_FULL_60_	-	-	-
Hymenobacter_sp._1-3-3-3	-	-	-
Desulfobacterales_bacterium_S3730MH5	-	-	-
Paraferrimonas_sedimenticola	-	-	-
Lactobacillus_timonensis	-	-	-
Mycoplasma_opalescens	-	-	-
Methylophilales_bacterium_BACL14_MAG-120910-bin43	-	-	-
Methylomonas_sp._MK1	-	-	-
Methylomonas_sp._DH-1	-	-	-
Methyloceanibacter_methanicus	-	-	-
Methylobacterium_platani	-	-	-
Natrinema_ejinorense	-	-	-
Natronoarchaeum_philippinense	-	-	-
Chlorociboria_aeruginascens	-	-	-
Myceligenerans_xiligouense	-	-	-
Chryseobacterium_sp._F5649	-	-	-
Citromicrobium_sp.	-	-	-
Mycoplasma_neurolyticum	-	-	-
Moelleriella_libera	-	-	-
Microtetraspora_niveoalba	-	-	-
Microbotryum_lychnidis-dioicae	-	-	-
Microbispora_rosea	-	-	-
Mesorhizobium_sp._M3A.F.Ca.ET.080.04.2.1	-	-	-
Nitrospirae_bacterium_RBG_13_39_12	-	-	-
Nitrospira_bacterium_SG8_3	-	-	-
Nitrospirae_bacterium_CG2_30_41_42	-	-	-
Caulobacterales_bacterium_RIFCSPHIGHO2_01_FULL_70_19	-	-	-
Macacine_betaherpesvirus_3	-	-	-
Marmoricola_sp._Leaf446	-	-	-
Marivita_sp._XM-24bin2	-	-	-
Marinomonas_sp._BSi20584	-	-	-
Neurospora_tetrasperma	-	-	-
Marinomonas_aquiplantarum	-	-	-
Chaetothyriales_sp._CBS_134916	-	-	-
Chaetomium_globosum	-	-	-
Cercospora_beticola	-	-	-
Cercopithecine_betaherpesvirus_5	-	-	-
Magnetospirillum_sp._LBB-42	-	-	-
Magnetospirillum_moscoviense	-	-	-
Magnetospirillum_magneticum	-	-	-
Formivibrio_citricus	-	-	-
Pedobacter_sp._KBW06	-	-	-
Candidatus_Falkowbacteria_bacterium_CG10_big_fil_rev_8_21	-	-	-
Dyella_sp._333MFSha	-	-	-
Geobacillus_virus_E2	-	-	-
Geobacillus_sp._T6	-	-	-
Parvularcula_bermudensis	-	-	-
Gammaproteobacteria_bacterium_HGW-Gammaproteobacteria-	-	-	-
Eremothecium_cymbalariae	-	-	-
Eremothecium_sinecaudum	-	-	-
Campylobacter_sp._P0124	-	-	-
Pisolithus_microcarpus	-	-	-
Erythrobacter_sp._HL-111	-	-	-
Pelagibacteraceae_bacterium_TMED267	-	-	-
Pelagibacterium_sp._SCN_68-10	-	-	-

Pelagicola_litorisediminis	-	-	-
Candidatus_Campbellbacteria_bacterium_CG22_combo_CG10-	-	-	-
Candidatus_Buchananbacteria_bacterium_RIFCSPHIGHO2_01_	-	-	-
Candidatus_Berkiella_aquae	-	-	-
Chryseobacterium_sp._YR460	-	-	-
Haliangium_ochraceum	-	-	-
Marssonina_coronariae	-	-	-
Marssonina_brunnea	-	-	-
Streptomyces_sp._NRRL_S-87	-	-	-
Streptomyces_sp._SMS_SU21	-	-	-
Enterococcus_phage_EFLK1	-	-	-
Meiothermus_roseus	-	-	-
Acidobacteria_bacterium_21-70-11	-	-	-
Bacillus_sp._AFS029637	-	-	-
Flavobacteriaceae_bacterium_CG02_land_8_20_14_3_00_34_1	-	-	-
Streptomyces_globisporus	-	-	-
Metarhizium_rileyi	-	-	-
Gymnopilus_dilepis	-	-	-
Bacillus_soli	-	-	-
Marinobacter_sp._F01	-	-	-
Syntrophobacterales_bacterium_CG_4_9_14_3_um_filter_49_8	-	-	-
Leptospira_brenneri	-	-	-
Acinetobacter_sp._ANC_4999	-	-	-
Wolbachia_endosymbiont_of_Dactylopius_coccus	-	-	-
Marine_Group_III_euryarchaeote_CG-Epi3	-	-	-
Acidovorax_cattleyae	-	-	-
Acidovorax_sp._SCN_68-22	-	-	-
Grimontia_celer	-	-	-
Flavobacterium_sp._ABG	-	-	-
Acinetobacter_haemolyticus	-	-	-
Acinetobacter_indicus	-	-	-
Lysinibacillus_sp._F5	-	-	-
Lutibaculum_baratangense	-	-	-
Exophiala_oligosperma	-	-	-
Sporosarcina_sp._P16b	-	-	-
Microvirga_sp._17_mud_1-3	-	-	-
Stagonospora_sp._SRC1lsM3a	-	-	-
Methanococcus_aeolicus	-	-	-
Aliivibrio_wodanis	-	-	-
Staphylococcus_pettenkoferi	-	-	-
Staphylococcus_simiae	-	-	-
Acaromyces_ingoldii	-	-	-
Mucilaginibacter_yixingensis	-	-	-
Exiguobacterium_aurantiacum	-	-	-
Mucilaginibacter_sp._PAMC_26640	-	-	-
Methylocystis_parvus	-	-	-
Haloferacaceae_archaeon_SYSU_A9-0	-	-	-
Xinfangfangia_sp._CIP_111625	-	-	-
Methylibium_sp._CF468	-	-	-
Streptococcus_sp._HMSC061D01	-	-	-
Clostridium_phage_susfortuna	-	-	-
uncultured_Ignavibacteria_bacterium_Rifle_16ft_4_minimus_38	-	-	-
Tepidicaulis_sp._EA10	-	-	-
Methylohalobius_crimeensis	-	-	-
Halobacteriales_archaeon_QH_10_70_21	-	-	-

Steroidobacter_sp._JW-3	-	-	-
Microbacterium_sp._Leaf151	-	-	-
Fibularhizoctonia_sp._CBS_109695	-	-	-
Methylopila_sp._M107	-	-	-
Methylophaga_nitratireducenticrescens	-	-	-
Halobacteriovorax_marinus	-	-	-
Treponema_pallidum	-	-	-
Trichoderma_gamsii	-	-	-
Ustilago_maydis	-	-	-
Kaistia_granuli	-	-	-
Alphaproteobacteria_bacterium_RIFCSPHIGHO2_12_FULL_6(	-	-	-
Alphaproteobacteria_bacterium_RIFOXYD12_FULL_60_8	-	-	-
Hymenobacter_norwichensis	-	-	-
Drechmeria_coniospora	-	-	-
Hymenobacter_perfusus	-	-	-
Cupriavidus_pinatubonensis	-	-	-
Kosakonia_sp._S29	-	-	-
Sphingopyxis_bauzanensis	-	-	-
Afipia_clevelandensis	-	-	-
Imhoffiella_purpurea	-	-	-
Diplodia_seriata	-	-	-
Ignavibacteria_bacterium_GWC2_38_9	-	-	-
Ignavibacteria_bacterium_GWA2_54_16	-	-	-
Vibrio_sp._EJY3	-	-	-
Dickeya_chrysanthemi	-	-	-
Hyphomonas_sp._Mor2	-	-	-
Hyphomonas_sp._CACIAM_19H1	-	-	-
Janthinobacterium_sp._TND4EL3	-	-	-
Verrucomicrobia_bacterium_GWF2_51_19	-	-	-
bacterium_BRH_c32	-	-	-
Jannaschia_sp._12N15	-	-	-
Deltaproteobacteria_bacterium_RBG_19FT_COMBO_52_11	-	-	-
Coxiella_sp._RIFCSPHIGHO2_12_FULL_44_14	-	-	-
Collinsella_sp._AM43-1	-	-	-
Aureimonas_altamirensis	-	-	-
Halioglobus_sp._U0301	-	-	-
Thermanaerovibrio_acidaminovorans	-	-	-
Atopobium_deltae	-	-	-
Aspergillus_thermomutatus	-	-	-
Actinobacteria_bacterium_RBG_16_70_17	-	-	-
Termitomyces_sp._J132	-	-	-
Leeuwenhoekiella_sp._MAR_2009_132	-	-	-
Azospirillum_sp._TSH100	-	-	-
Alkalispirochaeta_odontotermitis	-	-	-
Collinsella_sp._AM24-1	-	-	-
Lactobacillus_virus_LLKu	-	-	-
Arcticibacter_svalbardensis	-	-	-
Lactobacillus_farraginis	-	-	-
Arcobacter_lekithochrous	-	-	-
Gallionellales_bacterium_GWA2_59_43	-	-	-
candidate_division_BRC1_bacterium_HGW-BRC1-1	-	-	-
Thermotoga_sp.	-	-	-
Laccaria_amethystina	-	-	-
Consotaella_salsifontis	-	-	-
Arthrobotrys_oligospora	-	-	-

Coprobacillus_sp._AF16-47	-	-	-
Desulfuromonas_sp._TF	-	-	-
Lactobacillus_mellis	-	-	-
Armatimonadetes_bacterium	-	-	-
Niveispirillum_irakense	-	-	-
Pararhodobacter_sp._AM505	-	-	-
Rodentibacter_pneumotropicus	-	-	-
Cedecea_neteri	-	-	-
Rhodovulum_sp._PH10	-	-	-
Pseudogulbenkiania_subflava	-	-	-
Nonomuraea_sp._NEAU-YG30	-	-	-
Candidatus_Yanofskybacteria_bacterium_RIFCSPHIGHO2_01_	-	-	-
Candidatus_Yanofskybacteria_bacterium_RIFCSPHIGHO2_01_	-	-	-
Brevibacillus_sp._VP	-	-	-
Nostoc_sp._RF31Y	-	-	-
Nosema_apis	-	-	-
Nocardioides_allogilvus	-	-	-
Runella_sp.	-	-	-
Pedobacter_soli	-	-	-
Chitinophaga_ginsengisegetis	-	-	-
Candidatus_Daviesbacteria_bacterium_RIFCSPHIGHO2_01_FU	-	-	-
Propionibacteriaceae_bacterium_16Sb5-5	-	-	-
Parcubacteria_group_bacterium_GW2011_GWC1_39_12	-	-	-
Pseudoalteromonas_translucida	-	-	-
Cellulophaga_sp._W5C	-	-	-
Nitrosomonas_sp._Nm34	-	-	-
Rubrobacter_indicoceani	-	-	-
Nitrosomonas_nitrosa	-	-	-
Paucibacter_sp._KBW04	-	-	-
Bacteroidetes_bacterium_4572_114	-	-	-
Pseudomonas_psychrophila	-	-	-
Pseudorhodoferax_soli	-	-	-
Candidatus_Saccharicenans_subterraneum	-	-	-
Bordetella_genomosp._12	-	-	-
Bordetella_genomosp._4	-	-	-
Pseudozyma_hubeiensis	-	-	-
Blastopirellula_marina	-	-	-
Rachicladosporium_sp._CCFEE_5018	-	-	-
Ostreococcus_lucimarinus_virus_1	-	-	-
Pyrrhoderma_noxium	-	-	-
Candidatus_Rokubacteria_bacterium_RIFCSPLOWO2_02_FUL	-	-	-
Psychrobacter_alimentarius	-	-	-
Psychroflexus_gondwanensis	-	-	-
Pseudomonas_sp._45MFCol3.1	-	-	-
Bradyrhizobium_sp._CCBAU_53390	-	-	-
Candidatus_Uhrbacteria_bacterium_RIFCSPLOWO2_02_FULL	-	-	-
Candidatus_Viridilinea_mediisalina	-	-	-
Rhodocyclaceae_bacterium_Paddy-1	-	-	-
Rhodoferax_ferrireducens	-	-	-
Betaproteobacteria_bacterium_RIFCSPLOWO2_02_FULL_64_	-	-	-
Candidatus_Methylomirabilis_limnetica	-	-	-
Candidatus_Thermofonsia_Clade_1_bacterium	-	-	-
Rhodobacteraceae_bacterium_HTCC2083	-	-	-
Rhodobacteraceae_bacterium_WDS1C4	-	-	-
Paludibacter_sp._SCN_50-10	-	-	-

Candidatus_Melainabacteria_bacterium_RIFCSPLOWO2_12_F	-	-	-
Phenylobacterium_sp._LX32	-	-	-
Sphingobium_sp._YL23	-	-	-
Sphingomonadales_bacterium	-	-	-
Mycoplasma_edwardii	-	-	-
Shewanella_algidipiscicola	-	-	-
Sinomonas_sp._R1AF57	-	-	-
Mycoplasma_anseris	-	-	-
Solibacillus_silvestris	-	-	-
Sediminibacillus_halophilus	-	-	-
Shewanella_sp._10N.286.51.B7	-	-	-
Photorhabdus_thracensis	-	-	-
Mycoplasma_conjunctivae	-	-	-
Scleroderma_citrinum	-	-	-
Candidatus_Cloacimonas_acidaminovorans	-	-	-
Sphingomonas_astaxanthinifaciens	-	-	-
Pichia_membranifaciens	-	-	-
Myroides_sp._A21	-	-	-
Mycoplasma_maculosum	-	-	-
Piscirickettsiaceae_bacterium_CG18_big_fil_WC_8_21_14_2_5	-	-	-
Mycoplasma_leonicaptivi	-	-	-
Chromobacterium_violaceum	-	-	-
Sphingomonas_panacis	-	-	-
Chlamydiales_bacterium_SCGC_AB-751-O23	-	-	-
Planctomycetes_bacterium_UTPLA1	-	-	-
Pedobacter_sp._Leaf216	-	-	-
Mycoplasma_alkalescens	-	-	-
Hyaloscypha_variabilis	-	-	-
Phialophora_attae	-	-	-
Achromobacter_denitrificans	-	-	-
Desulfobacterium_autotrophicum	-	-	-
Pseudomonas_sp._W15Feb9B	-	-	-
Pseudomonas_sp._URMO17WK12:I2	-	-	-
Pseudomonas_sp._URMO17WK12:I12	-	-	-
uncultured_Thiohalocapsa_sp._PB-PSB1	-	-	-
uncultured_Flavobacterium_sp.	-	-	-
Candidatus_Magasanikbacteria_bacterium_RIFOXYC12_FULL	-	-	-
Hanstruepera_crassostreae	-	-	-
Halostella_sp._LT12	-	-	-
Enterococcus_sp._HSIEG1	-	-	-
Enterococcus_sp._HMSC072H05	-	-	-
Wolinella_succinogenes	-	-	-
Wuhan_sharpbelly_bornavirus	-	-	-
Bradyrhizobium_sp._URHD0069	-	-	-
marine_gamma_proteobacterium_HTCC2148	-	-	-
Histoplasma_capsulatum	-	-	-
Bradyrhizobium_elkanii	-	-	-
Holophagae_bacterium	-	-	-
Alloscardovia_criceti	-	-	-
Candidatus_Marinimicrobia_bacterium_CG_4_10_14_0_2_um_	-	-	-
Heterobasidion_irregulare	-	-	-
Weissella_halotolerans	-	-	-
Paraburkholderia_kirstenboschensis	-	-	-
uncultured_Pleomorphomonas_sp.	-	-	-
Helicocarpus_griseus	-	-	-

Hymenobacter_rubripertinctus	-	-	-
Psilocybe_cyanescens	-	-	-
Janthinobacterium_sp._1_2014MBL_MicDiv	-	-	-
Deltaproteobacteria_bacterium_RBG_16_47_11	-	-	-
Vibrio_crassostreae	-	-	-
uncultured_bacterium_Lq_007_G03	-	-	-
Ferriphaselus_amnicola	-	-	-
Deltaproteobacteria_bacterium_RIFCSPLOWO2_02_FULL_53_	-	-	-
Acetobacter_indonesiensis	-	-	-
Deltaproteobacteria_bacterium_RIFOXYA12_FULL_61_11	-	-	-
Ferriphaselus_sp._R-1	-	-	-
Deinococcus_radiophilus	-	-	-
Erythrobacter_sp._QSSC1-22B	-	-	-
Paenarthrobacter_aurescens	-	-	-
Erysiphe_necator	-	-	-
Pigmentiphaga_sp._H8	-	-	-
uncultured_bacterium_fosmid_pJB92C9	-	-	-
Exiguobacterium_sp._U13-1	-	-	-
Alphaproteobacteria_bacterium_MarineAlpha9_Bin7	-	-	-
Ideonella_sakaiensis	-	-	-
IC4_retrovirus	-	-	-
Alteromonas_sp._KUL49	-	-	-
Flavobacteriaceae_bacterium_(ex_Bugula_neritina_AB1)	-	-	-
Bovine_gammaherpesvirus_4	-	-	-
Candidatus_Micrarchaeota_archaeon_CG10_big_fil_rev_8_21_	-	-	-
Blumeria_graminis	-	-	-
Amycolatopsis_kentuckyensis	-	-	-
Ignisphaera_aggregans	-	-	-
Amycolatopsis_balhimycina	-	-	-
Candidatus_Nomurabacteria_bacterium_GW2011_GWE2_40_1	-	-	-
Pseudonocardiaceae_bacterium_YIM_PH_21723	-	-	-
Ignavibacteria_bacterium_GWA2_35_9	-	-	-
Candidatus_Nitrosopelagicus_sp.	-	-	-
Candida_orthopsilosis	-	-	-
Halomonas_sp._PR-M31	-	-	-
Candidatus_Magasanikbacteria_bacterium_CG1_02_41_34	-	-	-
Pontibacter_actiniarum	-	-	-
Acuticoccus_kandeliae	-	-	-
Actinotignum_timonense	-	-	-
Encephalitozoon_hellem	-	-	-
bacterium_enrichment_culture_clone_N47	-	-	-
Pontogeneia_inermis	-	-	-
Paxillus_involutus	-	-	-
Gemmatimonadaceae_bacterium_4484_173	-	-	-
Enterococcus_devriesei	-	-	-
Burkholderiales_bacterium_PBB4	-	-	-
Elapid_1_orthobornavirus	-	-	-
Elioraea_tepidiphila	-	-	-
Elsinoe_australis	-	-	-
Elusimicrobia_bacterium_CG_4_8_14_3_um_filter_50_9	-	-	-
Actinobacillus_succinogenes	-	-	-
Acinetobacter_ursingii	-	-	-
Prosthecochloris_sp._HL-130-GSB	-	-	-
Candidatus_Eisenbacteria_bacterium_RBG_16_71_46	-	-	-
Candidatus_Falkowbacteria_bacterium_CG1_02_37_44	-	-	-

Gallionellales_bacterium_35-53-114	-	-	-
Candidatus_Contendobacter_odensis	-	-	-
candidate_division_WS6_bacterium_GW2011_GWE2_33_157	-	-	-
Ectothiorhodospira_marina	-	-	-
candidate_division_MSBL1_archaeon_SCGC-AAA259I09	-	-	-
Proteobacteria_bacterium_CG1_02_64_396	-	-	-
endosymbiont_of_Galathealinum_brachiosum	-	-	-
Halobacillus_hunanensis	-	-	-
Zavarzinia_sp._HR-AS	-	-	-
Halarchaeum_sp._CBA1220	-	-	-
Paraglaciecola_sp._MB-3u-78	-	-	-
endosymbiont_of_unidentified_scaly_snail_isolate_Monju	-	-	-
Pseudomonas_nitroreducens	-	-	-
Flavobacterium_antarcticum	-	-	-
Zygosaccharomyces_rouxii	-	-	-
Candidatus_Jorgensenbacteria_bacterium_GWA1_54_12	-	-	-
Yersiniaceae_bacterium_2016Iso4	-	-	-
Halomonas_cupida	-	-	-
Pseudomonas_sp._GL14	-	-	-
Candidatus_Latescibacteria_bacterium_4484_107	-	-	-
Yersiniaceae_bacterium_2015Iso6	-	-	-
Candidatus_Aminicenantes_bacterium_RBG_13_62_12	-	-	-
Yarrowia_lipolytica	-	-	-
Pezoloma_ericae	-	-	-
Candidatus_Gracilibacteria_bacterium_CG1_02_38_174	-	-	-
Gordonia_rhizosphera	-	-	-
Penicillium_expansum	-	-	-
Diaporthe_ampelina	-	-	-
Burkholderia_sp._BDU8	-	-	-
Candidatus_Bathyarchaeota_archaeon_RBG_13_52_12	-	-	-
Polynucleobacter_necessarius	-	-	-
Geotoga_petraea	-	-	-
Acidobacteria_bacterium_KBS_146	-	-	-
Desulfuromonas_soudanensis	-	-	-
Pseudomonas_antarctica	-	-	-
[Candida]_haemulonis	-	-	-
Candidatus_Heimdallarchaeota_archaeon_LC_3	-	-	-
[Enterobacter]_lignolyticus	-	-	-
archaeon_GW2011_AR18	-	-	-
Niabella_soli	-	-	-
Streptomyces_sp._or20	-	-	-
Chelativorans_sp._BNC1	-	-	-
Streptomyces_uncialis	-	-	-
Marinilactibacillus_psychrotolerans	-	-	-
Sulfitobacter_mediterraneus	-	-	-
Nitrosomonas_sp._Nm166	-	-	-
Sulfolobus_islandicus	-	-	-
Thalassotalea_crassostreae	-	-	-
Chitinophaga_caeni	-	-	-
Bacillus_siamensis	-	-	-
Mesoflavibacter_aestuarii	-	-	-
Melanopsichium_pennsylvanicum	-	-	-
Neocallimastix_frontalis	-	-	-
Neochlamydia_sp._TUME1	-	-	-
Neonectria_ditissima	-	-	-

Saccharomycetaceae_sp._'Ashbya_aceri'	-	-	-
Saccharomyces_kudriavzevii	-	-	-
Streptomyces_sp._DJ	-	-	-
Chitinophaga_parva	-	-	-
Lysinibacillus_jejuensis	-	-	-
Bacteroidetes_bacterium_OLB9	-	-	-
Cobetia_crustatorum	-	-	-
Cognatiyoonia_koreensis	-	-	-
Tetrapisispora_phaffii	-	-	-
Lactococcus_fujiensis	-	-	-
Rhodothermus_profundi	-	-	-
Nocardioides_sp._Root79	-	-	-
Legionella_spiritensis	-	-	-
Luteococcus_japonicus	-	-	-
Synechococcus_sp._8F6	-	-	-
Leifsonia_sp._Root112D2	-	-	-
Leptospira_wolbachii	-	-	-
Leptolyngbya_sp._IPPAS_B-1204	-	-	-
Lentzea_waywayandensis	-	-	-
Mesorhizobium_sp._M2C.T.Ca.TU.002.02.1.1	-	-	-
Sporosarcina_sp._P7	-	-	-
Spraguea_lophii	-	-	-
Spathaspora_sp._JA1	-	-	-
Micromonospora_yangpuensis	-	-	-
Stanieria_sp._NIES-3757	-	-	-
Micromonospora_endolithica	-	-	-
Mycoplasma_elephantis	-	-	-
Mycoplasma_gallisepticum	-	-	-
Microcoleus_vaginatus	-	-	-
Smittium_simulii	-	-	-
Mitsuaria_sp._HWN-4	-	-	-
Sphingomonas_parapaucimobilis	-	-	-
Mycobacterium_angelicum	-	-	-
Chrysiogenes_arsenatis	-	-	-
Sphingomonas_sp._Leaf20	-	-	-
Sphingomonas_sp._CCH5-D11	-	-	-
Mumia_flava	-	-	-
Spiroplasma_poulsonii	-	-	-
Chryseobacterium_piperi	-	-	-
Mycoplasma_collis	-	-	-
Moesziomyces_antarcticus	-	-	-
Bacillus_swezeyi	-	-	-
Sphingobacteriales_bacterium_UTBCD1	-	-	-
Mycoplasma_columborale	-	-	-
Methanolobus_vulcani	-	-	-
Methanolobus_sp._T82-4	-	-	-
Nannocystis_exedens	-	-	-
Streptomyces_alni	-	-	-
Mesorhizobium_waimense	-	-	-
Microbacterium_sp._CQ0110Y	-	-	-
Sideroxydans_sp._GWF2_59_14	-	-	-
Methylophaga_aminisulfidivorans	-	-	-
Mycoplasma_pirum	-	-	-
Methylocaldum_sp._14B	-	-	-
Methylobacter_sp.	-	-	-

Corallococcus_sp._CA043D	-	-	-
Rhodobacter_ovatus	-	-	-
Archaeoglobus_fulgidus	-	-	-
Thermotogales_bacterium_46_20	-	-	-
Thermus_oshimai	-	-	-
Aquimarina_sp._MAR_2010_214	-	-	-
Ogataea_parapolymorpha	-	-	-
Rhizophydium_sphaerotheca	-	-	-
Coxiellaceae_bacterium_RA15029	-	-	-
Candidatus_Shapirobacteria_bacterium_CG09_land_8_20_14_0	-	-	-
Bifidobacterium_coryneforme	-	-	-
Rhodospirillaceae_bacterium_TMED167	-	-	-
Candidatus_Taylorbacteria_bacterium_RIFCSPHIGHO2_01_FU	-	-	-
Armatimonadetes_bacterium_CG06_land_8_20_14_3_00_66_2	-	-	-
Oceanithermus_profundus	-	-	-
Arhodomonas_aquaeolei	-	-	-
Lactobacillus_hokkaidonensis	-	-	-
Bifidobacterium_adolescentis_CAG:119	-	-	-
Sphingomonas_taxi	-	-	-
Candidatus_Ryanbacteria_bacterium_CG10_big_fil_rev_8_21_1	-	-	-
Tsuchiyaea_wingfieldii	-	-	-
Tuber_melanosporum	-	-	-
Tupanvirus_soda_lake	-	-	-
Uromyces_hobsonii	-	-	-
Oryzisolibacter_propanilivorax	-	-	-
Oscillatoria_acuminata	-	-	-
Kosakonia_sacchari	-	-	-
Thiothrix_sp.	-	-	-
Kosakonia_oryzae	-	-	-
Cyberlindnera_jadinii	-	-	-
Betaproteobacteria_bacterium_HGW-Betaproteobacteria-4	-	-	-
Thermobifida_cellulosilytica	-	-	-
Candidatus_Yanofskybacteria_bacterium_RIFCSPHIGHO2_02_	-	-	-
Asanoa_ishikariensis	-	-	-
Nostoc_minutum	-	-	-
Candidatus_Woesearchaeota_archaeon_CG10_big_fil_rev_8_21	-	-	-
Arthrospira_platensis	-	-	-
Asticcacaulis_sp._AC466	-	-	-
Oceanibacterium_hippocampi	-	-	-
Ascochyta_rabiei	-	-	-
Comamonas_kerstersii	-	-	-
Aspergillus_brasiliensis	-	-	-
Thermococcus_sp._AM4	-	-	-
Devosia_limi	-	-	-
Micrococcaceae_bacterium_C1-50	-	-	-
Sneathia_amnii	-	-	-
Candidatus_Arthromitus_sp._SFB-4	-	-	-
Cyanobacteria_bacterium_SW_9_44_58	-	-	-
Flavobacterium_album	-	-	-
Microbulbifer_marinus	-	-	-
Demequina_sp._NBRC_110052	-	-	-
Candidatus_Arthromitus_sp._SFB-1	-	-	-
Cyanobacteria_bacterium_J007	-	-	-
Pyricularia_oryzae	-	-	-
Blastomonas_sp._CCH1-A6	-	-	-

Planctomycetes_bacterium_RIFCSPHIGHO2_02_FULL_50_42	-	-	-
Algoriphagus_sp._32-45-6	-	-	-
Rheinheimera_sp._EpRS3	-	-	-
Campylobacter_sp._10_1_50	-	-	-
Chryseobacterium_solincola	-	-	-
Morganella_sp._EGD-HP17	-	-	-
Phenylobacterium_sp._Root700	-	-	-
Tessaracoccus_sp._T2.5-30	-	-	-
Leptographium_sp._'qinlingensis'	-	-	-
Spathaspora_passalidarum	-	-	-
Verrucomicrobiae_bacterium_Tous-C2TDCM	-	-	-
Candidatus_Accumulibacter_sp._SK-02	-	-	-
Laetiporus_sulphureus	-	-	-
Puccinia_cf._psidii_AE-2014	-	-	-
Vibrio_quintilis	-	-	-
Lyngbya_aestuarii	-	-	-
Arcobacter_sp._CECT_8983	-	-	-
Limnohabitans_sp._DM1	-	-	-
Dermacoccaceae_bacterium_RIT621	-	-	-
Massilia_sp._K1S02-61	-	-	-
Pseudoalteromonas_elyakovii	-	-	-
Pseudoalteromonas_sp._JB197	-	-	-
Fusarium_scirpi	-	-	-
Providencia_burhodogranariea	-	-	-
Helicobacter_canadensis	-	-	-
Bifidobacteriaceae_bacterium_WP012	-	-	-
Enterobacter_roggenkampii	-	-	-
Thioclava_arenosa	-	-	-
Polynucleobacter_sp._VK13	-	-	-
Aspergillus_aculeatus	-	-	-
Olleya_aquimaris	-	-	-
Methylocystis_rosea	-	-	-
Methanoculleus_horonobensis	-	-	-
Bacteroidetes_bacterium_GWF2_49_14	-	-	-
Chloroflexi_bacterium_GWC2_73_18	-	-	-
Sulfuricaulis_limicola	-	-	-
Pseudomonas_zhaodongensis	-	-	-
Pontibacter_virosus	-	-	-
Synechococcus_sp._CC9311	-	-	-
Bacillus_sp._AFS098217	-	-	-
Neisseria_sp._83E34	-	-	-
Swinepox_virus	-	-	-
Candidatus_Thioglobus_sp._TMED218	-	-	-
