# Supplementary material for: Angelica polysaccharides relieve blood glucose levels in diabetic KKAy mice possibly by modulating gut microbiota: an integrated gut microbiota and metabolism analysis
Source: BMC Microbiol. 2023 Oct 3;23:281. doi: 10.1186/s12866-023-03029-y (PMC10546737; doi:10.1186/s12866-023-03029-y)
Supplement: Supplementary file 6 — Additional file 6: Supplementary Table 3. The significant altered KEGG pathway by AP treatment. [file 12866_2023_3029_MOESM6_ESM.docx]

Supplementary Table 3. The significant altered KEGG pathway by AP treatment

| ID | logFC | se | pvalues |
| --- | --- | --- | --- |
| Limonene and pinene degradation | 0.516926 | 0.127276 | 4.88E-05 |
| Meiosis - yeast | 0.99424 | 0.303178 | 0.00104 |
| 2-Oxocarboxylic acid metabolism | 0.254378 | 0.084702 | 0.002671 |
| Nucleotide excision repair | 0.217363 | 0.076396 | 0.004438 |
| Mismatch repair | 0.172818 | 0.064373 | 0.007261 |
| Bacterial secretion system | 0.188943 | 0.070939 | 0.007734 |
| DNA replication | 0.18181 | 0.071966 | 0.011526 |
| Microbial metabolism in diverse environments | 0.139331 | 0.055211 | 0.011616 |
| Biosynthesis of antibiotics | 0.158369 | 0.065937 | 0.016314 |
| Homologous recombination | 0.160052 | 0.06674 | 0.016479 |
| Novobiocin biosynthesis | 0.558233 | 0.247205 | 0.023934 |
| Monobactam biosynthesis | 0.123019 | 0.057339 | 0.031914 |
| Caprolactam degradation | 1.004046 | 0.468769 | 0.032203 |
| RNA polymerase | 0.21313 | 0.103592 | 0.039648 |
| Terpenoid backbone biosynthesis | 0.140727 | 0.069242 | 0.042114 |
| Selenocompound metabolism | 0.144224 | 0.071475 | 0.043609 |
| Ribosome | 0.16221 | 0.080662 | 0.044326 |
| Lysine degradation | 0.260874 | 0.07917 | 0.000984 |
| Tryptophan metabolism | 0.328908 | 0.099962 | 0.001001 |
| Valine, leucine and isoleucine biosynthesis | 0.159112 | 0.054749 | 0.003659 |
| Biosynthesis of amino acids | 0.165353 | 0.057099 | 0.003781 |
| Galactose metabolism | 0.168227 | 0.058219 | 0.003858 |
| Glycolysis / Gluconeogenesis | 0.180154 | 0.067866 | 0.007941 |
| Amino sugar and nucleotide sugar metabolism | 0.129465 | 0.05036 | 0.010147 |
| Fructose and mannose metabolism | 0.164582 | 0.06408 | 0.010217 |
| Carbon metabolism | 0.168381 | 0.066567 | 0.011422 |
| Cysteine and methionine metabolism | 0.23511 | 0.09411 | 0.012482 |
| Sphingolipid metabolism | 0.208399 | 0.08351 | 0.012578 |
| Butanoate metabolism | 0.188944 | 0.077858 | 0.015234 |
| Methane metabolism | 0.137118 | 0.056801 | 0.015778 |
| Alanine, aspartate and glutamate metabolism | 0.236712 | 0.099736 | 0.017626 |
| Biosynthesis of secondary metabolites | 0.153939 | 0.065301 | 0.018405 |
| Arginine biosynthesis | 0.288393 | 0.122388 | 0.018454 |
| Pyruvate metabolism | 0.16866 | 0.072307 | 0.019672 |
| Phenylalanine, tyrosine and tryptophan biosynthesis | 0.231377 | 0.099835 | 0.020472 |
| Arginine and proline metabolism | 0.363421 | 0.159361 | 0.022579 |
| Purine metabolism | 0.14383 | 0.065295 | 0.027612 |
| Metabolic pathways | 0.149132 | 0.067955 | 0.028195 |
| Tyrosine metabolism | 0.542831 | 0.247668 | 0.028396 |
| Aminoacyl-tRNA biosynthesis | 0.183591 | 0.084357 | 0.029528 |
| Pantothenate and CoA biosynthesis | 0.124235 | 0.058601 | 0.034005 |
| Nitrogen metabolism | 0.190137 | 0.09003 | 0.034693 |
| Citrate cycle (TCA cycle) | 0.205576 | 0.098397 | 0.036686 |
| Lysine biosynthesis | 0.106719 | 0.05153 | 0.038359 |
| Phenylalanine metabolism | 0.391856 | 0.195093 | 0.044583 |
| Starch and sucrose metabolism | 0.123075 | 0.06145 | 0.045193 |
| Glutathione metabolism | 0.190858 | 0.095768 | 0.046271 |
| Protein processing in endoplasmic reticulum | 0.813855 | 0.410165 | 0.047232 |
| Glyoxylate and dicarboxylate metabolism | 0.178263 | 0.090102 | 0.047877 |
