# Supplementary material for: Angelica polysaccharides relieve blood glucose levels in diabetic KKAy mice possibly by modulating gut microbiota: an integrated gut microbiota and metabolism analysis
Source: BMC Microbiol. 2023 Oct 3;23:281. doi: 10.1186/s12866-023-03029-y (PMC10546737; doi:10.1186/s12866-023-03029-y)
Supplement: Supplementary file 7 — Additional file 7: Supplementary Table 4. The differential metabolites between. [file 12866_2023_3029_MOESM7_ESM.pdf]

Supplementary Table 4. The differential metabolites betwee

CT vs HFD up regulated meta	CT vs HFD down regulated n	HFD vs AP up regulated meta	HFD vs AP down regulated m
neg_1015; 5-Ethyl-2-(1-pyrrc	neg_1002; Muricin E	neg_1037; (R)-3-Hydroxy-he	neg_11; 2,3-Butanedione
neg_1026; 4-Hydroxy-4-meth	neg_1010; 5-(2-Furanyl)-1,2,	neg_1040; Pipercitine	neg_1148; 7,8-Dihydroyango
neg_1044; Fumonisin AK1	neg_1013; Triterpenoid	neg_1046; 7-Methyl-3-methy	neg_1151; cis- and trans-Ethy
neg_105; Phe-Gly-OH	neg_1014; TG(10:0/10:0/10:(	neg_1049; Curzerenone	neg_1169; 10,11-dihydro-20-
neg_1054; 2,4-Dihydroxy-2H	neg_1017; xi-Dihydro-5-octy	neg_1093; Glyceric acid	neg_1237; Kanokoside D
neg_1064; Decanal	neg_1020; Rubraflavone C	neg_1104; 6,8-Tricosanedion	neg_1261; Myrcenol
neg_1075; 4-Ethyl-2-methoxy	neg_1022; Digoxigenin monc	neg_1217; Propyl 1-(propylth	neg_1285; trans-p-Menth-2-er
neg_1078; Kamahine C	neg_1023; 1alpha-1-Hydroxy	neg_1249; Apo-12'-violaxant	neg_1458; Arecaidine
neg_1081; Desogestrel	neg_1024; 4-Acetoxy-2-hexy	neg_1347; (Z)-15-Oxo-11-eic	neg_1504; beta-Citraurin
neg_1085; AF Toxin II	neg_1031; Sativan	neg_1391; 28-Hydroxywithar	neg_1513; 5-Methoxytryptopl
neg_1104; 6,8-Tricosanedion	neg_1035; Glycerol 1-(5-hyd	neg_14; Serotonin hydrochlo	neg_154; 4-Gingerol
neg_1115; 3a,7b,21-Trihydro	neg_1046; 7-Methyl-3-methy	neg_1477; 2,5-Dimethyl-3-m	neg_1598; N-Desmethyltrama
neg_1140; Alectrol	neg_1047; (3S,7E,9S)-9-Hyd	neg_1505; L-Palmitoylcarniti	neg_1619; Phytolaccinic acid
neg_1148; 7,8-Dihydroyango	neg_1050; D-Glycero-D-gala	neg_1529; LysoPE(0:0/20:4(8	neg_1728; Megastigmatrienor
neg_1169; 10,11-dihydro-20-	neg_1051; Troxerutin	neg_1532; Perindoprilat gluc	neg_1833; Asimilobin
neg_117; 8,11-eicosadiynoic	neg_1053; trans-Jasmone	neg_1546; Esculentic acid (P	neg_1905; 19'-Hexanoyloxym
neg_1172; Lysyl-Tryptophan	neg_1058; 4-Methyl-2-(1-phe	neg_1553; 2,6 Dimethylhepta	neg_207; Artabsinolide D
neg_1179; 4-[(2-Methyl-3-fu	neg_1062; 3a,7a-Dihydroxy-5	neg_157; 6-Gingerol	neg_2095; Nigellic acid
neg_1191; Vulgarolide	neg_1066; Hovenine A	neg_1641; 5-Decanoyl-2-non	neg_2184; Ginsenoside Rh8
neg_1208; 1-Pentadecanecarl	neg_1073; Hordatine A	neg_1680; (S)-[10]-Gingerol	neg_2267; Ciliatine
neg_1212; Lentinic acid	neg_1079; Linoleic acid	neg_1690; LysoPE(0:0/18:0)	neg_2286; all-trans-Decapren
neg_1217; Propyl 1-(propylth	neg_108; p-Acetaminobenzal	neg_1691; Metabolite M6	neg_2371; cis-3-Hexenyl hexa
neg_1220; 5-Hexyltetrahydro	neg_1088; D-Glucose	neg_1703; Lisuride	neg_2406; Naringenin
neg_1257; 13-Hydroxy-9-me	neg_1095; Mukonal	neg_1727; 4-Methyl-4-(meth	neg_2567; 3,5-Dimethyl-2-vi
neg_1259; Anatabine	neg_1113; (3R,7R)-1,3,7-Oct	neg_1771; PS(14:0/16:0)	neg_2645; 3-(1,1-Dimethylall
neg_1272; Vanilloloside	neg_1117; Cavipetin D	neg_1786; D8'-Merulinic acic	neg_2703; Methysergide
neg_1276; Momorcharaside I	neg_113; Nanaomycin	neg_1924; 7-HDoHE	neg_2705; Cocaethylene
neg_1278; Panaquinquecol 1	neg_1133; Methyl 3,4,5-trime	neg_1952; PG(18:1(11Z)/20:	neg_2744; Hexylresorcinol
neg_1285; trans-p-Menth-2-e	neg_1136; Oxytetracycline	neg_1967; 10-Hydroxy-3-me	neg_2750; Bambuterol
neg_1301; (E)-Arachidin II	neg_1153; (8R,8'R,9S)-9-Hyc	neg_1999; Glabrolide	neg_2831; trans-p-Menthane-
neg_1307; Neopellitorine A	neg_1157; Neomycin	neg_2028; 24,25-Diacetylvul	neg_2900; Gossypurpurin
neg_1321; Asperxanthone	neg_1162; Neuromedin N (1-	neg_2033; PC(15:0/16:0)	neg_2913; LysoPE(0:0/20:1(1
neg_1323; Ivacaftor	neg_1164; Piperalol	neg_2060; PC(22:6(4Z,7Z,10	neg_294; 5-Dodecenoic acid
neg_134; Lovastatin acid (Me	neg_1180; Zileuton	neg_2061; Docosapentaenoic	neg_295; (E,Z)-2,4-Heptadier
neg_1347; (Z)-15-Oxo-11-eic	neg_1192; 6-Methylnicotinan	neg_222; 13-Hydroxymarasm	neg_2988; Pyronaridine
neg_1359; Decarbamoylneosa	neg_1194; D-1-[(3-Carboxyp	neg_2233; Scopolamine	neg_3017; Annopentocin A
neg_1363; Phenylalanyl-Isole	neg_1198; PE(20:5(5Z,8Z,11	neg_2251; (3beta,22E,24R)-I	neg_3056; Glutaminyl-Methic
neg_1390; Fluvoxamine	neg_1200; Anandamide	neg_2310; 14-oxo-DoHE(1-)	neg_310; 2-Carboxy-4-dodec
neg_14; Serotonin hydrochlo	neg_1207; Androstenedione	neg_2319; Solasodine	neg_3233; PGP(16:0/18:0)
neg_140; (卤)伪-CMBHC	neg_1213; Aloesol 7-glucosic	neg_2323; Falcarindione	neg_3247; PC(DiMe(11,5)/M
neg_1418; 6-Hydroxymelator	neg_123; maltotetraose	neg_235; N-(14-Methylhexac	neg_3297; 2',6'-Dihydroxy-4'-
neg_1420; All-trans-retinoic a	neg_1230; N-Glycolylneuram	neg_2413; PS(MonoMe(11,3	neg_3361; PE(18:0/20:3(5Z,8
neg_1432; Tomatidine	neg_1234; Dihomo-gamma-li	neg_2450; PE(15:0/P-18:0)	neg_3370; PC(DiMe(11,3)/M
neg_1434; O-Desmethyltrama	neg_1238; xi-7-Hydroxyhexa	neg_246; Docosapentaenoic a	neg_3468; L-Oxalylalbizziine
neg_1446; PGF2a ethanolami	neg_1239; Dihydrozeatin-7-N	neg_2466; Torvoside F	neg_3522; 15-keto-Prostaglar
neg_1449; Petasalbin	neg_1249; Apo-12'-violaxant	neg_2486; PG(16:0/22:5(7Z,	neg_3546; Valyl-Histidine
neg_1458; Arecaidine	neg_1253; 1-Undecanol	neg_2497; Musabalbisiane B	neg_358; 1-docosanoyl-glyce
neg_1459; 11'-Carboxy-alpha	neg_1254; 12-Methyltridecan	neg_2513; Dictyoquinazol C	neg_3582; 4-Oxoretinol
neg_149; Diacetyl-10-ginger	neg_1255; PC(20:5(5Z,8Z,11	neg_253; 8-Oxohexadecanoic	neg_374; 12-oxo-20-dihydrox
neg_151; ALPINETIN METI	neg_1262; L-Glutamine	neg_2594; Ethyl (卤)-2-meth	neg_402; Isopropyl beta-D-gl
neg_1510; Menthol	neg_1265; 4-Hydroxy-3-meth	neg_2673; PS(18:0/22:6(4Z,7	neg_411; Dibutyl malate
neg_1511; 1-Phenyl-1,3-hept	neg_1269; (E,E,E)-1,3,5,11-T	neg_2739; Saccharopine	neg_439; (S)-3-Methylthiohe
neg_152; Gamma-Glu-Leu	neg_127; 11-deoxy-PGF1伪	neg_2764; (2E,4E)-2,4-Octac	neg_461; Soyasapogenol D
neg_1539; Arginyl-Gamma-g	neg_1275; Valsartan	neg_2765; 3a,20b-Pregnaned	neg_519; cis-3-Hexenyl pyru
neg_154; 4-Gingerol	neg_1295; N-Oleoyl tyrosine	neg_2816; Trihexyphenidyl	neg_566; 1-Acetoxy-4,6-tetra
neg_1560; 2-hydroxyethinyle	neg_1300; Pantoyllactone glu	neg_2861; Ropivacaine	neg_578; Nepetariaside

neg_157; 6-Gingerol	neg_1302; Inositol 1,3,4,5-tet	neg_2946; Melilotoside D	neg_59; Quadrone
neg_1579; E-10-Hydroxyami	neg_1312; Ethanolaminium(1	neg_2951; 3-Methyl-5-pentyl	neg_596; alpha-Micropteroxa
neg_1586; L-Leucine	neg_1335; 2-Acetylpyrrole	neg_2969; PE(14:0/20:4(5Z,8	neg_676; [8]-Paradol
neg_1589; PE(20:2(11Z,14Z)	neg_1336; 5-Acetylamino-6-f	neg_3029; Isoleucyl-Asparag	neg_743; Oxyphencyclimine
neg_16; L-Tryptophan	neg_1342; Plastoquinone 3	neg_306; Theaspirone A	neg_749; Moracin O
neg_1624; Procyclidine	neg_1345; Furohyperforin	neg_3069; Terniflorin	neg_80; 19(R)-hydroxy-PGA
neg_1628; Dehydrocyanaropi	neg_1362; Calenduloside B	neg_3072; Heptylmalonic aci	neg_823; Lysyl-Proline
neg_1635; Indecainide	neg_1367; Balenine	neg_3077; 2-Methoxy-5-meth	neg_912; (1S,2S,4S,5R)-1,8-I
neg_1640; (10Z,14E,16E)-10	neg_1370; DG(16:0/14:1(9Z)	neg_308; 2-Hydroxy-6-tridec	neg_934; Bisacurol
neg_1668; Tacrolimus	neg_1376; 4-Methoxy-3-gera	neg_3129; Pipericine	neg_942; Reticulataxanthin
neg_1676; beta-Citraurinene	neg_138; 15-Keto-protagland	neg_3213; 3b,17b-Dihydroxy	neg_957; alpha-Butyl-omega-
neg_1680; (S)-[10]-Gingerol	neg_1383; 2-O-(4-O-Methyl-	neg_3217; Medicoside I	pos_1007; Carisoprodol
neg_1688; 2-Isopropyl-5-met	neg_1384; 3'-Ketolactose	neg_3287; Dimethylallylpyro	pos_1020; 1-(3-Aminopropyl
neg_1693; 3-carboxy-4-methy	neg_139; Cellotetraose	neg_3298; 20a,22b-Dihydrox	pos_1043; 2-Heptanone
neg_1703; Lisuride	neg_1394; Hexylbenzene	neg_3325; (5S)-hydroperoxy-	pos_106; PG(20:5(5Z,8Z,11Z
neg_1706; (5alpha,10alpha)-3	neg_1400; L-Galacto-2-heptu	neg_3352; Crotamiton	pos_1074; 10,20-Dihydroxye
neg_1754; 2-oleoyl-sn-glycer	neg_1403; 3alpha,4,5,7alpha-	neg_3400; 6beta-Hydroxytest	pos_1081; Humulinic acid A
neg_176; Val Asp Ile Arg	neg_1404; Isophysalin G	neg_3418; Nonadecanoic acid	pos_1095; 3alpha-O-trans-Fe
neg_177; Sequiterpene Lacto	neg_1407; Italipyrone	neg_3428; Helinorbisabone	pos_111; 9Z,12E-Tetradecadi
neg_1771; PS(14:0/16:0)	neg_1408; L-Pyridosine	neg_3429; Fludiazepam	pos_1110; N(2)-phenylacetyl-
neg_1774; Anhydrocinnzeyla	neg_1426; Gamma-CEHC	neg_3443; Yucalexin P21	pos_1138; Cellobiose
neg_1778; 8,12-Epoxy-4(15)	neg_1435; Uzarigenin 3-[xylo	neg_3466; CDP-DG(16:0/16:	pos_1157; 3-Methyl-3-buten-
neg_1785; 6-Hydroxyfluvasta	neg_1437; (1E,4Z,6a,8b,10a)	neg_3490; Asitrilobin D	pos_1178; 2-Methylcyclodod
neg_1786; D8'-Merulinic acid	neg_1438; Phomopsin A	neg_3535; Methyl linolenate	pos_1179; (E)-2-Octen-1-ol
neg_1788; Dirithromycin	neg_1440; (卤)-threo-1-(p-Hy	neg_371; 2-Methylbutyrylgly	pos_1353; 2,3-Dihydro-4-met
neg_1789; PE(20:5(5Z,8Z,11	neg_1441; DG(15:0/20:5(5Z,	neg_377; N-Jasmonoylisoleu	pos_1402; Morphinone
neg_179; 15(S)-HpEPE	neg_1444; Quinquenoside III	neg_380; Ustiloxin D	pos_1565; (S)-Nerolidol 3-O-
neg_1796; 6-Amino-9H-purin	neg_1451; 3'-Sialyl-3-fucosyl	neg_431; Dihydrodigoxin	pos_1569; Alpha-ketoisovaler
neg_1798; Gibberellin A94	neg_1452; Methyl 7-epi-12-h	neg_478; (2'E,4'Z,7'Z,8E)-Co	pos_1603; 3-(Acetyloxy)-2-hy
neg_1801; 1-(beta-D-Ribofur	neg_1453; Prednisolone	neg_568; 15(R)-hydroperoxy	pos_1680; 1-(2-Furanyl)-2-pr
neg_1815; N6-Methylagmatin	neg_1461; Peltatol C	neg_571; Ganodermic acid T	pos_1714; xi-5-Hydroxydode
neg_1825; 3a,4b,7a-Trihydro	neg_1465; Prostaglandin C1(	neg_574; 5-(10,13-Nonadeca	pos_1748; 1-Hydroxy-3-meth
neg_1833; Asimilobin	neg_1475; 1,1'-(Tetrahydro-6	neg_575; (ent-2alpha,3beta,1	pos_1798; E-10-Hydroxynort
neg_1873; Didesmethyl doxe	neg_1480; 3-Acetyl-2,5-dime	neg_585; 2-Palmitoylglycero	pos_1807; (3beta,5alpha,6bet
neg_1903; Sphingosine 1-pho	neg_1482; DG(20:3(5Z,8Z,1	neg_604; MG(0:0/20:1(11Z)/	pos_182; 9E,11-Dodecadiena
neg_1905; 19'-Hexanoyloxyn	neg_1488; 6-Hydroxyshogaol	neg_631; Homocitrulline	pos_1953; 2-Hexyl-4,5-dimet
neg_1914; Cyperine	neg_1495; Egonol gentiobios	neg_640; 2,3-Dinor-6-keto-pr	pos_1964; 7-Hydroxygliclazi
neg_1917; Methyl dihydrojas	neg_1498; 3-Butylidene-1(3H	neg_662; 1-Nonen-3-ol	pos_1979; beta-D-Glucosylox
neg_1924; 7-HDoHE	neg_1501; Mocimycin	neg_663; PE(14:1(9Z)/22:5(4	pos_2000; Polypodosaponin
neg_1936; N2-Galacturonyl-I	neg_1512; Fumonisin A2	neg_714; Kanzonol M	pos_2016; 13-HOTE
neg_194; 2-(N-morpholino)et	neg_1517; Prazepam	neg_783; ent-8(17),13(16),14	pos_2030; DG(20:0/14:0/0:0)
neg_1944; Prostaglandin H1	neg_1522; (3S,5R,6S,7E,9x)-	neg_820; (R)-2-Hydroxyhexa	pos_2073; 6-Deoxyfagomine
neg_1951; 3-Nonen-5-one	neg_153; Phe Asp Arg Asp	neg_839; 15-dehydro-prostag	pos_2086; 1-Arachidonoylgly
neg_1953; Auberganol	neg_1532; Perindoprilat gluc	neg_840; Pregnanediol	pos_2120; 14alpha-Hydroxyi
neg_1990; 4-Hydroxystachyd	neg_1535; Arbekacin	neg_860; Desglucocheirotoxi	pos_219; Glycerylphosphoryl
neg_202; Leu Asn Arg Glu	neg_1545; Litcubinine	neg_889; 3-Oxooctadecanoic	pos_221; 12-OPDA
neg_2021; 19-Oxoandrost-4-e	neg_1546; Esculentic acid (P	neg_943; 3-Ethenyl-4-hydrox	pos_2299; 2-Hexaprenyl-3-m
neg_203; METAXALONE	neg_1548; Gingerenone C	neg_964; Sagittariol	pos_2398; 25-Acetyl-6,7-dide
neg_2033; PC(15:0/16:0)	neg_155; Nabumetone	pos_1014; 4-Hydroxy-all-tran	pos_2476; Nicotinic acid mon
neg_2037; Caffeoylcycloarte	neg_1551; 3-Hydroxy-N6,N6	pos_1036; Acetaminophen cy	pos_254; Oleoyl-EA(d2)
neg_204; Glycerol 1-propano	neg_1558; L-isoleucyl-L-prol	pos_1058; 3-Methyl-alpha-io	pos_2550; Dihydro-2-methox
neg_2042; Crispanone	neg_1563; 2-Hydroxy-6-pent	pos_1108; L-Cysteine	pos_2574; Squamoxinone
neg_2046; PE(20:0/16:0)	neg_1577; 12b-Hydroxy-5b-c	pos_1206; (6S)-5-formyltetra	pos_2576; Paricalcitol
neg_2047; Cardoltriene	neg_159; Nitrotyrosine	pos_1212; Fasciculol C	pos_2586; Alpha-Heptasacch
neg_2053; Oryzalexin E	neg_1594; Gentian Violet	pos_1268; Ginsenoside F5	pos_259; 2,4-Undecadienal
neg_2059; Oxacyclotetradeca	neg_1601; 2-Methoxyestrone	pos_1330; PI(16:0/18:1(11Z)	pos_2649; Malonoben
neg_2060; PC(22:6(4Z,7Z,10	neg_1609; Calenduloside E	pos_1340; Cyclocalopin D	pos_2713; Lucidenic acid B
neg_2061; Docosapentaenoic	neg_1612; (E)-3-Heptenyl 2-	pos_1350; AS 1-5	pos_2867; Cholest-5-ene

neg_207; Artabsinolide D	neg_1617; Piperochromenoic	pos_1423; (S)-4',7-Dihydroxy	pos_2868; Hoduloside IX
neg_2078; Leukotriene B4 et	neg_1623; Cortexolone	pos_1429; Kiwiionoside	pos_2899; D-Sedoheptulose 7
neg_2089; PG(18:0/20:3(5Z,8	neg_1630; Nitroxoline	pos_1437; PE(18:3(6Z,9Z,12	pos_2908; Notoginsenoside R
neg_2091; 3alpha,7alpha,12a	neg_1631; PI(16:0/20:2(11Z,	pos_1442; Sildenafil	pos_291; 9Z,12Z-Octadecadi
neg_2102; Imidapril	neg_1661; Serinyl-Tryptopha	pos_1463; Hexaethylene glyc	pos_2946; (S)-[8]-Gingerol
neg_2103; Ineketone	neg_1675; Soyasapogenol B	pos_152; Val Ile Ile Asp	pos_2950; Ganoderic acid J
neg_213; Rishitin	neg_1681; Melilotoside B	pos_1527; Gingerglycolipid	pos_2984; N5-(3,4-Dioxo-1,5
neg_2133; Gestrinone	neg_1689; MG(18:0/0:0/0:0)	pos_1567; PE(22:1(13Z)/22:	pos_2989; L-Proline
neg_2141; PE(20:2(11Z,14Z)	neg_1696; 45,46,47-Trinorye	pos_1587; Protodioscin	pos_3039; 4-Methyl-2-pentyl-
neg_2154; Arginyl-Lysine	neg_17; 16-Hydroxypalmitic	pos_1624; 3-Hydroxyvaleric	pos_3054; Macrophorin A
neg_2159; N-Docosahexaeno	neg_1700; 6beta-hydroxybud	pos_1628; PC(24:1(15Z)/P-1	pos_3091; 5-Hydroxy-2-(5-m
neg_2188; Falcarinone	neg_1708; Murrayenol	pos_1629; Monoglucuronylgl	pos_3115; Nandrolone
neg_2197; Vulgarone A	neg_1711; 1-Ethyl-1H-pyrrol	pos_1685; DG(18:4(6Z,9Z,12	pos_3123; 3,4-Dimethyl-5-pe
neg_2200; trans-O-Methylgra	neg_173; (+)-Isomyristic acid	pos_1686; 3b-Allotetrahydro	pos_3134; Glyyunnansapogen
neg_2226; 2-(14,15-Epoxyeic	neg_1733; Nebularine	pos_1688; Oleoylcarnitine	pos_3193; (S)-4',5,7-Trihydro
neg_2227; Cinncassiol D2	neg_1734; Didymin	pos_1706; Valorphin	pos_3229; 5-Fluorouridine m
neg_2233; Scopolamine	neg_175; 1-Palmitoyl Lysoph	pos_1717; Hebevinoside V	pos_3337; Nonoxynol-9
neg_2234; 1-(5Z,8Z,11Z,14Z	neg_1750; Physapubenolide	pos_1720; TR-Saponin C	pos_3347; DG(20:5(5Z,8Z,11
neg_2240; 3,5,5-Trimethyl-2-	neg_1759; 20-Trihydroxy-leu	pos_1721; Galabiosylceramic	pos_3377; PE(15:0/24:0)
neg_2247; N-(Heptan-4-yl)be	neg_1777; L-N-(1H-Indol-3-	pos_1724; 19-hydroxyprostag	pos_3460; Ginsenoside Rc
neg_225; Beta-Alanine	neg_1779; delta3,5-Deoxytig	pos_1730; Ganoderic acid M	pos_3513; Ornithokinin
neg_2251; (3beta,22E,24R)-E	neg_1782; 8(S)-HPETE	pos_1738; Mulberranol	pos_353; (6Z,9Z)-6,9-Pentad
neg_2256; Cyperotundone	neg_1793; Dulcoside A	pos_1742; Squamosten A	pos_3560; Dodecyl butyrate
neg_2295; Cholestane-3,7,12	neg_1794; Nb-Methyltetrahy	pos_1745; 2-Heptanethiol	pos_3612; Eicosadienoic acid
neg_2308; 8-Acetylegelolide	neg_1800; Chlorogenoquinor	pos_1761; PS(18:2(9Z,12Z)/	pos_3632; N-[(4E,8E)-1,3-dih
neg_2312; Cyprodinil	neg_1803; Ganoderic acid A	pos_1846; Tsugaric acid A 2	pos_3636; 15-Acetoxyscirpen
neg_2323; Falcarindione	neg_181; IRIFLOPHENONE	pos_1863; PE(24:0/18:3(6Z,9	pos_3638; Phenformin
neg_2334; Cilastatin	neg_1810; Dihydrojasmone	pos_1864; PE(20:0/20:5(5Z,8	pos_3661; 4-(Glutamylamino)
neg_235; N-(14-Methylhexad	neg_1817; Docosahexaenoyl	pos_1865; Soyasaponin aa	pos_3760; L-4-Chlorotryptop
neg_2363; TG(22:6(4Z,7Z,10	neg_1822; Isolimonic acid 16	pos_1904; beta-Doradecin	pos_3767; Valyl-Glutamate
neg_2364; Bimatoprost	neg_1823; Phenytoin methylc	pos_1906; Blasticidin S	pos_383; Glycyrrhiza flavono
neg_2367; PGP(18:1(11Z)/22	neg_1834; 9alpha-(3-Methylb	pos_1980; Corchorusoside A	pos_3842; L-2,3-Dihydrodipi
neg_2386; 1,6-Hexanedithiol	neg_1838; 2-(3-Methylbutyl)-	pos_2022; Coutaric acid	pos_3928; Celastrol
neg_2390; N-[(4-Hydroxy-3-	neg_1842; 2-Hydroxy-6-(8,1	pos_2054; LysoPE(0:0/24:0)	pos_3982; 2-(Ethylsulfonylme
neg_2412; 3beta,6beta-Dihyd	neg_1844; 5-hydroxy-2-oxo-4	pos_2069; Polymyxin B Sulfa	pos_4035; 1-O-Sinapoylgluco
neg_2424; 3-Methylhistamine	neg_1845; Oleoside dimethyl	pos_2097; Crassostrea Secoc	pos_4040; 5-Androstene-3b,1
neg_2426; (2xi,3xi)-2,3-Dihy	neg_186; Glutaric acid	pos_2133; (R)-Laudanidine	pos_4047; Gluten exorphin C
neg_2431; Arachidyl carnitin	neg_1866; Koryoginsenoside	pos_2139; 11-beta-Hydroxya	pos_4067; Benazepril
neg_2436; 16b-Hydroxystanc	neg_1867; Maltoheptaose	pos_2172; CDP-DG(16:0/20:	pos_4073; Trihexosylceramid
neg_2465; cis-4-Decenoic aci	neg_1868; Tracheloside	pos_218; His Ile Leu Trp	pos_4173; 3-Methylcyclopent
neg_249; 3-Hydroxy-2-methy	neg_187; Lys-Lys-OH	pos_2193; Roxatidine acetate	pos_4189; 7-Hydroxy-2-meth
neg_2501; Janthitrem C	neg_1882; Vicine	pos_2213; PE(22:5(7Z,10Z,1	pos_4306; DG(14:0/24:1(15Z
neg_2502; PS(18:3(9Z,12Z,1	neg_1884; Halobetasol Propi	pos_2214; PS(16:0/20:4(5Z,8	pos_4324; N-Methyltryptamin
neg_2508; PIP(18:2(9Z,12Z)/	neg_1888; Histidinyl-Serine	pos_2234; Ginsenoside F2	pos_4331; Gonyautoxin VI
neg_2521; Pondaplin	neg_1892; (+)-Galeon	pos_2237; AzII	pos_4343; 3-(10-Heptadeceny
neg_2540; DG(15:0/18:3(9Z,	neg_1897; 4-HDoHE	pos_2263; PC(18:2(9Z,12Z)/	pos_435; 2-Methylpropanoyl
neg_2563; PS(DiMe(13,5)/M	neg_19; Methyl vanillate	pos_2264; PC(22:4(7Z,10Z,1	pos_4359; Betaine aldehyde
neg_2567; 3,5-Dimethyl-2-vi	neg_1907; 2-(trans-2-Penteny	pos_2325; Saringosterol 3-glu	pos_4361; 7-Keto-8-aminope
neg_2571; N-Acetyl-b-glucos	neg_1909; 2-Oxo-3-hydroxy-	pos_2338; 2-Dodecylbenzene	pos_4414; Helenalin
neg_2572; Eriojaposide B	neg_1921; (-)-(E)-1-(4-Hydro	pos_2343; Lucidenic acid G	pos_492; 2-Methylpropanal C
neg_2578; PE(20:3(5Z,8Z,11	neg_1945; Robustocin	pos_2366; Fumonisin B2	pos_513; Spisulosine
neg_2594; Ethyl (卤)-2-methy	neg_1947; DG(14:1(9Z)/15:0	pos_2369; Galabiosylceramic	pos_537; 5-Aminosalicylic A
neg_2599; Hebevinoside VIII	neg_1948; Sorbitan oleate	pos_2382; Cabergoline	pos_580; HEUDELOTTIN C
neg_2606; 1-(2,3-Dihydro-6-	neg_1958; 14alpha-Hydroxyp	pos_2403; Leucyl-Serine	pos_595; Polyoxyethylene (6
neg_2616; Muricatin C	neg_1964; Phenylpropanolam	pos_2405; omega-hydroxyfin	pos_606; Nitramine
neg_2635; Dihydrocumambri	neg_1968; Mabioside C	pos_2427; CDP-DG(16:0/22:	pos_649; 11-Hydroxycanthin-
neg_264; alpha-Amylcinnamy	neg_197; 3-hydroxy-hexadec	pos_2471; dIDP	pos_673; PI(22:5(4Z,7Z,10Z,
neg_2645; 3-(1,1-Dimethylal	neg_1970; PE(22:4(7Z,10Z,1	pos_2489; Azythromycin	pos_686; 4,2'-Dihydroxychalc

neg_266; cis-[8]-Shogaol	neg_1979; Macrophorin B	pos_2543; Ganodermic acid I	pos_80; 1-Pyrroline
neg_2695; 3-O-Sulfogalactos	neg_1987; a-L-Arabinofurano	pos_2563; (3b,5a,25R)-3-Hy	pos_824; (Z)-1,5-Tridecadien
neg_2697; Cotinine methoniu	neg_1989; N-Acetyl-D-gluco	pos_2614; PIP2(18:3(9Z,12Z	pos_866; Citronellyl cinnama
neg_2703; Methysergide	neg_1993; 5,8-Epoxy-5,8-dih	pos_2628; Pseudoginsenoside	pos_906; 2,5-Dimethyl-3(2H)
neg_271; 14,19-Dihydroaspic	neg_1995; Ginsenoside B2	pos_2653; Trigoneoside Xb	pos_926; Shyobunol
neg_2718; Enalapril	neg_2010; Rivastigmine	pos_2657; Spirolide F	pos_972; 3-Furanmethanol gl
neg_272; Asperagenin	neg_2050; Methionyl-Threon	pos_2668; Maslinic acid 3-O-b-D-glucoside	
neg_2727; PS(18:3(9Z,12Z,1	neg_2067; PIP(16:0/22:5(7Z,	pos_2678; 3-O-Sulfogalactosylceramide (d18:1/24:1(15Z))	
neg_273; [8]-Gingerdione	neg_2088; (3beta,9beta)-7-D	pos_2694; Jurubine	
neg_2733; Tetrahydrocortiso	neg_209; trans-2-Hexyl-1-cy	pos_2697; Cervonoyl ethanolamide	
neg_2744; Hexylresorcinol	neg_2096; Isopetasoside	pos_271; Erinacine B	
neg_2747; Clidinium	neg_2100; Maltol	pos_2718; S-Formylglutathione	
neg_2750; Bambuterol	neg_211; Avocadyne	pos_2762; Kentsin	
neg_2751; PE(DiMe(11,5)/D	neg_2121; 12,13-DHOME	pos_2766; 5'-((Z)-Feruloyl) 3-(2'-methylarabinosylxylose)	
neg_278; Octanal	neg_2134; 12-hydroxyicosan	pos_2776; (3b,16a,21b,22a)-12-Oleanene-3,16,21,23,28-pe	
neg_2781; 6-[2,3-Dihydroxy-	neg_214; Tetracosatetraenoic	pos_2777; 3-Oxotetradecanoic acid	
neg_281; [6]-Dehydroshogao	neg_2148; Gemcitabine	pos_2824; (E)-1-Cinnamoylpyrrolidine	
neg_2810; Cepagenin	neg_2151; 5'-Methylthioaden	pos_2840; Trihexosylceramide (d18:1/20:0)	
neg_2812; Buspirone N-oxid	neg_2152; Vitamin A2 aldeh	pos_2857; 1-Oleoylglycerophosphoinositol	
neg_282; Isoleucyl-Methioni	neg_2165; Alliosterol 1-rham	pos_2892; Centellasaponin C	
neg_2822; Aldosine	neg_2171; Brassilexin	pos_2894; Dehydroaporheine	
neg_2853; L-2-Amino-5-(me	neg_218; d-Tocotrienol	pos_2910; Ginsenoside Rg5	
neg_2861; Ropivacaine	neg_2194; Prostaglandin E1	pos_2911; 4,4-Dimethyl-14a-formyl-5a-cholesta-8,24-dien-	
neg_29; Corticosterone	neg_2203; 28-[Glucosyl-(1->	pos_2973; Goyaglycoside e	
neg_2929; Arabsin	neg_2204; Hydroxyprolyl-Cy	pos_3007; 1alpha,3beta,22R-Trihydroxyergosta-5,24E-dien	
neg_293; 5b-Cholestane-3a,7	neg_2208; Liquiritigenin 4'-[	pos_3055; TG(14:0/22:4(7Z,10Z,13Z,16Z)/18:4(6Z,9Z,12Z	
neg_2943; 22b-Hydroxychol	neg_2209; 3-(4-Hydroxy-3-m	pos_3064; Tertatolol	
neg_295; (E,Z)-2,4-Heptadie	neg_2212; Isomammein	pos_3070; PS(20:4(5Z,8Z,11Z,14Z)/22:6(4Z,7Z,10Z,13Z,1	
neg_2956; Methyl aminolevu	neg_222; 13-Hydroxymarasm	pos_3114; (2E,4E,6Z)-2,4,6-Decatrienoic acid dehydropipe	
neg_2983; PI(18:2(9Z,12Z)/1	neg_2228; Gibberellin A3	pos_3166; alpha-Solamarine	
neg_2991; Glutamyl-Leucine	neg_223; 5b-Cholestane-3a,7	pos_3175; Retrocalamin	
neg_3014; PG(18:3(6Z,9Z,12	neg_2230; Chrysophanol 8-g	pos_3182; PS(DiMe(9,3)/DiMe(9,3))	
neg_302; Ilicifolinoside A	neg_2235; Methyl (Z,Z)-10-h	pos_3202; M-Secociguatoxin 4A	
neg_304; 7-Aminonitrazepam	neg_2238; N-Arachidonoyl t	pos_3224; Isepamicin	
neg_3052; UDP-4-dehydro-6	neg_2252; 1-Acetyl-3,27-dih	pos_3272; 4'-N-desmethylolanzapine	
neg_306; Theaspirone A	neg_2255; 4alpha-Formyl-4b	pos_3381; Achyranthoside C	
neg_3067; LysoPC(18:4(6Z,9	neg_2280; Enalaprilat	pos_340; 15-Octadecene-9,11,13-triynoic acid	
neg_3079; PG(16:1(9Z)/18:2	neg_2281; 6-Hydroxy-4,6-dir	pos_342; Soyasaponin I	
neg_3086; PC(20:5(5Z,8Z,11	neg_2286; all-trans-Decapren	pos_3461; PE(20:0/22:6(4Z,7Z,10Z,13Z,16Z,19Z))	
neg_3109; Dehydroascorbide	neg_2289; Torvoside D	pos_3466; Alliospiroside C	
neg_3121; S-(3-Methylbutan	neg_2296; Polyporusterone C	pos_3515; Anidulafungin	
neg_3126; Decyl propionate	neg_2307; Dityrosine	pos_3519; Sativoside R2	
neg_315; 11-Hydroxy-12-me	neg_2316; PC(P-16:0/22:2(1	pos_3540; 1,7-Dimethylguanosine	
neg_3153; DG(20:4(8Z,11Z,	neg_233; 3-Hydroxyisovaler	pos_3570; Notoginsenoside R6	
neg_316; 11beta-Hydroxytes	neg_2332; 3-Hydroxy-9-hexa	pos_3575; Isobutyl N-methylanthranilate	
neg_3170; Epsilon-(gamma-C	neg_2337; 2-Polyprenyl-3-m	pos_3618; Pisatoside	
neg_3186; Capsoside A	neg_2338; Cotinine glucuron	pos_3653; Octyl 2-furoate	
neg_3190; Acorusnol	neg_2339; Lc3Cer	pos_3674; Sucrose acetate isobutyrate	
neg_3207; TG(14:1(9Z)/18:4	neg_2348; 18-Oxocortisol	pos_3683; PG(16:0/16:1(9Z))	
neg_3213; 3b,17b-Dihydroxy	neg_2355; Diethylcarbamazi	pos_3694; DG(15:0/22:0/0:0)	
neg_3221; Prostaglandin F3a	neg_2356; Prostaglandin E3	pos_3696; Oleanolic acid 3-[rhamnosyl-(1->4)-glucosyl-(1-	
neg_3233; PGP(16:0/18:0)	neg_2370; 20-COOH-leukotr	pos_3716; Lansioside B	
neg_3246; Carboxy-ibuprofe	neg_2378; Caraganoside A	pos_3728; PC(15:0/20:2(11Z,14Z))	
neg_3253; 2-Methylbutyroyl	neg_241; 5-Nonyltetrahydro-	pos_3773; Dynorphin A 9-17	
neg_328; Tryptamine	neg_2413; PS(MonoMe(11,3	pos_3775; (S)-4',5,7-Trihydroxy-6-prenylflavanone	
neg_3293; 2-Pyrrolidineaceti	neg_242; 2-Dodecenal	pos_384; CDP-DG(18:1(9Z)/22:6(4Z,7Z,10Z,13Z,16Z,19Z	
neg_3297; 2',6'-Dihydroxy-4'	neg_2425; Coriandrone D	pos_3868; PE(18:3(6Z,9Z,12Z)/22:6(4Z,7Z,10Z,13Z,16Z,1	
neg_3306; DG(15:0/16:1(9Z)	neg_2434; Dehydrocarpaine I	pos_3903; 2-Amino-5-benzoylbenzimidazole	

neg_3309; Dimethylbenzimid	neg_2445; 3'-(gamma,gamma	pos_3918; Erinacine G
neg_331; Calamendiol	neg_2449; Mabioside A	pos_3950; Physalolactone B
neg_3312; Glutamyl-Serine	neg_2452; Butorphanol	pos_3984; alpha-[3-[(Hydroxymethyl)nitrosoamino]propyl]
neg_332; Ascaridole	neg_2453; 2-Ethoxy-5-methy	pos_4043; PE(22:0/22:6(4Z,7Z,10Z,13Z,16Z,19Z))
neg_3322; PS(18:3(9Z,12Z,1	neg_246; Docosapentaenoic a	pos_4046; PIP2(18:2(9Z,12Z)/20:2(11Z,14Z))
neg_3325; (5S)-hydroperoxy-	neg_2462; 9-Oxooctadecanoi	pos_4048; Mulberrofuran T
neg_3326; 17-Hydroxyproges	neg_2463; Bisoprolol	pos_4077; PE(18:3(6Z,9Z,12Z)/22:1(13Z))
neg_3347; Ganoderic acid th	neg_2467; Ergocalciferol	pos_4101; Tocophersolan
neg_3350; Violanone	neg_2471; Gibberellin A54	pos_4112; Quinquenoside IV
neg_3359; 5-Dehydroepisterc	neg_2473; PIP2(16:0/18:2(9Z	pos_4127; Poppy acid
neg_3364; 3beta-Acetoxy-11a	neg_2476; 8-HDoHE	pos_4138; Stevioside
neg_3408; 3',5-Dihydroxy-4',	neg_2479; Suspensolide F	pos_4152; Piperochromanoic acid
neg_341; 5-Hydroxy-p-menth	neg_2480; Idarubicin	pos_4249; CDP-DG(18:0/20:4(8Z,11Z,14Z,17Z))
neg_3410; 11-Oxooctadecanc	neg_2487; Palmitoylstigmast	pos_4251; Soyasaponin II
neg_3440; Methyl 10-undece	neg_2493; N-Carbamoyl-2-ar	pos_4284; PIP(18:2(9Z,12Z)/18:1(11Z))
neg_3442; Myristic acid	neg_25; Sebacic acid	pos_4335; Pefloxacin N-oxide
neg_3443; Yucalexin P21	neg_2500; Prostaglandin D1	pos_4350; PE(20:1(11Z)/24:0)
neg_3448; PC(22:0/22:4(7Z,1	neg_251; 6-Hydroxypentadec	pos_4365; (+-)-Mevalonolactone
neg_3449; (3beta,19alpha)-3,	neg_2515; Dide-O-methylsim	pos_4372; 11(Z),14(Z)-Eicosadienoic Acid
neg_3452; TG(20:4(5Z,8Z,11	neg_252; 4-Hydroxy-3-(16-m	pos_44; Wogonin
neg_347; 4-Hydroxy-3-methc	neg_2520; Quinic acid	pos_442; PE(12:0/16:1(9Z))
neg_3472; Ethyl (3R,5Z)-3-h	neg_2522; omega-Hydroxym	pos_466; Panaquinquecol 4
neg_349; Coprocholic acid	neg_2530; Kudzusaponin SA	pos_588; 3-Dimethylallyl-4-hydroxymandelic acid
neg_3499; Imidaprilat	neg_2541; 1-(7Z,10Z,13Z,16	pos_594; PS(12:0/19:0)
neg_350; Trimipramine	neg_2544; 2,3-Dimethyl-2-cy	pos_610; (17Z)-1伪,25-dihydroxy-26,27-dimethyl-17,20,22
neg_3516; Pyridostigmine ca	neg_255; Imazamethabenz	pos_655; PE(16:0/22:5(4Z,7Z,10Z,13Z,16Z))
neg_3522; 15-keto-Prostaglan	neg_2552; 2-(1-Pentenyl)fura	pos_706; PE(15:0/15:1(9Z))
neg_3531; MG(18_2(9Z,12Z	neg_2553; Corchorosol A	pos_74; cis-12-Octadecenoic Acid methyl ester
neg_3532; Compactin	neg_2557; Oleanolic acid	pos_750; Quinaprilat
neg_3541; Valyl-Leucine	neg_2569; Heterophyllin	pos_797; 4-(Methylthio)-1-butanol
neg_3545; 11-Deoxycorticost	neg_2590; Dehydrocarpaine I	pos_819; Pro Val Ser Leu
neg_3546; Valyl-Histidine	neg_2609; (E)-3-(Tetrahydro	pos_848; 12-Hydroxy-12-octadecanoylcarnitine
neg_3553; 13,14-Dihydro-15	neg_2613; 15-Hydroxyleptoc	pos_991; Austroinulin
neg_3554; Tridecanoic acid (	neg_2614; Lysyl-Valine	
neg_3555; Venlafaxine	neg_2621; Araliasaponin V	
neg_3556; 5-Hydroxytryptop	neg_2622; Linalyl anthranilate	
neg_3566; Piperidione	neg_2626; 2,4-Dihydroxy-6,7-dimethoxy-2H-1,4-benzoxazin-3(4H)-one	
neg_3572; p-Tolualdehyde	neg_2629; 2,3-Dihydroxy-1-(4-hydroxy-3-methoxyphenyl)-1-propanone	
neg_3580; Misoprostol (free	neg_2631; 5'-Phosphoribosyl-N-formylglycinamide	
neg_3582; 4-Oxoretinol	neg_2642; 3-trans-p-Coumaroylrotundic acid	
neg_3583; Nervonic acid	neg_265; Cucurbic acid	
neg_3588; Jasmonic acid	neg_2651; Methyl acrylate-divinylbenzene, completely hydrolyzed, copolymer	
neg_359; Gabapentin	neg_2657; Kaempferol 7-sophoroside	
neg_3604; (-)-Usnic acid	neg_2662; Theogallin	
neg_374; 12-oxo-20-dihydro	neg_2663; L-Fucose	
neg_380; Ustiloxin D	neg_2675; 20-Dihydrodydrogesterone	
neg_386; (3S,6E)-Nerolidol	neg_2690; (鹵)-3',4'-Methylenedioxy-5,7-dimethylepicatechin	
neg_394; Neohesperidose	neg_2692; N-Desmethyl sildenafil (UK-103,320)	
neg_4; Pantothenate	neg_2698; Alpha-linolenyl carnitine	
neg_400; (S)-3-Methylthiohe	neg_2701; Amprenavir	
neg_402; Isopropyl beta-D-gl	neg_2728; Calenduloside H methyl ester	
neg_406; Antibiotic X 14889	neg_2738; (8R,8'R)-Secoisolariciresinol 9,9'-bis-[4-carboxy-3-hydroxy-3-methylbutanoyl-	
neg_422; Prostaglandin F2a	neg_2757; Zanthobisquinolone	
neg_425; 19-Hydroxytestoste	neg_277; 9-Decenoic acid	
neg_430; ACRL Toxin II	neg_2771; Muricoreacin	
neg_437; 3-keto Fusidic acid	neg_2775; Trigofoenoside F	
neg_446; (-)-trans-Carveol gl	neg_2777; 5-O-beta-D-Xylopyranosyl-L-arabinose	
neg_455; Notoginsenoside J	neg_2793; TG(15:0/20:5(5Z,8Z,11Z,14Z,17Z)/22:6(4Z,7Z,10Z,13Z,16Z,19Z))	

neg_458; Oleoyl glycine	neg_2799; L-2-Amino-3-(oxalylamino)propanoic acid
neg_463; Fagomine	neg_2800; Salsolinol 1-carboxylate
neg_464; 3-Methylcrotonylgl	neg_2806; Eremopetasitenin B2
neg_473; Ginkgoic acid	neg_2824; Goshonoside F4
neg_478; (2'E,4'Z,7'Z,8E)-Co	neg_2829; 6-Caffeoylsucrose
neg_483; 1,3-Diphenylpropan	neg_283; 1-Octen-3-yl glucoside
neg_484; 5-(14-Nonadecenyl	neg_2846; Albanin H
neg_496; 2-Methylacetophen	neg_2851; Oxytocin 1-8
neg_498; alpha-Zearalenol	neg_2855; 4,6-Heptadiyne-1,3-diol
neg_500; Tyrosyl-Isoleucine	neg_2856; Propyl propane thiosulfonate
neg_508; 5'-Carboxy-gamma-	neg_2882; 11-Hydroxy-9-tridecenoic acid
neg_514; Oleic acid	neg_2884; Nigellimine N-oxide
neg_521; Cobinamide	neg_2885; DL-Ornithino-L-alanine
neg_522; Prolyl-Tyrosine	neg_291; MG(0:0/14:1(9Z)/0:0)
neg_532; Pantothenic acid	neg_2920; Tragopogonsaponin C
neg_535; 5-(8,11-Pentadecad	neg_2923; L-phenylalanyl-L-hydroxyproline
neg_540; 1,5,5,8-Tetramethyl	neg_2933; Negletein 6-[rhamnosyl-(1->2)-fucoside]
neg_541; 5beta-Cholestane-3	neg_2937; L-2-Amino-3-(1-pyrazolyl)propanoic acid
neg_542; Phytocassane B	neg_294; 5-Dodecenoic acid
neg_55; 未-CEHC	neg_2946; Melilotoside D
neg_562; 3-Decenoic acid	neg_2955; 1'-Acetoxyeugenol acetate
neg_566; 1-Acetoxy-4,6-tetra	neg_2958; L-alpha-glutamyl-L-hydroxyproline
neg_574; 5-(10,13-Nonadeca	neg_2959; 2-Decarboxybetanin
neg_583; (14S)-14,15-Dihydr	neg_296; Aflatoxin B1 dialcohol
neg_59; Quadrone	neg_2961; Cajaisoflavone
neg_591; (3alphaOH,20S,24S	neg_2967; Threoninyl-Phenylalanine
neg_602; Rhazidigenine Nb-o	neg_2969; PE(14:0/20:4(5Z,8Z,11Z,14Z))
neg_604; MG(0:0/20:1(11Z)/	neg_2973; PC(22:4(7Z,10Z,13Z,16Z)/24:1(15Z))
neg_613; Alfacalcidol	neg_2978; 8-Hydroxyoctanoate
neg_618; 3-Oxo-1,4,11(13)-e	neg_2981; 4-hydroxy ketorolac
neg_626; Polyporusterone A	neg_2982; 5'-Hydroxy-3',4',7-trimethoxyflavan
neg_628; 3-Nonanon-1-yl ace	neg_3006; C.I. Natural Red 20
neg_633; Didanosine	neg_301; Menthyl ethylene glycol carbonate
neg_642; NAPQI	neg_3020; Adrenorphin
neg_655; (Z,Z)-2,9,16-Hepta	neg_3021; 2-Hexenoylcholine
neg_684; Isoleucyl-Lysine	neg_3025; 1,2,3,4-Tetrahydro-2-methyl-b-carboline
neg_692; 3-hydroxytridecano	neg_3026; Lactucin
neg_695; Cinchonidine	neg_305; Jasmolone
neg_696; Arginyl-Arginine	neg_3050; 14R,15S-EpETrE
neg_705; Copalic acid	neg_3051; Niaziminin A
neg_719; 3,7-Dihydroxy-12-o	neg_3055; S-nirvanol
neg_737; Allyl thiohexanoate	neg_3064; Withaperuvin H
neg_743; Oxyphencyclimine	neg_3068; Prostaglandin E2
neg_746; Sterebin A	neg_307; Setariol
neg_76; 伪-Phenylcyclohexyl	neg_3070; 3b,8b-Dihydroxy-6b-(3-chloro-2-hydroxy-2-methylbutanoyloxy)-7(11)-eremo
neg_762; 5-Phenyl-1,3-oxazi	neg_3078; 3-Hydroxymethylantipyrine
neg_764; PC(MonoMe(11,3)/	neg_3082; Acrimarine N
neg_772; Resveratrol 4'-(6-ga	neg_3101; 16-Hydroxy hexadecanoic acid
neg_783; ent-8(17),13(16),14	neg_311; Falcarindiol
neg_784; (22Alpha)-hydroxy-	neg_3111; 1,2-Diacylglycerol-LD-PI-pool
neg_791; Histidinyl-Isoleucin	neg_3118; 5(S)-Hydroperoxyeicosatetraenoic acid
neg_792; Secobarbital	neg_3131; 8-oxo-dGDP
neg_794; (14alpha,17beta,20	neg_3133; Ethyl 3-hydroxyoctanoate O-[glucosyl-(1->6)-glucoside]
neg_80; 19(R)-hydroxy-PGA	neg_3145; N-(1-Deoxy-1-fructosyl)tyrosine
neg_802; Absindiol	neg_3146; gamma-L-Glutamyl-L-methionine sulfoxide
neg_803; Citreovirenone	neg_3154; 10,12-Pentacosanedione
neg_806; 1b,3a,7b-Trihydrox	neg_3162; Fistuloside B
neg_813; Talinolol	neg_317; Hexyl acetate

neg_823; Lysyl-Proline	neg_3180; 4-Hydroxy-3-polyprenylbenzoate
neg_828; 24-Ethyllophenol	neg_3193; (S)-Oleuropeic acid
neg_829; 16-Acetylpriveroge	neg_3200; 2-Ethyldihydro-3(2H)-thiophenone
neg_83; 1,11-Undecanedicarl	neg_3202; Ethyl 2,4-dioxohexanoate
neg_869; 13'-Carboxy-gamma	neg_3204; LysoPE(0:0/18:4(6Z,9Z,12Z,15Z))
neg_877; (S)-Verimol F	neg_3217; Medicoside I
neg_884; 17a,21-Dihydroxy-5	neg_3220; Thiamine monophosphate
neg_892; Glyuranolide	neg_3222; Edultin
neg_894; 2-Acetyl-5-methylf	neg_3224; Methylphenidate
neg_897; Prolyl-Arginine	neg_3225; 3-Hydroxyglabrol
neg_898; thymidine 3'-monop	neg_3226; Chlorophyll d
neg_899; D-Glucuronic acid	neg_3228; 3-Hydroxy-beta-ionol 3-[glucosyl-(1->6)-glucoside]
neg_900; Furanofukinin	neg_3230; Ganoderic acid H
neg_908; 3-Acetoxyscirpene-	neg_3243; Hydroxyclomipramine glucuronide
neg_910; Valdiate	neg_3247; PC(DiMe(11,5)/MonoMe(11,3))
neg_924; 2beta,9xi-Dihydrox	neg_325; 4,6-Pentacosanedione
neg_929; CD 1790	neg_3257; Hexadecanedioic acid mono-L-carnitine ester
neg_957; alpha-Butyl-omega-	neg_3258; TG(20:5(5Z,8Z,11Z,14Z,17Z)/18:1(9Z)/22:6(4Z,7Z,10Z,13Z,16Z,19Z))[iso6]
neg_96; N-Alpha-acetyllysine	neg_3271; 4'-hydroxytrazodone
neg_961; Clarithromycin	neg_3289; N-[2-(4-Prenyloxyphenyl)ethyl]tiglamide
neg_962; 22-Acetylpriveroge	neg_329; Phenylethanolaminium
neg_973; (+)-(S)-Carvone	neg_3300; Withaperuvin E
neg_975; Methionyl-Proline	neg_3301; xi-7-Octen-2-ol
neg_979; Ganoderic acid G	neg_3311; (E,E)-Boviquinone 3
neg_999; PI(16:1(9Z)/16:1(9	neg_3313; Cysteinyl-Glutamate
pos_100; Val Ile Ile	neg_3315; Gentisin
pos_1004; Porrigenin A	neg_3318; PC(18:1(11Z)/22:6(4Z,7Z,10Z,13Z,16Z,19Z))
pos_1005; (Z)-4-Heptenal	neg_3329; PS(DiMe(11,3)/MonoMe(13,5))
pos_1009; Wyerone	neg_333; 5a-Cholestane-3a,7a,12a,25-tetrol
pos_1010; D-Alanyl-D-alanin	neg_3334; MG(0:0/20:0/0:0)
pos_1014; 4-Hydroxy-all-tran	neg_334; Lepidiumterpenyl ester
pos_1015; 5,6-Dihydro-11-m	neg_3345; beta-D-Galactopyranosyl-(1->4)-2-amino-2-deoxy-beta-D-glucopyranosyl-(1->
pos_102; Pro Pro Ala Thr	neg_335; 1-heptadecanoyl-glycero-3-phosphate
pos_1021; 2-Acetyl-3-ethylid	neg_3351; [6]-Gingerdiol 3-acetate
pos_1024; Ethinamate	neg_3358; DG(20:5(5Z,8Z,11Z,14Z,17Z)/22:2(13Z,16Z)/0:0)
pos_1027; 3,4,5,6-Tetrahydro	neg_336; delta-Methylionone
pos_1072; xi-2,3-Dihydro-3,5	neg_3372; Amylopectin
pos_1077; (卤)-2-Hydroxy-4-	neg_3394; L-Menthyl (R,S)-3-hydroxybutyrate
pos_1081; Humulinic acid A	neg_3404; P1,P4-Bis(5'-uridyl) tetraphosphate
pos_1083; 3-keto-Digoxigeni	neg_3414; 6,8-Heneicosanedione
pos_1103; Valerianol	neg_3421; Niazicinin A
pos_1120; meta-O-Dealkylate	neg_3429; Fludiazepam
pos_1139; p-Mentha-1,3,8-tri	neg_3434; Allyl butyrate
pos_1141; 1alpha,23(S),25-tr	neg_3451; Amaranthussaponin I
pos_116; Eremolactone	neg_3460; Deoxyinosine
pos_1169; Filfiline	neg_3468; L-Oxalylalbizziine
pos_1173; Australigenin	neg_3480; Glycerol tripropanoate
pos_1180; N-Acetylanonaine	neg_3482; Schinifoline
pos_1210; Pentacosanoylglyc	neg_3488; 13E-Tetranor-16-carboxy-LTE4
pos_1213; Glutaminyl-Hydro	neg_3500; Momordicoside G
pos_1216; Diphthamide	neg_3502; Parasiloxanthin
pos_123; 3-Deoxyestradiol	neg_3504; Myricatomentoside II
pos_1231; Austalide K	neg_3505; (6b,7b,13R)-6,7-Diacetoxy-8,14-labdadiene-13-ol
pos_1247; Pyridoxamine	neg_3506; Cepanone
pos_1253; 13-L-Hydroperoxy	neg_3511; Harmalol
pos_1255; Pyrazinemethaneth	neg_3518; Deoxycoformycin
pos_1258; Idoxuridine	neg_3525; 1-Palmitoyl-2-hydroxy-sn-glycero-3-phosphoethanolamine
pos_1260; 6-Methylthiopurin	neg_3527; (+)-Abscisic acid

pos_1268; Ginsenoside F5	neg_3540; delta-Tocotrienol
pos_1270; 2-beta-hydroxyme	neg_3563; 1-Palmitoylglycerol
pos_1272; Enol-3-Ethyl-1,2-c	neg_3569; Androstendione
pos_1276; 1-Methoxy-3-meth	neg_3571; 3-Methylcyclohexanethiol
pos_1278; 24-Hydroxycholes	neg_3573; Phenylalanyl-Glutamine
pos_1281; 4-Methyl-1-pheny	neg_3578; Leucyl-Glutamine
pos_1284; 7-hydroxygraniset	neg_3579; 2'-O-methylinosine
pos_1290; Sorbitan palmitate	neg_3593; Serotonin
pos_1292; Cymorcin monogl	neg_3602; 9Z,12Z-Linoleic acid
pos_1294; 1-Acetoxy-2-hydr	neg_3603; Geldanamycin
pos_13; Duloxetine	neg_361; Ribothymidine
pos_1305; 1,4'-Bipiperidine-1	neg_37; Tetradecanedioic acid
pos_1308; 7alpha,12alpha,26	neg_377; N-Jasmonoylisoleucine
pos_133; 5伪-Androst-16-en-	neg_388; Armillaripin
pos_1330; PI(16:0/18:1(11Z)	neg_389; 3-Oxohexadecanoic acid
pos_1332; Illudin C2	neg_393; Amyl salicylate
pos_1335; Ethyl menthane ca	neg_405; (2S,2'S)-Pyrosaccharopine
pos_1337; 15(S)-Hydroxyeic	neg_407; Lyciumoside III
pos_134; (22E)-3伪,7伪,12伪	neg_415; 2,2,7,7-Tetramethyl-1,6-dioxaspiro[4.4]nona-3,8-diene
pos_1349; cis-Hydroxy Perhe	neg_418; 13-Nor-6-eremophilene-8,11-dione
pos_135; m-Xylene	neg_420; (S)-3-Octanol glucoside
pos_1354; Tetrahydropersin	neg_423; trans-p-Menthane-7,8-diol 7-glucoside
pos_1358; Mupirocin	neg_438; 5,9-Epidioxy-3-hydroxyergost-7-en-6-one
pos_136; Val Glu Val Arg	neg_440; 17,23-Epoxy-29-hydroxy-27-norlanost-8-ene-3,15,24-trione
pos_1367; 41-O-demethylrap	neg_444; alpha-D-Xylopyranosyl-(1->6)-beta-D-glucopyranosyl-(1->4)-D-glucose
pos_137; Lys Lys Met Gln	neg_449; DG(16:1(9Z)/16:1(9Z)/0:0)
pos_1388; LysoPC(18:0)	neg_454; Polyethylene, oxidized
pos_139; PG(16:0/0:0)[U]	neg_460; LysoPE(0:0/14:0)
pos_1410; Tolbutamide	neg_465; 6-beta-hydrocortisol
pos_1421; 4-Butyl-2-methylo	neg_469; Muricin H
pos_1423; (S)-4',7-Dihydroxy	neg_482; (+)-threo-2-Amino-3,4-dihydroxybutanoic acid
pos_1432; 6-Succinoaminopu	neg_486; Capecitabine
pos_1437; PE(18:3(6Z,9Z,12	neg_497; Arachidonic acid
pos_1453; Spirolide B	neg_50; 5-cis Carbaprostacyclin
pos_1465; PE(18:4(6Z,9Z,12	neg_513; (1R*,2R*,4R*,8S*)-p-Menthane-1,2,8,9-tetrol 9-glucoside
pos_1466; 1-Phenyl-1-pentan	neg_518; 8-Hydroxynevirapine
pos_147; 1伪-hydroxy-23,24,	neg_52; 2-Isopropylmalic acid
pos_1482; isobutyrylcarnitine	neg_533; Ascorbalamic acid
pos_1491; 4-(2,6,6-Trimethyl	neg_538; 3-hydroxyropivacaine
pos_1497; (2E,11Z)-Wyerone	neg_539; Ganodermatriol
pos_1511; 3-Methyl-1-(2,4,6-	neg_54; 6-keto PGE1
pos_152; Val Ile Ile Asp	neg_551; 7(14)-Bisabolene-2,3,10,11-tetrol
pos_1520; (3E,7E)-4,8,12-Tr	neg_552; Neryl glucoside
pos_1527; Gingerglycolipid I	neg_553; Goshonoside F1
pos_1538; 5-b-Cholestane-3a	neg_556; LysoPE(0:0/14:1(9Z))
pos_1540; N-Succinyl-2-ami	neg_58; (卤)12,13-DiHOME
pos_1560; Hovenidulcioside	neg_580; Dihydro-3-(2-octenyl)-2,5-furandione
pos_1577; LysoPE(0:0/24:6(	neg_581; Lamivudine-monophosphate
pos_158; 13-METHYL-4,4-E	neg_585; 2-Palmitoylglycerophosphocholine
pos_1580; Octreotide	neg_590; 12-Oxo-20-trihydroxy-leukotriene B4
pos_1581; Cloversaponin I	neg_593; MG(0:0/22:2(13Z,16Z)/0:0)
pos_1585; Alpha-Cryptoxant	neg_594; 3-hydroxyhexadecanoyl carnitine
pos_1587; Protodioscin	neg_595; 22-Angeloylbarringtogenol C
pos_1603; 3-(Acetyloxy)-2-h	neg_598; Stercurensin
pos_1604; Fucoxanthin	neg_600; (R)-Oxypeucedanin
pos_1606; 24,25,26,27-Tetra	neg_601; Ganoderic acid L
pos_1607; 3-Hydroxyhexade	neg_606; Obtustyrene
pos_1609; Arginyl-Histidine	neg_609; N-Formyl-L-methionine

pos_1617; 4,5-Dimethyl-2-he	neg_612; Procainamide
pos_1626; O-Desmethylango	neg_616; (17alpha,23S)-17,23-Epoxy-29-hydroxy-27-norlanosta-1,8-diene-3,15,24-trione
pos_1627; Ganoderiol H	neg_629; (2E,6E)-Piperamide-C7:2
pos_1629; Monoglucuronylgl	neg_630; Cyclocalopin C1
pos_1656; Kukoamine B	neg_634; N-Acetylmuramoyl-Ala
pos_1663; 3b-Hydroxy-6b-m	neg_639; Ramiprilat
pos_1669; 3beta-Acetoxy-19a	neg_645; (S)-alpha-Amino-4-carboxy-3-furanpropanoic acid
pos_1677; 2-Methyl-1-hydro:	neg_650; LysoPE(0:0/15:0)
pos_1681; Palmitoyl glucuroi	neg_656; D-Glucaro-1,4-lactone
pos_1686; 3b-Allotetrahydro	neg_66; HU-211
pos_169; 5,8,11-Octadecatriy	neg_661; eta-Tocopherol
pos_1706; Valorphin	neg_664; Achillicin
pos_1707; 2-Deoxycastastero	neg_669; hydroxyrepaglinide
pos_1717; Hebevinoside V	neg_672; (3beta,5alpha,6alpha,7alpha,22E,24R)-5,6-Epoxyergosta-8,14,22-triene-3,7-dio
pos_1720; TR-Saponin C	neg_675; MG(0:0/22:1(13Z)/0:0)
pos_1724; 19-hydroxyprostag	neg_68; (卤)-Catechin
pos_1726; 4-oxo-Retinoic aci	neg_680; Oxybuprocaine
pos_1729; PE(14:1(9Z)/18:4(	neg_682; Tavulin
pos_1732; 7-Ethyl-2,3,6,7-tet	neg_683; Myricanene B 5-[arabinosyl-(1->6)-glucoside]
pos_1757; Hydroxyhomodest	neg_693; N-Ethyl trans-2-cis-6-nonadienamide
pos_1761; PS(18:2(9Z,12Z)/:	neg_698; Calenduloside H
pos_178; Glechomanolide	neg_703; Caffeic acid
pos_1785; 4-Hydroxyretinoic	neg_706; Melleolide
pos_1802; Araliasaponin III	neg_709; Digitoxin
pos_1807; (3beta,5alpha,6bet	neg_712; (9R,10S,12Z)-9,10-Dihydroxy-8-oxo-12-octadecenoic acid
pos_1808; PG(16:0/18:3(6Z,9	neg_718; 3-hydroxyundecanoyl carnitine
pos_181; LysoPC(20:4(5Z,8Z	neg_729; trans-3-Hydroxycotinine glucuronide
pos_1820; 9alpha-(3-Methyl-	neg_736; 2,2'-Dithenyl sulfide
pos_1835; PS(20:4(5Z,8Z,11	neg_747; 5alpha-Cholestanone
pos_1836; 1,2-Dihydrodehyd	neg_75; 13,14-dihydro Prostaglandin E1-d4
pos_1842; Collettiside I	neg_753; 3-(2-Heptenyloxy)-2-hydroxypropyl undecanoate
pos_1846; Tsugaric acid A 2	neg_756; Tetracosanoylglycine
pos_1860; 11-Hydroxyandros	neg_757; Vitamin K2
pos_1871; 3-[(3-Methylbutyl)	neg_759; 3-trans-Caffeoyltormentic acid
pos_1904; beta-Doradecin	neg_763; 2,4-Dihydroxy-7,8-dimethoxy-2H-1,4-benzoxazin-3(4H)-one 2-glucoside
pos_1908; Hydroxyclomipran	neg_773; 3-Fucosyllactose
pos_1918; PS(18:0/18:2(9Z,1	neg_778; Physapubescin
pos_1920; Eicosapentaenoyl	neg_781; Norbutorphanol
pos_1922; Pteroside A	neg_79; 11(R)-HEDE
pos_1924; Hexyl benzoate	neg_790; Notoginsenoside T2
pos_1926; Hoduloside V	neg_796; Desglucocoroloside
pos_1931; 4-(3-Hydroxybuty	neg_8; trans-3-Coumaric acid
pos_1935; 3-O-Sulfogalactos	neg_800; 9,12-Hexadecadienoylcarnitine
pos_1941; Testolactone	neg_815; 2-Arachidonoylglycerophosphocholine
pos_1952; (3beta,24xi)-Cyclo	neg_821; Maltotriose
pos_1960; 23S,25,26-Trihydr	neg_824; (3b,6b,8b,12a)-8,12-Epoxy-7(11)-eremophilene-6-angeloyloxy-8,12-dimethoxy
pos_1964; 7-Hydroxygliclazi	neg_827; 3-Hexaprenyl-4-hydroxy-5-methoxybenzoic acid
pos_1969; Ganoderic acid C2	neg_830; Pentadecanoylglycine
pos_1980; Corchorusoside A	neg_834; 3-Hydroxynevirapine glucuronide
pos_1988; (25S)-26-Hydroxy	neg_838; Campesteryl caffeate
pos_2012; 1-(4-Hydroxy-3-m	neg_84; DIHYDROJASMONIC ACID
pos_2014; 3a,7a-Dihydroxyc	neg_862; 2-Amino-4-ethoxy-3-hydroxybutanoic acid
pos_2015; Phytolaccoside D	neg_87; 1-Dehydro-12-gingerdione
pos_2016; 13-HOTE	neg_90; D-Xylulose
pos_2022; Coutaric acid	neg_904; Prosapogenin
pos_2023; Furaneol 4-glucosi	neg_920; DG(15:0/18:4(6Z,9Z,12Z,15Z)/0:0)
pos_203; (S)-11,12,13-Trinoi	neg_938; L-Menthyl acetoacetate
pos_2031; PC(18:3(9Z,12Z,1	neg_939; N2-Succinoylarginine

pos_2032; 2,4-Diamino-6-nit	neg_942; Reticulataxanthin
pos_2042; Pyrraline	neg_944; Oleanolic acid 3-[glucosyl-(1->4)-xyloside]
pos_2048; RPR112698	neg_945; 3,7,11,15,23-Pentaoxolanost-8-en-26-oic acid
pos_2049; Grevilline B	neg_947; N-Arachidonoyl GABA
pos_2054; LysoPE(0:0/24:0)	neg_978; 13'-Hydroxy-alpha-tocotrienol
pos_2069; Polymyxin B Sulfa	neg_982; 3-Hydroxy-1-phenyl-1-eicosanone
pos_207; Cicutoxin	neg_985; (23S,24S)-17,23-Epoxy-24,29-dihydroxy-27-norlanost-8-ene-3,15-dione
pos_2072; 2-Hydroxymyristo	neg_997; (3beta,4alpha,5alpha,24S)-4,14-Dimethylstigmasta-8,25-dien-3-ol
pos_2078; Pipazethate	pos_10; Xanthurenic acid
pos_2083; 10,11-dihydro-20-	pos_1008; 6,8-Dihydroxypurine
pos_2096; Difluprednate	pos_1016; Dihydrozeatin-O-glucoside
pos_2098; Glucosylceramide	pos_1018; 6-Cinnamoyl-1-galloylglucose
pos_2100; Trypanothione dis	pos_1022; Kobusone
pos_2104; 24-Hydroxyglabro	pos_1026; Butyl 3-methylbutanoate
pos_2114; Carbamazepine	pos_1036; Acetaminophen cystein
pos_2119; Persenone B	pos_1044; 7a,12a-Dihydroxy-cholestene-3-one
pos_212; 3-Deoxyestrone	pos_1058; 3-Methyl-alpha-ionyl acetate
pos_2126; Beta-D-Glucopyra	pos_1059; MG(0:0/15:0/0:0)
pos_2137; 4,4-Dimethyl-14al	pos_106; PG(20:5(5Z,8Z,11Z,14Z,17Z)/0:0)
pos_2139; 11-beta-Hydroxya	pos_1061; 2(R)-hydroxyicosanoic acid
pos_215; delta2-THA	pos_1062; Almotriptan
pos_2158; DG(22:1n9/0:0/22	pos_1063; (1beta,2beta,5beta)-p-Menth-3-ene-1,2,5-triol
pos_2169; (1R,2R,4S)-p-Men	pos_1068; 2-Methylpropyl butanoate
pos_2170; (-)-Matairesinol 4'	pos_1069; Butyl propionate
pos_2172; CDP-DG(16:0/20:	pos_1071; 5-Octadecynoic acid
pos_2177; 2-Ethylsuberic aci	pos_1074; 10,20-Dihydroxyeicosanoic acid
pos_2193; Roxatidine acetate	pos_1076; 4-Hydroxyphenylpyruvic acid
pos_220; Val Val Val	pos_1079; Zerumbone oxide
pos_2208; Capsicum annuum	pos_1080; Dopamine quinone
pos_2214; PS(16:0/20:4(5Z,8	pos_1082; Phytosphingosine-1-P
pos_2218; Secoeremopetasito	pos_1086; (R)-3-Hydroxy-Octadecanoic acid
pos_2224; Cedryl acetate	pos_1089; Alkaloid AQC2
pos_2248; 2-Hydroxy-2,6,6-t	pos_1095; 3alpha-O-trans-Feruloyl-2alpha-hydroxy-12-ursen-28-oic acid
pos_2254; Mammea B/BC cy	pos_1096; CE(20:4(8Z,11Z,14Z,17Z))
pos_2255; 7Z,10Z-Hexadeca	pos_1100; 5'-Deoxy-5-fluorocytidine
pos_2260; 2,3-Undecanedion	pos_1107; 6,11-Dihydroxy-2,2-dimethylpyrano[3,2-c]xanthen-7(2H)-one
pos_2264; PC(22:4(7Z,10Z,1	pos_1108; L-Cysteine
pos_2270; PE(18:2(9Z,12Z)/	pos_1111; Lepidiumterpenoid
pos_2286; 3-Hydroxy-3-(3,4-	pos_1112; 2-Acetyl-4,5-dihydrothiazole
pos_2294; Citreoviridin C	pos_1115; Glyceryl 5-hydroxydecanoate
pos_2299; 2-Hexaprenyl-3-m	pos_1116; Santene hydrate
pos_2310; S-Adenosylhomoc	pos_1117; Yuzu lactone
pos_234; 2-Methyl-4-phenylb	pos_1125; 4,6-Dihydroxy-2-quinolinecarboxylic acid
pos_2354; Tridemorph	pos_1128; (S)-Bilobanone
pos_2369; Galabiosylceramic	pos_1131; 1-pentadecanoyl-glycero-3-phosphate
pos_2382; Cabergoline	pos_1143; N-Lauroylglycine
pos_2388; (all-E)-1,8,10-Hep	pos_1147; Lotaustralin
pos_2403; Leucyl-Serine	pos_1148; (-)-3-Cyanomethyl-3-hydroxy-1H-indol-2(3H)-one
pos_2407; Amaranthussaponi	pos_1149; 4-Hydroxybenzylamine
pos_2410; (2E,5E,12Z,15Z)-	pos_1150; TG(15:0/20:3(5Z,8Z,11Z)/20:4(5Z,8Z,11Z,14Z))
pos_2423; Ethyl 7-epi-12-hyd	pos_1152; DHBOA-Glc
pos_2427; CDP-DG(16:0/22:	pos_1153; 6'-Hydroxyenterolactone
pos_2429; 2-acetyl-1-alkyl-sn	pos_1157; 3-Methyl-3-buten-2-one
pos_2443; Gomphidic acid	pos_1159; (Z)-6-Tetradecene-1,3-diyne-5,8-diol
pos_2467; 3'-Hydroxy-4'-met	pos_1163; Tetranor 12-HETE
pos_2471; dIDP	pos_1164; 3-Hydroxy-2-(4-methylbenzoyl)-4H-1-benzopyran-4-one
pos_2473; Capsicoside C3	pos_118; 6-Methylpretetramide
pos_2478; Ricinine	pos_1183; 5-(3',4',5'-Trihydroxyphenyl)-gamma-valerolactone-3'-O-glucuronide

pos_2505; 3-Epinobilin	pos_1185; Dulciol D
pos_2508; Fluorometholone	pos_1186; DG(22:4(7Z,10Z,13Z,16Z)/16:1(9Z)/0:0)
pos_2519; Asparaginyl-Hydr	pos_119; 3-ketosphinganine
pos_2522; (5Z,8Z)-1,5,8-Hep	pos_1190; Cerebronic acid
pos_2536; Terretonin	pos_1191; Floribundine
pos_2539; Trandolapril-d5 D	pos_1193; Bovolide
pos_2541; 2'-Oxoaloesol 7-gl	pos_1196; Kanzonol N
pos_2543; Ganodermic acid I	pos_1198; Camelliagenin B
pos_2560; 3-Oxo-alpha-ionol	pos_1199; Oxysolavetivone
pos_2562; Physagulin G	pos_12; Bis(2-ethylhexyl) phthalate
pos_2563; (3b,5a,25R)-3-Hy	pos_120; Zerumbone
pos_2568; PC(14:0/22:5(7Z,	pos_1204; (2R,3R,4R)-2-Amino-4-hydroxy-3-methylpentanoic acid
pos_2572; Melilotussaponin	pos_121; p-CHLOROPHENYLALANINE
pos_2573; Lucidumol A	pos_1219; N-methyl-L-glutamic Acid
pos_2578; Hydroxychloroqui	pos_122; R1128B
pos_2581; Enoxacin	pos_1232; hydroxytorsemide
pos_2598; L-Histidinol	pos_1234; Portensterol
pos_2599; 1,1'-Ethylidenebis	pos_1237; Taraxinic acid glucosyl ester
pos_2600; 3-cis-Hydroxy-b,e	pos_1239; 5,8-Epoxy-5,8-dihydro-10'-apo-b,y-carotene-3,10'-diol
pos_2602; 1,20-Eicosanediol	pos_1251; 3beta-3-Hydroxy-18-lupen-21-one
pos_2614; PIP2(18:3(9Z,12Z	pos_1252; Anileridine
pos_2617; 4-Methyl-1-pheny	pos_1257; Lenalidomide
pos_2628; Pseudoginsenosid	pos_126; 24-Dehydroprovitamin D3
pos_2629; Gravelliferone	pos_1263; Lysyl-Lysine
pos_2630; Momordicoside E	pos_1266; PC(18:4(6Z,9Z,12Z,15Z)/20:3(5Z,8Z,11Z))
pos_264; Neocembrene	pos_1277; Feruloyl-beta-sitosterol
pos_2648; Coniferan	pos_1279; (17alpha,23S)-Epoxy-28,29-dihydroxy-27-norlanost-8-ene-3,24-dione
pos_2653; Trigoneoside Xb	pos_1282; Ethylsuberenol
pos_2662; (1S,2R,4R,8S)-p-M	pos_1283; D-Pantothenoyl-L-cysteine
pos_2663; N-Nitrosoproline	pos_1289; (S)-10,16-Dihydroxyhexadecanoic acid
pos_2668; Maslinic acid 3-O	pos_1298; 1-Stearoylglycerophosphoserine
pos_2676; Digoxigenin bisdi	pos_1300; alpha-Tocopherolquinone
pos_2678; 3-O-Sulfogalactos	pos_1313; 5,10-Methylene-THF
pos_2703; 8-Hydroxypinores	pos_1316; Pentazocine
pos_271; Erinacine B	pos_1317; 5,6-epoxy,18R-HEPE
pos_2720; Balanitoside	pos_1322; (R)-Pterosin B
pos_2723; 6,7-Dihydro-7-hyc	pos_1324; Phytosphingosine
pos_2725; Trihydroxycopros	pos_1326; Momorcharaside A
pos_2728; Pregeijerene	pos_1327; Oleuroside
pos_2731; Bethanechol	pos_1336; 6-Hydroxy-1H-indole-3-acetamide
pos_2732; Ethanolamine	pos_1338; Propyl cinnamate
pos_2736; Cortol	pos_1339; Thiamine(1+) Diphosphate(1-)
pos_2761; Crustecdysone	pos_1341; PI(16:0/16:0)
pos_277; Demethoxycurcumi	pos_1346; (E)-4-[5-(4-Hydroxyphenoxy)-3-penten-1-ynyl]phenol
pos_2773; Gingerglycolipid	pos_1356; Ketotifen-N-glucuronide
pos_2775; DG(14:0/0:0/18:2	pos_1361; Isofucosterol glucoside
pos_2776; (3b,16a,21b,22a)-	pos_1362; Dihydrojasmonic acid
pos_2779; PE(DiMe(11,5)/D	pos_1378; Vanillic acid-4-O-glucuronide
pos_278; Maculine	pos_1379; Methyl 3,4-dihydroxy-5-prenylbenzoate 3-glucoside
pos_2799; Minaprine	pos_1381; Lacto-N-hexaose
pos_2819; Gibberellin A62	pos_1383; 4-Hydroxyproline
pos_2840; Trihexosylceramic	pos_1384; 7a-Hydroxytestosterone
pos_2886; N-(2,14-Eicosadie	pos_1391; PGP(18:0/22:4(7Z,10Z,13Z,16Z))
pos_2888; Alanyl-Serine	pos_1392; Tranexamic Acid
pos_2894; Dehydroaporheine	pos_1393; Glucosamine 6-phosphate
pos_2916; 11beta,20-Dihydr	pos_1395; Vanillin
pos_2932; Cerebroside B	pos_1398; Pteroside B
pos_2938; 1-Methylhistidine	pos_1399; Methyl 5-hydroxyoxindole-3-acetate

pos_294; Rhubafuran	pos_1407; Melleolide H
pos_2945; Nomilinic acid 17-	pos_1415; o-Tyrosine
pos_2946; (S)-[8]-Gingerol	pos_1420; Colupulone
pos_2954; 12-Hydroxy-13-O-	pos_1429; Kiwiionoside
pos_2955; Tuberoside A (All	pos_1430; Docosanamide
pos_2967; Tragopogonsaponi	pos_1438; Stigmasta-4,6-dien-3-one
pos_297; 1伪,25-dihydroxy-3	pos_144; 26,26,26-trifluoro-25-hydroxyvitamin D3 / 26,26,26-trifluoro-25-hydroxycholec
pos_2987; PE(15:0/20:1(11Z	pos_1440; MG(20:1(11Z)/0:0/0:0)
pos_2990; Molybdopterin pre	pos_1449; Pivampicillin
pos_30; rac-Glycerol 1-myris	pos_1450; 1,2,3,4,5,6-Hexahydro-5-(1-hydroxyethylidene)-7H-cyclopenta[b]pyridin-7-on
pos_3035; Methyl Arachidon	pos_1461; Furazolidone
pos_305; 1-H-Inden-1-one,2,	pos_1467; N,N-Dimethylsphingosine
pos_3050; N-Acetyl-L-pheny	pos_1470; Cinitapride
pos_3051; Diadenosine dipho	pos_1472; Margaroylglycine
pos_3055; TG(14:0/22:4(7Z,	pos_1475; Galabiosylceramide (d18:1/25:0)
pos_3064; Tertatolol	pos_1478; Petroselinic acid
pos_3070; PS(20:4(5Z,8Z,11	pos_1486; Propylene glycol alginate
pos_3089; (3b,9R)-5-Megasti	pos_1494; 6-Hydroxydopamine
pos_3108; PC(20:5(5Z,8Z,11	pos_1501; 2,4-Di-tert-butylphenol
pos_3109; Capryloylcholine	pos_1505; Valechlorin
pos_315; ANETHOLE	pos_1506; Alpha-Linolenoyl ethanolamide
pos_3163; 6,11-Dihydroxy-3-	pos_1510; Arjunolic acid
pos_3164; Austdiol	pos_1512; L-argininium(1+)
pos_3165; Melilotin	pos_1516; 3-(Acetyloxy)-2-hydroxypropyl icosanoate
pos_3167; 8-Nonen-2-one	pos_1519; 23-Hydroxy-3-oxocycloart-24-en-26-oic acid
pos_3182; PS(DiMe(9,3)/DiM	pos_1533; Acarbose
pos_3187; Hoduloside VIII	pos_1535; 5-Hydroxytryptophol glucuronide
pos_3193; (S)-4',5,7-Trihydro	pos_1543; (Z)-N-Feruloyl-5-hydroxyanthranilic acid
pos_3202; M-Secociguatoxin	pos_1545; Irisolidone 7-O-glucuronide
pos_3203; (3x,5x,10x)-9,10-I	pos_1546; Streptomycin
pos_3204; gamma-Chaconine	pos_1547; 12-Oxo-2,3-dinor-10,15-phytodienoic acid
pos_3207; N-Cyclopropyl-tra	pos_1549; N-Stearoyl GABA
pos_3224; Isepamicin	pos_1579; D-1-Amino-2-pyrrolidinecarboxylic acid
pos_323; Farfugin A	pos_1584; TG(15:0/18:1(11Z)/18:4(6Z,9Z,12Z,15Z))
pos_3241; 18-Deoxysagittaro	pos_1591; Aesculin
pos_3245; DG(15:0/18:0/0:0)	pos_1597; (1S,2S,4R,8S)-p-Menthane-1,2,8,9-tetrol 2-glucoside
pos_3249; Xylometazoline	pos_1611; Valganciclovir
pos_3259; Tryptophyl-Trypto	pos_1616; Stearoylglycine
pos_3272; 4'-N-desmethylola	pos_1618; 3-Methyluric acid
pos_3281; Quassimarin	pos_163; 11-Deoxocucurbitacin I
pos_3295; 11-Hydroxyproges	pos_1630; Demethylphylloquinone
pos_3301; DHAP(10:0)	pos_1634; Octyl 4-methoxycinnamic acid
pos_3304; Phytolaccoside A	pos_1641; erythro-7,9-Hexatriacontanediol
pos_3312; Oleoside 11-methy	pos_1642; Fludrocortisone
pos_3323; Tricosanoylglycine	pos_1643; Homoferreirin
pos_3331; 3-O-Sulfogalactosy	pos_1644; Pyridinoline
pos_3338; Hebevinoside II	pos_1646; 13'-Hydroxy-gamma-tocotrienol
pos_334; 10,13,16-Docosatri	pos_1650; Cinnamic acid
pos_335; 13-Oxo-9,11-tridec	pos_1651; Benzaldehyde
pos_3365; Neosilyhermin A	pos_1658; DG(16:0/22:6(4Z,7Z,10Z,13Z,16Z,19Z)/0:0)
pos_341; Ethyl (2E,4Z,7Z)-D	pos_1660; Mabioside D
pos_344; 7-Ethyl-3,11-dimeth	pos_1664; Ginsenoyne J
pos_3441; PC(22:2(13Z,16Z)	pos_167; Ganoderic acid beta
pos_3451; PE(15:0/22:1(13Z	pos_1671; (3S,3'R,4xi)-beta,beta-Carotene-3,3',4-triol
pos_3474; Emtricitabine	pos_1680; 1-(2-Furanyl)-2-propanone
pos_3485; Myricatomentosid	pos_1684; Octahydro-2H-1-benzopyran-2-one
pos_350; 7-Ethyl-3,11-dimeth	pos_1687; 24,25-Dihydroxyvitamin D
pos_3514; 54-Deoxyciguatox	pos_1689; Fencamfamine

pos_3519; Sativoside R2	pos_1694; Oxytocin
pos_354; TrHA	pos_1695; Milrinone
pos_3542; Aspartylglycosami	pos_1699; 3-Hydroxy-4-butanolide
pos_3545; TG(22:5(4Z,7Z,1(	pos_170; 7,8-Dehydro-3,4-dihydro-beta-ionol
pos_3546; Hoduloside X	pos_1702; Estradiol
pos_355; Ala Thr Tyr His	pos_1704; (3beta,17alpha,23S,24S)-17,23-Epoxy-3,24,29-trihydroxy-27-norlanost-8-en-1
pos_3566; PE(14:0/22:2(13Z	pos_1710; Lycopersiconolide
pos_3568; DG(16:0/22:1(13Z	pos_1715; 5-(3',4'-Dihydroxyphenyl)-gamma-valerolactone
pos_3574; Aegle marmelos A	pos_1716; 5-Hydroxy-L-tryptophan
pos_3576; Tsugaric acid A	pos_1738; Mulberranol
pos_3596; Docetaxel	pos_174; 1,25-Dihydroxy-24-oxo-16-ene-vitamin D3
pos_3600; Glucosylceramide	pos_1743; Ginsenoyne N
pos_3611; 1-(9Z-tetradecenoy	pos_1744; 1,2-Di-(9Z,12Z-octadecadienoyl)-sn-glycero-3-phosphate
pos_3613; PS(DiMe(13,5)/M	pos_1748; 1-Hydroxy-3-methoxy-7-primeverosyloxyxanthone
pos_3625; DG(22:6(4Z,7Z,1(	pos_1760; Oryzalide A
pos_364; (Z)-2-hexacos-17-e	pos_1767; (-)-beta-Elemene
pos_3640; 3'-p-Hydroxypacli	pos_1773; 3-Hydroxyanthranilic acid
pos_3645; Oleamide	pos_1774; 4-Hydroxybenzaldehyde
pos_3653; Octyl 2-furoate	pos_1776; Ginsenoside Rh5
pos_366; Peroxysimulenoline	pos_1780; Ac-Ser-Asp-Lys-Pro-OH
pos_3660; Epsilon-caprolacta	pos_1781; Octadecadienoate
pos_3661; 4-(Glutamylamino	pos_1787; Polyporusterone B
pos_3666; 7-a,25-Dihydroxy	pos_1788; 1-Heptadecanoylglycerophosphoethanolamine
pos_3671; DG(16:0e/18:0/0:(	pos_1791; Salsoline-1-carboxylate
pos_3674; Sucrose acetate iso	pos_1795; SM(d18:1/12:0)
pos_3695; DG(14:1(9Z)/22:1	pos_1806; 2-oxoglutarate(2-)
pos_3696; Oleanolic acid 3-[	pos_1811; MG(0:0/14:0/0:0)
pos_3697; PG(16:1(9Z)/20:4	pos_1818; Linatine
pos_3705; Notoginsenoside F	pos_1823; Bisbynin
pos_3707; Cyclotricuspidoge	pos_1826; Ceanothenic acid
pos_3715; Resolvin D5	pos_1828; beta-Citraurol
pos_3717; Methyl 3,4,5-trime	pos_1831; 27-Norcholestanehexol
pos_3720; Alpha-Tocotrienol	pos_1833; Theaflagallin
pos_3723; (+)-15,16-Dihydro	pos_1834; Butyric acid
pos_3741; 2-Hexaprenyl-6-m	pos_184; Cynaroside A
pos_3767; Valyl-Glutamate	pos_1840; 1b-Furanoeudesm-4(15)-en-1-ol acetate
pos_3773; Dynorphin A 9-17	pos_1845; (R)-1-O-[b-D-Glucopyranosyl-(1->6)-b-D-glucopyranoside]-1,3-octanediol
pos_3778; 3,6,10-Trimethylte	pos_1848; Azaspiracid 3
pos_379; 6-Valerene-11-ol	pos_1855; 3-O-alpha-D-Glucopyranuronosyl-D-xylose
pos_3816; Dihydromelilotosi	pos_1868; Neferine
pos_3828; Demethylcalabaxa	pos_1884; Primidone
pos_3848; Rubraflavone B	pos_1890; L-Homoserine
pos_3868; PE(18:3(6Z,9Z,12	pos_1892; N-Stearoyl phenylalanine
pos_3885; Serotinose	pos_1893; Norketamine
pos_3905; Zingiberenol	pos_1897; Galactosylceramide (d18:1/14:0)
pos_3906; DG(14:1(9Z)/20:0	pos_1906; Blasticidin S
pos_3914; Melilotoside C	pos_1907; 2-Propionylpyrrole
pos_3932; PG(16:0/18:2(9Z,	pos_1910; 2-phosphonato-D-glycerate(3-)
pos_3939; (9S,10S)-9,10-dih	pos_1913; DG(18:1(11Z)/14:1(9Z)/0:0)
pos_3983; 10-Undecenal	pos_1914; PC(22:1(13Z)/22:5(4Z,7Z,10Z,13Z,16Z))
pos_3993; Ginsenoside III	pos_1927; Cyclohexanecarboxylic acid
pos_400; Constanolactone E	pos_1928; gamma-Tocopheryl quinone
pos_4000; Diosbulbin H	pos_1929; Asparaginyl-Proline
pos_401; 5alpha-androst-16-e	pos_193; 25-hydroxy-16,17,23,24-tetradehydrovitamin D3 / 25-hydroxy-16,17,23,24-tetra
pos_4035; 1-O-Sinapoylgluc	pos_1932; xi-2,3-Dihydro-2-oxo-1H-indole-3-acetic acid
pos_4041; Phytyl diphosphat	pos_1936; Amasterol
pos_4046; PIP2(18:2(9Z,12Z	pos_1949; Matesaponin 3
pos_4048; Mulberrofuran T	pos_1953; 2-Hexyl-4,5-dimethyl-1,3-dioxolane

pos_4049; xi-2,2,6-Trimethyl	pos_1956; 8,11-Heptadecadienal
pos_4064; Dezocine	pos_1957; Cinnzeylanine
pos_407; diethyltoluamide	pos_196; 20(17+mm12尾)-abeo-1伪,25-Dihydroxy-24-dihomo-21-norvitamin D3
pos_4081; 2-(4-Methyl-5-thia	pos_1962; 4a-Carboxy-4b-methyl-5a-cholesta-8,24-dien-3b-ol
pos_4087; Glucoconringiin	pos_197; 4-Hydroxy-2-quinolone
pos_4092; Brassicanal C	pos_1977; 2-(2-Thienylmethylene)-1,6-dioxaspiro[4.4]non-3-ene
pos_4103; DG(18:3(6Z,9Z,1	pos_1979; beta-D-Glucosyloxydestruxin B
pos_4112; Quinquenoside IV	pos_1984; PE(20:4(5Z,8Z,11Z,14Z)/P-16:0)
pos_4113; PE(18:2(9Z,12Z)/	pos_1986; Methylthio 2-(propanoyloxy)propanoate
pos_4114; 3-alpha-Androstan	pos_1991; Artenolide
pos_413; 3-(3,4-Dihydroxypl	pos_1992; N-Acetylaminooctanoic acid
pos_4136; Kuwanon F	pos_1994; N-di-Demethyl roxithromycin
pos_4137; Sambubiose	pos_2001; PE(14:0/P-16:0)
pos_4138; Stevioside	pos_2017; Ubiquinone-4
pos_414; 5alpha-Gonane	pos_2019; Treprostinil
pos_4144; N-Acetylneuramin	pos_2026; (2alpha,3alpha,5alpha,22R,23R)-2,3,22,23-Tetrahydroxy-25-methylergost-24(
pos_416; Docosahexaenoic A	pos_2027; Armexifolin
pos_4161; 4,14-Dimethylerg	pos_2033; PE(14:1(9Z)/20:5(5Z,8Z,11Z,14Z,17Z))
pos_4164; 2-Methoxy-estradi	pos_2036; 4-[2,2'-Bithiophen-5-yl]-3-butyn-1-ol
pos_4169; Pomonic acid	pos_2039; LysoSM(d18:0)
pos_4172; Ganoderic acid C1	pos_2053; DG(14:0/14:0/0:0)
pos_4176; 5-Ethynyl-5'-(1-pr	pos_2056; Annomutacin
pos_4190; cis-3-Hexenyl tigl	pos_2066; Alitame
pos_4192; PC(16:0/24:0)	pos_2074; MG(0:0/22:0/0:0)
pos_4220; Jubanine A	pos_2077; (S)-Homostachydrine
pos_4239; Lucyoside R	pos_208; Longicamphenylone
pos_4255; Galactonic acid	pos_2092; Deoxynivalenol
pos_4284; PIP(18:2(9Z,12Z)	pos_2093; 4-Hydroxytamoxifen-O-glucuronide
pos_4301; PE(DiMe(13,5)/D	pos_2099; DG(18:2(9Z,12Z)/18:3(9Z,12Z,15Z)/0:0)
pos_4305; erythro-6,8-Tricos	pos_2108; alpha-Terpineol butanoate
pos_4319; PC(DiMe(9,3)/Mc	pos_2111; 1-(3,5-Dihydroxyphenyl)-2-pentadecanone
pos_4331; Gonyautoxin VI	pos_2113; 5-(2-Hydroxyethyl)-4-methylthiazole acetate
pos_4335; Pefloxacin N-oxid	pos_2121; 6-Deoxodolichosterone
pos_434; alatolide	pos_2140; SM(d17:1/24:1(15Z))
pos_4358; alpha-Linolenic ac	pos_2153; Dimethyl 2-galloylgalactarate
pos_436; 3-Deoxy-25-hydrox	pos_2157; LysoSM(d18:1)
pos_4362; Stearidonic Acid	pos_2163; Glycylproline
pos_4373; Eicosapentaenoic	pos_2164; Norepinephrine
pos_4382; C-8 Ceramide	pos_2168; Avocadyne 2-acetate
pos_4386; Epitestosterone	pos_2173; Linalyl propionate
pos_4393; 25-hydroxyvitami	pos_2186; 2-Pyrroloylglycine
pos_4411; Cholecalciferol (V	pos_219; Glycerylphosphorylethanolamine
pos_4429; cis-9-Palmitoleic	pos_2194; N-arachidonoylglycinate
pos_447; (+)-alpha-Longipin	pos_2197; 13'-Hydroxy-gamma-tocopherol
pos_45; cholesta-5,22E-dien-	pos_2200; 13'-Carboxy-gamma-tocotrienol
pos_450; 2-Phenylethanol	pos_2204; Latanoprost
pos_458; toxisterol3 E1	pos_2207; DG(14:1n5/0:0/18:3n6)
pos_495; 5a-androstane	pos_2216; 13'-Carboxy-alpha-tocopherol
pos_498; 2-Butyl-3-phenyl-2	pos_222; DG(16:1(9Z)/22:5(7Z,10Z,13Z,16Z,19Z)/0:0)[iso2]
pos_500; (20S,22E)-3尾-Hyd	pos_2228; Lamivudine sulfoxide
pos_502; toxisterol3 D1	pos_223; Ganoderic acid delta
pos_503; Lathosterol	pos_2233; [10]-Gingerdione
pos_506; 2-hexadecenal	pos_2239; (22Alpha)-hydroxy-5alpha-campestan-3-one
pos_510; Cholesterol	pos_2241; Heptadecanoyl carnitine
pos_512; 17-Methyl-18-nora	pos_2256; PC(o-22:0/22:6(4Z,7Z,10Z,13Z,16Z,19Z))
pos_524; 2,4,6-Heptatrienoic	pos_2267; 20-Hydroxy-leukotriene E4
pos_528; 11H-Benz[bc]acear	pos_2277; Pandamarilactam 3x
pos_532; 1-Naphthol	pos_2281; 5-[2H-Pyrrol-4-(3H)-ylidenemethyl]-2-furanmethanol

pos_547; Methylripariochron	pos_2287; Taurocholic acid 3-sulfate
pos_551; Val Phe Arg Val	pos_2289; 3-Acetyl-2,5-dimethylthiophene
pos_552; Gly Ile Met Ala	pos_2292; Licoricone
pos_553; methyl 9-butylpero:	pos_2300; Tragopogonsaponin A
pos_558; 3-Gymnomitrene	pos_2303; N-Linoleoyl GABA
pos_573; 3-Isopropyl-2-meth	pos_2312; Kaempferol 3-sophorotrioside
pos_594; PS(12:0/19:0)	pos_2328; PE(24:0/P-18:0)
pos_597; ent-7alpha-hydroxy	pos_233; JWH 302
pos_605; 5beta-pregnane-3,2	pos_2335; Acidissiminol epoxide
pos_619; Ala Leu	pos_2336; Isovalerylcarnitine
pos_641; Ser His Cys Arg	pos_2339; (3beta,22E,24R)-3-Hydroxyergosta-5,8,22-trien-7-one
pos_649; 11-Hydroxycanthin	pos_2340; 4,4-Dimethylcholesta-8(9),14-dien-3beta-ol
pos_655; PE(16:0/22:5(4Z,7Z	pos_2344; Zeranol
pos_656; Irehine	pos_2353; Betavulgaroside I
pos_673; PI(22:5(4Z,7Z,10Z,	pos_237; (3S,6E)-6-Caryophyllen-15-al
pos_682; NERYL ACETATE	pos_2374; (6R,7S)-6,7-Epoxy-1,3-tetradecadiyne
pos_692; Coriandrone E	pos_2375; PC(20:3(5Z,8Z,11Z)/20:4(5Z,8Z,11Z,14Z))
pos_697; MG(20:3(5Z,8Z,11	pos_2378; Galbanic acid
pos_7; Lumichrome	pos_2385; 4-Nonylphenol
pos_70; Sphingosine	pos_2392; 1-O-all-trans-retinoyl-beta-glucuronic Acid
pos_701; PS(17:0/18:4(6Z,9Z	pos_2399; Herniarin
pos_706; PE(15:0/15:1(9Z))	pos_240; 2,5-Diisopropyl-4-methylphenol
pos_713; 2,4,6-undecatrienal	pos_2400; keratan sulfate I
pos_724; Triazamate	pos_2405; omega-hydroxyfinasteride
pos_727; Leu Phe Trp	pos_2408; Dehydrophytosphingosine
pos_73; 7-Ethyl-3,6-dihydro-	pos_2411; 9'-Carboxy-gamma-chromanol
pos_732; Ganolucidic acid B	pos_2418; N2-Succinyl-L-glutamic acid 5-semialdehyde
pos_739; Erogorgiaene	pos_2424; (24E)-3alpha,15alpha-Diacetoxy-23-oxo-7,9(11),24-lanostatrien-26-oic acid
pos_755; Selina-6-en-4-ol	pos_2433; PE(16:0/20:3(5Z,8Z,11Z))
pos_78; (S)-gamma-Calacore	pos_2450; Carnosol
pos_781; 5-(2-hydroxyethyl)i	pos_2451; Lithocholate 3-O-glucuronide
pos_785; Ile Ile Leu	pos_2453; 1-(3-Methylbutanoyl)-6-apiosylglucose
pos_787; Senkirkine	pos_2463; Oxypinnatanine
pos_793; (6R)-6,19-epidioxy-	pos_248; 4-(4-Methyl-3-pentenyl)-3-cyclohexene-1-carboxaldehyde
pos_797; 4-(Methylthio)-1-bu	pos_2480; Juzirine
pos_803; Ala His Gln Thr	pos_2492; 28-Hydroxymangiferonic acid
pos_806; 2-(2-Methylpropoxy	pos_2493; Epimetendiol
pos_816; Lysyl-Isoleucine	pos_2496; Congmunoside XV
pos_819; Pro Val Ser Leu	pos_2498; Dronabinol
pos_834; 3-Oxo-delta4-steroi	pos_2499; Mizoribine
pos_838; 5a-Androst-3-en-17	pos_2501; Melibiose
pos_841; Ala Val	pos_2504; Aucubin
pos_846; Vitispirane	pos_2507; Sarmentosin
pos_85; 12伪-Hydroxy-3-oxc	pos_251; Galactose-beta-1,4-xylose
pos_853; Dodecanoic acid	pos_2513; 4,5-Dihydroorotic acid
pos_861; Tolterodine	pos_2514; Avenalumic acid
pos_864; 15-Hexadecanolide	pos_2515; Norrubrofusarin 6-beta-gentiobioside
pos_866; Citronellyl cinnama	pos_2526; 5beta-Coprostanol
pos_873; N-Decanoylglycine	pos_2564; Megestrol
pos_886; 3a,7a-Dihydroxy-5b	pos_2585; Lacto-N-difucopentaose II
pos_894; Tetrahydro-6-(2-hy	pos_2594; DG(14:1(9Z)/20:4(5Z,8Z,11Z,14Z)/0:0)
pos_898; 4,4alpha,5,6-Tetrah	pos_2597; Pitheduloside B
pos_90; L-Methionine	pos_2605; para-hydroxyrosiglitazone
pos_903; Pseudomonine	pos_2612; 3-phosphonato-D-glyceroyl Phosphate(4-)
pos_92; N-(3-Indolylacetyl)-I	pos_2613; triazolopropionic acid
pos_925; Propyl hexanoate	pos_2638; 2-Keto-6-acetamidocaproate
pos_933; N,N,O-Tridesmethy	pos_2649; Malonoben
pos_934; (R)-lipoic acid	pos_265; (6E,8E)-4,6,8-Megastigmatriene

pos_936; 4-Ethyl-2-methyl-5-	pos_2652; Myricanol 5-laminaribioside
pos_95; xi-3-(4-Isopropylphe	pos_2664; Oleacein
pos_956; Pipereicosalidine	pos_2665; Glucosinalbin
pos_958; Propiverine	pos_2675; 7a-Hydroxycholesterol
pos_962; (all-Z)-8,11,14-Hep	pos_268; (E)-Suberenol
pos_965; 5-Aminopentanal	pos_2683; PC(DiMe(11,3)/MonoMe(11,3))
pos_977; MG(0:0/24:6(6Z,9Z	pos_2690; Bisacurone epoxide
pos_978; Tetracosapentaenoi	pos_2696; 3-(2-Furanylmethyl)-1H-pyrrole
pos_982; Obtusilactone A	pos_2713; Lucidenic acid B
pos_991; Austroinulin	pos_274; 1-(1-Pyrrolidinyl)-2-propanone
	pos_2765; Phenylbutyrylglutamine
	pos_2774; (-)-Quebrachamine
	pos_2780; Ceanothine B
	pos_2784; (R)-2-Hydroxysterculic acid
	pos_2791; DG(16:1(9Z)/22:2(13Z,16Z)/0:0)
	pos_2794; O-Desmethylnaproxen
	pos_2796; Zymosterol intermediate 2
	pos_2800; Shoyuflavone B
	pos_2803; trans-2-Dodecenoylcarnitine
	pos_2814; N-desmethylrosiglitazone
	pos_2816; 11,12,13-Trinor-1(10)-spirovetivene-2,7-dione
	pos_282; 4E,6Z-Hexadecadienyl acetate
	pos_2823; 3beta-3-Hydroxy-11-oxolanosta-8,24-dien-26-al
	pos_2825; DG(18:4(6Z,9Z,12Z,15Z)/22:6(4Z,7Z,10Z,13Z,16Z,19Z)/0:0)
	pos_2828; 3-Nonyl-1H-pyrazole
	pos_2829; (Z)-6-(2-Methoxyvinyl)-7-methyl-2H-1-benzopyran-2-one
	pos_2836; PE(15:0/P-16:0)
	pos_2847; lysoPC(26:1(5Z))
	pos_2849; Chinenoside II
	pos_285; Nitrosylsulfuric acid
	pos_2854; alpha-Crocetin glucosyl ester
	pos_2867; Cholest-5-ene
	pos_2870; Corchoroside A
	pos_2872; 4-Hydroxydiphenylamine
	pos_2887; Maslinic acid
	pos_289; N1,N10-Dicoumaroylspermidine
	pos_2898; Ganoderic acid W
	pos_290; Eremopetasinorol
	pos_2903; Citranaxanthin
	pos_2919; Glutamyl-Tryptophan
	pos_292; 3,4-Dihydroxybenzaldehyde
	pos_2921; (2S,3S,4S)-5,7,9,11-Tridecatetrayne-1,2,3,4-tetrol
	pos_2925; MG(20:2(11Z,14Z)/0:0/0:0)
	pos_2944; Glutamyl-Arginine
	pos_2947; Lacinilene C
	pos_2950; Ganoderic acid J
	pos_2951; Erucic acid
	pos_2952; 2'-Apo-beta-carotenal
	pos_2959; Oltipraz
	pos_2964; Oxalic acid
	pos_2966; arabinofuranosylguanine
	pos_2979; 1,9-Nonanedithiol
	pos_2984; N5-(3,4-Dioxo-1,5-cyclohexadien-1-yl)-L-glutamine
	pos_2989; L-Proline
	pos_3001; (3b,16a,20R)-25-Acetoxy-3,16,20,22-tetrahydroxy-5-cucurbiten-11-one 3-gluc
	pos_3003; Foeniculoside VIII
	pos_3004; trans-isoeugenol-O-glucuronide
	pos_3011; Ethyladipic acid

pos_3014; Pentadecanal
pos_3015; 2,4-Heptadecanedione
pos_3016; 3-Hydroxy-9Z-octadecenoylcarnitine
pos_3023; Hypoglycin B
pos_3026; 4-(2-Furanylmethylene)-3,4-dihydro-2H-pyrrole
pos_3029; (S)-3-Butyl-1(3H)-isobenzofuranone
pos_3030; 3a,7a,12a-Trihydroxy-5b-cholest-24-enoyl-CoA
pos_3032; Avocadene 1-acetate
pos_3034; Ganglioside GM3 (d18:1/18:1(11Z))
pos_3037; Glucosyl 6-hydroxy-2,6-dimethyl-2E,7-octadienoate
pos_3062; Tenuazonic acid
pos_3065; alpha-Carissanol
pos_3071; 1,21-Heneicosanediol
pos_3074; Okadaic acid
pos_3075; Isocolumbin
pos_3085; Smilagenin 3-[2''-glucosyl-6''-arabinosylglucoside]
pos_3087; 3-Hydroxy-5, 8-tetradecadiencarnitine
pos_3093; 2,4-Difurfurylfuran
pos_3096; (卤)-2,2'-Iminobispropanoic acid
pos_3099; 1-nonadecanoyl-glycero-3-phosphate
pos_3106; Dihydroxyacetone Phosphate Acyl Ester
pos_3107; Nonate
pos_311; Cer(d18:0/16:0)
pos_3110; Muricin G
pos_3111; PE(24:1(15Z)/24:1(15Z))
pos_3114; (2E,4E,6Z)-2,4,6-Decatrienoic acid dehydropiperidide
pos_3115; Nandrolone
pos_3118; 5S,6S-epoxy-15R-hydroxy-ETE
pos_3119; Ganglioside GM3 (d18:1/16:0)
pos_3132; Triethanolamine
pos_3133; Methylergonovine
pos_3134; Glyyunnansapogenin B
pos_314; 1,4,5-Naphthalenetriol
pos_3153; 3 alpha,7 alpha,26-Trihydroxy-5beta-cholestane
pos_3154; Momordol
pos_3157; Pyranomammea C
pos_3159; Betavulgaroside VII
pos_3162; 8-Methoxykynurenate
pos_3166; alpha-Solamarine
pos_3181; (1xi,4xi,6xi)-Carvone oxide
pos_3184; N-Dodecane
pos_3201; Secoisolariciresinol
pos_3206; 5'-O-Methylmelledonal
pos_3211; PE(24:0/22:1(13Z))
pos_322; Benzyl trans-2-methyl-2-butenoate
pos_3227; Momordicoside D
pos_3228; Linalyl isobutyrate
pos_3229; 5-Fluorouridine monophosphate
pos_324; Ile Val Leu Ile
pos_3243; lysoPC(28:0)
pos_3248; PE(14:0/14:1(9Z))
pos_3266; Dapiprazole
pos_3269; Tetracosanoic acid
pos_327; Oleanolic aldehyde
pos_3280; 2,2-dichloro-1,1-ethanediol
pos_3284; (卤)-Aegeline
pos_3286; 3-Mercapto-2-butanol
pos_3294; 20-Oxo-leukotriene E4

pos_3297; CerP(d18:1/12:0)
pos_330; Val Leu
pos_3306; Debenzoylzucchini factor B
pos_3311; Cysteinyl-Lysine
pos_3319; (3beta,11alpha,13beta)-3,11,13-Oleananetriol
pos_3322; Hericene C
pos_333; Adynerin
pos_3336; alpha-Terpinyl pentanoate
pos_3349; Astin I
pos_3355; PG(16:0/20:3(5Z,8Z,11Z))
pos_3356; Ganglioside GM3 (d18:0/14:0)
pos_3357; (Z)-8-Tetradecenal
pos_3362; Tryptophyl-Threonine
pos_3363; TG(8:0/8:0/15:0)
pos_3369; Nigroxanthin
pos_3372; Melongoside G
pos_3373; Methylgingerol
pos_3380; Erythrose
pos_3394; 3-Hydroxytamoxifen (Droloxifene)
pos_3395; TG(14:1(9Z)/14:1(9Z)/14:1(9Z))
pos_3399; NPC
pos_3413; N-Acetyl-6-O-L-fucosyl-D-glucosamine
pos_342; Soyasaponin I
pos_3423; Cibaric acid
pos_3436; 4-Hydroxy-3-prenylbenzoic acid glucoside
pos_3438; alpha-Chaconine
pos_345; LG 100268
pos_3450; Farnesiferol A
pos_347; Anofinic acid
pos_3478; Perflutren
pos_3498; 7a,17-dimethyl-5b-Androstane-3a,17b-diol
pos_35; LysoPE(15:0/0:0)
pos_3502; Nicotine imine
pos_3513; Ornithokinin
pos_3516; Methionine sulfoximine
pos_352; 2-(1-Pyrrolidinyl)-3-pentanone
pos_3537; Glutaminyl-Tryptophan
pos_3540; 1,7-Dimethylguanosine
pos_3552; Periandrin V
pos_3559; lysoPC(6:0)
pos_3560; Dodecyl butyrate
pos_3564; HMBOA-Glc
pos_3567; 13'-Carboxy-alpha-tocotrienol
pos_357; Arachidonic Acid-biotin
pos_3573; Ginsenoyne M
pos_3578; 1-Ethenylhexyl butanoate
pos_3581; N1-trans-Feruloylagmatine
pos_3583; Tetrahydrodeoxycorticosterone
pos_3591; Sonchifolin
pos_3595; Testosterone glucuronide
pos_3597; APC
pos_3608; Campesterol 6'-(9Z-octadecenoyl)-glucoside
pos_3609; Hv-NCC-1
pos_361; R-4-benzyl-3-((R)-3-hydroxy-2,2-dimethyloctanoyl)-5,5-dimethyloxazolidin-2-[illegible]
pos_3623; Armillarin
pos_3632; N-[(4E,8E)-1,3-dihydroxyoctadeca-4,8-dien-2-yl]hexadecanamide
pos_3636; 15-Acetoxyscirpene-3,4-diol 4-O-a-D-glucopyranoside
pos_3638; Phenformin

pos_3650; 3-Hydroxyadipic acid 3,6-lactone
pos_3651; Glyoxylic acid
pos_3659; 2,4-Dimethyloxazole
pos_3675; 2-(Arabinosylamino)-3-(glucosylamino)propanenitrile
pos_3676; CDP-DG(18:0/22:3(10Z,13Z,16Z))
pos_369; Benzarone
pos_3700; (Z)-13-Hexadecenoic acid
pos_3738; PG(16:1(9Z)/16:1(9Z))
pos_3742; 9-Hydroxy-10-O-D-glucuronoside-12Z-octadecenoate
pos_3743; (24E)-3beta,15alpha,22S-Triacetoxylanosta-7,9(11),24-trien-26-oic acid
pos_3748; Rigin
pos_375; Mayolene-18
pos_3752; Gamma-Glutamyltyrosine
pos_3759; Thioguanosine 5'-diphosphate
pos_3760; L-4-Chlorotryptophan
pos_3765; Genipic acid
pos_3779; 12-O-D-Glucuronoside-13-hydroxyoctadec-9Z-enoate
pos_3785; Diflunisal
pos_3795; 1-Butanethiol
pos_3797; Octahydro-2,5,5,8a-tetramethyl-7H-1-benzopyran-7-one
pos_380; 4-O-Methylpinosylvic acid
pos_3801; Ganglioside GA2 (d18:1/9Z-18:1)
pos_3805; TG(14:0/14:1(9Z)/22:2(13Z,16Z))
pos_3806; PE(14:1(9Z)/16:1(9Z))
pos_3807; Bepotastine
pos_3808; Jujuboside B
pos_3827; PC(22:1(13Z)/24:0)
pos_3834; PI(16:0/22:4(10Z,13Z,16Z,19Z))
pos_3854; 3-Phenylpropyl isovalerate
pos_3860; 2,3-Dihydrowithanolide E
pos_3864; Vilazodone
pos_3893; cis-3-Chloroallyl aldehyde
pos_3896; Stearidonyl carnitine
pos_3897; Menthone 1,2-glyceryl ketal
pos_3907; Cer(d18:0/22:1(13Z))
pos_3928; Celastrol
pos_393; Sphinganine
pos_3935; Calenduloside G methyl ester
pos_3936; (1R,4R,5S)-5-Hydroxyfenchone glucoside
pos_3942; (Z)-Resveratrol 3,4'-diglucoside
pos_3952; 19alpha-19-Hydroxy-3,11-dioxo-12-ursen-28-oic acid
pos_3954; Chloroxine
pos_3957; 3-Pyridylacetic acid
pos_3960; 2-Tetradecanone
pos_3963; Irinotecan
pos_3964; Vindesine
pos_3975; Mabiogenin 3-[rhamnosyl-(1->6)-[glucosyl-(1->2)]-glucoside]
pos_3979; 2-Ethylacrylic acid
pos_3988; N-(1-Deoxy-1-fructosyl)threonine
pos_3991; PE(20:1(11Z)/P-18:1(11Z))
pos_3994; TG(14:1(9Z)/20:3(5Z,8Z,11Z)/22:5(7Z,10Z,13Z,16Z,19Z))
pos_3996; 1-Acetyl-2-methylcyclopentene
pos_40; PE(17:0/0:0)
pos_4002; Hydroxyisonobilin
pos_4011; Gibberellin A45
pos_4015; CPA(18:2(9Z,12Z)/0:0)
pos_4016; Azukisaponin I
pos_4018; Shikimic acid

pos_402; PUROMYCIN
pos_4023; MG(16:0/0:0/0:0)
pos_4024; PE(14:1(9Z)/15:0)
pos_4026; N-lactoyl-Methionine
pos_4028; 3-Methyluridine
pos_403; Rhodovibrin
pos_4034; Fevicordin B 2-gentiobioside
pos_4039; Ethambutol
pos_404; Aricine
pos_4045; Faradiol myristate
pos_405; 汞-Tocotrienol
pos_4054; (1R*,3R*,3'S*)-1,2,3,4-Tetrahydro-1-(2-thio-3-pyrrolidinyl)-beta-carboline-3-
pos_406; 4-Hydroxyquinoline
pos_4062; 9-(2-Carboxyphenyl)-3,6-bis(diethylamino)xanthylium(1+)
pos_4071; Aegelinol
pos_4093; 24-Oxo-1alpha,25-dihydroxyvitamin D3
pos_4094; 2-(5-Methyl-2-furanyl)piperidine
pos_4096; Tirofiban
pos_4118; Sanguisorbic acid dilactone
pos_4121; Hydroxyprolyl-Asparagine
pos_4126; Histidinyl-Cysteine
pos_4127; Poppy acid
pos_4132; 1'-O-Acetylpaxilline
pos_4142; Trimeprazine
pos_4147; psi-Pelletierine
pos_4168; Ethyl 3-methyl-9H-carbazole-9-carboxylate
pos_417; 3-Formyl-4,6-dihydroxy-2-methoxy-5-methylchalcone
pos_4173; 3-Methylcyclopentadecanone
pos_418; desethyletomidate
pos_4188; (N-acetylneuraminosyl(a2-6)lactosamine)
pos_4189; 7-Hydroxy-2-methyl-4-oxo-4H-1-benzopyran-5-acetic acid
pos_4204; (E)-10-Hydroxy-2-decene-4,6-diynoic acid
pos_4205; Ascorbic acid
pos_4207; Erinapyrone C
pos_421; p-Mentha-2,4(8)-dien-9,3-olide
pos_4212; Suxibuzone
pos_4213; Telithromycin
pos_4228; Vanillin 1,2-butylene glycol acetal
pos_4245; TG(22:5(4Z,7Z,10Z,13Z,16Z)/22:5(4Z,7Z,10Z,13Z,16Z)/22:6(4Z,7Z,10Z,13Z
pos_4247; Ganglioside GM3 (d18:1/14:0)
pos_4253; Hebevinoside XIII
pos_4254; 4-Methyl-2-methylene-1-(1methylethylidene)-cyclohexane
pos_4258; Histamine
pos_4261; Secaloside A
pos_4262; L-dopachromate
pos_4264; Gossypetin 8-glucoside 3-sulfate
pos_4265; Curcumin II
pos_4269; Erythrityl Tetranitrate
pos_4273; TG(14:0/22:4(7Z,10Z,13Z,16Z)/o-18:0)
pos_4280; 7-Hexadecenoic acid, methyl ester
pos_4281; Galabiosylceramide (d18:1/24:0)
pos_4283; Olanzapine
pos_4287; Dinitolmide
pos_4292; 2-Oxo-4-methylthiobutanoic acid
pos_4295; 2-methyl-1,3-thiazolidine-2-carboxamide
pos_4303; 4,4-Dimethyl-5a-cholesta-8-en-3b-ol
pos_4307; Thiophene
pos_4309; 2-Hydroxyundecanoate

pos_4313; Xanthochymol
pos_4322; TG(8:0/14:0/10:0)
pos_4323; (5E)-6-Oxo-5-(phenylhydrazono)-5,6-dihydro-2-naphthalenesulfonic acid
pos_4327; Lucyoside J
pos_4336; Hexacosanoic acid
pos_4343; 3-(10-Heptadecenyl)phenol
pos_4356; 16-hydroxy hexadecanoic acid
pos_4377; Glycerol 1-myristate
pos_4381; Phenylalanyl-Histidine
pos_440; Vinyl caffeate
pos_4400; Baccatin III
pos_4402; dodecanamide
pos_4403; Estradiol Cypionate
pos_4409; Caffeine
pos_4413; DL-Methionine sulfoxide
pos_4414; Helenalin
pos_4415; 5alpha-Androstane-3,17-dione
pos_4420; .beta.-Cryptoxanthin
pos_4425; Azacitidine
pos_4431; cis-4-Hydroxy-D-proline
pos_4432; 3-Methyl-2-oxopentanoate
pos_446; 11-Oxo-beta-amyrin
pos_463; 3',4',5'-Trimethoxycinnamyl alcohol acetate
pos_468; PC(6:0/6:0)
pos_471; Herculin
pos_473; Bombykol
pos_475; Gly Pro Gly Asp
pos_485; 2',3',5'-triacetyl-5-Azacytidine
pos_489; 3,7,11,15-Tetramethyl-6,10,14-hexadecatrien-1-ol
pos_490; 4,7,10,13-Docosatetraynoic acid
pos_492; 2-Methylpropanal O-methyloxime
pos_5; Uracil
pos_504; 3''-Hydroxy-geranylhydroquinone
pos_518; Propapyriogenin A2
pos_526; 2-hydroxyestradiol
pos_527; LEVULINIC ACID, 3-BENZYLIDENYL-
pos_531; 12-amino-octadecanoic acid
pos_535; Gly Gln Trp Pro
pos_536; Gestodene metabolite 4
pos_537; 5-Aminosalicylic Acid
pos_541; PC(24:0/20:2(11Z,14Z))
pos_544; azido-FTY720
pos_549; Pro Ile Phe Ile
pos_559; Asp Pro Ile
pos_564; 22-Deoxocucurbitacin D
pos_57; PE(16:0/0:0)
pos_571; 1-Nitronaphthalene-7,8-oxide
pos_572; Glutathione amide
pos_575; alpha-Santalyl acetate
pos_579; beta-Hydroxy-2',6'-dimethoxy-3',4'-methylenedioxydihydrochalcone
pos_588; 3-Dimethylallyl-4-hydroxymandelic acid
pos_592; Morpholine
pos_595; Polyoxyethylene (600) mono- ricinoleate
pos_596; Ricinoleic Acid methyl ester
pos_60; Guanine
pos_601; Norsanguinine
pos_603; Bisindolylmaleimide IV
pos_608; Glu Ile Leu Ile

pos_609; PI(21:0/16:0)
pos_610; (17Z)-1伪,25-dihydroxy-26,27-dimethyl-17,20,22,22,23,23-hexadehydrovitami
pos_615; 14-METHOXY-4,4-BISNOR-4,8,11,13-PODOCARPATETRAEN-3-ONE
pos_617; 8-Hydroxyalanylclavam
pos_618; Methyl(2-furoylamino)acetic acid
pos_628; 3-(2-chloro-10H-phenothiazin-10-yl)propan-1-amine
pos_631; U-46619
pos_635; Tylactone
pos_64; Mandelonitrile
pos_644; Ergoline-1,8-dimethanol, 10-methoxy-, (8b)-
pos_648; 2-docosanamidoethanesulfonic acid
pos_657; 16-oxo-octadecanoic acid
pos_664; Ser Asp Thr Leu
pos_675; Leu Leu Met Ile
pos_68; 4E,6E,11Z-Hexadecatrienal
pos_681; N-Carboxyethyl-纬-aminobutyric acid
pos_685; Cys Tyr Glu Trp
pos_686; 4,2'-Dihydroxychalcone 4-glucoside
pos_691; 3,5-diethyloctahydroindolizine
pos_693; (22S)-1伪,22,25-trihydroxy-26,27-dimethyl-23,23,24,24-tetradehydrovitamin D
pos_694; Thujyl 19-trachylobanoate
pos_700; Gly Asp Lys
pos_707; 1,2,4,5-Tetrahydrotestolactone
pos_712; (卤)-Dulciol E
pos_718; 4-tert-Octylphenol
pos_720; Phenolic steroid
pos_735; Thr Ile His Ile
pos_736; Dihydroshikonofuran
pos_737; (23R)-23,25-dihydroxyvitamin D3 / (23R)-23,25-dihydroxycholecalciferol
pos_74; cis-12-Octadecenoic Acid methyl ester
pos_740; Edulan I
pos_747; 6-Deoxyjacareubin
pos_75; Pfaffic acid
pos_750; Quinaprilat
pos_756; Gly Gly Trp
pos_759; 3-Hydroxy-1-phenyl-1-heneicosanone
pos_769; 2-Oxo-7-methylthioheptanoic acid
pos_77; (6alpha,22E)-6-Hydroxy-4,7,22-ergostatrien-3-one
pos_775; YM-53601
pos_782; Gly Ala Lys Trp
pos_79; Ectocarpen
pos_801; Geranylhydroquinone
pos_802; Elaidamide
pos_812; Tyr Leu Gly
pos_815; N-methyl arachidonoyl amine
pos_828; Ergosterol
pos_83; 1伪,25-dihydroxy-11伪-[(1R)-oxiranyl]vitamin D3 / 1伪,25-dihydroxy-11伪-[(1
pos_831; His Pro Trp Trp
pos_835; HECOGENIN ACETATE
pos_839; Pro Leu Arg Arg
pos_842; 2-Amino-3-methoxy-benzoic acid
pos_845; Nookatone
pos_851; Azaspiracid 4
pos_852; 2(R)-hydroxydocosanoic acid
pos_855; Dodecanal dimethyl acetal
pos_856; 13-Heptadecyn-1-ol
pos_859; Dioscorine
pos_86; Alisol C

pos_860; p-Hydroxynonanophenone
pos_862; 2-Hydroxy-desipramine glucuronide
pos_869; (3beta,5alpha,9alpha,22E,24R)-5,9-Epidioxy-3-hydroxyergosta-7,22-dien-6-one
pos_870; 2-(1,2,3,4-Tetrahydroxybutyl)-6-(2,3,4-trihydroxybutyl)pyrazine
pos_874; 4-Hydroxybenzyl alcohol
pos_875; Cyclandelate
pos_876; (E)-6,10-Dimethyl-9-methylene-5-undecen-2-one
pos_878; cis-3-Hexenyl pentanoate
pos_882; Monoethylhexyl phthalic acid
pos_884; 2,2,4,4,-Tetramethyl-6-(1-oxopropyl)-1,3,5-cyclohexanetrione
pos_888; 1-(2,3-Dihydro-1H-pyrrolizin-5-yl)-2-propen-1-one
pos_891; N-Methyltyramine
pos_893; (E)-5,8-Megastigmadien-4-one
pos_9; D-(+)-Trehalose dihydrate
pos_900; 5,10-Pentadecadien-1-ol
pos_901; Behenoylglycine
pos_902; Bilirubin
pos_909; Curlone
pos_91; D-erythro-Sphingosine C-17
pos_922; 3-Hydroxy-5Z-octenyl acetate
pos_93; 1伪,25-dihydroxy-24-nor-22-oxavitamin D3 / 1伪,25-dihydroxy-24-nor-22-oxacl
pos_931; 10-Oxo-11-octadecen-13-olide
pos_932; Ovalicin
pos_941; Niazinin A
pos_943; Cryptoxanthin diepoxide
pos_945; 1,4-Ipomeadiol
pos_953; Dihydroceramide
pos_954; S-Carboxymethyl-L-cysteine
pos_960; Cappariloside A
pos_963; (S)-a-Amino-2,5-dihydro-5-oxo-4-isoxazolepropanoic acid N2-glucoside
pos_968; N-Desmethylterbinafine
pos_970; 3-hydroxypristanic acid
pos_973; 5-Acetylamino-6-amino-3-methyluracil
pos_983; Tricycloekasantalol
pos_988; Gynocardin
pos_999; L-alpha-Amino-1H-pyrrole-1-hexanoic acid

2,22,23,23-hexadehydrovitamin D3 / (17Z)-1伪,25-dihydroxy-26,27-dimethyl-17,20,22,22,23,23-hexadehydrochole

calciferol
