# Supplementary material for: Angelica polysaccharides relieve blood glucose levels in diabetic KKAy mice possibly by modulating gut microbiota: an integrated gut microbiota and metabolism analysis
Source: BMC Microbiol. 2023 Oct 3;23:281. doi: 10.1186/s12866-023-03029-y (PMC10546737; doi:10.1186/s12866-023-03029-y)
Supplement: Supplementary file 8 — Additional file 8: Supplementary Table 5. The enrichment analysis of differential metabolites between HDF and ASP groups. [file 12866_2023_3029_MOESM8_ESM.docx]

Supplementary Table 5. The enrichment analysis of differential metabolites between HDF and ASP groups.

| Kegg_pathway | ko_id | rich_factor |
| --- | --- | --- |
| Sulfur relay system | ko04122 | 10.11538 |
| Penicillin and cephalosporin biosynthesis | ko00311 | 10.11538 |
| Pentose phosphate pathway | ko00030 | 8.092308 |
| Thiamine metabolism | ko00730 | 6.74359 |
| Starch and sucrose metabolism | ko00500 | 6.74359 |
| Sulfur metabolism | ko00920 | 5.057692 |
| Terpenoid backbone biosynthesis | ko00900 | 5.057692 |
| Glycerolipid metabolism | ko00561 | 5.057692 |
| Valine, leucine and isoleucine degradation | ko00280 | 5.057692 |
| Phosphonate and phosphinate metabolism | ko00440 | 4.046154 |
| Valine, leucine and isoleucine biosynthesis | ko00290 | 4.046154 |
| Fatty acid degradation | ko00071 | 4.046154 |
| Pantothenate and CoA biosynthesis | ko00770 | 4.046154 |
| Fatty acid metabolism | ko01212 | 4.046154 |
| Retinol metabolism | ko00830 | 4.046154 |
| Glycine, serine and threonine metabolism | ko00260 | 4.046154 |
| Biotin metabolism | ko00780 | 3.371795 |
| Lysine biosynthesis | ko00300 | 3.371795 |
| Carbon metabolism | ko01200 | 3.194332 |
| alpha-Linolenic acid metabolism | ko00592 | 3.112426 |
| Chemical carcinogenesis | ko05204 | 2.89011 |
| Ether lipid metabolism | ko00565 | 2.89011 |
| Biosynthesis of amino acids | ko01230 | 2.809829 |
| Aminoacyl-tRNA biosynthesis | ko00970 | 2.528846 |
| Metabolism of xenobiotics by cytochrome P450 | ko00980 | 2.528846 |
| Taurine and hypotaurine metabolism | ko00430 | 2.247863 |
| Linoleic acid metabolism | ko00591 | 2.023077 |
| Protein digestion and absorption | ko04974 | 2.023077 |
| Biosynthesis of unsaturated fatty acids | ko01040 | 2.023077 |
| Ferroptosis | ko04216 | 2.023077 |
| Mineral absorption | ko04978 | 1.839161 |
| Drug metabolism - other enzymes | ko00983 | 1.685897 |
| Central carbon metabolism in cancer | ko05230 | 1.685897 |
| Glutathione metabolism | ko00480 | 1.685897 |
| ABC transporters | ko02010 | 1.685897 |
| Glyoxylate and dicarboxylate metabolism | ko00630 | 1.685897 |
| Glycerophospholipid metabolism | ko00564 | 1.556213 |
| Retrograde endocannabinoid signaling | ko04723 | 1.556213 |
| Lysine degradation | ko00310 | 1.348718 |
| Cysteine and methionine metabolism | ko00270 | 1.348718 |
| Nicotinate and nicotinamide metabolism | ko00760 | 1.348718 |
| Arachidonic acid metabolism | ko00590 | 1.064777 |
| Choline metabolism in cancer | ko05231 | 1.011538 |
| Arginine and proline metabolism | ko00330 | 0.96337 |
| 2-Oxocarboxylic acid metabolism | ko01210 | 0.91958 |
| Ubiquinone and other terpenoid-quinone biosynthesis | ko00130 | 0.842949 |
| Tryptophan metabolism | ko00380 | 0.842949 |
| Purine metabolism | ko00230 | 0.778107 |
| Steroid hormone biosynthesis | ko00140 | 0.674359 |
| Bile secretion | ko04976 | 0.396682 |
